# Study on the community structure and function of symbiotic bacteria from different growth and developmental stages of *Hypsizygus marmoreus*

**DOI:** 10.1186/s12866-020-01998-y

**Published:** 2020-10-14

**Authors:** Shujing Sun, Fan Li, Xin Xu, Yunchao Liu, Xuqiang Kong, Jianqiu Chen, Ting Liu, Liding Chen

**Affiliations:** College of Life Sciences, Fujian Agriculture and Forestry University, Fuzhou, 350002 People’s Republic of China

**Keywords:** Community structure and function, GFP labeling, High-throughput sequencing, *Hypsizygus marmoreus*, *Serratia odorifera*, Symbiotic bacteria

## Abstract

**Background:**

The symbiotic bacteria associated with edible fungi are valuable microbial resources worthy of in-depth exploration. It is important to analyze the community structure and succession of symbiotic bacteria in mushrooms. This can assist in the isolation of growth-promoting strains that have an essential relationship with the cultivation cycle as well as the agronomic traits and yields of fruiting bodies.

**Results:**

In all of the samples from cultivation bags of *Hypsizygus marmoreus*, 34 bacterial phyla were detected. Firmicutes was *the most abundant* bacterial phylum (78.85%). The genus Serratia showed an exponential increase in *abundance* in samples collected from the cultivation bags in the mature period, reaching a peak abundance of 55.74% and the dominant symbiotic flora. The most predominant strain was *Serratia odorifera* HZSO-1, and its abundance increased with the amount of hyphae of *H. marmoreus*. *Serratia odorifera* HZSO-1 could reside in the hyphae of *H. marmoreus*, promote growth and development, shorten the fruiting cycle by 3–4 days, and further increase the fruiting body yield by 12%.

**Conclusions:**

This study is a pioneering demonstration of the community structure of the symbiotic microbiota and bacteria-mushroom interaction in the growth and development of edible fungi. This work lays a theoretical foundation to improve the industrial production of mushrooms with symbiotic bacteria as assisting agents.

## Background

*Hypsizygus marmoreus* has excellent potential for commercialization because of its remarkable flavor, delicious taste, nutritional value, and medicinal properties [1, 2]. However, *H. marmoreus* requires *a long* ripening *period* to achieve a high *fruiting body yield*, which inevitably results in a very long production cycle [3]. In the commercial cultivation of *H. marmoreus*, it generally takes 4–5 months to go from inoculation to fruiting body picking [4], which has severely limited the industrialized production of *H. marmoreus*. Therefore, it is necessary to develop new cultivation methods to shorten the production cycle of *H. marmoreus* and improve economic efficiency.

With the advancement of microecological research [5–7], many resources have been made available which show the beneficial relationship between microorganisms and mushrooms [8], the environment [9] and human health [10]. This provides excellent references for studying the beneficial microorganisms of edible fungi. It is well known that during the growth and development of edible fungi, environmental factors such as light [2], CO_2_ concentration, temperature, and humidity play a critical role. In addition to the influence of environmental factors [11], biological factors can also be a pivotal part of the production cycle of edible fungi. As hosts, edible fungi interact with many beneficial microorganisms [12]. These beneficial microorganisms play vital roles in the different growth and developmental stages of edible fungi.

Research on mushroom-symbiotic microorganisms has shown that there are many bacteria (including thermophilic bacteria, cellulolytic actinomycetes, and bacilli) which have been identified as having the ability to promote hyphal extension and increase compost productivity by accelerating the progressive breakdown of lignocellulose [13]. During the growth of *Agaricus bisporus*, *Pseudomonas putida* was identified as a critical microorganism responsible for breaking down the 1-octen-3-ol bond and inducing fruiting body formation [14, 15]. Zarenejad’s research showed that *P. putida* was the best growth-promoting inoculant among 23 tested bacterial strains that could increase the mushroom yield [16]. Fifty-six bacteria were isolated from the casing soil of *Agaricus blazei*. Diversity analysis revealed the relative abundance of these identified microbes: actinomycetes 60%, firmicutes 20%, and proteobacteria 20%. Most of these bacteria can promote mycelial growth, the synthesis of polysaccharide-protein complexes, and the fruiting body productivity of *Agaricus blazei* [17]. Furthermore, some researchers have reported that *Pseudomonas fluorescens* strains could promote the formation of the primordium and enhance the development of the fruiting body of *Pleurotus eryngii* [18] and *Pleurotus ostreatus* [19].

The literature indicates that the interaction between edible fungi and beneficial microorganisms is universal and that promotion of the production of edible mushrooms by adding beneficial microbes to the cultivation substrate is a feasible practice. The microbes mentioned above that interact positively with edible fungi are primarily from the open environment, such as compost and casing soil. Fungi and bacteria are found living together in a wide variety of environments. Increasingly, it is being recognized that symbiotic interactions between mushrooms and bacteria under controlled conditions can contribute to edible fungi productivity. Hence, it is necessary to perform some research on the community structure and succession of microorganism population in steam-sterilized bags filled with substrates after inoculation in the sterilized environment, which will increase our understanding of how these microorganisms interact with fungal hyphae and influence the growth and fructification of cultivated mushrooms. This will also result in the isolation of some growth-promoting strains closely related to the cultivation cycle, and agronomic traits and yields of fruiting bodies. They may be developed into useful agronomical amendments to increase mushroom productivity through growth promotion. Besides, a *new theoretical basis for* studying the symbiosis of microorganisms with mushrooms and will guide the development of novel microbial agents to improve the yield and quality of mushrooms in commercial production.

In an attempt to understand the microbial ecological distribution in cultivation bags of the edible fungi *H. marmoreus* at different growth and developmental stages, V3-V4 of 16S rRNA genes from various bacteria were sequenced by Illumina HiSeq 2500. After analysis of the dynamics and succession of symbiotic bacteria, the correlation between symbiotic bacteria and *H. marmoreus* was revealed. Then, growth-promoting bacteria were isolated from cultivation bags during the specific growth period of *H. marmoreus* and *inoculated* into the *cultivation* substrate to verify their growth-promoting effects*.* It is the first study to reveal the dynamics and succession of symbiotic bacteria in cultivation bags of *H. marmoreus* with Illumina sequencing techniques, presenting scientific evidence for genetic breeding and *high-efficiency cultivation of edible fungi.*

## Results

### Species composition and changes in symbiotic bacteria in *H. marmoreus* hyphae

**Fig. 1:**
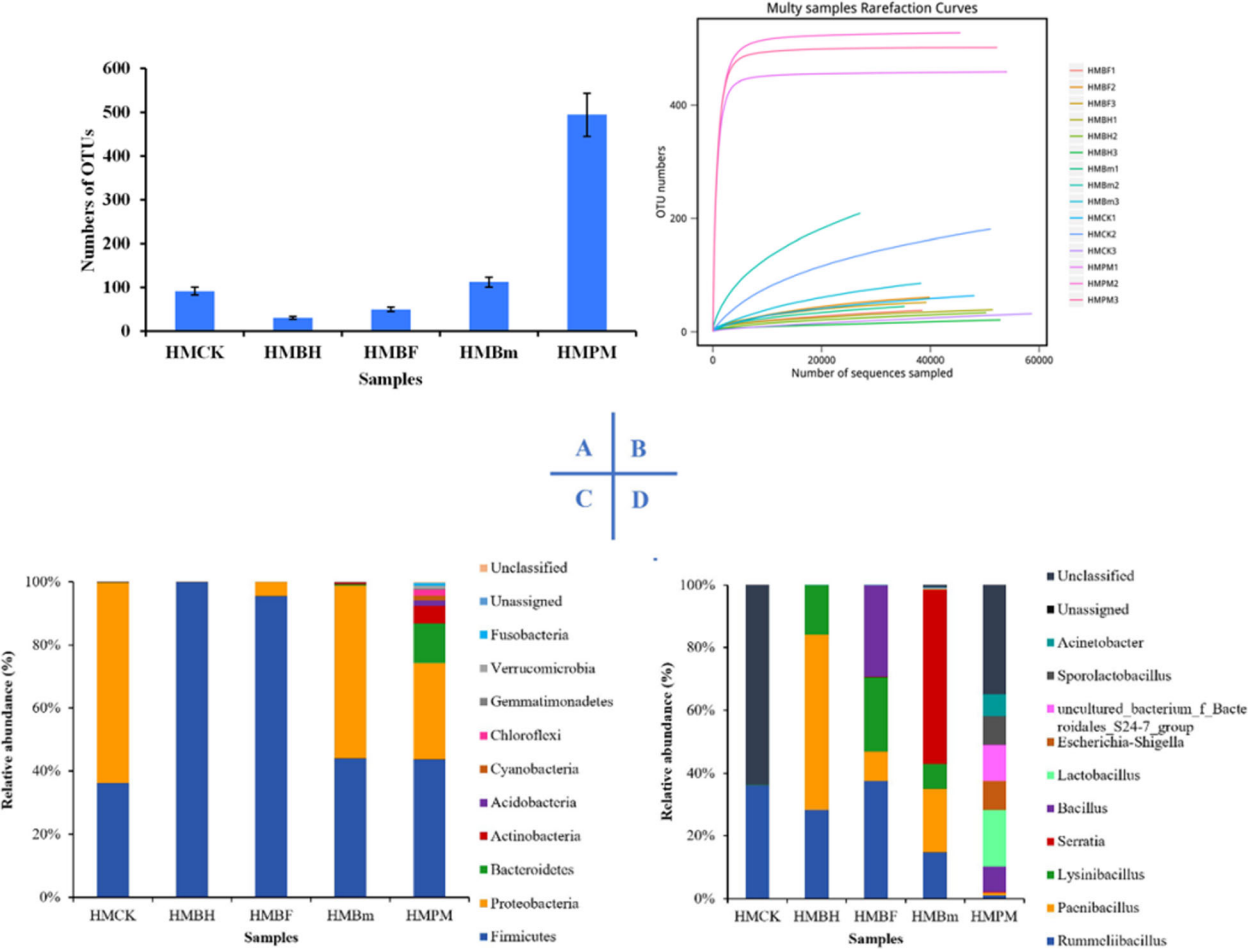
Numbers of OTUs distribution (**a**), multy samples rarefaction curves (**b**), *species* composition abundance at the phylum level (**c**), and species composition abundance at the genus level (**d**) in 15 samples collected from hyphae grown on PDA plates and in cultivation bags at different growth and developmental stages of *H. marmoreus.* HMCK: blank bag; HMBH: half bag period; HMBF: full bag period; HMBm: mature period; HMPM: hyphae *grown on* agar plates. The letters a and b represent the significantly different groups

Hyphal samples collected from plate culture and cultivation bags at different growth stages of *H. marmoreus* were sequenced using an Illumina HiSeq 2500 after total DNA extraction. After paired-end assembly, quality filtering, and the removal of chimeric reads, a total of 827,880 effective tags out of 983,157 pairs of reads from five different samples were obtained and *clustered* into 781 *OTUs* (Fig. 1a and Table S2) based on their sequence similarity at 97%. These tags were assigned to 34 phyla, 79 classes, 127 orders, 228 families, 417 genera, and 289 species. A comparison of the OTUs in the five groups indicated that the hyphae samples collected from plate culture shared 495 OTUs; this was significantly higher than the *number of* OTUs shared by other samples. There were no significant differences in the *number of* OTUs among other samples collected from cultivation bags. Besides, an increase in OTU abundance (Fig. 1a) was observed with the positive growth and development of *H. marmoreus*, implying that the abundance of bacteria was positively correlated with the number of hyphae (Figs. 1 and 7 c, d and c-e).

As shown in Fig. 1b, the number of OTUs in the rarefraction curves of 15 samples increased with the number of sequences, it is only the HMPM sample that reached saturation though the coverage rate was reasonable and sufficient to characterize the bacterial community structure and diversity in the samples. The OTU composition at the phylum taxonomic level is shown in Fig. 1c; 34 bacterial phyla were detected in the five samples. The 5 most abundant phyla were: Firmicutes (78.85%), Proteobacteria (15.08%), Bacteroidetes (2.68%), Actinobacteria (1.29%), and Acidobacteria (0.37%). Firmicutes was *the most abundant* bacterial phylum in the samples collected from cultivation bags, and bacteria involved in this phylum belonged to the class Bacilli. The abundance of Firmicutes bacteria decreased gradually from the half-bag period to the mature period. Proteobacteria was the second most common microbial phylum, mainly including: γ- Proteobacteria (11.34%), α- Proteobacteria (1.87%), and β- Proteobacteria (1.38%). The abundance of these bacteria increased and reached a peak (23.57%) in the mature period of *H. marmoreus* growth. Additionally, at the phylum level (Fig. 1c), the bacterial community (mainly including Sporolactobacillus, Lactobacillus, Bacillus, Serratia, Lysinibacillus) in the hyphae grown on PDA plates was more abundant than that in mycelia collected from cultivation bags.

At the genus level (Fig. 1d), only Rummeliibacillus, Lysinibacillus, Acinetobacter, and some unknown flora were present in the blank bags. Compared with control bags, the number of bacterial genera in the cultivation bags increased from the half-bag period to the mature period. The genera Rummeliibacillus, Paenibacillus, and Lysinibacillus were the dominant flora, accounting for 40.97, 23.03, and 21.98% of the total, respectively. These three bacterial genera belong to the Bacillales order. The abundance of the genus Serratia increased exponentially in cultivation bags in the mature period, and the relative abundance reached 55.74%, in contrast to the abundance (< 1%) during the period from half bag to full bag.

### Alpha and beta diversity analysis of the bacterial community

Table 1 shows data from the calculations of the average alpha diversity index of the five samples *at a similarity level of 97*%. *It* was found that the ACE and Chao 1 indices were significantly higher in the hyphae grown on PDA plates than in samples collected from cultivation bags, suggesting that there was a greater abundance of bacteria in the hyphae grown on agar plates than in the samples collected from cultivation bags. The Simpson index values were lowest in the hyphae grown on agar plates. Still, the opposite trend was observed for the Shannon index, which indicated that the bacterial diversity was significantly higher in the hyphal sample than in the samples collected from cultivation bags. Among the alpha diversity indices calculated for the different growth periods of *H. marmoreus*, the ACE, Chao, and Shannon indices showed a gradual increase with the growth of hyphae until their *maximum values*. Similarly, a *comparison of the Simpson index in* different *samples showed that the value in t*he full bag period was lower than those in the half bag period and the mature period, indicating that the microbial diversity in the full bag period reached a relatively stable state.

**Table 1:** Statistics of the average alpha diversity index

Samples	ACE	Chao 1	Simpson	Shannon	OTUs	Coverage value
HMCK	174.8341 ± 92.16a	177.4167 ± 110.11a	0.8284 ± 0.2576a	0.1005 ± 0.1063a	92	0.9991
HMBH	84.3187 ± 32.97a	56.0833 ± 19.71a	0.4271 ± 0.099b	0.9893 ± 0.1522b	31	0.9997
HMBF	86.3041 ± 21.18a	64.7792 ± 6.86a	0.3826 ± 0.1047b	1.1335 ± 0.3026b	50	0.9995
HMBm	218.0776 ± 166.19a	176.0830 ± 135.79a	0.4349 ± 0.126b	1.1955 ± 0.3128b	113	0.9980
HMPM	496.6860 ± 34.63b	497.4444 ± 34.80b	0.0099 ± 0.0012c	5.4694 ± 0.0678c	495	0.9999

**Fig. 2:**
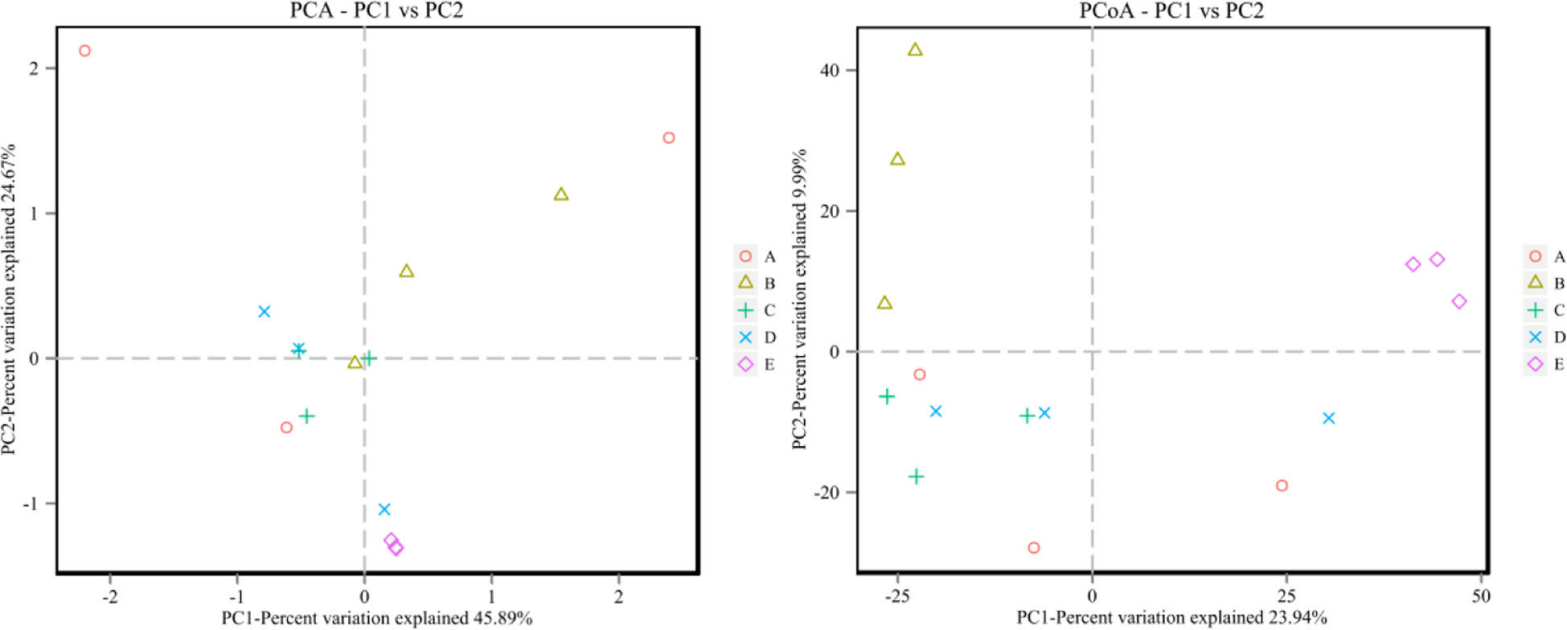
PCA and PCoA of bacterial community beta diversity in five samples. Left panel: PCA using PC1 and PC2 as the main axes. Right Panel: PCoA using PC1 and PC2 as the main axes. **a**: HMCK, blank bag; **b**: HMBH, half bag period; **c**: HMBF, full bag period; **d**: HMBm, mature period; **e**: HMPM, hyphae *grown on* agar plates

The alpha diversity in the cultivation bags differed during the growth and development of *H. marmoreus.* PCA and PCoA analyses were performed to characterize the beta diversity and compare the bacterial community differences between pairs of treatments. As shown in Fig. 2, the main axes 1 (PC1) and 2 (PC2) can explain 45.89 and 24.67% of the variation and 23.94 and 9.99% of the variation, respectively. Notably, three samples of hyphae *grown on* agar plates showed the most overlap and were mainly clustered in the positive-value quadrant. The samples collected from cultivation bags at the HMBH, HMBF, and HMBm periods were mainly clustered in the negative-value quadrant, and their distribution was relatively concentrated. This reflected the small difference between pairs of replicates in the same growth period. The distribution of samples from blank bags was relatively dispersed and had low homogeneity between samples from the same cultivation bag for their mycelium content differences. Therefore, this distribution could reflect the existence of OTUs in these samples to some extent. The distance between samples in different growth periods (HMBH, HMBF, and HMBm) was relatively high, but the distance between samples within the full bag and mature periods was low. *This phenomenon showed that* the OTU compositions of samples among these three periods were substantially different, and those of samples in HMBF and HMBm were very similar. This illustrates that the species of the flora had approached a stable state when the growth of *H. marmoreus* entered the full bag period.

**Fig. 3:**
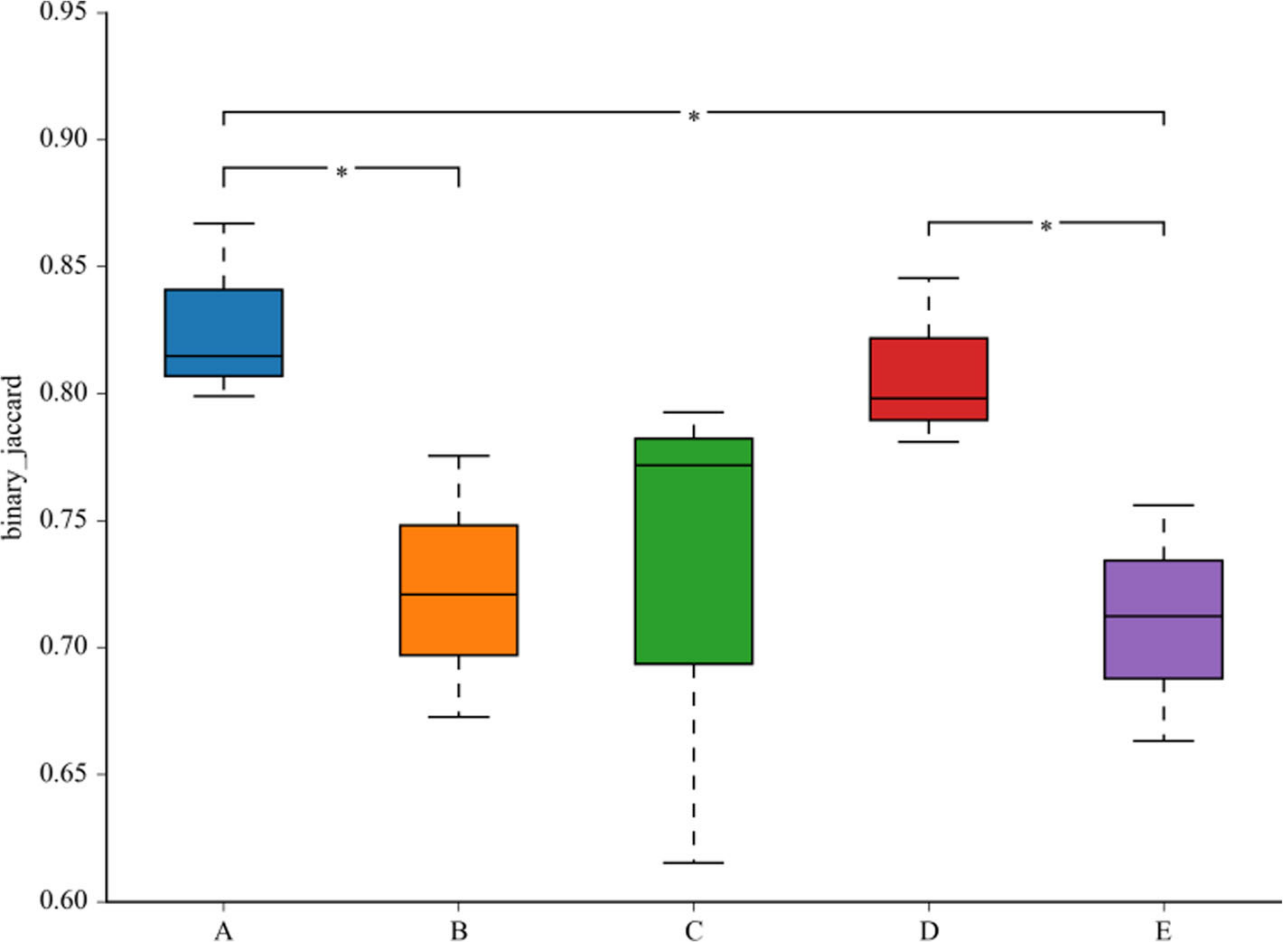
Box plot of beta diversity among different groups

As shown in Fig. 3, the OTU distribution in the hyphae *from* agar plates and cultivation bags in the HMBH period was relatively homogeneous, and the data were close to the mean values of the samples. However, the OTU distribution of samples in the full bag period was highly discrete. Moreover, there was a significant difference in OTU diversity among samples taken from the blank and half-bag periods. Still, there were no statistically significant differences between samples collected from cultivation bags in HMBH, HMBF, and HMBm. This indicates the high similarity in the bacterial community composition of samples from these three periods. These results, combined with the analysis of alpha and beta diversity, showed that the community structure changed with the transition of the growth and development period of *H. marmoreus*. The dominant flora varied from period to period, suggesting the dynamic nature of the process. The number and diversity of symbiotic bacteria in the hyphae *grown on* agar plates were the highest, and those of bacteria from the blank bags were the lowest. There was no significant difference in the composition of symbiotic bacteria among the samples from HMBH, HMBF, and HMBm based on diversity indices and OTUs analysis (Fig. 1a and Table 1). The number of bacteria found in samples in the mature period was higher than that in the HMBH and HMBF periods. Compared to those samples from other cultivation bags, the sequencing data quality of samples from blank bags was not very stable because of the low abundance of bacteria. Besides, the data from the HMPM sample *had excellent repeatability* and accuracy which could reflect the existence of symbiotic bacteria in the samples corresponding to different growth stages.

### Relationship between the community structure of symbiotic bacteria and the growth and development cycle of *H. marmoreus*

**Fig. 4:**
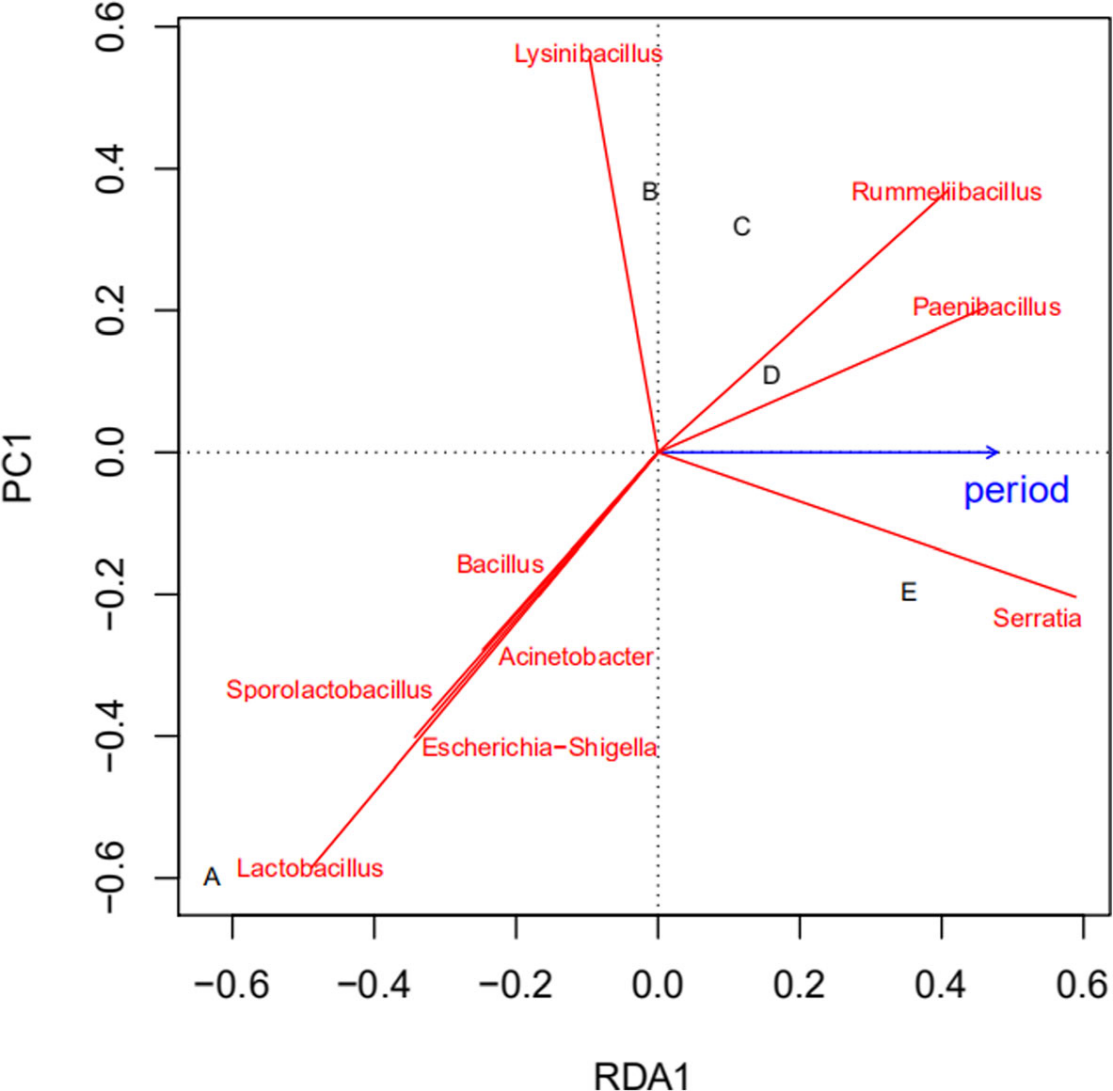
*Redundancy analysis (RDA) results of* the correlations between the community structure profiles of symbiotic bacteria in cultivation bags and the growth stages of *H. marmoreus*. The bacterial species are shown by solid red lines, and the growth period is shown by the solid blue arrow. **a**: hyphae grown *on* an agar plate; **b**: blank bag; **c**: half bag period; **d**: full bag period; **e**: mature period

As shown in Fig. 4, the abundance of the Rummeliibacillus, Paenibacillus, and Serratia genera was positively correlated with the growth cycle of *H. marmoreus*. In contrast, a negative correlation was observed between the abundance of Lysinibacillus, Lactobacillus, Escherichia, Shigella, Acinetobacter, Bacillus, and Sporolactobacillus and the growth cycle of *H. marmoreus*.

### Isolation and identification of dominant symbiotic bacteria and their effects on the growth and development of *H. marmoreus*

**Fig. 5:**
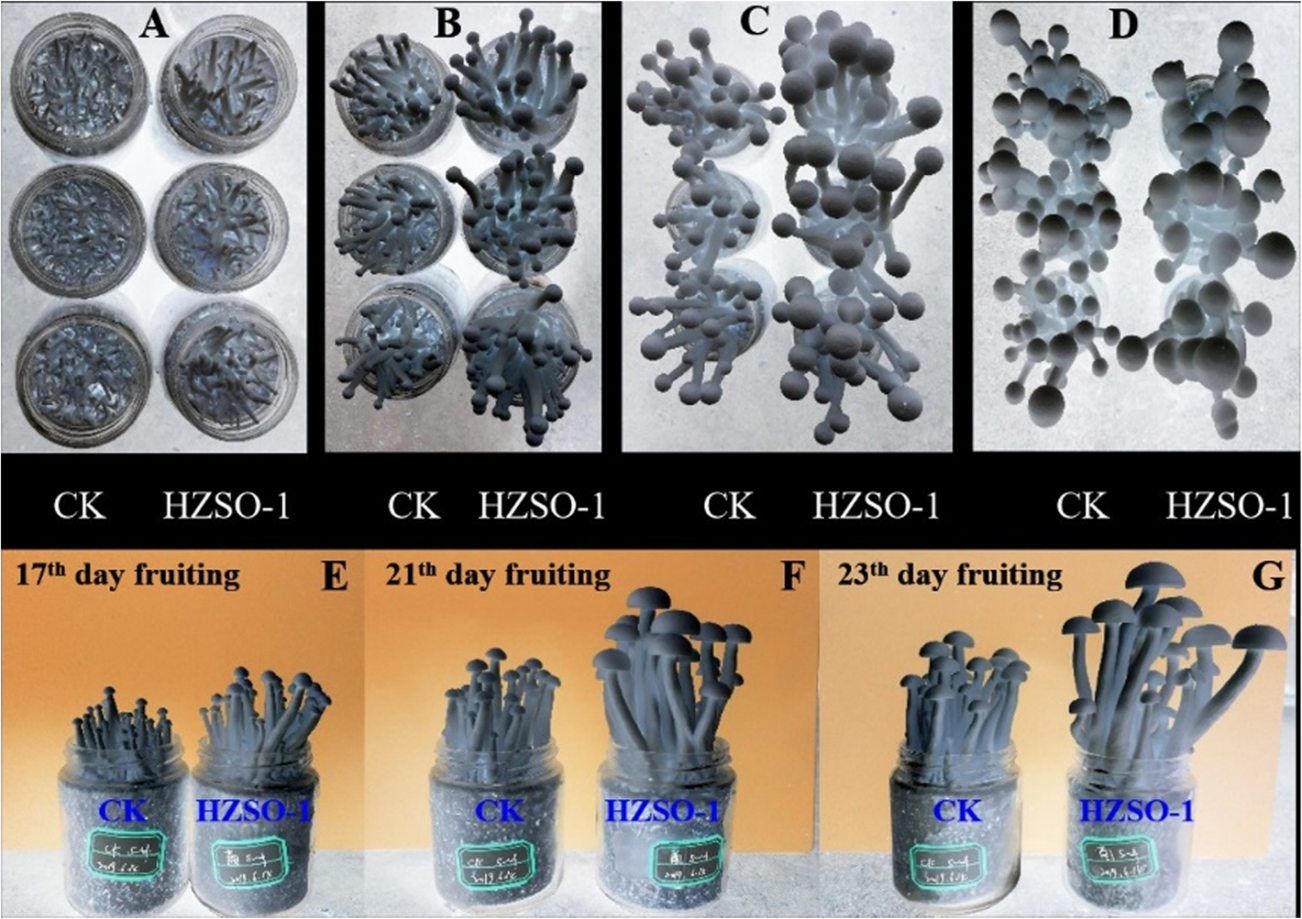
Effects of the addition of sterilized fermentation broth of *S. odorifera* HZSO-1 on the fruiting of *H. marmoreus*, as determined *by bottle cultivation (top-down view* in *the top panel* and straight-on view in the bottom panel*).* **a**: fruiting, 13 days; **b**: fruiting, 17 days; **c**: fruiting, 21 days; **d**: fruiting, 23 days; CK: blank control; HZSO-1: addition of sterile fermentation broth of HZSO-1

With the results of HiSeq analysis as a reference, different symbiotic bacteria were isolated from the samples taken from cultivation bags in the mature period. The obtained strains were identified as *Bacillus altitudinis*, *Bacillus stratosphericus*, *Lysinibacillus massiliensis, Lactobacillus amylovorus*, *Paenibacillus illinoisensis*, and *Serratia odorifera*. Of these bacteria, the dominant strain was *Serratia odorifera* HZSO-1 (NCBI accession number: MN959466.1) (Fig. S1), and its abundance changed considerably during the maturation period of *H. marmoreus*. Therefore, the symbiosis effects of these bacteria on the growth and development of *H. marmoreus* were separately evaluated in detail. A significant growth-promoting effect on the hyphal growth of *H. marmoreus* was observed at the *P* < 0.05 level when 3, 5, and 8 mL of 0.22-μm filter-sterilized fermentation broth of *Serratia odorifera* HZSO-1 was *added to culture plates* (Fig. S2). The growth-promoting effect was found to be optimal when 5 ml of sterilized fermentation broth was added. As shown in Fig. 5, the fruiting bodies of *H. marmoreus* cultivated in substrates supplemented with sterile fermentation broth of *Serratia odorifera* HZSO-1 grew faster than those in the control group. On the 13th day of fruiting, the pileus diameter reached 4–6 mm in the groups treated with the fermentation broth of *Serratia odorifera* HZSO-1. However, many mushroom buds formed in the blank groups were still in the needle tip stage. A comparison of the agronomic traits of fruiting bodies in the control and treatment groups was performed and the results are summarized in Tables 2 and 3. On the 21st day of fruiting, the fruiting bodies in the treatment groups reached the mature state with a stalk height of over 9 cm. The fruiting bodies were plump, and the early opening of the fruit body did not occur. However, mushroom buds in the blank group were only approximately 7 cm long and still in the immature stage. On the 23rd day of fruiting, the fruiting bodies in the blank group had just entered the mature phase, while the aging of fruiting bodies in the treatment groups occurred with the early opening of the fruit body. The addition of sterilized fermentation broth of *Serratia odorifera* HZSO-1 in the substrates significantly promoted the growth of the *H. marmoreus* fruiting body, shortened the fruiting cycle, and brought forward the harvest time by 3–4 days. Generally, the metabolites produced by *Serratia odorifera* HZSO-1 contain quorum sensing molecules as described in our published paper [20], which can stimulate *H. marmoreus* to secret some lignin-degrading enzymes (laccase, peroxidase, cellulase, and chitinase). These enzymes accelerated the decomposition of cultivation substrates, promoted the growth and development of *H. marmoreus*, and further increase yield of fruiting bodies.

**Table 2:** Agronomic characters comparison of *H. marmoreus* fruiting bodies (1)

Name of Samples	Pileus diameter (mm)	Pileus thickness (mm)	Stipe length (cm)	Stipe diameter (mm)
21d-CK	9.50 ± 0.8a	5.07 ± 0.4a	7.66 ± 0.4a	6.29 ± 0.55a
21d-HZSO-1	15.62 ± 1.71b	9.01 ± 1.27c	9.96 ± 0.88c	6.94 ± 1.06a
23d-CK	15.46 ± 2.66b	8.02 ± 1.23b	8.63 ± 0.8b	6.23 ± 0.72a
23d-HZSO-1	19.76 ± 2.12c	9.56 ± 1c	9.95 ± 0.69c	6.97 ± 1.02a

**Table 3:** Agronomic characters comparison of *H. marmoreus* fruiting bodies (2)

Name of Samples	Fresh weight of single sporophore (g)	Weight of worthless mushroom stuff (g)	Biological transformation efficiency (%)	The effective number of mushrooms	Weight of Effective mushrooms (g)
21d-CK	30.18 ± 1.06a	79 ± 4.18c	23.21 ± 1.23a	16a	25.07 ± 1.26a
21d-HZSO-1	46.20 ± 5.03c	65.71 ± 3.89b	35.53 ± 3.87c	20b	42.38 ± 4.77b
23d-CK	41.51 ± 5.33b	61.43 ± 9.45b	31.93 ± 4.10b	21b	37.98 ± 7.66b
23d-HZSO-1	55.39 ± 2.53d	55.45 ± 3.5a	42.61 ± 1.95d	23b	49.43 ± 2.24c

### Colonization by GFP-labeled *Serratia odorifera* HZSO-1 in hyphae of *H. marmoreus*

**Fig. 6:**
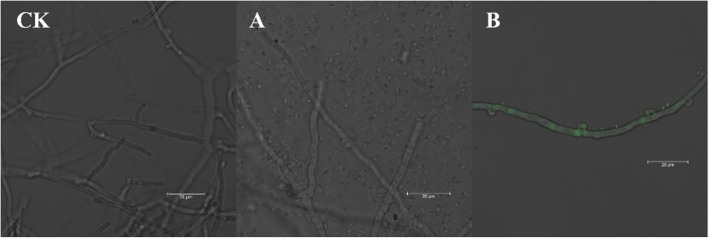
Colonization and identification of GFP-labeled *S. odorifera* HZSO-1 in hyphae of *H. marmoreus* (magnifying 20 diameters). CK: hyphae of *H. marmoreus* independently cultured in the liquid medium; **a**: hyphae of *H. marmoreus* cocultured with wild-type *S. odorifera* HZSO-1 in the liquid medium; **b**: hyphae of *H. marmoreus* cocultured with GFP-labeled *S. odorifera* HZSO-1 in the liquid medium

Under a fluorescence microscope (Fig. 6), intense green fluorescence was observed inside the hyphae of *H. marmoreus* cocultured with GFP-labeled *Serratia odorifera* HZSO-1. In contrast, the hyphae cocultured with wild-type *Serratia odorifera* HZSO-1 or independently cultured show no green fluorescence under the same conditions. The above results indicated that *Serratia odorifera* colonized the hyphae. The existence of the symbiotic *Serratia odorifera* HZSO-1 in the hyphae of *H. marmoreus* was also verified by PCR amplification, targeting variable region 4 (V4) of the 16S rRNA gene of *Serratia odorifera* HZSO-1 using the total DNA isolated from the hyphae of *H. marmoreus* as a template. Agarose gel electrophoresis analysis confirmed the successful amplification of a ~ 280 bp fragment, which was consistent with the expected size and sequence of the *V4* region of *Serratia odorifera*. This result *confirmed* that *Serratia odorifera* HZSO-1 was present inside the hyphae of *H. marmoreus* and acting as a symbiotic bacterium, promoting the growth and development of *H. marmoreus*.

## Discussion

In the diverse growth environment, many beneficial microorganisms play a crucial role in the development of edible mushrooms [12]. With increased in-depth microecological research, the beneficial effects of microbes in the production of edible fungi have captured widespread attention. Studies on a few symbiotic mycorrhizal fungi, such as truffle and boletus [21, 22], revealed that fungi need to form a symbiotic association with the roots of specific mushrooms during growth and development [8]. Due to the allelopathic effects of secreted metabolites during the growth of mycelia and mycorrhizae, a specialized microbial community is formed in rhizosphere soil of mycorrhizae which plays an essential role in the nutrient exchange between symbionts [13]. The existence of these microorganisms in the rhizosphere makes the domestication and artificial cultivation of wild mycorrhizal fungi rather tricky. In the process of *cultivation of* basidiomycetes with fermented *materials*, the thermophilic bacteria Pseudomonas and Bacillus in matrix substrates can assist the hyphae in decomposing macromolecular substances to facilitate the efficient *absorption of nutrients*. Some microorganisms in cultivation substrates can inhibit the pathogens present *during mushroom cultivation* [13]*.* Liu et al. found that *Bacillus subtilis* B154, isolated from *Agaricus bisporus* fermentation *materials,* exhibited potent inhibitory effects against Neurospora by effectively inhibiting the growth and spore germination and further improved *the fruiting body production of A. bisporus* [21]. Soil casing is an important step to stimulate fruiting in the cultivation process of *A. bisporus* and *Volvaria volvacea* [19]. The casing soil can maintain the temperature, humidity, and CO_2_ *necessary* for primordium differentiation [2], and more importantly, some beneficial microorganisms (Bacillus, Arthrobacter, *Pseudomonas putida*) present in this soil have a significant effect on the growth and morphogenesis of the edible fungi [23, 24]. Therefore, these beneficial microorganisms are naturally present in the production of edible mushrooms.

It is the first time that a large number of symbiotic bacteria associated with hyphae of *H. marmoreus* were detected using high-throughput sequencing technology. The sequencing results indicated that the community structure of symbiotic bacteria in the cultivation bags varied over the growth and development of *H. marmoreus*. Firmicutes and Proteobacteria were the dominant bacteria, followed by Bacteroides, Actinobacteria, Acidus, cyanobacteria, Campylobacter, Bacillus, Verrucomicrobia, and Fusobacterium. The abundance of Firmicutes decreased gradually from the half-bag period to the mature period, *which may be due to the following reasons*: (1) suppression of bacterial growth by metabolites secreted by *H. marmoreus*; and (2) microenvironmental changes in cultivation bags. In the cultivation bags of *H. marmoreus*, the relative abundance of three genera (Rummeliibacillus, Paenibacillus, and Lysinibacillus) was high in different stages of growth and development, especially the genus Bacillus. When the growth of *H. marmoreus* entered the full bag period, *Bacillus* spp. were the dominant species, and Bacillus bacteria promoted the growth and development of *H. marmoreus* by secreting antagonistic substances, *lysozymes, auxin, and gibberellin* [25–27]*. At the same time, Bacillus bacteria can secrete specific metabolites that can enhance stress tolerance of H. marmoreus* [25–27] *and could be used in the industrial production of mushrooms as a growth stimulant.* The abundance of the genus Serratia of the Enterobacteriaceae family increased. Subsequently, Serratia became the dominant genus in the mature period of *H. marmoreus* growth, with an increase in abundance from 1 to 23.31%, suggesting that Serratia played a critical role in the decomposition of *substrates,* and facilitated the formation of the primordium after the completion of maturation. It was once reported that the bacterial genus Serratia is a kind of biocontrol genus [28] and is therefore widely used in mushroom disease biological control because it plays a role in growth promotion [29], phosphorus solubilization [30], and pest and disease control [31] in mushrooms. These results also indicated that Proteobacteria and *H. marmoreus* could effectively promote each other’s growth and development to some extent. So as for protein, phosphorus, and potassium content in mycelia and fruiting bodies, there were no significant changes after symbiotic bacteria inoculation because *H. marmoreus* was grown in cultivation substrates containing lignin and cellulose and rich in carbohydrates.

A symbiotic bacterium with the potential for promoting the growth and development of *H. marmoreus* was isolated from the hyphae in the mature period of *H. marmoreus* growth; this bacterium was identified as *Serratia odorifera* HZSO-1 based on its 16S rDNA sequence and physiological characteristics. Its function was verified with growth promotion experiments by adding sterile fermented broth of *Serratia odorifera* HZSO-1 to agar plates and cultivation bottles. The results showed that the sterile fermentation broth of *Serratia odorifera* HZSO-1 could significantly promote the growth of hyphae and shorten the fruiting cycle by 3–4 days, which suggested that *Serratia odorifera* HZSO-1 could secrete some beneficial metabolites that promote the growth of *H. marmoreus*. However, the molecular mechanism underlying the improved growth caused by the symbiotic *Serratia odorifera* HZSO-1 remains to be clarified, and the source of the symbiotic bacterium remains unclear. *To further elucidate* the colonization of the symbiotic species *Serratia odorifera* HZSO-1, PCR amplification, and fluorescence labeling were used. The results confirmed the bacterium to be a symbiotic species residing in the hyphae of *H. marmoreus*, providing a theoretical basis for clarifying the colonization of mushroom *symbiotic bacteria and* for developing mushroom growth-promoting agents made from *S. odorifera* that can be used in the production of edible fungi [32, 33].

Interestingly, some members of the genus Serratia exhibit quorum-sensing [31]. *Serratia odorifera* HZSO-1 can also secrete N-acyl homoserine lactones to the external environment as signaling molecules under the regulation of the quorum-sensing system when it perceives a critical cell density [20]. The signaling molecule is a kind of hormone that plays a significant role in the interaction between bacteria and mushrooms [34, 35]. Therefore, it is worth studying whether Serratia bacteria can accelerate the decomposition of matrix substrates and the formation of primordia through quorum sensing after *hyphal maturation.* Taking advantage of beneficial microorganisms to promote the industrial production of edible fungi would be of great significance. In particular this study provides a promising research direction for the development and utilization of *S. odorifera* for these purposes.

## Conclusions

In this study, the concept of the effect of symbiotic microbiota on edible fungi was first raised. A correlation was established between the community structure of symbiotic bacteria and the growth and development of *H. marmoreus*. Thirty-four bacterial phyla were detected in samples taken from cultivation bags of *H. marmoreus.* Firmicutes was *the most abundant* bacterial phylum (78.85%). Proteobacteria was the second most common microorganism (15.08%). The *Serratia odorifera* HZSO-1 strain, which is capable of promoting the growth and development of edible fungi, was successfully isolated from cultivation substrates and identified as a symbiotic bacterium that resides in the hyphae of *H. marmoreus*. This strain can shorten the fruiting cycle by 3–4 days, and increase the fruiting body yield by 12%. This study is a pioneering demonstration of the community structure of *symbiotic* microbiota and bacteria-mushroom interaction in the growth and development of edible fungi. The use of symbiotic microbial resources is a novel strategy to improve the industrial production of edible fungi.

## Methods

### Strains, sample collection, and processing

**Fig. 7:**
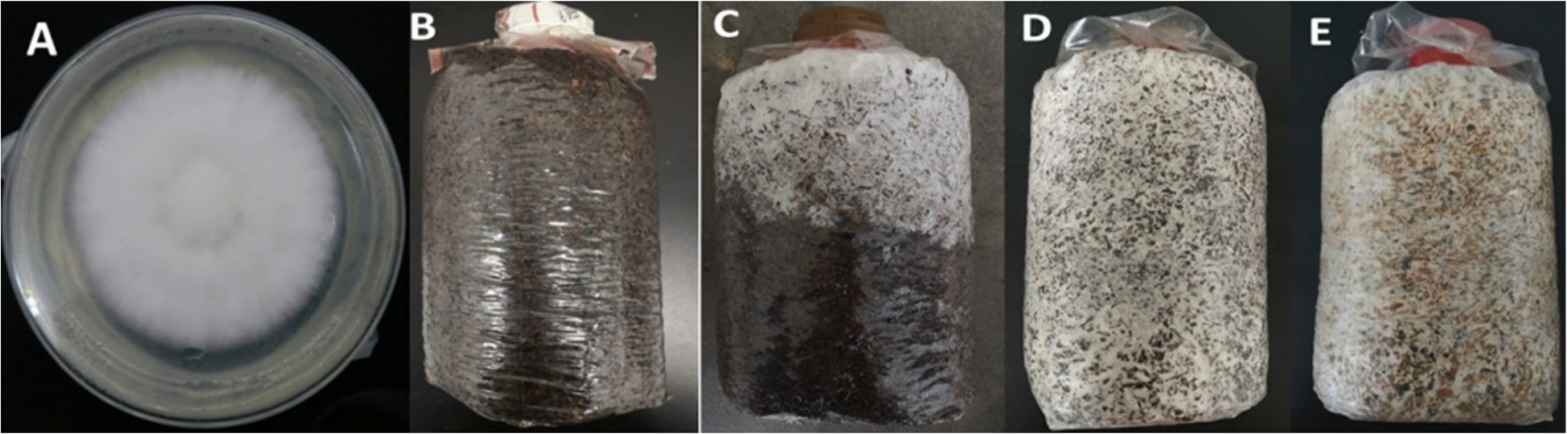
Samples of the hyphae from the plate and cultivation bags at the different growth and developmental stages of *H. marmoreus*. **a**: hyphae grown on agar plates; **b**: blank bag; **c**: half bag period; **d**: full bag period; **e**: mature period

The *H. marmoreus* 19C strain was bred by the isolation and fusion of protoplasts. The *E. coli* (pUC19-bud-kan-gfp) was constructed by genetic engineering. These two strains are available in our laboratory. All cultivation bags were obtained from Fukang Biotech Co., Ltd. (Gutian County, China). The PDA enrichment medium was prepared according to the following formula: 200 g potato infusion, 20 g dextrose, 3 g yeast powder, 3 g peptone, 1.5 g KH_2_PO_4_, 3.0 g MgSO_4_•7H_2_O, 20 g Agar, 1 L water, pH normal. Cultivation substrates were prepared as follows: 35% cottonseed hull, 35% sawdust, 25% wheat bran, 5% corn starch, and 1% CaO were mixed in water, and the water content reached 62%. Subsequently, these cultivation substrates were divided into polyethylene bags (15*25 cm) and were subjected to the high temperature and high pressure at 121 °C for 3 h. Hyphae were collected from *H. marmoreus* grown on PDA plates (Fig. 7a). The cultivation bags were chosen at random in triplicate at the different growth stages of *H. marmoreus* (Fig. 7b-e and Table S1). The hyphae in the upper, middle and lower parts of the cultivation bags were sampled at a depth of 2–5 cm and thoroughly mixed. Fifteen grams of each sample was placed in a 50-mL sterile centrifuge tube, *frozen* with liquid nitrogen, and stored at − 80 °C for subsequent experiments.

### 16S rDNA PCR amplification and high-throughput sequencing (HiSeq) [36, 37]

Total DNA was extracted from *H. marmoreus* hyphae with a Genomic DNA Kit (TransGen Biotech Co., Ltd., Beijing, China). When sufficient quantities (400–500 ng) *of high-quality* community DNA samples were available, the V3-V4 region of the 16S rDNA was amplified with universal primers (338 F: 5′-ACTCCTACGGGAGGCAGCA-3′; 806 R: 5′-GGACTACHVGGGTWTCTAAT-3′) in 50 uL reaction volume. The thermocycling parameters were set as follows: 98 °C for 2 min; 98 °C for 30 s, 50 °C for 30 s, and 72 °C for 1 min (30 cycles); and 72 °C for 5 min. After amplification, the products were purified with the GenElute™ PCR Clean-Up Kit according to the manufacturer’s instructions, quantified with a UV detector at an excitation wavelength of 260 nm, and subsequently homogenized to construct a sequencing library. After quality evaluation, the library was sequenced on an Illumina HiSeq 2500 platform at Baimaike Co., Ltd.

### Sequence data processing and bioinformatics analysis

The raw image data from the sequencing platform were converted to the original sequenced reads by CASAVA base calling analysis. Next, the raw double-ended sequences were assembled and filtered with Mothur v.1.22.2 [38] with the minimal quality average set to 30 to obtain clean tags. Finally, all chimeric reads were removed using the UCHIME method [39], and effective tags for further analysis were obtained. The effective tags were clustered into operational taxonomic units (OTUs) at 97% similarity using QIIME (Version 1.8.0) software [40]. The tag sequence with the highest abundance was selected as the representative sequence within each OTU. Next, according to the taxonomic database of bacteria and fungi, the OTUs were taxonomically annotated using Mothur (Version v.1.30) [41], and the community structure and species abundance distributions were investigated at different taxonomic levels. Subsequently, the alpha diversity index of the samples was assessed using Mothur software to compare community diversity indices of different samples and summarize ACE, Chao 1, Shannon and Simpson indices at a similarity level of 97%. A rarefaction curve was plotted to determine whether the amount of sequencing data was sufficient to reflect the species diversity in the sample. Principal component analysis (PCA) and principal coordinates analysis (PCoA) of the OTUs of the sample were carried out by the binary Jaccard measure. On this basis, the similarity of species diversity and microbial community structure characteristics of different samples was compared at various stages of growth and development of *H. marmoreus*. At the same time, the significance of the difference in beta diversity between samples was calculated using the two-tailed Student’s t-test. Finally, a redundancy analysis (RDA) was performed using the R software package to analyze the relationship of *H. marmoreus* and its symbiotic bacteria in cultivation bags to determine the association between the community structure of symbiotic bacteria and the growth cycle of *H. marmoreus*.

### Isolation and identification of dominant symbiotic bacteria and their effects on the growth and development of *H. marmoreus* [20, 42, 43]

According to the high-throughput sequencing results, the predominant symbiotic bacteria (Serratia genera) were isolated from cultivation bags when *H. marmoreus* entered the matured period*.* Single colony isolated by dilution plating procedure [44] was identified by PCR with universal primers targeting the 16S rDNA gene (16S-F: AGAGTTTGATCCTGGCTCAG; 16S-R: GGTTACCTTGTTACGACTT). The amplification procedure was as follows: 95 °C for 5 min; 95 °C for 30 s, 55 °C for 30 s, 72 °C for 90 s (30 cycles); and 72 °C for 10 min. The amplified product was verified by electrophoresis (1% agarose gel), and then the PCR products with appropriate molecular weights *were recovered from gels* and sequenced by Bioengineering Co., Ltd. The determined sequences were *aligned with the sequences deposited in the NCBI* database. The isolated symbiotic bacteria were grown at 30 °C with shaking (180 rpm) to OD_600_ = 1.9–2.1, and then, the culture was diluted with sterile LB medium to just final OD = 0.7. The diluted culture *was* subjected to centrifugation at 8000 rpm for 15 min at 4 °C, and the supernatant was filtered *with* a 0.22-μm *filter* and stored at 4 °C for subsequent experiments. Plates *were prepared* with melted PDA medium containing *different volumes of* the supernatant at ratios of 100:3, 100:5, and 100:8 (*v*/*v*), and the PDA medium was set as the control group. Next, *these plates were inoculated with* the *actively growing* mycelial part (− 1.5 cm diameter) of *H. marmoreus* and then *cultured in an incubator* at 25 °C. From the third day after inoculation, the diameter of the region covered by hyphae was measured once every 2 days, and the measurement lasted for 11 days. On this basis, the optimal amount of supernatant (5% v/v) supplementation of the PDA medium was determined and used as a reference for subsequent fruiting experiments with *H. marmoreus* in cultivation bottles. Subsequently, some *Serratia* bacteria were isolated on selective MacConkey agar media with 1% sorbitol and 2% colistin. The supernatant of strain HZSO-1 culture was added to the cultivation bottles immediately after the inoculation of *H. marmoreus*. A cultivation bottle without supernatant was used as the blank group. All cultivation bottles were incubated in a dark environment for 75–80 days under constant temperature (26 °C) and humidity (70%) conditions. At the late stage of hyphal maturation, all cultivation bottles were ready for fruiting after removal of bottle caps and stimulation of mycelia by scratching the substrate surface. The agronomic traits (Tables 2 and 3) associated with fruiting were recorded in detail after fruiting body initiation.

### Identification of the colonization pattern of the symbiotic bacteria

A plasmid (pUC19-bud-kan-*gfp*) *expressing* green fluorescent protein (GFP) was constructed and electroporated into the *Serratia odorifera* HZSO-1. The GFP-labeled bacteria were cultured at 180 rpm and 37 °C for 12 h (bacterial count: 2 × 10^7^ CFU/mL), and then inoculated into flasks containing *H. marmoreus* fermentation broth preincubated statically for 8–9 days. This mixed flora was cocultured at a ratio of approximately 1:10^4^ (*bacteria to fungi* ratio) for 5 days. A pure culture of *H. marmoreus* and hyphae cocultured with unlabeled bacteria were used as control groups. *After 5 days of coculture*, the hyphae were collected by centrifugation at 4000 rpm and washed three times with sterile water. Subsequently, *hyphae were imaged* using a confocal fluorescence microscope (Carl Zeiss LSM780, Inc., Thornwood, NY) to localize the fluorescence excitation site in *H. marmoreus* mycelia. At the same time, PCR amplification was carried out using the total DNA *extracted from H. marmoreus mycelia grown on* plates as the template with primers specific to the symbiotic *Serratia* isolates. The PCR product was analyzed with electrophoresis in 1% agarose to confirm the existence of endosymbiotic bacteria by successful PCR amplification of the V4 region in the 16S rRNA gene with the following primer pair: Serv4-F: 5′-ACGCAGGCGGTTTGTTAA-3′, Serv4-R: 5′-GAAGCCACGCCTCAAGGG-3′.

## Supplementary information

**Additional file 1: Figure S1.** Phylogenetic tree of HZSO-1 strain based on 16S rDNA sequences. Note: The value on the branch point is the support rate. The ruler 0.1 is the evolution distance. **Figure S2.** Dosage effects of 0.22-μm filter-sterilized fermentation broth of *S. odorifera* HZSO-1 on the growth rate of *H. marmoreus* hyphae. Note: Different lowercase letters indicate a significant difference between treatments at the *P*<0.05 level. **Table S1.** A description of the content of each sample as shown in Fig. 7. **Table S2.** OTUs from the different samples and replicates

**Additional file 2:.**

## Data Availability

The datasets generated and/or analysed during the current study are available in the NCBI website repository (Accession number: MN959466.1).

## Abbreviations

*H. marmoreus*: *Hypsizygus marmoreus*
*S. odorifera*: *Serratia odorifera*
*E. coli*: *Escherichia coli*
GFP: Green fluorescent protein
OTU: Operational taxonomic unit
PDA: Potato dextrose agar
ACE: Abundance-based coverage estimator
PCA: Principal component analysis
PCoA: Principal coordinates analysis
PCR: Polymerase chain reaction
DNA: Deoxyribonucleic acid
UV: Ultraviolet-visible
RDA: Redundancy analysis
CFU: Colony-forming unit
rRNA: Ribosomal RNA
OD: Optical density
NCBI: National Center for Biotechnology Information
