# Supplementary material for: Study on the community structure and function of symbiotic bacteria from different growth and developmental stages of Hypsizygus marmoreus
Source: BMC Microbiol. 2020 Oct 14;20:311. doi: 10.1186/s12866-020-01998-y (PMC7557082; doi:10.1186/s12866-020-01998-y)
Supplement: Supplementary file 1 — Additional file 1: Figure S1. Phylogenetic tree of HZSO-1 strain based on 16S rDNA sequences. Note: The value on the branch point is the support rate. The ruler 0.1 is the evolution distance. Figure S2. Dosage effects of 0.22-μm filter-sterilized fermentation broth of S. odorifera HZSO-1 on the growth rate of H. marmoreus hyphae. Note: Different lowercase letters indicate a significant difference between treatments at the P<0.05 level. Table S1. A description of the content of each sample as shown in Fig. 7. Table S2. OTUs from the different samples and replicates [file 12866_2020_1998_MOESM1_ESM.docx]

**Supplementary Materials**

**Study on the community structure and function of symbiotic bacteria from different growth and developmental stages of** ***Hypsizygus marmoreus***

**Shujing Sun^#*^, Fan Li^#^, Xin Xu, Yunchao Liu, Xuqiang Kong, Jianqiu Chen, Ting Liu, and Liding Chen**

*College of Life Sciences, Fujian Agriculture and Forestry University, Fuzhou 350002, People’s Republic of China*

*^#^These authors contributed equally to this work and joint first authors.*

**^*^** To whom correspondence should be addressed: College of Life Sciences, Fujian Agriculture and Forestry University, Fuzhou 350002, P. R. China. Tel.: +86-591-83789492; Fax: +86-591-83789352. E-mail address: shjsun2004@126.com

**Short title:** Mushroom symbiotic bacteria and their function


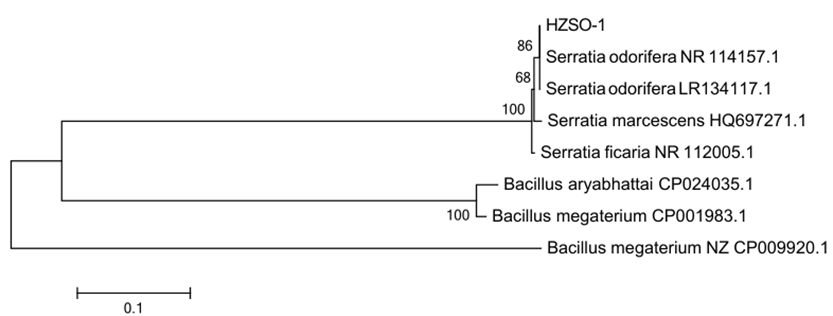


**Fig. S1** Phylogenetic tree of HZSO-1 strain based on 16S rDNA sequences

Note: The value on the branch point is the support rate. The ruler 0.1 is the evolution distance


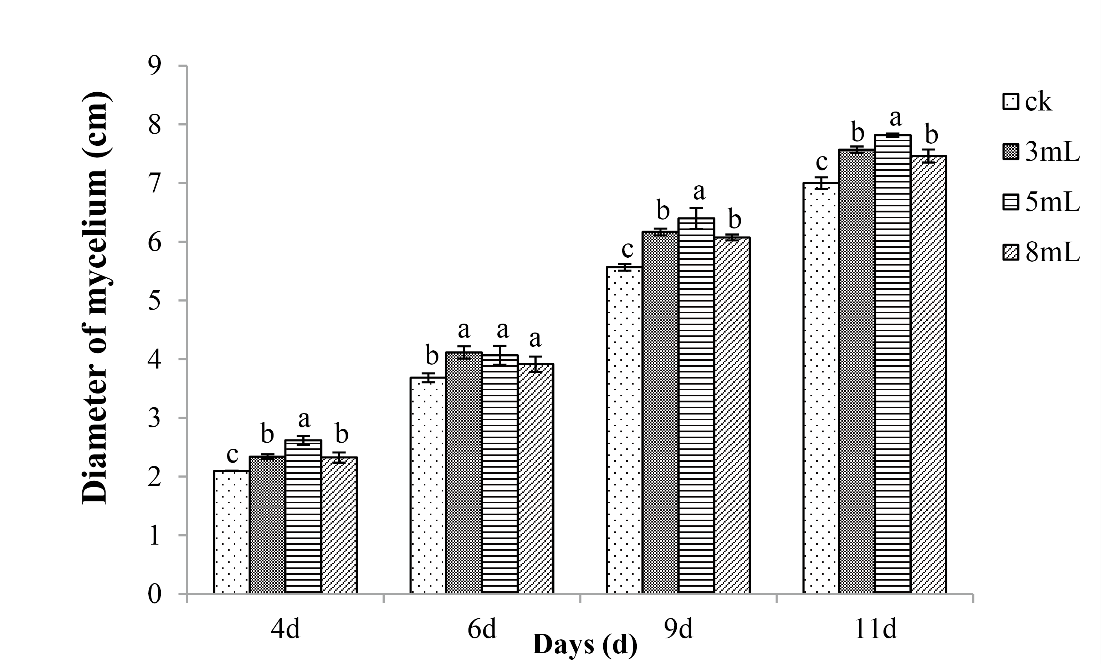


**Fig. S2** Dosage effects of 0.22-μm filter-sterilized fermentation broth of *S. odorifera* HZSO-1 on the growth rate of *H. marmoreus* hyphae. Note: Different lowercase letters indicate a significant difference between treatments at the P<0.05 level.

**Table S1 A description of the content of each sample as shown in Figure 7**

| A | B | C | D | E |
| --- | --- | --- | --- | --- |
| Hyphae | --- | Mycelia | Mycelia | Mycelia |
| PDA enrichment medium | Cultivation substrate | Cultivation substrate | Cultivation substrate | Cultivation substrate |

**Table S2 OTUs from the different samples and replicates**

| Samples  Replicates | HMCK | HMBH | HMBF | HMBm | HMPM |
| --- | --- | --- | --- | --- | --- |
| 1 | 64 | 39 | 38 | 45 | 458 |
| 2 | 181 | 34 | 61 | 209 | 527 |
| 3 | 32 | 21 | 52 | 86 | 501 |
