# Supplementary material for: Study on the community structure and function of symbiotic bacteria from different growth and developmental stages of Hypsizygus marmoreus
Source: BMC Microbiol. 2020 Oct 14;20:311. doi: 10.1186/s12866-020-01998-y (PMC7557082; doi:10.1186/s12866-020-01998-y)
Supplement: Supplementary file 2 — Additional file 2:. [file 12866_2020_1998_MOESM2_ESM.docx]

**Supplementary Materials**

**Study on the community structure and function of symbiotic bacteria from different growth and developmental stages of** ***Hypsizygus marmoreus***

**Shujing Sun^#*^, Fan Li^#^, Xin Xu, Yunchao Liu, Xuqiang Kong, Jianqiu Chen, Ting Liu, and Liding Chen**

*College of Life Sciences, Fujian Agriculture and Forestry University, Fuzhou 350002, People’s Republic of China*

*^#^These authors contributed equally to this work and joint first authors.*

**^*^** To whom correspondence should be addressed: College of Life Sciences, Fujian Agriculture and Forestry University, Fuzhou 350002, P. R. China. Tel.: +86-591-83789492; Fax: +86-591-83789352. E-mail address: shjsun2004@126.com

**Short title:** Mushroom symbiotic bacteria and their function

Shared OTUs sequences of the symbiotic bacteria isolated from *Hypsizygus marmoreus*

>OTU1GTAGGGAATCTTCCACAATGGACGAAAGTCTGATGGAGCAACGCCGCGTGAGTGATGAAGGTTTTCGGATCGTAAAACTCTGTTGTAAGGGAAGAACAAGTACGTTAGGAAATGAACGTACCTTGACGGTACCTTATTAGAAAGCCACGGCTAACTACGTGCCAGCAGCCGCGGTAATACGTAGGTGGCAAGCGTTGTCCGGATTTATTGGGCGTAAAGCGCGCGCAGGCGGTTTCTTAAGTCTGATGTGAAAGCCCACGGCTTAACCGTGGAGGGTCATTGGAAACTGGGAGACTTGAGTGCAGAAGAGGAAAGTGGAATTCCAAGTGTAGCGGTGAAATGCGTAGAGATTTGGAGGAACACCAGTGGCGAAGGCGACTTTCTGGTCTGCAACTGACGCTGAGGCGCGAAAGCATGGGGAGCAAACAGG

>OTU2GTAGGGAATCTTCCACAATGGGCGAAAGCCTGATGGAGCAACGCCGCGTGAGTGAAGAAGGTTTTCGGATCGTAAAACTCTGTTGTAAGGGAAGAACAAGTACAGTAGTAACTGGCTGTACCTTGACGGTACCTTATTAGAAAGCCACGGCTAACTACGTGCCAGCAGCCGCGGTAATACGTAGGTGGCAAGCGTTGTCCGGAATTATTGGGCGTAAAGCGCGCGCAGGCGGTCCTTTAAGTCTGATGTGAAAGCCCTCGGCTCAACCGAGGAGGGTCATTGGAAACTGGAGGACTTGAGTGCAGAAGAGGAAAGTGGAATTCCAAGTGTAGCGGTGAAATGCGTAGAGATTTGGAGGAACACCAGTGGCGAAGGCGACTTTCTGGTCTGTAACTGACGCTGAGGCGCGAAAGCGTGGGGAGCAAACAGG

>OTU3GTAGGGAATCTTCCGCAATGGGCGAAAGCCTGACGGAGCAATGCCGCGTGAGTGATGAAGGTTTTCGGATCGTAAAGCTCTGTTGCCAGGGAAGAACGTCCTTGAGAGTAACTGCTCAAGGAGTGACGGTACCTGAGAAGAAAGCCCCGGCTAACTACGTGCCAGCAGCCGCGGTAATACGTAGGGGGCAAGCGTTGTCCGGAATTATTGGGCGTAAAGCGCGCGCAGGCGGTCATTTAAGTCTGGTGTTTAATCCCGGGGCTCAACCCCGGATCGCACTGGAAACTGGATGACTTGAGTGCAGAAGAGGAGAGTGGAATTCCACGTGTAGCGGTGAAATGCGTAGAGATGTGGAGGAACACCAGTGGCGAAGGCGACTCTCTGGGCTGTAACTGACGCTGAGGCGCGAAAGCGTGGGGAGCAAACAGG

>OTU4GTGGGGAATATTGCACAATGGGCGCAAGCCTGATGCAGCCATGCCGCGTGTGTGAAGAAGGCCTTCGGGTTGTAAAGCACTTTCAGCGAGGAGGAAGGTGGTGAACTTAATACGTTCATCAATTGACGTTACTCGCAGAAGAAGCACCGGCTAACTCCGTGCCAGCAGCCGCGGTAATACGGAGGGTGCAAGCGTTAATCGGAATTACTGGGCGTAAAGCGCACGCAGGCGGTTTGTTAAGTCAGATGTGAAATCCCCGAGCTTAACTTGGGAACTGCATTTGAAACTGGCAAGCTAGAGTCTCGTAGAGGGGGGTAGAATTCCAGGTGTAGCGGTGAAATGCGTAGAGATCTGGAGGAATACCGGTGGCGAAGGCGGCCCCCTGGACGAAGACTGACGCTCAGGTGCGAAAGCGTGGGGAGCAAACAGG

>OTU5GTGGGGAATATTGCACAATGGGCGCAAGCCTGATGCAGCCATGCCGCGTGTATGAAGAAGGCCTTCGGGTTGTAAAGTACTTTCAGTCAGGAGGAAGGGTGTGAGCTTAATACGTTCATACATTGACGTTACTGACAGAAGAAGCACCGGCTAACTCCGTGCCAGCAGCCGCGGTAATACGGAGGGTGCAAGCGTTAATCGGAATTACTGGGCGTAAAGCGCACGCAGGCGGTTGGTTAAGTCAGATGTGAAATCCCCGGGCTCAACCCGGGAACTGCATTTGAAACTGGCCAGCTGGAGTCTCGTAGAGGGAGGTAGAATTCCAGGTGTAGCGGTGAAATGCGTAGAGATCTGGAGGAATACCGGTGGCGAAGGCGGCCTCCTGGACGAAGACTGACGCTCAGGTGCGAAAGCGTGGGGAGCAAACAGG

>OTU6GTAGGGAATCTTCCGCAATGGACGAAAGTCTGACGGAGCAACGCCGCGTGAGTGATGAAGGTTTTCGGATCGTAAAACTCTGTTGTTAGGGAAGAACAAGTACAAGAGTAACTGCTTGTACCTTGACGGTACCTAACCAGAAAGCCACGGCTAACTACGTGCCAGCAGCCGCGGTAATACGTAGGTGGCAAGCGTTGTCCGGAATTATTGGGCGTAAAGCGCGCGCAGGCGGTCCTTTAAGTCTGATGTGAAAGCCCACGGCTCAACCGTGGAGGGTCATTGGAAACTGGGGGACTTGAGTGCAGAAGAGAAGAGTGGAATTCCACGTGTAGCGGTGAAATGCGTAGAGATGTGGAGGAACACCAGTGGCGAAGGCGACTCTTTGGTCTGTAACTGACGCTGAGGCGCGAAAGCGTGGGGAGCAAACAGG

>OTU7GTGGGGAATATTGCACAATGGGCGCAAGCCTGATGCAGCCATGCCGCGTGTATGAAGAAGGCCTTCGGGTTGTAAAGTACTTTCAGCGGGGAGGAAGGGAGTAAAGTTAATACCTTTGCTCATTGACGTTACCCGCAGAAGAAGCACCGGCTAACTCCGTGCCAGCAGCCGCGGTAATACGGAGGGTGCAAGCGTTAATCGGAATTACTGGGCGTAAAGCGCACGCAGGCGGTTTGTTAAGTCAGATGTGAAATCCCCGGGCTCAACCTGGGAACTGCATCTGATACTGGCAAGCTTGAGTCTCGTAGAGGGGGGTAGAATTCCAGGTGTAGCGGTGAAATGCGTAGAGATCTGGAGGAATACCGGTGGCGAAGGCGGCCCCCTGGACGAAGACTGACGCTCAGGTGCGAAAGCGTGGGGAGCAAACAGG

>OTU8GTAGGGAATCTTCCACAATGGACGAAAGTCTGATGGAGCAACGCCGCGTGAGCGAAGAAGGTTTTCGGATCGTAAAGCTCTGTTGCCGGAGAAGAACGGATGCGGGAGGAAATGTCCGCGTCGTGACGGTATCCGGCCAGAAAGCCACGGCTAACTACGTGCCAGCAGCCGCGGTAATACGTAGGTGGCAAGCGTTGTCCGGAATTATTGGGCGTAAAGCGCGCGCAGGCGGCTTCTTAAGTCTGATGTGAAATTCTGCAGCTCAACTGCAGGCGGGCATTGGAAACTGGGGAGCTTGAGTACAGAAGAGGAGAGTAGAATTCCACGTGTAGCGGTGAAATGCGTAGAGATGTGGAGGAATACCAGTGGCGAAGGCGGCTCTCTGGTCTGTTACTGACGCTGAGGTGCGAAAGCGTGGGGAGCGAACAGG

>OTU9GTGGGGAATATTGGACAATGGGCGCAAGCCTGATCCAGCCATGCCGCGTGAGTGATGAAGGCCCTAGGGTTGTAAAGCTCTTTCACCGGTGAAGATAATGACGGTAACCGGAGAAGAAGCCCCGGCTAACTTCGTGCCAGCAGCCGCGGTAATACGAAGGGGGCTAGCGTTGTTCGGATTTACTGGGCGTAAAGCGCACGTAGGCGGACTTTTAAGTCAGGGGTGAAATCCCGGGGCTCAACCCCGGAACTGCCTTTGATACTGGAAGTCTTGAGTATGGTAGAGGTGAGTGGAATTCCGAGTGTAGAGGTGAAATTCGTAGATATTCGGAGGAACACCAGTGGCGAAGGCGGCTCACTGGACCATTACTGACGCTGAGGTGCGAAAGCGTGGGGAGCAAACAGG

>OTU10GTAGGGAATCTTCCGCAATGGGCGAAAGCCTGACGGAGCAATGCCGCGTGAGTGATGAAGGTTTTCGGATCGTAAAGCTCTGTTGCCAGGGAAGAACGCTTGGGAGAGTAACTGCTCTCAAGGTGACGGTACCTGAGAAGAAAGCCCCGGCTAACTACGTGCCAGCAGCCGCGGTAATACGTAGGGGGCAAGCGTTGTCCGGAATTATTGGGCGTAAAGCGCGCGCAGGCGGTCATTTAAGTCTGGTGTTTAATCCCGGGGCTCAACCCCGGATCGCACTGGAAACTGGGTGACTTGAGTGCAGAAGAGGAGAGTGGAATTCCACGTGTAGCGGTGAAATGCATAGATATGTGGAGGAACACCAGTGGCGAAGGCGACTCTCTGGGCTGTAACTGACGCTGAGGCGCGAAAGCGTGGGGAGCAAACAGG

>OTU11GTGGGGAATATTGCACAATGGGCGGAAGCCTGATGCAGCAACGCCGCGTGCGGGATGACGGCCTTCGGGTTGTAAACCGCTTTCGCCTGTGACGAAGCGTGAGTGACGGTAATGGGTAAAGAAGCACCGGCTAACTACGTGCCAGCAGCCGCGGTGATACGTAGGGTGCGAGCGTTGTCCGGATTTATTGGGCGTAAAGGGCTCGTAGGTGGTTGATCGCGTCGGAAGTGTAATCTTGGGGCTTAACCCTGAGCGTGCTTTCGATACGGGTTGACTTGAGGAAGGTAGGGGAGAATGGAATTCCTGGTGGAGCGGTGGAATGCGCAGATATCAGGAGGAACACCAGTGGCGAAGGCGGTTCTCTGGGCCTTTCCTGACGCTGAGGAGCGAAAGCGTGGGGAGCGAACAGG

>OTU12GTGGGGAATTTTGGACAATGGGGGAAACCCTGATCCAGCCATCCCGCGTGTGCGATGAAGGCCTTCGGGTTGTAAAGCACTTTTGGCAGGAAAGAAACGTCATGGGTTAATACCCCGTGAAACTGACGGTACCTGCAGAATAAGCACCGGCTAACTACGTGCCAGCAGCCGCGGTAATACGTAGGGTGCAAGCGTTAATCGGAATTACTGGGCGTAAAGCGTGCGCAGGCGGTTCGGAAAGAAAGATGTGAAATCCCAGAGCTTAACTTTGGAACTGCATTTTTAACTACCGAGCTAGAGTGTGTCAGAGGGAGGTGGAATTCCGCGTGTAGCAGTGAAATGCGTAGATATGCGGAGGAACACCGATGGCGAAGGCAGCCTCCTGGGATAACACTGACGCTCATGCACGAAAGCGTGGGGAGCAAACAGG

>OTU13GTGGGGAATATTGGACAATGGGGGGAACCCTGATCCAGCCATGCCGCGTGTGTGAAGAAGGCCTTATGGTTGTAAAGCACTTTAAGCGAGGAGGAGGCTCTCTTGGTTAATACCCAAGATGAGTGGACGTTACTCGCAGAATAAGCACCGGCTAACTCTGTGCCAGCAGCCGCGGTAATACAGAGGGTGCGAGCGTTAATCGGATTTACTGGGCGTAAAGCGTGCGTAGGCGGCTTTTTAAGTCGGATGTGAAATCCCCGAGCTTAACTTGGGAATTGCATTCGATACTGGGAAGCTAGAGTATGGGAGAGGATGGTAGAATTCCAGGTGTAGCGGTGAAATGCGTAGAGATCTGGAGGAATACCGATGGCGAAGGCAGCCATCTGGCCTAATACTGACGCTGAGGTACGAAAGCATGGGGAGCAAACAGG

>OTU14GTGGGGAATTTTGGACAATGGGGGAAACCCTGATCCAGCCATGCCGCGTGCAGGATGAAGGCCTTCGGGTTGTAAACTGCTTTTGTACGGAACGAAAAGATTCATTCTAATAAAGTGGATCCATGACGGTACCGTAAGAATAAGCACCGGCTAACTACGTGCCAGCAGCCGCGGTAATACGTAGGGTGCAAGCGTTAATCGGAATTACTGGGCGTAAAGCGTGCGCAGGCGGTTATGTAAGACAGATGTGAAATCCCCGGGCTCAACCTGGGAACTGCATTTGTGACTGCATAGCTGGAGTGCGGCAGAGGGGGATGGAATTCCGCGTGTAGCAGTGAAATGCGTAGATATGCGGAGGAACACCGATGGCGAAGGCAATCCCCTGGGCCTGCACTGACGCTCATGCACGAAAGCGTGGGGAGCAAACAGG

>OTU15GTAGGGAATCTTCGGCAATGGACGAAAGTCTGACCGAGCAACGCCGCGTGAGTGAAGAAGGTTTTCGGATCGTAAAACTCTGTTGTTAGAGAAGAACGACGAGGAGAGTGGAAAGCTCCTCGTGTGACGGTATCTAACCAGAAAGGGACGGCTAACTACGTGCCAGCAGCCGCGGTAATACGTAGGTCCCGAGCGTTGTCCGGATTTATTGGGCGTAAAGCGAGCGCAGGTGGTTTATTAAGTCTGGTGTAAAAGGCAGTGGCTCAACCATTGTATGCATTGGAAACTGGTAGACTTGAGTGCAGGAGAGGAGAGTGGAATTCCATGTGTAGCGGTGAAATGCGTAGATATATGGAGGAACACCGGTGGCGAAAGCGGCTCTCTGGCCTGTAACTGACACTGAGGCTCGAAAGCGTGGGGAGCAAACAGG

>OTU16GTGGGGGATCTTGCACAATGGAGGGAACTCTGATGCAGCGACGCCGCGTGAGGGAAGACGGTTTTCGGATTGTAAACCTCTGTCCCCGGTGACGATAATGACGGTAGCCGAGGAGGAAGCTCCGGCTAACTACGTGCCAGCAGCCGCGGTAATACGTAGGGAGCGAGCGTTGTCCGGATTTACTGGGTGTAAAGGGTGCGTAGGCGGCTGAACAAGTCAGGTGTGAAAACCATCGGCTCAACTGATGGATTGCACTTGAAACTGTGCAGCTTGAGTGAAGCAGAGGCAGGCGGAATTCCCGGTGTAGCGGTGAAATGCGTAGAGATCGGGAGGAACACCAGTGGCGAAGGCGGCCTGCTGGGCTTTAACTGACGCTGAGGCACGAAAGCATGGGTAGCAAACAGG

>OTU17GTAGGGAATCTTCCACAATGGACGAAAGTCTGATGGAGCAACGCCGCGTGAGTGAAGAAGGGTTTCGGCTCGTAAAACTCTGTTGTTAAAGAAGAACATATCTGAGAGTAACTGTTCAGGTATTGACGGTATTTAACCAGAAAGCCACGGCTAACTACGTGCCAGCAGCCGCGGTAATACGTAGGTGGCAAGCGTTGTCCGGATTTATTGGGCGTAAAGCGAGCGCAGGCGGTTTTTTAAGTCTGATGTGAAAGCCTTCGGCTCAACCGAAGAAGTGCATCGGAAACTGGGAAACTTGAGTGCAGAAGAGGACAGTGGAACTCCATGTGTAGCGGTGAAATGCGTAGATATATGGAAGAACACCAGTGGCGAAGGCGGCTGTCTGGTCTGTAACTGACGCTGAGGCTCGAAAGTATGGGTAGCAAACAGG

>OTU18GTGGGGAATATTGCACAATGGGCGCAAGCCTGATGCAGCCATGCCGCGTGTATGAAGAAGGCCTTCGGGTTGTAAAGTACTTTCAGCGGGGAGGAAGGCGATAAGGTTAATAACCTTGTCGATTGACGTTACCCGCAGAAGAAGCACCGGCTAACTCCGTGCCAGCAGCCGCGGTAATACGGAGGGTGCAAGCGTTAATCGGAATTACTGGGCGTAAAGCGCACGCAGGCGGTCTGTCAAGTCGGATGTGAAATCCCCGGGCTCAACCTGGGAACTGCATTCGAAACTGGCAGGCTAGAGTCTTGTAGAGGGGGGTAGAATTCCAGGTGTAGCGGTGAAATGCGTAGAGATCTGGAGGAATACCGGTGGCGAAGGCGGCCCCCTGGACAAAGACTGACGCTCAGGTGCGAAAGCGTGGGGAGCAAACAGG

>OTU19GTGGGGAATATTGGACAATGGGGGCAACCCTGATCCAGCAATGCCGCGTGTGTGAAGAAGGTTTTCGGATTGTAAAGCACTTTCGGCGGGGACGATGATGACGGTACCCGCAGAAGAAGCCCCGGCTAACTTCGTGCCAGCAGCCGCGGTAATACGAAGGGGGCTAGCGTTGCTCGGAATGACTGGGCGTAAAGGGCGTGTAGGCGGTTTGTACAGTCAGATGTGAAATCCCCGGGCTTAACCTGGGAGCTGCATTTGATACGTGCAAACTAGAGTGTGAGAGAGGGTTGTGGAATTCCCAGTGTAGAGGTGAAATTCGTAGATATTGGGAAGAACACCGGTGGCGAAGGCGGCAACCTGGCTCATAACTGACGCTGAGGCGCGAAAGCGTGGGGAGCAAACAGG

>OTU20GTAAGGAATATTGGACAATGGGCGCAAGCCTGATCCAGCCATGCCGCGTGAAGGATTAAGGTCCTCTGGATTGTAAACTTCTTTTATAAGGGACGAAAAAAGGGAATTCTTTCTCACTTGACGGTACCTTATGAATAAGCACCGGCTAACTCCGTGCCAGCAGCCGCGGTAATACGGAGGGTGCAAGCGTTATCCGGATTCACTGGGTTTAAAGGGTGCGTAGGCGGGTATTTAAGTCAGTGGTGAAATCCTAGAGCTTAACTCTAGAACTGCCATTGATACTATTTATCTTGAATATTGTGGAGGTAAGCGGAATATGTCATGTAGCGGTGAAATGCTTAGATATGACATAGAACACCTATTGCGAAGGCAGCTTACTACGCATATATTGACGCTGAGGCACGAAAGCGTGGGGATCAAACAGG

>OTU21GTAGGGAATCTTCCACAATGGGCGAAAGCCTGATGGAGCAACGCCGCGTGTGTGATGAAGGGTTTCGGCTCGTAAAACACTGTTGTAAGAGAAGAATGACATTGAGAGTAACTGTTCAATGTGTGACGGTATCTTACCAGAAAGGAACGGCTAAATACGTGCCAGCAGCCGCGGTAATACGTATGTTCCAAGCGTTATCCGGATTTATTGGGCGTAAAGCGAGCGCAGACGGTTATTTAAGTCTGAAGTGAAAGCCCTCAGCTCAACTGAGGAATTGCTTTGGAAACTGGATGACTTGAGTGCAGTAGAGGAAAGTGGAACTCCATGTGTAGCGGTGAAATGCGTAGATATATGGAAGAACACCAGTGGCGAAGGCGGCTTTCTGGACTGTAACTGACGTTGAGGCTCGAAAGTGTGGGTAGCAAACAGG

>OTU22GTGGGGAATATTGCACAATGGAGGAAACTCTGATGCAGCAACGCCGCGTGAGTGATGAAGGTCTTCGGATTGTAAAGCTCTGTCTTCTGGGACGATAATGACGGTACCAGAGGAGGAAGCCACGGCTAACTACGTGCCAGCAGCCGCGGTAATACGTAGGTGGCAAGCGTTGTCCGGATTTACTGGGCGTAAAGGATGTGTAGGCGGATATTTAAGTGAGATGTGAAATCCCCGGGCTCAACTTGGGGGCTGCATTTCAAACTGGATATCTAGAGTGCAGGAGAGGAAAGCGGAATTCCTAGTGTAGCGGTGAAATGCGTAGAGATTAGGAAGAACATCAGTGGCGAAGGCGGCTTTCTGGACTGTAACTGACGCTGAGGCATGAAAGCGTGGGGAGCAAACAGG

>OTU23GTAGGGAATCTTCGGCAATGGACGAAAGTCTGACCGAGCAACGCCGCGTGAGTGAAGAAGGTTTTCGGATCGTAAAACTCTGTTGTTAGAGAAGAACAAGGATGAGAGTAACTGTTCATCCCTTGACGGTATCTAACCAGAAAGCCACGGCTAACTACGTGCCAGCAGCCGCGGTAATACGTAGGTGGCAAGCGTTGTCCGGATTTATTGGGCGTAAAGCGAGCGCAGGCGGTTTCTTAAGTCTGATGTGAAAGCCCCCGGCTCAACCGGGGAGGGTCATTGGAAACTGGGAGACTTGAGTGCAGAAGAGGAGAGTGGAATTCCATGTGTAGCGGTGAAATGCGTAGATATATGGAGGAACACCAGTGGCGAAGGCGGCTCTCTGGTCTGTAACTGACGCTGAGGCTCGAAAGCGTGGGGAGCAAACAGG

>OTU24GTGAGGAATATTGGTCAATGGTCGTGAGACTGAACCAGCCAAGTAGCGTGCGGGATGAAGGCCCTCCGGGTCGTAAACCGCTTTTAGACGGGGATAAAAGGGCATACGTGTATGCCGTATTGCATGTACCGTCAGAAAAAGGACCGGCTAATTCCGTGCCAGCAGCCGCGGTAATACGGAAGGTCCGGGCGTTATCCGGATTTATTGGGTTTAAAGGGAGCGTAGGCGGACTTTCAAGTCAGTGGTAAAATCGTGTGGCTCAACCATACCTCGCCATTGAAACTGGAAGTCTTGAGTGCACGCAGGGGTGCCGGAATTCATGGTGTAGCGGTGAAATGCTTAGATATCATGAAGAACTCCGATCGCGAAGGCAAGTGCCCGGAGTGCAACTGACGCTGAGGCTCGAAGGTGCGGGTATCGAACAGG

>OTU25GTAGGGAATCTTCCACAATGGGCGAAAGCCTGATGGAGCAACGCCGCGTGAGTGAAGAAGGTTTTCGGATCGTAAAGCTCTGTTGTTGGTGAAGAAGGATAGAGGTAGTAACTGGCCTTTATTTGACGGTAATCAACCAGAAAGTCACGGCTAACTACGTGCCAGCAGCCGCGGTAATACGTAGGTGGCAAGCGTTGTCCGGATTTATTGGGCGTAAAGCGAGCGCAGGCGGAAAGATAAGTCTGATGTGAAAGCCCTCGGCTTAACCGGGGAAGTGCATCGGAAACTGTTTTTCTTGAGTGCAGAAGAGGAGAGTGGAACTCCATGTGTAGCGGTGGAATGCGTAGATATATGGAAGAACACCAGTGGCGAAGGCGGCTCTCTGGTCTGCAACTGACGCTGAGGCTCGAAAGCATGGGTAGCGAACAGG

>OTU26GTGGGGAATCTTGCGCAATGGGCGAAAGCCTGACGCAGCCATGCCGCGTGAATGATGAAGGTCTTAGGATTGTAAAATTCTTTCACCGGGGACGATAATGACGGTACCCGGAGAAGAAGCCCCGGCTAACTTCGTGCCAGCAGCCGCGGTAATACGAAGGGGGCTAGCGTTGCTCGGAATTACTGGGCGTAAAGGGCGCGTAGGCGGACAGTTAAGTCGGGGGTGAAAGCCCGGGGCTCAACCTCGGAATTGCCTTCGATACTGGCTGTCTTGAGTACGGGAGAGGTGTGTGGAACTCCGAGTGTAGAGGTGAAATTCGTAGATATTCGGAAGAACACCAGTGGCGAAGGCGACACACTGGCCCGTTACTGACGCTGAGGCGCGAAAGCGTGGGGAGCAAACAGG

>OTU27GTGGGGAATATTGGACAATGGGGGGAACCCTGATCCAGCCATGCCGCGTGTGTGAAGAAGGCCTTTTGGTTGTAAAGCACTTTAAGCGAGGAGGAGGCTACTCTAGTTAATACCTAGGGATAGTGGACGTTACTCGCAGAATAAGCACCGGCTAACTCTGTGCCAGCAGCCGCGGTAATACAGAGGGTGCGAGCGTTAATCGGATTTACTGGGCGTAAAGCGTGCGTAGGCGGCTTTTTAAGTCGGATGTGAAATCCCTGAGCTTAACTTAGGAATTGCATTCGATACTGGGAAGCTAGAGTATGGGAGAGGATGGTAGAATTCCAGGTGTAGCGGTGAAATGCGTAGAGATCTGGAGGAATACCGATGGCGAAGGCAGCCATCTGGCCTAATACTGACGCTGAGGTACGAAAGCATGGGGAGCAAACAGG

>OTU28GTGGGGAATTTTCCGCAATGGGCGAAAGCCTGACGGAGCAATGCCGCGTGGAGGTAGAAGGCCTACGGGTCCTGAACTTCTTTTCCCAGAGAAGAAGCAATGACGGTATCTGGGGAATAAGCATCGGCTAACTCTGTGCCAGCAGCCGCGGTAATACAGAGGATGCAAGCGTTATCCGGAATGATTGGGCGTAAAGCGTCTGTAGGTGGCTTTTTAAGTCCGCCGTCAAATCCCAGGGCTCAACCCTGGACAGGCGGTGGAAACTACCAAGCTTGAGTACGGTAGGGGCAGAGGGAATTTCCGGTGGAGCGGTGAAATGCGTAGAGATCGGAAAGAACACCAACGGCGAAAGCACTCTGCTGGGCCGACACTGACACTGAGAGACGAAAGCTAGGGGAGCGAATGGG

>OTU29GTGGGGAATATTGCACAATGGGGGAAACCCTGATGCAGCAACGCCGCGTGAGTGATGACGGTCTTCGGATTGTAAAGCTCTGTCTTCAGGGACGATAATGACGGTACCTGAGGAGGAAGCCACGGCTAACTACGTGCCAGCAGCCGCGGTAATACGTAGGTGGCAAGCGTTGTCCGGATTTACTGGGCGTAAAGGGAGCGTAGGTGGATATTTAAGTGGGATGTGAAATACTCGGGCTTAACCTGGGTGCTGCATTCCAAACTGGATATCTAGAGTGCAGGAGAGGAAAGTAGAATTCCTAGTGTAGCGGTGAAATGCGTAGAGATTAGGAAGAATACCAGTGGCGAAGGCGACTTTCTGGACTGTAACTGACACTGAGGCTCGAAAGCGTGGGGAGCAAACAGG

>OTU30GTGGGGAATATTGCACAATGGGGGAAACCCTGATGCAGCGACGCCGCGTGAGTGAAGAAGTATTTCGGTATGTAAAGCTCTATCAGCAGGGAAGATGATGACGGTACCTGAGTAAGAAGCCCCGGCTAACTACGTGCCAGCAGCCGCGGTAATACGTAGGGGGCAAGCGTTATCCGGATTTACTGGGTGTAAAGGGAGCGTAGACGGCATGGCAAGTCTGAAGTGAAAGCCCGGGGCTCAACCGCGGGACTGCTTTGGAAACTGTTAAGCTGGAGTGCAGGAGAGGTAAGTGGAATTCCTAGTGTAGCGGTGAAATGCGTAGATATTAGGAGGAACACCAGTGGCGAAGGCGGCTTACTGGACTGTAACTGACGTTGAGGCTCGAAAGCGTGGGGAGCAAACAGG

>OTU31GTGGGGAATATTGCACAATGGGCGAAAGCCTGATGCAGCAACGCCGCGTGAGTGATGAAGGTCTTCGGATTGTAAAGCTCTGTCTTTTGGGACGATAATGACGGTACCAAAGGAGGAAGCCACGGCTAACTACGTGCCAGCAGCCGCGGTAATACGTAGGTGGCGAGCGTTGTCCGGATTTACTGGGCGTAAAGGGTGCGTAGGCGGATGTTTAAGTGAGATGTGAAATACCCGGGCTTAACTTGGGTGCTGCATTTCAAACTGGATATCTAGAGTGCAGGAGAGGAGAATGGAATTCCTAGTGTAGCGGTGAAATGCGTAGAGATTAGGAAGAACACCAGTGGCGAAGGCGATTCTCTGGACTGTAACTGACGCTGAGGCACGAAAGCGTGGGTAGCAAACAGG

>OTU32GTGGGGGATATTGCACAATGGAGGAAACTCTGATGCAGCAACGCCGCGTGAAGGATGAAGGTCTTCGGATTGTAAACTTTTGTACTTGGGGACGATAATGACGGTACCCAAGCAGCAAGCTCCGGCTAACTACGTGCCAGCAGCCGCGGTAATACGTAGGGAGCAAGCGTTGTCCGGATTTACTGGGTGTAAAGGGTGCGTAGGCGGCTATGCAAGTCAGTTGTGAAAACTATGGGCTCAACCCATAGCCTGCAATTGAAACTGCGTGGCTTGAGTGAAGTAGAGGTAGGTGGAATTCCCGGTGTAGCGGTGAAATGCGTAGAGATCGGGAGGAACACCAGTGGCGAAGGCGACCTACTGGGCTTTAACTGACGCTGAGGCACGAAAGCATGGGTAGCAAACAGG

>OTU33GTAGGGAATCTTCCGCAATGGACGAAAGTCTGACGGAGCAACGCCGCGTGAGTGATGAAGGCTTTCGGGTCGTAAAACTCTGTTGTTAGGGAAGAACAAGTGCTAGTTGAATAAGCTGGCACCTTGACGGTACCTAACCAGAAAGCCACGGCTAACTACGTGCCAGCAGCCGCGGTAATACGTAGGTGGCAAGCGTTATCCGGAATTATTGGGCGTAAAGCGCGCGCAGGTGGTTTCTTAAGTCTGATGTGAAAGCCCACGGCTCAACCGTGGAGGGTCATTGGAAACTGGGAGACTTGAGTGCAGAAGAGGAAAGTGGAATTCCATGTGTAGCGGTGAAATGCGTAGAGATATGGAGGAACACCAGTGGCGAAGGCGACTTTCTGGTCTGTAACTGACACTGAGGCGCGAAAGCGTGGGGAGCAAACAGG

>OTU34GTGGGGAATATTGCACAATGGGGGGAACCCTGATGCAGCCATGCCGCGTGTATGAAGAAGGCCTTAGGGTTGTAAAGTACTTTCGTGGAGGAGGAAGGGTTTACGTTTAATAGGCGTGGACATTGACGTTACTCCAAGAAGAAGCACCGGCTAACTCCGTGCCAGCAGCCGCGGTAATACGGAGGGTGCGAGCGTTAATCGGAATGACTGGGCGTAAAGGGCATGTAGGCGGATGATTAAGTTAGATGTGAAATCCCTGGGCTTAACCTAGGAATTGCATTTAAAACTGGTTATCTAGAGTATTGTAGAGGAAGGTAGAATTCCATGTGTAGCGGTGAAATGCGTAGAGATGTGGAGGAATACCGGTGGCGAAGGCGGCCTTCTGGACAGATACTGACGCTGAGATGCGAAAGCGTGGGGAGCAAACAGG

>OTU35GTGGGGAATATTGCACAATGGGGGAAACCCTGATGCAGCGACGCAGCGTGCGGGATGACGGCCTTCGGGTTGTAAACCGCTTTCAGCAGGGAAGAAGCGCAAGTGACGGTACCTGCAGAAGAAGTACCGGCTAACTACGTGCCAGCAGCCGCGGTAATACGTAGGGTACGAGCGTTGTCCGGAATTATTGGGCGTAAAGAGCTCGTAGGTGGTTGGTCACGTCTGCTGTGGAAACGCAACGCTTAACGTTGCGCGTGCAGTGGGTACGGGCTGACTAGAGTGCAGTAGGGGAGTCTGGAATTCCTGGTGTAGCGGTGAAATGCGCAGATATCAGGAGGAACACCGGTGGCGAAGGCGGGACTCTGGGCTGTAACTGACACTGAGGAGCGAAAGCATGGGGAGCGAACAGG

>OTU36GTGGGGAATATTGCACAATGGGGGAAACCCTGATGCAGCGACGCCGCGTGAGTGAAGAAGTATTTCGGTATGTAAAGCTCTATCAGCAAGGAAGATAATGACGGTACTTGACTAAGAAGCCCCGGCTAACTACGTGCCAGCAGCCGCGGTAATACGTAGGGGGCAAGCGTTATCCGGATTTACTGGGTGTAAAGGGAGCGTAGGTGGCATGGTAAGTCAGATGTGAAAGCCCAGGGCTCAACCCTGGGACTGCATTTGAAACTATCAGGCTAGAGTGCAGGAGAGGTAAGTGGAATTCCTAGTGTAGCGGTGAAATGCGTAGATATTAGGAAGAACACCAGTGGCGAAGGCGGCTTACTGGACTGTAACTGACACTGAGGCTCGAAAGCGTGGGGAGCAAACAGG

>OTU37GTGAGGAATATTGGTCAATGGGCGAGAGCCTGAACCAGCCAAGTAGCGTGAAGGACGACGGCCCTATGGGTCTTAAACTTCTTTTATAAGGGAATAAAATGCGCCACGTGTGGTGTTTTGTATGTACCTTATGAATAAGCATCGGCTAATTCCGTGCCAGCAGCCGCGGTAATACGGAAGATGCGAGCGTTATCCGGATTTATTGGGTTTAAAGGGAGCGTAGGCGGGCTGTTAAGTCAGCGGTCAAATCGTGCGGCTCAACCGTACCTTGCCGTTGAAACTGGTGGTCTTGAGTTCGGACAGGGCAGATGGAATTCGTGGTGTAGCGGTGAAATGCTTAGATATCACGAAGAACTCCGACAGCGAAGGCATTCTGCCGGGGCGAAACTGACGCTGAGGCTCGAAAGTGCGGGTATCAAACAGG

>OTU38GTGGGGAATTTTGGACAATGGGCGAAAGCCTGATCCAGCAATGCCGCGTGTGTGAAGAAGGCCTTCGGGTTGTAAAGCACTTTTGTCCGGAAAGAAATGGCTCTGGTTAATACCTGGGGTCGATGACGGTACCGGAAGAATAAGGACCGGCTAACTACGTGCCAGCAGCCGCGGTAATACGTAGGGTCCAAGCGTTAATCGGAATTACTGGGCGTAAAGCGTGCGCAGGCGGTTGTGCAAGACCGATGTGAAATCCCCGAGCTTAACTTGGGAATTGCATTGGTGACTGCACGGCTAGAGTGTGTCAGAGGGGGGTAGAATTCCACGTGTAGCAGTGAAATGCGTAGAGATGTGGAGGAATACCGATGGCGAAGGCAGCCCCCTGGGATAACACTGACGCTCATGCACGAAAGCGTGGGGAGCAAACAGG

>OTU39GTGGGGAATATTGCACAATGGGGGGAACCCTGATGCAGCGATGCCGCGTGGAGGAAGAAGGTTTTCGGATTGTAAACTCCTGTCGTAAGGGACGATAATGACGGTACCTTACAAGAAAGCTCCGGCTAACTACGTGCCAGCAGCCGCGGTAATACGTAGGGAGCGAGCGTTGTCCGGAATTACTGGGTGTAAAGGGAGCGTAGGCGGGACGGCAAGTCAGATGTGAAATATACGTGCTCAACATGTAGACTGCATTTGAAACTGTCGTTCTTGAGTGAGGTAGAGGTAAGCGGAATTCCTGGTGTAGCGGTGAAATGCGTAGAGATCAGGAGGAACATCGGTGGCGAAGGCGGCTTACTGGGCCTTTACTGACGCTGAGGCTCGAAAGCGTGGGGAGCAAACAGG

>OTU40GTGGGGAATTTTGGACAATGGGCGCAAGCCTGATCCAGCAATGCCGCGTGCAGGAAGAAGGCCTTCGGGTTGTAAACTGCTTTTGTACGGAACGAAAAGGTTCGGGTTAATACCCTGGGCTCATGACGGTACCGTAAGAATAAGCACCGGCTAACTACGTGCCAGCAGCCGCGGTAATACGTAGGGTGCAAGCGTTAATCGGAATTACTGGGCGTAAAGCGTGCGCAGGCGGTTTTGTAAGACAGGCGTGAAATCCCCGGGCTCAACCTGGGAATGGCGCTTGTGACTGCAAAGCTGGAGTGCGGCAGAGGGGGATGGAATTCCGCGTGTAGCAGTGAAATGCGTAGATATGCGGAGGAACACCAATGGCGAAGGCAATCCCCTGGGCCTGCACTGACGCTCATGCACGAAAGCGTGGGGAGCAAACAGG

>OTU41GTGGGGAATATTGCACAATGGGGGAAACCCTGATGCAGCGACGCCGCGTGAGCGAAGAAGTATTTCGGTATGTAAAGCTCTATCAGCAGGGAAGAAAAATGACGGTACCTGACTAAGAAGCACCGGCTAAATACGTGCCAGCAGCCGCGGTAATACGTATGGTGCAAGCGTTATCCGGATTTACTGGGTGTAAAGGGAGCGCAGGCGGTACGGCAAGTCTGATGTGAAAGCCCGGGGCTCAACCCCGGTACTGCATTGGAAACTGTCGGACTAGAGTGTCGGAGGGGTAAGTGGAATTCCTAGTGTAGCGGTGAAATGCGTAGATATTAGGAGGAACACCAGTGGCGAAGGCGGCTTACTGGACGATTACTGACGCTGAGGCTCGAAAGCGTGGGGAGCAAACAGG

>OTU42GTGGGGAATATTGCGCAATGGGCGAAAGCCTGACGCAGCGACGCCGCGTGAGGGATGAAGGTCTTTGGATCGTAAACCTCTGTCAGGGGGGAAGAAGTGTATAGATTCAAATAGGGTTTATATTTGACGGTACCCCCAGAGGAAGCACCGGCTAACTCCGTGCCAGCAGCCGCGGTAATACGGAGGGTGCAAGCGTTAATCGGAATTACTGGGCGTAAAGCGCGCGTAGGTGGTTATATAAGTCAGAGGTGAAATCCCACCGCTCAACGGTGGAACTGCCTTTGATACTGTGTAACTAGAGTATCGGAGAGGGTGGCGGAATTCCAGGTGTAGGGGTGAAATCCGTAGATATCTGGAGGAACACCAGTGGCGAAGGCGGCCACCTGGACGAAGACTGACACTGAGGTGCGAAAGCGTGGGGAGCAAACAGG

>OTU43GTGGGGGATATTGCACAATGGAGGAAACTCTGATGCAGCAACGCCGCGTGAGGGAAGACGGTCTTCGGATTGTAAACCTTTGTCCTTGGTGAAGATAATGACGGTAGCCGAGGAGGAAGCTCCGGCTAACTACGTGCCAGCAGCCGCGGTAATACGTAGGGAGCAAGCGTTGTCCGGATTTACTGGGTGTAAAGGGTGCGTAGGCGGCTCTGCAAGTCAGGTGTGAAAACCATCGGCTCAACTGATGGACTGCATTTGAAACTGTGGGGCTTGAGTGAAGTAGAGGCAGGTGGAATTCCCGGTGTAGCGGTGGAATGCGTAGAGATCGGGAGGAACACCAGTGGCGAAGGCGGCCTGCTGGGCTTTAACTGACGCTGAGGCACGAAAGCATGGGTAGCAAACAGG

>OTU44GTAGGGAATCTTCCGCAATGGACGAAAGTCTGACGGAGCAACGCCGCGTGAGCGAAGAAGGCCTTCGGGTCGTAAAGCTCTGTTGTAAGGGAAGAACAAGCGCCGGTTAACTGCCGGCGCCCTGACGGTACCTTACCAGAAAGCCACGGCTAACTACGTGCCAGCAGCCGCGGTAATACGTAGGTGGCAAGCGTTGTCCGGAATTATTGGGCGTAAAGCGCGCGCAGGCGGTTTCTTAAGTCTGATGTGAAAGCCCACGGCTCAACCGTGGAGGGTCATTGGAAACTGGGAGACTTGAGTGCAGAAGAGGAAAGCGGAATTCCACGTGTAGCGGTGAAATGCGTAGAGATGTGGAGGAACACCAGTGGCGAAGGCGGCTTTCTGGTCTGTAACTGACGCTGAGGCGCGAAAGCGTGGGGAGCAAACAGG

>OTU45GTGGGGAATATTGCACAATGGGCGCAAGCCTGATGCAGCGACGCCGCGTGGGGGATGAAGGCCTTCGGGTTGTAAACTCCTTTCGCTAGGGACGAAGCCTTATGGTGACGGTACCTGGAGAAGAAGCACCGGCTAACTACGTGCCAGCAGCCGCGGTAATACGTAGGGTGCGAGCGTTGTCCGGAATTACTGGGCGTAAAGAGCTCGTAGGTGGTTTGTCGCGTCGTCTGTGAAATCCCGGGGCTTAACTTCGGGCGTGCAGGCGATACGGGCATAACTTGAGTGCTGTAGGGGAGACTGGAATTCCTGGTGTAGCGGTGAAATGCGCAGATATCAGGAGGAACACCAATGGCGAAGGCAGGTCTCTGGGCAGTAACTGACGCTGAGGAGCGAAAGCATGGGTAGCGAACAGG

>OTU46GTGAGGAATATTGGTCAATGGGCGAGAGCCTGAACCAGCCAAGTAGCGTGAAGGATGAAGGTCCTACGGATTGTAAACTTCTTTTATAAGGGAATAAAACGCTCCACGTGTGGAGCCTTGTATGTACCTTATGAATAAGCATCGGCTAACTCCGTGCCAGCAGCCGCGGTAATACGGAGGATGCGAGCGTTATCCGGATTTATTGGGTTTAAAGGGAGCGCAGACGGGATGTTAAGTCAGCTGTGAAAGTTTGCGGCTCAACCGTAAAATTGCAGTTGATACTGGCGTTCTTGAGTGCAGTTGAGGTGTGCGGAATTCGTGGTGTAGCGGTGAAATGCTTAGATATCACGAAGAACTCCGATTGCGAAGGCAGCTCACTAAACTGTAACTGACGTTCATGCTCGAAAGTGTGGGTATCAAACAGG

>OTU47GTGGGGAATATTGCACAATGGGGGAAACCCTGATGCAGCAACGCCGCGTGAGTGATGAAGGCCTTCGGGTTGTAAAGCTCTGTTTCCAGGGACGATAATGACGGTACCTGGGGAGGAAGCCACGGCTAACTACGTGCCAGCAGCCGCGGTAATACGTAGGTGGCAAGCGTTGTCCGGATTTACTGGGCGTAAAGGGAGCGTAGGCGGATTTTTAAGTGGGATGTGAAATCCCCGGGCTCAACCCGGGGGCTGCATTCCAAACTGGAAATCTAGAGTGTCGGAGAGGAAAGCAGAATTCCTAGTGTAGCGGTGAAATGCGTAGAGATTAGGAAGAATACCAGTGGCGAAGGCGGCTTTCTGGACGATAACTGACGCTGAGGCTCGAAAGCGTGGGGAGCAAACAGG

>OTU48GTGGGGAATATTGGACAATGGGCGAAAGCCTGATCCAGCCATGCCGCGTGTGTGAAGAAGGTCTTCGGATTGTAAAGCACTTTAAGTTGGGAGGAAGGGCAGTAAGTTAATACCTTGCTGTTTTGACGTTACCAACAGAATAAGCACCGGCTAACTTCGTGCCAGCAGCCGCGGTAATACGAAGGGTGCAAGCGTTAATCGGAATTACTGGGCGTAAAGCGCGCGTAGGTGGTTCGTTAAGTTGGATGTGAAAGCCCCGGGCTCAACCTGGGAACTGCATCCAAAACTGGCGAGCTAGAGTATGGCAGAGGGTGGTGGAATTTCCTGTGTAGCGGTGAAATGCGTAGATATAGGAAGGAACACCAGTGGCGAAGGCGACCACCTGGGCTAATACTGACACTGAGGTGCGAAAGCGTGGGGAGCAAACAGG

>OTU49GTAAGGAATATTGGTCAATGGGGGGAACCCTGAACCAGCCATGCCGCGTGCAGGAAGACGGCCCTACGGGTTGTAAACTGCTTTTGTACGGGAATAACCCCCAATACGGGTATTGGGCTGAATGTACCGTAAGAATAAGGATCGGCTAACTCCGTGCCAGCAGCCGCGGTAATACGGAGGATCCGAGCGTTATCCGGAATTATTGGGTTTAAAGGGTGCGTAGGCGGCCCGGTAAGTCAGTGGTGAAAGACGGTGGCTCAACCATCGCAGTGCCGTTGATACTGTCGGGCTTGAATGTACTTGAGGTAGGCGGAATGTGGCAAGTAGCGGTGAAATGCATAGATATGCCACAGAACACCGATTGCGAAGGCAGCTTACTAAGGTATGATTGACGCTGAGGCACGAAAGCGTGGGGATCGAACAGG

>OTU50GTAGGGAATCTTCCACAATGGGCGAAAGCCTGATGGAGCAACGCCGCGTGGGTGAAGAAGGTCTTCGGATCGTAAAACCCTGTTGTTAGAGAAGAAAGTGCGTGAGAGTAACTGTTCACGTTTCGACGGTATCTAACCAGAAAGCCACGGCTAACTACGTGCCAGCAGCCGCGGTAATACGTAGGTGGCAAGCGTTATCCGGATTTATTGGGCGTAAAGGGAACGCAGGCGGTCTTTTAAGTCTGATGTGAAAGCCTTCGGCTTAACCGGAGTAGTGCATTGGAAACTGGGAGACTTGAGTGCAGAAGAGGAGAGTGGAACTCCATGTGTAGCGGTGAAATGCGTAGATATATGGAAGAACACCAGTGGCGAAAGCGGCTCTCTGGTCTGTAACTGACGCTGAGGTTCGAAAGCGTGGGTAGCAAACAGG

>OTU51GTGGGGAATATTGCACAATGGGCGAAAGCCTGATGCAGCAACGCCGCGTGAGTGATGACGGCCTTCGGGTTGTAAAACTCTGTCTTCAGGGACGATAATGACGGTACCTGAGGAGGAAGCCACGGCTAACTACGTGCCAGCAGCCGCGGTAATACGTAGGTGGCAAGCGTTGTCCGGATTTACTGGGCGTAAAGGGAGCGTAGGCGGATGTTTAAGTGGGATGTGAAATACTCGGGCTCAACTTGAGTGCTGCATTCCAAACTGGATATCTAGAGTGCAGGAGAGGAAAGGAGAATTCCTAGTGTAGCGGTGAAATGCGTAGAGATTAGGAAGAATACCAGTGGCGAAGGCGCCTTTCTGGACTGTAACTGACGCTGAGGCTCGAAAGCGTGGGGAGCAAACAGG

>OTU52GTGGGGAATATTGGGCAATGGGGGAAACCCTGACCCAGCAACGCCGCGTGAAGGAAGAAGGCTTTCGGGTTGTAAACTTCTTTTACCAGGGACGAAGGACGTGACGGTACCTGGAGAAAAAGCAACGGCTAACTACGTGCCAGCAGCCGCGGTAATACGTAGGTTGCAAGCGTTGTCCGGATTTACTGGGTGTAAAGGGCGTGTAGGCGGAGAAGCAAGTTGGGAGTGAAATCTATGGGCTCAACCCATAAACTGCTCTCAAAACTGTTTCCCTTGAGTATCGGAGAGGCAAGCGGAATTCCTAGTGTAGCGGTGAAATGCGTAGATATTAGGAGGAACACCAGTGGCGAAGGCGGCTTGCTGGACGACAACTGACGCTGAGGCGCGAAAGCGTGGGGAGCAAACAGG

>OTU53GTAGGGAATCTTCCGCAATGGACGAAAGTCTGACGGAGCAACGCCGCGTGAGTGAAGAAGGTTTTCGGATCGTAAAACTCTGTTGTCAGGGAAGAACAAGTGCTGTTCGAACAGGGCGGCACCTTGACGGTACCTGACCAGAAAGCCACGGCTAACTACGTGCCAGCAGCCGCGGTAATACGTAGGTGGCAAGCGTTGTCCGGAATTATTGGGCGTAAAGCGCGCGCAGGCGGCTTCTTAAGTCTGATGTGAAATCTTGCGGCTCAACCGCAAGCGGTCATTGGAAACTGGGAGGCTTGAGTGCAGAAGAGGAGAGTGGAATTCCACGTGTAGCGGTGAAATGCGTAGAGATGTGGAGGAACACCAGTGGCGAAGGCGACTCTCTGGTCTGTAACTGACGCTGAGGCGCGAAAGCGTGGGGAGCGAACAGG

>OTU54GTAGGGAATCTTCCACAATGGACGCAAGTCTGATGGAGCAACGCCGCGTGAGTGAAGAAGGTTTTCGGATCGTAAAGCTCTGTTGTTGGTGAAGAAAGATAGTGAGAGTAACTGCTCATTATTTGCCGGTAATCAACCAGAAAGTCACGGCTAACTACGTGCCAGCAGCCGCGGTAATACGTAGGTGGCAAGCGTTGTCCGGATTTATTGGGCGTAAAGCGAGCGCAGGCGGAAAGATAAGTCAGATGTGAAAGCCCTCGGCTTAACCGAGGAATAGCATCGGAAACTGTCTTTCTTGAGTGCAGAAGAGGAGAGTGGAACTCCATGTGTAGCGGTGGAATGCGTAGATATATGGAAGAACACCAGTGGCGAAGGCGGCTCTCTGGTCTGTAACTGACGCTGAGGCTCGAAAGCATGGGTAGCGAACAGG

>OTU55GTGAGGAATATTGGTCAATGGGCGAGAGCCTGAACCAGCCAAGTCGCGTGAAGGAAGACAGTCCTAAGGATTGTAAACTTCTTTTATACGGGAATAACGGGCGATACGAGTATTGCATTGAATGTACCGTAAGAATAAGCATCGGCTAACTCCGTGCCAGCAGCCGCGGTAATACGGAGGATGCGAGCGTTATCCGGATTTATTGGGTTTAAAGGGTGCGTAGGTTGTTCGGTAAGTCAGCGGTGAAACCTGAGCGCTCAACGTTCAGCCTGCCGTTGAAACTGCCGGGCTTGAGTTCAGTGGCGGCAGGCGGAATTCGTGGTGTAGCGGTGAAATGCATAGATATCACGAGGAACTCCGATTGCGAAGGCAGCTTGCCATACTGCGACTGACACTGAAGCACGAAGGCGTGGGTATCAAACAGG

>OTU56GTGGGGAATTTTGGACAATGGGCGAAAGCCTGATCCAGCAATGCCGCGTGCAGGATGAAGGCCTTCGGGTTGTAAACTGCTTTTGTACGGAACGAAAAAGCTTCTCCTAATACGAGAGGCCCATGACGGTACCGTAAGAATAAGCACCGGCTAACTACGTGCCAGCAGCCGCGGTAATACGTAGGGTGCGAGCGTTAATCGGAATTACTGGGCGTAAAGCGTGCGCAGGCGGTTATGTAAGACAGATGTGAAATCCCCGGGCTCAACCTGGGAACTGCATTTGTGACTGCATGGCTAGAGTACGGTAGAGGGGGATGGAATTCCGCGTGTAGCAGTGAAATGCGTAGATATGCGGAGGAACACCGATGGCGAAGGCAATCCCCTGGACCTGTACTGACGCTCATGCACGAAAGCGTGGGGAGCAAACAGG

>OTU57GTGGGGAATATTGCACAATGGGCGAAAGCCTGATGCAGCAACGCCGCGTGAGCGATGAAGGCCTTCGGGTCGTAAAGCTCTGTCCTCAAGGAAGATAATGACGGTACTTGAGGAGGAAGCCCCGGCTAACTACGTGCCAGCAGCCGCGGTAATACGTAGGGGGCTAGCGTTATCCGGAATTACTGGGCGTAAAGGGTGCGTAGGTGGTTTCTTAAGTCAGAGGTGAAAGGCTACGGCTCAACCGTAGTAAGCCTTTGAAACTGGGAAACTTGAGTGCAGGAGAGGAGAGTGGAATTCCTAGTGTAGCGGTGAAATGCGTAGATATTAGGAGGAACACCAGTTGCGAAGGCGGCTCTCTGGACTGTAACTGACACTGAGGCACGAAAGCGTGGGGAGCAAACAGG

>OTU58GTGGGGGATATTGCACAATGGAGGAAACTCTGATGCAGCAACGCCGCGTGAGGGAAGAAGGTTTTCGGATTGTAAACCTCTGTCCTCGGGGACGATAATGACGGTACCCGAGGAGGAAGCTCCGGCTAACTACGTGCCAGCAGCCGCGGTAATACGTAGGGAGCAAGCGTTGTCCGGATTTACTGGGTGTAAAGGGTGCGTAGGCGGCAGTGCAAGTCAGGTGTGAAAACTATGGGCTTAACCCATAGATTGCACTTGAAACTGTGTTGCTTGAGTGAAGTAGAGGCAAGCGGAATTCCCGGTGTAGCGGTGAAATGCGTAGAGATCGGGAGGAACACCAGTGGCGAAGGCGGCTTGCTGGGCTTTAACTGACGCTGAGGCACGAAAGCGTGGGTAGCAAACAGG

>OTU59GTGAGGAATATTGGTCAATGGGCGAGAGCCTGAACCAGCCAAGTAGCGTGAAGGATGACTGCCCTATGGGTTGTAAACTTCTTTTATAAAGGAATAAAGTCGGGTATGGATACCCGTTTGCATGTACTTTATGAATAAGGATCGGCTAACTCCGTGCCAGCAGCCGCGGTAATACGGAGGATCCGAGCGTTATCCGGATTTATTGGGTTTAAAGGGAGCGTAGATGGATGTTTAAGTCAGTTGTGAAAGTTTGCGGCTCAACCGTAAAATTGCAGTTGATACTGGATATCTTGAGTGCAGTTGAGGCAGGCGGAATTCGTGGTGTAGCGGTGAAATGCTTAGATATCACGAAGAACTCCGATTGCGAAGGCAGCCTGCTAAGCTGCAACTGACATTGAGGCTCGAAAGTGTGGGTATCAAACAGG

>OTU60GTGAGGAATATTGGTCAATGGGCGCTAGCCTGAACCAGCCAAGTAGCGTGAAGGATGACTGCCCTATGGGTTGTAAACTTCTTTTATATGGGAATAAAGTGCAGTATGTATACTGCTTTGCATGTACCTTATGAATAAGGATCGGCTAACTCCGTGCCAGCAGCCGCGGTAATACGGAGGATCCGAGCGTTATCCGGATTTATTGGGTTTAAAGGGAGCGTAGGCGGGTTGTTAAGTCAGTTGTGAAAGTTTGCGGCTCAACCGTAAAATTGCAGTTGATACTGGCATCCTTGAGTACAGTAGAGGTAGGCGGAATTCGTGGTGTAGCGGTGAAATGCTTAGATATCACGAAGAACTCCGATTGCGAAGGCAGCCTGCTGGACTGTAACTGACGCTGATGCTCGAAAGTGTGGGTATCAAACAGG

>OTU61GTGGGGAATATTGGACAATGGGGGCAACCCTGATCCAGCCATGCCGCGTGTGTGAAGAAGGCCTTTTGGTTGTAAAGCACTTTAAGCAGGGAGGAGAGGCTAATGGTTAATACCCATTAGATTAGACGTTACCTGCAGAATAAGCACCGGCTAACTCTGTGCCAGCAGCCGCGGTAATACAGAGGGTGCGAGCGTTAATCGGAATTACTGGGCGTAAAGCGAGTGTAGGTGGCTCATTAAGTCACATGTGAAATCCCCGGGCTTAACCTGGGAACTGCATGTGATACTGGTGGTGCTAGAATATGTGAGAGGGAAGTAGAATTCCAGGTGTAGCGGTGAAATGCGTAGAGATCTGGAGGAATACCGATGGCGAAGGCAGCTTCCTGGCATAATATTGACACTGAGATTCGAAAGCGTGGGTAGCAAACAGG

>OTU62GTGGGGAATATTGCACAATGGGCGCAAGCCTGATGCAGCGACGCCGCGTGGGGGATGACGGCCTTCGGGTTGTAAACTCCTTTCGCTAGGGACGAAGCTTTTTGTGACGGTACCTAGATAAGAAGCACCGGCTAACTACGTGCCAGCAGCCGCGGTAATACGTAGGGTGCGAGCGTTGTCCGGAATTACTGGGCGTAAAGGGCTCGTAGGTGGTTTGTCGCGTCGTCTGTGAAATTCCGGGGCTTAACTCCGGGCGTGCAGGCGATACGGGCATAACTTGAGTACTGTAGGGGTAACTGGAATTCCTGGTGTAGCGGTGAAATGCGCAGATATCAGGAGGAACACCGATGGCGAAGGCAGGTTACTGGGCAGTTACTGACGCTGAGGAGCGAAAGCATGGGTAGCGAACAGG

>OTU63GTGGGGAATATTGCGCAATGGGGGAAACCCTGACGCAGCAACGCCGCGTGGGTGATGAAGGTTTTCGGATCGTAAAACCCTGTTTTCTGGGACGATAATGACGGTACCAGATGAGGAAGCCACGGCTAACTACGTGCCAGCAGCCGCGGTAATACGTAGGTGGCAAGCGTTGTCCGGATTTACTGGGCGTAAAGGGTGCGTAGGCGGATGCTTAAGTGAGATGTGAAATACCTAGGCTTAACTTAGGGGCTGCATTTCAAACTGGGCATCTAGAGTACAGGAGAGGGAAACGGAATTCCTAGTGTAGCGGTGAAATGCGTAGAGATTAGGAAGAACACCAGTGGCGAAGGCGGTTTCCTGGACTGTAACTGACGCTGAGGCACGAAAGCGTGGGTAGCAAACAGG

>OTU64GTGGGGAATATTGGACAATGGGCGCAAGCCTGATCCAGCCATACCGCGTGGGTGAAGAAGGCCTTCGGGTTGTAAAGCCCTTTTGTTGGGAAAGAAATCCAGCTGGTTAATACCCGGTTGGGATGACGGTACCCAAAGAATAAGCACCGGCTAACTTCGTGCCAGCAGCCGCGGTAATACGAAGGGTGCAAGCGTTACTCGGAATTACTGGGCGTAAAGCGTGCGTAGGTGGTCGTTTAAGTCTGTTGTGAAAGCCCTGGGCTCAACCTGGGAACTGCAGTGGAAACTGGACGACTAGAGTGTGGTAGAGGGTAGCGGAATTCCTGGTGTAGCAGTGAAATGCGTAGAGATCAGGAGGAACATCCATGGCGAAGGCAGCTACCTGGACCAACACTGACACTGAGGCACGAAAGCGTGGGGAGCAAACAGG

>OTU65GTGGGGAATATTGCACAATGGGGGAAACCCTGATGCAGCAACGCCGCGTGAAGGAAGACGGTTTTCGGATTGTAAACTTCTGTTCTTAGTGAAGAATAATGACGGTAGCTAAGGAGCAAGCCACGGCTAACTACGTGCCAGCAGCCGCGGTAATACGTAGGTGGCAAGCGTTGTCCGGAATTACTGGGTGTAAAGGGAGCGTAGGCGGGAAGCCAAGTCAGCTGTGAAAACTACGGGCTTAACTTGTAGACTGCAGTTGAAACTGGTTTTCTTGAGTGAAGTAGAGGTTGGCGGAATTCCGAGTGTAGCGGTGAAATGCGTAGATATTCGGAGGAACACCGGTGGCGAAGGCGGCCAACTGGGCTTTAACTGACGCTGAGGCTCGAAAGTGTGGGGAGCAAACAGG

>OTU66GTAGGGAATTTTTCACAATGGGCGAAAGCCTGATGGAGCAATGCCGCGTGTTTGATGACGGCCTTTGGGTTGTAAAAAACTGTTATATGGGATGAAAAAAATAGATAGGAAATGATCTATTATTGACAGTACCATATTAGAAAGCCACGGCTAACTACGTGCCAGCAGCCGCGGTAATACGTAGGTGGCAAGCATTATCCGGAATTATTGGGTGTAAAGGGAGCGTAGACGGTTTTATAAGTCTGAGGTTAAATGCATAAGCTTAACTTATGCCTGCCTTAGAAACTATGAGACTAGAATGTGATAGAGGACAATGGAATTCCATGTGTAGCGGTGGAATGCATAGATATATGGAGGAACACCAATTGCGAAAGCAGTTGTCTGGGTCATTATTGACGTTGAGGCTCGAAAGCATGGGGAGCAAATAGG

>OTU67GTAGGGAATCTTCGGCAATGGACGGAAGTCTGACCGAGCAACGCCGCGTGAGTGAAGAAGGTTTTCGGATCGTAAAGCTCTGTTGTAAGAGAAGAACGAGTGTGAGAGTGGAAAGTTCACACTGTGACGGTATCTTACCAGAAAGGGACGGCTAACTACGTGCCAGCAGCCGCGGTAATACGTAGGTCCCGAGCGTTGTCCGGATTTATTGGGCGTAAAGCGAGCGCAGGCGGTTAGATAAGTCTGAAGTTAAAGGCTGTGGCTTAACCATAGTACGCTTTGGAAACTGTTTAACTTGAGTGCAAGAGGGGAGAGTGGAATTCCATGTGTAGCGGTGAAATGCGTAGATATATGGAGGAACACCGGTGGCGAAAGCGGCTCTCTGGCTTGTAACTGACGCTGAGGCTCGAAAGCGTGGGGAGCAAACAGG

>OTU68GTGGGGAATATTGCGCAATGGGGGAAACCCTGACGCAGCAACGCCGCGTGAGTGAAGAAGGCCTTCGGGTCGTAAAACTCTTTGATAAGGGAAGAAGAAGTGACAGTACCTTAAGAAAAAGCCCCGGCTAACTACGTGCCAGCAGCCGCGGTAATACGTAGGGGGCAAGCGTTGTCCGGAATTACTGGGCGTAAAGGGTGCGTAGGCGGTCAAATAAGTCAGATGTGAAAGTCCAGGGCTCAACCGTGGAATCGCATTTGAAACTGTTTGACTTGAGTGCAGGAGAGGAGAGTGGAATTTCTAGTGTAGCGGTGAAATGCGTAGATATTAGGAAGAACACCAGTGGCGAAGGCGGCTCTCTGGACTGTAACTGACGCTGAGGCACGAAAGCGTGGGAAGCGAACAGG

>OTU69GTGGGGAATATTGGACAATGGGCGAAAGCCTGATCCAGCAATGCCGCGTGGGTGAAGAAGGTCTTCGGATCGTAAAGCCCTTTCGACGGGGACGATGATGACGGTACCCGTAGAAGAAGCCCCGGCTAACTTCGTGCCAGCAGCCGCGGTAATACGAAGGGGGCTAGCGTTGCTCGGAATTACTGGGCGTAAAGGGCGCGTAGGCGGCGGCCCAAGTCAGGCGTGAAATTCCTGGGCTCAACCTGGGGACTGCGCTTGATACTGGGTTGCTTGAGGATGGAAGAGGCTCGTGGAATTCCCAGTGTAGAGGTGAAATTCGTAGATATTGGGAAGAACACCGGTGGCGAAGGCGGCGAGCTGGTCCATTACTGACGCTGAGGCGCGACAGCGTGGGGAGCAAACAGG

>OTU70GTGAGGAATATTGGTCAATGGACGCAAGTCTGAACCAGCCAAGTAGCGTGCAGGATGACGGCCCTCCGGGTTGTAAACTGCTTTTAGTTGGGAATAAAAGGGGGCTCGTGAGCCCCGTATTGTATGTACCATCAGAAAAAGGACCGGCTAATTCCGTGCCAGCAGCCGCGGTAATACGGAAGGTCCGGGCGTTATCCGGATTTATTGGGTTTAAAGGGAGCGTAGGCCGGCTTTTAAGTCAGCTGTGAAAGTCCGCGGCTCAACCGTGGAATTGCAGTTGAAACTGGAGGCCTTGAGTGCACACAGGGATGCCGGAATTCATGGTGTAGCGGTGAAATGCTTAGATATCATGAAGAACTCCGATCGCGAAGGCAAGTGTCCGGGGTGCAACTGACGCTGAGGCTCGAAAGTGCGGGTATCAAACAGG

>OTU71GTAGGGAATCTTCGGCAATGGGGGCAACCCTGACCGAGCAACGCCGCGTGAGTGAAGAAGGTTTTCGGATCGTAAAGCTCTGTTGTAAGAGAAGAACGTGTGTGAGAGTGGAAAGTTCACACAGTGACGGTAACTTACCAGAAAGGGACGGCTAACTACGTGCCAGCAGCCGCGGTAATACGTAGGTCCCGAGCGTTGTCCGGATTTATTGGGCGTAAAGCGAGCGCAGGCGGTTTAATAAGTCTGAAGTTAAAGGCAGTGGCTTAACCATTGTTCGCTTTGGAAACTGTTAGACTTGAGTGCAGAAGGGGAGAGTGGAATTCCATGTGTAGCGGTGAAATGCGTAGATATATGGAGGAACACCGGTGGCGAAAGCGGCTCTCTGGTCTGTAACTGACGCTGAGGCTCGAAAGCGTGGGGAGCAAACAGG

>OTU72GTGGGGAATATTGCACAATGGGGGGAACCCTGATGCAGCCATGCCGCGTGAATGAAGAAGGCCTTCGGGTTGTAAAGTTCTTTCGGTGATGAGGAAGGGTGATGTTTTAATAGAGCATTACATTGACGTTAGTCACAGAAGAAGCACCGGCTAACTCCGTGCCAGCAGCCGCGGTAATACGGAGGGTGCGAGCGTTAATCGGAATGACTGGGCGTAAAGGGCACGCAGGCGGTGACTTAAGTGAGATGTGAAAGCCCCGAGCTTAACTTGGGAATTGCATTTCATACTGGGTTGCTAGAGTATTTTAGGGAGGGGTAGAATTCCACGTGTAGCGGTGAAATGCGTAGAGATGTGGAGGAATACCGAAGGCGAAGGCAGCCCCTTGGGAAAATACTGACGCTCATGTGCGAAAGCGTGGGGAGCAAACAGG

>OTU73GTAAGGAATTTTCCACAATGAGCGAAAGCTTGATGGAGCGACACAGCGTGCAGGATGAAGTTCTTCGGAATGTAAACTGCTGTTATAAGGGAAGAAAAAATAGAATAGGAAATGATTTTATCTTGACGGTACCTTATTAGAAAGCGACGGCAAACTATGTGCCAGCAGCCGCGGTAATACATAGGTCGCAAGCGTTATCCGGAATTATTGGGCGTAAAGCGTCCGTAGGTTTTTTGCTAAGTCTGGAGTTAAATGCTGAAGCTCAACTTCAGTCCGCTTTGGATACTGGCAAAATAGAATTATAAAGAGGTTAGCGGAATTCCTAGTGAAGCGGTGGAATGCGTAGATATTAGGAAGAACACCAATAGGCGAAGGCAGCTAACTGGTTATATATTGACACTAAGGGACGAAAGCGTGGGGAGCAAACAGG

>OTU74GTGGGGAATCTTGCGCAATGGGGGGAACCCTGACGCAGCGACGCCGCGTGCGGGACGAAGGCCCTCGGGTCGTAAACCGCTTTCAGCAGGGATGAGAAGAGACGGTACCTGCAGAAGAAGCCCCGGCTAACTACGTGCCAGCAGCCGCGGTAATACGTAGGGGGCGAGCGTTATCCGGATTCATTGGGCGTAAAGCGCGCGTAGGCGGCCCGGCAGGCAGGGGGTCAAATGGCGGGGCTCAACCCCGTCCCGCCCCCTGAACCGCCGGGCTCGGGTCCGGTAGGGGAGGGTGGAACACCCGGTGTAGCGGTGGAATGCGCAGATATCGGGTGGAACACCGGTGGCGAAGGCGGCCCTCTGGGCCGAGACCGACGCTGAGGCGCGAAAGCTGGGGGAGCGAACAGG

>OTU75GTGAGGAATATTGGTCAATGGGCGGGAGCCTGAACCAGCCAAGTCGCGTGAGGGAAGAAGGTGCTAAGCATTGTAAACCTCTTTTGCCGGGGAACAAAAGATCCCACGCGTGGGATTATGAGTGTACCCGGAGAAAAAGCATCGGCTAACTCCGTGCCAGCAGCCGCGGTAATACGGAGGATGCGAGCGTTATCCGGATTTATTGGGTTTAAAGGGTGCGCAGGCGGCTCGTCAAGTCAGCGGTAAAATGTCGGGGCTCAACCCCGGCCGGCCGTTGAAACTGACGAGCTGGAGTTGGAGAGAAGTACGCGGAATTCGTGGTGTAGCGGTGAAATGCTTAGATATCACGAAGAACTCCGATTGCGAAGGCAGCGTACCGGCTCCATACTGACGCTGAGGCACGAAAGCGTGGGGATCGAACAGG

>OTU76GTGGGGAATATTGCACAATGGGCGAAAGCCTGATGCAGCGACGCCGCGTGGGGGATGACGGTCTTCGGATTGTAAACCCCTTTCAGTAGGGACGAAGCGCAAGTGACGGTACCTGCAGAAGAAGCACCGGCTAACTACGTGCCAGCAGCCGCGGTAATACGTAGGGTGCGAGCGTTGTCCGGAATTACTGGGCGTAAAGAGCTCGTAGGCGGTTTGTCACGTCGTCTGTGAAATCCCTCGGCTTAACCGGGGGCGTGCAGGCGATACGGGCAGACTTGAGTACTACAGGGGAGACTGGAATTCCTGGTGTAGCGGTGAAATGCGCAGATATCAGGAGGAACACCGGTGGCGAAGGCGGGTCTCTGGGTAGTAACTGACGCTGAGGAGCGAAAGCATGGGTAGCGAACAGG

>OTU77GTAGGGAATCTTCCACAATGGACGAAAGTCTGATGGAGCAACGCCGCGTGAGTGAAGAAGGTTTTCGGATCGTAAAACTCTGTTGTTGGAGAAGAACGTATTTGATAGTAACTGATCAGGTAGTGACGGTATCCAACCAGAAAGCCACGGCTAACTACGTGCCAGCAGCCGCGGTAATACGTAGGTGGCAAGCGTTGTCCGGATTTATTGGGCGTAAAGCGAGCGCAGGCGGTTTCTTAAGTCTGATGTGAAAGCCTTCGGCTCAACCGAAGAAGTGCATCGGAAACTGGGAAACTTGAGTGCAGAAGAGGACAGTGGAACTCCATGTGTAGCGGTGAAATGCGTAGATATATGGAAGAACACCAGTGGCGAAGGCGGCTGTCTGGTCTGTAACTGACGCTGAGGCTCGAAAGCATGGGTAGCAAACAGG

>OTU78GTGGGGAATTTTGGACAATGGGCGCAAGCCTGATCCAGCAATGCCGCGTGCAGGATGAAGGCCTTCGGGTTGTAAACTGCTTTTGTACGGAGCGAAAAGGTCTCTTCTAATACAGGAGGCCCATGACGGTACCGTAAGAATAAGCACCGGCTAACTACGTGCCAGCAGCCGCGGTAATACGTAGGGTGCAAGCGTTAATCGGAATTACTGGGCGTAAAGCGTGCGCAGGCGGTGTTGTAAGACAGAGGTGAAATCCCCGGGCTCAACCTGGGAACTGCCTTTGTGACTGCAACGCTAGAGTACGGCAGAGGGGGATGGAATTCCGCGTGTAGCAGTGAAATGCGTAGATATGCGGAGGAACACCGATGGCGAAGGCAATCCCCTGGGCCTGTACTGACGCTCATGCACGAAAGCGTGGGGAGCAAACAGG

>OTU79GTAGGGAATCTTCCACAATGGACGCAAGTCTGATGGAGCAACGCCGCGTGTATGAAGAAGGTCTTCGGATCGTAAAATACTGTTGTCAGAGAAGAACACGTGATAGAGTAACTGCTATGGCGCTGACGGTATCTGACCAGCAAGTCACGGCTAACTACGTGCCAGCAGCCGCGGTAATACGTAGGTGGCAAGCGTTGTCCGGATTTATTGGGCGTAAAGGGAACGCAGGCGGTCTTTTAAGTCTGATGTGAAAGCCTTCGGCTTAACCGGAGAAGTGCATTGGAAACTGGAAGACTTGAGTGCAGAAGAGGAGAGTGGAACTCCATGTGTAGCGGTGAAATGCGTAGATATATGGAAGAACACCAGTGGCGAAAGCGGCTCTCTGGTCTGTAACTGACGCTGAGGTTCGAAAGCGTGGGGAGCGAACAGG

>OTU80GTGGGGAATATTGCACAATGGGGGAAACCCTGATGCAGCGACGCCGCGTGAGCGAAGAAGTATTTCGGTATGTAAAGCTCTATCAGCAGGGAAGATAATGACGGTACCTGACTAAGAAGCCCCGGCTAACTACGTGCCAGCAGCCGCGGTAATACGTAGGGGGCAAGCGTTATCCGGATTTACTGGGTGTAAAGGGAGCGTAGACGGCAAGGCAAGTCTGATGTGAAAACCCAGGGCTTAACCCTGGGACTGCATTGGAAACTGTCTGGCTCGAGTGCCGGAGAGGTAAGCGGAATTCCTAGTGTAGCGGTGAAATGCGTAGATATTAGGAAGAACACCAGTGGCGAAGGCGGCTTACTGGACGGTAACTGACGTTGAGGCTCGAAAGCGTGGGGAGCAAACAGG

>OTU81GTGAGGAATATTGGACAATGGGCGCAAGCCTGATCCAGCCATGCCGCGTGCAGGAAGACTGCCCTATGGGTTGTAAACTGCTTTTATACAGGAAGAATAAGCCTTACGTGTAAGGTGATGACGGTACTGTAAGAATAAGGACCGGCTAACTCCGTGCCAGCAGCCGCGGTAATACGGAGGGTCCAAGCGTTATCCGGAATTATTGGGTTTAAAGGGTCCGTAGGTGGATAATTAAGTCAGGGGTGAAAGTTTGCAGCTCAACTGTAAAATTGCCTTTGATACTGGTTATCTTGAATTGTATTGAAGTAGGCGGAATATGTAGTGTAGCGGTGAAATGCATAGATATTACATAGAACACCAATTGCGAAGGCAGCTTACTAAGTACCAATTGACACTGATGGACGAAAGCGTGGGTAGCGAACAGG

>OTU82GTGGGGAATATTGCGCAATGGGCGAAAGCCTGACGCAGCGACGCCGCGTGAGGGATGAAGGTCTTCGGATCGTAAACCTCTGTCAGCAGGGAAGAACGGTCACTGTGCTAATCAGCAGTGAATTGACGGTACCTGCAAAGGAAGCACCGGCTAACTCCGTGCCAGCAGCCGCGGTAATACGGAGGGTGCGAGCGTTAATCGGAATTACTGGGCGTAAAGCGCTCGTAGGCGGTATGTCAAGTCAAGGGTGAAATCCCCGCGCTCAACGTGGGAACTGCCTTTGAAACTGGCAAACTGGAGTGTGTGAGAGGATAGTGGAATTCCAGGTGTAGGAGTGAAATCCGTAGATATCTGGAGGAACATCAGTGGCGAAGGCGACTATCTGGCACATAACTGACGCTGAGGAGCGAAAGCGTGGGTAGCAAACAGG

>OTU83GTGGGGAATATTGCACAATGGGCGAAAGCCTGATGCAGCAACGCCGCGTGAGTGATGAAGGCCTTCGGGTCGTAAAGCTCTTTGATCAGGGATGATAATGACAGTACCTGAAAAACAAGCCACGGCTAACTACGTGCCAGCAGCCGCGGTAATACGTAGGTGGCGAGCGTTGTCCGGAATTACTGGGCGTAAAGGATGCGTAGGTGGATATTTAAGTGGGATGTGAAATCCCCGGGCTCAACCCGGGAACTGCATTCCAAACTGGGTATCTAGAGTGCAGGAGAGGAAAGCGGAATTCCTAGTGTAGCGGTGAAATGCGTAGATATTAGGAGGAACACCAGTGGCGAAGGCGGCTTTCTGGACTGTAACTGACACTGAGGCATGAAAGCGTGGGTAGCAAACAGG

>OTU84GTAGGGAATATTGGACAATGGGGGCAACCCTGATCCAGCAATGCCGCGTGTGTGAAGAAGGCCTTAGGGTTGTAAAACACTTTCAACTGTGAAGATGATGACAGTAGCAGTAGAAGAAGCTCCGGCTAACTCCGTGCCAGCAGCCGCGGTAATACGGAGGGAGCTAGCGTTGTTCGGAATTACTGGGCGTAAAGAGCACGTAGGCGGCTAGATGCGTCAGGGGTGAAATCCCGAGGCTTAACCTCGGAACTGCCTCTGATACGGTTTAGCTAGAATATCGTAGAGGTTAGTGGAATTCCAAGTGTAGAGGTGAAATTCGTAGATATTTGGAAGAACATCAGTGGCGAAGGCGACTAACTGGGCGATTATTGACGCTGAGGTGCGAAAGCGTGGGGAGCAAACGGG

>OTU85GTAGGGAATCTTCCACAATGGACGCAAGTCTGATGGAGCAACGCCGCGTGTGTGAAGAAGGTCTTCGGATCGTAAAACACTGTTGTTAGAGAAGAATACAAGTGAGAGTAACTGTTCACTTGCTGACGGTATCTAACCAGAAAGCCACGGCTAACTACGTGCCAGCAGCCGCGGTAATACGTAGGTGGCAAGCGTTGTCCGGATTTATTGGGCGTAAAGGGAACGCAGGCGGTTTTTTAAGTCTGATGTGAAAGCCTTCGGCTTAACCGGAGTAGTGCATTGGAAACTGGAAGACTTGAGTGCAGAAGAGGAGAGTGGAACTCCATGTGTAGCGGTGAAATGCGTAGATATATGGAAGAACACCAGTGGCGAAAGCGGCTCTCTGGTCTGTAACTGACGCTGAGGTTCGAAAGCGTGGGTAGCAAACAGG

>OTU86GTAGGGAATCTTCCGCAATGGGCGAAAGCCTGACGGAGCAACGCCGCGTGAGTGATGAAGGTCTTCGGATCGTAAAACTCTGTTATTAGGGAAGAACAAATGTGTAAGTAACTATGCACGTCTTGACGGTACCTAATCAGAAAGCCACGGCTAACTACGTGCCAGCAGCCGCGGTAATACGTAGGTGGCAAGCGTTATCCGGAATTATTGGGCGTAAAGCGCGCGTAGGCGGTTTTTTAAGTCTGATGTGAAAGCCCACGGCTCAACCGTGGAGGGTCATTGGAAACTGGAAAACTTGAGTGCAGAAGAGGAAAGTGGAATTCCATGTGTAGCGGTGAAATGCGCAGAGATATGGAGGAACACCAGTGGCGAAGGCGACTTTCTGGTCTGTAACTGACGCTGATGTGCGAAAGCGTGGGGATCAAACAGG

>OTU87GTGAGGAATATTGGTCAATGGGCTTACGCCTGAACCAGCCAAGTCGCGTGAGGGAAGACTGCCCTATGGGTTGTAAACCTCTTTTGTCAGGGAGCAACGCCGGCTACGTGTAGCCGGACTGAGAGTACCTGAAGAAAAAGCATCGGCTAACTCCGTGCCAGCAGCCGCGGTAATACGGAGGATGCGAGCGTTATCCGGATTTATTGGGTTTAAAGGGTGCGTAGGCGGAATGCCAAGTCAGCGGTAAAATCGCGGGGCTCAACCTCGCTCCGCCGTTGAAACTGGCGTTCTTGAGTGGGCGAGAAGTATGCGGAATGCGTGGTGTAGCGGTGAAATGCATAGATATCACGCAGAACTCCGATTGCGAAGGCAGCATACCGGCGCCCAACTGACGCTGAAGCACGAAAGCGTGGGTATCGAACAGG

>OTU88GTGGGGAATATTGCACAATGGAGGAAACTCTGATGCAGCGACGCCGCGTGAGTGAAGAAGTATTTCGGTATGTAAAGCTCTATCAGCAGGGAAGATAGTGACGGTACCTGACTAAGAAGCACCGGCTAAATACGTGCCAGCAGCCGCGGTAATACGTATGGTGCAAGCGTTATCCGGATTTACTGGGTGTAAAGGGAGTGTAGGTGGCATGGCAAGTCAGAAGTGAAAGCCCAGGGCTCAACCCTGGGACTGCTTTTGAAACTGCCGAGCTAGAGTGCAGGAGAGGTAAGTGGAATTCCTAGTGTAGCGGTGAAATGCGTAGATATTAGGAGGAACACCAGCGGCGAAGGCGGCTTACTGGACTGTAACTGACACTGAGGCTCGAAAGCGTGGGGAGCAAACAGG

>OTU89GTGGGGAATATTGGGCAATGGGCGCAAGCCTGACCCAGCAACGCCGCGTGAAGGAAGAAGGCTTTCGGGTTGTAAACTTCTTTTGTCAGGGACGAAGCAAGTGACGGTACCTGACGAATAAGCCACGGCTAACTACGTGCCAGCAGCCGCGGTAATACGTAGGTGGCAAGCGTTATCCGGATTTATTGGGTGTAAAGGGCGTGTAGGCGGGATTGCAAGTCAGATGTGAAAACCACGGGCTCAACCTGTGGCCTGCATTTGAAACTGTAGTTCTTGAGTGTCGGAGAGGCAATCGGAATTCCGTGTGTAGCGGTGAAATGCGTAGATATACGGAGGAACACCAGTGGCGAAGGCGGATTGCTGGACGATAACTGACGCTGAGGCGCGAAAGCGTGGGGAGCAAACAGG

>OTU90GTGGGGAATATTGCACAATGGGGGAAACCCTGATGCAGCAACGCCGCGTGAAGGAAGACGGTTTTCGGATTGTAAACTTCTTTTCTTAGTGAAGAAGCAAGTGACGGTAGCTAAGGAATAAGCATCGGCTAACTACGTGCCAGCAGCCGCGGTAATACGTAGGATGCGAGCGTTATCCGGAATTACTGGGTGTAAAGGGAGCGCAGGCGGGACTGCAAGTTGGATGTGAAATACCGCAGCTTAACTGCGGAGCTGCATCCAAAACTGTAGTTCTTGAGTGGAGTAGAGGCAAGCGGAATTCCGAGTGTAGCGGTGAAATGCGTAGATATTCGGAGGAACACCAGTGGCGAAGGCGGCTTGCTGGGCTCTAACTGACGCTGAGGCTCGAAAGTGTGGGGAGCAAACAGG

>OTU91GTGAGGAATATTGGTCAATGGAGGCAACTCTGAACCAGCCAAGTCGCGTGAAGGATGAAGGTCTTATGGATTGTAAACTTCTTTTGCAAGGGAATAAAGTGGGGGACGTGTCCTCCTTTGCATGTACCTTGCGAATAAGGATCGGCTAACTCCGTGCCAGCAGCCGCGGTAATACGGAGGATCCGAGCGTTATCCGGATTTATTGGGTTTAAAGGGTGCGCAGGCGGGATGTTAAGTCGGCGGTGAAATTTTGCAGCTCAACTGTAAAAGTGCCTTCGATACTGGCTTTCTTGAGTGTGGATGAAGTAGGCGGAATTTGTGGTGTAGCGGTGAAATGCTTAGATATCACGAGGAACTCCGATTGCGCAGGCAGCTTACTAAACCACTACTGACGCTCATGCACGAAGGCGTGGGGATCAAACAGG

>OTU92GTGGGGAATATTGGACAATGGGCGAAAGCCTGATCCAGCAATGCCGCGTGAGTGATGAAGGCCTTAGGGTTGTAAAGCTCTTTTACCAGGGATGATAATGACAGTACCTGGAGAATAAGCTCCGGCTAACTCCGTGCCAGCAGCCGCGGTAATACGGAGGGAGCTAGCGTTGTTCGGAATTACTGGGCGTAAAGCGCACGTAGGCGGCTTTTCAAGTCAGGGGTGAAATCCCGGGGCTCAACCCCGGAACTGCCCTTGAAACTGGATGGCTAGAATACTGGAGAGGTGAGTGGAATTCCGAGTGTAGAGGTGAAATTCGTAGATATTCGGAAGAACACCAGTGGCGAAGGCGACTCACTGGACAGTTATTGACGCTGAGGTGCGAAAGCGTGGGGAGCAAACAGG

>OTU93GTAGGGAATTTTCGTCAATGGGCGCAAGCCTGAACGAGCAATGCCGCGTGAGTGAGGAAGGCCTTCGGGTCGTAAAGCTCTGTTGCGAGGGAAAAAGGAACGGAAGAGGAAATGATTCCGTTTTGATGGTACCTCGCCAGAAAGTCACGGCTAACTACGTGCCAGCAGCCGCGGTAATACGTAGGTGGCAAGCGTTATCCGGAATGATTGGGCGTAAAGGGTGCGCAGGCGGCATATCAAGTCTGAAGTGAAAGGTACGGGCTCAACCTGTACAGGCTTTGGAAACTGGTATGCTCGAGGACAGGAGAGGGCGGTGGAACTCCACGTGTAGCGGTAAAATGCGTAGAGATGTGGAAGAACACCAGTGGCGAAGGCGGCCGCCTGGCCTGTAACTGACGCTCAGGCACGAAAGCGTGGGGAGCAAATAGG

>OTU94GTGGGGGATATTGCACAATGGGGGAAACCCTGATGCAGCAACGCCGCGTGAAGGAAGAAGGTCTTCGGATTGTAAACTTTTGTCCTTGGTGAAGATAATGACGGTAGCCAAGGAGGAAGCTCCGGCTAACTACGTGCCAGCAGCCGCGGTAATACGTAGGGAGCGAGCGTTGTCCGGATTTACTGGGTGTAAAGGGTGCGTAGGCGGAAGAGCAAGTCAGTAGTGAAATCCATGGGCTTAACCCGTGAACTGCTATTGAAACTGTTCTTCTTGAGTGAAGTAGAGGTAGGCGGAATTCCCGGTGTAGCGGTGAAATGCGTAGAGATCGGGAGGAACACCAGTGGCGAAGGCGGCCTACTGGGCTTTAACTGACGCTGAGGCACGAAAGCATGGGTAGCAAACAGG

>OTU95GTGGGGAATATTGCACAATGGGCGGAAGCCTGATGCAGCAACGCCGCGTGAGTGAAGAAGGTTTTCGGATTGTAAAGCTCTGTCATCCGGGACGATAATGACGGTACCGGATGAGGAAGCCACGGCTAACTACGTGCCAGCAGCCGCGGTAATACGTAGGTGGCGAGCGTTGTCCGGAATTACTGGGCGTAAAGGGTGCGCAGGCGGACATTTAAGTGAGATGTGAAATACCCGGGCTTAACCCGGGCAGTGCATTTCAAACTGGGTGTCTGGAGTGCAGGAGAGGAGAACGGAATTCCTAGTGTAGCGGTGAAATGCGTAGAGATTAGGAAGAACACCAGTGGCGAAGGCGGTTCTCTGGACTGTAACTGACGCTGAGGCACGAAAGCGTGGGTAGCAAACAGG

>OTU96GTGAGGAATATTGGTCAATGGTCGGGAGACTGAACCAGCCAAGCCGCGTGAGGGAAGAAGGCGCTAAGCGTCGTAAACCTCTTTTGTCGGGGAACAAACGAAGGCACGTGTGCCTTTCTGAGTGTACCCGAAGAAAAAGCATCGGCTAACTCCGTGCCAGCAGCCGCGGTAATACGGAGGATGCGAGCGTTATCCGGATTTATTGGGTTTAAAGGGTGCGCAGGCGGGCTGTTAAGTCAGCGGTAAAAGCCCGGGGCTCAACCCCGGCGAGCCGTTGAAACTGGCGGTCTTGAGTTGGAGAGAAGTATGCGGAATGCGCAGTGTAGCGGTGAAATGCTTAGATATTGCGCAGAACTCCGATTGCGAAGGCAGCATACCGGCTCCATACTGACGCTCAGGCACGAAAGTGCGGGGATCGAACAGG

>OTU97GTGGGGAATTTTCCGCAATGGGCGAAAGCCTGACGGAGCAATGCCGCGTGGAGGTGGAAGGCCCACGGGTCGTCAACTTCTTTTCTCGGAGAAGAAACAATGACGGTATCTGAGGAATAAGCATCGGCTAACTCTGTGCCAGCAGCCGCGGTAAGACAGAGGATGCAAGCGTTATCCGGAATGATTGGGCGTAAAGCGTCTGTAGGTGGCTTTTCAAGTCCGCCGTCAAATCCCAGGGCTCAACCCTGGACAGGCGGTGGAAACTACCAAGCTGGAGTACGGTAGGGGCAGAGGGAATTTCCGGTGGAGCGGTGAAATGCATTGAGATCGGAAAGAACACCAACGGCGAAAGCACTCTGCTGGGCCGACACTGACACTGAGAGACGAAAGCTAGGGGAGCAAATGGG

>OTU98GTAGGGAATCTTCCACAATGGACGAAAGTCTGATGGAGCAACGCCGCGTGAGTGATGAAGGCTTTAGGGTCGTAAAACTCTGTTGTTGGAGAAGAACGTGTGTGAGAGTAACTGCTCATGCAGTGACGGTATCCAACCAGAAAGCCACGGCTAACTACGTGCCAGCAGCCGCGGTAATACGTAGGTGGCAAGCGTTATCCGGATTTATTGGGCGTAAAGCGAGCGCAGGCGGTTTTTTAAGTCTAATGTGAAAGCCTTCGGCTTAACCGAAGAAGTGCATTGGAAACTGGGAAACTTGAGTGCAGAAGAGGACAGTGGAACTCCATGTGTAGCGGTGAAATGCGTAGATATATGGAAGAACACCAGTGGCGAAGGCGGCTGTCTGGTCTGTAACTGACGCTGAGGCTCGAAAGCATGGGTAGCGAACAGG

>OTU99GTGGGGAATATTGCACAATGGGGGAAACCCTGATGCAGCGACGCCGCGTGAAGGAAGAAGTATCTCGGTATGTAAACTTCTATCAGCAGGGAAGAAAATGACGGTACCTGACTAAGAAGCCCCGGCTAACTACGTGCCAGCAGCCGCGGTAATACGTAGGGGGCAAGCGTTATCCGGATTTACTGGGTGTAAAGGGAGCGTAGACGGAAGAGCAAGTCTGATGTGAAAGGCTGGGGCTTAACCCCAGGACTGCATTGGAAACTGTTGTTCTAGAGTGCCGGAGAGGTAAGCGGAATTCCTAGTGTAGCGGTGAAATGCGTAGATATTAGGAGGAACACCAGTGGCGAAGGCGGCTTACTGGACGGTAACTGACGTTGAGGCTCGAAAGCGTGGGGAGCAAACAGG

>OTU100GTGGGGAATCTTGCGCAATGGGCGAAAGCCTGACGCAGCCATGCCGCGTGAATGATGAAGGTCTTAGGATTGTAAAATTCTTTCACCGGGGACGATAATGACGGTACCCGGAGAAGAAGCCCCGGCTAACTTCGTGCCAGCAGCCGCGGTAATACGAAGGGGGCTAGCGTTGCTCGGAATTACTGGGCGTAAAGGGCGCGTAGGCGGATCGTTAAGTCAGAGGTGAAATCCCAGGGCTCAACCCTGGAACTGCCTTTGATACTGGCGATCTTGAGTATGAGAGAGGTATGTGGAACTCCGAGTGTAGAGGTGAAATTCGTAGATATTCGGAAGAACACCAGTGGCGAAGGCGACATACTGGCTCATTACTGACGCTGAGGCGCGAAAGCGTGGGGAGCAAACAGG

>OTU101GTGAGGAATTTTGCGCAATGGAGGCAACTCTGACGCAGCCACGCCGCGTGCAGGAGGACGCCCCTACGGGGTGTAAACTGCTTTTCTGCGAGAAGAAGGCCTTGCTTTGGGCAGGGTGTGACGGTATCGCAGGAATAAGCACCGGCTAACTCCGTGCCAGCAGCCGCGGTAATACGGAGGGTGCAAGCGTTGTCCGGAATTATTGGGTGTAAAGGGTGTGCAGGCGGGGCGGTAAGTCAGGGGTGAAAGTCTACGGCTCAACCGTAGGATGGCCTTTGATACTGTCGTCCTTGAGTCCCGGAGAGGCCATTAGAATTCGTGGTGTAGCGGTGAAATGCGTAGATATCACGAGGAATACCGGAAGCGTAGGCGGATGGCTGGACGGCGACTGACGCTCAGGCACGAAAGCGTGGGGAGCGAACAGG

>OTU102GTGGGGAATATTGCACAATGGGGGAAACCCTGATGCAGCCATGCCGCGTGTGTGAAGAAGGCCTTCGGGTTGTAAAGCACTTTCAGTAGGGAGGAAGGTAGTGTAGTTAACACCTGCATTATTTGACGTTACCTACAGAAGAAGCACCGGCTAACTCCGTGCCAGCAGCCGCGGTAATACGGAGGGTGCGAGCGTTAATCGGAATTACTGGGCGTAAAGCGCATGCAGGCGGCCTGTTAAGTCAGATGTGAAAGCCCGGGGCTTAACCTCGGAATTGCATTTGAAACTGGCAGGCTAGAGTCTTGTAGAGGGGGGTAGAATTTCAGGTGTAGCGGTGAAATGCGTAGAGATCTGAAGGAATACCAGTGGCGAAGGCGGCCCCCTGGACAAAGACTGACGCTCAGATGCGAAAGCGTGGGGAGCAAACAGG

>OTU103GTGGGGAATATTGGACAATGGGCGAAAGCCTGATCCAGCAATGCCGCGTGAGTGATGAAGGCCTTAGGGTTGTAAAGCTCTTTTACCAGGGATGATAATGACAGTACCTGGAGAATAAGCTCCGGCTAACTCCGTGCCAGCAGCCGCGGTAATACGGAGGGAGCTAGCGTTGTTCGGAATTACTGGGCGTAAAGCGCACGTAGGCGGCTACTCAAGTCAGAGGTGAAAGCCCGGGGCTCAACCCCGGAACTGCCTTTGAAACTAGGTAGCTAGAATCTTGGAGAGGTCAGTGGAATTCCGAGTGTAGAGGTGAAATTCGTAGATATTCGGAAGAACACCAGTGGCGAAGGCGACTGACTGGACAAGTATTGACGCTGAGGTGCGAAAGCGTGGGGAGCAAACAGG

>OTU104GTGGGGAATCTTAGACAATGGGCGCAAGCCTGATCTAGCCATGCCGCGTGAGTGATGAAGGCCTTAGGGTCGTAAAGCTCTTTCGCTGGGGAAGATAATGACGGTACCCAGTAAAGAAACCCCGGCTAACTCCGTGCCAGCAGCCGCGGTAATACGGAGGGGGTTAGCGTTGTTCGGAATTACTGGGCGTAAAGCGCGCGTAGGCGGATTAGAAAGTTGGGGGTGAAATCCCGGGGCTCAACCCCGGAACTGCCTCCAAAACTACTAGTCTAGAGGTCGAGAGAGGTGAGTGGAATTCCGAGTGTAGAGGTGAAATTCGTAGATATTCGGAGGAACACCAGTGGCGAAGGCGGCTCACTGGCTCGATACTGACGCTGAGGTGCGAAAGCGTGGGGAGCAAACAGG

>OTU105GTGGGGAATATTGGACAATGGGCGCAAGCCTGATCCAGCCATGCCGCGTGAGTGATGAAGGCCCTAGGGTTGTAAAGCTCTTTTGTGCGGGAAGATAATGACTGTACCGCAAGAATAAGCCCCGGCTAACTTCGTGCCAGCAGCCGCGGTAATACGAAGGGGGCTAGCGTTGCTCGGAATCACTGGGCGTAAAGCGCACGTAGGCGGACTCTTAAGTCGGTGGTGAAATCCTGGAGCTCAACTCCAGAACTGCCTTCGATACTGGGAGTCTCGAGTTCGGGAGAGGTGAGTGGAACTGCGAGTGTAGAGGTGAAATTCGTAGATATTCGCAAGAACACCAGTGGCGAAGGCGGCTCACTGGCCCGATACTGACGCTGAGGTGCGAAAGCGTGGGGAGCAAACAGG

>OTU106GTAGGGAATCTTCCGCAATGGACGAAAGTCTGACGGAGCAACGCCGCGTGAGTGAAGAAGGTTTTCGGATCGTAAAACTCTGTTGTCAGAGAAGAACAAGTCGGAGAGTAACTGCTCCGGCCTTGACGGTATCTGACCAGAAAGCCACGGCTAACTACGTGCCAGCAGCCGCGGTAATACGTAGGTGGCAAGCGTTGTCCGGATTTATTGGGCGTAAAGCGAGCGCAGGCGGTTCCTTAAGTCTGATGTGAAAGCCCACGGCTCAACCGTGGAAGGTCATTGGAAACTGGGGAACTTGAGTGCAGAAGAGGAGAGTGGAATTCCATGTGTAGCGGTGAAATGCGTAGATATATGGAGGAACACCAGTGGCGAAGGCGACTCTCTGGTCTGTAACTGACGCTGAGGCTCGAAAGCGTGGGGAGCAAACAGG

>OTU107GTGGGGGATATTGCACAATGGGGGAAACCCTGATGCAGCGACGCCGCGTGAGTGAAGAAGTATTTCGGTATGTAAAGCTCTATCAGCAGGGACGAAAATGACGGTACCTGAGTAAGAAGCCCCGGCTAACTACGTGCCAGCAGCCGCGGTAATACGTAGGGGGCAAGCGTTATCCGGATTTACTGGGTGTAAAGGGAGCGTAGACGGTTAAGCAAGTCTGGAGTGAAAGGCGGGGGCTCAACCCCCGGACTGCTCTGGAAACTGTATAACTAGAGTGCAGGAGGGGTAAGTGGAATTCCTAGTGTAGCGGTGAAATGCGTAGATATTAGGAGGAACACCAGTGGCGAAGGCGGCTTACTGGACTGTAACTGACGTTGAGGCTCGAAAGCGTGGGGAGCAAACAGG

>OTU108GTGGGGAATATTGCACAATGGGCGCAAGCCTGATGCAGCGACGCCGCGTGAGGGATGACGGCCTTCGGGTTGTAAACCTCTGTTAGCAGGGAAGAAGAGAAATTGACGGTACCTGCAGAGAAAGCGCCGGCTAACTACGTGCCAGCAGCCGCGGTAATACGTAGGGCGCGAGCGTTGTCCGGAATTATTGGGCGTAAAGAGCTTGTAGGCGGTTTGTCGCGTCTGCTGTGAAAGGCCGGGGCTTAACCCCGTGTATTGCAGTGGGTACGGGCAGACTAGAGTGCAGTAGGGGAGACTGGAATTCCTGGTGTAGCGGTGGAATGCGCAGATATCAGGAGGAACACCGATGGCGAAGGCAGGTCTCTGGGCTGTAACTGACGCTGAGAAGCGAAAGCATGGGGAGCGAACAGG

>OTU109GTGGGGAATCTTGGACAATGGGCGCAAGCCTGATCCAGCAATATTGCGTGAGTGAAGAAGGGCAATTCCGCTCGTAAAGCTCTTTCGTCGAGTGCGCGATCATGACATGACTCGAGGAAGAAGCCCCGGCTAACTCCGTGCCAGCAGCCGCGGTAAAACGGGGGGGGCAAGTGTTCTTCGGAATGACTAGGCGTAAAGGGCACGTAGGCGGTTCATCGGGTTGAAAGTGAAAGTCGTCAAAAAGTGGCGGAATGCTTTCGAAACCACTGAACTTGAGTGAGACAGAGGAGAGTGGAATTTCGTGTGGAGGGGTGAAATCTACGGATCTACGAAGGAACGCCAAAAGCGAAGGCAGCTCTCTGGGTCTCTACCGACGCTGGTGTGCGAAAGCATGGGGAGCGAACGGG

>OTU110GTGGGGAATATTGGACAATGGGCGAAAGCCTGATCCAGCAATGCCGCGTGTGTGAAGAAGGTCTTCGGATTGTAAAGCACTTTCGACGGGGACGATGATGACGGTACCCGTAGAAGAAGCCCCGGCTAACTTCGTGCCAGCAGCCGCGGTAATACGAAGGGGGCTAGCGTTGCTCGGAATGACTGGGCGTAAAGGGCGCGTAGGCGGTTTATGCAGTCAGATGTGAAATCCCCGGGCTTAACCTGGGAACTGCATTTGAGACGCATAGACTAGAGGTCGAGAGAGGGTTGTGGAATTCCCAGTGTAGAGGTGAAATTCGTAGATATTGGGAAGAACACCGGTGGCGAAGGCGGCAACCTGGCTCGATACTGACGCTGAGGCGCGAAAGCGTGGGGAGCAAACAGG

>OTU111GTGGGGAATATTGCACAATGGGCGCAAGCCTGATGCAGCCATGCCGCGTGTATGAAGAAGGCCTTAGGGTTGTAAAGTACTTTCAGCGGGGAGGAAGGTGATAAGGTTAATACCCTTATCAATTGACGTTACCCGCAGAAGAAGCACCGGCTAACTCCGTGCCAGCAGCCGCGGTAATACGGAGGGTGCAAGCGTTAATCGGAATTACTGGGCGTAAAGCGCACGCAGGCGGTCAATTAAGTCAGATGTGAAAGCCCCGAGCTTAACTTGGGAATTGCATCTGAAACTGGTTGGCTAGAGTCTTGTAGAGGGGGGTAGAATTCCATGTGTAGCGGTGAAATGCGTAGAGATGTGGAGGAATACCGGTGGCGAAGGCGGCCCCCTGGACAAAGACTGACGCTCAGGTGCGAAAGCGTGGGGAGCAAACAGG

>OTU112GTGGGGAATATTGGACAATGGGCGAAAGCCTGATCCAGCCATGCCGCGTGTGTGAAGAAGGTCTTCGGATTGTAAAGCACTTTAAGTTGGGAGGAAGGGCAGTAACTTAATACGTTGCTGTTTTGACGTTACCGACAGAATAAGCACCGGCTAACTCTGTGCCAGCAGCCGCGGTAATACAGAGGGTGCAAGCGTTAATCGGAATTACTGGGCGTAAAGCGCGCGTAGGTGGTTTGTTAAGTTGGATGTGAAAGCCCCGGGCTCAACCTGGGAACTGCATTCAAAACTGACAAGCTAGAGTATGGTAGAGGGTGGTGGAATTTCCTGTGTAGCGGTGAAATGCGTAGATATAGGAAGGAACACCAGTGGCGAAGGCGACCACCTGGACTGATACTGACACTGAGGTGCGAAAGCGTGGGGAGCAAACAGG

>OTU113GTGAGGAATATTGGTCAATGGGTGCAAGCCTGAACCAGCCATCCCGCGTGAAGGACGACTGCCCTATGGGTTGTAAACTTCTTTTGTATAGGGATAAACCTACCCTCGTGAGGGTAGCTGAAGGTACTATACGAATAAGCACCGGCTAACTCCGTGCCAGCAGCCGCGGTAATACGGAGGGTGCAAGCGTTATCCGGATTTATTGGGTTTAAAGGGTCCGTAGGCGGACTTATAAGTCAGTGGTGAAAGCCTGTCGCTTAACGATAGAACTGCCATTGATACTGTAAGTCTTGAGTATATTTGAGGTAGCTGGAATAAGTAGTGTAGCGGTGAAATGCATAGATATTACTTAGAACACCAATTGCGAAGGCAGGTTACCAAGATATAACTGACGCTGAGGGACGAAAGCGTGGGGAGCGAACAGG

>OTU114GTGAGGAATATTGGTCAATGGGCGCAAGCCTGAACCAGCCAAGTCGCGTGAGGGAAGACGGTCCTAAGGATTGTAAACCTCTTTTGTCAGGGAGCAAAACGTCCACGTGTGGATAATCGAGAGTACCTGAAGAAAAAGCATCGGCTAACTCCGTGCCAGCAGCCGCGGTAATACGGAGGATGCGAGCGTTATCCGGATTTATTGGGTTTAAAGGGTGCGTAGGCGGAAGATTAAGTCAGCGGTAAAATCGAGTGGCTCAACCACTTCCGGCCGTTGAAACTGGTTTTCTTGAGTGAGCGAGAAGTATGCGGAATGCGTGGTGTAGCGGTGAAATGCATAGATATCACGCAGAACTCCGATTGCGAAGGCAGCATACCGGCGCTCAACTGACGCTGAAGCACGAAAGCGTGGGGATCGAACAGG

>OTU115GTGGGGAATATTGCGCAATGGGGGAAACCCTGACGCAGCAACGCCGCGTGCGGGATGAAGGCTTTCGGGTTGTAAACCGCTTTCAGCAGGGAAGATAATGACGGTACCTGCAGAAGAAGCCCCGGCTAACTACGTGCCAGCAGCCGCGGTAATACGTAGGGGGCAAGCGTTATCCGGATTCATTGGGCGTAAAGCGCGCGCAGGCGGGTCGCTAAGCGGGACCTCTAATCTTGGGGCTCAACCTCAAGCCGGGTCCCGAACTGACGGCCTCGAGTGCGGTAGGGGTAAGCGGAATTCCCGGTGTAGCGGTGGAATGCGCAGATATCGGGAAGAACACCGATGGCGAAGGCAGCTTACTGGGCCGACACTGACGCTGAGGCGCGAAAGCTAGGGGAGCGAACAGG

>OTU116GTAGGGAATCTTCCACAATGGACGAAAGTCTGATGGAGCAACGCCGCGTGAGCGATGAAGGCCTTCGGGTCGTAAAGCTCTGTTGTTAGGGAAGAACAAGTGCGAGAGTAACTGCTCGCACCTTGACGGTACCTAACCAGAAAGCCACGGCTAACTACGTGCCAGCAGCCGCGGTAATACGTAGGTGGCAAGCGTTGTCCGGAATTATTGGGCGTAAAGCGCGCGCAGGCGGTTTCTTAAGTCTGATGTGAAAGCCCACGGCTCAACCGTGGAGGGTCATTGGAAACTGGGAGACTTGAGTGCAGAAGAGGAGAGTGGAATTCCACGTGTAGCGGTGAAATGCGTAGAGATGTGGAGGAACACCAGTGGCGAAGGCGACTCTCTGGTCTGTAACTGACGCTGAGGCGCGAAAGCGTGGGGAGCAAACAGG

>OTU117GTGAGGAATATTGGTCAATGGGCGAGAGCCTGAACCAGCCAAGTAGCGTGAAGGATGACTGCCCTATGGGTTGTAAACTTCTTTTATATGGGAATAAAGTGCAGTATGTATACTGTTTTGTATGTACCATACGAATAAGGATCGGCTAACTCCGTGCCAGCAGCCGCGGTAATACGGAGGATCCGAGCGTTATCCGGATTTATTGGGTTTAAAGGGAGCGTAGGCGGATTATTAAGTCAGTTGTGAAAGTTTGCGGCTCAACCGTAAAATTGCAGTTGATACTGGTAGTCTTGAGTGCAGCAGAGGTAGGCGGAATTCGTGGTGTAGCGGTGAAATGCTTAGATATCACGAAGAACTCCGATTGCGAAGGCAGCTTACTGGACTGTAACTGACGCTGATGCTCGAAAGTGTGGGTATCAAACAGG

>OTU118GTAGGGAATCTTCGGCAATGGGCGAAAGCCTGACCGAGCAACGCCGCGTGAATGAAGAAGGCCTTCGGGTTGTAAAATTCTGTTATAAGGGAAGAAAGGTGATAGGAGGAAATGACTATCAATTGACGGTACCTTATGAGAAAGCCACGGCTAACTACGTGCCAGCAGCCGCGGTAATACGTAGGTGGCAAGCGTTATCCGGAATTATTGGGCGTAAAGAGCGCGCAGGTGGTTAATTAAGTCTGATGTGAAAGCCCACGGCTTAACCGTGGAGGGTCATTGGAAACTGGTTGACTTGAGTGCAGAAGAGGGAAGTGGAATTCCATGTGTAGCGGTGAAATGCGTAGAGATATGGAGGAACACCAGTGGCGAAGGCGGCTTCCTGGTCTGCAACTGACACTGAGGCGCGAAAGCGTGGGGAGCAAACAGG

>OTU119GTGGGGAATATTGCACAATGGGGGGAACCCTGATGCAGCGACGCCGCGTGAGTGAAGAAGTATTTCGGTATGTAAAGCTCTATCAGCAGGGAAGATAATGACGGTACCTGACTAAGAAGCCCCGGCTAACTACGTGCCAGCAGCCGCGGTAATACGTAGGGGGCAAGCGTTATCCGGATTTACTGGGTGTAAAGGGTGCGTAGGTGGCAGGGCAAGTCAGATGTGAAAGCCCGGGGCCCAACCCCGGTACTGCATTTGAAACTGTCCGGCTGGAGTGCAGGAGAGGTAAGCGGAATTCCTAGTGTAGCGGTGAAATGCGTAGATATTAGGAGGAACACCAGTGGCGAAGGCGGCTTACTGGACTGTAACTGACACTGAGGCACGAAAGCGTGGGGAGCAAACAGG

>OTU120GTGGGGGATATTGGACAATGGGGGGAACCCTGATCCAGCGACGCCGCGTGAGTGAAGAAGTATTTCGGTATGTAAAGCTCTATCAGCAGGGAAGAAGAAATGACGGTACCTGAGTAAGAAGCCCCGGCTAACTACGTGCCAGCAGCCGCGGTAATACGTAGGGGGCAAGCGTTATCCGGAATTACTGGGTGTAAAGGGAGCGTAGACGGTGTAGCAAGTCTGGAGTGAAAGGCTGGGGCCCAACCCCGGGACTGCTCTGGAAACTGTTAAACTGGAGTGCAGGAGAGGCAGGCGGAATTCCTAGTGTAGCGGTGAAATGCGTAGATATTAGGAGGAACACCAGTGGCGAAGGCGGCCTGCTGGACTGTAACTGACGTTGAGGCTCGAAAGCGTGGGGAGCAAACAGG

>OTU121GTGAGGAATATTGGTCAATGGGCGCGAGCCTGAACCAGCCAAGTCGCGTGAGGGAAGACGGCCCTACGGGTTGTAAACCTCTTTTGTCGGGGAGCAAGGACTGCCACGAGTGGCAGGGCGAGAGTACCCGAAGAAAAAGCATCGGCTAACTCCGTGCCAGCAGCCGCGGTAATACGGAGGATGCGAGCGTTATCCGGATTTATTGGGTTTAAAGGGTGCGTAGGCGGAAGTTCAAGTCAGCGGTAAAATCGAGGGGCTCAACCCCTTTAAGCCGTTGAAACTGGGTTTCTTGAGTGGGCGAGAAGTGTGCGGAATGTGTGGTGTAGCGGTGAAATGCATAGATATCACACAGAACTCCGATTGCGAAGGCAGCACACCGGCGCCCAACTGACGCTGAGGCACGAAAGCGTGGGGATCAAACAGG

>OTU122GTGGGGAATATTGCACAATGGGCGAAAGCCTGATGCAGCGACGCCGCGTGGGGGATGAAGGCCTTCGGGTTGTAAACCCCTTTCAGTAGGGAAGAAGCGAAAGTGACGGTACCTGCAGAAGAAGCGCCGGCTAACTACGTGCCAGCAGCCGCGGTAATACGTAGGGCGCAAGCGTTGTCCGGAATTATTGGGCGTAAAGAGCTCGTAGGCGGTTTGTCGCGTCTGCCGTGAAAACCCAACGCTTAACGTTGGGCTTGCGGTGGGTACGGGCAGGCTAGAGTGCGGTAGGGGTGACTGGAATTCCTGGTGTAGCGGTGGAATGCGCAGATATCAGGAGGAACACCGATGGCGAAGGCAGGTCACTGGGCCGCAACTGACGCTGAGGAGCGAAAGCATGGGGAGCGAACAGG

>OTU123GTGGGGAATATTGCACAATGGGCGAAAGCCTGATGCAGCGACGCCGCGTGAGGGATGACGGCCTTCGGGTTGTAAACCTCTTTCAGCAGGGAAGAAGCGCAAGTGACGGTACCTGCAGAAGAAGCACCGGCTAACTACGTGCCAGCAGCCGCGGTAATACGTAGGGTGCGAGCGTTGTCCGGAATTATTGGGCGTAAAGAGCTCGTAGGCGGCCTGTCGCGTCGGATGTGAAAGCCCGGGGCTTAACCCCGGGTCTGCATTCGATACGGGCAGGCTAGAGTGTGGTAGGGGAGATCGGAATTCCTGGTGTAGCGGTGAAATGCGCAGATATCAGGAGGAACACCGGTGGCGAAGGCGGATCTCTGGGCCATTACTGACGCTGAGGAGCGAAAGCGTGGGGAGCGAACAGG

>OTU124GTCGGGAATATTGCGCAATGGAGGAAACTCTGACGCAGTGACGCCGCGTATAGGAAGAAGGTTTTCGGATTGTAAACTATTGTCGTTAGGGAAGAAAAAAGACAGTACCTAAGGAGGAAGCCCCGGCTAACTATGTGCCAGCAGCCGCGGTAATACATAGGGGGCAAGCGTTATCCGGAATTATTGGGTGTAAAGGGTGCGTAGACGGGTGTTTAAGTTGGTTGTGAAATCCCTCGGCTCAACTGAGGAACTGCAACCAAAACTGGATATCTTGAGTGTCGGAGAGGAAAGTGGAATTCCTAGTGTAGCGGTGAAATGCGTAGATATTAGGAGGAACACCAGTGGCGAAGGCGACTTTCTGGACGATAACTGACGTTGAGGCACGAAAGTGTGGGGAGCAAACAGG

>OTU125GTGGGGAATATTGGACAATGGGCGCAAGCCTGATCCAGCCATGCCGCGTGAGTGATGAAGGCCCTAGGGTTGTAAAGCTCTTTTGTGCGGGAAGATAATGACGGTACCGCAAGAATAAGCCCCGGCTAACTTCGTGCCAGCAGCCGCGGTAATACGAAGGGGGCTAGCGTTGCTCGGAATCACTGGGCGTAAAGGGTGCGTAGGCGGGTCTTTAAGTCAGGGGTGAAATCCTGGAGCTCAACTCCAGAACTGCCTTTGATACTGAGGATCTTGAGTTCGGGAGAGGTGAGTGGAACTGCGAGTGTAGAGGTGAAATTCGTAGATATTCGCAAGAACACCAGTGGCGAAGGCGGCTCACTGGCCCGATACTGACGCTGAGGCACGAAAGCGTGGGGAGCAAACAGG

>OTU126GTGGGGAATATTGCACAATGGGCGCAAGCCTGATGCAGCGACGCCGCGTGCGGGATGGAGGCCTTCGGGTTGTAAACCGCTTTTGTTCAAGGGCAAGGCAGGTTTTTTGGCCTGTTGAGTGGATTGTTCGAATAAGCACCGGCTAACTACGTGCCAGCAGCCGCGGTAATACGTAGGGTGCAAGCGTTATCCGGATTTATTGGGCGTAAAGGGCTCGTAGGCGGTTCGTCGCGTCCGGTGTGAAAGTCCATCGCTTAACGGTGGATCCGCGCCGGGTACGGGCGGGCTTGAGTGCGGTAGGGGAGACTGGAATTCCCGGTGTAACGGTGGAATGTGTAGATATCGGGAAGAACACCAATGGCGAAGGCAGGTCTCTGGGCCGTTACTGACGCTGAGGAGCGAAAGCGTGGGGAGCGAACAGG

>OTU127GTGGGGAATATTGCACAATGGGGGAAACCCTGATGCAGCGACGCCGCGTGAGCGATGAAGTATTTCGGTATGTAAAGCTCTATCAGCAGGGAAGAAAATGACGGTACCTGACTAAGAAGCCCCGGCTAACTACGTGCCAGCAGCCGCGGTAATACGTAGGGGGCAAGCGTTATCCGGATTTACTGGGTGTAAAGGGAGCGTAGACGGCATGGCAAGCCAGATGTGAAAGCCCGGGGCTCAACCCCGGGACTGCATTTGGAACTGTCAGGCTAGAGTGTCGGAGAGGAAAGCGGAATTCCTAGTGTAGCGGTGAAATGCGTAGATATTAGGAGGAACACCAGTGGCGAAGGCGGCTTTCTGGACGATGACTGACGTTGAGGCTCGAAAGCGTGGGGAGCAAACAGG

>OTU128GTCGAGAATCATTCACAATGGGGGCAACCCTGATGGTGCAACGCCGCGTGGAGGATGAAGGCCCTAGGGTCGTAAACTCCTGTCATGTGAGAGTAAGGCATAATCGTTAATAGCTTTTATGTTTGATAGTATCACAAGAGGAAGGGACGGCTAACTTCGTGCCAGCAGCCGCGGTAATACGAAGGTCCCGAGCGTTATTCGGAATCACTGGGCGTAAAGGGAGCGTAGGCTGCGCGGTAAGTCAGATGTGAAATCTCAGGGCTCAACCCTGAAACTGCATCCGATACTGCCGCGCTAGAGTAATGGAGAGGTAAGTGGAATTCTCAGTGTAGCAGTGAAATGCGTAGATATTGAGAGGAAGACCAATGGCGAAGGCAGCTTACTGGACATTTACTGACGCTGAGGCTCGAAGGCTAGGGTAGCGAAAGGG

>OTU129GTAGGGAATCTTCCACAATGGACGAAAGTCTGATGGAGCAATGCCGCGTGAGTGAAGAAGGGTTTCGGCTCGTAAAACTCTGTTGTTAAAGAAGAACACCTTTGAGAGTAACTGTTCAAGGGTTGACGGTATTTAACCAGAAAGCCACGGCTAACTACGTGCCAGCAGCCGCGGTAATACGTAGGTGGCAAGCGTTGTCCGGATTTATTGGGCGTAAAGCGAGCGCAGGCGGTTTTTTAAGTCTGATGTGAAAGCCTTCGGCTTAACCGGAGAAGTGCATCGGAAACTGGGAGACTTGAGTGCAGAAGAGGACAGTGGAACTCCATGTGTAGCGGTGGAATGCGTAGATATATGGAAGAACACCAGTGGCGAAGGCGGCTGTCTAGTCTGTAACTGACGCTGAGGCTCGAAAGCATGGGTAGCAAACAGG

>OTU130GTGGGGAATATTGGACAATGGGCGCAAGCCTGATCCAGCCATGCCGCGTGTGTGAAGAAGGCCTTCGGGTTGTAAAGCACTTTCAGTAGGGAGGAAGCGTTGTGTGTTAATAGTACACAGCGTTGACGTTACCTACAGAAGAAGCACCGGCTAACTCTGTGCCAGCAGCCGCGGTAATACAGAGGGTGCAAGCGTTAATCGGAATTACTGGGCGTAAAGCGCACGTAGGCGGTTTTTTAAGTCAGATGTGAAAGCCCCGGGCTCAACCTGGGAATTGCATTTGAAACTGGAAAACTAGAGTGTGTGAGAGGGGGGTAGAATTCCAAGTGTAGCGGTGAAATGCGTAGAGATTTGGAGGAATACCAGTGGCGAAGGCGGCCCCCTGGCACAACACTGACGCTCAGGTGCGAAAGCGTGGGGAGCAAACAGG

>OTU131GTGGGGAATATTGCGCAATGGAGGCAACTCTGACGCAGCAATGCCGCGTGGGTGAAGAAGGGATTCGTCCTGTAAAGCCCTGTTGTCGGGGAAGAGTTAAGACGGTACCCGGCGTATGAGCCCCAGCCAACCACGTGCCAGCAGCTGCGGTAACACGTGGGGGGCGAGCGTTATCCGGATTTATTGGGCGTAAAGGGCACGCAGGCGGCAGCGAAGGTGCGGTGTTAAAACCCGGGGCCCAACCCCGGGTTGCACGGCAAACCGTGCTGCTGGAGTGAAGCAGAGGCAGGCAGAATTCCTGGTGTAGCGGTGAAATGCGCAGAGATCAGGAGGAATACCGGTGGCGGAGGCGGCCTGCTGGGCTTATACTGACGCTGAGGTGCGAAAGCGTGGGGAGCGAACAGG

>OTU132GTAGGGAATATTGCTCAATGGGGGAAACCCTGAAGCAGCAACGCCGCGTGGAGGATGAAGGTTTTCGGATTGTAAACTCCTTTTGTTAGAGAAGATAATGACGGTATCTAACGAATAAGCACCGGCTAACTCCGTGCCAGCAGCCGCGGTAATACGGAGGGTGCAAGCGTTACTCGGAATCACTGGGCGTAAAGAGCGCGTAGGCGGGATAGCAAGTCAGATGTGAAATCCTATGGCTTAACCATAGAACTGCATTTGAAACTGTTATTCTAGAGTATGGGAGAGGTAGGTGGAATTCTTGGTGTAGGGGTAAAATCCGTAGAGATCAAGAGGAATACTCATTGCGAAGGCGACCTGCTAGAACATAACTGACGCTGATGCGCGAAAGCGTGGGGAGCAAACAGG

>OTU133GTGGGGAATATTGGACAATGGGCGAAAGCCTGATCCAGCAATGCCGCGTGAGTGATGAAGGCCTTAGGGTTGTAAAGCTCTTTTACCCGGGATGATAATGACAGTACCGGGAGAATAAGCTCCGGCTAACTCCGTGCCAGCAGCCGCGGTAATACGGAGGGAGCTAGCGTTATTCGGAATTACTGGGCGTAAAGCGCACGTAGGCGGCTTTGTAAGTTAGAGGTGAAAGCCTGGAGCTCAACTCCAGAATTGCCTTTAAGACTGCATCGCTTGAATCCAGGAGAGGTGAGTGGAATTCCGAGTGTAGAGGTGAAATTCGTAGATATTCGGAAGAACACCAGTGGCGAAGGCGGCTCACTGGACTGGTATTGACGCTGAGGTGCGAAAGCGTGGGGAGCAAACAGG

>OTU134GTGGGGAATATTGCGCAATGGGGGAAACCCTGACGCAGCAACGCCGCGTGAGCGATGAAGGTTTTCGGATCGTAAAGCTCTGTCATTTGGGACGATAATGACGGTACCAAATGAGGAAGCCACGGCTAACTACGTGCCAGCAGCCGCGGTAATACGTAGGTGGCGAGCGTTGTCCGGATTTACTGGGTGTAAAGGGTGCGTAGGCGGATACTTAAGTCAGATGTGAAATACCCGGGCTCAACTTGGGTGCTGCATTTGAAACTGGATATCTAGAGTACAGGAGAGGAAAGCGGAATTCCTAGTGTAGCGGTGAAATGCGTAGAGATTAGGAAGAACACCGGTGGCGAAGGCGGCTTTCTGGACTGTAACTGACGCTGAGGCACGAAAGCGTGGGGAGCAAACAGG

>OTU135GTGGGGAATCTTCCGCAATGGGCGCAAGCCTGACGGAGTGACGCCGCGTGGGGGACGAAGGCCTTCGGGTCGTAAACCCCTGTCAGAGGGAAAGAAGGGTCATGCGGTGAACAGCCGCGTGGCTTGACGGTACCTTCAGAGGAAGCCCCGGCCAACTCTGTGCCAGCAGCCGCGGTAAGACAGAGGGGGCAAGCGTTGCTCGGAATCACTGGGCGTAAAGGGCGCGTAGGCGGGACGGCAAGTCGGTCGTGAAAGCCCTCGGCTCAACCGAGGAGAGTCGGCCGAGACTGTCGTTCTTGAGGGATGCAGAGGAGACTGGAATTCCCGGTGTAGCGGTGAAATGTGTAGAGATCGGGAGGAACACCGGTGGCGAAGGCGGGTCTCTGGGCATCTCCTGACGCTGAGGCGCGAAAGCGTGGGGAGCAAACGGG

>OTU136GTGAGGAATATTGGTCAATGGTCGTGAGACTGAACCAGCCAAGTAGCGTGCGGGATGAAGGCCCTCCGGGTCGTAAACCGCTTTTAGACGGGGATAAAAGGGCATACGTGTATGCCGTATTGCATGTACCGTCAGAAAAAGGACCGGCTAATTCCGTGCCAGCAGCCGCGGTAATACGGAAGGTCCGGGCGTTATCCGGATTTATTGGGTTTAAAGGGAGCGTAGATGGGTTGTTAAGTCAGTTGTGAAAGTTTGCGGCTCAACCGTAAAATTGCAATTGATACTGGCGTCCTTGAGTACAGTTGAGGTGGGCGGAATTCGTGGTGTAGCGGTGAAATGCTTAGATATCACGAAGAACTCCTATTGCGAAGGCAGCTCACTAAACTGCAACTGACATTGAGGCTCGAAAGTGTGGGTATCAAACAGG

>OTU137GTGAGGAATATTGGTCAATGGGCGCAAGCCTGAACCAGCCATCCCGCGTGCAGGAAGACGGCGCTATGCGTTGTAAACTGCTTTTCCAGGGAGAGAAACTTGCGCACGTGTTGCGCAATTGACGGTACCCTGGGAATAAGCATCGGCTAACTCCGTGCCAGCAGCCGCGGTAATACGGAGGATGCGAGCGTTATCCGGATTCATTGGGTTTAAAGGGTGCGTAGGCGGACTATTAAGTCAGTGGTGAAATCCTGCAGCTTAACTGCAGAACTGCCATTGATACTGATAGCCTTGAGTTTGGTTAAGGTAGGCGGAATGTGTAATGTAGCGGTGAAATGCTTAGATATTACACAGAACACCAATTGCGTAGGCAGCTTACTGAGCCGACACTGACGCTGAGGCACGAAAGCGTGGGGATCGAACAGG

>OTU138GTGAGGAATATTGGTCAATGGGCGCGAGCCTGAACCAGCCAAGTAGCGTGAAGGATGACTGCCCTACGGGTTGTAAACTTCTTTTATAAAGGAATAAAGTGAGGCACGTGTGCCTTTTTGTATGTACTTTATGAATAAGGATCGGCTAACTCCGTGCCAGCAGCCGCGGTAATACGGAGGATCCGAGCGTTATCCGGATTTATTGGGTTTAAAGGGAGCGTAGATGGGTTGTTAAGTCAGTTGTGAAAGTTTGCGGCTCAACCGTAAAATTGCAATTGATACTGGCAGTCTTGAGTACAGTTGAGGTAGGCGGAATTCGTGGTGTAGCGGTGAAATGCTTAGATATCACGAAGAACTCCGATTGCGAAGGCAGCTTACTAAACTGCCACTGACATTGATGCTCGAAAGTGTGGGTATCAAACAGG

>OTU139GTGGGGAATATTGCGCAATGGGCGGAAGCCTGACGCAGCGACGCCGCGTGCGGGATGGAGGCCTTCGGGTCGTGAACCGCTTTTGATTGGGAGCAAGCGAGAGTGAGTGTACCTTTCGAATAAGGACCGGCTAACTACGTGCCAGCAGCCGCGGTAATACGTAGGGTCCGAGCGCTGTCCGGAATTATTGGGCGTAAAGGGCTCGTAGGCGGTCCGTCGCGTCTGGTGTGAAAGCCCGCTGCCTAACGGCGGGTCTGCGCTGGATACGGGCGGGCTTGAGTGCGGTAGGGGAGAACGGAATTCCCGGTGTAACGGTGGAATGTGTAGATATCGGGAAGAACACCGATGGCGAAGGCAGTTCTCTGGGCCGTCACTGACGCTGAGGAGCGAAAGCGTGGGGAGCGAACCGG

>OTU140GTAAGGAATATTGCGCAATGGGCGAAAGCCTGACGCAGCGACGCCGCGTGGGGGATGAAGGTCTTCGGATTGTAAACCCCTTTCGGGAGGGAAGATGGGATGGGGTAACCCATTCGGACGGTACCTCCAGAAGCAGCCACGGCTAACTTCGTGCCAGCAGCCGCGGTAATACGAAGGTGGCAAGCGTTGTTCGGATTTACTGGGCGTACAGGGAGCGTAGGCGGTTGGATAAGCCCTCCGTGAAATCTCCAGGCTTAACCTGGAAAGTGCAGAGGGGACTGTTCAGCTAGAGGATGGGAGAGGAGCGCGGAATTCCCGGTGTAGCGGTGAAATGCGTAGAGATCGGGAGGAAGGCCGGTGGCGAAGGCGGCGCTCTGGAACATTTCTGACGCTGAGGCTCGAAAGCGTGGGGAGCAAACAGG

>OTU141GTGGGGAATATTGGGCAATGGGCGAAAGCCTGACCCAGCAACGCCGCGTGAAGGAAGAAGGTCTTCGGATTGTAAACTTCTTTTATGAGGGACGAAGGAAGTGACGGTACCTCATGAATAAGCCACGGCTAACTACGTGCCAGCAGCCGCGGTAATACGTAGGTGGCAAGCGTTATCCGGATTTACTGGGTGTAAAGGGCGCGTAGGCGGGGATGCAAGTCAGATGTGAAATCTATGGGCTTAACTCATAAACTGCATTTGAAACTGTATCTCTTGAGTGCTGGAGAGGTAGATGGAATTCCTTGTGTAGCGGTGAAATGCGTAGATATAAGGAAGAACACCAGTGGCGAAGGCGATCTACTGGACAGTAACTGACGCTGAGGCGCGAAAGCGTGGGGAGCAAACAGG

>OTU142GTGGGGAATATTGCGCAATGGGGGGAACCCTGACGCAGCAATGCCGCGTGAAGGATGAAGGTTTTCGGATTGTAAACTTCTTTTACTGAGGACGAAGAATGACGGTACTCAGAGAATAAGCCACGGCTAACTACGTGCCAGCAGCCGCGGTAATACGTAGGTGGCAAGCGTTATCCGGATTTACTGGGTGTAAAGGGCGAGTAGGCGGGATAGCAAGTCAGATGTGAAATACCGGGGCTCAACCCCGGAGCTGCATTTGAAACTGTTATTCTTGAGTGGTGGAGAGGCAAGTGGAATTCCCGGTGTAGCGGTGGAATGCGTAGATATCGGGAGGAACACCAGTGGCGAAGGCGACTTGCTGGACACTAACTGACGCTGAGGAGCGAAAGCGTGGGGAGCAAACAGG

>OTU143GTGAGGAATATTGGTCAATGGGCGTGAGCCTGAACCAGCCAAGCCGCGTGAAGGAAGAAGGTGCAGGGCATCGTAAACTTCTTTTGCCGGGGAACAATAAGCGGGACTAGTCCCGCGACGAGTGTACCCGGAGAAAAAGCATCGGCTAACTCCGTGCCAGCAGCCGCGGTAATACGGAGGATGCGAGCGTTATCCGGATTTATTGGGTTTAAAGGGTGCGTAGGCGGCATTGCAAGTCGGCGGTGAAAGACCATGGCTCAACCATGGCGAGCCGTCGAAACTGCGGGGCTTGAGAGGACGAGAGGTACGCGGAATGCGCGGTGTAGCGGTGAAATGCATAGATATCGCGCAGAACTCCGATTGCGAAGGCAGCGTACCGGTGTCTTTCTGACGCTGAGGCACGAAAGCGTGGGGATCGAACAGG

>OTU144GTGGGGAATATTGGGCAATGGGCGCAAGCCTGACCCAGCAATGCCGCGTGAAGGAAGAAGGCCCTCGGGTTGTAAACTTCTTTTATCAGGGACGAAGGATGTGACGGTACCAGATGAATAAGCTCCGGCTAACTACGTGCCAGCAGCCGCGGTAATACGTAGGGAGCAAGCGTTATCCGGATTTACTGGGTGTAAAGGGCGCGTAGGCGGGATGGCAAGTCAGATGTGAAATCCAAGGGCTCAACCCTTGAACTGCATTTGAAACTGTCGTTCTTGAGTACTGGAGAGGTTGACGGAATTCCTAGTGTAGCGGTGAAATGCGTAGATATTAGGAGGAACACCAGTGGCGAAGGCGGTCAACTGGACAGCAACTGACGCTGAGGCGCGAAAGCGTGGGGAGCAAACAGG

>OTU145GTGAGGAATATTGGGCAATGGAGGCAACTCTGACCCAGCCATGCCGCGTGAAGGATGACTGCCCTATGGGTTGTAAACTTCTTTTATATGGGAAGAAACTTATCTACGTGTAGATAACTGACGGTACCATACGAATAAGGATCGGCTAACTCCGTGCCAGCAGCCGCGGTAATACGGAGGATCCAAGCGTTATCCGGATTCATTGGGTTTAAAGGGTCCGTAGGCGGGTCTTTAAGTCAGTGGTGAAAGCCGACAGCTCAACTGTCGAACTGCCATTGATACTGGAGACCTTGAGTACAAATGAAGTAGGCGGAATGAGTCATGTAGCGGTGAAATGCATAGATATGACTCAGAACACCGATTGCGAAGGCAGCTTACTAACATGTAACTGACGCTGAGGGACGAAAGCGTGGGGAGCGAACAGG

>OTU146GTAGGGAATCTTCCGCAATGGACGAAAGTCTGACGGAGCAACGCCGCGTGAGTGAAGAAGGTCTTCGGATCGTAAAGCTCTGTTGTTAGGGAAGAACAAGTATCGGAGGAAATGCCGGTACCTTGACGGTACCTGACGAGAAAGCCACGGCTAACTACGTGCCAGCAGCCGCGGTAATACGTAGGTGGCAAGCGTTGTCCGGAATTATTGGGCGTAAAGCGCGCGCAGGCGGTCCTTTAAGTCTGATGTGAAATCTTGCGGCTCAACCGCAAGCGGTCATTGGAAACTGGGGGACTTGAGTGCAGAAGAGGAAAGCGGAATTCCACGTGTAGCGGTGAAATGCGTAGAGATGTGGAGGAACACCAGTGGCGAAGGCGGCTTTCTGGTCTGTAACTGACGCTGAGGCGCGAAAGCGTGGGGAGCAAACAGG

>OTU147GTGGGGAATCTTCCGCAATGGACGAAAGTCTGACGGAGCAACGCCGCGTGAGTGATGACGGCCTTCGGGTTGTAAAGCTCTGTTAATCGGGACGAAAGGCCTTCTTGCGAATAGTTAGAAGGATTGACGGTACCGGAATAGAAAGCCACGGCTAACTACGTGCCAGCAGCCGCGGTAATACGTAGGTGGCAAGCGTTGTCCGGAATTATTGGGCGTAAAGCGCGCGCAGGCGGATTGGTCAGTCTGTCTTAAAAGTTCGGGGCTTAACCCCGTGATGGGATGGAAACTGCCAATCTAGAGTATCGGAGAGGAAAGTGGAATTCCTAGTGTAGCGGTGAAATGCGTAGATATTAGGAAGAACACCAGTGGCGAAGGCGACTTTCTGGACGAAAACTGACGCTGAGGCGCGAAAGCCAGGGGAGCGAACGGG

>OTU148GTGGGGGATATTGCACAATGGGGGAAACCCTGATGCAGCGACGCCGCGTGGAGGAAGAAGGTTTTCGGATTGTAAACTCCTGTCGTTAGGGACGATAATGACGGTACCTAACAAGAAAGCACCGGCTAACTACGTGCCAGCAGCCGCGGTAAAACGTAGGGTGCAAGCGTTGTCCGGAATTACTGGGTGTAAAGGGAGCGCAGGCGGACCGGCAAGTTGGAAGTGAAAACTATGGGCTCAACCCATAAATTGCTTTCAAAACTGCTGGCCTTGAGTAGTGCAGAGGTAGGTGGAATTCCCGGTGTAGCGGTGGAATGCGTAGATATCGGGAGGAACACCAGTGGCGAAGGCGACCTACTGGGCACCAACTGACGCTGAGGCTCGAAAGCATGGGTAGCAAACAGG

>OTU149GTGGGGAATCTTGCGCAATGGGCGAAAGCCTGACGCAGCCATGCCGCGTGAATGATGAAGGTCTTAGGATTGTAAAATTCTTTCACCGGGGACGATAATGACGGTACCCGGAGAAGAAGCCCCGGCTAACTTCGTGCCAGCAGCCGCGGTAATACGAAGGGGGCTAGCGTTGCTCGGAATTACTGGGCGTAAAGGGAGCGTAGGCGGACTGTTTAGTCAGAGGTGAAAGCCCAGGGCTCAACCTTGGAATTGCCTTTGATACTGGCAGTCTTGAGTACGGAAGAGGTATGTGGAACTCCGAGTGTAGAGGTGAAATTCGTAGATATTCGGAAGAACACCAGTGGCGAAGGCGACATACTGGTCCGTTACTGACGCTGAGGCTCGAAAGCGTGGGGAGCAAACAGG

>OTU150GTGGGGAATATTGGGCAATGGGCGCAAGCCTGACCCAGCAACGCCGCGTGAAGGAAGAAGGCTTTCGGGTTGTAAACTTCTTTTGTCGGGGACGAAACAAATGACGGTACCTGACGAATAAGCCACGGCTAACTACGTGCCAGCAGCCGCGGTAATACGTAGGTGGCAAGCGTTATCCGGATTTACTGGGTGTAAAGGGCGTGTAGGCGGGATTGCAAGTCAGATGTGAAAACTGGGGGCTCAACCTCCAGCCTGCATTTGAAACTGTAGTTCTTGAGTGCTGGAGAGGCAATCGGAATTCCGTGTGTAGCGGTGAAATGCGTAGATATACGGAGGAACACCAGTGGCGAAGGCGGATTGCTGGACAGTAACTGACGCTGAGGCGCGAAAGCGTGGGGAGCAAACAGG

>OTU151GTGGGGAATATTGGGCAATGGGGGAAACCCTGACCCAGCAACGCCGCGTGAAGGAAGAAGGCCTTCGGGTTGTAAACTTCTTTTAGCAGGGACGAAGGACGTGACGGTACCTGCAGAAAAAGCAACGGCTAACTACGTGCCAGCAGCCGCGGTAATACGTAGGTTGCGAGCGTTGTCCGGAATTACTGGGTGTAAAGGGCGTGTAGGCGGAGCTGCAAGTCAGATGTGAAATGTCATGGCTCAACCATGGAACTGCATTTGAAACTGTGGCCCTTGAGTATCGGAGAGGCAAGCGGAATTCCTAGTGTAGCGGTGAAATGCGTAGATATTAGGAGGAACACCAGTGGCGAAGGCGGCTTGCTGGACGACAACTGACGCTGAGGCGCGAAAGCGTGGGGAGCAAACAGG

>OTU152GTGGGGAATATTGCACAATGGGCGAAAGCCTGATGCAGCGACGCCGCGTGGGGGATGACGGCCTTCGGGTTGTAAACCCCTTTCAGTAGGGAAGAAGCGAGAGTGACGGTACCTGCAGAAGAAGCGCCGGCTAACTACGTGCCAGCAGCCGCGGTAATACGTAGGGCGCAAGCGTTGTCCGGAATTATTGGGCGTAAAGAGCTTGTAGGTGGCTTGTCGCGTCTGCCGTGAAAACCCGAGGCTCAACCTCGGGCGTGCGGTGGGTACGGGCAGGCTAGAGTGTGGTAGGGGAGACTGGAACTCCTGGTGTAGCGGTGAAATGCGCAGATATCAGGAAGAACACCGATGGCGAAGGCAGGTCTCTGGGCCATTACTGACACTGAGAAGCGAAAGCATGGGTAGCGAACAGG

>OTU153GTGGGGAATATTGGACAATGGGGGGAACCCTGATCCAGCGACGCCGCGTGAGTGAAGAAGTATTTCGGTATGTAAAGCTCTATCAGCAGGGAAGAAAATGACGGTACCTGACTAAGAAGCCCCGGCTAACTACGTGCCAGCAGCCGCGGTAATACGTAGGGGGCAAGCGTTATCCGGATTTACTGGGTGTAAAGGGAGCGTAGACGGTGCAGCAAGTCTGATGTGAAAGGTCGGGGCCCAACCCCGGGACTGCATTGGAAACTGTTGAACTGGAGTACAGGAGAGGTAAGCGGAATTCCTAGTGTAGCGGTGAAATGCGTAGATATTAGGAGGAACACCGGTGGCGAAGGCGGCCTGCTGGACGACAACTGACGCTGAGGCTCGAAAGCGTGGGGAGCAAACAGG

>OTU154GTGAGGAATATTGGTCAATGGGCGGTAGCCTGAACCAGCCAAGTCGCGTGAGGGAAGACGGTCCTATGGATTGTAAACCTCTTTTGTCAGGGAGCAATTCCCGCCACGCGTGGCGGGAGGGAGAGTACCTGAAGAAAAAGCATCGGCTAACTCCGTGCCAGCAGCCGCGGTAATACGGAGGATGCGAGCGTTATCCGGATTTATTGGGTTTAAAGGGTGCGTAGGCGGAGTGCCAAGTCAGCGGTAAAAATTCGGGGCTCAACCCCGTCGTGCCGTTGAAACTGGCGCCCTTGAGTGGGCGAGAAGTATGCGGAATGCGTGGTGTAGCGGTGAAATGCATAGATATCACGCAGAACCCCGATTGCGAAGGCAGCATACCGGCGCCCTACTGACGCTGAAGCACGAAAGCGTGGGTATCGAACAGG

>OTU155GTGAGGAATATTGGTCAATGGGCGGGAGCCTGAACCAGCCAAGCCGCGTGAAGGACTAAGGCCCTATGGGTCGTAAACTTCTTTTGTCAGGGAACAAAAACGGGGACGCGTCCCCGCCTGCGTGTACCTGAAGAAAAAGCATCGGCTAACTCCGTGCCAGCAGCCGCGGTAATACGGAGGATGCGAGCGTTATCCGGATTTATTGGGTTTAAAGGGTGCGTAGGCGGTCTTGTAAGTCAGCGGTAAAAGCCCGGGGCTCAACCCCGGCGAGCCGTTGAAACTGCGGGACTAGAGACGGCGAGAAGTAGGCGGAATGCGCAGTGTAGCGGTGAAATGCTTAGATATTGCGCAGAACTCCGATTGCGAAGGCAGCCTACCGGCGCCGGACTGACGCTGAGGCACGAAAGCGTGGGTATCGAACAGG

>OTU156GTGGGGAATCTTCCGCAATGGGCGAAAGCCTGACGGAGCAATGCCGCGTGAGTGAAGAAGGGATTCGTTCCGTAAAGCTCTTTTGTTTATGACGAATGTGCTTACTGTGAATAATGGTAAGTAATGACGGTAGTAAACGAATAAGCCACGGCTAACTACGTGCCAGCAGCCGCGGTAATACGTAGGTGGCGAGCGTTGTCCGGAATTATTGGGCGTAAAGAGCATGTAGGCGGTTTTTTAAGTCCGGAGTGAAAATGCGGGGCTCAACCCCGTATGGCTCTGGATACTGGAAGACTTGAGTGCAGGAGAGGAAAGGGGAATTCCCAGTGTAGCGGTGAAATGCGTAGATATTGGGAGGAACACCAGTGGCGAAGGCGCCTTTCTGGACTGTGTCTGACGCTGAGATGCGAAAGCCAGGGTAGCGAACGGG

>OTU157GTAGGGAATCTTCCACAATGGGCGCAAGCCTGATGGAGCAACACCGCGTGAGTGAAGAAGGGTTTCGGCTCGTAAAGCTCTGTTGTTGGAGAAGAACGTGCGTGAGAGTAACTGTTCACGCAGTGACGGTATCCAACCAGAAAGTCACGGCTAACTACGTGCCAGCAGCCGCGGTAATACGTAGGTGGCAAGCGTTATCCGGATTTATTGGGCGTAAAGCGAGCGCAGGCGGTTGCTTAGGTCTGATGTGAAAGCCTTCGGCTTAACCGAAGAAGTGCATCGGAAACCGGGCGACTTGAGTGCAGAAGAGGACAGTGGAACTCCATGTGTAGCGGTGGAATGCGTAGATATATGGAAGAACACCAGTGGCGAAGGCGGCTGTCTGGTCTGCAACTGACGCTGAGGCTCGAAAGCATGGGTAGCGAACAGG

>OTU158GTGAGGAATATTGGTCAATGGGCGAAAGCCTGAACCAGCCAAGTCGCGTGAGGGATTAAGGCCCTATGGGTCGTAAACCTCTTTTGTCAGGGAGCAAGGCCTCTCACGTGTGGGAGGTTGGAGAGTACCTGAAGAAAAAGCATCGGCTAACTCCGTGCCAGCAGCCGCGGTAATACGGAGGATGCGAGCGTTATCCGGATTTATTGGGTTTAAAGGGTGCGTAGGCGGGACCTTAAGTCAGCGGTAAAATTGCGGTGCTCAACGCCGTAGAGCCGTTGAAACTGGGGTTCTTGAGTGAGCGAGAAGTATGCGGAATGCGTGGTGTAGCGGTGAAATGCATAGATATCACGCAGAACCCCGATTGCGAAGGCAGCATACCGGCGCTCAACTGACGCTGAGGCACGAAAGTGCGGGTATCGAACAGG

>OTU159GTGGGGAATATTGGACAATGGACCAAAAGTCTGATCCAGCAATTCTGTGTGCACGATGAAGTTTTTCGGAATGTAAAGTGCTTTCAGTTGGGACGAAGTAAGTGACGGTACCAACAGAAGAAGCGACGGCTAAATACGTGCCAGCAGCCGCGGTAATACGTATGTCGCAAGCGTTATCCGGATTTATTGGGCGTAAAGCGCGTCTAGGCGGTTTGGTAAGTCTGATGTGAAAATGCGGGGCTCAACTCCGTATTGCGTTGGAAACTGCCAAACTAGAGTACTGGAGAGGTGGGCGGAACTACAAGTGTAGAGGTGAAATTCGTAGATATTTGTAGGAATGCCGATGGGGAAGCCAGCCCACTGGACAGATACTGACGCTAAAGCGCGAAAGCGTGGGTAGCAAACAGG

>OTU160GTGGGGAATATTGCGCAATGGAGGCAACTCTGACGCAGCGATGCCGCGTGAGTGAGGAAGGGATTCGTCCCGCAGAGCTCAGTCGAAGGGGAAAAGAGAGATTCAACCCTTTTTTCAAGCCCCGGCTAACTACGTGCCAGCAGCCGCGGTAATACGTAGGGGGCGAGCGTTGTCCGGAATTACTGGGCGTAAAGGGCGCGTAGGCGGCGGCGGAGGCCTGTTGTAAAATATCCGGGCTCAACCCGGAGAGGCGACAGGAACCGAGTCAGCTGGAGTGCTGGAGAGGCAGGCAGAATTCCTGGTGTAGCGGTGAAATGCGCAGAGATCAGGAGGAATACCGGAGGCGAAGGCGGCCTGCTGGACAGCAACTGACGCTAAGGCGCGAGAGCGTGGGGAGCAAACAGG

>OTU161GTAAGGAATATTGGTCAATGGAGGCAACTCTGAACCAGCCATGCCGCGTGCAGGAAGACAGCCCTCTGGGTCGTAAACTGCTTTTATTCGGGAATAAACCTTTTTACGTGTAAGAAGCTGAATGTACCGAAAGAATAAGGATCGGCTAACTCCGTGCCAGCAGCCGCGGTAATACGGAGGATCCAAGCGTTATCCGGATTTATTGGGTTTAAAGGGTGCGTAGGCGGCCTATTAAGTCAGGGGTGAAAGACGGTAGCTCAACTATCGCAGTGCCTTTGATACTGATGGGCTTGAATGAACTAGAGGTAGGCGGAATGTGACAAGTAGCGGTGAAATGCATAGATATGTCACAGAACACCGATTGCGAAGGCAGCTTACTATGGTTTTATTGACGCTGAGGCACGAAAGCGTGGGGATCAAACAGG

>OTU162GTGGGGAATATTGGACAATGGGGGGAACCCTGATCCAGCCATGCCGCGTGTGTGAAGAAGGCCTTTTGGTTGTAAAGCACTTTAAGCGAGGAGGAGGCTACCGAGATTAATACTCTTGGATAGTGGACGTTACTCGCAGAATAAGCACCGGCTAACTCTGTGCCAGCAGCCGCGGTAATACAGAGGGTGCAAGCGTTAATCGGATTTACTGGGCGTAAAGCGCGCGTAGGTGGCCAATTAAGTCAAATGTGAAATCCCCGAGCTTAACTTGGGAATTGCATTCGATACTGGTTGGCTAGAGTATGGGAGAGGATGGTAGAATTCCAGGTGTAGCGGTGAAATGCGTAGAGATCTGGAGGAATACCGATGGCGAAGGCAGCCATCTGGCCTAATACTGACACTGAGGTGCGAAAGCATGGGGAGCAAACAGG

>OTU163GTCGAGAATCATTCGCAATGGACGAAAGTCTGACGGTGCGACGCCGCGTGGAGGACGAAGGCCCTAGGGTCGTAAACTCCTGTCATCAGAGAGTAAATGCTTAGTTAGTAACTGATCTAAGTCTTGATAGTATCTGAAGAGGAAGGGACGGCAAACTTCGTGCCAGCAGCCGCGGTAATACGAAGGTCCCAAGCGTTGTTCGGATTTACTGGGCGTAAAGCGTCTGTAGGCGGCATGGTAAGTCAGATGTGAAATCCCGGGGCTCAACCCCGGAACTGCATCCGATACTGCCAAGCTTGAGTATTGGAGGGGAGTCTGGAATTCTCGGTGTAGCAGTGAAATGCGTAGATATCGAGAGGAACACTAGTGGCGAAGGCGAGACTCTGGACAATAACTGACGCTGAGAGACGAAGGCCGGGGTAGCGAAAAGG

>OTU164GTGGGGAATTTTGGACAATGGGCGCAAGCCTGATCCAGCCATGCCGCGTGCGGGAAGAAGGCCTTCGGGTTGTAAACCGCTTTTGTCAGGGAAGAAAAGGTTCTGGCTAATACCTGGGACTCATGACGGTACCTGAAGAATAAGCACCGGCTAACTACGTGCCAGCAGCCGCGGTAATACGTAGGGTGCAAGCGTTAATCGGAATTACTGGGCGTAAAGCGTGCGCAGGCGGTTATGCAAGACAGAGGTGAAATCCCCGGGCTCAACCTGGGAACTGCCTTTGTGACTGCATGGCTAGAGTACGGTAGAGGGGGATGGAATTCCGCGTGTAGCAGTGAAATGCGTAGATATGCGGAGGAACACCGATGGCGAAGGCAATCCCCTGGACCTGTACTGACGCTCATGCACGAAAGCGTGGGGAGCAAACAGG

>OTU165GTGGGGAATCTTGGACAATGGGGGCAACCCTGATCCAGCGACGCCGCGTGTGTGAAGAAGGCCTGCGGGTTGTAAAGCACTTTTAGTGGGGATGAAAAGCACATCGCTAATACCGGTGTGTCTTGACCTAACCCAAAGAAAAAGCACCGGCTAACTCTGTGCCAGCAGCCGCGGTAATACAGAGGGTGCGAGCGTTAATCGGAATTACTGGGCGTAAAGCGTGCGTAGACGGCTATCTAAGTCGGGTGTGAAAGCCCCGGGCTCAACCTGGGAATTGCATTCGAGACTGAATAGCTAGGGTACGGTAGAGGGAAGCGGAATTTCCGGTGTAGCGGTGAAATGCGTAGATATCGGAAGGAACATCAGTGGCGAAAGCGGCTTCCTGGACCAGTACCGACGTTCAGGCACGAAAGCGTGGGGAGCAAACAGG

>OTU166GTGGGGAATCTTGCACAATGGGGGAAACCCTGATGCAGCGACGCCGCGTGAGCGATGAAGCCCTTCGGGGTGTAAAGCTCTTTCGGCAGGAACGATAATGACGGTACCTGAAGAAGAAGCTGCGGCTAACTACGTGCCAGCAGCCGCGGTAATACGTAGGCAGCAAGCGTTGTTCGGAGTTACTGGGCGTAAAGAGTGCGTAGGCGGTCCTCTAAGTTTGGTGTGAAATCTCCCGGCTCAACCGGGAGGGTGCGCCGAAAACTGGGGGGCTCGAGTGTGGGAGAGGTAAGCGGAATTCCTGGTGTAGCGGTGAAATGCGTAGATATCAGGAGGAACACCTGTGGTGTAGACGGCTTACTGGACCATTACTGACGCTGAGGCACGAAAGCGTGGGTAGCAAACAGG

>OTU167GTAAGGAATATTGGACAATGGACGAAAGTCTGATCCAGCCATCCCGCGTGCAGGATGACGGCCCTATGGGTTGTAAACTGCTTTTATGAGAGAAGAAACGCCTTAATTTATTTAGGGTCTGACGGTATCTCATGAATAAGCACCGGCTAACTCCGTGCCAGCAGCCGCGGTAATACGGAGGGTGCAAGCGTTATCCGGAATTACTGGGTTTAAAGGGTGCGTAGGCGGTTAAGTAAGTCAGAGGTGAAATGTTATCGCTCAACGATAAAATTGCCTTTGATACTGCTTAACTTGAATCAGAATGAGGTTGGCGGAATGTGACATGTAGCGGTGAAATGCATAGATATGTCATGGAACACCGATTGCGAAGGCAGCTGACTGGCACTTGATTGACGCTGAGGCACGAAAGCGTGGGGAGCGAACAGG

>OTU168GTAGGGAATATTGGTCAATGGATGGAAGTCTGAACCAGCCATGCCGCGTGCAGGAAGAAGGCCTTCTGGGTTGTAAACTGCTTTTGCCAGGGGATAAAATGACCGTGCGCGGTTAATTGAAGGTACCTGGTGAATAAGCCACGGCTAACTACGTGCCAGCAGCCGCGGTAATACGTAGGTGGCAAGCGTTGTCCGGATTTATTGGGTTTAAAGGGTGCGTAGGCGGTCTGTTAAGTCAGTGGTGAAATATGGCAGCTTAACTGTCAGGGTGCCATTGATACTGATGGACTTGAGTGAAGTCGAAGTAGGCGGAATTGACGGTGTAGCGGTGAAATGCTTAGATATCGTCAAGAACACCGATAGTGAAGACAGCTTACTAGGCTTATACTGACGCTGAGGCACGAAAGTGTGGGGATCAAACAGG

>OTU169GTGGGGAATATTGGACAATGGGGGGAACCCTGATCCAGCGACGCCGCGTGAGTGAAGAAGTATTTCGGTATGTAAAGCTCTATCAGCAGGGAAGAAAGAAATGACGGTACCTGACTAAGAAGCCCCGGCTAAATACGTGCCAGCAGCCGCGGTAATACGTATGGGGCAAGCGTTATCCGGATTTACTGGGTGTAAAGGGAGCGCAGGCGGCTTGGCAAGTCTGATGTGAAAGGCTGGGGCTCAACCCCAGGACTGCATTGGAAACTGTTAGGCTGGAGTGTCGGAGAGGTAAGCGGAATTCCTGGTGTAGCGGTGAAATGCGTAGATATCAGGAGGAATACCGTTAGCGAAGGCGGCAATCTGGCTGGAAACTGACGCTGAGGTGCGAAAGCGTGGGTAGCAAACAGG

>OTU170GTGGGGAATATTGCACAATGGACGAAAGTCTGATGCAGCAACGCCGCGTGGAGGATGACACATTTCGGTGCGTAAACTCCTTTTATATGGGAAGATAATGACGGTACCATATGAATAAGCACCGGCTAACTCCGTGCCAGCAGCCGCGGTAATACGGAGGGTGCAAGCGTTACTCGGAATCACTGGGCGTAAAGAGCGTGTAGGCGGGTATATAAGTCAGAAGTGAAATCCAATAGCTTAACTATTGAACTGCTTTTGAAACTGTGTACCTAGAATGTGGGAGAGGTAGATGGAATTTCTGGTGTAGGGGTAAAATCCGTAGAGATCAGAAGGAATACCGATTGCGAAGGCGATCTACTGGAACATTATTGACGCTGAGACGCGAAAGCGTGGGGAGCAAACAGG

>OTU171GTGGGGAATCTTGCGCAATGGGCGAAAGCCTGACGCAGCCATGCCGCGTGAATGATGAAGGTCTTAGGATTGTAAAATTCTTTTACCAGGGACGATAATGACGGTACCTGGAGAAAAAGCCCCGGCTAACTTCGTGCCAGCAGCCGCGGTAATACGAAGGGGGCTAGCGTTGCTCGGAATTACTGGGCGTAAAGGGAGCGTAGGCGGGTTGACAAGTTGGAGGTGAAAGCCCAGGGCTCAACCCTGGAATTGCCTTCAAAACTGTCAGCCTTGAGTATGGAAGAGGTAAGTGGAACTCCGAGTGTAGAGGTGAAATTCGTAGATATTCGGAAGAACACCAGTGGCGAAGGCGACTTACTGGTCCATGACTGACGCTGAGGCTCGAAAGCGTGGGGAGCAAACAGG

>OTU172GTGGGGAATCTTAGACAATGGGGGCAACCCTGATCTAGCCATGCCGCGTGAGTGATGAAGGCCTTAGGGTTGTAAAGCTCTTTCAGCTGGGAAGATAATGACGGTACCAGCAGAAGAAGCCCCGGCTAACTCCGTGCCAGCAGCCGCGGTAATACGGAGGGGGCTAGCGTTGTTCGGAATTACTGGGCGTAAAGCGCACGTAGGCGGACCAGAAAGTTGGGGGTGAAATCCCGGGGCTCAACCCCGGAACTGCCTTCAAAACTATTGGTCTGGAGTTCGAGAGAGGTGAGTGGAATACCGAGTGTAGAGGTGAAATTCGTAGATATTCGGTGGAACACCAGTGGCGAAGGCGGCTCACTGGCTCGATACTGACGCTGAGGTGCGAAAGCGTGGGGAGCAAACAGG

>OTU173GTGGGGAATATTGCACAATGGGGGAAACCCTGATGCAGCGACGCCGCGTGAAGGAAGAAGTATCTCGGTATGTAAACTTCTATCAGCAGGGAAGAAAATGACGGTACCTGACTAAGAAGCCCCGGCTAACTACGTGCCAGCAGCCGCGGTAATACGTAGGGGGCAAGCGTTATCCGGATTTACTGGGTGTAAAGGGAGCGTAGACGGACTTGCAAGTCTGATGTGAAAATCCGGGGCCCAACCCCGGAACTGCATTGGAAACTGCGCGGCTCGAGTGCAGGAGGGGCAGGCGGAATTCCTAGTGTAGCGGTGAAATGCGTAGATATTAGGAGGAACACCAGTGGCGAAGGCGGCCTGCTGGACTGTAACTGACGTTGAGGCTCGAAAGCGTGGGGAGCAAACAGG

>OTU174GCTAAGAATCTTGCGCAATGGGCGAAAGCCTGACGCAGCAATTCCGCGTGGGGGACGAAGGCCCTAGGGTCGTAAACCCCTTTTGCGAGGGAAGAAGTTCTGACGGTACCTCGCGAATAAGCCACGGCTAACTACGTGCCAGCAGCCGCGGTAATACGTAGGTGGCAAGCGTTGTCCGGATTCATTGGGCGTAAAGCGCCCGCAGGCGGCTCAGCAAGTTTCAGGTGAAATCTCCCGGCTCAACTGGGAGGGGTCTTGGAAAACTGCTGGGCTTGAGGCAGGGAGAGGGATGCGGAATTCCAGGTGTAGTGGTGAAATGCGTAGATATCTGGAGGAACACCAGTGGCGAAGGCGGCATCCTGGCCCTGTCCTGACGCTCAGGGGCGAAAGCGTGGGGAGCAAACCGG

>OTU175GTGGGGGATATTGCACAATGGAGGAAACTCTGATGCAGCAATGCCGCGTGAGGGAAGACGGTTTTCGGATTGTAAACCTCTGTCCTTGGTGACGATAATGACGGTAGCCGAGGAGGAAGCTCCGGCTAACTACGTGCCAGCAGCCGCGGTAATACGTAGGGAGCAAGCGTTGTCCGGATTTACTGGGTGTAAAGGGTGCGTAGGCGGCTCTGTAAGTCGGGCGTGAAAGGTGTGGGCTTAACTCACAAACTGCGTTCGAAACTGTGGAGCTTGAGTGAAGCAGAGGCAGGCGGAATTCCCGGTGTAGCGGTGAAATGCGTAGAGATCGGGAGGAACACCAGTGGCGAAGGCGGCCTGCTGGGCTTTAACTGACGCTGAGGCACGAAAGCATGGGTAGCAAACAGG

>OTU176GTGGGGAATATTGCACAATGGGCGAAAGCCTGATGCAGCGACGCCGCGTGAGCGATGAAGGCCTTCGGGTCGTAAAGCTCTGTCCTAAGGGAAGAATTTTGACGGTACCTTAGGAGGAAGCCCCGGCTAACTACGTGCCAGCAGCCGCGGTAATACGTAGGGGGCAAGCGTTATCCGGAATCACTGGGCGTAAAGGGTGCGTAGGCGGCTCTTCAAGTCAGGGGTGAAAGGCTACGGCTCAACCGTAGTAAGCCTTTGAAACTGTAGAGCTTGAGTGCAGGAGAGGAGAGTGGAATTCCTAGTGTAGCGGTGAAATGCGTAGATATTAGGAGGAACACCAGTGGCGAAGGCGACTCTCTGGACTGTAACTGACGCTGAGGCACGAAAGCGTGGGGAGCAAACAGG

>OTU177GTGAGGAATATTGGTCAATGGACGGAAGTCTGAACCAGCCATGCCGCGTGCAGGAAGACGGCTCTATGAGTTGTAAACTGCTTTTGTACAAGGGTAAACCTGAATACGTGTATTCAGCTGAAAGTACTGTACGAATAAGGATCGGCTAACTCCGTGCCAGCAGCCGCGGTAATACGGAGGATCCAAGCGTTATCCGGATTTATTGGGTTTAAAGGGTGCGTAGGCGGATTGATAAGTTAGAGGTGAAATGTCCGAGCTTAACTCGGGAACTGCCTCTAATACTGTTGATCTAGAGAGTAGATGCGGTAGGCGGAATGTATGGTGTAGCGGTGAAATGCTTAGAGATCATACAGAACACCGATTGCGAAGGCAGCTTACCAATCTATATCTGACGTTGAGGCACGAAAGCGTGGGGAGCAAACAGG

>OTU178GTGGGGAATTTTGGACAATGGGGGCAACCCTGATCCAGCAATGCCGCGTGTGTGAAGAAGGCCTTCGGGTTGTAAAGCACTTTTGTCCGGAAAGAAATCGCTTCGGTTAATACCTGGAGTGGATGACGGTACCGGAAGAATAAGGACCGGCTAACTACGTGCCAGCAGCCGCGGTAATACGTAGGGTCCAAGCGTTAATCGGAATTACTGGGCGTAAAGCGTGCGCAGGCGGTTGTGCAAGACCGATGTGAAATCCCCGGGCTTAACCTGGGAATTGCATTGGTGACTGCACAGCTAGAGTGTGTCAGAGGGAGGTAGAATTCCACGTGTAGCAGTGAAATGCGTAGAGATGTGGAGGAATACCGATGGCGAAGGCAGCCTCCTGGGATAACACTGACGCTCATGCACGAAAGCGTGGGGAGCAAACAGG

>OTU179GTGGGGGATATTGGACAATGGGGGGAACCCTGATCCAGCGACGCCGCGTGAGTGAAGAAGTATTTCGGTATGTAAAGCTCTATCAGCAGGGAAGAAAGAAATGACGGTACCTGAGTAAGAAGCCCCGGCTAACTACGTGCCAGCAGCCGCGGTAATACGTAGGGGGCAAGCGTTATCCGGATTTACTGGGTGTAAAGGGAGCGTAGACGGTAATGCAAGTCTGGAGTGAAAGGCAGGGGCCCAACCCCTGGACTGCTTTGGAAACTGTGTAACTGGAGTGCAGGAGAGGTAAGTGGAATTCCTAGTGTAGCGGTGAAATGCGTAGATATTAGGAGGAACACCAGTGGCGAAGGCGGCCTGCTGGACGATGACTGACGCTGAGGCTCGAAAGCGTGGGGAGCAAACAGG

>OTU180GTGAGGAATATTGGTCAATGGGCGGAAGCCTGAACCAGCCAAGTCGCGTGAGGGAAGACGGTCCTATGGATTGTAAACCTCTTTTGTCAGGGAGCAAGAAGGGAACGCGTTCCCCAGTGAGAGTACCTGAAGAAAAAGCATCGGCTAACTCCGTGCCAGCAGCCGCGGTAATACGGAGGATGCGAGCGTTATCCGGATTTATTGGGTTTAAAGGGTGCGTAGGCGGACGGTTAAGTCAGCGGTAAAATCGAGGGGCTCAACCTCTTCCGGCCGTTGAAACTGGCCATCTTGAGTGAGCGAGAAGTATGCGGAATGCGTGGTGTAGCGGTGAAATGCATAGATATCACGCAGAACTCCGATTGCGAAGGCAGCATACCGGCGCTCAACTGACGCTGAAGCACGAAAGCGTGGGGATCAAACAGG

>OTU181GTGGGGAATCTTGGACAATGGGCGCAAGCCTGATCCAGCCATGCCGCGTGGATGATGAAGGCCCTAGGGTTGTAAAATCCTTTCGTCAGGGACGATAATGACGGTACCTGAAGAAGAAGCCCCGGCTAACTTCGTGCCAGCAGCCGCGGTAATACGAAGGGGGCTAGCGTTGTTCGGATTTACTGGGCGTAAAGCGCGTGTAGGCGGGCTTGCAAGTTGGGGGTGAAATCCCGAGGCTCAACCTCGGAACTGCCTCCAAAACTGCAAGCCTGGAGCAAGATAGAGGCAAGTGGAATTGCGAGTGTAGAGGTGAAATTCGTAGATATTCGCAGGAACACCAGTGGCGAAGGCGACTTGCTGGATCTTTGCTGACGCTGAGACGCGAAAGCGTGGGGAGCAAACAGG

>OTU182GTGAGGAATATTGGTCAATGGGCGAGAGCCTGAACCAGCCAAGTCGCGTGAGGGAGTACTGCCCTATGGGTTGTAAACCTCTTTTGTCGGGGAGCAAAAGCCGGACGTGTCCGTCTGTGAGAGTACCCGAAGAAAAAGCATCGGCTAACTCCGTGCCAGCAGCCGCGGTAATACGGAGGATGCGAGCGTTATCCGGATTTATTGGGTTTAAAGGGTGCGCAGGCGGATTTTTAAGTCAGCGGTCAAATCGTGGGGCTCAACCCCATCCAGCCGTTGAAACTGGGGATCTAGAGTGTGCGAGAGGTATGCGGAATGCGTGGTGTAGCGGTGAAATGCATAGATATCACGCAGAACCCCGATTGCGAAGGCAGCATACCGGTGCACAACTGACGCTCAGGCACGAAAGCGTGGGTATCGAACAGG

>OTU183GTGGGGAATATTGGACAATGGGCGCAAGCCTGATCCAGCAATGCCGCGTGTGTGAAGAAGGTCTTCGGATCGTAAAGCACTTTCGGCGGGGACGATGATGACGGTACCCGCAGAAGAAGCCCCGGCTAACTTCGTGCCAGCAGCCGCGGTAATACGAAGGGGGCTAGCGTTGCTCGGAATTACTGGGCGTAAAGGGCGCGTAGGCGGCTTGGCCAGTCAGGCGTGAAATTCCTGGGCTTAACCTGGGGACTGCGCTTGATACTGCTGAGCTTGAGGCCGAGAGAGGGTGGTGGAATTCCCAGTGTAGAGGTGAAATTCGTAGATATTGGGAAGAACACCGGTGGCGAAGGCGACCACCTGGCTCGGTACTGACGCTGAGGCGCGACAGCGTGGGGAGCAAACAGG

>OTU184GTAAGGAATATTGGGCAATGGAGGCAACTCTGACCCAGCCATCCCGCGTGCAGGATGACTGCCCTATGGGTTGTAAACTGCTTTTACAAGGGAAGAAACAGGTTTATTTATAAGCCTCTGACGGTACCTTGGGAATAAGCACCGGCTAACTCCGTGCCAGCAGCCGCGGTAATACGGAGGGTGCAAGCGTTATCCGGAATCACTGGGTTTAAAGGGTGCGTAGGCGGCTTAGTAAGTCAGAAGTGAAAGCCCGTCGCTCAACGATGGAATTGCTTTTGATACTGCTGAGCTTGAATTGAGTTGAGGCTGGCGGAATGTGGCATGTAGCGGTGAAATGCATAGATATGCCATAGAACACCAATTGCGAAGGCAGCTGGCTGGGCTTTGATTGACGCTGAGGCACGAAAGCGTGGGGAGCGAACAGG

>OTU185GTGGGGAATATTGGACAATGGGGGCAACCCTGATCCAGCCATGCCGCGTGAGTGATGAAGGCCTTCGGGTTGTAAAGCTCTTTTGGCGGGGACGATGATGACGGTACCCGCAGAATAAGCCCCGGCTAACTTCGTGCCAGCAGCCGCGGTAAGACGAAGGGGGCTAGCGTTGTTCGGAATTACTGGGCGTAAAGCGCGTGTAGGCGGTTGTTCAAGTCGGGTGTGAAAGCCTTGAGCTCAACTCAAGAAATGCACTCGGTACTGGATGACTAGAGGACCGGAGAGGATAGTGGAATTCCCAGTGTAGTGGTGAAATACGTAGAGATTGGGAAGAACACCAGTGGCGAAGGCGGCTATCTGGACGGTTTCTGACGCTAAGACGCGAAAGCGTGGGGAGCAAACAGG

>OTU186GTGGGGAATATTGGACAATGGGCGCAAGCCTGATCCAGCCATGCCGCGTGAGTGATGACGGCCTTAGGGTTGTAAAGCTCTTTCGGCGGGGACGATAATGACGGTACCCGCAGAAGAAGCCCCGGCTAACTTCGTGCCAGCAGCCGCGGTAATACGAAGGGGGCTAGCGTTGTTCGGAATCACTGGGCGTAAAGGGCGCGTAGGCGGCTTGATAAGTCGGGGGTGAAAGCCTGTGGCTCAACCACAGAATTGCCTTCGATACTGTCTGGCTTGAGACCGGAAGAGGTAAGTGGAACTGCGAGTGTAGAGGTGAAATTCGTAGATATTCGCAAGAACACCAGTGGCGAAGGCGGCTTACTGGTCCGGTTCTGACGCTGAGGCGCGAAAGCGTGGGGAGCAAACAGG

>OTU187GTGGGGAATATTGGACAATGGGGGCAACCCTGATCCAGCAATGCCGCGTGTATGAAGAAGGTCTTCGGATTGTAAAGTACTTTCGGTAGGGACGATGATGACGGTACCTACAGAAGAAGCCCCGGCTAACTTCGTGCCAGCAGCCGCGGTAATACGAAGGGGGCTAGCGTTGCTCGGAATGACTGGGCGTAAAGGGCGCGCAGGCGGTCTGGACAGTCAGATGTGAAATTCCCGGGCTTAACCTGGGGGCTGCATTTGATACGTTCAGACTAGAGTGTGAAAGAGGGTTGTGGAACTCCAAGTGTAGAGGTGAAATTCGTAGATATTTGGAAGAACACCGGTGGCGAAGGCGGCGACCTGGTTCACAACTGACGCTGAGGCGCGAAAGCGTGGGGAGCAAACAGG

>OTU188GTGGGGAATATTGGGCAATGGGCGCAAGCCTGACCCAGCAACGCCGCGTGAAGGAAGAAGGCTTTCGGGTTGTAAACTTCTTTTATTGGGGACGAAACAAATGACGGTACCCGATGAATAAGCTCCGGCTAACTACGTGCCAGCAGCCGCGGTAATACGTAGGGAGCAAGCGTTATCCGGATTTACTGGGTGTAAAGGGCGTGTAGGCGGGAAAGCAAGTCAGATGTGAAAACCAGGGGCTCAACCTCTGGCCTGCATTTGAAACTGTTTTTCTTGAGTACTGGAGAGGCAGGCGGAATTCCGTGTGTAGCGGTGAAATGCGTAGATATACGGAGGAACACCAGTGGCGAAGGCGGCCTGCTGGACAGCAACTGACGCTGAGGCGCGAAAGCGTGGGGAGCAAACAGG

>OTU189GTGGGGAATATTGGGCAATGGGGGAAACCCTGACCCAGCAACGCCGCGTGAAGGAAGAAGGCCTTCGGGTTGTAAACTTCTTTTACCAGGGACGAAGGACGTGACGGTACCTGGAGAAAAAGCAACGGCTAACTACGTGCCAGCAGCCGCGGTAATACGTAGGTTGCAAGCGTTATCCGGATTTACTGGGTGTAAAGGGAGCGTAGACGGCATTGCAAGCCAGATGTGAAAGCCCGGGGCTCAACCCCGGGACTGCATTTGGAACTGCAGGGCTGGAGTGTCGGAGAGGCAAGTGGAATTCCTAGTGTAGCGGTAAAATGCGCAGAGATATGGAAGAACACCAGTGGCGAAGGCGGCCGCCTGGCCTGTGACTGACGCTGAGGCACGAAAGCGTGGGGAGCAAATAGG

>OTU190GTGGGGAATTTTGGACAATGGGCGCAAGCCTGATCCAGCAATGCCGCGTGCAGGAAGAAGGCCTTCGGGTTGTAAACTGCTTTTGTCAGGGAAGAAATCTTCTGGGCTAATACCCCGGGAGGATGACGGTACCTGAAGAATAAGCACCGGCTAACTACGTGCCAGCAGCCGCGGTAATACGTAGGGTGCGAGCGTTAATCGGAATTACTGGGCGTAAAGCGTGCGCAGGCGGCTTTGCAAGACAGATGTGAAATCCCCGGGCTCAACCTGGGAACTGCATTTGTGACTGCAAGGCTAGAGTACGGCAGAGGGGGATGGAATTCCGCGTGTAGCAGTGAAATGCGTAGATATGCGGAGGAACACCAATGGCGAAGGCAATCCCCTGGGCCTGTACTGACGCTCATGCACGAAAGCGTGGGGAGCAAACAGG

>OTU191GTGAGGAATATTGGTCAATGGGCGAGAGCCTGAACCAGCCAAGTAGCGTGCAGGATGACGGCCCTATGGGTTGTAAACTGCTTTTGTATGGGAATAAAGTACTCCACGTGTGGAGTTTTGTAGGTACCATACGAATAAGGACCGGCTAATTCCGTGCCAGCAGCCGCGGTAATACGGAAGGTCCGGGCGTTATCCGGATTTATTGGGTTTAAAGGGAGCGTAGGCCGTGGATTAAGTGTGTTGTGAAATGTAGGCGCTCAACGTCTGACTTGCAGCGCATACTGGTCCACTTGAGTGCGCACAACGCGGGCGGAATTTGTCGTGTAGCGGTGAAATGCTTAGATATGACGAAGAACCCCGATTGCGAAGGCAGCTCGCGGGAGCGCAACTGACGCTGAAGCTCGAAAGTGCGGGTATCGAACAGG

>OTU192GTCGGGAATATTGCGCAATGGAGGAAACTCTGACGCAGTGACGCCGCGTGCAGGAAGAAGGTTTTCGGATTGTAAACTGCTTTAGACAGGGAAGAAAAAAATGACAGTACCTGTAGAATAAGCTCCGGCTAACTACGTGCCAGCAGCCGCGGTAATACGTAGGGAGCAAGCGTTATCCGGATTTATTGGGTGTAAAGGGTGCGTAGACGGGAAATTAAGTTAGTTGTGAAATCCCTCGGCTTAACTGAGGAACTGCAACTAAAACTGATTTTCTTGAGTGCTGGAGAGGAAAGTGGAATTCCTAGTGTAGCGGTGAAATGCGTAGATATTAGGAGGAACACCAGTGGCGAAGGCGACTTTCTGGACAGTAACTGACGTTGAGGCACGAAAGTGTGGGGAGCAAACAGG

>OTU193GTGGGGAATTTTGGACAATGGACGAAAGTCTGATCCAGCGACGCCGCGTGGAGGACGAAGGTTTTCGGATCGTAAACTCCTTTTACAGGGGACGAAAGCGCGCAAGCGTTTGACGGTACTCTGTGAATAAGCCACGGCTAACTACGTGCCAGCAGCCGCGGTAATACGTAGGTGGCGAGCGTTACTCGGAATTACTAGGCGTAAAGCGCGTGTAGGCGGGAGCTTAAGTCTGTCGTTAAAGCTCAGGGCTCAACCCTGAAATGTCGACGGAAACTGGGCTTCTTGAGTGCGGTAGAGGAGATCGGAATTCCAGGTGTAGCGGTGAAATGCGTAGATATCTGGAAGAACGCCAAAGGCGAAGGCAGATCTCTGGGCCGTTACTGACGCTGAGACGCGAAAGCTAGGGGAGCAAACAGG

>OTU194GTAAGGGATATTGCGACAATGGGCGAGAGCCTGACGCAGCAACGCCGCGTGCGGGAGGAAGGCCTTCGGGTTGTAAACCGCTTTTGAGGGGGAAGAGGAAGGACGGTACCCTTCGAATAAGTCTCGGCTAACTACGTGCCAGCAGCCGCGGTAAAACGTAGGAGGCGAGCGTTATCCGGATTTACTGGGTGTAAAGCGCGTGCAGGCGGTGGGACAAGTTAACTGTGAAAGCGCCCGGCTCAACCGGGCGAGGACAGTTAAGACTGTTTTGCTGGAGGCAGGTAGAGGTGTGTGGAATTCCGGGTGTAGTGGTGAAATGCGTAGAGATCCGGAGGAACACCAGTGGCGAAGGCGACACACTGGGCCTGGCCTGACGCTCAGACGCGAAAGCATGGGGAGCGAACGGG

>OTU195GTAAGGAATATTGGTCAATGGACGCAAGTCTGAACCAGCCATGCCGCGTGGAGGATGAAGGTCCTCTGGATTGTAAACTTCTTTTATCTGGGAAGAAACCTGTCTTTTCTTGGGCAGCTGACGGTACCAGATGAATAAGCACCGGCTAACTCCGTGCCAGCAGCCGCGGTAATACGGAGGGTGCAAGCGTTATCCGGATTCACTGGGTTTAAAGGGTGCGTAGGTGGGCTGATAAGTCAGTGGTGAAATCTCTAGGCTTAACCTGGAAACTGCCATTGATACTATCGGTCTTGAATCTCGCGTAGGTAAGCGGAATATGTCATGTAGCGGTGAAATGCTTAGATATGACATAGAACACCAATTGCGAAGGCAGCTTACTTCACGAGTATTGACACTGAGGCACGAAAGCGTGGGGAGCAAACAGG

>OTU196GTGGGGAATATTGCACAATGGGCGGAAGCCTGATGCAGCGACGCCGCGTGAGGGATGACGGCCTTCGGGTTGTAAACCTCTTTCAGCAGGGACGAAGCGTAAGTGACGGTACCTGCAGAAGAAGCACCGGCTAACTATGTGCCAGCAGCCGCGGTAATACGTAGGGTGCGAGCGTTGTCCGGAATTACTGGGCGTAAAGAGCTCGTAGGCGGTTTGTCACGTCGTCTGTGAAATCCTAGGGCTTAACCCTGGACGTGCAGGCGATACGGGCTGACTTGAGTACTACAGGGGAGACTGGAATTTCTGGTGTAGCGGTGGAATGCACAGATATCAGGAAGAACACCGATGGCGAAGGCAGGTCTCTGGGTAGTAACTGACGCTGAGGAGCGAAAGCATGGGTAGCGAACAGG

>OTU197GTGGGGAATCTTGGACAATGGGCGAAAGCCCGATCCAGCAATATCGCGTGAGTGAAGAAGGGCAATGCCGCTTGTAAAGCTCTTTCGTCGAGTGCGCGATCATGACAGGACTCGAGGAAGAAGCCCCGGCTAACTCCGTGCCAGCAGCCGCGGTAAGACGGGGGGGGCAAGTGTTCTTCGGAATGACTGGGCGTAAAGGGCACGTAGGCGGTGAATCGGGTTGAAAGTGAAAGTCGCCAAAAACTGGTGGAATGCTCTCGAAACCAATTCACTTGAGTGAGACAGAGGAGAGTGGAATTTCGTGTGTAGGGGTGAAATCCGCAGATCTACGAAGGAACGCCAAAAGCGAAGGCAGCTCTCTGGGTCCCTACCGACGCTGGAGTGCGAAAGCATGGGGAGCGAACGGG

>OTU198GTGGGGAATATTGGACAATGGGCGAAAGCCTGATCCAGCAATGCCGCGTGGGTGAAGAAGGTCTTCGGATTGTAAAGCCCTTTCGGCGGGGACGATGATGACGGTACCCGCAGAAGAAGCCCCGGCTAACTTCGTGCCAGCAGCCGCGGTAATACGAAGGGGGCTAGCGTTGCTCGGAATGACTGGGCGTAAAGGGCGCGTAGGCGGCTCATAGAGTCGGGCGTGAAATTCCTGGGCTTAACCCGGGGGCTGCGTTCGATACCTGTGGGCTCGAGTGAGGAAGAGGGTCGTGGAATTCCCAGTGTAGAGGTGAAATTCGTAGATATTGGGAAGAACACCGGTGGCGTAGGCGGCGACCTGGTCCTTTACTGACGCTGAGGCGCGAAAGCGTGGGGAGCAAACAGG

>OTU199GCAAGGAATCTTGGGCAATGGGCGAAAGCCTGACCCAGCGACGCCGCGTGAGGGATGAAGGCCTTCGGGTCGTAAACCTCTTTTATCGGGGAAGAAGTTCTGACGGTACCCGATGAATAAGCCACGGCTAACTACGTGCCAGCAGCCGCGGTAATACGAAGGGGGCTAGCGTTGTTCGGATTTACTGGGCGTAAAGCGCGTGTAGGCGGGCCGGTAAGTTGGGGGTGAAATCCCGAGGCTCAACCTCGGAACTGCCTCCAAAACTGGCGGCCTGGAGCAAGATAGAGGCGAGTGGAATTGCGAGTGTAGAGGTGAAATTCGTAGATATTCGCAGGAACACCAGTGGCGAAGGCGACTCGCTGGATCTTTGCTGACGCTGAGACGCGAAAGCGTGGGGAGCAAACAGG

>OTU200GCAAGGAATCTTCCGCAATGGGGGCAACCCTGACGGAGCAACGCCGCGTGGGGGACGACACCCTTCGGGGCGTAAACCCCTTTTCTCCGGGAAGAAGCGCTTCGGCGTTGACGGTACCGGAGGAAGAAGGCCCGGCTAACCACGTGCCAGCAGCCGCGGTAATACGTGGGGGCCGAGCGTTGTCCGGAGTTACTGGGCGTAAAGGGCGCGCAGGCGGCGGCGTGGGTCCGGCGTGAAAGCCCCCGGCTCAACCGGGGAACGTCGCCGGGGACCGCGCCGCTTGAGGGCGGCAGGGGCCGGTGGAATGCCCGGTGTAGTGGTGAAATGCGTAGAGATCGGGCGGAACACCCGTGGCGAAGGCGGCCGGCTGGGCCGTCCCTGACGCTGAGGCGCGAAGGCGTGGGGAGCGAACGGG

>OTU201GTGAGGAATATTGGTCAATGGACGAGAGTCTGAACCAGCCAAGTAGCGTGAAGGATGACTGCCCTATGGGTTGTAAACTTCTTTTATATGGGAATAAAAAAGAGCACGTGTGCTCTATTGTATGTACCTTATGAATAAGCATCGGCTAATTCCGTGCCAGCAGCCGCGGTAATACGGAAGATGCGAGCGTTATCCGGATTTATTGGGTTTAAAGGGAGCGTAGGCGGACGATTAAGTCAGCGGTAAAATAGAGTGGCTCAACCATTCTCCGCCGTTGATACTGGTTGTCTTGAGTTCACACAAGGAAGATGGAATTCGTGGTGTAGCGGTGAAATGCTTAGATATCACGAAGAACTCCGATTGCGAAGGCAGTCTTCTGGGGTGTTACTGACGCTGAGGCTCGAAAGTGCGGGTATCAAACAGG

>OTU202GTGGGGAATATTGCACAATGGGGGGAACCCTGATGCAGCGACGCCGCGTGAGGGAAGAAGGTTTTCGGATTGTAAACCTCTGTCTTGTGGGACGATAATGACGGTACCACAGGAGGAAGCCATGGCTAACTACGTGCCAGCAGCCGCGGTAATACGTAGATGGCGAGCGTTGTCCGGAATTACTGGGTGTAAAGGGAGTGTAGGCGGGATCATAAGTTGCGTGTGAAATGCAGGGGCTCAACCCCTGAACTGCGCGCAAAACTGTGGTTCTTGAGTGAAGTAGAGGCAGGCGGAATTCCCGGTGTAGCGGTGGAATGCGTAGATATCGGGAGGAACACCAGTGGCGAAGGCGGCCTGCTGGGCTTTTACTGACGCTGAGGCTCGAAAGCATGGGGAGCAAACAGG

>OTU203GTGAGGAATATTGGACAATGGGTTAGCGCCTGATCCAGCCATCCCGCGTGAAGGACGACGGCCCTATGGGTTGTAAACTTCTTTTGTACAGGGATAAACCTACTCTCGTGAGAGTAGCTGAAGGTACTGTACGAATAAGCACCGGCTAACTCCGTGCCAGCAGCCGCGGTAATACGGAGGGTGCAAGCGTTATCCGGATTTATTGGGTTTAAAGGGTCCGTAGGCGGATCTGTAAGTCAGTGGTGAAATCTCACAGCTTAACTGTGAAACTGCCATTGATACTGCAGGTCTTGAGTAAGGTAGAAGTGGCTGGAATAAGTAGTGTAGCGGTGAAATGCATAGATATTACTTAGAACACCAATTGCGAAGGCAGGTCACTATGTCTTAACTGACGCTGATGGACGAAAGCGTGGGGAGCGAACAGG

>OTU204GTAAGGGATCTTGCGACAATGGGCGAAAGCCTGACGCAGCAACGCCGCGTGCGGGAGGACGGCCTTCGGGTTGTAAACCGCTTTTCGGGGGGAAGAGGAAGGACGGTACCCCCGGAAGAAGTCTCGGCTAACTACGTGCCAGCAGCCGCGGTAAAACGTAGGAGGCGAGCGTTATCCGGATTTACTGGGTGTAAAGCGCGTGTAGGCGGTCCTGCAAGTGGTGCGTGAAAGCGCCCGGCTCAACCGGGCGAGGACGTGGGCGAACTGCAGGGCTAGAGGCAGGTAGAGGCGTGTGGAATTCCGGGTGTAGTGGTGAAATGCGTAGAGATCCGGAGGAACACCAGTGGCGAAGGCGACACGCTGGGCCTGGCCTGACGCTGAGAGGCGAAAGCATGGGGAGCGAACGGG

>OTU205GTGGGGAATATTGCGCAATGGCCGAAAGGCTGACGCAGCGACGCCGCGTGGGGGATGAAGCATTTCGGTGTGTAAACCCCTGTTGCCCGGGACGAACAACTGGTTTCGACCAGTCTGACGGTACCGGGTGAGGAAGCACCGGCTAACTCCGTGCCAGCAGCCGCGGTAATACGGAGGGTGCGAGCGTTGTCCGGAATCACTGGGCGTAAAGGGTGAGTAGGCGGTCTGTTAAGCGTGTGGTGAAAGCCTGGGGCTCAACCCCAGGTCTGCCATGCGAACTGGTGGACTTGAGCACTGTAGAGGCAGGTGGAATTCCGGGTGTAGCGGTGGAATGCGTAGAGATCCGGAAGAACACCGGTGGCGAAGGCGGCCTGCTGGGCAGTTGCTGACGCTGAATCACGACAGCGTGGGGAGCAAACAGG

>OTU206GTGGGGGATATTGGACAATGGGGGGAACCCTGATCCAGCGACGCCGCGTGAGTGAAGAAGTATCTCGGTATGTAAAGCTCTGTCAGCAGGGAAGAAAGATATGACGGTACCCCCAAAGGAAGCACCGGCTAACTCCGTGCCAGCAGCCGCGGTAATACGGAGGGTGCGAGCGTTAATCGGAATCACTGGGCGTAAAGCGCACGTAGGTGGCGTGGTAAGTCGGGGGTGAAATCCCACAGCTCAACTGTGGAACTGCCTTCGATACTGTCATGCTTGAGTATCGGAGAGGGTGGCGGAATTCCAGGTGTAGGAGTGAAATCCGTAGATATCTGGAAGAACACCGGTGGCGAAGGCGGCCACCTGGACGAAAACTGACGCTGAGGTGCGAAAGCGTGGGTAGCAAACAGG

>OTU207GTGAGGAATATTGGTCAATGGCCGGAAGGCTGAACCAGCCAAGTCGCGTGAGGGAATAAGGCCCTATGGGTCGTAAACCTCTTTTGTCAGGGAGCAAAGGCGTCCACGTGTGGACGAAAGGAGAGTACCTGAAGAAAAAGCATCGGCTAACTCCGTGCCAGCAGCCGCGGTAATACGGAGGATGCGAGCGTTATCCGGATTTATTGGGTTTAAAGGGTGCGTAGGCGGATTTTTAAGTCAGCGGTAAAATGTCCGGGCTCAACCCGGGCCGGCCGTTGAAACTGGGGATCTTGAGTGGGCGAGAAGTATGCGGAATGCGTGGTGTAGCGGTGAAATGCATAGATATCACGCAGAACTCCGATTGCGAAGGCAGCATACCGGCGCCCGACTGACGCTGAGGCACGAAAGCGTGGGGATCGAACAGG

>OTU208GTGGGGAATATTGCGCAATGGGGGCAACCCTGACGCAGCAACGCCGCGTGCGGGATGAAGGCTTTCGGGTTGTAAACCGCTTTCAGCAGGGATGACATCGACAGTACCTGCAGAAGAAGCCCCGGCTAACTACGTGCCAGCAGCCGCGGTAATACGTAGGGGGCGAGCGTTATCCGGATTCATTGGGCGTAAAGCGCGCGCAGGCGGGCTTGCAAGCGGGGCCTTTAATCTTGGGGCTTAACCTCAAGTCGGGTTCCGAACTGCAAGCCTCGAGTGTGGTAGGGGAAGGCGGAATTCCCGGTGTAGCGGTGGAATGCGCAGATATCGGGAAGAACACCGATGGCGAAGGCAGCCTTCTGGGCCATTACTGACGCTGAGGCGCGAAAGCTGGGGGAGCGAACAGG

>OTU209GTGGGGAATCTTGCGCAATGCGCGAAAGCGTGACGCAGCAACGCCGCGTGGGGGAAGAAGGCCTTCGGGTTGTAAACCCCTTTCAGTTGGGACGAAGCTTCGCCGGTGAATAGCCGGCCGGAGTGACGGTACCTTCAGAAGAAGCCCCGGCTAACTACGTGCCAGCAGCCGCGGTAATACGTAGGGGGCAAGCGTTGTCCGGAATCATTGGGCGTAAAGAGCGTGTAGGCGGCTCGGTAAGTCCGCTGTGAAAGTCCAGGGCTCAACCCTGGAAAGCCGGTGGAAACTGTCGAGCTAGAGTCCGGAAGAGGCGAGTGGAATTCCTGGTGTAGCGGTGAAATGCGCAGATATCAGGAGGAACACCAATGGCGAAGGCAGCTCGCTGGGACGGTACTGACGCTGAGACGCGAAAGCGTGGGGAGCAAACAGG

>OTU210GTGGGGAATATTGGACAATGGGCGAAAGCCTGATCCAGCAATGCCGCGTGAGTGATGAAGGCCTTAGGGTTGTAAAGCTCTTTTACCCGGGATGATAATGACAGTACCGGGAGAATAAGCCCCGGCTAACTCCGTGCCAGCAGCCGCGGTAATACGGAGGGGGCTAGCGTTGTTCGGAATTACTGGGCGTAAAGCGCACGTAGGCGGCTTTGTAAGTTAGAGGTGAAAGCCCAGAGCTCAACTCTGGAACTGCCTTTAAGACTGCATCGCTTGAATTGTGGAGAGGTAAGTGGAATTCCGAGTGTAGAGGTGAAATTCGTAGATATTCGGAAGAACACCAGTGGCGAAGGCGACTTACTGGACACATATTGACGCTGAGGTGCGAAAGCGTGGGGAGCAAACAGG

>OTU211GTCGAGAATTTTTCTCAATGGGGGAAACCCTGAAGGAGCGACGCCGCGTGGGGGATGAATGGCTTCGGCCCGTAAACCCCTGTCATTTGCGAACAAACCTCGTTATTTAAAAGATGACGAGCTGATGGTAGCGAAAGAGGAAGGGACGGCTAACTCTGTGCCAGCAGCCGCGGTAATACAGAGGTCCCAAGCGTTGTTCGGATTCACTGGGCGTAAAGGGTGCGTAGGTGGTTGGGTAAGTCTGATGTGAAATCTCCGAGCTCAACTCGGAAAATGCATTGGAAACTACCTGGCTGGAGGGTTGGAGGGGGGACTGGAATTCTCGGTGTAGCAGTGAAATGCGTAGATATCAAGAGGAACACCAGTGGCGAAGGCGAGTCCCTGGACAACACCTGACACTGAGGCACGAAAGCCGGGGGAGCAAACAGG

>OTU212GTGGGGAATATTGCGCAATGGGGGCAACCCTGACGCAGCAACGCCGCGTGATTGATGAAGGTCTTCGGATTGTAAAAATCTTTAATCAGGGACGAAGAAAATGACGGTACCTGAAGAATAAGCTCCGGCTAACTACGTGCCAGCAGCCGCGGTAATACGTAGGGAGCAAGCGTTATCCGGATTTACTGGGTGTAAAGGGCGTGTAGGCGGGCTTGTAAGTTGGAAGTGAAATCTCGGGGCTTAACCCCGAAACTGCTTTCAAAACTGCGAGTCTTGAGTGATGGAGAGGCAGGCGGAATTCCCAGTGTAGCGGTGAAATGCGTAGATATTGGGAGGAACACCAGTGGCGAAGGCGGCCTGCTGGACATTAACTGACGCTGAGGCGCGAAAGCGTGGGGAGCAAACAGG

>OTU213GTGGGGAATATTGCGCAATGGGCGAAAGCCTGACGCAGCGACGCCGCGTGAGGGATGAAGGTCTTCGGATCGTAAACCTCTGTCAGGGGGGAAGAAACGTGTCTATTCAAATAGAGTAGACATTTGACGGTACCCCCAAAGGAAGCACCGGCTAACTCCGTGCCAGCAGCCGCGGTAATACGGAGGGTGCAAGCGTTAATCGGAATTACTGGGCGTAAAGCGCACGTAGGTGGTTTTGTAAGTCAGAGGTGAAATCCCACCGCTCAACGGTGGAACAGCCTTTGATACTGCATCACTAGAGTATCGGAGAGGGTGGCGGAATTCCAGGTGTAGGAGTGAAATCCGTAGATATCTGGAGGAACACCAGTGGCGAAGGCGGCCACCTGGACGAAGACTGACACTGAGGTGCGAAAGCATGGGGAGCAAACAGG

>OTU214GTGAGGAATTTTCCGCAATGGGCGCAAGCCTGACGGAGCAACGCCGCGTGCAGGATGACGGCCTTCGGGTTGTAAACTGCTTTTCTGAGGGACGAGCAAGGACGGTACCTCAGGAATCAGCTCCGGCTAACTACGTGCCAGCAGCCGCGGTAATACGTAGGGAGCAAGCGTTGTCCGGAGTTACTGGGCGTAAAGCGCACGCAGGCGGTGAGCCAAGTTTGGAGTGACAGTCCTCGGCTTAACTGGGGAAGGACTTCGAAAACTGGCTCACTTGAGGGCTTCAGAGGGACAGGGAATTCCGGGTGTAGTGGTGAAATGCGTAGATATCCGGAGGAACACCAAAGGCGAAGGCACTGTCCTGGGGAGTCACTGACGCTCAGGTGCGAAAGCTAGGGGAGCGAACGGG

>OTU215GTGGGGAATATTGGTCAATGGGCGAGAGCCTGAACCAGCCAAGTCGCGTGAGGGAAGACGGTCCTATGGATTGTAAACCTCTTTTGTCGGGGAGCAAGAAAGGGTATGTATACCCTACTGAGAGTACCCGAAGAAAAAGCATCGGCTAACTCCGTGCCAGCAGCCGCGGTAATACGGAGGATGCGAGCGTTATCCGGATTTATTGGGTTTAAAGGGTGCGCAGGCGGACTGTTAAGTCAGCGGTAAAATTGCGGGGCTCAACCCCGTCGAGCCGTTGAAACTGGTGGTCTGGAGTGGGCGAGAAGTATGCGGAATGCGCAGTGTAGCGGTGAAATGCATAGATATTGCGCAGAACTCCGATTGCGAAGGCAGCATACCGGTTCCATACTGACGCTGAGGCACGAAAGCGTGGGGATCAAACAGG

>OTU216GTTAGGAATCTTCCACAATGGGCGCAAGCCTGATGGAGCGACGCCGCGTGAGGGATGAAGGTTCTCGGATCGTAAACCTCTGAACTAGGGACGAAAGAGCCGTATGGCAGATGACGGTACCTAGGTAATAGCACCGGCTAACTCCGTGCCAGCAGCCGCGGTAATACGGAGGGTGCAAGCGTTACCCGGAATCACTGGGCGTAAAGGGCGTGTAGGCGGAATGTTAAGTCTGGTTTTAAAGACTGGGGCTCAACCCCAGGAGTGGACTGGATACTGGCAATCTTGACCTCTGGAGAGGTAACTGGAATTCCTGGTGTAGCGGTGGAATGCGTAGATACCAGGAGGAACACCAATGGCGAAGGCAAGTTACTGGACAGAAGGTGACGCTGAGGCGCGAAAGTGTGGGGAGCAAACCGG

>OTU217GTAGGGAATATTGGGCAATGGACGCAAGTCTGACCCAGCCATGCCGCGTGCAGGATGAAGGCGCTCAGCGTTGTAAACTGCTTTTGATAGGGAAGAACGGCCTTCTTGCGAGAAGGTGTGACGGTACCTACAGAATAAGCACCGGCTAACTCCGTGCCAGCAGCCGCGGTAATACGGAGGGTGCAAGCGTTGTCCGGATTTATTGGGTTTAAAGGGTGCGTAGGTGGCTTTTTAAGTCTGACCTGAAAGTGGGCCGCTTAACGGCACAGGGTGGTTGGATACTGAAGAGCTTGAAGAGGGTGGAGGCCGCCGGAACGGATCGTGTAGCGGTGAAATGCATAGAGATGATCCAGAACCCCGATTGCGTAGGCAGGCGGCTACGCCCCACTTGACACTGAGGCACGAGAGCATGGGGAGCAAACAGG

>OTU218GTAGGGAATCTTCCACAATGGACGAAAGTCTGATGGAGCAACGCCGCGTGAGTGAAGAAGGGTTTCGGCTCGTAAAGCTCTGTTGGTAGTGAAGAAAGATAGAGGTAGTAACTGGCCTTTATTTGACGGTAATTACTTAGAAAGTCACGGCTAACTACGTGCCAGCAGCCGCGGTAATACGTAGGTGGCAAGCGTTGTCCGGATTTATTGGGCGTAAAGCGAGTGCAGGCGGTTCAATAAGTCTGATGTGAAAGCCTTCGGCTCAACCGGAGAATTGCATCAGAAACTGTTGAACTTGAGTGCAGAAGAGGAGAGTGGAACTCCATGTGTAGCGGTGGAATGCGTAGATATATGGAAGAACACCAGTGGCGAAGGCGGCTCTCTGGTCTGCAACTGACGCTGAGGCTCGAAAGCATGGGTAGCGAACAGG

>OTU219GTGGGGAATATTGCACAATGGGCGCAAGCCTGATGCAGCGACGCCGCGTGGGGGATGACGGCCTTCGGGTTGTAAACTCCTTTCGTCAGGGACGAAGCGAAAGTGACGGTACCTGGATAAGAAGCACCGGCTAACTACGTGCCAGCAGCCGCGGTAATACGTAGGGTGCGAGCGTTGTCCGGAATTACTGGGCGTAAAGAGCTCGTAGGTGGTTTGTCGCGTCGTTTGTGTAAGTCCACGGCTTAACTGTGGGATGGCAGGCGATACGGGCATAACTTGAGTGCTGTAGGGGAGACTGGAATTCCTGGTGTAGCGGTGAAATGCGCAGATATCAGGAGGAACACCGATGGCGAAGGCAGGTCTCTGGGCAGTAACTGACGCTGAGGAGCGAAAGCATGGGTAGCGAACAGG

>OTU220GTAGGGAATATTGGTCAATGGATGGAAGTCTGAACCAGCCATGCCGCGTGCAGGAAGACGGCCTTCTGGGTTGTAAACTGCTTTTGCCAGGGGCTAAAAAGCTCATGCGTGAGAAATTGAAAGTACCTGGTGAATAAGCCACGGCTAACTACGTGCCAGCAGCCGCGGTAATACGTAGGTGGCAAGCGTTGTCCGGATTTATTGGGTTTAAAGGGTGCGTAGGCGGCTTTGTAAGTCAGTGGTGAAATATGGCAGCTCAACTGTCAGGGTGCCATTGATACTGCGGAGCTTGAGTACAGATGAGGTAGGCGGAATTGACGGTGTAGCGGTGAAATGCTTAGATATCGGGAAGAACACCTATGGCGAAGGCAGCTCTCTGGGACGGTACTGACGCTGAGACGCGAAAGCGTGGGGAGCGAACAGG

>OTU221GTAGGGAATATTGGACAATGGGCGCAAGCCTGATCCAGCCATCCCGCGTGAAGGATTAAGGTCCTATGGATTGTAAACTTCTTTTCTCTGGGAATAAAAACCGGTATTTATACTGGCTTGAAGGTACCAGAGGAATAAGCACCGGCTAACTCCGTGCCAGCAGCCGCGGTAATACGGAGGGTGCAAGCGTTATCCGGATTCACTGGGTTTAAAGGGTGCGTAGGTGGCTTTGTAAGTCAGTGGTGAAAGCCCGGAGCTTAACTCCGGAACTGCCATTGATACTGCTTAGCTTGAATCAACTTGAGGTGGATGGAATATTACATGTAGCGGTGAAATGCTTAGATATGACATAGAACACCAATTGCGAAGGCAGCTGGCTACGCGAATATTGACACTGAGGCACGAAAGCGTGGGGATCAAACAGG

>OTU222GTGAGGAATATTGGTCAATGGCCGCAGGGCTGAACCAGCCAAGTCGCGTGAGGGATGACGGTCCTATGGATTGTAAACCTCTTTTGTCAGGGAGCAAAGGACGCCACGCGTGGTGTTTTGCGAGTACCTGAAGAAAAAGCATCGGCTAACTCCGTGCCAGCAGCCGCGGTAATACGGAGGATGCGAGCGTTATCCGGATTTATTGGGTTTAAAGGGTGCGCAGGCGGAATGTCAAGTCAGCGGTAAAATTTCGGGGCTCAACCCCGTCGTGCCGTTGAAACTGGCGTTCTTGAGTGAGCGAGAAGTATGCGGAATGCGTGGTGTAGCGGTGAAATGCATAGATATCACGCAGAACTCCGATTGCGAAGGCAGCATACCGGCGCTCAACTGACGCTCATGCACGAAAGCGTGGGTATCGAACAGG

>OTU223GTAGGGAATCTTCCACAATGGACGAAAGTCTGATGGAGCAACGCCGCGTGAGTGAAGAAGGTTTTCGGATCGTAAAACTCTGTTATCGGAGAAGAACGTATCTGGTAGTAACTGGCCAGGTAGTGACGGTATCCGATCAGAAAGCCACGGCTAACTACGTGCCAGCAGCCGCGGTAATACGTAGGTGGCAAGCGTTGTCCGGATTTATTGGGCGTAAAGCGAGTGCAGGCGGTTTTTTAAGTCTGATGTGAAAGCCTTCGGCTTAACCGAAGAAATGCATTGGAAACTGGGGAACTTGAGTGCAGAAGAGGAGAGTGGAACTCCATGTGTAGCGGTGAAATGCGTAGATATATGGAAGAACACCAGTGGCGAAGGCGGCTCTCTGGTCTGTAACTGACGCTGAGGCTCGAAAGCGTGGGTAGCAAACAGG

>OTU224GTGGGGAATATTGGACAATGGGCGCAAGCCTGATCCAGCAATGCCGCGTGGGTGAAGAAGGTCTTCGGATCGTAAAGCCCTTTCGGCGGGGACGATGATGACGGTACCCGCAGAAGAAGCCCCGGCTAACTTCGTGCCAGCAGCCGCGGTAATACGAAGGGGGCTAGCGTTACTCGGAATTACTGGGCGTAAAGGGCGCGTAGGCGGCGCACCAAGTTAGGCGTGAAAGCCCTGGGCTCAACCTGGGGACTGCGCTTAAGACTGGTGTGCTTGAGGATGGAAGAGGCTCGTGGAATTCCCAGTGTAGAGGTGAAATTCGTAGATATTGGGAAGAACACCGGTGGCGAAGGCGGCGAGCTGGTCCATTACTGACGCTGAGGCGCGATAGCGTGGGGAGCAAACAGG

>OTU225GTGGGGAATATTGCACAATGGGGGAAACCCTGATGCAGCCATGCCGCGTGTGTGAAGAAGGCCTTCGGGTTGTAAAGCACTTTCAGCGAGGAGGAAAGGGTGTAAGTTAATACCTTACATCTGTGACGTTACTCGCAGAAGAAGCACCGGCTAACTCCGTGCCAGCAGCCGCGGTAATACGGAGGGTGCGAGCGTTAATCGGAATTACTGGGCGTAAAGCGTGCGCAGGCGGTTTGTTAAGCGAGATGTGAAAGCCCCGGGCTCAACCTGGGAACCGCATTTCGAACTGGCAAACTAGAGTCTTGTAGAGGGGGGTAGAATTCCAGGTGTAGCGGTGAAATGCGTAGAGATCTGGAGGAATACCGGTGGCGAAGGCGGCCCCCTGGACAAAGACTGACGCTCAGGCACGAAAGCGTGGGGAGCAAACAGG

>OTU226GTGGGGAATCTTGGACAATGGGCGCAAGCCTGATCCAGCCATGCCGCGTGAGTGATGAAGGCCTTAGGGTCGTAAAGCTCTTTCGCCAGGGATGATAATGACAGTACCTGGTAAAGAAACCCCGGCTAACTCCGTGCCAGCAGCCGCGGTAATACGGAGGGGGTTAGCGTTGTTCGGAATTACTGGGCGTAAAGCGCGCGTAGGCGGATCAGAAAGTTGGGGGTGAAATCCCGGGGCTCAACCCCGGAACTGCCTCCAAAACTCCTGGTCTTGAGTTCGAGAGAGGTGAGTGGAATTCCGAGTGTAGAGGTGAAATTCGTAGATATTCGGAGGAACACCAGTGGCGAAGGCGGCTCACTGGCTCGATACTGACGCTGAGGTGCGAAAGTGTGGGGAGCAAACAGG

>OTU227GTGGGGAATATTGGACAATGGGCGCAAGCCTGATCCAGCAACGCCGCGTGGAGGACGAAGGTCTTCGGATCGTAAACTCCTGTCAGGCGGGACGAAGGCTGCAGGGCAAATAGTCCTGTAGCTTGACGGTACTGCCAGAGGAAGCCCCGGCTAACTCCGTGCCAGCAGCCGCGGTAATACGGAGGGGGCGAGCGTTATTCGGAATTATTGGGCGTAAAGGGCGCGTAGGCGGCCCGGTAAGTCAAAGGTGAAATCCCTCGGCTCAACTGAGGAACTGCCTTTGAAACTGTCGGGCTTGAGGCCGGGAGGGGGTAGCGGAATTCCCAGTGTAGCGGTGAAATGCGTAGATATTGGGAGGAACACCGGTGGCGAAGGCGGCTACCTGGACCGGTTCTGACGCTGATGCGCGAAAGCGTGGGTAGCAAACAGG

>OTU228GTTAGGGATATTGGGCAATGGAGGAAACTCTGACCCAGCAATGCCGCGTGAGTGAAGAAGTACTTCGGTATGTAAAGCTCTGTTGTATAGGAGGAAAGAACTAGTAGGAAATGGCTAGGAGATGACTGTACTATACGAGAAAGTCACGGCTAACTACGTGCCAGCAGCCGCGGTAATACGTAGGTGGCAAGCGTTATCCGGAATTATTGGGCGTAAAGGGTGCGTAGGTGGCATGTTAAGTCTGGTGTAAAAGGCAATAGCTCAACTATTGTATGCATTGGAAACTGGCAAGCTAGAGTGTGTGAGGGGTAAGTGGAATTCCAAGTGTAGCGGTGGAATGCGTAGATATTTGGAGGAACACCAGTGGCGAAGGCGGCTTACTGGCACACAACTGACACTGAGGCACGACAGCGTGGGGAGCAAATAGG

>OTU229GTAAGGAATATTGGTCAATGGACGCAAGTCTGAACCAGCCATGCCGCGTGGAGGATGAAGGTCCTCTGGATTGTAAACTTCTTTTATCTGGGACGAAAAAAGGGAATTCTTTCTCGTCTGACGGTACCAGATGAATAAGCACCGGCTAACTCCGTGCCAGCAGCCGCGGTAATACGGAGGGTGCAAGCGTTATCCGGATTCACTGGGTTTAAAGGGTGCGTAGGCGGGTATGTAAGTCCGTGGTGAAATCTCCGAGCTTAACTCGGAAACTGCCGTGGGTACTGCGTATCTTGAATGTTGTGGAGGTGAGCGGAATATGTCATGTAGCGGTGAAATGCTTAGATATGACATAGAACACCAATTGCGAAGGCAGCTCACTACACAAATATTGACGCTGAGGCACGAAAGCGTGGGGATCAAACAGG

>OTU230GTGGGGAATATTGCACAATGGGCGCAAGCCTGATGCAGCAACGCCGCGTGAGGGATGACGGCCTTCGGGTTGTAAACCTCTTTTAGTAGGGAAGAAGCGAAAGTGACGGTACCTGCAGAAAAAGCACCGGCTAACTACGTGCCAGCAGCCGCGGTAATACGTAGGGTGCAAGCGTTGTCCGGAATTATTGGGCGTAAAGAGCTCGTAGGCGGCTTGTCGCGTCTGCTGTGAAATCCCGGGGCTCAACCCCGGGCCTGCAGTGGGTACGGGCAAGCTAGAGTGCGGTAGGGGAGATTGGAATTCCTGGTGTAGCGGTGGAATGCGCAGATATCAGGAGGAACACCGATGGCGAAGGCAGATCTCTGGGCCGCTACTGACGCTGAGGAGCGAAAGCATGGGGAGCGAACAGG

>OTU231GTTAGGAATCTTCCACAATGGGCGAAAGCCTGATGGAGCGACGCCGCGTGAGGGATGAAGGTTTTCGGATCGTAAACCTCTGAATCAGGGACGAAAGACGCGCAAGCGGGATGACGGTACCTGAGTAATAGCACCGGCTAACTCCGTGCCAGCAGCCGCGGTAATACGGAGGGTGCAAGCGTTACCCGGAATCACTGGGCGTAAAGGGCGTGTAGGCGGATCGTTAAGTCTGGTTTTAAAGACCGTGGCTCAACCACGGGAGTGGACTGGATACTGGCAATCTTGACCTCTGGAGAGGTAACTGGAATTCCTGGTGTAGCGGTGGAATGCGTAGATACCAGGAGGAACACCAATGGCGAAGGCAAGTTACTGGACAGAAGGTGACGCTGAGGCGCGAAAGTGTGGGGAGCGAACCGG

>OTU232GTGAGGAATATTGGTCAATGGACGCAAGTCTGAACCAGCCATGCCGCGTGCAGGAAGACGGCTCTATGAGTTGTAAACTGCTTTTGTATTAGGGTAAACTCAGGTACGTGTACCTGACTGAAAGTATAATACGAATAAGGATCGGCTAACTCCGTGCCAGCAGCCGCGGTAATACGGAGGATCCAAGCGTTATCCGGATTTATTGGGTTTAAAGGGTGCGTAGGCGGTTTGATAAGTTAGAGGTGAAATACCGGGGCTTAACTCCGGAACTGCCTCTAATACTGTTGAACTAGAGAGTAGTTGCGGTAGGCGGAATGTATGGTGTAGCGGTGAAATGCTTAGAGATCATACAGAACACCGATTGCGAAGGCAGCTTACCAAACTATATCTGACGTTGAGGCACGAAAGCGTGGGGAGCAAACAGG

>OTU233GTGGGGAATATTGCACAATGGAGGAAACTCTGATGCAGCGACGCCGCGTGAGTGAAGAAGTAATTCGTTATGTAAAGCTCTATCAGCAGGGAAGATAGTGACGGTACCTGACTAAGAAGCTCCGGCTAAATACGTGCCAGCAGCCGCGGTAATACGTATGGAGCAAGCGTTATCCGGATTTACTGGGTGTAAAGGGAGTGTAGGTGGCATCACAAGTCAGAAGTGAAAGCCCGGGGCTCAACCCCGGGACTGCTTTTGAAACTGTGGAGCTAGAGTGCAGGAGGGGCAAGTGGAATTCCTAGTGTAGCGGTGAAATGCGTAGATATTAGGAGGAACACCAGTGGCGAAGGCGGCTTGCTGGACTGTAACTGACACTGAGGCTCGAAAGCGTGGGGAGCAAACAGG

>OTU234GTAGGGAATATTGGTCAATGGGCGAGAGCCTGAACCAGCCATGCCGCGTGAAGGATGAAGGCGTTCTGCGTTGTAAACTTCTTTTATCTGGGAAGAAAAAGGGCCTGCGGGCCAAATTGACGGTACCAGATGAATAAGCACCGGCTAACTCCGTGCCAGCAGCCGCGGTAATACGGAGGGTGCAAGCGTTGTCCGGATTTATTGGGTTTAAAGGGTGCGTAGGCGGGTCATTAAGTCAGTGGTGAAAGCCCACAGCTCAACTGTGGAACTGCCATTGATACTGGTGATCTTGAGTATAGATGAGGCAGGCGGAATTTACGATGTAGCGGTGAAATGCATAGATATCGTAAAGAACACCTATAGCGAAGGCAGCTTGCTAAACTATAACTGACGCTGAGGCACGAAAGCATGGGTAGCGAACAGG

>OTU235GTAGGGAATCTTCCGCAATGGACGAAAGTCTGACGGAGCAACGCCGCGTGAGTGAAGAAGGTTTTCGGATCGTAAAACTCTGTTGTTAGGGAAGAACAAGTCAGGTAGTAACTGACCTGACCTTGACGGTACCTAACCAGAAAGCCACGGCTAACTACGTGCCAGCAGCCGCGGTAATACGTAGGTGGCAAGCGTTGTCCGGATTTATTGGGCGTAAAGCGCTCGCAGGCGGTCTTTTAAGTCTGATGTGAAAGCCCACGGCTTAACCGTGGAGGGTCATTGGAAACTGGAGGACTTGAGTGCAGAAGAGGAGAGTGGAATTCCATGTGTAGCGGTGAAATGCGTAGAGATATGGAGGAACACCAGTGGCGAAGGCGACTCTCTGGTCTGTAACTGACGCTGAGGAGCGAAAGCGTGGGTAGCGAACAGG

>OTU236GTGAGGAATATTGGTCAATGGGCGAGAGCCTGAACCAGCCAAGTAGCGTGAAGGATTACTGCCCTACGGGTTTTAAACTTCTTTTATAAGGGAATAAAGTCGTCCACGTGTGGGCGTTTGCATGTACCTTATGAATAAGGATCGGCTAACTCCGTGCCAGCAGCCGCGGTAATACGGAGGATCCGAGCGTTATCCGGATTTATTGGGTTTAAAGGGTGCGTAGGCCGGCGTGTAAGTCAGCGGTGAAAAGTTGGTGCTCAACATTGACCCTGCCGTTGATACTGCATGCCTTGAGTGCGGATAAGGAAGGCGGAACTCGTGGTGTAGCGGTGAAATGCTTAGATATCACGATGAACCCCGATTGCGAAGGCAGCCTTCCGGGCCGCAACTGACGCTGAAGCACGAAAGTGTGGTTATCAAACAGG

>OTU237GTGGGGAATCTTGGACAATGGGGGCAACCCTGATCCAGCCATGCCGCGTGAGTGAAGAAGGCCTTAGGGTCGTAAAGCTCTTTCGCCTGTGAAGATAATGACGGTAGCAGGTAAAGAAACCCCGGCTAACTCCGTGCCAGCAGCCGCGGTAATACGGAGGGGGTTAGCGTTGTTCGGAATTACTGGGCGTAAAGCGTACGTAGGCGGATCAGAAAGTAAGGGGTGAAATCCCGGGGCTCAACCCCGGAACTGCCTCTTAAACTCCTGGTCTTGAGTTCGAGAGAGGTGAGTGGAATTCCGAGTGTAGAGGTGAAATTCGTAGATATTCGGAGGAACACCAGTGGCGAAGGCGGCTCACTGGCTCGATACTGACGCTGAGGTACGAAAGTGTGGGGAGCAAACAGG

>OTU238GTAGGGAATATTGCATAATGGGCGAAAGCCTGATGCAGCAACGCCGCGTGCGCGATGAAGGCCTTCGGGTCGTAAAGCGCTTTTCAGAGGGATGAGAAAGGACAGTACCTCTGGAATAAGTCTCGGCTAACTACGTGCCAGCAGCCGCGGTAAAACGTAGGAGGCAAGCGTTATCCGGATTTACTGGGCGTAAAGCGTGTGCAGGCGGTTTGGAAAGTTGGATGTGAAAGCTCCCGGCTCAACTGGGAGAGGTCGTTCAAAACTTCCAGACTTGAGGATGGTAGAGGGAGGTGGAATTCCGGGTGTAGTGGTGAAATGCGTAGATATCCGGAGGAACACCAGTGGCGAAAGCGGCCTCCTGGACCATTTCTGACGCTCAGACACGAAAGCTAGGGGAGCAAACGGG

>OTU239GTGAGGAATATTGGTCAATGGCCGGAAGGCTGAACCAGCCAAGCCGCGTGAAGGACGAAGGTGCCAAGCATTGTAAACTTCTTTTGTCAAGGGACAAAATCAGGCTCGAGAGCCTGACCGAGGGTACTTGAAGAAAAAGCATCGGCTAACTCCGTGCCAGCAGCCGCGGTAATACGGAGGATGCGAGCGTTATCCGGATTTATTGGGTTTAAAGGGTGCGTAGGCGGGCTGTTAAGTCAGCGGTAAAAGCCCGGGGCTCAACCCCGGCGAGCCGTTGAAACTGGCTGTCTTGAGATGTCGAGAGGTATGCGGAATGCGCAGTGTAGCGGTGAAATGCTTAGATATTGCGCAGAACTCCGATTGCGAAGGCAGCATACCGGCGGCGATCTGACGCTGAGGCACGAAAGCGTGGGTATCGAACAGG

>OTU240GTGGGGAATATTGCGCAATGGGCGAAAGCCTGACGCAGCGACGCCGCGTGAGGGATGAAGGTCTTCGGATCGTAAACCTCTGTCAGCAGGGAAGAAAGTTATGCGTGCTAATCAGCGTGTAATTGACGGTACCTGCAAAGGAAGCACCGGCTAACTCCGTGCCAGCAGCCGCGGTAATACGGAGGGTGCGAGCGTTAATCGGAATTACTGGGCGTAAAGCGCTCGTAGGTAGTATATCAAGTCAAGGGTGAAATCCCCGCGCTCAACGTGGGAACTGCCTTTGAAACTGGTAGACTTGAGTGTGTGAGAGGATAGTGGAATTCCAGGTGTAGGAGTGAAATCCGTAGATATCTGGAGGAACATCAGTGGCGAAGGCGACTATCTGGCACATAACTGACACTGAGGAGCGAAAGCGTGGGTAGCAAACAGG

>OTU241GTGGGGAATCTTAGACAATGGGCGCAAGCCTGATCTAGCCATGCCGCGTGAGTGACGAAGGCCTTAGGGTCGTAAAGCTCTTTCGCTGGGGAAGATAATGACGGTACCCAGTAAAGAAACCCCGGCTAACTCCGTGCCAGCAGCCGCGGTAATACGGAGGGGGTTAGCGTTGTTCGGAATTACTGGGCGTAAAGCGCGCGTAGGCGGATTGGAAAGTTGGGGGTGAAATCCCGGAGCTTAACTCCGGAACTGCCTTTGAGACTGATAGGCTTGAGTTCGGGAGAGGTAAGTGGAATACCTAGTGTAGAGGTGAAATTCGTAGATATTAGGTGGAACACCAGTGGCGAAGGCGACTTACTGGACCGATACTGACGCTGAGGTGCGAAAGCGTGGGGAGCAAACAGG

>OTU242GTGGGGAATATTGCACAATGGGCGAAAGCCTGATGCAGCAACGCCGCGTGAGGGATGACGGCCTTCGGGTTGTAAACCTCTTTTAGCAGGGAAGAAGCGAAAGTGACGGTACCTGCAGAAAAAGCGCCGGCTAACTACGTGCCAGCAGCCGCGGTAATACGTAGGGCGCAAGCGTTATCCGGAATTATTGGGCGTAAAGAGCTCGTAGGCGGTTTGTCGCGTCTGCTGTGAAATCCCGAGGCTCAACCTCGGGCCTGCAGTGGGTACGGGCAGACTAGAGTGCGGTAGGGGAGATTGGAATTCCTGGTGTAGCGGTGGAATGCGCAGATATCAGGAGGAACACCGATGGCGAAGGCAGATCTCTGGGCCGTAACTGACGCTGAGGAGCGAAAGGGTGGGGAGCAAACAGG

>OTU243GTGGGGAATATTGGACAATGGGGGGAACCCTGATCCAGCCATGCCGCGTGTGTGAAGAAGGCCCTCGGGTTGTAAAGCACTTTCAGTGAGGAAGAACGCCTGGTGGTTAATACCCATCAGGAAAGACATCACTCACAGAAGAAGCACCGGCTAACTCCGTGCCAGCAGCCGCGGTAATACGGAGGGTGCGAGCGTTAATCGGAATTACTGGGCGTAAAGCGCGCGTAGGTGGCTTGATAAGCCGGTTGTGAAAGCCCCGGGCTCAACCTGGGAACGGCATCCGGAACTGTCAAGCTAGAGTGCAGGAGAGGAAGGTAGAATTCCCGGTGTAGCGGTGAAATGCGTAGAGATCGGGAGGAATACCAGTGGCGAAGGCGGCCTTCTGGACTGACACTGACACTGAGGTGCGAAAGCGTGGGTAGCAAACAGG

>OTU244GTGAGGAATATTGGTCAATGGGCGAGAGCCTGAACCAGCCAAGTAGCGTGAAGGATGAAGGTTCTATGGATTGTAAACTTCTTTTATAAGGGAATAACCGACACCACGTGTGGTGTTCTGCATGTACCTTATGAATAAGCATCGGCTAACTCCGTGCCAGCAGCCGCGGTAATACGGAGGATGCGAGCGTTATCCGGATTTATTGGGTTTAAAGGGAGCGTAGACGGGATGTTAAGTCAGCTGTGAAAGTTTGGGGCTCAACCTTAAAATTGCAGTTGAAACTGGCGTTCTTGAGTGCGGTAGAGGCAGGCGGAATTCGTGGTGTAGCGGTGAAATGCTTAGATATCACGAAGAACTCCAATTGCGAAGGCAGCCTGCTGGAGCGTAACTGACGTTGATGCTCGAAAGTGTGGGTATCAAACAGG

>OTU245GTGGGGAATTTTGGACAATGGGCGCAAGCCTGATCCAGCTATTCCGCGTGTGGGATGAAGGCCCTCGGGTTGTAAACCACTTTTGTAGAGAACGAAAAGACACCTTTTAATAAAGGGTGTTGCTGACGGTACTCTAAGAATAAGCACCGGCTAACTACGTGCCAGCAGCCGCGGTAATACGTAGGGTGCGAGCGTTAATCGGAATTACTGGGCGTAAAGGGTGCGCAGGCGGTTGAGTAAGACAGATGTGAAATCCCCGAGCTTAACTCGGGAATGGCATATGTGACTGCTCGACTAGAGTGTGTCAGAGGGAGGTGGAATTCCACGTGTAGCAGTGAAATGCGTAGATATGTGGAAGAACACCGATGGCGAAGGCAGCCTCCTGGGACATAACTGACGCTCAGGCACGAAAGCGTGGGGAGCAAACAGG

>OTU246GTGAGGAATATTGCACAATGGGGGAAACCCTGATGCAGCGACGCCGCGTGAGTGAAGAAGTATTTCGGTATGTAAAGCTCTATCAGCAGGGAAGAAAATGACGGTACCTGACTAAGAAGCACCGGCTAAATACGTGCCAGCAGCCGCGGTAATACGTATGGTGCAAGCGTTATCCGGATTTACTGGGTGTAAAGGGTGCGTAGGTGGTGAGACAAGTCTGAAGTGAAAATCCGGGGCTTAACCCCGGAACTGCTTTGGAAACTGCCTGACTGGAGTACAGGAGAGGTAAGTGGAATTCCTAGTGTAGCGGTGAAATGCGTAGATATTAGGAGGAACACCAGTGGCGAAGGCGACTTACTGGACTGGTACTGACACTGAGGCACGAAAGCGTGGGGAGCAAACAGG

>OTU247GTGAGGAATATTGGTCAATGGGCGGGAGCCTGAACCAGCCAAGCCGCGTGAGGGAATAAGGCCCTACGGGTCGTAAACCTCTTTTGTCGGGGAACAAAAGCGGGGACGCGTCCCCGTCTGCGTGTACCCGAAGAAAAAGCATCGGCTAACTCCGTGCCAGCAGCCGCGGTAATACGGAGGATGCGAGCGTTATCCGGATTTATTGGGTTTAAAGGGTGCGTAGGCGGTCCGGAAAGTCAGCGGTAAAACTTCGGGGCTCAACCCCGTTTAGCCGTTGAAACTGTCGGACTAGAGAGGAAGAGAAGCAGGCGGAATGCGCGGTGTAGCGGTGAAATGCATAGATATCGCGCAGAACTCCGATTGCGAAGGCAGCCTGCCGGCTTCTGTCTGACGCTGAGGCACGAAAGCGTGGGTATCGAACAGG

>OTU248GTGGGGAATATTGGGCAATGGAGGCAACTCTGACCCAGCAACGCCGCGTGAATGAAGAAGGCCTTCGGGTTGTAAAGTTCTTTAATGGGGGACGAAGAAAGTGACGGTACCCCAAGAATAAGCCACGGCTAACTACGTGCCAGCAGCCGCGGTAATACGTAGGTGGCAAGCGTTGTCCGGAATGACTGGGCGTAAAGGGAGCGTAGGCGGCGCAGTAAGTTAGGAGTGAAATCCCGAGGCTTAACCTCGGAACTGCTTTTAAAACTGCTGTGCTTGAGTGATGGAGAGGAAAGCGGAATTCCTAGTGTAGCGGTGGAATGCGTAGATATTAGGAGGAACACCAGTGGCGAAGGCGGCTTACTGGACTGTAACTGACGTTGAGGCTCGAAAGCGTGGGGAGCAAACAGG

>OTU249GTGGGGAATATTGCACAATGGGGGAAACCCTGATGCAGCGACGCCGCGTGAGTGAAGAAGTATTTCGGTATGTAAAGCTCTATCAGCAGGGAAGAAGAAATGACGGTACCTGAGTAAGAAGCCCCGGCTAACTACGTGCCAGCAGCCGCGGTAATACGTAGGGGGCAAGCGTTATCCGGAATTACTGGGTGTAAAGGGAGCGTAGACGGTGATGTAAGTCTGGAGTGAAAGGCGGGGGCCCAACCCCCGGACTGCTCTGGAAACTATGTGACTGGAGTGCAGGAGAGGTGAGCGGAATTCCTAGTGTAGCGGTGAAATGCGTAGATATTAGGAGGAACACCAGTGGCGAAGGCGGCTCACTGGACTGTAACTGACGTTGAGGCTCGAAAGCGTGGGGAGCAAACAGG

>OTU250GTGGGGAATATTGGACAATGGAGGAAACTCTGATGCAGCGACGCCGCGTGAGCGATGAAGCCCTTCGGGGTGTAAAGCTCTTTCGACGGGAAAGATGATGACGGTACCCGGAGAAGAAGCTGCGGCTAACTACGTGCCAGCAGCCGCGGTAATACGTAGGCAGCGAGCGTTGTTCGGAATTACTGGGCGTAAAGAGTGTGTAGGCGGTGTTCCAAGTCTGGTGTGAAATCTCCCGGCTTAACTGGGAGGGTGCGCCGGAAACTGGAATGCTCGAGGGTGGGAGAGGTAAGCGGAATTCCTGGTGTAGCGGTGAAATGCGTAGATATCAGGAGGAACACCTGTGGTGTAGACAGCTTACTGGACCACTTCTGACGCTGAGACACGAAAGCGTGGGTAGCAAACAGG

>OTU251GTTGGGAATCTTGCACAATGGGGGAAACCCTGATGCAGCGACGCCGCGTGGAGGATGACGGATCTAGGTCTGTAAACTCCTTTTTCATGGAAAGACTTAGGACGGTACCATGAGAATAAGGACCGGCTAACTACGTGCCAGCAGCCGCGGTAAGACGTAGGGTCCAAGCGTTGTCCGGATTTACTGGGCGTAAAGAGCGCGTAGGCGGTCTGTTAAGTGTGAAGTGAAATCTCCAGGGCTCAACCCGGAAACTGCTTTACATACTGGCAGACTTGAGGAATGCAGAGGTTTGTGGAATTCCTGGTGTAACGGTGAAATGTGTTGATATCAGGAGGAACACCCATGGCGAAGGCAGCAAACTGGGCATTATCTGACGCTGAGGCGCGAAAGCGTGGGTAGCAAACAGG

>OTU252GTGGGGAATATTCCGCAATGGGCGCAAGCCTGACGGAGCAACGCCGCGTGAATGAAGAAGGTTTTCGGATTGTAAAGTTCTGTCATTAGGGACGAAGGACCTGTGCGAATAGTGCAGGGGATGACGGTACCTAAGGAGGAAGCTCCGGCTAACTACGTGCCAGCAGCCGCGGTAATACGTAGGGAGCAAGCGTTGTCCGGAATTACTGGGCGTAAAGGGCGCGTAGGCGGATTGTTAAGTCAGATGTGAAATCTCCGGGCTCAACCCGGAGCGTGCATTTGAAACTGGCTATCTTGAGGGCAGGAGAGGAAAGTGGAATTCCTAGTGTAGCGGTGAAATGCGTAGATATTAGGAGGAACACCAGTGGCGAAGGCGACTTTCTGGACTGAACCTGACGCTGAGGCGCGAAAGCATGGGTAGCGAACGGG

>OTU253GTAGGGAATTTTCGTCAATGGGCGCAAGCCTGAACGAGCAATGCCGCGTGAACGAGGAAGGTCTTCGGATCGTAAAGTTCTGTTGAGAGGGAAAAAGGGTCACCAGAGGAAATGCTGGTGAAGTGATATTACCTTTCGAGGAAGTCACGGCTAACTACGTGCCAGCAGCCGCGGTAATACGTAGGTGGCGAGCGTTATCCGGAATGATTGGGCGTAAAGGGTGCGTAGGCGGCCTGTTAAGTCTGAAGTGAAAGGTACCGGCTCAACCGGTACAGGCTTTGGAAACTGGCAGGCTGGAGGACAGGAGAGGGCGGTGGAACTCCATGTGTAGCGGTAAAATGCGTAGATATATGGAAGAACACCAGTGGCGAAGGCGGCCGCCTGGCCTGTTACTGACGCTGAGGCACGAAAGCGTGGGGAGCAAATAGG

>OTU254GTGAGGAATATTGGTCAATGGGCGAGAGCCTGAACCAGCCAAGTAGCGTGCAGGATGACGGCCCTATGGGTTGTAAACTGCTTTTGTATGGGGATAAAGTCAGTCACGTGTGATTGTTTGCAGGTACCATACGAATAAGGACCGGCTAATTCCGTGCCAGCAGCCGCGGTAATACGGAAGGTCCGGGCGTTATCCGGATTTATTGGGTTTAAAGGGAGCGTAGGCTGGAGATTAAGTGTGTTGTGAAATGTAGACGCTCAACGTCTGACTTGCAGCGCATACTGGTTTCCTTGAGTACGCACAACGTTGGCGGAATTCGTCGTGTAGCGGTGAAATGCTTAGATATGACGAAGAACTCCGATTGCGAAGGCAGCTGACGGGAGCGCAACTGACGCTGAAGCTCGAAGGTGCGGGTATCGAACAGG

>OTU255GTTAGGAATCTTCCACAATGGGCGCAAGCCTGATGGAGCGACGCCGCGTGAGGGATGAAGGTTCTCGGATCGTAAACCTCTGAACCAACGACGAAAGACCCGACAAGGGAGATGACGGTAGTTGGGTAATAGCACCGGCTAACTCCGTGCCAGCAGCCGCGGTAATACGGAGGGTGCAAGCGTTACCCGGAATCACTGGGCGTAAAGGGCGTGTAGGCGGTTACCTAAGTCCGATTTTAAAGACCGAAGCTCAACTTCGGGAGTGGATTGGATACTGAGTGACTTGACCTCTGGAGAGGAAACCGGAATTCCTGGTGTAGCGGTGGAATGCGTAGATACCAGGAGGAACACCAATGGCGAAGGCAGGTTTCTGGACAGAAGGTGACGCTGAGGCGCGAAAGTGTGGGGAGCGAACCGG

>OTU256GTGGGGAATATTGGACAATGGGCGCAAGCCTGATCCAGCCATGCCGCGTGGATGATGAAGGCCTTAGGGTTGTAAAGTCCTTTCAGCGGGGAAGATAATGACGGTACCCGCAGAAGAAGCCCCGGCTAACTTCGTGCCAGCAGCCGCGGTAATACGGAGGGTGCAAGCGTTGTTCGGAATTACTGGGCGTAAAGCGCGTGTAGGCGGCTGGTTAGGTGTGATGTGAAATCTTCCGGCTCAACCGGAAAACTGCATTGCAAACCGGCCTGGCTAGAGTGCAGGAGAGGGAAGCGGAATTCCAGGTGTAGCGGTGAAATGCGTAGATATCTGGAGGAACACCTGCGGTGTAGACGGCTTTCTGGACCATTACTGACACTGAGACACGAAAGCGTGGGTAGCAAACAGG

>OTU257GTGGGGAATATTGCGCAATGGGGGAAACCCTGACGCAGCAATGCCGCGTGAGTGAAGAAGGCTTTTGGGTCGTAAAGCTCTTTCGACAGGAAAGAAGCCGTCACGTATAAATAATGCGTGGGGGTGACGGTACCTGAAGAAGAAGTACCGGCTAACTCCGTGCCAGCAGCCGCGGTAATACGGAGGGTACAAGCGTTGTTCGGAATTATTGGGCGTAAAGAGCACGTAGGCGGATTCGTATGTCGATTGTGAAATCCCAGGGCTTAACCTTGGAATTGCAGTCGAAACTGCGGATCTTGAATACTTGAGAGGGTGGGGGAATTCCTGGTGGAGAAGTGAAATTCGTAGAGATCAGGAGGAACACCGGAGGCGTAGGCGCCTGCCTGGCAAGATATTGACGCTGAGGTGCGAAAGCGTGGGGAGCAAACAGG

>OTU258GTGGGGAATTTTGCGCAATGGGGGAAACCCTGACGCAGCAACGCCGCGTGGATGATGAAGCCCTTTGGGGTGTAAAATCCTTTCGGCAGGGACGAAACCCGCGCAAGCGGGCCGACGGTACCTGAAGAAGAAGCCCCGGCTAACTCCGTGCCAGCAGCCGCGGTAATACGGGGGGGGCGAGCGTTGTTCGGAATTATTGGGCGTAAAGGGAGCGTAGGCGGTGCGGTAAGTCGGGTGTGAAACCTCCAGGCTCAACTTGGAGCTGGCATCCGAAACTGCCGTGCTAGAGTGCGGGAGAGGAGAGTGGAATTCCCGGTGTAGCGGTGAAATGCGTAGATATCGGGAGGAACATCGGTGGCGAAGGCGGCTCTCTGGACCGTAACTGACGCTGAGGCTCGAAAGCTAGGGGAGCAAACAGG

>OTU259GTGGGGAATATTGCACAATGGGCGCAAGCCTGATCCAGCCATGCCGCGTGTGTGAAGAAGGTCTTCGGATTGTAAAGCACTTTAAGTTGGGAGGAAGGGCAGTAAGTTAATACCTTGCTGTTTTGACGTTACCAACAGAATAAGCACCGGCTAACTTCGTGCCAGCAGCCGCGGTAATACGAAGGGTGCAAGCGTTAATCGGAATTACTGGGCGTAAAGCGCGCGTAGGTGGTTTTGTAAGTTGGAGGTGAAATCCCCGGGCTCAACCTGGGAACTGCACCTGATACTGCTGAGCTAGAGTACGGTAGAGGGTAGTGGAATTTCCGGTGTAGCGGTGAAATGCGTAGAGATCGGAAGGAACACCAGTGGCGAAGGCGACTACCTGGACCGATACTGACACTGAGGTGCGAAAGCGTGGGTAGCAAACAGG

>OTU260GTGGGGAATATTGCGCAATGGAGGGAACTCTGACGCAGCGATGCCGCGTGGGCGAAGAAGGACTTCGGTTCGTAAAGCCCTGTTGCCGGGGAAGAATAGAGACGGTACCCGGCGAGGAAGCCCCAGCCAACCACGTGCCAGCAGCTGCGGTAACACGTGGGGGGCGGGCGTTGTCCGGATTCATTGGGCGTAAAGGGCACGCAGGCGGCCGCGAGGGCGCGCCGTGAAAGGCCGGGGCCCAACCCCGGAACGGCGGCGCGAACCGCGCGGCTCGAGTGCCGGAGAGGCAGGCGGAACTCCTGGTGTAGCGGTGAAATGCGCAGAGATCAGGAGGAACACCGGAGGCGAAGGCGGCCTGCCGGCCGGGCACTGACGCTCAGGTGCGAAAGCGCGGGGAGCGAACAGG

>OTU261GTGGGGAATTTTGCACAATGGGGGAAACCCTGATGCAGCGACGCCGCGTGATTTAGAAGGCCTTCGGGTTGTAAAAATCTTTTGTATAGGAAGAAAATGACAGTACTATACGAATAAGGTCCGGCTAATTACGTGCCAGCAGCCGCGGTAATACGTAAGGACCGAGCGTTGTCCGGAATCATTGGGCGTAAAGGGTACGTAGGCGGGTTTTTAAGTTAGAAGTCAAAGGCTATAGCTCAACTATAGTAAGCTTCTAAAACTGGAAACCTTGAGTAATGGAAGGGAAAGTGGAATTCCTAGTGTAGCGGTGGAATGCGCAGATATTAGGAGGAATACCGGTGGCGAAGGCGACTTTCTGGCCATTTTCTGACGCTGAGGTACGAAAGCGTGGGTAGCAAACAGG

>OTU262GTAGGGAATCTTCCGCAATGGGCGAAAGCCTGACGGAGCAACGCCGCGTGAGTGATGAAGGTCTTAGGATCGTAAAACTCTGTTGTTAGGGAAGAACAAATTTGTTAGTAACTGAACAAGTCTTGACGGTACCTAACCAGAAAGCCACGGCTAACTACGTGCCAGCAGCCGCGGTAATACGTAGGTGGCAAGCGTTATCCGGAATTATTGGGCGTAAAGCGCGCGTAGGCGGTTTCTTAAGTCTGATGTGAAAGCCCACGGCTCAACCGTGGAGGGTCATTGGAAACTGGGGAACTTGAGTGCAGAAGAGGAGAGTGGAATTCCATGTGTAGCGGTGAAATGCGCAGAGATATGGAGGAACACCAGTGGCGAAGGCGGCTCTCTGGTCTGTAACTGACGCTGATGTGCGAAAGCGTGGGGATCAAACAGG

>OTU263GTAGGGAATATTGGGCAATGGCCGAGAGGCTGACCCAGCCATGCCGCGTGCAGGAAGAAGGCCTTCTGGGTTGTAAACTGCTTTTATATGGGAAGAAAACGCCTTTGCGAAGGTAACTGACGGTACCATATGAATAAGCACCGGCTAACTCCGTGCCAGCAGCCGCGGTAATACGGAGGGTGCAAGCGTTGTCCGGATTTATTGGGTTTAAAGGGTGCGTAGGCGGCCCGTTAAGTCAGCGGTGAAATCCCAGGGCTCAACCCTGGAACTGCCGTTGATACTGGCGGGCTTGAGTTCGGTCGAGGCGGGCGGAACTGGTGGTGTAGCGGTGAAATGCATAGATACCACCAAGAACCCCGATTGCGAAGGCAGCTCGCTGGCCCGAAACTGACGCTGAGGCACGAAAGCGTGGGGAGCGAACAGG

>OTU264GTGGGGAATTTTGGACAATGGGGGCAACCCTGATCCAGCCATGCCGCGTGTGTGAAGAAGGCCTTCGGGTTGTAAAGCACTTTCGGACGGAACGAAATCGCGCGGGCGAATATCCCGCGTGGATGACGGTACCGTAAGAAGAAGCACCGGCTAACTACGTGCCAGCAGCCGCGGTAATACGTAGGGTGCGAGCGTTAATCGGAATTACTGGGCGTAAAGTGTGCGCAGGCGGCCTCGCAAGTCGAGTGTGAAATCCCCGGGCTTAACTTGGGAATTGCGCTCGAAACTACGGGGCTGGAGTGTGGCAGAGGGGGGTGGAATTCCACGTGTAGCGGTGAAATGCGTAGAGATGTGGAGGAACACCAATGGCGAAGGCAGCCCCCTGGGCCAACACTGACGCTCATGCACGAAAGCGTGGGGAGCAAACAGG

>OTU265GTGGGGAATCTTGCACAATGGGGGCAACCCTGATGCAGCGACGCCGCGTGAGCGATGAAGCCCTTCGGGGTGTAAAGCTCTTTCGACCGGAACGATGATGACGGTACCGGGAGAAGAAGCTGCGGCTAACTACGTGCCAGCAGCCGCGGTAATACGTAGGCAGCGAGCGTTGTTCGGAGTTACTGGGCGTAAAGCGTGCGTAGGCGGTTTTCTAAGTCTGGTGTGAAATCTCCCGGCTTAACCGGGAGGGTGCGCCGGATACTGGAAGGCTTGGAGTGCGGGAGAGGTACGCGGAATTCCTGGTGTAGCGGTGAAATGCGTAGATATCAGGAGGAACACCGGCGGTGTAGACGGCGTACTGGACCGTGACTGACGCTGAGGCACGAAAGCGTGGGGAGCAAACAGG

>OTU266GTGAGGAATATTGGTCAATGGGCGAGAGCCTGAACCAGCCAAGTCGCGTGAGGGAAGACGGCCCTATGGGTTGTAAACCTCTTTTGTCGGGGAGCAAGGAGCACCACGCGTGGTGCGGCGAGAGTACCCGAAGAAAAAGCATCGGCTAACTCCGTGCCAGCAGCCGCGGTAATACGGAGGATGCGAGCGTTATCCGGATTTATTGGGTTTAAAGGGTGCGTAGGCGGCATTTCAAGTCAGCGGTAAAATTGAGAGGCTCAACCTCTTCGAGCCGTTGAAACTGGAGTGCTTGAGTGGGCGAGAAGTGTGCGGAATGCGTGGTGTAGCGGTGAAATGCATAGATATCACGCAGAACTCCGATTGCGAAGGCAGCACGCCGGCGCCTTACTGACGCTGAAGCACGAAAGCGTGGGTATCGAACAGG

>OTU267GTGGGGAATATTGCACAATGGGCGGAAGCCTGATGCAGCGACGCCGCGTGAGGGATGACGGCCTTCGGGTTGTAAACCTCTTTCGGCCCTGACGAAGCGTGAGTGACGGTAGGGGCAGAAGAAGCGCCGGCCAACTACGTGCCAGCAGCCGCGGTAAGACGTAGGGCGCAAGCGTTGTCCGGATTTATTGGGCGTAAAGAGCTCGTAGGCGGCTTGTCGCGTCGACTGTGAAATCCCGCAGCTCAACTGCGGGCTTGCAGCCGATACGGGCAGGCTAGAGTTCGGTAGGGGAGACTGGAATTCCTGGTGTAGCGGTGAAATGCGCAGATATCAGGAGGAACACCGGTGGCGAAGGCGGGTCTCTGGGCCGATACTGACGCTGAGGAGCGAAAGCGTGGGGAGCGAACAGG

>OTU268GTAGGGAATATTGCAAAATGGGCGAAAGCCTGATGCAGCAACGCCGCGTGGGCGATGAAGGCCTTCGGGTCGTAAAGCCCTTTTTGAGGGGATGAGGAAGGACAGTACCCTCAGAATAAGCCTCGGCTAACTACGTGCCAGCAGCCGCGGTAAAACGTAGGAGGCGAGCGTTATCCGGATTTACTGGGTGTAAAGCGCGTGCAGGCGGATTGGAAAGTTGGATGTGAAAGCTCCTGGCTCAACTAGGAGAGGTCGTTCAAAACTTCCAGTCTTGAGTACAGTAGAGGGAGGTGGAATTCCGGGTGTAGTGGTGAAATGCGTAGATATCCGGAGGAACACCAGTGGCGAAAGCGGCCTCCTGGACTGTAACTGACGCTCAGACGCGAAAGCTAGGGTAGCAAACGGG

>OTU269GTGGGGAATATTGCACAATGGGGGAAACCCTGATGCAGCAACGCCGCGTGAGTGAAGAAGTATTTCGGTATGTAAAGCTCTATCAGCAGGGAAGAAAATGACGGTACCTGACTAAGAAGCCCCGGCTAACTACGTGCCAGCAGCCGCGGTAATACGTAGGGGGCAAGCGTTATCCGGATTTACTGGGTGTAAAGGGAGCGTAGGTGGTGCGGCAAGTCAGATGTGAAAGCCCGGGGCTCAACCCCGGGACTGCATTTGAAACTGTCGGACTGGAGTGCAGGAGAGGTAAGTGGAATTCCTAGTGTAGCGGTGAAATGCGTAGATATTAGGAGGAACACCAGTGGCGAAGGCGGCTTACTGGACTGTAACTGACACTGAGGCTCGAAAGCGTGGGGAGCAAACAGG

>OTU270GTAGGGAATTTTCGGCAATGGGGGGAACCCTGACCGAGCAACGCCGCGTGAAGGAAGAAGGAATTCGTTCTGTAAACTTCTGTTATAAAGGAAGAAAGACGGATGGAGGAAATGACATCCGAGTGACGGTACTTTATGAGAAAGCCACGGCTAACTACGTGCCAGCAGCCGCGGTAATACGTAGGTGGCAAGCGTTATCCGGAATTATTGGGCGTAAAGAGGGAGCAGGCGGCAGCAAAGGTCTGTGGTGAAAGACTGAAGCTTAACTTCAGTAAGCCATAGAAACCGGGCAGCTAGAGTGCAGGAGAGGATCGTGGAATTCCATGTGTAGCGGTGAAATGCGTAGATATATGGAGGAACACCAGTGGCGAAGGCGACGATCTGGCCTGCAACTGACGCTCAGTCCCGAAAGCGTGGGGAGCAAATAGG

>OTU271GTAGGGAATCTTTCACAATGGGCGAAAGCCTGATGGAGCAACGCCGCGTGCAGGATGACGGCCTTCGGGTTGTAAACTGCTTTTATATCCGAGAAATATGATGGTAAGATATGAATAAGGACCGGCTAACTACGTGCCAGCAGCCGCGGTCATACGTAGGGTCCAAGCATTATCCGGATTGACTGGGTGTAAAGAGTTGCGTAGGTGGCAGGGTAAGTAGATAGTGAAATCTGGTGGCTCAACCATTCAGACTATTATCTAAACTGCTCCGCTCGAGAACGTTAGGGGTAGCTGGAATTTCTAGTGTAGGAGTGAAATCCGTAGATATCAGAAAGAACACCGATAGCGTAGGCAGGCTACTGGGACGTTTCTGACACTAAGGCACGAAAGCGTGGGGAGCAAACGGG

>OTU272GTAAGGAATATTGGTCAATGGACGCAAGTCTGAACCAGCCATGCCGCGTGGAGGATGAAGGTCCTCTGGATTGTAAACTTCTTTTATTTGGGAAGAAATCTATCTTTTCTTGGATAGTTGACGGTACCAGATGAATAAGCACCGGCTAACTCCGTGCCAGCAGCCGCGGTAATACGGAGGGTGCAAGCGTTATCCGGATTCACTGGGTTTAAAGGGTGCGTAGGTGGGCAGGTAAGTCAGTGGTGAAATCTCCGGGCTTAACCCGGAAACTGCCATTGATACTATCTGTCTTGAATACCCTGGAGGTGAGCGGAATATGTCATGTAGCGGTGAAATGCTTAGATATGACATAGAACACCCATTGCGAAGGCAGCTCACTACGGGAATATTGACACTGAGGCACGAAAGCGTGGGGATCAAACAGG

>OTU273GTGGGGAATATTGGACAATGGGGGCAACCCTGATCCAGCAATGCCGCGTGTGTGAAGAAGGCCTGCGGGTTGTAAAGCACTTTCAGAAGAGAAGAAAAGCGCAGTGCTAATATCACTGTGTCTTGACGTAACCTTCAGAAGAAGCACCGGCTAACTCCGTGCCAGCAGCCGCGGTAATACGGAGGGTGCAAGCGTTAATCGGAATTACTGGGCGTAAAGCGTGCGTAGGCGGTTGAATTAGTCGGATGTGAAAGCCCCGGGCTTAACCTGGGAATTGCATTCGATACTGTTCGGCTAGAGTGTGGAAGAGGGAAGTGGAATTCCAGGTGTAGCGGTGAAATGCGTAGATATCTGGAGGAACATCAGTGGCGAAGGCGACTTCCTGGTCCAACACTGACGCTGAGGCACGAAAGCGTGGGGAGCAAACAGG

>OTU274GTGGGGGATATTGCACAATGGGGGAAACCCTGATGCAGCGACGCCGCGTGAGCGAAGAAATATTTCGGTATGTAAAGCTCTATCAGCAGGGAAGAAAATGACGGTACCTGACTAAGAAGCCCCGGCTAACTACGTGCCAGCAGCCGCGGTAATACGTAGGGGGCAAGCGTTATCCGGATTTACTGGGTGTAAAGGGAGCGTAGACGGCAAGACAAGCCAGATGTGAAAGGCAGGGGCTCAACCTCTGGATTGCATTTGGAACTGCCTAGCTAGAGTGTCGGAGAGGTAAGTGGAATTCCTAGTGTAGCGGTGAAATGCGTAGATATTAGGAGGAACACCAGTGGCGAAGGCGGCTTACTGGACGACAACTGACGTTGAGGCTCGAAAGCGTGGGGAGCAAACAGG

>OTU275GTGGGGAATATTGCACAATGGGGGAAACCCTGATGCAGCGACGCCGCGTGAGTGAAGAAGTTATTCGTAATGTAAAGCTCTATCAGCAGGGAAGATAATGACGGTACCTGACTAAGAAGCCCCGGCTAACTACGTGCCAGCAGCCGCGGTAATACGTAGGGGGCAAGCGTTATCCGGATTTACTGGGTGTAAAGGGTGCGTAGGTGGTATGGCAAGTCAGAAGTGAAAACCCGGGGCTCAACCCCGTGGATTGCTTTTGAAACTGTCAGACTGGAGTACCGGAGAGGTAAGCGGAATTCCTAGTGTAGCGGTGAAATGCGTAGATATTAGGAGGAACACCAGTGGCGAAGGCGGCTTACTGGACGGTAACTGACACTGAGGCACGAAAGCGTGGGGAGCAAACAGG

>OTU276GTGGGGAATATTGGGCAATGGAGGAAACTCTGACCCAGCGACGCCGCGTGAGGGATGAAGGCCTTCGGGTTGTAAACCTCTTTCAGTAGGGAAGAAGCGAAAGTGACGGTACCTACAGAAGAAGCACCGGCTAACTATGTGCCAGCAGCCGCGGTAATACATAGGGTGCAAGCGTTGTCCGGAATTATTGGGCGTAAAGAGCTCGTAGGTGGTTCGATACGTCGGATGTGAAAATCAGGGGCTCAACCCCTGACCTGCATCCGATACGGTCGAGCTAGAGTTTGGTAGGGGAGACTGGAATTCCTGGTGTAGCGGTGGAATGCGCAGATATCAGGAGGAACACCGATGGCGAAGGCAGGTCTCTGGGCCAATACTGACACTGAGGAGCGAAAGCGTGGGGAGCGAACAGG

>OTU277GTGAGGAATATTGGTCAATGGACGCAAGTCTGAACCAGCCATGCCGCGTGAAGGATGAAGGCCTTCTGGGTTGTAAACTTCTTTTATGTGGGAAGAAACCTCTGATTTCTATTAGAGTTGACGGTACCATAGGAATAAGCACCGGCTAACTCCGTGCCAGCAGCCGCGGTAATACGGAGGGTGCAAGCGTTATCCGGATTTACTGGGTTTAAAGGGTGTGTAGGCGGATCTTTAAGTCAGTGGTGAAATCTCCGAGCTTAACTTGGAAACTGCCATTGATACTATTGATCTTGAATTCTGTTGAGGTGGGCGGAATAAGTCATGTAGCGGTGAAATGCATAGATATGACTTAGAACACCAATTGCGAAGGCAGCTCGCTAAGCAGATATTGACGCTGAGGCACGAAAGCGTGGGGATCAAACAGG

>OTU278GTAGGGAATCTTCCGCAATGGACGCAAGTCTGACGGAGCAACGCCGCGTGAGCGAAGAAGGTTTTCGGATCGTAAAGCTCTGTTGCCGGAGAAGAACGGGCGGGCGAGGAAATGCACCCGTCGTGACGGTAACCGGCCAGAAAGCCACGGCTAACTACGTGCCAGCAGCCGCGGTAATACGTAGGTGGCAAGCGTTGTCCGGAATTATTGGGCGTAAAGCGCGCGCAGGCGGCCCCTTAAGTCTGATGTGAAAGACCGCGGCTTAACCGCGGGGGTGCATTGGAAACTGAGGAGCTTGAGTGCAGAAGAGGAGAGCAGAATTCCACGTGTAGCGGTGAAATGCGTAGAGATGTGGAGGAATACCGGTGGCGAAGGCGGCTCTCTGGTCTGTGACTGACGCTGAGGAGCGAAAGCGTGGGGAGCGAACAGG

>OTU279GTGGGGAATATTGGACAATGGGGGCAACCCTGATCCAGCAATGCCGCGTGAGTGAGGAAGGCCTTAGGGTTGTAAAGCTCTTTCGGCGGGGACGATGATGACGGTACCCGCAGAAGAAGCCCCGGCTAACTTCGTGCCAGCAGCCGCGGTAATACGAAGGGGGCGAGCGTTGTTCGGATTTACTGGGCGTAAAGGGCGCGCAGGCGGCCATCTTTGTCAGGCGTGAAAGCCCCGGGCTCAACCTGGGAGGTGCGCTTGGGACGGGGTGGCTAGAGATCGGGAGAGGAGCGTGGAATTCCCAGTGTAGAGGTGAAATTCGTAGATATTGGGAAGAACACCGGTGGCGAAGGCGGCGCTCTGGACCGAGACTGACGCTGAGGCGCGAAAGCGTGGGGAGCAAACAGG

>OTU280GTGGGGAATATTGCGCAATGGGGGGAACCCTGACGCAGCAACGCCGCGTGCAGGAAGAAGGTCTTCGGATTGTAAACTGTTGTCGCGAGGGAAGAAAAGAATGACGGTACCTCGTGAGAAAGTCACGGCTAACTACGTGCCAGCAGCCGCGGTAATACGTAGGGGGCAAGCGTTATCCGGATTCACTGGGTGTAAAGGGAGCGTAGACGGCAATGCAAGTCAGATGTGAAAGCCCGGGGCTCAACCCCCGGACTGCTCTGGAAACTGTATAACTAGAGTGCAGGAGGGGTAAGTGGAATTCCTAGTGTAGCGGTGAAATGCGTAGATATTAGGAGGAACACCAGTGGCGAAGGCGGCTTTCTGGACGACAACTGACGCTGAGGCGCGAAAGCGTGGGGAGCAAACAGG

>OTU281GTGAGGAATATTGGTCAATGGACGCAAGTCTGAACCAGCCATGCCGCGTGCAGGAAGACGGCTCTATGAGTTGTAAACTGCTTTTGTACGAGGGTAAACGCAGATACGTGTATCTGTCTGAAAGTATCGTACGAATAAGGATCGGCTAACTCCGTGCCAGCAGCCGCGGTAATACGGAGGATTCAAGCGTTATCCGGATTTATTGGGTTTAAAGGGTGCGTAGGCGGTCGGATAAGTTAGAGGTGAAATCCCGAGGCTCAACTTCGGAATTGCCTCTGATACTGTTCGGCTAGAGAGTAGTTGCGGTAGGCGGAATGTATGGTGTAGCGGTGAAATGCTTAGAGATCATACAGAACACCGATTGCGAAGGCAGCTTACCAAGCTACTTCTGACGTTGAGGCACGAAAGCGTGGGGAGCAAACAGG

>OTU282GTAGGGAATCTTCCGCAATGGACGCAAGTCTGACGGAGCAACGCCGCGTGAGCGAAGAAGGTTTTCGGATCGTAAAGCTCTGTTGCCGGAGAAGAACGGGAGGGAGAGGCAATGCTCCCTTCGTGACGGTATCCGGCCAGAAAGCCACGGCTAACTACGTGCCAGCAGCCGCGGTAATACGTAGGTGGCAAGCGTTGTCCGGAATTATTGGGCGTAAAGCGCGCGCAGGCGGCTTCTTAAGTCTGATGTGAAAGGCTGCGGCTCAACCGCAGACGGTCATTGGAAACTGGGGAGCTTGAGGGCAGAAGAGGAGAGTAGAATTCCACGTGTAGCGGTGAAATGCGTAGAGATGTGGAGGAATACCGGTGGCGAAGGCGGCTCTCTGGTCTGCAACTGACGCTGAGGCGCGAAAGCGTGGGGAGCAAACAGG

>OTU283GTGGGGAATTTTTCGCAATGGGGGCAACCCTGACGAAGCAATGCCGCGTGGATGAAGAAGGCCTTCGGGTTGTAAAGTCCTTTCGTGGAGGACGAAAAGGTGGGTTCTAATACAATCTGCTATTGACGTGAATCCAAGAAGAAGCACCGGCTAACTCCGTGCCAGCAGCCGCGGTAATACGGGGGGTGCAAGCGTTAATCGGAATCACTGGGCGTAAAGGGTGCGTAGGCGGTACGTTAGGTCTGTCGTGAAATCCCCGGGCTCAACCTGGGAATGGCGGTGGAAACCGGTGTACTAGAGTATGGGAGAGGGTGGTGGAATTCCAGGTGTAGCGGTGAAATGCGTAGAGATCTGGAGGAACATCAGTGGCGAAGGCGGCCACCTGGCCCAATACTGACGCTGAGGCACGAAAGCGTGGGGAGCAAACAGG

>OTU284GTGGGGAATATTGCACAATGGGGGAAACCCTGATGCAGCCATGCCGCGTGTGTGAAGAAGGCTTTCGGGTTGTAAAGCACTTTAAGTTGTGAGGAAAGGTTGGAGATTAATACTCTCTAGCTATGACGTTAACAACAGAATAAGCACCGGCTAACTCCGTGCCAGCAGCCGCGGTAATACGGAGGGTGCAAGCGTTAATCGGAATTACTGGGCGTAAAGCGCGCGTAGGCGGCTATTTAAGCTAGATGTGAAAGCCCAGGGCTTAACCTTGGAACTGCATTTAGAACTGGGTAGCTAGAGTACAGCAGAGGATAGTGGAATTTCAGGTGTAGCGGTGAAATGCGTAGAGATCTGAAGGAACATCAGTGGCGAAGGCGACTGTCTGGGCTGATACTGACGCTGAGGTGCGAAAGCGTGGGGAGCAAACAGG

>OTU285GTGGGGAATCTTGCGCAATGGCCGAAAGGCTGACGCAGCGACGCCGCGTGGGGGATGAAGGTTTTCGGATCGTAAACCCCTGTTGCCCGGGACGAACTTCCTCTTTAGAGAGGATTGACGGTACCGGGTGAGGAAGCACCGGCTAACTCCGTGCCAGCAGCCGCGGTAATACGGAGGGTGCGAGCGTTGTCCGGAATCACTGGGCGTAAAGGGCGCGTAGGCGGTTGGGTAAGCGTGCGGTGAAAGCTCGGGGCTTAACCCCGAGTCGGCCGTGCGAACTGCTCGACTCGAGCACTGTAGAGGCAGGTGGAATTCCGGGTGTAGCGGTGGAATGCGTAGAGATCCGGAAGAACACCGGTGGCGAAGGCGGCCTGCTGGGCAGTGGCTGACGCTGAGGCGCGACAGCGTGGGGAGCAAACAGG

>OTU286GTGAGGAATATTGGACAATGGTCGGAAGACTGATCCAGCCATGCCGCGTGAAGGATAAGGTCCTATGGATTGTAAACTTCTTTTGTACGAGAGGAATAAGGTATATGAATATATCGATGACAGTATCGTACGAATAAGCATCGGCTAACTCCGTGCCAGCAGCCGCGGTAATACGGAGGATGCAAGCGTTATCCGGATTTATTGGGTTTAAAGGGTGCGTAGGCGGCCTTGTAAGTCAGCGGTGAAATTTTGCAGCTTAACTGTAAAAGTGCCGTTGATACTGTGAGGCTAGGATTTGGTTGCTGTGGGTGGAATGTGTGGTGTAGCGGTGAAATGCTTAGAGATCACACAGAATATCGATTGCGAAGGCAGCTCACAAAGCCAAGATTGACGCTGAGGCACGAAAGTGTGGGGATCAAACAGG

>OTU287GTGGGGAATATTGCGCAATGGAGGGAACTCTGACGCAGCAACGCCGCGTGAAGGATGAAGGTTTTCGGATTGTAAACTTCTTTGATCGGGGACGAAAATGACGGTACCCGAAGAACAAGCCACGGCTAACTACGTGCCAGCAGCCGCGGTAATACGTAGGTGGCAAGCGTTGTCCGGAATTACTGGGTGTAAAGGGCGTGTAGGCGGGAAAGCAAGTCAGATGTGAAAGCCCATGGCTTAACTGTGGAATTGCATCTGAAACTGTTTTTCTTGAGTACTGGAGAGGAAAGTGGAATTCCTAGTGTAGCGGTGAAATGCGTAGAGATTAGGAGGAACACCAGTGGCGAAGGCGGCTTTCTGGACAGTAACTGACGCTGAGGCGCGAAAGCGTGGGGAGCAAACAGG

>OTU288GTAGGGAATCTTCCACAATGGACGCAAGTCTGATGGAGCAACGCCGCGTGAGTGAAGAAGGGTTTCGGCTCGTAAAGCTCTGTTGTTAAAGAAGAACGTGGGTAAGAGTAACTGTTTACCCAGTGACGGTATTTAACCAGAAAGCCACGGCTAACTACGTGCCAGCAGCCGCGGTAATACGTAGGTGGCAAGCGTTATCCGGATTTATTGGGCGTAAAGCGAGCGCAGGCGGTCTTTTAAGTCTAATGTGAAAGCCTTCGGCTCAACCGAAGAAGTGCATTGGAAACTGGGAGACTTGAGTGCAGAAGAGGACAGTGGAACTCCATGTGTAGCGGTGAAATGCGTAGATATATGGAAGAACACCAGTGGCGAAGGCGGCTGTCTGGTCTGCAACTGACGCTGAGGCTCGAAAGCATGGGTAGCGAACAGG

>OTU289GTCGAGAATCTTCCGCAATGGACGAAAGTCTGACGGAGCGACGCCGCGTGGAGGACGAAGGCATTAGTGTTGTAAACTCCTGTCACGAATTATGAATGTGCAGGCGATAATCTCGCCTGCATTGACGTAAGTTCGAGAGGAAGCACCGGCTAACTCTGTGCCAGCAGCCGCGGTAATACAGAGGGTGCGAACGTTGTTCGGAATTACTGGGCATAAAGCGCGCGTAGGCGGTCCGCTAAGTGTGGAGTGAAAGCCCGCGGCTTACCCGTGGAATGGCTCTGCATACTGGCGAACTAGAGGATGGTAGGGGAGAGCGGAACTCTTGGTGGAGCGGTGAAATGCGTAGATATCAAGAGGAACACCGATGGCGAAGGCAGCTCTCTGGTCCATTTCTGACGCTGAGGCGCGAAAGCTAGGGTAGCAAACGGG

>OTU290GTGAGGAATATTGCGCAATGGGGGAAACCCTGACGCAGCGACGCCGCGTGAGTGAGGAAGGCCTTCGGGTCGTAAAGCTCTGTCAAAGGGAAAGAAGTGCATGATGGCTAATACCTGTCATGTTTGACGGTACCCTTAAAGGAAGCACCGGCTAACTCCGTGCCAGCAGCCGCGGTAATACGGAGGGTGCAAGCGTTGTTCGGAATCACTGGGCGTAAAGGGCGCGTAGGCGGTTTGATAAGTCAGATGTGAAAGCCCACGGCTTAACTGTGGAAGTGCATTTGAAACTGTCAGACTTGAGTATCAGAGGGGAAAGTGGAATTCCCGGTGTAGAGGTGAAATTCGTAGATATCGGGAGGAATACCGGTGGCGAAGGCGACTTTCTGGCTGAATACTGACGCTGAGGCGCGAAAGCGTGGGGAGCAAACAGG

>OTU291GTGAGGAATATTGGTCAATGGGAGAGATCCTGAACCAGCCAAGCCGCGTGAGGGAAGACGGCCCTATGGGTTGTAAACCTCTTTTGTCGGAGAACAAAACCCGGGACGTGTCCCGGACTGCGTGTATCCGAAGAAAAAGCATCGGCTAACTCCGTGCCAGCAGCCGCGGTAATACGGAGGATGCGAGCGTTATCCGGATTTATTGGGTTTAAAGGGTGCGTAGGCGGTCCGTTAAGTCAGCGGTAAAATTGCGGGGCTCAACCCCGTCGAGCCGTTGAAACTGGCAGACTTGAGTTGGCGAGAAGTACGCGGAATGCGCGGTGTAGCGGTGAAATGCATAGATATCGCGCAGAACTCCGATTGCGAAGGCAGCGTACCGGCGCCAGACTGACGCTGAGGCACGAAAGCGTGGGGATCGAACAGG

>OTU292GCAAGGAATTTTCGTCAATGGGCGCAAGCCTGAACGAGCAACGCCGCGTGCAGGATGACGGCCTTCGGGTTGTAAACTGCTTTTCAGGGGGAAGACATCGACGGTACCCCTGGAATAAGGCTCGGCTAACTCTGTGCCAGCAGCCGCGGTAAGACAGAGGAGCCAAGCGTTGTCCGGATTGACTGGGCGTAAAGCGCACGCAGGTGGTTGCGGGCGTCGGTGCTGAAAGTTCGCCGCTTAACGGCGAATCGTGTGCCGATACGCCGCGACTTGAGGCCGTGAGAGGTCCGTGGAATTGCCGGTGTAGTGGTGAAATGCGTAGAGATCGGCAGGAACACCCAAGGAGAAATCAGCGGACTGGCGCGGTTCTGACACTGAGGTGCGAAAGCGTGGGGAGCGAACTGG

>OTU293GTGGGGGATATTGGACAATGGGGGGAACCCTGATCCAGCGACGCCGCGTGAGTGAAGAAGTATCTCGGTATGTAAAGCCCTATCAGCAGGGAAGAAAAATGACGGTACCTGACTAAGAAGCACCGGCTAAATACGTGCCAGCAGCCGCGGTAATACGTATGGTGCAAGCGTTATCCGGATTTACTGGGTGTAAAGGGAGCGCAGGCGGCACGCTAAGCCTGATGTGAAAACCCGCGGCCCAACTGTGGGACTGCATTGGGAACTGGCGCGCTGGAGTGCCGGAGGGGTAGGCGGAATTCCTGGTGTAGCGGTGAAATGCGTAGATATCAGGAGGAACACCAGTGGCGAAGGCGGCCTACTGGACGGTGACTGACGCTGAGGCTCGAAAGCGTGGGGAGCAAACAGG

>OTU294GTGGGGAATATTGGACAATGGGCGAAAGCCTGATCCAGCCATGCCGCGTGTGTGAAGAAGGTCTTAGGATTGTAAAGCACTTTAAGTTGGGAGGAAGAGCAGTTAACTAATATTTGACTGTTTTGACGTTACCAACAGAATAAGCACCGGCTAACTTCGTGCCAGCAGCCGCGGTAATACGAAGGGTGCAAGCGTTAATCGGAATTACTGGGCGTAAAGCGCGCGTAGGTGGTTCAGTAAGTTAGGAGTGAAAGCCCCGGGCTTAACCTGGGAATTGCTTCTAAAACTGCTGAGCTAGAGTACGGTAGAGGGTGGTGGAATTTCCTGTGTAGCGGTGAAATGCGTAGATATAGGAAGGAACATCAGTGGCGAAGGCGACCACCTGGACTGATACTGACACTGAGGTGCGAAAGCGTGGGGAGCAAACAGG

>OTU295GTGGGGAATTTTGGACAATGGGCGAAAGCCTGATCCAGCCATGCCGCGTGCGGGAAGAAGGCCTTCGGGTTGTAAACCGCTTTTGGCAGGAACGAAACGGCGCGGGCCAATACCCCGCGCTAATGACGGTACCTGCAGAATAAGCACCGGCTAACTACGTGCCAGCAGCCGCGGTAATACGTAGGGTGCAAGCGTTAATCGGAATTACTGGGCGTAAAGCGTACGCAGGCGGCTTTGCAAGACAGGTGTGAAATCCCCGGGCCTAACCTGGGAACTGCATTTGTGACTGCATGGCTAGAGTCTGTCAGAGGGGGGTGGAATTCCACGTGTAGCAGTGAAATGCGTAGAGATGTGGAAGAACACCGATGGCGAAGGCAGCCCCCTGGGATGAGACTGACGCTCATGTACGAAAGCGTGGGGAGCAAACAGG

>OTU296GTGGGGAATATTGGGCAATGGGCGCAAGCCTGACCCAGCCACGCCGCGTGAGTGATGAAGGCTTTCGGGTCGTAAAGCTCTGTGGGGAGGGACGAAAGCCCTTTGGCTAATATCCAGAGGTTTGACGGTACCTCCTTAGCAAGCACCGGCTAACTCCGTGCCAGCAGCCGCGGTAATACGGGGGGTGCAAACGTTGCTCGGAATTATTGGGCGTAAAGCGCACGTAGGCGGTCCGCTAAGTCGGGTGTGAAAGCCCTCGGCTTAACCGAGGAAGTGCATTCGAAACTGGCGGACTTGAGTACCGGAGAGGGTGGCGGAATTCCCGGTGTAGAGGTGAAATTCGTAGATATCGGGAGGAACACCAGCGGCGAAGGCGGCCATCTGGACGGATACTGACGCTGAGGTGCGAAAGCGTGGGGAGCAAACAGG

>OTU297GTGAGGAATATTGGTCAATGGGCGAGAGCCTGAACCAGCCAAGTCGCGTGAGGGACGACTGCCCTATGGGTTGTAAACCTCTTTTGTATGGGGGGAATAAGCTTTACGAGTAGAGTGATGACAGTACCATACGAATAAGCATCGGCTAACTCCGTGCCAGCAGCCGCGGTAATACGGAGGATGCGAGCGTTATCCGGATTTATTGGGTTTAAAGGGTGCGTAGGCGGCATTATAAGTTAGTGGTAAAATATTGGGGCTTCACCTCACTACGCCATTAATACTGTAGAGCTAGAGAACAGACGAGGTAGGCGGAATAAGTTAAGTAGCGGTGAAATGCATAGATATAACTTAGAACACCGATAGCGAAGGCAGCTTACCAGACTGAGTCTGACGCTGATGCACGAGAGCATGGGTAGCGAACAGG

>OTU298GTGGGGGATATTGGACAATGGGGGAAACCCTGATCCAGCGATGCCGCGTGAGGGAAGAAGGTTTTCGGATTGTAAACCTCTGTGGAGGGGGACGATAATGACGGTACCCCTTTAGGAAGCCACGGCTAACTACGTGCCAGCAGCCGCGGTAATACGTAGGTGGCGAGCGTTGTCCGGAATTACTGGGTGTAAAGGGAGTGTAGGCGGGAAAGCAAGTCAGAAGTGAAAACTATGGGCTTAACCCATAGCCTGCTTTTGAAACTGTTTTTCTTGAGTGAAGTAGAGGCAAGCGGAATTCCCAGTGTAGCGGTGAAATGCGTAGATATTGGGAGGAACACCAGTGGCGAAGGCGGCTTGCTGGGCTTTTACTGACGCTGAGGCTCGAAAGCGTGGGGAGCAAACAGG

>OTU299GTAGGGAATTTTCGGCAATGGGCGAAAGCCTGACCGAGCAACGCCGCGTGAAGGAAGAAGGAATTCGTTTTGTAAACTTCTGTCATAGAGGAAGAACGGTATGATCAGGGAATGGGTCATAAGTGACGGTACTCTATAAGAAAGCCACGGCTAACTACGTGCCAGCAGCCGCGGTAATACGTAGGTGGCGAGCGTTATCCGGAATTATTGGGCGTAAAGAGGGAGCAGGCGGCACTAAAGGTCTGTGGTGAAAGCCCGAAGCTTAACTTCGGTAAGCCATGGAAACCGTAGAGCTAGAGTGTGTGAGAGGATCGTGGAATTCCATGTGTAGCGGTGAAATGCGTAGATATATGGAGGAACACCAGTGGCGAAGGCGACGATCTGGCGCACAACTGACGCTCAGTCCCGAAAGCGTGGGGAGCAAATAGG

>OTU300GTGGGGAATATTGGACAATGGGCGCAAGCCTGATCCAGCCATGCCGCGTGAGTGATGAAGGCCTTAGGGTTGTAAAGCTCTTTCGGCGGGGACGATAATGACGGTACCCGCAGAAGAAGCCCCGGCTAACTTCGTGCCAGCAGCCGCGGTAATACGAAGGGGGCTAGCGTTGCTCGGAATCACTGGGCGTAAAGCGCACGTAGGCGGGTTGCCAAGTCGGGGGTGAAATCCTGGAGCTCAACTCCAGAACTGCCTTCGATACTGGCAATCTTGGAGTCCGGGAGAGGTGAGTGGAACTGCGAGTGTAGAGGTGAAATTCGTAGATATTCGCAAGAACACCAGTGGCGAAGGCGGCTCACTGGCCCGGTACTGACGCTGAGGTGCGAAAGCGTGGGGAGCAAACAGG

>OTU301GTGGGGAATTGTTCGCAATGGGCGCAAGCCTGACGACGCAACGCCGCGTGGAGGATGAAGACTTTCGGGTCGTAAACTCCTTTCGACCGAGATGAATTTCCGCCGACCCAACACGTCGGTGGAGTGACAGTATCGAGGGAAGAAGCCCCGGCTAACTCCGTGCCAGCAGCCGCGGTAATACGGGGGGGGCAAGCGTTGTTCGGAATTACTGGGCGTAAAGGGTTCGTAGGTGGCCAACTAAGTCAGACGTGAAATCCCTCAGCTTAACTGGGGAACTGCGTCTGATACTGATTGGCTTGAGTGCAGGAGAGGAACGCGGAATTCCAGGTGTAGCGGTGAAATGCGTAGATATCTGGAGGAACACCGGTGGCGAAGGCGGCGTTCTGGACTGCAACTGACACTGAGGAACGAAAGCCAGGGGAGCAAACGGG

>OTU302GTGGGGAATATTGCACAATGGGCGAAAGCCTGATGCAGCCATGCCGCGTGTGTGAAGAAGGCTCTAGGGTTGTAAAGCACTTTCATTAGTGAGGAAAGGTTGTAGCTTAATACGTTGCAACTGTGACGTTAGCTAAAGAAGAAGCACCGGCTAACTCCGTGCCAGCAGCCGCGGTAATACGGAGGGTGCAAGCGTTAATCGGAATTACTGGGCGTAAAGCGCGCGTAGGTGGTTTGTTAAGCGGAATGTGAAAGCCCCGGGCTTAACCTGGGAACTGCATTGCGAACTGGCAAACTAGAGTACAGTAGAGGGTAGTGGAATTTCCTGTGTAGCGGTGAAATGCGTAGAGATGGGAAGGAACATCAGTGGCGAAGGCGACTGCCTGGACTGATACTGACACTGAGGTGCGAAAGCGTGGGGAGCAAACAGG

>OTU303GTGAGGAATATTGGTCAATGGGCGCGAGCCTGAACCAGCCAAGTAGCGTGAAGGATGAAGGTCCTAGGGATTGTAAACTTCTTTTATAAGGGAATAAAGTCACCCACGTGTGGGTGTTTGTATGTACCTTATGAATAAGCATCGGCTAACTCCGTGCCAGCAGCCGCGGTAATACGGAGGATGCGAGCGTTATCCGGATTTATTGGGTTTAAAGGGAGCGTAGACGGGTCGTTAAGTCAGCTGTGAAAGTTTGAGGCTCAACCTTAAAATTGCAGTTGATACTGGCGTCCTTGAGTACGGTTGAGGCAGGCGGAATTCGTGGTGTAGCGGTGAAATGCTTAGATATCACGAAGAACCCCGATTGCGAAGGCAGCCTGCTAAGCCGCGACTGACGTTGAGGCTCGAAAGTGTGGGTATCAAACAGG

>OTU304GTGGGGAATATTGCACAATGGGGGAAACCCTGATGCAGCGACGCCGCGTGAAGGAAGACGTATTTCGGTATGTAAACTTCTATCAGCAAGGAAGATGATGACGGTACTTGACTAAGAAGCCCCGGCTAACTACGTGCCAGCAGCCGCGGTAATACGTAGGGGGCAAGCGTTATCCGGATTTACTGGGTGTAAAGGGAGCGTAGGCGGCCATGCAAGTCAGAAGTGAAAGCCCGGGGCTCAACCCCGGGACTGCTTTTGAAACTGTAGGGCTAGATTGCCGGAGAGGTGAGCGGAATTCCTAGTGTAGCGGTGAAATGCGTAGATATTAGGAGGAACACCAGTGGCGAAGGCGGCTCACTGGACGGTAAATGACGCTGAGGCTCGAAAGCGTGGGGAGCAAACAGG

>OTU305GTGAGGAATATTGGTCAATGGACGAGAGTCTGAACCAGCCAAGTAGCGTGAAGGATGACTGCCCTATGGGTTGTAAACTTCTTTTATACGGGAATAAAGTGGAGTATGCATACTCCTTTGTATGTACCGTATGAATAAGGATCGGCTAACTCCGTGCCAGCAGCCGCGGTAATACGGAGGATCCGAGCGTTATCCGGATTTATTGGGTTTAAAGGGAGCGTAGGCGGGTGCTTAAGTCAGTTGTGAAAGTTTGCGGCTCAACCGTAAAATTGCAGTTGATACTGGGCGCCTTGAGTGCAGCATAGGTAGGCGGAATTCGTGGTGTAGCGGTGAAATGCTTAGATATCACGAAGAACTCCGATTGCGAAGGCAGCTTACTGGACTGTAACTGACGCTGATGCTCGAAAGTGTGGGTATCAAACAGG

>OTU306GTCGAGGATCTTCCGCAATGGGCGAAAGCCTGACGGAGCGACACCGCGTGCAGGATGAAGGCCTTCGGGTTGTAAACTGCTGTCACATTCTAGGAATGGCGCGTGATGAACAGTCACGCGCTTGACTAAGGTGAAAGGAAGCACCGGCTAACTCCGTGCCAGCAGCCGCGGTAAGACGGAGGGTGCAAACGTTGTTCGGAATCACTGGGCATAAAGCGCACGTAGGCGGTGCACTAAGTCAGGTGTGAAAGCCCCCGGCTCAACCGGGGAATGGCATCTGATACTGGTGTGCTCGAGGACGGTTGGGGTGAGTGGAACTCCTGGTGGAGCGGTGAAATGCGTAGATATCAGGAGGAACACCGGTGGCGAAAGCGGCTCACTGGGCCGTCTCTGACGCTGAGGTGCGAAAGCTAGGGTAGCGAACGGG

>OTU307GTCGAGAATCTTCGGCAATGGGCGCAAGCCTGACCGAGCGACGCCGCGTGGAGGATGAAGGCCTTCGGGTTGTAAACTCCTGTCGAGGGGGAAGAAGGCCCCGCGAGGGGCTTGACTGATCCCTGGAGGAAGCACGGGCTAAGTTCGTGCCAGCAGCCGCGGTAAGACGAACCGTGCGAACGTTATTCGGAATCACTGGGCTTAAAGCGCGTGTAGGCGGGACGGCACGTCGGTCGCTGAAATCCCCCGGCTCAACCGGGGAACGGGCGCCGATACGACCGCCCTGGAGGGACGTAGGGGGACCTGGAACTTCCGGTGGAGCGGTGAAATGCGTTGAGATCGGAAGGAACGCCCGTGGCGAAAGCGAGGTCCTGGACGTTTTCTGACGCTGAGACGCGAAAGCCAGGGGAGCGAACGGG

>OTU308GTGAGGAATTTTGGACAATGGGGGAAACCCTGATCCAGCGACGCCGCGTGGAGGACGAAGGTCTTCGGATTGTAAACTCCTTTTGTTTGGGACTAAGAAACCTGTAGAAATACGGGGAGTGAATGTACCAGATGAATAAGCCACGGCTAACTTCGTGCCAGCAGCCGCGGTAATACGAAGGTGGCAAGCGTTACTCGGAATTACTGGGTTTAAAGGGTGCGTAGGCGGCGCAGTAAGTCAGGAGTGAAAGTTTGCGGCTCAACCGTAAAATTGCTTTTGATACTGCTGTGCTGGAATTAGGATGAGGTCAGCGGAATGTGGCATGTAGCGGTGAAATGCATAGATATGCCATAGAACACCAATTGCGAAGGCAGCTGGCTAGACCTGGATTGACGCTGAGGCACGAAAGCGTGGGGAGCGAACAGG

>OTU309GTCGAGAATCATTCACAATGGGGGAAACCCTGATGGTGCGACGCCGCGTGGGGGAATGAAGGTCTTCGGATTGTAAACCCCTGTCATGTGGGAGCAAATTAAAAAGATAGTACCACAAGAGGAAGAGACGGCTAACTCTGTGCCAGCAGCCGCGGTAATACAGAGGTCTCAAGCGTTGTTCGGAATCACTGGGCGTAAAGCGTGCGTAGGCTGTTTCGTAAGTCGTGTGTGAAAGGCGCGGGCTCAACCCGCGGACGGCACATGATACTGCGAGACTAGAGTAATGGAGGGGGAACCGGAATTCTCGGTGTAGCAGTGAAATGCGTAGATATCGAGAGGAACACTCGTGGCGAAGGCGGGTTCCTGGACATTAACTGACGCTGAGGCACGAAGGCCAGGGGAGCGAAAGGG

>OTU310GTAGGGAATCTTTCACAATGGGCGAAAGCCTGATGGAGCAACGCCGCGTGCAGGATGAAGGCCTTCGGGTTGTAAACTGCTTTTATAAGCGAGAAATATGATGGTAACTTATGAATAAGGATCGGCTAACTACGTGCCAGCAGCCGCGGTCATACGTAGGATCCGAGCATTATCCGGAGTGACTGGGTGTAAAGAGTTGCGTAGGTGGCATAGTAAGTAGCTAGTGAAATCTGGTGGCTCAACCATTCAGACTATTAGCTAAACTGCTAAGCTCGAGACCGTTAGGGGTAACTGGAATTTCTAGTGTAGGAGTGAAATCCGTAGATATTAGAAGGAACACCGATAGCGTAGGCAGGTTACTGGGACGGTTCTGACACTAAGGCACGAAAGCGTAGGGAGCAAACGGG

>OTU311GTAGGGAATCTTCCACAATGGACGAAAGTCTGATGGAGCAACGCCGCGTGAGTGAAGAAGGTTTTAGGATCGTAAAACTCTGTTGTTGGAGAAGAACAGGGACTAGAGTAACTGTTAGTCCTTTGACGGTATCCAACCAGAAAGCCACGGCTAACTACGTGCCAGCAGCCGCGGTAATACGTAGGTGGCAAGCGTTGTCCGGATTTATTGGGCGTAAAGCGAGCGCAGGCGGTTTTTTAAGTCTGATGTGAAAGCCTTCGGCTTAACCGAAGAAGTGCATTAGAAACTGGGAAACTTGAGTGCAGAAGAGGACAGTGGAACTCCATGTGTAGCGGTGAAATGCGTAGATATATGGAAGAACACCAGTGGCGAAGGCGGCTGTCTGGTCTGTAACTGACGCTGAGGCTCGAAAGTATGGGGAGCGAACAGG

>OTU312GTGAGGAATATTGGTCAATGGGCGAGAGCCTGAACCAGCCAAGTCGCGTGAGGGAAGACGGTCCTATGGATTGTAAACCTCTTTTGTCGGGGAGCAAAAGACGCCACGCGTGGCGTTCCGAGAGTACCCGAAGAAAAAGCATCGGCTAACTCCGTGCCAGCAGCCGCGGTAATACGGAGGATGCGAGCGTTATCCGGATTTATTGGGTTTAAAGGGTGCGCAGGCGGAAGATCAAGTCAGCGGTAAAATTGAGAGGCTCAACCTCTTCGAGCCGTTGAAACTGGTTTTCTTGAGTGAGCGAGAAGTATGCGGAATTCGTGGTGTAGCGGTGAAATGCATAGATATCACGAAGAACCCCGATTGCGAAGGCAGCATACCGGCTCCACACTGACGCTGAGGCACGAAAGCGTGGGTATCGAACAGG

>OTU313GTAGGGAATCTTCCACAATGGGCGCAAGCCTGATGGAGCAACACCGCGTGAGTGAAGAAGGGTTTCGGCTCGTAAAGCTCTGTTGTTAGAGAAGAACGTGCGTGAGAGCAACTGTTCACGCAGTGACGGTATCTAACCAGAAAGTCACGGCTAACTACGTGCCAGCAGCCGCGGTAATACGTAGGTGGCAAGCGTTATCCGGATTTATTGGGCGTAAAGCGAGCGCAGGCGGTTTGATAAGTCTGATGTGAAAGCCTTTGGCTTAACCAAAGAAGTGCATCGGAAACTGTCAGACTTGAGTGCAGAAGAGGGCAGTGGAACTCCATGTGTAGCGGTGGAATGCGTAGATATATGGAAGAACACCAGTGGCGAAGGCGGCTGCCTGGTCTGCAACTGACGCTGAGGCTCGAAAGCATGGGTAGCGAACAGG

>OTU314GTGAGGAATATTGGTCAATGGGCGCGAGCCTGAACCAGCCAAGTAGCGTGCAGGACGACGGCCCTATGGGTTGTAAACTGCTTTTATACGGGGATAAAGTATGCCACGTGTGGTTTATTGCAGGTACCATATGAATAAGGACCGGCTAATTCCGTGCCAGCAGCCGCGGTAATACGGAAGGTCCGGGCGTTATCCGGATTTATTGGGTTTAAAGGGAGCGTAGGCCGGGTTTTAAGCGTGCCGTGAAATGTCGGGGCTCAACCTTGACACTGCGGCGCGAACTGGAGTCCTTGAGTGCGCGGAACGTATGCGGAATTCGTGGTGTAGCGGTGAAATGCTTAGATATCACGAAGAACCCCGATTGCGAAGGCAGCATACGGCAGCGCTACTGACGCTGAAGCTCGAAGGCGCGGGTATCGAACAGG

>OTU315GTGGGGAATTTTGCGCAATGGGCGAAAGCCTGACGCAGCGACGCCGCGTGGAGGATGAAGGTTTTCGGATTGTAAACTCCTTTTGTAGGGGAAAATAATGATGGTACCCTACGAATAAGCCACGGCTAACTCTGTGCCAGCAGCCGCGGTAAGACAGAGGTGGCAAGCGTTGTTCGGAATTACTGGGCTTAAAGGGCGCGTAGGTGGTGGCATAAGTCCGAGGTGGAAGACTAAGGCTTAACCTTAGAATTGCCTTGGAAACTGTATCACTTGAGTCAGCGAGGGGATGGCGGAATTCCAGGTGTAGCGGTGAAATGCGTAGATATCTGGAGGAAGGCCGGTGGCGAAGGCGGCCATCTGGCGCTGAACTGACACTGAGGCGCGAAAGCGTGGGGAGCAAACAGG

>OTU316GTGAGGAATATTGGTCAATGGCCGGAAGGCTGAACCAGCCAAGCCGCGTGGAGGATGAAGGCGCTAAGCGTCGTAAACTTCTTTTGTTGAGGGACAAAATCCGTCACGAGTGACGGACTGAGGGTACTCAAAGAAAAAGCATCGGCTAACTCCGTGCCAGCAGCCGCGGTAATACGGAGGATGCGAGCGTTATCCGGATTTATTGGGTTTAAAGGGTGCGTAGGCGGACTGTTAAGTCAGCGGTAAAAGCCCGGAGCTCAACTCCGGCGAGCCGTTGAAACTGGTGGTCTTGAGTTGGAGAGAAGTATGCGGAATGCGCAGTGTAGCGGTGAAATGCATAGATATTGCGCAGAACTCCGATTGCGAAGGCAGCATACCGGTTCCATACTGACGCTGAGGCACGAAAGCGTGGGGATCAAACAGG

>OTU317GTGAGGAATATTGGTCAATGGACGCAAGTCTGAACCAGCCACGTCGCGTGAAGGAAGACGGCCCTACGGGTTGTAAACTTCTTTTATACGGGAATAAAGTGCCCCACGCGTGGGGTTTTGCATGTACCGTATGAATAAGGATCGGCTAACTCCGTGCCAGCAGCCGCGGTAATACGGAGGATCCGAGCGTTATCCGGATTTATTGGGTTTAAAGGGTGCTCAGGCGGGGGATTAAGTCGGCGGTGAAATTTTGCAGCTCAACTGTAAAAGTGCCTTCGAAACTGGTTTCCTTGAGTGTGGATGAGGTAGGCGGAATTTGTGGTGTAGCGGTGAAATGCATAGATATCACGAGGAACTCCGATTGCGCAGGCAGCTTACTAAACCATAACTGACGCTCAAGCACGAAGGCGTGGGGATCAAACAGG

>OTU318GTTAGGAATTTTGCGCAATGGGAGAAATCCTGACGCAGCAACGCCGCGTGAGGGATGAAGGCCTTCGGGTTGTAAACCTCTTTTAGTGGGGAAAAACACAATGATGGTACCCGCAGAAAAAGCACCGGCGAACTCTGTGCCAGCAGCCGCGGTAATACAGAGGGTGCGAGCGTTATTCGGATTCACTGGGCGTAAAGGGCGCGTAGGCGGCTTGGTAAGTCTGATGTGAAATCCCTGGGCTCAATCCAGGAAGTGCATCGGATACTGCCAGGCTGGAGTATGGGAGAGGAGGACGGAATTCCTGGTGTAGAGGTGAAATTCGTAGATATTGGGAAGAACACCGGTGGCGAAGGCGGCCATCTGGCCCGCAATTGACGCTGAGGCGCGAAAGCGTGGGGAGCAAACAGG

>OTU319GTAGGGAATATTGGTCAATGGGTGAGAGCCTGAACCAGCCATGCCGCGTGCAGGAAGAAGGCCTTCTGGGTTGTAAACTGCTTTTGCCAGGGGATAAAATAGCTTTGCGAAGCAAATTGAAGGTACCTGGTGAATAAGCCACGGCTAACTACGTGCCAGCAGCCGCGGTAATACGTAGGTGGCAAGCGTTGTCCGGATTTATTGGGTTTAAAGGGTGCGTAGGCGGTTCCATAAGTCAGTGGTGAAATACAGCCGCTTAACGGTTGAGGTGCCATTGATACTGCGGAACTTGAGTACAGACGAGGTAGGCGGAATTGACGGTGTAGCGGTGAAATGCTTAGATATCGTCAAGAACACCGATAGCGAAGGCAGCTTACTAGACTGTAACTGACGCTGAGGCACGAAAGTGTGGGGATCAAACAGG

>OTU320GTGGGGGATATTGCACAATGGAGGAAACTCTGATGCAGCAACGCCGCGTGAGGGAAGAAGGTTTTCGGATTGTAAACCTCTGTCCTTGGTGACGATAATGACGGTAGCCAAGGAGGAAGCTCCGGCTAACTACGTGCCAGCAGCCGCGGTAATACGTAGGGAGCAAGCGTTGTCCGGATTTACTGGGTGTAAAGGGTGCGTAGGCGGCACTGCAAGTCAGGCGTGAAAGGCGGAGGCTTAACCTCCGAGTTGCGTTTGAAACTGTGGTGCTTGAGTGAAGTAGAGGTAGGCGGAATTCCCGGTGTAGCGGTGAAATGCGTAGAGATCGGGAGGAACACCAGTGGCGAAGGCGGCCTACTGGGCTTTAACTGACGCTGAGGCACGAAAGCATGGGTAGCAAACAGG

>OTU321GTGAGGAATATTGGACAATGGATGGAAATCTGATCCAGCCATGCCGCGTGAAGGACGACGGTCCTATGGATTGTAAACTTCTTTTGTATGGGAGCAATAAGGTCTACGTGTAGATCGATGAGAGTACCATACGAATAAGCATCGGCTAACTCCGTGCCAGCAGCCGCGGTAATACGGAGGATGCGAGCGTTATCCGGATTTATTGGGTTTAAAGGGTGCGTAGGCGGTTGTATAAGTCAGCGGTGAAATTTTCCGGCTTAACCGGGAGACTGCCGTTGATACTGTATAGCTTGAATACGGTTGCTGTGAGTGGAATGTGTGGTGTAGCGGTGAAATGCATAGATATCACACAGAATATCGATTGCGAAGGCAGCTCACAAAGCCGTTATTGACGCTGATGCACGAAAGTGTGGGGATCAAACAGG

>OTU322GTGGGGAATATTGCACAATGGGGGAAACCCTGATGCAGCCATGCCGCGTGTGTGAAGAAGGCCTTCGGGTTGTAAAGCACTTTCAGTAGGGAGGAAAGGTTGTAAGTTAATACCTTGCAGCTGTGACGTTACCTACAGAAGAAGGACCGGCTAACTCCGTGCCAGCAGCCGCGGTAATACGGAGGGTCCAAGCGTTAATCGGAATTACTGGGCGTAAAGCGTGCGCAGGCGGTTTGTTAAGCGAGATGTGAAAGCCCCGGGCTCAACCTGGGAATTGCATTTCGAACTGGCAAACTAGAGTCTTGTAGAGGGGGGTAGAATTCCAGGTGTAGCGGTGAAATGCGTAGAGATCTGGAGGAATACCGGTGGCGAAGGCGGCCCCCTGGACAAAGACTGACGCTCAGGCACGAAAGCGTGGGGAGCAAACAGG

>OTU323GTAGGGAATCTTGCGCAATGGGGGGAACCCTGACGCAGCAATGCCGCGTGCGGGATGACGGCCCTCGGGTTGTAAACCGCTTTCAGCAGGGAAGATAGTGACGGTACCTGCACAAGAAGCCCCGGCTAACTACGTGCCAGCAGCCGCGGTAACACGTAGGGGGCGAGCGTTATCCGGATTCATTGGGCGTAAAGCGCGCGCAGGCGGCCGCCTAAGCGGGACCTCTAATCCTGGGGCTCAACCTCAGGCCGGGTCCCGAACTGGGCGGCTCGGGTGCGGTAGGGGTAGGCGGAATTCCCGGTGTAGCGGTGGAATGCGCAGATATCGGGAAGAACACCGACGGCGAAGGCAGCCTACTGGGCCGCCACCGACGCTGAGGCGCGAAAGCTAGGGGAGCGAACAGG

>OTU324GTGGGGAATATTGCACAATGGGGGAAACCCTGATGCAGCGACGCCGCGTGAGTGAAGAAGTATTTCGGTATGTAAAGCTCTGTCAGCAGGGAAGAAAATGACGGTACCTGACCAAGAAGCCCCGGCTAACTACGTGCCAGCAGCCGCGGTAATACGTAGGGGGCAAGCGTTATCCGGATTTACTGGGTGTAAAGGGAGCGTAGACGGTGATGCAAGTCTGATGTGAAAGCCCGGGGCCCAACCCCGGGACTGCATTGGAAACTGTATGGCTGGAGTGCAGGAGAGGTAAGTGGAATTCCTAGTGTAGCGGTGAAATGCGTAGATATTAGGAGGAACACCAGTGGCGAAGGCGGCTTACTGGACTGTAACTGACGTTGAGGCTCGAAAGCGTGGGGAGCAAACAGG

>OTU325GTGGGGAATCTTGGACAATGGGGGCAACCCTGATCCAGCGACGCCGCGTGGGTGACGAAGGCCTTCGGGTTGTAAAGCCCTTTCACCGGGGACGATGGTGACGGTACCCGGGGAAGAAGCACCGGCTAACTCCGTGCCAGCAGCCGCGGTAAGACGGAGGGTGCGAGCGTTGTTCGGAATCACTGGGCGTAAAGGGCGCGTAGGCGGCCTGCCAAGTCGGGCGTGAAAGCCCGGGGCTCAACCCCGGAGGTGCGCCCGAGACTGGCGGGCTCGAGGCCGGGAGAGGGGGGTGGAATACCCAGTGGAGAGGTGAAATTCGTAGATATTGGGTGGAACACCGGTGGCGAAAGCGGCCCCCTGGCCCGGGTACCTGACGCTGAGGCGCGAAAGCGTGGGGAGCAAACAGG

>OTU326GTGGGGAATTGTTCGCAATGGGCGCAAGCCTGACGACGCAACGCCGCGTGGAGGACGAAGATCTTCGGGTCGTAAACTCCTGTCGAGCGGGAAGAACCCCGCGGGGACGAATACTCCCTGCGGCTGACGGTACCGCTAAAGGAAGCCCCGGCTAACTCCGTGCCAGCAGCCGCGGTAATACGGGGGGGGCAAGCGTTGTTCGGAATTACTGGGCGTAAAGGGCTCGTAGGCGGCCAACTAAGTCGGATGTGAAATCCCCAGGCTCAACCTGGGAACTGCATCCGATACTGGATGGCTTGAATCCGGGAGAGGGATGCAGAATTCCAGGTGTAGCGGTGAAATGCGTAGATATCTGGAGGAATACCGGTGGCGAAGGCGGCATCCTGGACCGGCATTGACGCTGAGGAGCGAAAGCCAGGGGAGCAAACGGG

>OTU327GTAGGGAATTTTCCGCAATGGACGAAAGTCTGACGGAGCAATGCCGCGTGAGTGATGAAGGTTTTCGAATCGTAAAGCTCTGTTGTAAGGGAAGAACAGCACTAGTAGGAAATGGCTAGTGTGTGACGGTACCTTACCAGAAAGCCACGGCTAACTACGTGCCAGCAGCCGCGGTAATACGTAGGTGGCAAGCGTTATCCGGAATTATTGGGCGTAAAGCGCATGTAGGCGGCTCTTTAAGTCTGATGTGAAAGCCAGTGGCTCAACCACTGAATTGCATTGGATACTGGAGAGCTAGAATCTTGGAGAGGTAAGTGGAATTTCATGTGTAGCGGTGGAATGCGTAGATATATGAAGGAACACCAATGGCGAAGGCAGCTTACTGGACAAGTATTGACGCTGAGATGCGAAAGTGTGGGTAGCAAACAGG

>OTU328GTAAGGAATATTGGACAATGGCCGCAAGGCTGATCCAGCCATCCAGCGTGCAGGAAGAAGGCCCTATGGGTTGTAAACTGCTTTTGTCAGGGAAGAAACCTCTGGATTTACTCCGGAGCTGACGGTACCTGAAGAATAAGCACCGGCTAACTACGTGCCAGCAGCCGCGGTAATACGTAGGGTGCAAGCGTTAATCGGAATTACTGGGCGTAAAGCGTGCGCAGGCGGTTTCGTAAGTCTGTCGTGAAATCCCCGGGCTTAACCTGGGAATGGCGATGGAGACTGCGAGGCTAGAGTTTGGCAGAGGGGGGTAGAATTCCACGTGTAGCAGTGAAATGCGTAGAGATGTGGAGGAACACCGATGGCGAAGGCAGCCCCCTGGGTCAAAACTGACGCTCATGCACGAAAGCGTGGGGAGCAAACAGG

>OTU329GTGAGGAATATTGCACAATGGGCGAAAGCCTGATGCAGCAACGCCGCGTGAGCGAAGAAGGCCTTTGGGTCGTAAAGCTCTGTCCTTGGGGAAGAAACAAATGACGGTACCCTTGGAGGAAGCCCCGGCTAACTACGTGCCAGCAGCCGCGGTAATACGTAGGGGGCGAGCGTTATCCGGAATTATTGGGCGTAAAGAGTGCGTAGGTGGTCTTGTAAGCGTGGGGTGAAAGGCAGTGGCTTAACCATTGTTAGCCTTGCGAACTGTGAGACTTGAGTGCAGGAGAGGAAAGCGGAATTCCTAGTGTAGCGGTGAAATGCGTAGATATTAGGAGGAACACCAGTGGCGAAGGCGGCTTTCTGGACTGTAACTGACACTGAGGCACGAAAGCGTGGGGAGCAAACAGG

>OTU330GTGGGGAATATTGCACAATGGGCGCAAGCCTGATGCAGCAACGCCGCGTGCGGGATGACGGCCTTCGGGTTGTAAACCGCTTTCAGCAGGGACGAAGCTTTTTGTGACGGTACCTGCAGAAGAAGCACCGGCTAACTACGTGCCAGCAGCCGCGGTGATACGTAGGGTGCGAGCGTTGTCCGGATTTATTGGGCGTAAAGGGCTCGTAGGCGGTTGATCGCGTCGGAAGTGGAAACTTGATGCTTAACGTTGAGCGTGCTTTCGATACGGGTTGACTTGAGGAAGGTAGGGGAGAATGGAATTCCTGGTGGAGCGGTGGAATGCGCAGATATCAGGAGGAACACCAGTGGCGAAGGCGGTTCTCTGGACCTTTCCTGACGCTGAGGAGCGAAAGCGTGGGGAGCGAACAGG

>OTU331GTGGGGAATATTGGGCAATGGGCGCAAGCCTGACCCAGCAACGCCGCGTGAAGGAAGAAGGCTTTCGGGTTGTAAACTTCTTTTGACAGGGAAGAGGAGAAGACGGTACCTGTCGAATAAGCTCCGGCTAACTACGTGCCAGCAGCCGCGGTAATACGTAGGGAGCGAGCGTTGTCCGGATTTACTGGGTGTAAAGGGCGTGCAGCCGGGAGTACAAGTCAGATGTGAAATGCGGGGGCTTAACCCCCGAACTGCATTTGAAACTGTTGATCTTGAGTATCGGAGAGGCAGGCGGAATTCCTAGTGTAGCGGTGAAATGCGTAGATATTAGGAGGAACACCAGTGGCGAAGGCGGCCTGCTGGACGACAACTGACGGTGAGGCGCGAAAGCGTGGGGAGCAAACAGG

>OTU332GTGGGGAATATTGCACAATGGGGGAAACCCTGATGCAGCGACGCCGCGTGGAGGAAGAAGGTCTTCGGATTGTAAACTCCTGTTGTTGAGGAAGATAATGACGGTACTCAACAAGGAAGTGACGGCTAACTACGTGCCAGCAGCCGCGGTAAAACGTAGGTCACAAGCGTTGTCCGGAATTACTGGGTGTAAAGGGAGCGCAGGCGGGAAGGCAAGTTGGAAGTGAAATCCATGGGCTCAACCCATGAACTGCTTTCAAAACTGTTTTTCTTGAGTAGTGCAGAGGTAGGCGGAATTCCCGGTGTAGCGGTGGAATGCGTAGATATCGGGAGGAACACCAGTGGCGAAGGCGGCCTACTGGGCACCAACTGACGCTGAGGCTCGAAAGTGTGGGTAGCAAACAGG

>OTU333GTGAGGAATATTGGTCAATGGCCGAGAGGCTGAACCAGCCAAGTCGCGTGAGGGATGACGGCCCTATGGGTTGTAAACCTCTTTTGTCAGGGAGCAAGACCCTCCACGTGTGGAGGAGCGAGAGTACCTGAAGAAAAAGCATCGGCTAACTACGTGCCAGCAGCCGCGGTAATACGTAGGTGGCAAGCGTTGTCCGGATTTATTGGGCGTAAAGCGAGCGCAGGCGGAAAGATAAGTCTGATGTGAAAGCCCTCGGCTTAACCGGGGAAGTGCATCGGAAACTGTTTTTCTTGAGTGCAGAAGAGGAGAGTGGAACTCCATGTGTAGCGGTGGAATGCGTAGATATATGGAAGAACACCAGTGGCGAAGGCGGCTCTCTGGTCTGCAACTGACGCTGAGGCTCGAAAGCATGGGTAGCGAACAGG

>OTU334GTGGGGAATATTGGGCAATGGGGGCAACCCTGACCCAGCGACGCCGCGTGGGGGAAGACGGCCTTCGGGTTGTAAACCTCTTTTGTACGGGAAGAAGGAAGTGACGGTACTGTACGAATAAGTCCCGGCTAACTACGTGCCAGCAGCCGCGGTAATACGTAGGGGACGAGCGTTGTCCGGAATTACTGGGCGTAAAGGGCGCGCAGGCTGTGCTGCAAGTCAGCTGTTAAATGTCTGGGCTTAACCCGGGCATGCGGTTGAGACTGCGGTGCTGGAGTGCTGTAGAGGCAAGTGGAATTCCCAGTGTAGCGGTGAAATGCGTAGATATTGGGAAGAACACCGGTGGCGAAGGCGACTTGCTGGGCAGCAACTGACGCTGAGGCGCGAAAGCCAGGGGAGCAAACGGG

>OTU335GTGGGGAATTGTTCGCAATGGGCGCAAGCCTGACGACGCAACGCCGCGTGGAGGATGAAGATCTTCGGGTCGTAAACTCCTGTCGAGCGGGACGAAAGATCCGCGACCTAATACGTCGCGGAAGTGACGGTACCGCTAGAGGAAGCCCCGGCTAACTCCGTGCCAGCAGCCGCGGTAATACGGGGGGGGCTAGCGTTGTTCGGAATTACTGGGCGTAAAGGGCTCGTAGGTGGCCAACTAAGTCATGCGTGAAATCCCTCGGCTTAACCGGGGAACTGCGTCTGATACTGGATGGCTTGAATCCGGGAGAGGGATGCGGAATTCCAGGTGTAGCGGTGAAATGCGTAGATATCTGGAGGAACACCGGTGGCGAAGGCGGCCCCCTGGGCTGGTACTGACGCTGAGGCGCGAAAGCGTGGGTAGCAAACGGG

>OTU336GTGGGGAATATTGGGCAATGGGCGCAAGCCTGACCCAGCAACGCCGCGTGAAGGAAGAAGGCTTTCGGGTTGTAAACTTCTTTTCTCAGGGACGAAGAAAGTGACGGTACCTGAGGAATAAGCCACGGCTAACTACGTGCCAGCAGCCGCGGTAATACGTAGGTGGCAAGCGTTATCCGGATTTATTGGGTGTAAAGGGCGTGTAGGCGGGAGCGCAAGTCAGATGTGAAAACTCAGGGCTCAACCCTGAGCCTGAATTTGAAACTGTGTTTCTTGAGTGCTGGAGAGGCAATCGGAATTCCGTGTGTAGCGGTGAAATGCGTAGATATACGGAGGAACACCAGTGGCGAAGGCGGATTGCTGGACAGTAACTGACGCTGAGGCGCGAAAGCGTGGGGAGCAAACAGG

>OTU337GTGGGGAATCTTGCGCAATGGGCGAAAGCCTGACGCAGCGACGCCGCGTGGGTGATGAAGGCCTTCGGGTTGTAAAGCACTTTCGGTGGGAACGAAATATGCCGGGCTAATACCCCGGTAGGCTGACGTTACTCAATGAAGAAGCACCGGCTAACTCTGTGCCAGCAGCCGCGGTAATACAGAGGGTGCGAGCGTTAATCGGAATTACTGGGCGTAAAGCGCACGTAGGTGGTTTTGTAAGTCGGATGTGAAATCCCCGGGCTTAACCCGGGAACTGCATTCGAGACTGCATTACTCGAGTATGGGAGAGGGAAGCGGAATTTCCGGTGTAGCGGTGAAATGCGTAGATATCGGAAGGAACATCAGTGGCGAAAGCGGCTTCCTGGCCCAATACTGACACTCATGTGCGAAAGCGTGGGGAGCAAACAGG

>OTU338GTGAGGAATATTGGACAATGGTCGAAAGACTGATCCAGCCATGCCGCGTGCAGGATGAATGCCCTATGGGTTGTAAACTGCTTTTATACGGGAAGAAAAACACCTACGTGTAGGTGACTGACGGTACCGTAAGAATAAGGACCGGCTAACTCCGTGCCAGCAGCCGCGGTAATACGGAGGGTCCAAGCGTTATCCGGAATCATTGGGTTTAAAGGGTCCGTAGGCGGGCTATTAAGTCAGAGGTGAAAGTTTGCAGCTCAACTGTAAAATTGCCTTTGATACTGATAGTCTTGAATTGTAGTGAAGTGGTTAGAATATGTGGTGTAGCGGTGAAATGCATAGATATCACATAGAATACCGATTGCGAAGGCAGATCACTAACTATATATTGACGCTGAGGGACGAAAGCGTGGGGAGCGAACGGG

>OTU339GTGGGGAATATTGCACAATGGGGGAAACCCTGATGCAGCCATGCCGCGTGTGTGAAGAAGGCCCTAGGGTTGTAAAGCACTTTCAGTAGGGAGGAAAGGGTGTTGATTAATACTCAACATCTGTGACGTTACCTACAGAAGAAGCACCGGCTAACTTCGTGCCAGCAGCCGCGGTAATACGAGGGGTGCAAGCGTTAATCGGAATTACTGGGCGTAAAGCGTGCGTAGGCGGTTTGTTAAGCAAGATGTGAAAGCCCAGGGCTCAACCTTGGAACTGCATTTTGAACTGGCAAACTAGAGTACTGTAGAGGGTGGTGGAATTTCCAGTGTAGCGGTGAAATGCGTAGAGATTGGAAGGAACATCAGTGGCGAAGGCGGCCACCTGGACAGATACTGACGCTGAGGCACGAAAGCGTGGGGAGCAAACAGG

>OTU340GTGGGGAATATTGCACAATGGGTGCAAGCCTGATGCAGCGACGCCGCGTGAGGGATGACGGCCTTCGGGTTGTAAACCTCTTTCGCTGGGGACGAAGCCTTTCGGGGTGACGGTACCTGGAGAAGAAGCACCGGCTAACTACGTGCCAGCAGCCGCGGTAATACGTAGGGTGCGAGCGTTGTCCGGAATTATTGGGCGTAAAGAGCTCGTAGGCGGTTTGTCGCGTCGGCCGTGAAAACCTACAGCTTAACTGTGGGCGTGCGGTCGATACGGGCAGACTTGAGTTCGGTAGGGGAGTCTGGAATTCCTGGTGTAGCGGTGAAATGCGCAGATATCAGGAGGAACACCGGTGGCGAAGGCGGGACTCTGGGCCGATACTGACGCTGAGGAGCGAAAGCGTGGGGAGCGAACAGG

>OTU341GTGGGGAATATTGCGCAATGGGCGAAAGCCTGACGCAGCGACGCCGCGTGAGGGATGAAGGTTCTCGAATCGTAAACCTCTGTCAGAAGGGAAGAAATTACATTGTGCTAATCAGCAGTGTATTGACGGTACCTTCAAAGGAAGCACCGGCTAACTCCGTGCCAGCAGCCGCGGTAATACGGAGGGTGCAAGCGTTAATCGGAATTACTGGGCGTAAAGCGCATGTAGGCGGCTTTATAAGTCAGGGGTGAAATCCCACGGCTCAACCGTGGAATTGCCCTTGATACTGTATAGCTTGAATTCAGTAGAGGGTGGCGGAATTTCAGGTGTAGGAGTGAAATCCGTAGATATCTGAAAGAACATCAGTGGCGAAGGCGGCCACCTGGACTGATATTGACGCTGAGATGCGAAAGCGTGGGGAGCAAACAGG

>OTU342GTGGGGAATATTGCACAATGGAGGGAACTCTGATGCAGCAACGCCGCGTGAGCGAAGAAGGTTTTAGGATCGTAAAGCTCTGTCCTAGGGGAAGATAATGACGGTACCCGAGGAGGAAGCCCCGGCTAACTACGTGCCAGCAGCCGCGGTAATACGTAGGGGGCGAGCGTTGTCCGGAATTATTGGGCGTAAAGGGTGCGTAGGCGGCTGAACAAGTCAGATGTGAAAGGTTAAGGCTTAACTTTAATAAGCATATGAAACTGTTCAGCTTGAGTTAAGGAGAGGGGAGTGGAATTCCTAGTGTAGCGGTGAAATGCGTAGATATTAGGAGGAATACCAGTGGCGAAGGCGACTCTCTGGACTTAAACTGACGCTGAGGCACGAAAGCGTGGGGAGCAAACAGG

>OTU343GTGGGGAATATTGCACAATGGAGGAAACTCTGATGCAGCGACGCCGCGTGAATGATGAAGGCCTTCGGGTCGTAAAATTCTGTCCTTGGGGAAGATAATGACGGTACCCAAGGAGGAAGCCCCGGCTAACTACGTGCCAGCAGCCGCGGTAATACGTAGGGGGCGAGCGTTGTCCGGAATTATTGGGCGTAAAGGGTTCGCAGGCGGTCTGATAAGTCAGATGTGAAAGGCTATGGCTCAACCATAGCAAGCATTTGAAACTGTCAGACTTGAGTTAAGGAGAGGAAAGTGGAATTCCTAGTGTAGCGGTGAAATGCGTAGATATTAGGAGGAATACCAGTGGCGAAGGCGACTTTCTGGACTTATACTGACGCTGAGGAACGAAAGCGTGGGGAGCAAACAGG

>OTU344GTGGGGAATATTGGGCAATGGGCGCAAGCCTGACCCAGCAACGCCGCGTGAAGGAAGAAGGCTTTCGGGTTGTAAACTTCTTTTATTCGGGACGATAATGACGGTACCGAATGAATAAGCCACGGCTAACTACGTGCCAGCAGCCGCGGTAATACGTAGGTGGCAAGCGTTATCCGGATTTACTGGGTGTAAAGGGCGTGTAGGCGGGACTGCAAGTCAGATGTGAAAACTATGGGCTCAACCCATAGCCTGCATTTGAAACTGTAGTTCTTGAGTACTGGAGAGGCAGGCGGAATTCCGTGTGTAGCGGTGAAATGCGTAGATATTAGGAGGAACATCAGTGGCGAAGGCGGCTTACTGGGCTTTAACTGACGCTGAGGCTCGAAAGCGTGGGGAGCAAACAGG

>OTU345ATTAGGAATCTTGCGCAATGGGGGCAACCCTGACGCAGCGACACAGCGTGGAGGATGAAGGTCTTCGGATTGTAAACTCCTTTTATATGGGAAGAATAAATGACTGTACCATATGAATAAGTACCTGCTAACTACGTGCCAGAAGCATCGGTGATGCGTAGGGTACGAACGTTATCCGGATTTACTGGGCGTATAGAGATGCGTAGGCGGTTTGATAAGTCTTGAGTTAAAGACCTCGGCTCAACCGAGGGAGAGCTTTCGAAACTATCAGACTTGAGACATGGAGGGGTGAACGGAATTCCGAGTGGAGTGGTGAAATACGTTGATATTCGGAGGAACATCAGTGGCGAAGGCGGTTCACTGGCCATTGTCTGACGCTAAGGCTCGAAAGCGTGGGTAGCAAAACGG

>OTU346GTGGGGAATCTTGCGCAATGGCCGAAAGGCTGACGCAGCGACGCCGCGTGGGGGAGGAAGGCCTTCGGGTCGTAAACCCCTGTTGCTCGGGACGAATGCCTGGTTTCGACCAGGGTGACGGTACCGGGTGAGGAAGCACCGGCTAACTCCGTGCCAGCAGCCGCGGTAATACGGAGGGTGCGAGCGTTGTCCGGAATCACTGGGCGTAAAGGGCGCGTAGGCGGCTTCCTAAGCGGGTGGTGAAAGCTCGGGGCTCAACCCCGAGTCGGCCATGCGAACTGGGAGGCTCGAGCACTGTAGAGGCAGGCGGAATTCCGGGTGTAGCGGTGGAATGCGTAGAGATCCGGAAGAACACCGGTGGCGAAGGCGGCCTGCTGGGCAGTAGCTGACGCTGAGGCGCGACAGCGTGGGGAGCAAACAGG

>OTU347GTGGGGAATATTGCACAATGGAGGGAACTCTGATGCAGCGATGCCGCGTGAGGGAAGAAGGTTTTCGGATTGTAAACCTCTGTCAACGGGGACGAAAATGACGGTACCCGAGAAGGAAGCTCCGGCTAACTACGTGCCAGCAGCCGCGGTAATACGTAGGGAGCGAGCGTTGTCCGGAATTACTGGGTGTAAAGGGAGCGTAGGCGGGGCTTTAAGTCAGGTGTGAAAACTACGGGCTCAACTCGTAGACTGCACTTGAAACTGAGGCTCTTGAGTGGAGCAAAGGCAGGCGGAATTCCCGGTGTAGCGGTGAAATGCGTAGATATCACGCAGAACCCCGATTGCGAAGGCAGCATACCGGCGCCCTACTGACGCTGAAGCACGAAAGCGTGGGTATCGAACAGG

>OTU348GTAAGGAATATTGGTCAATGGACGCAAGTCTGAACCAGCCATGCCGCGTGGAGGATGAAGGTCCTCTGGATTGTAAACTTCTTTTATCTGAGAAGAAACCCGGGAAATCCTTTCCGGTTGACGGTACCAGATGAATAAGCACCGGCTAACTCCGTGCCAGCAGCCGCGGTAATACGGAGGGTGCAAGCGTTATCCGGATTCACTGGGTTTAAAGGGTGCGTAGGTGGGTTGGTAAGTCCGTGGTGAAATCTCCGAGCTTAACTCGGAAACTGCCGTGGATACTATCAATCTTGAATACTGTGGAGGTAAGCGGAATATGTCATGTAGCGGTGAAATGCTTAGATATGACATAGAACACCGATAGCGAAGGCAGCTTGCTACACAGTCATTGACACTGAGGCACGAAAGCGTGGGGAGCAAACAGG

>OTU349GTGGGGGATATTGCACAATGGAGGAAACTCTGATGCAGCGACGCCGCGTGAGTGAAGAAGTATCTCGGTATGTAAAGCTCTATCAGCAGGGAAGAAAATGACAGTACCTGACCAAGAAGCCCCGGCTAACTACGTGCCAGCAGCCGCGGTAATACGTAGGGGGCAAGCGTTATCCGGATTTACTGGGTGTAAAGGGAGCGTAGACGGAATGTCAAGTCTGAAGTGAAATCCCCGGGCTTAACCCGGGAACTGCTTTGGAAACTGGTATTCTTGAGTGTCGGAGAGGTAAGCGGAATTCCTGGTGTAGTAGTGAAATGCGTAGATATCAGGAAGAACACCGGAGGCGAAGGCGGCTTACTGGACGATAACTGACGTTGAGGCTCGAAAGCGTGGGGAGCAAACAGG

>OTU350GTGAGGAATATTGGTCAATGGTCGGAAGACTGAACCAGCCAAGCCGCGTGAAGGAAGAAGGTGCTCGGCATCGTAAACTTCTTTTGTCAGGGAACAAAGGGTGGTACGTGTACCGCTGTGAGTGTACCTGAAGAAAAAGCATCGGCTAACTCCGTGCCAGCAGCCGCGGTAATACGGAGGATGCGAGCGTTATCCGGATTTATTGGGTTTAAAGGGTGCGTAGGCGGGACCTTAAGTCAGCGGTAAAATTGCGGTGCTCAACGCCGTAGAGCCGTTGAAACTGGGGTTCTTGAGTGAGCGAGAAGTATGCGGAATGCGTGGTGTAGCGGTGAAATGCATAGATATCACGCAGAACCCCGATTGCGAAGGCAGCATACCGGCGCTCAACTGACGCTGAGGCACGAAAGTGCGGGTATCGAACAGG

>OTU351GTGAGGAATATTGGTCAATGGGCTAAGGCCTGAACCAGCCAAGTCGCGTGAGGGAAGACGGTCCTATGGATTGTAAACCTCTTTTGTCGGGGAGCAAAAGCCGGACGTGTCCGGCTGTGAGAGTACCCGAAGAAAAAGCATCGGCTAACTCCGTGCCAGCAGCCGCGGTAATACGGAGGATGCGAGCGTTATCCGGATTTATTGGGTTTAAAGGGTGCGCAGGCGGATTTTTAAGTCAGCGGTGAAAGTCTGTGGCTCAACCATAGAATTGCCGTTGAAACTGGGGGGCTTGAGTATGTTTGAGGCAGGCGGAATGCGTGGTGTAGCGGTGAAATGCTTAGATATTGCGCAGAACTCCGATTGCGAAGGCAGCATACCGGCGGCGATCTGACGCTGAGGCACGAAAGCGTGGGTATCGAACAGG

>OTU352GTAGGGAATCTTCCGCAATGGACGAAAGTCTGACGGAGCAACGCCGCGTGAGTGATGAAGGTTTTCGGATCGTAAAACTCTGTTGTCGGGGAAGAACAAGTATGATAGTAACTGATCATACCTTGACGGTACCCGACCAGAAAGCCACGGCTAACTACGTGCCAGCAGCCGCGGTAATACGTAGGTGGCAAGCGTTGTCCGGAATTATTGGGCGTAAAGCGCTCGCAGGCGGTTCTTTAAGTCTGATGTGAAATCTTGCGGCTCAACCGTAAACGTGCATTGGAAACTGGAGGACTTGAGTGCAGAAGAGGAGAGTGGAATTCCACGTGTAGCGGTGAAATGCGTAGAGATGTGGAGGAACACCAGTGGCGAAGGCGACTCTCTGGTCTGTAACTGACGCTGAGGAGCGAAAGCGTGGGGAGCGAACAGG

>OTU353GTGGGGAATCTTGCGCAATGGGCGAAAGCCTGACGCAGCGACGCCGCGTGAGTGATGAAGGCCTTCGGGTTGTAAAGCTCTGTGGAGGGGGACGAATAAGGGTCGGCGAACACCCGGCCCGATGACGGTACCTCTTTAGCAAGCACCGGCTAACTCTGTGCCAGCAGCCGCGGTAAGACAGAGGGTGCGAACGTTGTTCGGAATTACTGGGCGTAAAGCGCGTGTAGGCAGCTTGGTAAGTCGGGTGTGAAAGCCCTGGGCTCAACCCAGGAAGTGCACTCGAAACTGCCGGGCTTGAGTTCCGGAGAGGAAGGCGGAATTCTCGGTGTAGAGGTGAAATTCGTAGATATCGAGAGGAACACCGGTGGCGAAGGCGGCCTTCTGGACGGCAACTGACGCTGAGACGCGAAAGCGTGGGGAGCAAACAGG

>OTU354GTGGGGAATTGTTCGCAATGGGCGCAAGCCTGACGACGCAACGCCGCGTGGAGGATGAAGGTCTTCGGATTGTAAACTCCTGTTGATCGGGACGAACGGGCTCCGGCCTAACACGCCGGAGTAATGACGGTACCGATTGAGGAAGCCACGGCTAACTCTGTGCCAGCAGCCGCGGTAATACAGAGGTGGCAAGCGTTGTTCGGAATTACTGGGCGTAAAGGGCGCGTAGGCGGCCTTCTTAGTCAGACGTGAAATCCCCGGGCTTAACTCGGGAACTGCGTCTGATACTGGAAGGCTCGAGTTCGGGAGAGGAATGTGGAATTCCAGGTGTAGCGGTGAAATGCGTAGATATCTGGAGGAACACCGGTGGCGAAGGCGGCATTCTGGACCGAAACTGACGCTGAGGCGCGAAAGCCAGGGGAGCAAACGGG

>OTU355GTGGGGAATATTGCGCAATGGACGAAAGTCTGACGCAGCGACGCCGCGTGTGGGATGACGGTCTTCGGATTGTAAACCACTGTCGGGAGGGACGAATACGCCGCAAGGCGGGTGACGGTACCTCCAAAGGAAGCACCGGCTAACTCCGTGCCAGCAGCCGCGGTAATACGGAGGGTGCGAGCGTTGTCCGGAATCACTGGGCGTAAAGGGCGCGTAGGCGGCCTGATAAGTAGGGGGTGAAATCCTGCGGCTTAACCGCAGGGCTGCCTTCTAAACTGTCGGGCTCGAGCACAGTAGAGGCAGGTGGAATTCCCGGTGTAGCGGTGGAATGCGTAGAGATCGGGAAGAACATCAGTGGCGAAGGCGGCCTGCTGGGCTGTTGCTGACGCTGAGGCGCGACAGCGTGGGGAGCAAACAGG

>OTU356GTCGGGAATTTTGGGCAATGGGCGAAAGCCTGACCCAGCAACGCCGCGTGAAGGATGAAGTATTTCGGTATGTAAACTTCGAAAGAATAGGAAGAATAAATGACGGTACTATTTATAAGCTCCGGCTAACTACGTGCCAGCAGCCGCGGTAATACGTAGGGAGCAAGCGTTGTTCGGATTTACTGGGCGTAAAGGGCGCGTAGGCGGCGCGGTAAGTCACTTGTGAAATCTCTGAGCTTAACTCAGAACGGCCAAGTGATACTGCAGTGCTAGAGTGCAGAAGGGGCAATCGGAATTCTTGGTGTAGCGGTGAAATGCGTAGATATCAAGAGGAACACCTGAGGTGAAGACGGGTTGCTAGGCTGACACTGACGCTGAGGCGCGAAAGCTAGGGGAGCAAACGGG

>OTU357GTGGGGAATCTTGCGCAATGGGCGAAAGCCTGACGCAGCAACGCCGCGTGGGGGATGAAGGCCCTCGGGTTGTAAACCCCTTTCAGCAGGAACGAATCTGACGGTACCTGCAGAAGAAGGCCCGGCCAACTACGTGCCAGCAGCCGCGGTAATACGTAGGTGGCAAGCGTTGTCCGGATTTACTGGGCGTAAAGAGCACGCAGGCGGTCGTTCAAGTCGAGTGTGAAAGCCCCCGGCTCAACTGGGGAGGGTCACTCGATACTGATCGACTCGAAGGCGGGAGAGGGAAGCGGAATTCCCGGTGTAGCGGTGAAATGCGTAGATATCGGGAAGAACACCAGTGGCGAAGGCGGCTCTCTGGACCATCACTGACGCTGAGACGCGAAAGCGTGGGGAGCAAACAGG

>OTU358GTGAGGAATATTGCCCAATGGACGCAAGTCTGAGGCAGCAACGCCGCGTGGGCGATGAAGGCCTTCGGGTTGTAAAGCCCTTTTCTGGGGGAAGAGGAAGGACGGTACCTCAGGAATAAGTCACGGCTAACTACGTGCCAGCAGCCGCGGTAATACGTAGGTGGCAAGCGTTATCCGGATTCACTGGGCGTAAAGCGCGTGCAGGCGGTTTCGTAAGTCGGACGTGAAAGCTCCTGGCTTAACTGGGAGAGGTCGTTCGATACTGCGGGGCTTGAGGTCGGGAGAGGGACGTGGAATTCCCGGTGTAGTGGTGGAATGCGTAGATATCGGGAGGAACACCAGTGGCGAAGGCGACGTCCTGGCCCGCACCTGACGCTGAGACGCGAAAGCGTGGGTAGCGAACGGG

>OTU359GTGGGGAATTTTGGACAATGGGCGCAAGCCTGATCCAGCCATGCCGCGTGAGTGAAGAAGGCCTTCGGGTTGTAAAGCTCTTTCGGCTGGGACGAAAAGGTCCGTGTTAACACCACGGATCCATGACGGTACCAGCAGAAGAAGCACCGGCTAACTACGTGCCAGCAGCCGCGGTAATACGTAGGGTGCAGGCGTTAATCGGAATTACTGGGCGTAAAGCGTGCGCAGGCGGTTTGCTAAGACAGGTGTGAAATCCCCGGGCTTAACCTGGGAACTGCATTTCAAACTGGCCGACTAGAGTACGGTAGAGGGAGGTAGAATTTCCTGTGTAGCGGTGAAATGCGTAGATATAGGAAGGAAGACCAGTGGCGAAGGCAGCCCCCTGGGCTAACACTGACGCTCATGCACGAAAGCGTGGGGAGCAAACAGG

>OTU360GTGGGGAATATTGGGCAATGGGGGGAACCCTGACCCAGCGACGCCGCGTGAGGGAAGAAGGTTTTCGGATCGTAAACCTCTGTTGTACGGGAAGAAGGAAGTGACGGTACCGTACGAGGAAGCCCCGGCAAACTACGTGCCAGCAGCCGCGGTAACACGTAGGGGGCGAGCGTTGTCCGGAATTACTGGGCGTAAAGGGCGCGCAGGCTGTTATGCGAGTCTGCTGTGAAATGTACCGGCTCAACCGGTGCCTTGCGGCGGATACTGCATAGCTGGAGTACTGGAGAGGGAAGTGGAATTCCCGGTGTAGCGGTGAAATGCGTAGATATCGGGAGGAACACCGGTGGCGAAGGCGGCTTTCTGGCCAGTTACTGACGCTCATGCGCGAAAGCTGGGGTAGCGAACCGG

>OTU361GTGAGGAATATTGGTCAATGGGCGAGAGCCTGAACCAGCCAAGTCGCGTGAAGGATGAAGGATCTATGGTTTGTAAACTTCTTTTATATGGGAATAAAGTGAGGAACGTGTTCCTTTTTGTATGTACCATATGAATAAGCATCGGCTAACTCCGTGCCAGCAGCCGCGGTAATACGGAGGATGCGAGCGTTATCCGGATTTATTGGGTTTAAAGGGTGCGTAGGTGGTTAATTAAGTCAGCGGTGAAAGTTTGTGGCTCAACCATAAAATTGCCGTTGAAACTGGTTGACTTGAGTATATTTGAGGTAGGCGGAATGCGTGGTGTAGCGGTGAAATGCATAGATATCACGCAGAACTCCGATTGCGAAGGCAGCTTACTAAACTATAACTGACACTGAAGCACGAAAGCGTGGGGATCAAACAGG

>OTU362GTCGGGAATTTTGGGCAATGGGCGAAAGCCTGACCCAGCAACGCCGCGTGGAGGATGAAGTTTCTCGGAATGTAAACTCCGTAAGAATGGGACGAATAAGGAGGGGCTAATACTCCCTCTGATGACGGTACCATTCGTAAGCTCCGGCTAACTCCGTGCCAGCAGCCGCGGTAATACGGGGGGAGCAAGCGTTGTTCGGATTTACTGGGCGTAAAGGGCGCGTAGGCGGCGCAACAAGTCACTTGTGAAATCTCCGGGCTTAACCCGGAGCGGCCAAGTGATACTGTCGTGCTAGAGTGCGGAAGGGGCTACTGGAATTCTCGGTGTAGCGGTGAAATGCGTAGATATCGAGAGGAACACCTGTGGCGAAGGCGGGTAGCTGGGCCGACACTGACGCTGAGGCGCGAAAGCTAGGGGAGCGAACGGG

>OTU363GTGGGGAATATTGCACAATGGGCGCAAGCCTGATGCAGCCATGCCGCGTGTGTGAAGAAGGCCTTCGGGTTGTAAAGCACTTTCAGCGGGGAGGAAGGGTCACTAGTTAATACCTAGTGGCATTGACGTTACTCGCAGAAGAAGCACCGGCTAACTCCGTGCCAGCAGCCGCGGTAATACGGAGGGTGCAAGCGTTAATCGGAATTACTGGGCGTAAAGCGCACGCAGGCGGTTTGTTAAGTCAGATGTGAAATCCCCGAGCTCAACTTGGGAACTGCATTTGAAACTGGCAAGCTAGAGTCTTGTAGAGGGGGGTAGAATTCCAGGTGTAGCGGTGAAATGCGTAGAGATCTGGAGGAATACCGGTGGCGAAGGCGGCCCCCTGGACAAAGACTGACGCTCAGGTGCGAAAGCGTGGGGAGCAAACAGG

>OTU364GTGGGGAATATTGGACAATGGGCGCAAGCCTGATCCAGCAATGCCGCGTGTGTGAAGAAGGCCTTCGGGTTGTAAAGCACTTTTATCAGGAACGAAACACTGTCGGCTAATACCCGGCGGGACTGACGGTACCTGAGGAATAAGCACCGGCTAACTTCGTGCCAGCAGCCGCGGTAATACGAAGGGTGCAAGCGTTAATCGGAATTACTGGGCGTAAAGCGTGCGTAGGCGGTTTGTTAAGTCTGTTGTGAAATCCCCGGGCTCAACCTGGGAATGGCAATGGATACTGGCAAGCTAGAGTGTGTCAGAGGATGGTGGAATTCCCGGTGTAGCGGTGAAATGCGTAGAGATCGGGAGGAACATCAGTGGCGAAGGCGGCCCCCTGGACAAAGACTGACGCTCAGGTGCGAAAGCGTGGGGAGCAAACAGG

>OTU365GTGGGGAATATTGCACAATGGGCGAAAGCCTGATGCAGCAACGCCGCGTGAAGGATGAAGGTCTTCGGATTGTAAACTTCTATCAGCAGGGAAGAAGAAAGTGACGGTACCTGAATAAGAAGCTCCGGCTAACTACGTGCCAGCAGCCGCGGTAATACGTAGGGAGCAAGCGTTATCCGGAATTACTGGGTGTAAAGGGTGCGCAGGCGGTCCTGTAAGTCACGAGTGAAATTTTGAGGCTCAACCTCAAAGCTGCTAGTGAAACTATAGGGCTAGAGTGTGGGAGAGGAAAGTGGAACTTTGAGTGTAGCGGTGAAATGCGTAGAGATTCAAAGGAACACCAGTAGCGAAGGCGGCTTTCTGGACCATAACTGACGCTCAGGCACGAAAGCGTGGGGAGCAAACAGG

>OTU366GTGGGGAATATTGGACAATGGGCGCAAGCCTGATCCAGCCATGCCGCGTGGATGATGAAGGCCCTAGGGTTGTAAAGTCCTTTCGGCGGGGAAGATAATGACGGTACCCGGAGAAGAAGCACCGGCTAACTCCGTGCCAGCAGCCGCGGTAAGACGGAGGGTGCTAGCGTTGTTCGGAATCACTGGGCGTAAAGGGCGCGTAGGCGGCCCGGCAAGTCGGATGTGAAAGCCCGGGGCTCAACCCCGGAACGGCATCCGAGACTGTCGGGCTGGAGGCCGGAAGAGGAGGGTGGAATTCCCAGTGTAGAGGTGAAATTCGTAGATATTGGGAGGAACACCGGTGGCGAAAGCGGCTCTCTGGTCCGGTTCTGACGCTGAGGCGCGAAAGCGTGGGGAGCAAACAGG

>OTU367GTGAGGAATATTGGTCAATGGGCGCGAGCCTGAACCAGCCAAGTCGCGTGAGGGAAGACGGCCCTACGGGTTGTAAACCTCTTTTGTCGGGGAGCAAGGACTGCCACGAGTGGCAGGGCGAGAGTACCCGAAGAAAAAGCATCGGCTAACTCCGTGCCAGCAGCCGCGGTAATACGGAGGATGCGAGCGTTATCCGGATTTATTGGGCGTAAAGCGTGTGTAGGCGGTTTATTAAGTCTAAGATTAAAGCCCGGGGCTTAACCTCGGTTTGTTTTAGAAACTGGTAGACTTGAGTGTGGTAGAGGCAAGTGGAATTTCTAGTGTAGCGGTTAAATGCGTAGATATTAGAAGGAACACCAGTGGCGAAGGCGGCTTGCTGGGCCATTACTGACGCTGAGACACGAAAGCGTGGGGAGCAAATAGG

>OTU368GTGGGGAATATTGCACAATGGGGGGAACCCTGATGCAGCAATGCCGCGTGGGTGAAGAAGTACTTCGGTATGTAAAGCCCTATCAGCAGGGAAGAAAATGACGGTACCTGACTAAGAAGCCCCGGCTAACTACGTGCCAGCAGCCGCGGTAATACGTAGGGGGCAAGCGTTATCCGGATTTACTGGGTGTAAAGGGAGCGTAGACGGCGATGCAAGTCTGAAGTGAAAGGCGGGGGCCCAACCCCCGGACTGCTTTGGAAACTGTATAGCTGGAGTGCAGGAGAGGTAAGTGGAATTCCTAGTGTAGCGGTGAAATGCGTAGATATTAGGAGGAACACCAGTGGCGAAGGCGGCTTACTGGACTGTAACTGACGTTGAGGCTCGAAAGCGTGGGGAGCAAACAGG

>OTU369GTGGGGAATCTTGGACAATGGGCGCAAGCCTGATCCAGCCATGCCGCGTGAGTGACGAAGGCCTTAGGGTTGTAAAGCTCTTTTGCCGGGGACGATAATGACGGTACCCGGAGAATAAGTCCCGGCTAACTTCGTGCCAGCAGCCGCGGTAATACGAAGGGGACTAGCGTTGTTCGGAATTACTGGGCGTAAAGCGCACGTAGGCGGATTTGTTAGTCAGGGGTGAAATCCCGGGGCTCAACCTCGGAACTGCCTTTGATACTGCAAATCTCGAGTCCGATAGAGGTGGGTGGAATTCCTAGTGTAGAGGTGAAATTCGTAGATATTGGGAGGAACACCGGTGGCGAAAGCGGCTCTCTGGTCCGGTTCTGACGCTGAGGCGCGAAAGCGTGGGGAGCAAACAGG

>OTU370GTGGGGAATATTGCGCAATGGGCGAAAGCCTGACGCAGCGACGCCGCGTGAGGGATGAAGGTTTTCGGATCGTAAACCTCTGTCGGAAGGGAAGAAGGCGCACCGTGCCAATCAGCGGTGTGTTGACGGTACCTTCAAAGGAAGCGCCGGCTAACTCCGTGCCAGCAGCCGCGGTAATACGGAGGGCGCAAGCGTTAATCGGAATTACTGGGCGTAAAGCGCGCGTAGGCTGTTTCGTAAGTCAGGGGTGAAATCCCACGGCTCAACCGTGGAACTGCCCTTGATACTGCGGGACTTGAATCCGGGAGAGGGTGGCGGAATTCCAGGTGTAGGAGTGAAATCCGTAGATATCTGGAGGAACATCAGTGGCGAAGGCGACTATCTGGCACATAACTGACACTGAGGAGCGAAAGCGTGGGTAGCAAACAGG

>OTU371GTGAGGAATATTGGTCAATGGCCGGAAGGCTGAACCAGCCAAGTCGCGTGAGGGATGAAGGATCTATGGTTCGTAAACCTCTTTTATAAGGGAATAAAGTGCGGGACGTGTCCTGTTTTGTATGTACCTTATGAATAAGGATCGGCTAACTCCGTGCCAGCAGCCGCGGTAATACGGAGGATCCGAGCGTTATCCGGATTTATTGGGTTTAAAGGGTGCGTAGGCGGCCTTTTAAGTCAGCGGTGAAAGTCTGTGGCTCAACCATAGAATTGCCGTTGAAACTGGGGGGCTTGAGTATGTTTGAGGCAGGCGGAATGCGTGGTGTAGCGGTGAAATGCATAGATATCACGCAGAACCCCGATTGCGAAGGCAGCCTGCCAAGCCATGACTGACGCTGATGCACGAAAGCGTGGGGATCGAACAGG

>OTU372GTGAGGAATATTGGTCAATGGGCGAGAGCCTGAACCAGCCAAGTCGCGTGAAGGACGACGGTTCTATGGATTGTAAACTTCTTTTGTAGAGGAATAATGGCAGCTACGCGTAGCTGAGATGCATGTACTCTACGAATAAGTATCGGCTAACTCCGTGCCAGCAGCCGCGGTAATACGGAGGATACGAGCGTTATCCGGAATTATTGGTTTTAAAGGGTGCGTAGGTGGCGTATTAAGTCAGTGGTGAAAAGCTGCAGCTCAACTGTAGTCTTGCCGTTGAAACTGATATGCTAGAGAGGAGACGAGGTATGCGGAATGTGTGGTGTAGCGGTGAAATGCATAGATATCACACAGAACGCCGATTGCGAAGGCAGCGTACCAGGCTCCGTCTGACACTGAAGCACGAAAGCGTGGGGATCAAACAGG

>OTU373GTGGGGAATATTGCACAATGGGGGAAACCCTGATGCAGCGACGCCGCGTGGGGGATGACGGCCTTCGGGTTGTAAACCTCTTTCAGTAGGGGAGAAGCCTTTTTTAGGTGACGGTACCTGCAGAAGAAGTACCGGCTAACTACGTGCCAGCAGCCGCGGTAATACGTAGGGTACAAGCGTTGTCCGGAATTATTGGGCGTAAAGAGCTCGTAGGTGGTTTGTCGCGTCTGCTGTGGAAACGCAAGGCTCAACCTTGCGCGTGCAGTGGGTACGGGCGGACTAGAGTGCAGTAGGGGAGTCTGGAATTCCTGGTGTAGCGGTGAAATGCGCAGATATCAGGAGGAACACCGGTGGCGAAGGCGGGACTCTGGGCTGTTACTGACGCTGAGGAGCGAAAGCATGGGGAGCGAACAGG

>OTU374GTGAGGAATATTGGTCAATGGCCGAGAGGCTGAACCAGCCAAGTCGCGTGAGGGAAGACGGCCCTACGGGTTGTAAACCTCTTTTGTCGGGGAGCAAAGCGAACCACGTGTGGTTCATTGAGAGTACCCGAAGAAAAAGCATCGGCTAACTCCGTGCCAGCAGCCGCGGTAATACGGAGGATGCGAGCGTTATCCGGATTTATTGGGTTTAAAGGGTGCGCAGGCGGAGGTGCAAGTCAGCGGTCAAATTGCGGGGCTCAACCCCGTACTGCCGTTGAAACTGCATCCCTTGAGTGCGCGAGAAGTATGCGGAATGCGTGGTGTAGCGGTGAAATGCATAGATATCACGCAGAACTCCGATTGCGAAGGCAGCATACCGGCGCGCAACTGACGCTCATGCACGAAAGCGTGGGTATCGAACAGG

>OTU375GTAGGGAATCTTCCACAATGGGCGAAAGCCTGATGGAGCAACGCCGCGTGAGTGAAGAAGGATTTCGGTTCGTAAAACTCTGTTGTAAGGGAAGAACAAGTACAGTAGTAACTGGCTGTACCTTGACGGTACCTTATTAGAAAGCCACGGCTAACTACGTGCCAGCAGCCGCGGTAATACGTAGGTGGCAAGCGTTGTCCGGAATTATTGGGTTTAAAGGGTGCGTAGGCAGAAGATTAAGTCAGCGGTGAAATGTATATGCTCAACATATAACGTGCCGTTGAAACTGGTTTACTAGGATACGAATGCTGTGGGAGGAATGTGTAATGTAGCGGTGAAATGCATAGATATTACACAGAACACCGATTGCGAAGGCATCTCACAAATTCGTCATTGACGCTGAGGCACGAAAGTGCGGGGATCAAACAGG

>OTU376GTGGGGAATTTTGGACAATGGGGGCAACCCTGATCCAGCCATTCCGCGTGAGTGAAGAAGGCCTTCGGGTTGTAAAGCTCTTTTGTCCGGAACGAAACGGGTAGCGTGAACATCGCTGCCTACTGACGGTACCGGAAGAATAAGCACCGGCTAACTACGTGCCAGCAGCCGCGGTAATACGTAGGGTGCGAGCGTTAATCGGAATTACTGGGCGTAAAGCGTGCGCAGGCGGTTTTGTAAGCCAGATGTGAAAGCCCCGGGCTTAACCTGGGAATGGCATTTGGGACTGCAAGGCTTGAGTGCGGCAGAGGAGACTGGAATTCCCGGTGTAGCAGTGAAATGCGTAGATATCGGGAGGAATACCGATGGCGAAGGCAGGTCTCTGGGCTGACACTGACGCTCATGCACGAAAGCGTGGGGAGCAAACAGG

>OTU377GTGGGGAATCTTGCGCAATGGCCGAAAGGCTGACGCAGCGACGCCGCGTGAGGGATGACGGCCTTCGGGTTGTAAACCTCTGTTGCCCGGGACGAACAGCCTGACTCGTCAGGCCTGACGGTACCGGGTGAGGAAGCACCGGCTAACTCTGTGCCAGCAGCCGCGGTAAGACAGAGGTGGCAAGCGTTGTCCGGATTTACTGGGCGTAAAGGGCGTGTAGGCGGTTCCACAAGTCGGCCGTGAAAAGCGTCGGCTCAACCGAAGCAGGGCGACCGAGACTGTGGGGCTTAGAGTGCGGGAGAGGGAAGTGGAATTCCCGGTGTAGCAGTGAAATGCGTAGATATCGGGAAGAACACCAATGGCGAAAGCGACTTCCTGGCCCGCTACTGACGCTGAGGCGCGAAAGCCAGGGGAGCGAACGGG

>OTU378GTAAGGGATATTGCACAATGGGCGCAAGCCTGATGCAGCAACGCCGCGTGCCCGATGAAGGCCTTCGGGTCGTAAAGGGCTTTTCTGACCGAAGAGACAAGGACAGTAAGTCAGGAATAAGTGTCGGCTAACTACGTGCCAGCAGCCGCGGTAAAACGTAGGACGCAAGCGTTATCCGGAGTTACTGGGCGTAAAGCGCGGGCAGGCGGCCTGTAAAGTCTGATGTGAAAGCGCCCGGCTCAACCGGGCGAGGCCATTGGAAACTAGCAGGCTGGAGGGCAGTAGAGGCAGGCGGAATTCCGGGTGGAGTGGTGAAATGCGTAGAGATCCGGAGGAACACCAGAGGCGAAGGCGGCCTGCTGGGCTGCACCTGACGCTGAGCCGCGAAAGCCAGGGGAGCGAACGGG

>OTU379GTGAGGGATCTTGCGCAATGGGGGCAACCCTGACGCAGCGACGCCGCGTGGGCGATGAAGGCTTTCGGGTCGTAAAGCCCTTTTCCCACTGAGGAGAACAAGGACAGTAGGTGGGGAAGAAGTCTCGGCTAACTACGTGCCAGCAGCCGCGGTAAAACGTAGGAGGCGAGCGTTATCCGGAGTTACTGGGCGTAAAGCGCGTGCAGGCGGCAGGTCAAGTGGCGGGTGAAAGCGCCCGGCTCAACCGGGCGAGTGCCGGTCAGACTGACGTGCTGGAGGACCGGAGAGGGACGTGGAATTCCGGGTGTAGCGGTGAAATGTGCAGAGATCCGGAGGAACACCAGCGGCGCAAGCGGCGTCCTGGACGGGAACTGACGCTGAGACGCGACAGCGTGGGGAGCGAACGGG

>OTU380GTGGGGAATATTGCGCAATGGCCGCAAGGCTGACGCAGCGACGCCGCGTGTGGGATGAAGGCCTTCGGGTTGTAAACCACTGTCAGGGGAGACGAATACCCGGCCTCGAATAGGGGACCGGGGGTGACGGTACCCCCAAAGGAAGCACCGGCTAACTCCGTGCCAGCAGCCGCGGTAATACGGAGGGTGCGAGCGTTGTCCGGAATCACTGGGCGTAAAGGGCGCGTAGGCGGTCTGGTAAGTCACTTGTGAAATCCCGGTGCTCAACGCCGGGGCCGCAGGTGAGACTGTCAGACTGGAGCTTGGTAGAGGCGAGTGGAATTCCCGGTGTAGCGGTGGAATGCGTAGATATCGGGAAGAACACCGGTGGCGAAGGCGGCTCGCTGGGCCAATGCTGACGCTGAGGCGCGAAAGCGTGGGGAGCAAACAGG

>OTU381GTGGGGAATATTGGACAATGGGCGAAAGCCTGATCCAGCCATGCCGCGTGTGTGAAGAAGGTCTTCGGATTGTAAAGCACTTTAAGTTGGGAGGAAGGGTACTTACCTAATACGTGAGTATTTTGACGTTACCGACAGAATAAGCACCGGCTAACTCTGTGCCAGCAGCCGCGGTAATACAGAGGGTGCAAGCGTTAATCGGAATTACTGGGCGTAAAGCGCGCGTAGGTGGTTCGTTAAGTTGAATGTGAAATCCCCGGGCTCAACCTGGGAACTGCATCCAAAACTGGCGAGCTAGAGTATGGTAGAGGGTGGTGGAATTTCCTGTGTAGCGGTGAAATGCGTAGATATAGGAAGGAACACCAGTGGCGAAGGCGACCACCTGGACTGATACTGACACTGAGGTGCGAAAGCGTGGGGAGCAAACAGG

>OTU382GTGAGGAATATTGGACAATGGGTGGAAGCCTGATCCAGCCATCCTGCGTGTAGGATGACGGCCTTATGGGTTGTAAACTACTTTTATCTGGGGATAAACCTACTTACGTGTAAGTAGCTGAAGGTACCAGAAGAATAAGCACCGGCTAACTCCGTGCCAGCAGCCGCGGTAATACGGAGGGTGCAAGCGTTATCCGGATTTATTGGGTTTAAAGGGTCCGTAGGCGGATTAATCAGTCAGTGGTGAAATCTCATAGCTTAACTATGAAACTGCCATTGATACTGTTAGTCTTGAGTGATGTTGAAGTTGCTGGAATGTGTAGTGTAGCGGTGAAATGCTTAGATATCACGCAGAACTCCGATTGCGAAGGCAGCATACCGGCGCTCAACTGACGCTCATGCACGAAAGTGTGGGTATCGAACAGG

>OTU383GTCGGGAATATTGCCCAATGGACGAAAGTCTGAGGCAGCAACGCCGCGTGCGCGATGAAGGCCTTCGGGTCGTAAAGCGCTTTTCGGGAGGATGAGGAAGGACAGTACTCCCGGAATAAGGAACGGCTAACTACGTGCCAGCAGCCGCGGTAAAACGTAGGTTCCGAGCGTTATCCGGATTTACTGGGCGTAAAGCGTGTTCAGGCGGCTCAGCAAGTCGGGCATGAAATCTCTCGGCTCAACCGGGAGAGGCTGTCCGATACTGCTGGGCTTGAGGGCAGTAGAGGGTGGTGGAATTCCGGGTGTAGTGGTGAAATGCGTAGATATCCGGAGGAACACCAGTGGCGAAAGCGGCCACCTGGACTGTACCTGACGCTCAAACACGAAAGCTAGGGGAGCGAACGGG

>OTU384GTGGGGAATCTTGCGCAATGGGCGAAAGCCTGACGCAGCGACGCCGCGTGAGTGATGAAGGCCTTCGGGTTGTAAAGCTCTGTGGGGAGAGACGAATAAGTGCAGCCTAATACGTTGCATGATGACGGTATCTCCTTAGCAAGCACCGGCTAACTCTGTGCCAGCAGCCGCGGTAAGACAGAGGGTGCAAACGTTGTTCGGAATTACTGGGCGTAAAGCGTGTGTAGGCGGTCTCCTAAGTCGGGTGTGAAAGCCCTGGGCTCAACCCAGGAAGTGCACTCGAAACTGGGAGGCTAGAGTATCGGAGAGGTTGGTGGAATTCTCGGTGTAGAGGTGAAATTCGTAGATATCGAGAGGAACACCGGTGGCGAAGGCGGCCAACTGGACGAATACTGACGCTGAGACACGAAAGCGTGGGGAGCAAACAGG

>OTU385GGGGGGAATATTGCGCAATGGGCGAAAGCCTGACGCAGCGACGCCGCGTGGAGGATGAAGGTCTTAGGATTGTAAACTCCTGTCAGATGGGAAGAAAAGATTTGTTTCTAATAAAGATGAATTATGACGGTACCATCAGAGGAAGCACCGGCTAACTTCGTGCCAGCAGCCGCGGTAATACGAAGGGTGCGAGCGTTATTCGGAATCACTGGGCGTAAAGAGTGCGTAGACGGTGTGTTAAGTTTGTTGTTAAAGACTTCAGCCTAACTGGAGGTTCGCAATGAAAACTGGCATGCTAGAGGGTGAGAGAGAGAAGTGGAATTCCTGGAGTAGCGGTAAAATGCGTGGATCTCAGGAGGAACACCAATGGCGAAGGCAGCTTCTTGGCTCATTTCTGACGTTGAGGCACGAAAGCGTGGGGAGCAAACAGG

>OTU386GTGGGGAATATTGCACAATGGGGGAAACCCTGATGCAGCGACGCCGCGTGAGTGAAGAAGTATTTCGGTATGTAAAGCTCTATCAGCAGGGAAGATGATGACGGTACCTGAGTAAGAAGCCCCGGCTAACTACGTGCCAGCAGCCGCGGTAATACGTAGGGGGCAAGCGTTATCCGGATTTACTGGGTGTAAAGGGAGCGTAGACGGCTGTGCAAGTCTGAAGTGAAAGGCATGGGCTCAACCCGTGGACTGCTTTGGAAACTGTGCAGCTAGAGTGTCGGAGAGGCAAGTGGAATTCCTAGTGTAGCGGTGAAATGCGTAGAGATTAGGAAGAACACCAGTGGCGAAGGCGACTTACTGGACTGTAACTGACGCTGAGGCATGAGAGCATGGGGAGCAAACAGG

>OTU387GTGGGGAATTGTTCACAATGGGCGCAAGCCTGATGACGCAACGCCGCGTGGGGGATGAAGGTCTTCGGATTGTAAACCCCTGTCGAACGGGACGAAAGACTCGGGAGCTAATCATCTCGAGAGTGACGGTACCGTTAAAGGAAGCCACGGCTAACTCTGTGCCAGCAGCCGCGGTAATACAGAGGTGGCAAGCGTTGTTCGGAATTACTGGGCGTAAAGGGCGCGTAGGCGGCCTTCTAAGTCAGACGTGAAATCCCCCGGCTTAACCTGGGAACTGCGTCTGATACTGGGAGGCTTGAGTATGGGAGAGGGATGTAGAATTCCAGGTGTAGCGGTGAAATGCGTAGATATCTGGAGGAATACCGGTGGCGAAGGCGGCATCCTGGACCATAACTGACGCTGATGCGCGAAAGCTAGGGGAGCAAACGGG

>OTU388GTAAGGAATATTGGTCAATGGACGCAAGTCTGAACCAGCCATGCCGCGTGAAGGATTAAGGCCCTCAGGGTTGTAAACTTCTTTTATTTGGGAAGAAACCCACGGTTTCTACTGTGGTTGACGGTACCAGATGAATAAGCACCGGCTAACTCCGTGCCAGCAGCCGCGGTAATACGGAGGGTGCAAGCGTTATCCGGATTCACTGGGTTTAAAGGGTGCGTAGGCGGGTTGGTAAGTCCGTGGTGAAATCCCCAAGCTTAACTTGGGAACTGCCGTGGATACTATCAATCTTGAATATCGTGGAGGTAAGCGGAATATGTCATGTAGCGGTGAAATGCTTAGATATGACATAGAACACCAATTGCGTAGGCAGCTTACTACACGATCATTGACGCTGAGGCACGAAAGCGTGGGGAGCAAACAGG

>OTU389GTGGGGAATATTGGACAATGGGCCACAAGCCTGATCCAGCAATTCTGTGTGCACGATGAAGGTCTTCGGATTGTAAAGTGCTTTCAGTTGGGAAGAAGAAAGTGACGGTACCAACAGAAGAAGCGACGGCTAAATACGTGCCAGCAGCCGCGGTAATACGTATGTCGCAAGCGTTATCCGGATTTATTGGGCGTAAAGCGCGTCTAGGCGGAAAAATAAGTCTGATGTTAAAATGCGGGGCTCAACTCCGTATTGCGTTGGAAACTGTTTTTCTAGAGTACTGGAGAGGTGGGCGGAACTACAAGTGTAGAGGTGAAATTCGTAGATATTTGTAGGAATGCCGATGGAGAAGTCAGCTCACTGGACAGATACTGACGCTAAAGCGCGAAAGCGTGGGGAGCAAACAGG

>OTU390GTGGGGAATATTGGACAATGGGCGCAAGCCTGATCCAGCCATGCCGCGTGAGTGATGAAGGCCTTAGGGTTGTAAAGCTCTTTTACCCGGGAAGATAATGACGGTACCGGGAGAATAAGCCCCGGCTAACTTCGTGCCAGCAGCCGCGGTAATACGAAGGGGGCTAGCGTTGTTCGGATTTACTGGGCGTAAAGCGCACGTAGGCGGATCTTTAAGTCAGAGGTGAAATCCCAGGGCTCAACCCTGGAACTGCCTTTGATACTGGGGATCTCGAGTCCGGGAGAGGTGAGTGGAACTGCGAGTGTAGAGGTGAAATTCGTAGATATTCGCAAGAACACCAGTGGCGAAGGCGGCTCACTGGCCCGGAACTGACGCTGAGGTGCGAAAGCGTGGGGAGCAAACAGG

>OTU391GTGAGGAATATTGGTCAATGGACGGAAGTCTGAACCAGCCAAGTAGCGTGCAGGATGACGGCCCTATGGGTTGTAAACTGCTTTTGTATGGGGATAAAGTTAGGGACGTGTCCCTATTTGCAGGTACCATACGAATAAGGACCGGCTAATTCCGTGCCAGCAGCCGCGGTAATACGGAAGGTCCAGGCGTTATCCGGATTTATTGGGTTTAAAGGGAGCGTAGGCTGGATATTAAGTGTGTTGTGAAATGTAGACGCTCAACGTCTGACTTGCAGCGCATACTGGTTTCCTTGAGTACGCACAACGTTGGCGGAATTCGTCGTGTAGCGGTGAAATGCTTAGATATGACGAAGAACTCCGATTGCGAAGGCAGCTGACGGGAGCGCAACTGACGCTTAAGCTCGAAGGTGCGGGTATCAAACAGG

>OTU392GTCGAGAATAATTCACAATGGACGAAAGTCTGATGGTGCAACGCCGCGTGGAGGATGACGGTCTTCGGATTGTAAACTCCTGTCATCCGGGAGTAAGACCTGGCTGTGAATAGCAGACAGGGTTGATAGTACCGGAAGAGGAAGGGACGGCTAACTTCGTGCCAGCAGCCGCGGTAATACGAAGGTCCCGAGCGTTGTTCGGAATCACTGGGCGTAAAGGGAGCGTAGGCGGCGTGGTAAGTCAGATGTGAAATCCCGGGGCTCAACCCCGGAACTGCATCCGATACTGCCGTGCTAGAGGATTGGAGAGGTAGCTGGAATTCTTGGTGTAGCAGTGAAATGCGTGGAGATCAAGAGGAACACTCGTGGCGAAAGCGAGCTACTGGACAATATCTGACGCTGAGGCTCGAAGGCCAGGGTAGCGAAAGGG

>OTU393GTAAGGAATATTGGACAATGGCCGCAAGGCTGATCCAGCCATCCAGCGTGCAGGAAGAAGGCCCTATGGGTTGTAAACTGCTTTTGTCAGGGAATAAACCCTTGGATTTACTCCGAGGCTGAAGGTACCTGGTGAATAAGCCACGGCTAACTACGTGCCAGCAGCCGCGGTAATACGTAGGTGGCAAGCGTTGTCCGGATTTATTGGGTTTAAAGGGTGCGTAGGCGGTTGTATAAGTCAGTGCTGAAATATCCCGGCTTAACCGGGAGGGTGGCATTGATACTGTATGACTTGAGTAAAGTTGAGGTTGGCGGAATTGACGGTGTAGCGGTGAAATGCTTAGATATCGTCAAGAACACCTATTGCGAAGGCAGCTAACTAAGCTTTAACTGACGCTGAGGCACGAAAGTGTGGGGATCAAACAGG

>OTU394GTGAGGAATATTGGACAATGGATGGAAATCTGATCCAGCCATGCCGCGTGCAGGAAGAAGGTCCTATGGATTGTAAACTGCTTTAGTGCGGGAGCAATAAGGTCCACGAGTGGACCGATGAGAGTACCGCAAGAATAAGCATCGGCTAACTCCGTGCCAGCAGCCGCGGTAATACGGGGGATGCAAGCGTTATCCGGATTTATTGGGTTTAAAGGGTGCGTAGGCGGTCTGTCAAGTCGGCGGTGAAAACAGCATGCTAAACATGGTGCTTGCCGTCGAGACTGGTGGGCTTGAATGTGGATGATGCCGGCGGAATGTGTAAAGTAGCGGTGAAATGCATAGATATTACACAGAAGGTCGATTGCGAAAGCAGCTGGCAAAGGCACTATTGACGCTGAGGCACGAAAGTGCGGGGATCGAACAGG

>OTU395GTGGGGAATATTGCGCAATGGGGGAAACCCTGACGCAGCAACGCCGCGTGAGTGACGAAGGCCTTCGGGTCGTAAAGCTCTGTCGGAGGGAAAGAAACTATTTGTGGCTAATATCCACGAATATTGACGGTACCCTCAAAGGAAGCACCGGCTAACTCCGTGCCAGCAGCCGCGGTAATACGGAGGGTGCAAGCGTTGTTCGGAATTACTGGGCGTAAAGCGCGTGTAGGTGGCTTGTCATGTCAGGTGTGAAAGCCCTCGGCCTAACCGAGGAAGTGCGCCTGAAACTGCCAAGCTTGAGTACGAGAGAGGAGGACGGAATTCCCAGTGTAGAGGTGAAATTCGTAGATATTGGGAGGAACACCGGTGGCGAAAGCGGTTCTCTGGATCGATACTGACACTAAGACGCGAAAGCGTGGGGATCAAACAGG

>OTU396GTGAGGAATTTTGGACAATGGGGGAAACCCTGATCCAGCGACGCCGCGTGAAGGATGAAGGTCTTCGGATTGTAAACTTCTTTTGCCTGGGAATAACCGCCCCGTAGAAATACGGGGAATGAATGTACCAGGCGAATAAGCCACGGCTAACTTCGTGCCAGCAGCCGCGGTAATACGAAGGTGGCAAGCGTTACTCGGAATTACTAGGCGTAAAGGGCAGGTAGGCGGTTTGGTAAGTCTGTTGTGAAAGCTCCTTGCTTAACGGGGAGAGGCCAACAGATACTGCCAGGCTTGAGTATAGGAGAGGGTACTGGAATTCCCGGTGTAGCGGTGAAATGCGCAGAGATCGGGAGGAACACCAATGGCGAAAGCAGGTACCTGGACTATTACTGACGCTCAGCTGCGAAAGCTAGGGGAGCAAACAGG

>OTU397GTAGGGAATCTTCCACAATGGACGAAAGTCTGATGGAGCAACGCCGCGTGAGTGAAGAAGGATTTCGGTTCGTAAAACTCTGTTGCAAGGGAAGAACAAGTAGCGTAGTAACTGGCGCTACCTTGACGGTACCTTGTTAGAAAGCCACGGCTAACTACGTGCCAGCAGCCGCGGTAATACGTAGGTGGCAAGCGTTGTCCGGAATTATTGGGCGTAAAGCGCGCGCAGGTGGTTCCTTAAGTCTGATGTGAAAGCCCCCGGCTCAACCGGGGAGGGTCATTGGAAACTGGGGAACTTGAGTGCAGAAGAGGATAGTGGAATTCCAAGTGTAGCGGTGAAATGCGTAGAGATTTGGAGGAACACCAGTGGCGAAGGCGACTGTCTGGTCTGTAACTGACACTGAGGCGCGAAAGCGTGGGGAGCAAACAGG

>OTU398GTGGGGAATATTGGACAATGGGGGCAACCCTGATCCAGCCATGCCGCGTGAGTGAAGAAGGCCTTCGGGTTGTAAAGCTCTTTCGGCGGGGACGATGATGACGGTACCCGCATAAGAAGCCCCGGCAAACTTCGTGCCAGCAGCCGCGGTAATACGAAGGGGGCTAGCGTTGTTCGGAATTACTGGGCGTAAAGCGCGCGTAGGCGGTGATCTTTGTCAGAGGTGAAATCCCGAGGCTCAACTTCGGAATTGCCTTTGAAACGGGATCGCTTGAGTCCGAGAGAGGATGGCGGAATTCCTAGTGTAGAGGTGAAATTCGTAGATATTAGGAAGAACACCGGTGGCGAAGGCGGCCATCTGGCTCGGTACTGACGCTCAGGCGCGAAAGCGTGGGGAGCAAACAGG

>OTU399GTGGGGAATATTGGACAATGGGCGCAAGCCTGATCCAGCAATGCCGCGTGAGTGAAGAAGGCCTTAGGGTTGTAAAGCTCTTTCGGCGGGGAAGATGATGACGGTACCCGCAGAAGAAGCCCCGGCTAACTCCGTGCCAGCAGCCGCGGTAATACGGAGGGGGCTAGCGTTGTTCGGAATTACTGGGCGTAAAGGGCGCGTAGGCGGTGCGGTAAGTTAGGCGTGAAAGGCCCGGGCTCAACCCGGGAACTGCGCTTAAAACTGCCGCGCTCTGAGTCCGAGAGAGGATAGCGGAATTCCCAGTGTAGAGGTGAAATTCGTAGATATTGGGAAGAACACCAGTGGCGAAGGCGGCTATCTGGCTCGGTACTGACGCTGAGGCGCGAAAGCGTGGGGAGCAAACAGG

>OTU400GTGAGGAATATTGGTCAATGGGCGGTAGCCTGAACCAGCCAAGTAGCGTGAAGGATGAAGGTTCTATGGATTGTAAACTTCTTTTATAAAGGAATAAAGTGAGGCACGTGTGCCTTTTTGTATGTACTTTATGAATAAGGATCGGCTAACTCCGTGCCAGCAGCCGCGGTAATACGGAGGATCCGAGCGTTATCCGGATTTATTGGGTTTAAAGGGAGCGTAGATGGGTTGTTAAGTCAGTTGTGAAAGTTTGCGGCTCAACCGTAAAATTGCAATTGATACTGGCGTCCTTGAGTACAGTTGAGGTGGGCGGAATTCGTGGTGTAGCGGTGAAATGCTTAGATATCACGAAGAACTCCTATTGCGAAGGCAGCTCACTAAACTGCAACTGACATTGAGGCTCGAAAGTGTGGGTATCAAACAGG

>OTU401GTGAGGAATATTGGTCAATGGGCGGGAGCCTGAACCAGCCAAGCCGCGTGAGGGAGGAAGGCCCTATGGGTCGTAAACCTCTTTTGTCAGGGAACAAAAGCGAGTATGTATACTAGTCTGAGTGTACCTGAAGAAAAAGCATCGGCTAACTCCGTGCCAGCAGCCGCGGTAATACGGAGGATGCGAGCGTTATCCGGATTTATTGGGTTTAAAGGGTGCGTAGGCGGCGATGCAAGTCAGCGGTAAAAGCCCGGGGCTCAACCCCGGCGAGCCGTTGAAACTGCAGTGCTAGAGAAGGCAAGAGGTACGCGGAATGCGCAGTGTAGCGGTGAAATGCTTAGATATTGCGCAGAACTCCGATTGCGAAGGCAGCGTACTGGCGCCTGACTGACGCTGAGGCACGAAAGCGTGGGGATCGAACAGG

>OTU402GTGAGGAATATTGGTCAATGGGCGGGAGCCTGAACCAGCCAAGCCGCGTGAGGGAAGAAGGCGCTCAGCGTCGTAAACCTCTTTAGCCGGGGAACAAAGAGCTGCTCGGGAAGCAGCGTTGAGCGTACCCGGAGAATAAGCATCGGCTAACTCCGTGCCAGCAGCCGCGGTAATACGGAGGATGCGAGCGTTATCCGGATTTATTGGGTTTAAAGGGTGCGCAGGCGGCGCGGTAAGTCAGCGGTAAAAGCCCGGGGCTCAACCCCGGCGAGCCGTTGAAACTGCCGTGCTAGAGTAAGGTCGAGGTATGCGGAATGCGCGGTGTAGCGGTGAAATGCATAGATATCGCGCAGAACTCCGATTGCGAAGGCAGCATACCGGTCCTTTACTGACGCTCAGGCACGAAAGCGTGGGGATCAAACAGG

>OTU403GTGGGGAATATTGCGCAATGGGCGAAAGCCTGACGCAGCGACGCCGCGTGAGGGATGAAGGTTTTCGGATCGTAAACCTCTGTCAGAAGGGAAGAAGTTATGCCGTTCTAATCAGCGGCATATTGACGGTACCTTCAAAGGAAGCACCGGCTAACTCCGTGCCAGCAGCCGCGGTAATACGGAGGGTGCGAGCGTTAATCGGAATTACTGGGCGTAAAGCGCACGTAGGCTGTATGTCAAGTCAAGGGTGAAATCCCACGGCTCAACCGTGGAACTGCCTTTGAAACTGGCAAACTGGAGTATGTGAGAGGATGGCGGAATTCCTGGTGTAGGAGTGAAATCCGTAGATATCAGGAGGAACATCAGTGGCGAAGGCGGCCATCTGGCACATAACTGACGCTGAGGTGCGAAAGCGTGGGTAGCAAACAGG

>OTU404GTGGGGAATATTGGACAATGGGGGCAACCCTGATCCAGCCATGCCGCGTGAGTGATGACGGCCTTAGGGTTGTAAAGCTCTTTTACCCGGGACGATAATGACGGTACCGGGTGAATAAGCCCCGGCTAACTTCGTGCCAGCAGCCGCGGTAATACGAAGGGGGCTAGCGTTGCTCGGAATCACTGGGCGTAAAGGGCGCGTAGGCGGCTTGCCAAGTCGGGGGTGAAAGCCCGTGGCTCAACCACGGAATGGCCTTCGATACTGGCAGGCTTGAGACCGGAAGAGGACAGCGGAACTGCGAGTGTAGAGGTGAAATTCGTAGATATTCGCAAGAACACCAGTGGCGAAGGCGGCTGTCTGGTCCGGTTCTGACGCTGAGGCGCGAAAGCGTGGGGAGCAAACAGG

>OTU405GTGGGGGATATTGCGCAATGGGGGAAACCCTGACGCAGCAACGCCGCGTGAAGGAAGACGGTTTTCGGATTGTAAACTTCTTTTGTTAAGGACGAACAATGACGGTACTTAACGAATAAGCTCCGGCTAACTACGTGCCAGCAGCCGCGGTAATACGTAGGGAGCAAGCGTTGTCCGGATTTACTGGGTGTAAAGGGTGCGTAGGCGGCAAGGCAAGTCAGATGTGAAATGCACGGGCTTAACCCGTGAGCTGCATTTGAAACTGTTTTGCTTGAGTGAAGTAGAGGCAGGCGGAATTCCCGGTGTAGCGGTGAAATGCGTAGAGATCGGGAGGAACACCAGTGGCGAAGGCGGCCTGCTGGGCTTTAACTGACGCTGAGGCACGAAAGCGTGGGTAGCAAACAGG

>OTU406GTCGGGAATATTGCGCAATGGAGGAAACTCTGACGCAGTGACGCCGCGTATAGGAAGAAGGTTTTCGGATTGTAAACTATTGTCGTTAGGGAAGATAAAAGACTGTACCTAAGGAGGAAGCCCCGGCTAACTATGTGCCAGCAGCCGCGGTAATACATAGGGGGCAAGCGTTATCCGGAATTATTGGGTGTAAAGGGTGCGTAGACGGAAGAACAAGTTGGTTGTGAAATCCCTCGGCTCAACTGAGGAACTGCAACCAAAACTATTCTCCTTGAGTGTCGGAGAGGAAAGTGGAATTCCTAGTGTAGCGGTGAAATGCGTAGATATTAGGAGGAACACCAGTGGCGAAGGCGACTTTCTGGACGATAACTGACGTTGAGGCACGAAAGTGTGGGGAGCAAACAGG

>OTU407GTGAGGAATATTGGTCAATGGGCGGAAGCCTGAACCAGCCAAGTCGCGTGAGGGAAGACGGCCCTACGGGTTGTAAACCTCTTTTGCCGGGGAGCAATGCCCAGCTCGCGAGCTGGGAAGGAGAGTACCCGGAGAAAAAGCATCGGCTAACTCCGTGCCAGCAGCCGCGGTAATACGGAGGATGCGAGCGTTATCCGGATTTATTGGGTTTAAAGGGTGCGTAGGCGGTCTGTTAAGTCAGCGGTCAAATGTCGGCGCTCAACGCCGGCCTGCCGTTGAAACTGGCGCCCTTGAGTGGGCGAGAAGTATGCGGAATGCGTGGTGTAGCGGTGAAATGCATAGATATCACGCAGAACCCCGATTGCGAAGGCAGCATACCGGCGCCCTACTGACGCTGAAGCACGAAAGCGTGGGTATCGAACAGG

>OTU408GTGAGGAATATTGGTCAATGGCCGAAGGGCTGAACCAGCCAAGTCGCGTGAAGGATGAAGGTTCTATGGATTGTAAACTTCTTTTATCAGGGAATAAAGTGTGGTACGTGTACCATTTTGTATGTACCTGATGAATAAGCATCGGCTAACTCCGTGCCAGCAGCCGCGGTAATACGGAGGATGCGAGCGTTATCCGGATTTATTGGGTTTAAAGGGTGCGTAGGTGGTAAATTAAGTCAGCGGTGAAAGTTTGTGGCTCAACCATAAAATTGCCGTTGAAACTGTTTTACTTGAGTGTGTTTGAGGTAGGCGGAATGCGTGGTGTAGCGGTGAAATGCATAGATATCACGCAGAACTCCAATTGCGAAGGCAGCTTACTAAACCATAACTGACACTGAAGCACGAAAGCGTGGGGATCAAACAGG

>OTU409GGAAGGAATATTGGACAATGGGCGAAAGCCTGATCCAGCAATACCGCGTGAATGATGAAGGCCTTAGGGTTGTAAAGTTCTTTTAATAGGGAAGATAATGACGGTACCTATAGAAAAAGCCCCGGCTAACTCCGTGCCAGCAGCCGCGGTAAGACGGAGGGGGCTAGCGTTGTTCGGAATTACTGGGCGTAAAGGGCGCGTAGGCGGATTAGTAAGTTGGGAGTGAAATCCCGGGGCTCAACCTCGGAATGGCTCTCAAAACTACTAGTCTTGAGTGAAGTAGGGGATGATGGAATTTCTAGTGTAGAGGTGAAATTCTTAGATATTAGAAGGAACACCGGTGGCGAAGGCGGTCATCTGGACTTCAACTGACGCTGAGGCGCGAAAGCGTGGGGAGCAAACAGG

>OTU410GTGGGGAATATTGCACAATGGGGGAAACCCTGATGCAGCCATGCCGCGTGTGTGAAGAAGGCCTTCGGGTTGTAAAGCACTTTCAGTAGGGAGGAAGGTGTTGTCGTTAATAGCGGCAGCATTTGACGTTACCTACAGAAGAAGCACCGGCTAACTCCGTGCCAGCAGCCGCGGTAATACGGAGGGTGCGAGCGTTAATCGGAATTACTGGGCGTAAAGCGCATGCAGGTGGCTTGTTAAGCCAGATGTGAAAGCCCGGGGCTTAACCTCGGAATAGCATTTGGAACTGGCAAGCTAGAGTACTGTAGAGGGGGGTAGAATTTCAGGTGTAGCGGTGAAATGCGTAGAGATCTGAAGGAATACCGGTGGCGAAGGCGGCCCCCTGGACAGATACTGACACTCAGATGCGAAAGCGTGGGGAGCAAACAGG

>OTU411GTGGGGAATCTTGCGCAATGGGCGAAAGCCTGACGCAGCGACGCCGCGTGAGTGATGAAGGCCTTCGGGTTGTAAAGCTCTGTGGAGGGGGACGAATAAGGGCCGTCGAATAGACGGCTCGATGACGGTACCCCTTTAGCAAGCACCGGCTAACTCTGTGCCAGCAGCCGCGGTAAGACAGAGGGTGCAAACGTTGTTCGGAATTACTGGGCGTAAAGCGCGTGTAGGCGGCTCGGCAAGTCGGGTGTGAAAGCCCTGGGCTTAACCTAGGAAGTGCACTCGAAACTGCCAAGCTTGAGTTCTGGAGAGGAAGGCGGAATTCTCGGTGTAGAGGTGAAATTCGTAGATATCGAGAGGAACACCGGTGGCGAAGGCGGCCTTCTGGACAGCAACTGACGCTGAGACGCGAAAGCGTGGGGAGCAAACAGG

>OTU412GTGGGGAATTTTGGGCAATGGGGGAAACCCTGACCCAGCGACGCCGCGTGAGGGAAGACGGCCTTCGGGTTTTAAACCTCTTTTGTACGGGAAGATAATGACGGTACTGTACGAATAAGTCCCGGCTAACTACGTGCCAGCAGCCGCGGTAATACGTAGGGGACGAGCGTTGTCCGGAATTACTGGGCGTAAAGGGCGCGCAGGCTGCGTTACAAGTCAGCTGTTAAATGCATGGGCTTACCCCATGAGTGCGGTTGAAACTGTAGTGCTAGAGTACTGTAGAGGCAAGCGGAATTCCCAGTGTAGCGGTGAAATGCGTAGATATTGGGAGGAACACCGGTGGCGTAGGCGGCTTGCTGGACAGTAACTGACGCTGAGGCGCGAAAGCCAGGGGAGCAAACGGG

>OTU413GTGGGGAATATTGCACAATGGGAGAAAGCCTGATGCAGCAACGCCGCGTGAGCGAAGAAGGTCTTCGGATCGTAAAGATCTGTCCTAGGGGAAGAAAAAAATGACGGTACCCAAGGAGGAAGCCCCGGCTAACTACGTGCCAGCAGCCGCGGTAATACGTAGGGGGCAAGCGTTATCCGGAATTATTGGGCGTAAAGAGTGCGTAGGTGGTTACCTAAGCAGGGGGTGAAAGGCAATGGATCAACCATTGTTAGCCCTCTGAACTGGGCTACTTGAGTGCAGGAGAGGAAAGCGGAATTCCTAGTGTAGCGGTGAAATGCGTAGATATTAGGAGGAACACCAGTGGCGAAGGCGGCTTTCTGGACTGAAACTGACACTGAGGCACGAAAGTGTGGGGAGCAAACAGG

>OTU414GTGGGGAATTTTGGACAATGGGGGCAACCCTGATCCAGCAATGCCGCGTGAGTGAAGAAGGCCTTCGGGTTGTAAAGCTCTTTTGTCAGGGAAGAAACGGTTGTGGCTAATATCCATGGCTAATGACGGTACCTGAAGAATAAGCACCGGCTAACTACGTGCCAGCAGCCGCGGTAATACGTAGGGTGCAAGCGTTAATCGGAATTACTGGGCGTAAAGCGTGCGCAGGCGGTTTTATAAGACAGAGGTGAAATCCCCGGGCTCAACCTGGGAACTGCCTTTGTGACTGTAAGGCTAGAGTGTGTCAGAGGGGGGTAGAATTCCACGTGTAGCAGTGAAATGCGTAGATATGTGGAGGAATACCGATGGCGAAGGCAGCCCCCTGGGATAACACTGACGCTCATGCACGAAAGCGTGGGGAGCAAACAGG

>OTU415GTGAGGAATCTTCCACAATGGGCGAAAGCCTGATGGAGCAACGCCGCGTGCAGGATGAAGGCCTTCGGGTCGTAAACTGCTTTTATAAGTGAAGAATATGACGGTAGCTTATGAATAAGCACCGGCTAACTACGTGCCAGCAGCCGCGGTCATACGTAGGGTGCAAGCATTATCCGGAGTGACTGGGCGTAAAGAGTTGCGTAGGTGGTTTGTTAAGTAGGTAGTGAAATCTGGCGGCTCAACCGTACAGGCTATTATCTAAACTGGCAAACTCGAGAATGGTAGAGGTAACTGGAATTTCTTGTGTAGGAGTGAAATCCGTAGATATAAGAAGGAACACCAATGGCGTAGGCAGGTTACTGGACCATTTCTGACACTAAGGCACGAAAGCGTGGGGAGCGAACGGG

>OTU416GTGAGGAATCTTCCACAATGGGCGAAAGCCTGATGGAGCAACGCCGCGTGAAGGATGAAGGCCCTCGGGTTGTAAACTTCTTTTATGAGTGAAGAATATGACGGTAACTCATGAATAAGCACCGGCTAACTACGTGCCAGCAGCCGCGGTCATACGTAGGGTGCAAGCATTATCCGGAGTGACTGGGCGTAAAGAGTTGCGTAGGCGGTCTACAAAGTGAATAGTGAAACCTGGTGGCTCAACCATACAGACTATTATTCAAACTCGTAGACTCGAGAGTGGTAGAGGTAACTGGAATTTCTTGTGTAGGAGTGAAATCCGTAGATATAAGAAGGAACACCAATGGCGTAGGCAGGTTACTGGACCATTTCTGACGCTAAGGCACGAAAGCGTGGGGAGCGAACCGG

>OTU417GTAGGGAATCTTCCGCAATGGACGAAAGTCTGACGGAGCAACGCCGCGTGAACGATGAAGGCCTTCGGGTCGTAAAGTTCTGTTGTTAGGGAAGAACAAGTACCGGAGTAACTGCCGGTACCTTGACGGTACCTAACCAGAAAGCCACGGCTAACTACGTGCCAGCAGCCGCGGTAATACGTAGGTGGCAAGCGTTGTCCGGAATTATTGGGCGTAAAGCGCGCGCAGGCGGTCCTTTAAGTCTGATGTGAAAGCCCACGGCTCAACCGTGGAGGGTCATTGGAAACTGGGGGACTTGAGTGCAGAAGAGGAAAGCGGAATTCCACGTGTAGCGGTGAAATGCGTAGAGATGTGGAGGAACACCAGTGGCGAAGGCGGCTTTCTGGTCTGTAACTGACGCTGAGGCGCGAAAGCGTGGGGAGCAAACAGG

>OTU418GTGGGGAATATTGGGCAATGGGCGCAAGCCTGACCCAGCAACGCCGCGTGAAGGAAGAAGGCTTTCGGGTTGTAAACTTCTTTTCTTAGGGACGAATACATGACGGTACCTAAGGAATAAGCCACGGCTAACTACGTGCCAGCAGCCGCGGTAATACGTAGGTGGCAAGCGTTATCCGGATTTATTGGGTGTAAAGGGCGTGTAGGCGGGAATGCAAGTCAGATGTGAAAACTATGGGCTCAACCCATAGCCTGCATTTGAAACTGTATTTCTTGAGTGCTGGAGAGGCAATCGGAATTCCGTGTGTAGCGGTGAAATGCGTAGATATACGGAGGAACACCAGTGGCGAAGGCGGATTGCTGGACAGTAACTGACGCTGAGGCGCGAAAGCGTGGGGAGCAAACAGG

>OTU419GTGGGGAATATTGCACAATGGGGGAAACCCTGAAGCAGCAACGCCGCGTGGAGGATGAAGGTTTTAGGATTGTAAACTCCTTTTGTTAGAGAAGATTATGACGGTATCTAACGAATAAGCACCGGCTAACTCCGTGCCAGCAGCCGCGGTAATACGGAGGGTGCAAGCGTTACTCGGAATCACTGGGCGTAAAGAGTGCGTAGGCGGGGTAATAAGTCAGATGTGAAATCCTGTAGCTTAACTACAGAACTGCATTTGAAACTGTTACTCTGGAGTGTGGGAGAGGTAGGTGGAATTCTTGGTGTAGGGGTAAAATCCGTAGAGATCAAGAGGAATACTCATTGCGAAGGCGACCTGCTGGAACATTACTGACGCTGATGCACGAAAGCGTGGGGAGCAAACAGG

>OTU420GTGAGGAATATTGGTCAATGGGCGCGAGCCTGAACCAGCCAAGTCGCGTGAGGGAAGACGGCCCTACGGGTTGTAAACCTCTTTTGTCAGGGAGCAACGGCCGCCACGTGTGGCGGAGAGGAGAGTACCTGAAGAAAAAGCATCGGCTAACTCCGTGCCAGCAGCCGCGGTAATACGGAGGATGCGAGCGTTATCCGGATTTATTGGGTTTAAAGGGTGCGTAGGCGGGAAGTTAAGTCAGCGGTAAAATTGAGGGGCTCAACCCCTTCGAGCCGTTGAAACTGGCTTTCTTGAGTGGAAGAGAAGTACGCGGAATGCGTGGTGTAGCGGTGAAATGCATAGATATCACGCAGAACTCCGATTGCGAAGGCAGCGTACCGGCATCCGACTGACGCTGAGGCACGAAAGCGTGGGGATCAAACAGG

>OTU421GTGAGGAATATTGGTCAATGGGCGAGAGCCTGAACCAGCCAAGTCGCGTGAGGGAAGACGGTCCTATGGATTGTAAACCTCTTTTGTCGGGGAGCAAGAAAGGGTATGTATACCCTACTGAGAGTACCCGAAGAAAAAGCATCGGCTAACTCCGTGCCAGCAGCCGCGGTAATACGGAGGATGCGAGCGTTATCCGGATTTATTGGGTTTAAAGGGTGCGTAGGCGGAGTGCCAAGTCAGCGGTAAAAATTCGGGGCTCAACCCCGTCGTGCCGTTGAAACTGGCGCCCTTGAGTGGGCGAGAAGTATGCGGAATGCGTGGTGTAGCGGTGAAATGCATAGATATCGCGCAGAACTCCGATTGCGAAGGCAGCGTACCGGTGTCTTTCTGACGCTGAGGCACGAAAGCGTGGGGATCGAACAGG

>OTU422GTGAGGAATATTGGACAATGGGTGAGAGCCTGATCCAGCCATCCCGCGTGAAGGATGACGGTCCTATGGATTGTAAACTTCTTTTGTACAGGGATAAACCTACTCTCGTGAGGGTAGCTGAAGGTACTGTACGAATAAGCACCGGCTAACTCCGTGCCAGCAGCCGCGGTAATACGGAGGGTGCAAGCGTTATCCGGATTTATTGGGTTTAAAGGGTCCGTAGGCGGACCTGTAAGTCAGTGGTGAAATCTCATAGCTTAACTATGAAACTGCCATTGATACTGCAGGTCTTGAGTAAATTTGAAGTGGCTGGAATAAGTAGTGTAGCGGTGAAATGCATAGATATTACTTAGAACACCAATTGCGAAGGCAGGTCACTAAGATTTAACTGACGCTGATGGACGAAAGCGTGGGTAGCGAACAGG

>OTU423GTGAGGGATATTGGTCAATGGGGGAAACCCTGAACCAGCAACGCCGCGTGAGGGAAGACGGTTTTCGGATTGTAAACCTCTGTCCTCTGTGAAGATGATGACGGTAGCAGAGGAGGAAGCTCCGGCTAACTACGTGCCAGCAGCCGCGGTAATACGTAGGGAGCGAGCGTTGTCCGGATTTACTGGGTGTAAAGGGTGCGTAGGCGGCCCTGCAAGTCAGAAGTGAAATCCATGGGCTTAACCCGTGAACGGCTTTTGAAACTGCAGGACTTGAGTGAAGTAGAGGCAGGCGGAATTCCCGGTGTAGCGGTGAAATGCGTAGAGATCGGGAGGAACACCAGTGGCGAAGGCGGCCTGCTGGGCTTTAACTGACGCTGAAGCACGAAAGCGTGGGTAGCAAACAGG

>OTU424GTGAGGAATATTGGTCAATGGCCGGAAGGCTGAACCAGCCAAGTCGCGTGAGGGATGACGGCCCTACGGGTTGTAAACCTCTTTTGTCAGGGAGCAAAGAACGTCACGTGTGGCGTCTCGAGAGTACCTGAAGAAAAAGCATCGGCTAACTCCGTGCCAGCAGCCGCGGTAATACGGAGGATGCGAGCGTTATCCGGATTTATTGGGTTTAAAGGGTGCGTAGGCGGAAGATTAAGTCAGCGGTAAAATCGAGTGGCTCAACCACTTCCGGCCGTTGAAACTGGTTTTCTTGAGTGAGCGAGAAGTATGCGGAATGCGTGGTGTAGCGGTGAAATGCATAGATATCGCGCAGAACTCCGATTGCGAAGGCAGCATACCGGCGGCTATCTGACGCTGAGGCACGAAAGCGTGGGTATCGAACAGG

>OTU425GTAGGGAATCTTCCGCAATGGACGAAAGTCTGACGGAGCAACGCCGCGTGAACGATGAAGGCTTTCGGGTCGTAAAGTTCTGTTGTCAGGGAAGAACAAGTACCGGAGTAACTGCCGGTACCTTGACGGTACCTGACCAGAAAGCCACGGCTAACTACGTGCCAGCAGCCGCGGTAATACGTAGGTGGCAAGCGTTGTCCGGAATTATTGGGCGTAAAGCGCGCGCAGGCGGTTCCTTAAGTCTGATGTGAAAGCCCGGGGCTCAACCCCGGCGGGTCTTTGGATACTGTCGAACTTGAGTACGGAAGAGGAGAGTGGAATTCCAGGTGTAGCGGTGAAATGCGTAGATATCTGGAGGAACACCGGTGGCGAAAGCGGCTCTCTGGTCCGATACTGACGCTCATGCTCGAAAGCGTGGGGAGCAAACGGG

>OTU426GTCGGGAATTTTGCTCAATGGACGAAAGTCTGAAGCAGCAACGCCGCGTGAGGGATGAAGGCCTTCGGGTTGTAAACCTCTTTTATCAGGGACGATAATGACGGTACCTGATGAATAAGTCACGGCTAACTACGTGCCAGCAGCCGCGGTAATACGTAGGTGACTAGCGTTGTCCGGATTTACTGGGCGTAAAGAGCGCGCAGGCGGTCGATTTAGTCGAGTGTGAAAGCCCCCGGCTCAACTGGGGAGGGTCACTCGATACTGATCGACTCGAAGGCGGGAGAGGGAAGCGGAATTCCCGGTGTAGTGGTGAAATGCGTAGATATCGGGAGGAACACCAGTGGCGAAGGCGGCTTCCTGGCCCGTTCTTGACGCTGAGGCGCGAAAGCTAGGGGAGCAAACGGG

>OTU427GTGGGGAATTGTTCGCAATGGGCGCAAGCCTGACGACGCAACGCCGCGTGGAGGATGAAGGTCTTCGGATTGTAAACTCCTGTTGATCGGGACGAACAACCATCGGGATAACACCCCGGAGGTCTGACGGTACCGATTGAGGAAGCCCCGGCTAACTCTGTGCCAGCAGCCGCGGTAATACAGAGGGGGCAAGCGTTGTTCGGAATTACTGGGCGTAAAGGGCGCGTAGGCGGCCGCCTAAGTCAGACGTGAAATCCCTCGGCTCAACCGGGGAACTGCGTCTGATACTGGGTGGCTTGAATCCGGGAGAGGGATGCGGAATTCCAGGTGTAGCGGTGAAATGCGTAGATATCTGGAGGAACACCGGTGGCGAAGGCGGCATCCTGGACCGGCATTGACGCTGAGGCGCGAAAGCCAGGGGAGCAAACGGG

>OTU428GTAGGGAATATTGGACAATGGGTGCAAGCCTGATCCAGCCATGCCGCGTGCAGGAAGAAGGCCTTCTGGGTTGTAAACTGCTTTTACCAGGGGATAAAAAGCCCATGCGTGGGAAATTGAAGGTACCTGGTGAATAAGCCACGGCTAACTACGTGCCAGCAGCCGCGGTAATACGTAGGTGGCAAGCGTTGTCCGGATTTATTGGGTTTAAAGGGTGCGTAGGTGGTCTATTAAGTCAGTGCTGAAATATCCCGGCTTAACCGGGAGGGTGGCATTGATACTGATGGACTTGAGTAGAGTCGAGGTAGGCGGAATTGACGGTGTAGCGGTGAAATGCTTAGATATCGTCAAGAACACCTATAGCGAAGGCAGCTTACTAGGCTCTAACTGACACTGAGGCACGAAAGTGTGGGGATCAAACAGG

>OTU429GTGGGGAATATTGGACAATGGGGGAAACCCTGATCCAGCCATGCCGCGTGAGTGATGAAGGCCTTCGGGTTGTAAAACTCTTTCGACGGGGACGATAATGACGGTAACCGTAGAAGAAGCTCCGGCTAACTTCGTGCCAGCAGCCGCGGTAATACGAAGGGGGCTAGCGTTGTTCGGAATTACTGGGCGTAAAGCGCGCGCAGGCGGCTATCCAAGTCAGTGGTGAAAGCCCGGAGCTCAACTCCGGAACTGCCATTGAAACTGTTTAGCTTGAGGATGAGAGAGGTGAGTGGAATTCCCAGTGTAGAGGTGAAATTCGTAGATATTGGGAAGAACACCGGTGGCGAAGGCGGCTCACTGGCTCATTACTGACGCTCAGGCGCGATAGCGTGGGGATCAAACAGG

>OTU430GTGGGGAATATTGGACAATGGGCGAAAGCCTGATCCAGCCACGCCGCGTGAGTGATGAAGGCCTTCGGGTCGTAAAGCTCTGTGGGGAGGGACGAATATCCCGGCGTAGAGCCGGGGGTGACGGTACCTCCTTAGCAAGCACCGGCTAACTTCGTGCCAGCAGCCGCGGTAATACGAAGGGTGCAAACGTTGCTCGGAATTATTGGGCGTAAAGGGCGCGTAGGCGGCCTAGCAAGTGGCGGGTGAAATCCCTCGGCTTAACCGAGGAACTGCCTGCCAGACTGCCTGGCTCGAGACCGGGAGAGGTGAGTGGAATTCCCAGTGTAGCGGTGAAATGCGTAGATATTGGGAGGAACACCAGTGGCGAAGGCGGCTCACTGGACCGGAACTGACGCTGAGGCGCGAAAGCGTGGGGAGCAAACAGG

>OTU431GTGGGGAATCTTGCGCAATGGGCGAAAGCCTGACGCAGCGACGCCGCGTGAGGGAAGAAGGCCTTCGGGTTGTAAACCTCTTTCAGGAGGGACGAAGCTACTCGGGTGAATAGCCCAGAGGGTGACGGTACCTCCAGAAGAAGCCCCGGCTAACTACGTGCCAGCAGCCGCGGTAATACGTAGGGGGCAAGCGTTGTCCGGATTTACTGGGCGTAAAGAGCGTGTAGGCGGCCTGACAGGTCTGTTGTGAAAACTGGAGGCTCAACCTCCAGACGTCGACAGAAACCGTCAGGCTAGAGTCCGGAAGAGGAGAGTGGAATTCCTGGTGTAGCGGTGAAATGCGCAGATATCAGGAAGAACACCGATGGCGAAGGCAGCTCTCTGGGACGGTACTGACGCTGAGACGCGAAAGCGTGGGGAGCGAACAGG

>OTU432GTGAGGGATATTGCACAATGGAGGAAACTCTGATGCAGCGATGCCGCGTGGAGGAAGAAGGTTTTCGGATTGTAAACTCCTGTTTCGAGGGACGATAATGACGGTAGCCAAGGAGAAAGCTCCGGCTAACTACGTGCCAGCAGCCGCGGTAATACGTAGGGAGCGAGCGTTGTCCGGATTTACTGGGTGTAAAGGGTGCGCAGGCGGCTCTGCAAGTCAGGCGTGAAAGGTGAGGGCTCAACCCTCAAACTGCGCTTGAAACTGTGGGGCTTGAGTGAAGTAGAGGCAGGCGGAATTCCCGGTGTAGCGGTGAAATGCGTAGAGATCGGGAGGAACACCAGTGGCGAAGGCGGCCTGCTGGGCTTTAACTGACGCTCAGGCACGAAAGCATGGGTAGCAAACAGG

>OTU433GTGAGGAATATTGGACAATGGGTGGAAGCCTGATCCAGCCATCCCGCGTGTAGGAAGACGGCCTTATGGGTTGTAAACTACTTTTATCTGGGGATAAACCTATCTACGAGTAGATAGCTGAAGGTACCAGAAGAATAAGCACCGGCTAACTCCGTGCCAGCAGCCGCGGTAATACGGAGGGTGCAAGCGTTATCCGGATTTATTGGGTTTAAAGGGTCCGTAGGCGGATTAATCAGTCAGTGGTGAAATCTCATAGCTTAACTATGAAACTGCCATTGATACTGTTAGTCTTGAGTAATGTTGAAGTTGCTGGAATGTGTAGTGTAGCGGTGAAATGCTTAGATATTACGCAGAACACCAATTGCGAAGGCAGGTGACTAAACATTAACTGACGCTGATGGACGAAAGCGTGGGGAGCGAACAGG

>OTU434GTGGGGGATATTGCACAATGGGGGAAACCCTGATGCAGCGACGCCGCGTGAGTGAAGAAGTATTTCGGTATGTAAAGCTCTATCAGCAGGGAAGAAAATGACAGTACCTGACTAAGAAGCCCCGGCTAACTACGTGCCAGCAGCCGCGGTAATACGTAGGGGGCAAGCGTTATCCGGATTTACTGGGTGTAAAGGGAGCGTAGACGGTTATGTAAGTCTGGAGTGAAAGCCCGGGGCCCAACCCCGGGACTGCTTTGGAAACTGTGTAACTAGAGTACAGGAGGGGCAGGCGGAATTCCTGGTGTAGCGGTGAAATGCGTAGATATCAGGAGGAACACCGGCGGCGAAGGCGGCCTGCTGGACTGAAACTGACGTTGAGGCTCGAAAGCGTGGGGAGCAAACAGG

>OTU435GTGGGGAATATTGGACAATGGGCGCAAGCCTGATCCAGCAATGCCGCGTGTGTGAAGAAGGCCTTCGGGTTGTAAAGCACTTTTATCAGGAGCGAAATACTACGGGTTAATAACCCGTGGGGCTGACGGTACCTGAGGAATAAGCACCGGCTAACTTCGTGCCAGCAGCCGCGGTAATACGAAGGGTGCAAGCGTTAATCGGAATTACTGGGCGTAAAGGGTGCGTAGGCGGTTCGTTAAGTCTGTTGTGAAATCCCCGGGCTCAACCTGGGAATGGCAATGGATACTGGCGAGCTAGAGTGTGTCAGAGGATGGTGGAATTCCCGGTGTAGCGGTGAAATGCGTAGAGATCGGGAGGAACATCAGTGGCGAAGGCGGCCATCTGGGACAACACTGACGCTGAAGCACGAAAGCGTGGGGAGCAAACAGG

>OTU436GTAAGGAATATTGCACAATGGGCGCAAGCCTGATGCAGCAACGCCGCGTGCGCGACGAAGGCCTTCGGGTCGTAAAGCGCTTTTCGGGGAGATGAGCACGGACAGTATCCCCGGAAGAAGGATCGGCTAACTACGTGCCAGCAGCCGCGGTAAAACGTAGGATCCGAGCGTTATCCGAATTCACTGGGCGTAAAGCGCATGCAGGCGGCCGCACAAGTTGGATGTGAAATCTCCCGGCTCAACTGGGAGGGGACGTTCAAAACTGTGCGGCTCGAGGGCGGTAGAGGAAGGTAGAATTCCCGGTGTAGTGGTGAAATGCGTAGATATCGGGAGGAATACCCGTGGCGAAGGCGACCTTCTGGACCGTTTCTGACGCTCAGATGCGAAAGCTAGGGGAGCGAACGGG

>OTU437GTGGGGAATCTTGCGCAATGGGCGAAAGCCTGACGCAGCGACGCCGCGTGAGTGATGAAGGCCTTCGGGTTGTAAAGCTCTGTGGGGAGAGAAGAATTAGTGCAGCCTAATACGCTGCATGATGACGGTATCTCCTTAGCAAGCACCGGCTAACTCTGTGCCAGCAGCCGCGGTAAGACAGAGGGTGCAAACGTTGTTCGGAATTACTGGGCGTAAAGCGAGTGTAGGCGGTCTGAAAAGTCAGGTGTGAAAGCCCTGGGCTCAACCCAGGAAGTGCACTTGAAACTGCCAGGCTAGAGTACTGGAGAGGTTGGTGGAATTCTCGGTGTAGAGGTGAAATTCGTAGATATCGAGAGGAACACCGGTGGCGAAGGCGGCCAACTGGACAGATACTGACGCTCAAACTCGAAAGCGTGGGGAGCAAACAGG

>OTU438GTGGGGAATCTTCCGCAATGGGCGAAAGCCTGACGGAGCAACGCCGCGTGAGTGAAGACGGCCTTCGGGTTGTAAAGCTCTGTTATACGGGACGAATAATCTTGTGGTTAATACCCATAAGAAGTGACGGTACCGTAAGAGAAAGCCACGGCTAACTACGTGCCAGCAGCCGCGGTAATACGTAGGTGGCAAGCGTTGTCCGGAATTATTGGGCGTAAAGGGCGCGCAGGCGGCTTCTTAAGTCTGTCTTAAAAGTGCGGGGCTTAACCCCGTGATGGGATGGAAACTGGGAAGCTCAGAGTATCGGAGAGGAAAGCGGAATTCCTAGTGTAGCGGTGAAATGCGTAGATATTAGGAGGAACACCAGTGGCGAAAGCGGCTTTCTGGACGAAAACTGACGCTGAGGCGCGAAAGCCAGGGGAGCGAACGGG

>OTU439GTGGGGAATTTTGCACAATGGGGGAAACCCTGATGCAGCGACGCCGCGTGATTTAGAAGGCCTTCGGGTTGTAAAAATCTTTTGTATAGGAAGAAAATGACAGTACTATACGAATAAGGTCCGGCTAATTACGTGCCAGCAGCCGCGGTAATACGTAAGGACCGAGCGTTGTCCGGAATCATTGGGCGTAAAGGGTACGTAGGCGGCTAGAAAAGTTAGAAGTCAAAGGCTATAGCTCAACTATAGTAAGCTTCTAAAACTATTTAGCTTGAGAAATGGAAGGGAAAGTGGAATTCCTAGTGTAGCGGTGGAATGCGCAGATATTAGGAAGAATACCGGTGGCGAAGGCGACTTTCTGGCCATTATCTGACGCTGAGGTACGAAAGCGTGGGTAGCAAACAGG

>OTU440GTGGGGAATCTTCCGCAATGGGCGAAAGCCTGACGGAGCAATGCCGCGTGAGTGAAGAAGGCCTTCGGGTTGTAAAGCTCTGTCCTAAGGGAAGAATGTACCTTATGTGAATAATATGAGGTAATGACGGTACCTTAGGAGGAAGCCCCGGCTAACTACGTGCCAGCAGCCGCGGTAATACGTAGGGGGCGAGCGTTGTCCGGAATTACTGGGCGTAAAGCGCGCGTAGGCGGGTTATTAAGTCAGATGTGAAAACTCTGGGCTCAACCGAGAGCATGCATCTGATACTGGTAATCTTGAGTGTAGAAGAGGAGAGTGGAATTCCTAGTGTAGCGGTGAAATGCGTAGATATTAGGAGGAACACCAGTGGCGAAGGCGACTCTCTGGTCTATAACTGACGCTGAGGCGCGAAAGCGTGGGGAGCAAACAGG

>OTU441GTGGGGAATATTGCACAATGGGGGAAACCCTGATGCAGCAACGCCGCGTGAAGGATGAAGGTTTTCGGATCGTAAACTTCTATCAACAGGGACGAAACAAATGACGGTACCTGAATAAGAAGCCCCGGCTAACTACGTGCCAGCAGCCGCGGTAATACGTAGGGGGCAAGCGTTATCCGGAATTACTGGGTGTAAAGGGAGCGTAGGCGGCACGTCAAGCCAGATGTGAAAGCCCGGGGCTTAACCCCGTGGATTGCATTTGGAACTGGCGAGCTAGAGTACAGGAGAGGAAAGCGGAATTCCTAGTGTAGCGGTGAAATGCGTAGATATTAGGAAGAACACCAGTGGCGAAGGCGGCTTTCTGGACTGAAACTGACGCTGAGGCTCGAAAGCGTGGGGAGCAAACAGG

>OTU442GTGAGGAATATTGGTCAATGGGCGTGAGCCTGAACCAGCCAAGCCGCGTGAGGGAAGAAGGCGCCAGGCGTCGTAAACCTCTTTTGCCGGGGAACAAAGGGCGCCACGTGTGGCGTTGTGAGTGTACCCGGAGAAAAAGCATCGGCTAACTCCGTGCCAGCAGCCGCGGTAATACGGAGGATGCGAGCGTTATCCGGATTTATTGGGTTTAAAGGGTGCGTAGGCGGACCGGAAAGTCAGCGGTAAAAGCCCGGGGCTCAACCCCGGCGAGCCGTTGAAACTGCCGGACTAGAGACGGCGAGAAGTACGCGGAATGCGCGGTGTAGCGGTGAAATGCATAGATATCGCGCAGAACTCCGATTGCGAAGGCAGCGTACCGGCGCCGGACTGACGCTGAGGCACGAAAGCGTGGGTATCGAACAGG

>OTU443GTGGGGAATATTGCACAATGGGCGAAAGCCTGATGCAGCAACGCCGCGTGAAGGAAGAAGGTCTTCGGATCGTAAACTTCTGTCCTTGGGGAAGATAATGACGGTACCCTTGGAGGAAGCCCCGGCTAACTACGTGCCAGCAGCCGCGGTAATACGTAGGGGGCAAGCGTTATCCGGAATTATTGGGCGTAAAGAGTGCGTAGGTGGTTACTTAAGCGCGGGGTTTAAGGCAATGGCTCAACCATTGTTCGCCCTGCGAACTGGGTTACTTGAGTGCAGGAGAGGAAAGCGGAATTCCTAGTGTAGCGGTGAAATGCGTAGATATTAGGAGGAACACCAGTGGCGAAGGCGGCTTTCTGGACTGTTACTGACACTGAGGCACGAAAGTGTGGGGAGCAAACAGG

>OTU444GTGGGGAATTGTTCGCAATGGGCGCAAGCCTGACGACGCAACGCCGCGTGGAGGATGAAGGTCTTCGGATTGTAAACTCCTGTTGACCGGGACGAATGGGCCTCGGGCTAATCACCGAGGCAGTGACGGTACCGGTTGAGGAAGCCACGGCTAACTCTGTGCCAGCAGCCGCGGTAATACAGAGGTGGCAAGCGTTGTTCGGAATTACTGGGCGTAAAGGGCGCGTAGGCGGCCTTCTCAGTCAGACGTGAAATCCCCGGGCTTAACTCGGGAACTGCGTCTGATACTGGAAGGCTGGAGTTCGGGAGAGGGATGTGGAATTCCAGGTGTAGCGGTGAAATGCGTAGATATCTGGAGGAACACCGGTGGCGAAGGCGGCATCCTGGACCGAAACTGACGCTGAGGCGCGAAAGCCAGGGGAGCAAACGGG

>OTU445GTGGGGGATATTGGACAATGGGGGGAACCCTGATCCAGCGACGCCGCGTGAGTGAAGAAGTATCTCGGTATGTAAAGCTCTGTCAGCAGGGAAGAAAGAAATGACGGTACCTGACCAAGAAGCCCCGGCTAACTACGTGCCAGCAGCCGCGGTAATACGTAGGGGGCAAGCGTTATCCGGAATCACTGGGTGTAAAGGGAGCGTAGACGGTTGTGCAAGTCTGGAGTGAAAGGCGGGGGCCCAACCCCCGGACTGCTCTGGAAACTGTGTAACTGGAGTGCAGGAGAGGCAGGCGGAATTCCTGGTGTAGCGGTGAAATGCGTAGATATCAGGAGGAACACCGGTGGCGAAGGCGGCCTGCTGGACTGTAACTGACGTTGAGGCTCGAAAGCGTGGGGAGCAAACAGG

>OTU446GTGGGGGATATTGCACAATGGGGGGAACCCTGATGCAGCGACGCCGCGTGGAGGAAGAAGTATCTCGGTATGTAAACTCCTATCAGCAGGGAAGAAGACAGACGGTACCTGACTAAGAAGCCCCGGCTAACTACGTGCCAGCAGCCGCGGTAATACGTAGGGGGCGAGCGTTATCCGGATTTACTGGGTGTAAAGGGAGCGTAGACGGCCATGCAAGCTCGGTGTGAAAGGTGCGGGCATAACCCGCAGACTGCACTGAGAACTGTATGGCTGGAGTGCTGGAGGGGCAGGCGGAATTCCTGGTGTAGCGGTGAAATGCGTAGATATCAGGAGGAACACCGGTGGCGAAGGCGGCCTGCTGGACAGCGACTGACGTTGAGGCTCGAGAGCGTGGGGAGCGAACAGG

>OTU447GTGAGGAATATTGGTCAATGGCCGGAAGGCTGAACCAGCCAAGTCGCGTGAGGGAATAAGGCCCTACGGGTCGTAAACCTCTTTTGTCAGGGAGCAAGGCCGTCCACGTGTGGGCGGAAGGAGAGTACCTGAAGAAAAAGCATCGGCTAACTCCGTGCCAGCAGCCGCGGTAATACGGAGGATGCGAGCGTTATCCGGATTTATTGGGTTTAAAGGGTGCGTAGGCGGCCTGCCAAGTCAGCGGTAAAATTGCGGGGCTCAACCCCGTAGAGCCGTTGAAACTGACGGGCTCGAGTGGGCGAGAAGTATGCGGAATGCGTGGTGTAGCGGTGAAATGCATAGATATCACGCAGAACCCCGATTGCGAAGGCAGCATACCGGCGCCCTACTGACGCTGAGGCACGAAAGTGCGGGGATCAAACAGG

>OTU448GTGAGGAATATTGGTCAATGGGCGTGAGCCTGAACCAGCCAAGCCGCGTGAAGGAAGAAGGTGCAGGGCATCGTAAACTTCTTTTGCCGGGGAACAATAAGCGGGACTAGTCCCGCGACGAGTGTACCCGGAGAAAAAGCATCGGCTAACTCCGTGCCAGCAGCCGCGGTAATACGGAGGATGCGAGCGTTATCCGGATTTATTGGGTGTAAAGCGTGTGTAGGCGGTGATATAAGTCTAAGGTTTAAGCCCGGAGCTCAACTCCGGATCGCCTTAGAAACTGTAACACTTGAGTATGGTAGAGGTAAACGGAATTTCTAGTGTAGCGGTAAAATGCGTAGATATTAGAAGGAACACCAGTGGCGAAGGCGGTTTACTGGGCCACCACTGACGCTGAGACACGAAAGCGTGGGGAGCAAATAGG

>OTU449GTGGGGAATATTGGACAATGGGCGAAAGCCTGATCCAGCAATACCGCGTGTGTGAAGAAGGCCCTAGGGTTGTAAAGCACTTTTGTTAGGGAGAAAGGGTTTTGTAGTTAATACCTACAGGATCTGATGTTACCTAAAGAATAAGCACCGGCTAACTCCGTGCCAGCAGCCGCGGTAATACGGAGGGTGCAAGCGTTAATCGGAATTACTGGGCGTAAAGGGCGCGTAGGTGGTAACTTAAGTTGGGTGTGAAATCCCCGGGCTCAACCTGGGAATTGCATCCAAAACTGGGTTGCTAGAGTGTGGTAGAGGGTAGCGGAATTTCTGGTGTAGCGGTGAAATGCGTAGATATCAGAAGGAACATCAATGGCGAAGGCAGCTACCTGGGCCAACACTGACGCTGAGGCGCGAAAGCGTGGGGAGCAAACAGG

>OTU450GTGGGGAATATTGCACAATGGGCGCAAGCCTGATGCAGCAACGCCGCGTGAACGAAGAAGGTCTTCGGATTGTAAAGTTCTGTCCTTAGGGAAGAAGGAAGTGACGGTACCTAAGGAGGAAGCCCCGGCTAACTACGTGCCAGCAGCCGCGGTAATACGTAGGGGGCAAGCGTTATCCGGAATGATTGGGCGTAAAGAGTACGTAGGTGGTTTCGTAAGCGCGAGGTGAAAGGCAGTGGCTTAACCATTGTAAGCCTTGCGAACTGCGAGACTTGAGTGCAGGAGAGGAAAGCGGAATTCCTAGTGTAGCGGTGAAATGCTTAGATATTAGGAGGAACACCAGTGGCGAAGGCGGCTTTCTGGACTGTAACTGACACTGAGGTACGAAAGCGTGGGGAGCAAACAGG

>OTU451GTTAGGAATATTCGTCAATGGGGGAAACCCTGAACGAGCAATGCCGCGTGAACGATGAAGGCCCTTCGGGTTGTAAAGTTCTGTTGTTTGGAAAGAATATCGAGTATAGGAAATGATACTCGACTGACGGTACCATTCAAGAAAGCCACGGCTAACTACGTGCCAGCAGCCGCGGTAATACGTAGGTGGCGAGCGTTATCCGGATTTATTGGGCGTAAAGCGTCCGCAGCCGGTTTATTAAGTCTAAGACTAAAGCCCGAAGCTCAACTTCGGTTCGTTTTAGAAACTGGTAGACTCGAGTGTGGTAGAGGCAAGTGGAACTTCTAGTGTAGCGGTAAAATGCGTAGATATTAGAAAGAACACCAGTGGCGAAGGCGACTTGCTGGGCCATTACTGACGGTCAGGGACGAAAGCGTGGGGAGCAAATAGG

>OTU452GTGAGGAATATTGGTCAATGGACGCAAGTCTGAACCAGCCAAGTAGCGTGAAGGATGACTGCCCTATGGGTTGTAAACTTCTTTTATAAGGGAATAAAGTGAGGGACGTGTCCCTTTTTGTATGTACCTTATGAATAAGGATCGGCTAACTCCGTGCCAGCAGCCGCGGTAATACGGAGGATCCGAGCGTTATCCGGATTTATTGGGTTTAAAGGGAGCGTAGGTGGACTGGTAAGTCAGTTGTGAAAGTTTACGGCTCAACCGTGAAATTGCAGTTGATACTGTATGTCTTGAGTGTACAAGAGGTGGGCGGAATTCGTGGTGTAGCGGTGAAATGCTTAGATATCACGAAGAACTCCAATTGCGAAGGCAGCTCACTGGGGTACAACTGACACTGAGGCTCGAAAGTGTGGGTATCAAACAGG

>OTU453GTGGGGAATTTTGGACAATGGACGCAAGTCTGATCCAGCCATGCCGCGTGCGGGAAGAAGGCCTTCGGGTTGTAAACCGCTTTTGTCAGGGAAGAAAAGCTCCGAACTAATACTTTGGGGTGATGACGGTACCTGAAGAATAAGCACCGGCTAACTACGTGCCAGCAGCCGCGGTAATACGTAGGGTGCAAGCGTTAATCGGAATTACTGGGCGTAAAGCGTGCGCAGGCGGTTCTATAAGACAGATGTGAAATCCCCGGGCTCAACCTGGGAACTGCATTTGTGACTGTAGAGCTGGAGTGCGGCAGAGGGGGATGGAATTCCGCGTGTAGCAGTGAAATGCGTAGATATGCGGAGGAACACCGATGGCGAAGGCAATCCCCTGGGCCTGCACTGACGCTCATGCACGAAAGCGTGGGGAGCAAACAGG

>OTU454GTGGGGAATATTGCACAATGGGGGAAACCCTGATGCAGCGACGCCGCGTGAGCGAAGAAGGCCTTCGGGTCGTAAAGCTCTGTCCTAAGGGAAGAAAAAGACGGTACCTTAGGAGGAAGCCCCGGCTAACTACGTGCCAGCAGCCGCGGTAATACGTAGGGGGCGAGCGTTATCCGGAATTACTGGGCGTAAAGGGTGCGTAGGCGGCCTTTTAAGTCAGATGTGAAAGACTACGGCTTAACCGTAGTTAGCATTTGAAACTAAAAGGCTTGAGTGCAGGAGAGGAGAGTGGAATTCCTAGTGTAGCGGTGAAATGCGTAGATATTAGGAGGAACACCAGTGGCGAAGGCGACTCTCTGGACTGCAACTGACGCTGAGGCACGAAAGCGTGGGTAGCGAACAGG

>OTU455GTGAGGAATATTGGTCAATGGGCGCATGCCTGAACCAGCCAAGTCGCGTGAGGGACGACGGCCCTACGGGTTGTAAACCTCTTTTGCCGGGGAGCAAGGCTTTCCACGAGTGGAAAGCCGGAGAGTACCCGGAGAAAAAGCATCGGCTAACTCCGTGCCAGCAGCCGCGGTAATACGGAGGATGCGAGCGTTATCCGGATTTATTGGGTTTAAAGGGTGCGTAGGCGGGATGCCAAGTCAGCGGTAAAATCGCGGGGCTCAACCCCGCTCCGCCGTTGAAACTGGCGTTCTTGAGTGGGCGAGAAGTATGCGGAATGCGTGGTGTAGCGGTGAAATGCATAGATATCACGCAGAACTCCGATTGCGAAGGCAGCATGCCGGCGCCCAACTGACGCTGAAGCACGAAAGCGTGGGTATCGAACAGG

>OTU456GTGGGGAATATTGCACAATGGGGGAAACCCTGATGCAGCGACGCCGCGTGAGCGAAGAAGTATTTCGGTATGTAAAGCTCTATCAGCAGGGAAGAAAATGACGGTACCTGACTAAGAAGCTCCGGCTAAATACGTGCCAGCAGCCGCGGTAATACGTATGGAGCAAGCGTTATCCGGATTTACTGGGTGTAAAGGGCGTGTAGGCGGGCATGCAAGCCAGAAGTGAAATCTGGGGGCTTAACCCCCAAACTGCTTTTGGAACTGCGTGTCTTGAGTGATGGAGAGGCAGGCGGAATTCCCAGTGTAGCGGTGAAATGCGTAGATATTGGGAGGAACACCAGTGGCGAAGGCGGCCTGCTGGACATTAACTGACGCTGAGGCGCGAAAGCGTGGGGAGCAAACAGG

>OTU457GTAGGGAATCTTCCACAATGGACGAAAGTCTGATGGAGCAACGCCGCGTGAGTGAAGAAGGGTTTCGGCTCGTAAAACTCTGTTATTGGAGAAGAAGGATAAGGTAAGTAACTGTACTTTATTTGACGGTAACCAACCAGAAAGTCACGGCTAACTACGTGCCAGCAGCCGCGGTAATACGTAGGTGGCAAGCGTTATCCGGAATTATTGGGCGTAAAGCGAGTGCAGGCGGTTGTATAAGTCTGATGTGAAAGCCCTCGGCTCAACCGAGGAATTGCATCAGAAACTGTATAACTTGAGTGCAGAAGAGGAGAGTGGAACTCCATGTGTAGCGGTGGAATGCGTAGATATATGGAAGAACACCAGTGGCGAAGGCGGCTCTCTGGTCTGTAACTGACGCTGAGGCTCGAAAGCATGGGTAGCGAACAGG

>OTU458GTAGGGAATATTGCTTAATGGGCGCAAGCCTGAAGCAGCAACGCCGCGTGTGCGATGAAGGCCTTCGGGTTGTAAAGCACTTTTTGCTGGGAAGAGGAAGGACGGTACCAGCAGAATAAGCCTCGGCTAACTACGTGCCAGCAGCCGCGGTAAAACGTAGGAGGCGAGCGTTATCCGGATTTACTGGGTGTAAAGCGCGTGCAGGCGGTTTGTTAAGTTGGGTGTGAAAGCTCCTGGCTCAACTGGGAGAGGTCGCTCAAGACTGGCAGACTCGAGAGCAGCAGGGGAAGGTGGAATTCCGGGTGTAGTGGTGAAATGCGTAGATATCCGGAGGAACACCAGTGGCGAAGGCGGCCTTCTGGGCTGCATCTGACGCTCAGACGCGAAAGCTAGGGGAGCAAACGGG

>OTU459GTGGGGAATCTTGCGCAATGGTCGAAAGACTGACGCAGCGACGCCGCGTGCGGGAGGAAGCCCTTCGGGGTGTAAACCGCTGTTACTCGGGACGAACCTCCTGTTTCGACAGGATTGACGGTACCGGGGGAGGAAGCACCGGCTAACTCTGTGCCAGCAGCCGCGGTAATACAGAGGGTGCGAGCGTTGTCCGGAATCACTGGGCGTAAAGGGCGCGTAGGCGGCTTCTCAAGCGTGCGGTGAAAGCTCGGGGCTTAACTCCGAGTCTGCCGTGCGAACTGGGAGGCTTGAGTCTGGTAGAGGTCGATGGAATTCCGGGTGTAGCGGTGGAATGCGTAGAGATCCGGAAGAACACCGGTGGCGAAGGCGACTCTCTGGACGGTTCCTGACGCTGAGGCGCGAAAGCTGGGGGAGCAAACAGG

>OTU460GTGGGGAATTTTGCGCAATGGACGAAAGTCTGACGCAGCGACGCCGCGTGGGTGATGAAGGCCTTCGGGTTGTAAAGCCCTGTGGGGAGGGAAGAATAAGGTATGGTTAATACCCATATTGATGACGGTACCTCCTTAGCAAGCACCGGCTAACTCTGTGCCAGCAGCCGCGGTAAGACAGAGGGTGCGAACGTTGTTCGGAATTACTGGGCGTAAAGGGCGTGTAGGCGGCTACGCGGGTCGGATGTGAAAGCCCTGGGCTTAACCTAGGAAGTGCATTCGAAACTGCGTGGCTAGAGTCCCGGAGAGGATGGTGGAATTCTCGGTGTAGAGGTGAAATTCGTAGATATCGAGAGGAACACCGGTGGCGAAGGCGGCCATCTGGACGGGTACTGACGCTGAGACGCGAAAGCGTGGGTAGCAAACAGG

>OTU461GTGGGGAATCTTGCGCAATGCGCGAAAGCGTGACGCAGCAACGCCGCGTGGGGGAAGACGGCCTTAGGGTTGTAAACCCCTTTCAGGAGGGACGAAGGCCGGCACGTTAATAGCGTGTCCGGTTGACGGTACCTCCACAAGAAGCCCCGGCTAACTACGTGCCAGCAGCCGCGGTAATACGTAGGGGGCAAGCGTTGTCCGGAATCATTGGGCGTAAAGAGCGCGTAGGCGGTCCGGTAAGTCAGCTGTGAAAGTCAAGGGCTCAACCCTTGAAAGCCGGTTGATACTGTCGGGCTAGAGTCCGGAAGAGGCGAGTGGAATTCCCGGTGTAGCGGTGAAATGCGCAGATATCGGGAGGAACACCAATGGCGAAGGCAGCTCGCTGGGACGGTACTGACGCTGAGGCGCGAAAGCGTGGGGAGCAAACAGG

>OTU462GTGGGGAATTTTGGACAATGGGCGCAAGCCTGATCCAGCCATGCCGCGTGAGTGAAGAAGGCCTTCGGGTTGTAAAGCTCTTTCGGCCGGGAAGAAATCGGCAGGGTGAATAATCCTGCTGGATGACGGTACCGGAAGAAGAAGCACCGGCTAACTACGTGCCAGCAGCCGCGGTAATACGTAGGGTGCGAGCGTTAATCGGAATTACTGGGCGTAAAGCGTGCGCAGGCGGTTGTGTAAGACAGGTGTGAAATCCCCGGGCTTAACCTGGGAATGGCGCTTGTGACTGCACGACTCGAGTTCGGCAGAGGGGGGTGGAATTCCACGTGTAGCAGTGAAATGCGTAGATATCTGGAGGAACACCGATGGCGAAGGCAGCCCCCTGGGCTAACACTGACGCTCATGCACGAAAGCGTGGGGAGCAAACAGG

>OTU463GTGGGGAATATTGCACAATGGGCGAAAGCCTGATGCAGCAACGCCGCGTGAGTGAAGAAGGTTTTCGGATTGTAAAGCTCTGTCTTTGGGGACGATAATGACGGTACCCAAGGAGGAAGCCACGGCTAACTACGTGCCAGCAGCCGCGGTAATACGTAGGTGGCGAGCGTTGTCCGGAATTACTGGGCGTAAAGAGTGCGTAGGCGGATATTTAAGTGAGATGTGAAATACCCGGGCTTAACCCGGGCACTGCATTTCAAACTGGATATCTAGAGTGCGGGAGAGGAGAATGGAATTCCTAGTGTAGCGGTGAAATGCGTAGAGATTAGGAAGAACACCAGTGGCGAAGGCGATTCTCTGGACCGTAACTGACGCTGAGGCACGAAAGCGTGGGTAGCAAACAGG

>OTU464GTGGGGAATATTGGACAATGGGGGCAACCCTGATCCAGCAATGCCGCGTGAGTGATGAAGGCCTTAGGGTTGTAAAGCTCTTTCGCACGCGACGATGATGACGGTAGCGTGAGAAGAAGCCCCGGCTAACTTCGTGCCAGCAGCCGCGGTAATACGAAGGGGGCGAGCGTTGTTCGGAATTACTGGGCGTAAAGGGCGCGTAGGCGGCCTGTTTAGTCAGAAGTGAAAGCCCCGGGCTTAACCTGGGAACGGCTTTTGATACTGGCAGGCTTGAGTTCCGGAGAGGATGGTGGAATTCCCAGTGTAGAGGTGAAATTCGTAGATATTGGGAAGAACACCGGTGGCGAAGGCGGCCATCTGGACGGACACTGACGCTGAGGCGCGAAAGCGTGGGGAGCAAACAGG

>OTU465GTTTGGAATTTTGGTCAATGGGGGAAACCCTGAACCAGCGACGCCGCGTGGACGATGAAGGTCTTCGGATTGTAAAGTCCTTTTGGCAGGGAAAAACTTTGATTGTACCTGCAGAATAAGAGGTTGCTAACTCTGTGCCAGCAGCAGCGGTAATACAGAGACCTCAAGCGTTATCCGGAATCATTGGGCGTAAAGCGTACCGATAGGTGGTCTTGAAAGTCAGATGTGAAATGACGAAGCTTAACTTCGTTACTGTCATTTGAAACTTCAGGACTAGAGGGGCAAAGAGGAAGCTGGAACAAACGGTGTAGTAGTAAAATGCGTTGATATCGTTTGGAACACCAATAGCGTAGGCAGGCTTCTGGGTGCCACCTGACACTGCTAGGACGAAAGCGTGGGGAGCGAATGGG

>OTU466GTGGGGAATATTGGACAATGGGGGCAACCCTGATCCAGCAATGCCGCGTGAGTGATGAAGGCCTTAGGGTTGTAAAGCTCTTTCGCCCGCGACGATGATGACGGTAGCGGGAGAAGAAGCCCCGGCTAACTTCGTGCCAGCAGCCGCGGTAATACGAAGGGGGCGAGCGTTGTTCGGAATTACTGGGCGTAAAGGGCGCGCAGGCGGCCCGAGAAGTCAGATGTGAAAGCCCCGGGCTCAACCTGGGAATTGCATTTGATACTCTGGGGCTGGAGTGCGGGAGAGGAGAGTGGAATTCCCAGTGTAGAGGTGAAATTCGTAGATATTGGGAAGAACACCGGTGGCGAAGGCGGCTCTCTGGACCGTAACTGACGCTGAGGCGCGAAAGCGTGGGGAGCAAACAGG

>OTU467GTGGGGAATATTGCACAATGGGGGAAACCCTGATGCAGCGACGCCGCGTGGAGGAAGAAGGTCTTCGGATTGTAAACTCCTGTCCTTGGGGACGATAATGACGGTACCCAAGGAGGAAGCACCGGCTAACTACGTGCCAGCAGCCGCGGTAAAACGTAGGGTGCAAGCGTTGTCCGGAATTACTGGGTGTAAAGGGAGCGTAGACGGCATGGCAAGTCTGAAGTGAAAGCCCGGGGCTCAACCGCGGGACTGCTTTGGAAACTGTTAAGCTGGAGTGCAGGAGAGGTAAGTGGAATTCCTAGTGTAGCGGTGAAATGCGTAGATATTAGGAGGAACACCAGTGGCGAAGGCGGTCTGCTGGACAGCAACTGACGCTGAGGCGCGAAAGCGTGGGGAGCAAACAGG

>OTU468GTTGGGGATATTGCACAATGGGGGAAACCCTGATGCAGCGACGCCGCGTGAGGGAAGAAGGTTTTCGGATTGTAAACCTCTGTCTTCGGGGACGATAATGACGGTACCCGAGGAGGAAGCCACGGCTAACTACGTGCCAGCAGCCGCGGTAATACGTAGGTGGCGAGCGTTGTCCGGAATTACTGGGTGTAAAGGGAGCGTAGGCGGTTTGGTAAGTTGAGTGTGAAATCTACCGGCTTAACTGGTAGGCTGCGCTCAAAACTACCAAACTTGAGTGAAGTAGAGGCAGGCGGAATTCCCGGTGTAGCGGTGGAATGCGTAGATATCGGGAGGAACACCAGTGGCGAAGGCGGCCTGCTGGGCTTTTACTGACGCTGATGCTCGAAAGCATGGGGAGCAAACAGG

>OTU469GTGGGGAATATTGCACAATGGGCGAAAGCCTGATGCAGCAACGCCGCGTGAGTGATGAAGGTCTTCGGATTGTAAAGCTCTGTCTTTTGGGACGATAATGACGGTACCAAAGGAGGAAGCCACGGCTAACTACGTGCCAGCAGCCGCGGTAATACGTAGGTGGCAAGCGTTGTCCGGATTTACTGGGCGTAAAGGGTGCGTAGGTGGATATTTAAGTCAGATGTGAAACCCCTGGGCTCAACTCAGGGACTGCATTTGAAACTGGATATCTTGAGTGCGGGAGAGGTAAGTGGAATTCCTAGTGTAGCGGTGAAATGCGTAGAGATTAGGAAGAACACCAGTGGCGAAGGCGACTTACTGGACCGTAACTGACACTGAGGCACGAAAGCGTGGGTAGCAAACAGG

>OTU470GTGGGGGATATTGCACAATGGGGGAAACCCTGATGCAGCGACGCCGCGTGAGTGAAGAAGTATTTCGGTATGTAAAGCTCTATCAGCAGGGACGAAAATGACGGTACCTGAGTAAGAAGCCCCGGCTAACTACGTGCCAGCAGCCGCGGTAATACGTAGGGGGCAAGCGTTATCCGGAATCACTGGGTGTAAAGGGAGCGTAGACGGCAGGGCAAGCCTGGAGTGAAAGGCGGGGGCCCAACCCCCGGACTGCTCTGGGAACTGCCCGGCTTGAGTGCAGGAGAGGTAAGTGGAATTCCTAGTGTAGCGGTGAAATGCGTAGATATTAGGAGGAACACCAGTGGCGAAGGCGGCTTACTGGACTGTAACTGACGTTGAGGCTCGAAAGCGTGGGGAGCAAACAGG

>OTU471GTTGGGAATATTGGACAATGGAGGAAACTCTGATCCAGTGACGCCGCGTGAAGGAAGAAGGTCTTCGGATTGTAAACTTATTTTATCAGGGAAGAAAAAAATGACTGTACCTGAAGAAAAAGCACCGGCTAACTACGTGCCAGCAGCCGCGGTAATACGTAGGGTGCAAGCGTTATCCGGATTTACTGGGTGTAAAGGGCGAGTAGACGGCGTTGCAAGTCAGATGTGAAAACCTAGGGCTCAACCGTAGGACTGCATCTGAAACTGTAATGCTAGAGTGCAGGAGAGGTAAGCGGAATTCCTAGTGTAGCGGTGAAATGCGTAGATATTAGGAGGAACACCAGTGGCGAAGGCGGCTTACTGGACTGTAACTGACGTTGAGGAGCGAAAGTGTGGGGAGCAAACAGG

>OTU472GTGGGGAATTTTGGACAATGGGGGAAACCCTGATCCAGCCATGCCGCGTGCGGGAAGAAGGCCTTCGGGTTGTAAACCGCTTTTGTCTGGGAAGAAAAGGCTCTCCCTAATACGGAGGGTTCTTGACGGTACCGGAAGAATAAGCACCGGCTAACTACGTGCCAGCAGCCGCGGTAATACGTAGGGTGCGAGCGTTAATCGGAATTACTGGGCGTAAAGCGTGCGCAGGCGGTTCGTTAAGTTCGATGTGAAAGCCCCGGGCTCAACCTGGGAATGGCATTGGAAACTGGCGGGCTCGAGTGTGGCAGAGGGGGGTGGAATTCCACGTGTAGCAGTGAAATGCGTAGAGATGTGGAGGAACACCGATGGCGAAGGCAGCCCCCTGGGCTAACACTGACGCTCAGGCACGAAAGCGTGGGGAGCAAACAGG

>OTU473GTAGGGAATATTGGTCAATGGGTGAGAGCCTGAACCAGCCATGCCGCGTGCAGGAAGAAGGCCTTCTGGGTTGTAAACTGCTTTTGCCAGGGGCTAAAAAGTCCATGCGTGGAAAATTGAAAGTACCTGGTGAATAAGCCACGGCTAACTACGTGCCAGCAGCCGCGGTAATACGTAGGTGGCAAGCGTTGTCCGGATTTATTGGGTTTAAAGGGTGCGTAGGCGGCCCTGTAAGTCAGTGCTGAAATATCCCGGCTTAACCGGGAGGGTGGCATTGATACTGCGGGGCTAGAGTACAGATGAGGTAGGCGGAATTGACGGTGTAGCGGTGAAATGCTTAGATATCGTCAAGAACACCTATAGCGAAGGCAGCTTACTAGGCTGTAACTGACGCTGAGGCACGAAAGTGTGGGGATCAAACAGG

>OTU474GTGAGGAATATTGGTCAATGGACGGAAGTCTGAACCAGCCATGCCGCGTGCAGGATGACGGCTCTATGAGTTGTAAACTGCTTTTGTACGAGAATAATAGTTCTTACGTGTAGGGATTTGAATGTATCGTACGAATAAGGATCGGCTAACTCCGTGCCAGCAGCCGCGGTAATACGGAGGATTCGAGCGTTATCCGGATTTATTGGGTTTAAAGGGTGCGTAGGCTGTTGGATAAGTTAGAGGTCAAATGTCGGGGCTTAACCCCGTGCCTGCCTCTGATACTGTTCGACTAGAGAGCGGATGCAGCAGGCGGAATGAGTAGTGTAGCGGTGAAATGCTTAGATATTACTCAGAACACCGATTGCGCAGGCAGCTTGCTAATCCGCATCTGACGCTGATGCACGAAAGCGTGGGTAGCGAACAGG

>OTU475GTGGGGAATATTGCACAATGGACGCAAGTCTGATGCAGCGACGCCGCGTGAGGGATGACGGCCTTCGGGTTGTAAACCTCTTTCGCCGGGGACGAAGTGCCTGTGAGGGTGTGACGGTACCTGGAGAAGAAGCGCCGGCCAACTACGTGCCAGCAGCCGCGGTAAGACGTAGGGCGCAAGCGTTGTCCGGATTTATTGGGCGTAAAGAGCTCGTAGGCGGCTTGCCGCGTCTGCCGTGAAAACCAGCAGCTTAACTGTTGGCTTGCGGTTGATACGGGTGGGCTGGAGTTCGGTAGGGGAGACTGGAATTCCTGGTGTAGCGGTGAAATGCGCAGATATCAGGAGGAACACCGGTGGCGAAGGCGGGTCTCTGGGCCGATACTGACGCTGAGGAGCGAAAGCGTGGGGAGCGAACAGG

>OTU476GTGGGGAATATTGCGCAATGGAGGCAACTCTGACGCAGCGATGCCGCGTGAGTGAGGAAGGGATTCGTCCCGTAAAGCTCTGCCGCCGGGGAAGAGAGAGGACAGTACCCGGCAAGGAAGCCCCGGCCAATCACGTGCCAGCAGCCGCGGTAAGACGTGAGGGGCGAGCGTTGTCCGGAATTACTGGGCGTAAAGGGCGCGCAGGCGGCTTTTTAGGTGAGGTGTTAAAGACCGGGGCCCAACCCCGGGCGAGCATCTCAAACCGGAGAGCTCGAGTGCTGGAGAGGCAGGCAGAATTCCTGGTGTAGCGGTGAAATGCGCAGATATCAGGAGGAATACCGGAGGCGAAGGCGGCCTGCCGGACAGACACTGACGCTCAGGCGCGAAAGCGTGGGGAGCAAACAGG

>OTU477GTGGGGAATATTGGACAATGGGCGAAAGCCTGATCCAGCCATGCCGCGTGAGTGATGAAGGCCCTAGGGTTGTAAAGCTCTTTTGTGCGGGAAGATAATGACGGTACCGCAAGAATAAGCCCCGGCTAACTTCGTGCCAGCAGCCGCGGTAATACGAAGGGGGCTAGCGTTGCTCGGAATCACTGGGCGTAAAGGGTGCGTAGGCGGGTTTCTAAGTCAGAGGTGAAAGCCTGGAGCTCAACTCCAGAACTGCCTTTGATACTGGAAGTCTTGAGTATGGCAGAGGTGAGTGGAACTGCGAGTGTAGAGGTGAAATTCGTAGATATTCGCAAGAACACCAGTGGCGAAGGCGGCTCACTGGGCCATTACTGACGCTGAGGCACGAAAGCGTGGGGAGCAAACAGG

>OTU478GTGGGGAATATTGGGCAATGGAGGAAACTCTGACCCAGCAACGCCGCGTGAGGGATGAAGGTCTTCGGATTGTAAACCTCTGTCCCATGGGACGAATAAATGACGGTACCATGGGAGGAAGCTCCGGCTAACTACGTGCCAGCAGCCGCGGTAATACGTAGGGAGCAAGCGTTGTCCGGAATTACTGGGCGTAAAGGGTGCGCAGGCGGACCTTTAAGTCAGATGTGAAAGACCGGGGCTTAACCCCGGGTGTGCATTTGAAACTGGAGGTCTTGAGGATGGCAGAGGCAGATGGAATTCCTAGTGTAGCGGTGGAATGCGTAGATATTAGGAGGAACACCAGTGGCGAAGGCGGTCTGCTGGGCCATTTCTGACGCTGAGGCACGAAAGCGTGGGGAGCAAACAGG

>OTU479GTGGGGAATTTTGGACAATGGGGGAAACCCTGATCCAGCCATGCCGCGTGCAGGATGAAGGCCTTCGGGTTGTAAACTGCTTTTGTACGGAACGAAAAGCCCTGGGTTAATACCCTGGGGTCATGACGGTACCGTAAGAATAAGCACCGGCTAACTACGTGCCAGCAGCCGCGGTAATACGTAGGGTGCAAGCGTTAATCGGAATTACTGGGCGTAAAGCGTGCGCAGGCGGTTATGTAAGACAGATGTGAAATCCCCGGGCTCAACCTGGGAACTGCATTTGTGACTGCATAGCTGGAGTGCGGCAGAGGGGGATGGAATTCCGCGTGTAGCAGTGAAATGCGTAGATATGCGGAGGAACACCGATGGCGAAGGCAATCCCCTGGGCCTGCACTGACGCTCATGCACGAAAGCGTGGGGAGCAAACAGG

>OTU480GTGAGGAATATTGGACAATGGGCGAGAGCCTGATCCAGCCATGCCGCGTGCAGGAAGACGGCCCTATGGGTTGTAAACTGCTTTTATACGGGAAGAAACACTCCTTCGTGAAGGGGCTTGACGGTACCGTAAGAATAAGGATCGGCTAACTCCGTGCCAGCAGCCGCGGTAATACGGAGGATCCAAGCGTTATCCGGAATCATTGGGTTTAAAGGGTCCGTAGGCGGGACAATAAGTCAGCGGTGAAAGTCTGTGGCTCAACCATAGAATTGCCATTGATACTGTTGTTCTTGAGTGTTTATGAAGTGGTTAGAATATGTAGTGTAGCGGTGAAATGCATAGATATTACATGGAATACCGATTGCGAAGGCAGATCACTAATAAGATACTGACGCTGATGGACGAAAGCGTGGGTAGCGAACAGG

>OTU481GTGGGGAATCTTGCGCAATGGGCGAAAGCCTGACGCAGCGACGCCGCGTGAGTGATGAAGGCCTTCGGGTTGTAAAGCTCTGTGGAGGGGGACGAATAAGGGTCGTCGAACAGACGGCTTGATGACGGTACCCCTTTAGCAAGCACCGGCTAACTCTGTGCCAGCAGCCGCGGTAAGACAGAGGGTGCAAACGTTGTTCGGAATTACTGGGCGTAAAGCGCGTGTAGGCTGCTTCGCAAGTCGGGTGTGAAAGCCCTGGGCTCAACCTAGGAAGTGCACTCGAAACTGCGGAGCTGGAGTCCTGGAGAGGAAGGCGGAATTCTCGGTGTAGAGGTGAAATTCGTAGATATCGAGAGGAACACCGGTGGCGAAGGCGGCCTTCTGGACAGTGACTGACGCTGAGACGCGAAAGCGTGGGGAGCAAACAGG

>OTU482GTTAGGAATATTGGACAATGGGCGAAAGCCTGATCCAGCCATGCCGCGTGTGTGACGAAGGCCTTAGGGTTGTAAAGCACTTTCATCTGCGAAGATAATGACAGTAACAGAAGAAGAAGCTCCGGCTAAATTCGTGCCAGCAGCCGCGGTAATACGAATGGAGCGAGCGTTGTTAGGAATCACTGGGCGTAAAGCGTGCGCAGGCGGTTGTGAAAGTCAGGAGTGAAATCCCAGGGCTCAACCTTGGAATTGCTTTTGAAACTTCACGACTAGAGTACTGGAGGGGTTGGCGGAATTTCGAGTGTAGCAGTGAAATGCGTAGATATTCGAAGGAACACCGATGGCGTAGGCAGCCAACTGGACAGTAACTGACGCTCATGCACGAAAGTATGGGGAGCAAACAGG

>OTU483GTAGGGAATATTGCATAATGGGCGAAAGCCTGATGCAGCAACGCCGCGTGCGCGATGAAGGCCTTCGGGTCGTAAAGCGCTTTTTGAGGGGATGAGGAAGGACAGTACCCTCAGAATAAGTCTCGGCTAACTACGTGCCAGCAGCCGCGGTAAAACGTAGGAGGCAAGCGTTATCCGGATTTACTGGGCGTAAAGCGCGTGTAGGCGGTTCGTTAAGTTGGATGTGAAAGCTCCCGGCTCAACTGGGAGAGGTCGTTCAAGACTGGCCGACTGGAGAGCAGGATGGGAAGGTGGAATTCCGGGTGTAGTGGTGAAATGCGTAGATATCCGGAGGAACACCAGTGGCGAAAGCGGCTTCCTGGACTGAAACTGACGCTCAGACGCGAAAGCTAGGGTAGCAAACGGG

>OTU484GTAAGGAATATTGCGCAATGGGCGAAAGCCTGACGCAGCGACGCCGCGTGGGGGATGAAGGTCTTCGGATTGTAAACCCCTTTCGGGAGGGAAGATGGAATAGGGTAACCTATTCGGACGGTACCTCCAGAAGCAGCCACGGCTAACTTCGTGCCAGCAGCCGCGGTAATACGAAGGTGGCAAGCGTTGTTCGGAATTACTGGGCGTAAAGCGTGCGTAGTCGGTATTGAGAGTCACGGGTGAAAGCCCCACGCTTAACGTGGGAACTGCCTGTGAGACCTCAGTACTAGAGTGTGAGAGGGGATAGTGGAATACCAAGTGTAGCGGTGAAATGCGTAGAGATTTGGTGGAACACCGGTGGCGAAGGCGGCTATCTGGCTCACAACTGACGATCAGGCACGAAAGCGTGGGGAGCAAACAGG

>OTU485GTAAGGAATATTGGTCAATGGACGCAAGTCTGAACCAGCCATGCCGCGTGGAGGATTAAGGCCCTCTGGGTTGTAAACTCCTTTTATCTGGGAAGAAATGTACTTTTTCTTGAGTACTTGACGGTACCAGAGGAATAAGCACCGGCTAACTCCGTGCCAGCAGCCGCGGTAATACGGAGGGTGCAAGCGTTATCCGGATTCACTGGGTTTAAAGGGTGCGTAGGCGGACAGGTAAGTCAGTGGTGAAATCTCCGAGCTTAACTCGGAAACTGCCGTTGATACTATTTGTCTTGAATATTGTGGAGGTAAGCGGAATATGTCATGTAGCGGTGAAATGCTTAGATATGACATAGAACACCAATTGCGAAGGCAGCTTACTACACAACGATTGACGCTGAGGCACGAAAGCGTGGGGATCAAACAGG

>OTU486GTAGGGAATATTGCGCAATGGGGGAAACCCTGACGCAGCAACGCCGCGTGGAGGATGACACTTTTCGGAGCGTAAACTCCTTTTCTTAGGGAAGAATTCTGACGGTACCTAAGGAATAAGCACCGGCTAACTCCGTGCCAGCAGCCGCGGTAATACGGAGGGTGCAAGCGTTACTCGGAATCACTGGGCGTAAAGGGCGCGTAGGCGGATTATCAAGTCTCTTGTGAAATCTAATGGCTTAACCATTAAACTGCTTGGGAAACTGATAGTCTAGAGTGAGGGAGAGGCAGATGGAATTGGTGGTGTAGGGGTAAAATCCGTAGATATCACCAAGAATACCCATTGCGAAGGCGATCTGCTGGAACTCAACTGACGCTAAGGCGCGAAAGCGTGGGGAGCAAACAGG

>OTU487GTGGGGAATCTTGCGCAATGGGGGAAACCCTGACGCAGCGACGCCGCGTGCGGGATGAAGGCCTTCGGGTTGTAAACCGCTTTCAGCAGGGACGAAGCGAAAGTGACGGTACCTGCAGAAGAAGCCCCGGCCAACTACGTGCCAGCAGCCGCGGTAAGACGTAGGGGGCGAGCGTTGTCCGGAATCACTGGGCGTAAAGCGCGCGCAGGCGGCCCGGTAAGTCGACAGTGAAAACTCGGGGCTCAACCCCGAGCCTGCTGCCGATACTGTCGGGCTCGAGGCAGGTAGGGGAGATTGGAATTCCCGGTGTAGCGGTGAAATGCGCAGATATCGGGAGGAACACCGGTGGCGAAGGCGGATCTCTGGGCCTGTCCTGACGCTCAGGCGCGAAAGCTGGGGGAGCAAACAGG

>OTU488GTGGGGAATATTGGACAATGGGGGCAACCCTGATCCAGCAATGCCGCGTGTATGAAGAAGGTCTTCGGATTGTAAAGTACTTTCGGTAGGGACGATGATGACGGTACCTAAAGAATAAGCACCGGCTAACTACGTGCCAGCAGCCGCGGTAATACGTAGGGTGCGAGCGTTAATCGGAATTACTGGGCGTAAAGCGAGCGCAGACGGTTAATTAAGTCAGATGTGAAATCCCCGAGCTCAACTTGGGACGTGCATTTGAAACTGGTTAACTAGAGTGTGTCAGAGGGAGGTAGAATTCCACGTGTAGCAGTGAAATGCGTAGAGATGTGGAGGAATACCGATGGCGAAGGCAGCCTCCTGGGATAACACTGACGTTCATGCTCGAAAGCGTGGGTAGCAAACAGG

>OTU489GTGGGGAATATTGGGCAATGGGGGAAACCCTGACCCAGCGACGCCGCGTGAGGGAAGAAGGTTTTCGGATTGTAAACCTCTGTTCTTGGTGAAGAGAAGAAGACGGTAGCCAAGGAGGAAGCCCCGGCTAACTACGTGCCAGCAGCCGCGGTAATACGTAGGGGGCGAGCGTTGTCCGGAATGATTGGGCGTAAAGGGCGCGTAGGCGGCTGATTAAGTCTGGAGTGGAAGTCCTATTTTCAAGGTAGGAATTGCTTTGGAAACTGATCAGCTTGAGTGCAGGAGAGGTTATCGGAATTCCCGGTGTAGCGGTGAAATGCGTAGAGATCGGGAGGAACACCAGTGGCGAAGGCGGGTAACTGGACTGCAACTGACGCTGAGGCGCGAAAGTGTGGGGAGCAAACAGG

>OTU490GTGGGGAATATTGGACAATGGGCGCAAGCCTGATCCAGCCATGCCGCGTGGGTGAAGAAGGCCTTCGGGTTGTAAAGCCCTTTTGTTGGGAAAGAAAACTTTCCGGTCAATACCCGGAGAGAATGACGGTACCCAAAGAATAAGCACCGGCTAACTTCGTGCCAGCAGCCGCGGTAATACGAAGGGTGCAAGCGTTACTCGGAATTACTGGGCGTAAAGCGTGCGTAGGTGGTTCGTTAAGTCTGATGTGAAAGCCCTGGGCTCAACCTGGGAATTGCATTGGAAACTGGCGGGCTAGAGTACGGTAGAGGGTAGTGGAATTCCTGGTGTAGCAGTGAAATGCGTAGAGATCAGGAGGAACATCCGTGGCGAAGGCGACTACCTGGACCAGTACTGACACTGAGGCACGAAAGCGTGGGGAGCAAACAGG

>OTU491GTGGGGAATATTGGACAATGGGCGCAAGCCTGATCCAGCCATGCCGCGTGTGTGAAGAAGGCCTTCGGGTTGTAAAGCACTTTTGTCCGGGAAGAAAAGCCACGGGTTAATACCTTGGGGTTCTGACGGTACCGGAAGAATAAGCACCGGCTAACTTCGTGCCAGCAGCCGCGGTAATACGAAGGGTGCAAGCGTTACTCGGAATTACTGGGCGTAAAGCGTGCGTAGGTGGTTCGTTAAGTCTGCCGTGAAAGCCCCGGGCTCAACCTGGGAATGGCGGTGGATACTGGCGGACTAGAGTGCGGTAGAGGGTGGTGGAATTCCCGGTGTAGCAGTGAAATGCGTAGAGATCGGGAGGAACATCTGTGGCGAAGGCGGCCACCTGGACCAGCACTGACACTGAGGCACGAAAGCGTGGGGAGCAAACAGG

>OTU492GTGGGGAATATTGCACAATGGGGGAAACCCTGATGCAGCGACGCCGCGTGAAGGAAGAAGTATCTCGGTATGTAAACTTCTATCAGCAGGGAAGATAATGACGGTACCTGACTAAGAAGCCCCGGCTAACTACGTGCCAGCAGCCGCGGTAATACGTAGGGGGCAAGCGTTATCCGGATTTACTGGGTGTAAAGGGAGCGTAGACGGCATGGCAAGTCTGATGTGAAAGGCAGGGGCTCAACTCCTGGACTGCATTGGAAACTGCCGGGCTTGAGTGCCGGAGGGGTAAGCGGAATTCCTAGTGTAGCGGTGAAATGCGTAGATATTAGGAGGAACACCAGTGGCGAAGGCGGCTTACTGGACGGTAACTGACGTTGAGGCTCGAAAGCGTGGGGAGCAAACAGG

>OTU493GTGGGGAATCTTGCACAATGGGCGAAAGCCTGATGCAGCAACGCCGCGTGCGGGAAGAAGGCCCTCGGGTTGTAAACCGCTTTCAGAAGGAACGAAACTGACGGTACTTTCAGAAGAAGGTGCGGCCAACTACGTGCCAGCAGCCGCGGTGACACGTAGGCACCAAGCGTTGTCCGGATTTATTGGGCGTAAAGAGCTCGTAGGCGGTTCAGTAAGTCGGGTGTGAAAACTTTGGGCTTAACCCAAAGCGTGCATCCGATACTGCTGTGACTTGAGTACGGTAGGGGAGCGGGGAATTCCTAGTGTAGCGGTGAAATGCGCAGATATTAGGAGGAACACCGGTGGCGAAGGCGCCGCTCTGGGCCGAAACTGACGCTGAGGAGCGAAAGCATGGGTAGCAAACAGG

>OTU494GTGGGGAATCTTGCACAATGGGGGAAACCCTGATGCAGCGACGCCGCGTGAGCGATGAAGCCCTTCGGGGTGTAAAGCTCTTTCGGCAGGAACGATTATGACGGTACCTGCAGAAGAAGCTGCGGCTAACTACGTGCCAGCAGCCGCGGTAATACGTAGGCAGCAAGCGTTGTTCGGAATTACTGGGCGTAAAGAGTGTGTAGGCGGTTCTCTAAGTTCCGTGTGAAATCTCCCGGCTTAACTGGGAGGGTGCGCGGGAAACTGGAGGGCTGGAGTATGGGAGAGGAAAGCGGAATTCCTGGTGTAGCGGTGAAATGCGTAGATATCAGGAGGAACACCTGCGGTGTAGACAGCTTTCTGGACCATCACTGACGCTGAGACACGAAAGCGTGGGTAGCAAACAGG

>OTU495GTCGGGAATTTTGCTCAATGGGCGCAAGCCTGAAGCAGCAACGCCGCGTGAGGGATGAAGGCCTTCGGGTTGTAAACCTCTTTTTCCAGGGACGATGATGACGGTACCTGAAGAATAAGTCACGGCTAACTACGTGCCAGCAGCCGCGGTAATACGTAGGTGACCAGCGTTGTCCGGATTTACTGGGCGTAAAGAGCGCGCAGGCGGTCGTTCAAGTCGAGTGTGAAAGCCCCCGGCTCAACTGGGGAGGGTCACTCGATACTGATCGACTTGAAGGCAGGAGAGGGAAGCGGAATTCCCGGTGTAGTGGTGAAATGCGTAGATATCGGGAGGAACATCAGTGGCGAAGGCGGCCATCTGGATCAACACTGACGCTGAGGCACGAAAGCGTGGGGAGCAAACAGG

>OTU496GTGAGGAATATTGGTCAATGGGCGAGAGCCTGAACCAGCCAAGTCGCGTGAGGGAAGACGGTCCTATGGATTGTAAACCTCTTTTGTCGGGGAGCAAAAAGGTCTCGTGAGACCATAAGCGAGAGTACCCGAAGAAAAAGCATCGGCTAACTCCGTGCCAGCAGCCGCGGTAATACGGAGGATGCGAGCGTTATCCGGATTTATTGGGTTTAAAGGGTGCGTAGGCGGACTGGTAAGTCAGCGGTTAAACTGCGGAGCTCAACTCCGTATTGCCGTTGAAACTGTCGGTCTTGAGTGAGCGAGAAGTATGCGGAATGCGTGGTGTAGCGGTGAAATGCATAGATATCACGCAGAACTCCGATTGCGAAGGCAGCATACCGGCGCTCAACTGACGCTGAAGCACGAAAGCGTGGGGATCGAACAGG

>OTU497GTGGGGAATATTGCACAATGGGGGGAACCCTGATGCAGCGACGCCGCGTGGGTGAGGAAGTACTTCGGTATGTAAAGCCCTATCAGCAGGGAAGAAAATGACGGTACCTGACTAAGAAGCCCCGGCTAACTACGTGCCAGCAGCCGCGGTAATACGTAGGGGGCAAGCGTTATCCGGATTTACTGGGTGTAAAGGGAGCGTAGACGGCGTGCCAAGTCTGATGTGAAAGGCCGGGGCTCAACCCCGGGACTGCATTGGAAACTGGCATGCTGGAGTGCCGGAGGGGCAAGCGGAATTCCTAGTGTAGCGGTGAAATGCGTAGATATTAGGAGGAACACCGGTGGCGAAGGCGGCTTGCTGGACGGTAACTGACGTTGAGGCTCGAAAGCGTGGGGAGCAAACAGG

>OTU498GTGAGGAATATTGGTCAATGGCCGGGAGGCTGAACCAGCCAAGTCGCGTGAGGGAAGAATGTCCTATGGATTGTAAACCTCTTTTGTCGGGGAGCAAAACGGCACTGCGGTGCCCGATTGAGAGTACCCGAAGAAAAAGCATCGGCTAACTCCGTGCCAGCAGCCGCGGTAATACGGAGGATGCGAGCGTTATCCGGATTTATTGGGCGTAAAGCGAGCGCAGGCGGAAAAATAAGTCTAATGTGAAAGCCCTCGGCTTAACCGAGGAATTGCATCGGAAACTGTTTTTCTTGAGTGCAGAAGAGGAGAGTGGAACTCCATGTGTAGCGGTGGAATGCGTAGATATATGGAAGAACACCAGTGGCGAAGGCGGCTCTCTGGTCTGCAACTGACGCTGAGGCTCGAAAGCATGGGTAGCGAACAGG

>OTU499GTGGGGAATATTGGGCAATGGGCGCAAGCCTGACCCAGCAACGCCGCGTGAAGGAAGAAGGCTTTCGGGTTGTAAACTTCTTTTGACAGGGAAGAGTAGAAGACGGTACCTGTCGAATAAGCCACGGCTAACTACGTGCCAGCAGCCGCGGTAATACGTAGGTGGCAAGCGTTGTCCGGATTTACTGGGTGTAAAGGGCGTGTAGCCGGGTTGACAAGTCAGATGTGAAATCCTGCGGCTTAACCGCAGAACTGCATTTGAAACTGTTGATCTTGAGTACTGGAGAGGCAGACGGAATTCCTAGTGTAGCGGTGAAATGCGTAGATATTAGGAGGAACACCAGTGGCGAAGGCGGTCTGCTGGACAGCAACTGACGGTGAGGCGCGAAAGCGTGGGGAGCAAACAGG

>OTU500GTAGGGAATTTTCGGCAATGGGGGAAACCCTGACCGAGCAACGCCGCGTGAAGGAAGAAGGAATTCGTTTCGTAAACTTCTGTTATAAAGGAAGAAAGATGATATCAGGAAATGGATGTCAAGTGACGGTACTTTATGAGAAAGCCACGGCTAACTACGTGCCAGCAGCCGCGGTAATACGTAGGTGGCAAGCGTTATCCGGAATTATTGGGCGTAAAGAGGGAGCAGGCGGTAACAAAGGTCTGTGGTGAAAGCCTGAAGCTTAACTTCAGTAAGCCATAGAAACCAGGTAACTAGAGTGCAAGAGAGGATCGTGGAATTCCATGTGTAGCGGTGAAATGCGTAGATATATGGAGGAACACCAGTGGCGAAGGCGACGATCTGGCTTGTAACTGACGCTGAGTCCCGAAAGCGTGGGGAGCAAATAGG

>OTU501GTGGGGAATATTGGACAATGGGCGCAAGCCTGATCCAGCCATGCCGCGTGGATGATGAAGGCCCTAGGGTTGTAAAGTCCTTTTAACGGGGAAGATAATGACGGTACCCGTAGAATAAGCCCCGGCTAACTTCGTGCCAGCAGCCGCGGTAATACGAAGGGGGCTAGCGTTGCTCGGAATTACTGGGCGTAAAGCGCACGTAGGCGGTCTTTTAAGTCGGGGGTGAAATCCTGGAGCTCAACTCCAGAACTGCCTTCGATACTGGAAGACTTGAGTCCGGGAGAGGTGAGTGGAACTGCGAGTGTAGAGGTGAAATTCGTAGATATTCGCAAGAACACCAGTGGCGAAGGCGGCTCACTGGCCCGGTACTGACGCTGAGGTGCGAAAGCGTGGGGAGCAAACAGG

>OTU502GTGGGGAATATTGGACAATGGGGGCAACCCTGATCCAGCAATTCTGTGTGCACGAAGAAGGTTTTCGGATTGTAAAGTGCTTTCAGCAGGGAAGAAGAAAGTGACGGTACCTGCAGAAGAAGCGACGGCTAAATACGTGCCAGCAGCCGCGGTAATACGTATGTCGCAAGCGTTATCCGGAATTATTGGGCATAAAGGGCATCTAGGCGGCCAGACAAGTCTGGGGTGAAAACTTGCGGCTCAACCGCAAGCCTGCCCTGGAAACTGTTTGGCTAGAGTGCTGGAGAGGTGGACGGAACTGCACGAGTAGAGGTGAAATTCGTAGATATGTGCAGGAATGCCGATGATGAAGATAGTTCACTGGACGGTAACTGACGCTGAAGTGCGAAAGCTGGGGGAGCGAACAGG

>OTU503GTCGGGAATTTTGCGCAATGGGCGAAAGCCTGACGCAGCAACGCCGCGTGAGGGATGAAGGCCTTCGGGTTGTAAACCTCTTTTCTCAGGGACGATGATGACGGTACCTGAGGAATAAGCCACGGCTAACTACGTGCCAGCAGCCGCGGTAATACGTAGGTGGCAAGCGTTGTCCGGATTTACTGGGCGTAAAGAGCGCGCAGGCGGTCGTGCAAGTCGCGTGTGAAAGCCCCCGGCTCAACTGGGGAGGGTCACGCGATACTGTTCGACTCGAAGGCAGGAGAGGGAAGCGGAATTCCCGGTGTAGTGGTGAAATGCGTAGATATCGGGAGGAACACCAGTGGCGAAGGCGGCTTCCTGGCCTGTTCTTGACGCTGAGGCGCGAAAGCTAGGGGAGCAAACGGG

>OTU504GTGAGGGATCTTCGGCAATGGGGGCAACCCTGACCGAGCAACGCCGCGTGCGGGATGACGCCTTTCGGGGTGTAAACCGCTTTTCTGGAGGACGAGGAAGGACGGTACTCCAGGAAGAAGGCTCGGCTAACTACGTGCCAGCAGCCGCGGTAAAACGTAGGAGCCAAGCGTTATCCGAATTCACTGGGCGTAAAGCGCGAGCAGGCGGCTCGCTAAGTTGGATGTGAAATCTCCTGGCTCAACTGGGAGAGGACGTACAAGACTGGCGGGCTAGAGGACGGTAGAGGGAGGTGGAATTCCCGGTGTAGTGGTGAAATGCGTAGATATCGGGAGGAACACCAGTGGCGAAAGCGGCCTCCTGGGCCGATCCTGACGCTGAGATGCGAAAGCTAGGGGAGCAAACGGG

>OTU505GTCGGGAATTTTGGGCAATGGGCGAAAGCCTGACCCAGCAACGCCGCGTGAAGGATGAAATCCCTCGGGATGTAAACTTCGCAAGAACGGGAAGAATAAGTTTGTGCTAATACCACAAGCGATGACGGTACTGTTTGTAAGCTCCGGCTAACTCCGTGCCAGCAGCCGCGGTAATACGGGGGGAGCAAGCGTTGTTCGGATTTACTGGGCGTAAAGGGCGCGTAGGCGGCCTCCGCAAGTCAGTTGTGAAATCTCCGAGCTTAACTCGGAAAGGTCAACTGATACTGCGGGGCTAGAGTGCGGAAGGGGCAACTGGAATTCTTGGTGTAGCGGTGAAATGCGTAGATATCAGGTGGAACACCGATGGCGAAGGCAGTGATCTGGGCCAGTACTGACGCTGAGGAGCGAAAGCGTGGGGAGCAAACAGG

>OTU506GTAGGGAATCTTGCGCAATGGGCGAAAGCCTGACGCAGCGATGCCGCGTGAGGGATGAAGCCGTTCGCGGTGTAAACCTCTTTCAGTAGGGAAGAAGCCACGGGTTACCGTGGTGACGGTACCTGCAGAAGAAGCCCCGGCTAACTACGTGCCAGCAGCCGCGGTAAAACGTAGGGGGCAAGCGTTGCCCGGATTCACTGGGCGTAAAGCGGGTGTAGGCGGTCCGTTAAGTCGGATGTGAAATCCCGAGGCTCAACCTCGGAATCGCATCCGATACTGGCGGACTTGAGGACGGTAGAGGTCGGTGGAATACCAGGTGTACCGGTGAAATGGGCAGATATCTGGTGGAACACCAGTGGCGAAGGCGGCCGACTGGGCCGATCCTGACGCTGAGACCCGAAAGCCAGGGGAGCGAACCGG

>OTU507GTAGGGAATATTGGGCAATGGGCGAGAGCCTGACCCAGCCATGCCGCGTGCAGGATGAAGGCTTTCTGAGTCGTAAACTGCTTTTGCCAGGGAAGAAAAAAGGGGATGCGTCCTCTACTGACGGTACCTGGTGAATAAGCACCGGCTAACTCCGTGCCAGCAGCCGCGGTAATACGGAGGGTGCAAGCGTTGTCCGGATTTATTGGGTTTAAAGGGTGCGTAGGCGGCCGATTAAGTCTGGGGTGAAAGCCCGTTGCTCAACAACGGAACTGCCCTGGAAACTGGTCGGCTTGAGTACAGACGAGGGTGGCGGAATGGATGGTGTAGCGGTGAAATGCATAGATACCATCCAGAACCCCGATTGCGAAGGCAGCTGCCTAGACTGTAACTGACGCTGAGGCACGAAAGCGTGGGGAGCGAACAGG

>OTU508GTGGGGAATATTGCGCAATAGAGGAAACTCTGACGCAGCGATGCCGCGTGAGTGAGGAAGGGATTCGTCCCGTAAAGCTCAGTCGCCGGTTAGGAGAGAAGACCAGCCGGTTTTGAGCCCCGGCTAACTACGTGCCAGCAGCCGCGGCAATACGTAGGGGGCGAGCGTTGTCCGGAATTATTGGGCGTAAAGGGCGCGCAGGCGGCAAGAGAGGCTTTTTGTAAAAGCCCCGGGCCCACCCCGGGGAAGCAGAAAGAACCAGCACAGCTAGAGTACTCTAGAGGCAGGCAGAATTCCTGGTGTAGTGGTGAAATACGCAGAGATCAGGAGGAATACCGGAGGCGAAGGCGGCCTGCTGGAGAGAGACTGACGCTCAGGCGCGAGAGCGTGGGGAGCAAACAGG

>OTU509GTGAGGAATATTGGTCAATGGGCGCGAGCCTGAACCAGCCAAGTCGCGTGAGGGAGGACGGCCCTACGGGTTGTAAACCTCTTTTGCCGGGGAGCAACGGGCGTCACGTGTGGCGCCACTGAGAGTACCCGGAGAAAAAGCATCGGCTAACTCCGTGCCAGCAGCCGCGGTAATACGGAGGATGCGAGCGTTATCCGGATTTATTGGGTTTAAAGGGTGCGTAGGCGGATTTTTAAGTCAGCGGTAAAATGTCCGGGCTCAACCCGGGCCGGCCGTTGAAACTGGGGATCTTGAGTGGGCGAGAAGTATGCGGAATGCGTGGTGTAGCGGTGAAATGCATAGATATCACGCAGAACCCCGATTGCGAAGGCAGCCTTCCGGCGCCCTACTGACGCTGAGGCACGAAAGTGCGGGGATCGAACAGG

>OTU510GTGGGGAATTTTGGACAATGGGGGCAACCCTGATCCAGCAATGCCGCGTGAGTGAAGAAGGCCTTCGGGTTGTAAAGCTCTTTTGTCAGGGAAGAAACAGCAGCTCTAACACAGTTTGCGAATGACGGTACCTGAAGAATAAGCACCGGCTAACTACGTGCCAGCAGCCGCGGTAATACGTAGGGTGCAAGCGTTAATCGGAATTACTGGGCGTAAAGCGTGCGCAGGCGGTTATGTAAGACAGGCGTGAAATCCCCGGGCTTAACCTGGGAATTGCGCTTGTGACTGCATAGCTAGAGTATGTCAGAGGGGGGTAGAATTCCACGTGTAGCAGTGAAATGCGTAGAGATGTGGAGGAATACCAATGGCGAAGGCAGCCCCCTGGGATAATACTGACGCTCATGCACGAAAGCGTGGGGAGCAAACAGG

>OTU511GTGGGGAATTTTGGACAATGGGGGCAACCCTGATCCAGCCATGCCGCGTGTGTGAAGAAGGCCTTCGGGTTGTAAAGCACTTTCGGCCGGAACGAAATCGCGCGATCTAATAAGTCGCGTGGATGACGGTACCGGAAGAAGAAGCACCGGCTAACTACGTGCCAGCAGCCGCGGTAATACGTAGGGTGCGAGCGTTAATCGGAATTACTGGGCGTAAAGTGTGCGCAGGTGGCCGCGCAAGTCGAGTGTGAAATCCCCGGGCTTAACTTGGGAATTGCGCTCGAAACTACGTGGCTCGAGTGTGGCAGAGGAAGGTGGAATTCCACGTGTAGCGGTGAAATGCGTAGAGATGTGGAGGAACACCGATGGCGAAGGCAGCCTTCTGGGCCAACACTGACGCTCATGCACGAAAGCGTGGGGAGCAAACAGG

>OTU512GTGGGGAATATTGGGCAATGGGGGAAACCCTGACCCAGCGACGCCGCGTGGGTGAAGAAATCCTTCGGGATGTAAAGCCCTGTTGTATGGGAAGAGCGGAAGACGGTACCATACGAGGAAGCCCCGGCAAACTACGTGCCAGCAGCCGCGGTAATACGTAGGGGGCAAGCGTTGTCCGGAATTACTGGGCGTAAAGCGCACGCAGGCTGAATTATAAGTCGGTAGTCAAAGGCGGAGGCTCAACCTCTGTATATCTATCGATACTGTAAATCTGGAGTATGTGAGAGGGAAGCGGAATTCCCGGTGTAGCGGTGAAATGCGTAGATATCGGGAGGAACACCAGTGGCGAAGGCGGCTTCCTGGCACAATACTGACGCTCATGTGCGAAAGCTAGGGCAGCGAACGGG

>OTU513GTGGGGAATCTTGGACAATGGGGGAAACCCTGATCCAGCGACGCCGCGTGGGTGAAGAAGGCCTTCGGGTCGTAAAGCCCTTTTGCCAGGGAAGAGGCAGGACGGTACCTGGCGAATAAGCCCCGGCTAACTACGTGCCAGCAGCCGCGGTAATACGTAGGGGGCGAGCGTTGTCCGGAATTACTGGGCGTAAAGAGCGCGTAGGCGGTGCGTTAAGTCGGCTGTGAAATCTCCCGGCTCAACTGGGAGGGGACGGCCGAGACTGGCGGGCTTGAGGGTGGCAGAGGGAGGCGGAATTCCCGGAGTAGTGGTGAAATGCGTAGATACCGGGAGGAACACCGGTGGCGAAGGCGGCCTCCTGGGCTTCTCCTGACGCTGAGGCGCGAAAGCTAGGGGAGCAAACGGG

>OTU514GTGGGGAATTTTGCGCAATGCTCGAGAGAGTGACGCAGCGACGCCGCGTGAATGACGAAGGCCTTCGGGTCGTAAAGTTCTTTCGACAGGGAAGAATGTGTATAGTAGTAACTGACTATACAGTGACGGTACCTGTATAAGCAGCCCCGGCTAACTCCGTGCCAGCAGCCGCGGTAATACGGAGGGGGCGAGCGTTGTTCGGAGTGACTGGGCGTAAAGAGCACGTAGGCGGTGTTGTAAGTCATTAGTCAAAGGCTAGAGCTCAACTTTAGTAAGGCTAGTGATACTATAATACTAGAGTATCAGAGAGGATTGCAGAATTCCTGGTGTAGCGGTGAAATGCGTAGATATCAGGAGGAATACCGGTAGCGAAGGCGGCAATCTGGCTGGAAACTGACGCTGAGGTGCGAAAGCGTGGGTAGCAAACAGG

>OTU515GTAGGGAATTTTCGGCAATGGGGGCAACCCTGACCGAGCAACGCCGCGTGAAGGAAGAAGGTCTTCGGATTGTAAACTTCTGTTATAGGGAAAGAACGATAGGTAGAGGAAATGCTACCTGTGTGACGGTACCCTATGAGAAAGCCACGGCTAACTACGTGCCAGCAGCCGCGGTAATACGTAGGTGGCAAGCGTTATCCGGAATTATTGGGCGTAAAGAGTGAGTAGGCGGTTTCTTAAGTTTGGAGTGAAAAGCAGTTGCTTAACGACTGTTTGCTTCAAAAACTGGGAAGCTAGAGTGCAGGAGAGGTTAACGGAATTCCATGTGTAGCGGTGAAATGCGTAGATATATGGAGGAACACCAGTGGCGAAGGCGGTTAACTGGCCTGTGACTGACGCTCAGTCACGAAAGCGTGGGGAGCAAATAGG

>OTU516GTGAGGAATATTGGTCAATGGAGGCAACTCTGAACCAGCCAAGTCGCGTGAAGGATGACTGCCCTACGGGTTGTAAACTTCTTTTTTAAGGGAATAAAGTGAGCCACGTGTGGCTTTTTGCATGTACCTTACGAATAAGGATCGGCTAACTCCGTGCCAGCAGCCGCGGTAATACGGAGGATCCGAGCGTTATCCGGATTTATTGGGTTTAAAGGGTGCGCAGGCGGAATATTAAGTCGGCGGTGAAAGTTTGCAGCTTAACTGTAAAATTGCCGTTGAAACTGGTTTTCTTGAGTGTATATGAAGTAGGCGGAATTTGTAGTGTAGCGGTGAAATGCATAGATATTACAAGGAACTCCGATTGCGAAGGCAGCTTACTAAAATACAACTGACGCTCAGGCACGAAGGCGTGGGTATCAAACAGG

>OTU517GTGAGGAATATTGGTCAATGGACGCAAGTCTGAACCAGCCATGCCGCGTGCAGGAAGACGGCTCTATGAGTTGTAAACTGCTTTTGTACGAGGGTAAATCCCGATACGTGTATCGGGTTGAAAGTATCGTACGAATAAGGATCGGCTAACTCCGTGCCAACAGCCGCGGTAATACGGAGGATTCAAGCGTTATCCGGATTTATTGGGTTTAAAGGGTGCGTAGGCGGTTCGATAAGTTAGAGGTGAAATCCCGGGGCTCAACTCCGGCACTGCCTCTGATACTGTCGGGCTAGAGTTTAGTTGCGGTAGGCGGAATGTATGGTGTAGCGGTGAAATGCATAGAGATCATACAGAACACCGATTGCGAAGGCAGCTTACCAAACTACGACTGACGTTGAGGCACGAAAGCGTGGGGAGCAAACAGG

>OTU518GTCGAGAATCTTCCGCAATGGGCGCAAGCCTGACGGAGCGACGCCGCGTGAAGGATGACGGCCTTCGGGTTGTAAACTTCTGTCACCCGAGAAGAACGTTCGAGGTAACCCCTGGGACTGACGTTATCGGGAGAGGAAGCCACGGCTAACTTCGTGCCAGCAGCCGCGGTAATACGAAGGTGGCAACCGTTGCTCGGATTTACTGGGCATAAAGCGCGCGTAGGCGGTTCAGCAAGTCAACGGTGAAATCCCCCGGCCCAACCGGGGAATAGCCGCTGAAACTGCTGAGCTTGAGTACGGGAGGGGAGAGTGGAACTCTTGGTGTAGCGGTGAAATGCGTAGATATCAAGAGGAACGCCGGTGGCGAAAGCGACTCTCTGGACCGTAACTGACGCTGAGGCGCGAAAGCCAGGGGAGCAAACAGG

>OTU519GTGGGGAATTTTCCGCAATGGGCGAAAGCCTGACGGAGCAATCTCGCGTGAGGGATGACGGCCTATGGGTTGTAAACCTCTTTTTTCAGGGAGGAATAAAATGACGTGTACCTGAAGAATAAGCATCGGCTAACTCCGTGCCAGCAGCCGCGGTAAGACGGAGGATGCAAGTGTTATCCGGAATCACTGGGCGTAAAGCGTCTGTAGGTGGTTTAATAAGTCAACTGTTAAATCTTGAGGCTCAACCTCAAAATCGCAGTCGAAACTATTAGGCTAGAGTATAGTAGGGGTAAAGGGAATTTCCAGTGGAGCGGTGAAATGCGTAGAGATTGGAAAGAACACCGATGGCGAAGGCACTTTACTGGGCTATTACTAACACTCAGAGACGAAAGCTAGGGTAGCAAACAGG

>OTU520GTGAGGAATATTGGTCAATGGTCGCAAGACTGAACCAGCCATGCCGCGTGCAGGAAGACGGTCCTATGGATTGTAAACTGCTTTTATACAGGAAGAAACCCTCCCACGTGTGGGAGCTTGACGGTACTGTAGGAATAAGGATCGGCTAACTCCGTGCCAGCAGCCGCGGTAATACGGAGGATCCAAGCGTTATCCGGAATCATTGGGTTTAAAGGGTCCGTAGGCGGTCTTATAAGTCAGTGGTGAAAGCCCATCGCTCAACGATGGAACTGCCATTGATACTGTAAGACTTGAATGCTTAGGAAGTAACTAGAATATGTAGTGTAGCGGTGAAATGCTTAGATATTACATGGAATACCAATTGCGAAGGCAGGTTACTACTAAGTGATTGACGCTGATGGACGAAAGCGTGGGGAGCGAACAGG

>OTU521GTGGGGAATCTTGCGCAATGGGGGCAACCCTGACGCAGCGACGCCGCGTGCGGGATGAAGGTCTTCGGATCGTAAACCGCTTTCAGCAGGGACGAGGGCGAAAGCTGACGGTACCTGCAGAAGAAGCTCCGGCTAACTACGTGCCAGCAGCCGCGGTAACACGTAGGGGGCGAGCGTTATCCGGATTCATTGGGCGTAAAGCGCGCGTAGGCGGCCTGTTAGGTCGAGGGTCTAATCTCGGGGCTCAACCTCGAGCCGCTCTCGATACCGGCAGGCTTGAGTCTGGTAGGGGAAGGCGGAACTCCCGGTGTAGCGGTGGAATGCGCAGATATCGGGAGGAACACCGGTGGCGAAGGCGGCCTTCTGGGCCATGACTGACGCTCAGGCGCGAAAGCTAGGGGAGCAAACAGG

>OTU522GTAAGGGATATTGCGCAATGGGCGAAAGCCTGACGCAGCAACGCCGCGTGGAGGATGACGGCCTTCGGGTTGTAAACTCCTTTTGAGGCTGACGAGGAAGGACGGTAAGCCTTGAAGAAGTCACGGCTAACTACGTGCCAGCAGCCGCGGTAAAACGTAGGTGGCGAGCGTTATCCGGATTTACTGGGCGTAAAGCGTGTGCAGGCGGGGTATCAAGTGGTGCATGAAATCGCTCGGCTTAACCGGGCGCGGTTGTGCCAGACTGATATGCTAGAGTGCGGGAGAGGGGCGTGGAATTCCGGGTGTAGTGGTGAAATGCGTAGAGATCCGGAGGAACCCCAGAGGCGAAGGCGGCGCCCTGGCCCGACACTGACGCTCAGACACGACAGCATGGGGAGCGAACGGG

>OTU523GTGGGGAATATTGGACAATGGGGGAAACCCTGATCCAGCGACGCCGCGTGGGTGAAGAAGGCCTGCGGGTTGTAAAGCCCTTTCGGTAGGGACGAAAGCTCTTGCCTTAATACGGCAGGAGGTTGACTTAACCTACATAAGAAGCACCGGCTAACTCTGTGCCAGCAGCCGCGGTAATACAGAGGGTGCGAGCGTTAATCGGAATTACTGGGCGTAAAGCGCACGTAGTCGGCTTTGCAAGTCGGGTGTGAAATCCCCGGGCTCAACCTGGGAACTGCATCCGAGACTGCATTGCTAGAGTATGGGAGAGGGAAGCGGAATTTCTGGTGTAGCAGTGAAATGCGTAGATATCAGAAGGAACATCAGTGGCGAAGGCGGCTTCCTGGACCAATACTGACGATCAGGTGCGAAAGCGTGGGGAGCAAACAGG

>OTU524GTGAGGAATATTGGTCAATGGGCGAGAGCCTGAACCAGCCAAGCCGCGTGAGGGATGAAGGCCCTACGGGTCGTAAACCTCTTTTGCCGGGGAACAAAAGCGGGTATGTATACCCGTATGAGTGTACCCGGAGAAAAAGCATCGGCTAACTCCGTGCCAGCAGCCGCGGTAATACGGAGGATGCGAGCGTTATCCGGATTTATTGGGTTTAAAGGGTGCGTAGGCGGCATTTCAAGTCAGCGGTAAAATTGCGGGGCTCAACCCCGTACAGCCGTTGAGACTGAGGAGCTAGAGACGGCGAGAGGTACGCGGAATGCGCAGTGTAGCGGTGAAATGCATAGATATTGCGCAGAACTCCGATTGCGAAGGCAGCGTACTGGCGCCGGACTGACGCTGAGGCACGAAAGCGTGGGGATCGAACAGG

>OTU525GTGGGGAATATTGCACAATGGGGGAAACCCTGATGCAGCGACGCCGCGTGAACGAAGAAGGTATTCGTATCGTAAAGTTCTGTCCTATGGGAAGATAATGACAGTACCATAGAAGAAAGCTCCGGCTAAATACGTGCCAGCAGCCGCGGTAATACGTATGGAGCGAGCGTTGTCCGGAATTATTGGGCGTAAAGGGTACGCAGGCGGTTTAATAAGTCGAATGTTAAAGATCGGGGCTCAACCCCGTAAAGCATTGGAAACTGATAAACTTGAGTAGTGGAGAGGAAAGTGGAATTCCTAGTGTAGTGGTGAAATACGTAGATATTAGGAGGAATACCAGTAGCGAAGGCGACTTTCTGGACACAAACTGACGCTGAGGTACGAAAGCGTGGGGAGCAAACAGG

>OTU526GTGGGGAATCTTGCGCAATGGGCGAAAGCCTGACGCAGCCATGCCGCGTGTATGATGAAGGTCTTAGGATTGTAAAATACTTTCACCGGTGAAGATAATGACTGTAGCCGGAGAAGAAGCCCCGGCTAACTTCGTGCCAGCAGCCGCGGTAATACGAAGGGGGCTAGCGTTGCTCGGAATTACTGGGCGTAAAGGGAGCGTAGGCGGACATTTAAGTCAGAGGTGAAATCCCGGAGCTTAACTTCGGAACTGCCTTTGATACTGGGTGTCTTGAGTGTGAGAGAGGTATGTGGAACTCCGAGTGTAGAGGTGAAATTCGTAGATATTCGGAAGAACACCAGTGGCGAAGGCGACATACTGGCTCATTACTGACGCTGAGGCTCGAAAGCGTGGGGAGCAAACAGG

>OTU527GTGGGGAATTGTTCGCAATGGGCGCAAGCCTGACGACGCAACGCCGCGTGGAGGATGAAGGTCTTCGGATTGTAAACTCCTGTTGATCGGGAAGAATGGACTCCGGGTGAATACTCCGGGGTATTGACGGTACCGATTGAGGAAGCCACGGCTAACTCTGTGCCAGCAGCCGCGGTAATACAGAGGTGGCAAGCGTTGTTCGGAATTACTGGGCGTAAAGGGCGCGTAGGCGGTCGCCTAAGTCGGACGTGAAATCCCCAAGCTTAACTTGGGAACTGCGTCCGATACTGGGTGACTTGAGTTCGGGAGAGGAATGTGGAATTCCAGGTGTAGCGGTGAAATGCGTAGATATCTGGAGGAACACCGGTGGCGAAGGCGGCATTCTGGACCGAAACTGACGCTGAGGAGCGAAAGCGTGGGGAGCGAACAGG

>OTU528GTGGGGAATATTGCACAATGGACGAAAGTCTGATGCAGCAACGCCGCGTGAGGGACGAAGGCCTTCGGGTTGTAAACCTCTTTTAGCAGGGAAGAAGCGAAAGTGACGGTACCTGCAGAAAAAGCACCGGCTAACTACGTGCCAGCAGCCGCGGTAATACGTAGGGTGCAAGCGTTGTCCGGAATTATTGGGCGTAAAGAGCTCGTAGGCGGTTTGTCGCGTCTGCTGTGAAATTTCGAGGCTCAACCTCGAACTTGCAGTGGGTACGGGCAGGCTAGAGTGCAGTAGGGGAGATGGGAATTCCTGGTGTAGCGGTGGAATGCGCAGATATCAGGAGGAACACCAATGGCGAAGGCACATCTCTGGGCTGTAACTGACGCTGAGGAGCGAAAGCGTGGGGAGCGAACAGG

>OTU529GTGGGGAATCTTGGACAATGGGGGCAACCCTGATCCAGCCATGCCGCGTGAGCGATGAAGGCCTTAGGGTTGTAAAGCTCTTTCAGCGGGGAAGATAATGACGGTACCCGCAGAAGAAGCCCCGGCTAACTCCGTGCCAGCAGCCGCGGTAATACGGAGGGGGCTAGCGTTGTTCGGAATTACTGGGCGTAAAGCGCACGTAGGCGGATCAGTTAGTCGGGTGTGAAATCCCGGGGCTCAACCCCGGAACTGCCTTCGATACTGCTGGTCTGGAGGTCGAGAGAGGTGAGTGGAATTCCGAGTGTAGAGGTGAAATTCGTAGATATTCGGAGGAACACCAGTGGCGAAGGCGGCTCACTGGCTCGATACTGACGCTGAGGTGCGAAAGCGTGGGGAGCAAACAGG

>OTU530GTCGAGAATTTTTCTCAATGGGGGAAACCCTGAAGGAGCGACGCCGCGTGGAGGATGAAGGCTTTCGGGTTGTAAACTCCTGTCATTTGGGAACAAGGTGCGAGTATTAACTGTACTCGCATTGATAGTACCAGAAGAGGAAGGGACGGCTAACTCTGTGCCAGCAGCCGCGGTAATACAGAGGTCCCAAGCGTTGTTCGGATTCATTGGGCGTAAAGGGTGTGTAGGTGGCGCCGTAAGTCGGGTGTGAAATCTCGGGGCTTAACTCCGAAACTGCATTCGATACTGCGGTGCTTGAGGACTGGAGAGGAGACTGGAATTCATGGTGTAGCAGTGAAATGCGTAGAGATCATGAGGAAGACCAGTGGCGAAGGCGGGTCTCTGGACAGTTCCTGACACTGAGACACGAAGGCCAGGGGAGCAAACGGG

>OTU531GCCGAGAATCTTCCGCAATGGGCGAAAGCCTGACGGAGCGACACCGCGTGTAGGATGAAGGCCTTCGGGTTGTAAACTACTGTCAAGTGGGAAAAGATTCACTATGTCTAATAAGCATGGTGATTGATTGTACCGCTAGAGGAAGCCACGGCTAACTCTGTGCCAGCAGCCGCGGTAATACAGAGGTGGCGAGCGTTGTTCGGATTGATTGGGTGTAAAGGGTGCGTAGGCGGTTTAGAAAGTCGGGTGTGAAATCCTATGGCTTAACCATAGAATTGCATTCGAAACTTCTAGACTAGAGTGCGAGAGAGGTAAAGGGAATTCCCGGTGTAAGGGTGAAATCTGTAGAGATCGGGAGGAACACCAGTAGCGAAGGCGCTTTACTGGCTCGTAACTGACGCTGAGTGCACGAAAGCATGGGGAGCAAACGGG

>OTU532GTGAGGAATATTGGTCAATGGAAGAGATTCTGAACCAGCCAAGCCGCGTGAGGGAAGACGGCGCTACGCGTTGTAAACCTCTTTTGTCGGAGAACAAAAAACGGGATGTATCCCGTGTTGAGTGTATCCGAAGAAAAAGCATCGGCTAACTCCGTGCCAGCAGCCGCGGTAATACGGAGGATGCGAGCGTTATCCGGATTTATTGGGTTTAAAGGGTGCGTAGGCGGGGACTTAAGTCAGCGGTAAAATTGTGAGGCTCAACCTCATCGAGCCGTTGAAACTGAGTTTCTTGAGTGGGCGAGAAGTATGCGGAATGCGTGGTGTAGCGGTGAAATGCATAGATATCACGCAGAACTCCGATTGCGAAGGCAGCATACCGGCGCCCAACTGACGCTGAAGCACGAAAGCGTGGGTATCGAACAGG

>OTU533GTGGGGAATCTTGCACAATGGGGGAAACCCTGATGCAGCGACGCCGCGTGAGCGATGAAGCCCTTCGGGGTGTAAAGCTCTTTCGTCAGGGAAGATTATGACGGTACCTGGAGAAGCAGCTGCGGCTAACTACGTGCCAGCAGCCGCGGTAATACGTAGGCAGCGAGCGTTGTTCGGAGTTACTGGGCGTAAAGGGTGTGTAGGCGGTTCTTTAAGTTTGGTGTGAAATCTCCCGGCTCAACCGGGAGGGTGCGCCGAATACTGAGGGACTTGGAGTGCGGGAGAGGAAAGTGGAATTCCTGGTGTAGCGGTGAAATGCGTAGATATCAGGAGGAACACCGGTGGTGTAGACGGCTTTCTGGACCGTAACTGACGCTGAGACACGAAAGCGTGGGTAGCAAACAGG

>OTU534GTAGGGAATTTTCGGCAATGGGGGAAACCCTGACCGAGCAACGCCGCGTGAAGGAAGAAGGTTTTCGGATTGTAAACTTCTGTTATAAAGGAAGAACGGCGGCTACAGGAAATGGTAGCCGAGTGACGGTACTTTATTAGAAAGCCACGGCTAACTACGTGCCAGCAGCCGCGGTAATACGTAGGTGGCAAGCGTTATCCGGAATTATTGGGCGTAAAGAGGGAGCAGGCGGCAGCAAGGGTCTGTGGTGAAAGCCTGAAGCTTAACTTCAGTAAGCCATAGAAACCAGGCAGCTAGAGTGCAGGAGAGGATCGTGGAATTCCATGTGTAGCGGTGAAATGCGTAGATATATGGAGGAACACCAGTGGCGAAGGCGACGATCTGGCCTGCAACTGACGCTCAGTCCCGAAAGCGTGGGGAGCAAATAGG

>OTU535GTTAAGAATCTTGGACAATGGACGCAAGTCTGATCCAGCGACGCCGCGTGAACGATGAAGGTCTTCGGATTGTAAAGTTCGGTAAGATGGGACGAAGAAGCGGAGGGTAATATACTCCGTGGTGACGGTACCATCCTAAAGCCTCGGCTAACTACGTGCCAGCAGCCGCGGTAATACGTAGGGGGCGAGCGTTGTCCGAATTTATTGGGCGTAAAGCGCGTGTAGGCGGCTCTTTAAGTCAGATGTGAAATCCTGCGGCTCAACCGCAGAACTGCATTTGATACTGGAGGGCTTGAGTGTGGGAGAGGAAAGTGGAATTCCAGGTGTAGCGGTGAAATGCGTAGATATCTGGAGGAACACCCGTGGCGAAGGCGGCTTTCTGGCCCACCACTGACGCTGAGACGCGAAGGCTAGGGGAGCGAACGGG

>OTU536GTGGGGAATATTGGACAATGGGCGCAAGCCTGATCCAGCCATGCCGCGTGAGTGAAGAAGGCCTTCGGGTTGTAAAGCTCTTTTGTCCGGAAAGAAAAGCTTTCGGTTAATACCCGGAAGTCCTGACGGTACCGGAAGAATAAGCACCGGCTAACTTCGTGCCAGCAGCCGCGGTAATACGAAGGGTGCAAGCGTTACTCGGAATTACTGGGCGTAAAGCGTGCGTAGGTGGTTCGTTAAGTCTGATGTGAAAGCCCTGGGCTCAACCTGGGAATTGCATTGGATACTGGCGGGCTAGAGTGCGGTAGAGGATGGCGGAATTCCCGGTGTAGCAGTGAAATGCGTAGAGATCGGGAGGAACATCCGTGGCGAAGGCGGCCATCTGGACCAGCACTGACACTGAGGCACGAAAGCGTGGGGAGCAAACAGG

>OTU537GTGGGGAATCTTCCGCAATGGGCGAAAGCCTGACGGAGCAATGCCGCGTGAGTGAAGAAGGCCTTCGGGTTGTAAAACTCTGTCCTTATCGAAGAGAGGTGGGTATGTGAATAATGTACTCATAGGACGGTAGATAAGGAGGAAGCCCCGGCTAACTACGCGCCAGCAGCCGCGGTAATACGTAGGGTGCAAGCGTTAATCGGAATTACTGGGCGTAAAGCGTGCGCAGGCGGCTTGATAAGACAGGTGTGAAATCCCCGAGCTCAACTTGGGAATAGCACTTGTGACTGTCAGGCTAGAGTATGTCAGAGGGAGGTGGAATTCCAAGTGTAGCAGTGAAATGCGTAGATATTTGGAAGAACACCGATGGCGAAGGCAGCCTCCTGGGATAATACTGACGCTCATGCACGAAAGCGTGGGGAGCAAACAGG

>OTU538GTGGGGAATATTGGACAATGGGGGCAACCCTGATCCAGCCATGCCGCGTGAGTGATGAAGGCCTTCGGGTTGTAAAGCTCTTTTGGCGGGGACGATGATGACGGTACCCGCAGAATAAGCCCCGGCTAACTTCGTGCCAGCAGCCGCGGTAATACGGGGGGGGCAAGCGTTGTTCGGAATTACTGGGCGTAAAGGGCTCGTAGGTGGCCAACTAAGTCAGACGTGAAATCCCTCGGCTTAACCGGGGAACTGCGTCTGATACTGGATGGCTTGAGTTTGGGAGAGGGATGCGGAATTCCAGGTGTAGCGGTGAAATGCGTAGATATCTGGAGGAACACCGGTGGCGAAGGCGGCATCCTGGACCAACACTGACACTGAGGAGCGAAAGCCAGGGGAGCAAACGGG

>OTU539GTGGGGAATATTGGGCAATGGGGGAAACCCTGACCCAGCAACGCCGCGTGAAGGAAGAAGGCTTTCGGGTTGTAAACTTCTTTTACCAGGGACGAAGGACGTGACGGTACCTGGAGAAAAAGCAACGGCTAACTACGTGCCAGCAGCCGCGGTAATACGTAGGTTGCAAGCGTTGTCCGGATTTACTGGGTGTAAAGGGAGCGTAGACGGTAATGCAAGTCTGGAGTGAAAGGCAGGGGCCCAACCCCTGGACTGCTTTGGAAACTGTGTAACTGGAGTGCAGGAGAGGTAAGTGGAATTCCTAGTGTAGCGGTGAAATGCGTAGATATTAGGAGGAACACCAGTGGCGAAGGCGGCTTACTGGACGATTACTGACGCTGAGGCTCGAAAGCGTGGGGAGCAAACAGG

>OTU540GTGAGGAATATTGGTCAATGGCCGGAAGGCTGAACCAGCCATGCCGCGTGAAGGTCAGTGCCCTATGGGCGTTAAACTTCTTTTGTGCGGGAGCAATAAGGTCCACGTGTGGGCCGACGAGAGTACCGTACGAATAAGCATCGGCTAACTCCGTGCCAGCAGCCGCGGTAATACGTAGGGGGCAAGCGTTATCCGGATTTACTGGGTGTAAAGGGAGCGTAGACGGAAGAGCAAGTCTGATGTGAAAGGCTGGGGCTTAACCCCAGGACTGCATTGGAAACTGTGCAGCTTGAGTGCGGGAGAGGTAAGTGGAATTCCTAGTGTAGCGGTGAAATGCGTAGATATTAGGAGGAACACCAGTGGCGAAGGCGGCTTACTGGACTGTAACTGACGTTGAGGCTCGAAAGCGTGGGGAGCAAACAGG

>OTU541GTGGGGAATTTTGGACAATGGGGGCAACCCTGATCCAGCCATGCCGCGTGTGTGAAGAAGGCCTTCGGGTTGTAAAGCACTTTCGGACGGAACGAAATCGCGCGGGTTAATAGTCCGCGTGGATGACGGTACCGTAAGAAGAAGCACCGGCTAACTACGTGCCAGCAGCCGCGGTAATACGTAGGGTGCGAGCGTTAATCGGAATTACTGGGCGTAAAGCGTGCGCAGGCGGTTCGCCAAGTCAGGTGTGAAATCCCCGGGCTTAACCTGGGAATTGCGCTTGAAACTAGCGGGCTTGAGTGTGGCAGAGGGGGGTGGAATTCCACGTGTAGCAGTGAAATGCGTAGAGATGTGGAGGAACACCGATGGCGAAGGCAGCCCCCTGGGTCAACACTGACGCTCATGCACGAAAGCGTGGGGAGCAAACAGG

>OTU542GTGGGGAATATTGCACAATGGAGGAAACTCTGATGCAGCCATGCCGCGTGTGTGAAGAAGGCTTTCGGGTTGTAAAGCACTTTCAGCGAGGAGGAAAGGTTAGTAGTTAATACCTGCTAGCTGTGACGTTACTCGCAGAAGAAGCACCGGCTAACTCCGTGCCAGCAGCCGCGGTAATACGGAGGGTGCAAGCGTTAATCGGAATTACTGGGCGTAAAGCGCGCGTAGGCGGTTTGTTAAGCTAGATGTGAAAGCCCCGGGCTCAACCTGGGAACTGCATTTAGAACTGGCAGGCTAGAATACGGTAGAGGTTAGTGGAATTTCCTGTGTAGCGGTGAAATGCGTAGAGATGGGAAGGAACACCAGTGGCGAAGGCGACTGACTGGACCGATATTGACGCTGAGGTGCGAAAGCGTGGGTAGCAAACAGG

>OTU543GTGGGGAATTTTGCGCAATGGGGGAAACCCTGACGCAGCGATGCCGCGTGGAGGACGAAGGTCTTCGGATTGTAAACTCCTTTTGCGGGGGAAAAATGGCGGCGTGAATAACGCCGTTCGATGGTACTCCGCGAATAAGCCACGGCTAACTTCGTGCCAGCAGCCGCGGTAAGACGAAGGTGGCGAGCGTTACTCGGAATTACTAGGCGTAAAGCGCGAGCAGGCGGTTCGGTAAGTCTATTGTGAAAGCTCCGGGCTCAACCCGGAGAGGCCAATAGATACTGCTGGACTTGAATACGGGAGAGGTCACTGGAATTCCTGGTGTAGCGGTGAAATGCGTAGATATCAGGAGGAACACCAAAGGCGAAGGCAGGTGACTGGACCGTTATTGACGCTGAGTCGCGAAAGCCGGGGGAGCAAACAGG

>OTU544GTAGGGAATCTTGCGCAATGGGCGAAAGCCTGACGCAGCGACGCCGCGTGAGCGATGAAGGCCTTCGGGTTGTAAAGCTCTGTGGGGGGAGAAGAATAAGGGCGAGCTAATACCTCGCTCGATGACGGTATCCCCTTAGCAAGCACCGGCTAACTCTGTGCCAGCAGCCGCGGTAAGACAGAGGGTGCAAACGTTGTTCGGAATTACTGGGCGTAAAGCGCGTGTAGGCGGCTAAGCAAGTCGGGCGTGAAATCCCACGGCTCAACCGTGGAAGTGCACCCGAAACTGCATAGCTAGAGTCCTGGAGAGGAAGGTGGAATGCTTGGTGTAGAGGTGAAATTCGTAGATATCAAGCGGAACACCGGTGGCGAAGGCGGCCTTCTGGACAGTGACTGACGCTGAGACGCGAAAGCGTGGGGAGCAAACAGG

>OTU545GTAGGGAATCTTCCACAATGGACGAAAGTCTGATGGAGCAACGCCGCGTGAGTGAAGAAGGTTTTCGGATCGTAAAGCTCTGTTGTTGGTGAAGAAGGATAGAGGTAGTAACTGGCCTTTATTTGACGGTAATCAACCAGAAAGTCACGGCTAACTACGTGCCAGCAGCCGCGGTAATACGTAGGTGGCAAGCGTTGTCCGGAATTATTGGGCGTAAAGGGAGCGCAGGCGGAAGGACAAGCCGGATTTGAAAGTTCGGGGCTCAGCCCCGTGAGGGATCCGGAACTGTCCTCCTTGAGAGCGGGAGAAGGAAGCGGAATTCCTGGTGTAGCGGTGAAATGCATAGATATCGTCAGGAACACCGATAGCGAAGGCAGCTTACTAGACTGTAACTGACGCTGAGGCACGAAAGTGTGGGGATCAAACAGG

>OTU547GTGGGGAATATTGCGCAATGGAGGGAACTCTGACGCAGCGATGCCGCGTGAGTGAAGAAGGGATTCGTCCCGTAAAGCTCTGTTGCCAGGGATGATCAAGACAGTACCTGGCGAGAAAGCCCCGGCTAACTACGTGCCAGCAGCCGCGGTAACACGTAGGGGGCGAGCGTTGTCCGGAATTACTGGGCGTAAAGGGCGCGTAGGCGGCGGCGAAGGCCTGTTGTAAAATATCCGGGCTCAACCCGGAGAGGCGACAGGAACCGAGTCAGCTGGAGTGCTGGAGAGGCAGGCAGAATTCCTGGTGTAGCGGTGAAATGCGCAGAGATCAGGAGGAATACCGGAGGCGAAGGCGGCCTGCTGGACAGCAACTGACGCTAAGGCGCGAGAGCGTGGGGAGCAAACAGG

>OTU548GTAGGGAATTTTCGTCAATGGGCGCAAGCCTGAACGAGCAATGCCGCGTGAGTGAGGAAGGTCTTCGGATCGTAAAGCTCTGTTGCGGGGGAAAAAGGAAGCAGAAAGGAAATGGTCTGCTTTTGATGGTACCCCGCCAGAAAGTCACGGCTAACTACGTGCCAGCAGCCGCGGTAATACGTAGGTGGCGAGCGTTATCCGGAATGATTGGGCGTAAAGGGTGCGCAGGCGGCGCGTCAAGTCTGAAGTAAAAGGTACAGGCTCAACCTGTGCAGGCTTTGGAAACTGGCGCGCTCGAGGACAGGAGAGGGCGGTGGAACTCCATGTGTAGCGGTAAAATGCGTAGATATATGGAAGAACACCAGTTGCGAAGGCGGCCGCCTGGACTGTTACTGACGCTGAGGCACGAAAGCGTGGGGAGCAAATAGG

>OTU549GTGGGGAATCTTGCACAATGGGCGAAAGCCTGATGCAGCAACGCCGCGTGAGCGAAGAAGGCCTTTGGGTCGTAAAGCTCTGTTCTAGGGGAAGAAAAAAATGACGGTACCCTAGAAGAAAGCCCCGGCTAACTCCGTGCCAGCAGCCGCGGTAATACGGAGGGGGCAAGCGTTATCCGGAATTATTGGGCGTAAAGGGTACGTAGGTGGTTCTTTAAGCGCAGGGTTTAAGGCAATGGCTCAACCATTGTTCGCCCTGCGAACTGGGGAACTTGAGTGCAGGAGAGGAAAGCGGAATTCCTAGTGTAGCGGTGAAATGCGTAGATATTAGGAGGAACACCAGTGGCGAAGGCGGCTTTCTGGACTGTAACTGACACTGAGGTACGAAAGCGTGGGGAGCAAACAGG

>OTU550GTCGAGGATCTTCGGCAATGGGCGCAAGCCTGACCGAGCGACGCCGCGTGTGCGATGAAGGCCTTCGGGTTGTAAAGCACTGTCGAGGGGGAGGAAAGCCGAAAGGTCTGACCTATCCCTGGAGGAAGCACGGGCTAAGTTCGTGCCAGCAGCCGCGGTAAGACGAACCGTGCGAACGTTGTTCGGAATCACTGGGCTTAAAGGGCGCGTAGGCGGGTGTCCAAGTCAGGGGTGAAATCCTCCAGCTTAACTGGAGAACTGCCTGTGATACTGGACGTCTGGAGTGAGGTAGGGGCATGTGGAACTTCCGGTGGAGCGGTGAAATGCGTAGATATCGGAAGGAACACCGGTGGCGAAGGCGGCATGCTGGACCTCAACTGACGCTGAGGCGCGAAAGCCAGGGGAGCAAACGGG

>OTU551GTTAGGAATATTCGTCAATGGGGGAAACCCTGAACGAGCAATGCCGCGTGAGTGATGAAGGTCTTTTTGATCGTAAAACTCTGTTGTAAAGGAAGAAACCTTAGCATAGGTAATGATGCTAAGCTGACAGTACTTTACCAGAAAGCCCCGGCTAACTACGTGCCAGCAGCCGCGGTAATACGTAGGGGGCGAGCGTTATCCGGATTTATTGGGCGTAAAGCGTGTGTAGGCGGTTTATTAAGTATAAGATTAAAGCCCGAGGCTTAACCTCGGTTCGTTTTATAAACTGGTAGACTTGAGTGTGGTAGAGGCAAGTGGAATTTCTAGTGTAGCGGTTAAATGCGTAGATATTAGAAGGAACATCAGTGGCGAAGGCGGCTTGCTGGGCCATTACTGACGCTGAGACACGAAAGCGTGGGGAGCAAATAGG

>OTU552GTGGGGAATATTGCGCAATGGAGGCAACTCTGACGCAGCAATGCCGCGTGAGTGAAGAAGGGATTCGTCCCGCAGAGCTCAGGAGTCAGCAAAGAGAGAGACGGTCGCTGACTTTAAGCCCCGGCTAACTACGTGCCAGCAGCCGCGGCAATACGTAGGGGGCAAGCGTTGTCCGGAATCATTGGGCGTAAAGCGCGCGCAGGCGGCGGGAGAGGCTTTTTGTAAAAGTCCCGGGCTCACCCCGGGGAGGCAGAAAGAACCAATCCGCTAGAGTGCTCTAGAGGCAGGCAGAATTCCTGGTGTAGTGGTGAAATACGCAGAGATCAGGAGGAATACCGGAGGCGAAGGCGGCCTGCTGGAGAGAGACTGACGCTCAGGCGCGAGAGCGTGGGGAGCAAACAGG

>OTU553GTCGAGAATTTTTCACAATGGGGGAAACCCTGATGGAGCGACGCCGCGTGGGGGATGAATGGCTTCGGCCCGTAAACCCCTGTCATTTGCGATCAAAGCGCATTATTTAAAAGATGATGTGTTGATAGTAGCGAAAGAGGAAGGGACGGCTAACTCTGTGCCAGCAGCCGCGGTAATACAGAGGTCCCGAGCGTTGTTCGGATTCACTGGGCGTAAAGGGTGCGTAGGTGGTGAGGTGTGTCGGATGTGAAATCTCGGAGCTCAACTCCGAAACTGCATTGGAAACTACCTTGCTGGAGGGTTGGAGGGGGGACTGGAATACTTGGTGTAGCAGTGAAATGCGTAGATATCAAGTGGAACACCAGTGGCGAAGGCGAGTCCCTGGACAACTCCTGACACTGAGGCACGAAAGCTAGGGGAGCAAACAGG

>OTU554GTGGGGAATATTGCACAATGGGGGAAACCCTGATGCAGCAACGCCGCGTGAGTGAAGAAGTATTTCGGTATGTAAAGCTCTATCAGCAGGGAAGAAAGTGACGGTACCTGACTAAGAAGCCCCGGCTAACTACGTGCCAGCAGCCGCGGTAATACGTAGGGGGCAAGCGTTATCCGGATTTACTGGGTGTAAAGGGAGCGTAGACGGCACAGCAAGTCTGAAGTGAAATCCCCGGGCTCAACCCGGGAACTGCTTTGGAAACTGTTGGGCTGGAGTGTTGGAGAGGCAAGCGGAATTCCTAGTGTAGCGGTGAAATGCGTAGATATTAGGAGGAACACCAGTGGCGAAGGCGGCTTGCTGGACAATCACTGACGTTGAGGCTCGAAAGCGTGGGGAGCAAACAGG

>OTU555GTAGGGAATTTTCGGCAATGGGCGAAAGCCTGACCGAGCAACGCCGCGTGAACGAAGAAGGTCTTCGGATTGTAAAGTTCTGTTATAGAGGAAGAACGGTGTACGTAGGAAATGGCGTACAAATGACGGTACTCTGTGAGGAAGCCACGGCTAACTACGTGCCAGCAGCCGCGGTAATACGTAGGTGGCGAGCGTTATCCGGAATCATTGGGCGTAAAGAGGGAGCAGGCGGCCTGGAGGGTCTATTGTTAAAAGGCAGTGGCTTAACCATTGCAAGGCGAAGAAACCGGCAGGCTAGAGTTCTAAAGAGGATCGTGGAATTCCATGTGTAGCGGTGAAATGCGTAGATATATGGAGGAACACCAGTGGCGAAGGCGACGATCTGGGAAGAAACAGACGCTGAGTCCCGAAAGCGTGGGGAGCAAATAGG

>OTU556GTGGGGAATCTTCCGCAATGGGCGAAAGCCTGACGGAGCAATGCCGCGTGAGTGAAGAAGGCCTTCGGGTTGTAAAACTCTGTCCTTATCGAAGAGAGGTGGGTATGTGAATAATGTACTCATAGGACGGTAGATAAGGAGGAAGCCCCGGCTAACTACGTGCCAGCAGCCGCGGTAATACGTAGGGGGCGAGCGTTGTCCGGAATCACTGGGCGTAAAGGGCGCGTAGGCGGTTTAATAAGTCAGTGGTGAAAACTGAGGGCTCAACCCTCAGCCTGCCACTGATACTGTTAGACTTGAGTATGGAAGAGGAGAATGGAATTCCTAGTGTAGCGGTGAAATGCGTAGATATTAGGAGGAACACCAGTGGCGAAGGCGATTCTCTGGGCCAAGACTGACGCTGAGGCGCGAAAGCGTGGGGAGCAAACAGG

>OTU557GTGGGGAATATTGGGCAATGGGCGAAAGCCTGACCCAGCAACGCCGCGTGAGGGAAGAAGGGTTTCGGCTCGTAAACCTCTGTCCTATGGGACGAAGGAAGTGACGGTACCATAGGAGGAAGCCCCGGCTAACTACGTGCCAGCAGCCGCGGTAATACGTAGGGGGCGAGCGTTGTCCGGAATGATTGGGCGTAAAGGGCGCGTAGGCGGCCTGATAAGTCTGGAGTGAAAGTCCTGCTTTCAAGGTGGGAATTGCTTTGGATACTGTCGGGCTTGAGTGCAGGAGAGGAAAGCGGAATTACCGGTGTAGCGGTGAAATGCGTAGAGATCGGTAGGAACACCAGTGGCGAAGGCGGCTTTCTGGACTGAAACTGACGCTGAGGCGCGAAAGCGTGGGGAGCAAACAGG

>OTU558GTGAGGAATATTGGTCAATGGCCGGAAGGCTGAACCAGCCAAGTCGCGTGAGGGAATAAGGCCCTACGGGTCGTAAACCTCTTTTGTCAGGGAGCAAAGCCTATTACGTGTAATGGGTCAGAGAGTACCTGAAGAAAAAGCATCGGCTAACTCCGTGCCAGCAGCCGCGGTAATACGGAGGATGCGAGCGTTATCCGGATTTATTGGGTTTAAAGGGTGCGTAGGCGGACTGTTAAGTCAGCGGTAAAATTGAGAGGCTCAACCTCTTCGAGCCGTTGAAACTGGCGGTCTTGAGTGAGCGAGAAGTATGCGGAATGCGTGGTGTAGCGGTGAAATGCATAGATATCACGCAGAACTCCGATTGCGAAGGCAGCATACCGGCGCTCAACTGACGCTGAAGCACGAAAGCGTGGGTATCGAACAGG

>OTU559GTGGGGAATATTGGACAATGGGGGCAACCCTGATCCAGCAATACCGCGTGTGTGAAGAAGGCCTTCGGGTTGTAAAGCACTTTAGGCGGGGAAGAAGGTAGGTATTTTAAGAGATAACTTACTTGACGGTACCCGCAGAATAAGCACCGGCTAACTCTGTGCCAGCAGCCGCGGTAATACAGAGGGTGCAAGCGTTAATCGGATTTACTGGGCGTAAAGGGCGCGTAGGTGGTTGCGTAAGTGAGATGTGAAAGCCCCGGGCTCAACCTGGGAACTGCATCTTATACTGCGTAGCTGGAGTGAAGTAGAGGGTAGTGGAATTTCCGGTGTAGCGGTGAAATGCGTAGATATCGGAAGGAACACCAGTGGCGAAGGCGGCTACCTGGACTTACACTGACACTGAGGCGCGAAAGCGTGGGTAGCAAACAGG

>OTU560GTGGGGAATATTGCGCAATGGAGGAAACTCTGACGCAGCGATGCCGCGTGGGCGAAGAAGGGCTCCGGCCCGTAAAGCCCTGCCGCCGGGGAAGAGAGAAGACGGTACCCGGCAAGCAAGCCCCGGCCAATCACGTGCCAGCAGCCGCGGTAAGACGTGAGGGGCGAGCGTTGTCCGGAATCACTGGGCGTAAAGGGCGCGCAGGCGGCTGCGCGGGTGGGATGTCAAAGCCCGGGGCCCAACCCCGGAGAGGCCTTCCAAACCGCGCGGCTCGGGTGCTGGAGGGGCAGGCAGAATTCCTGGTGTAGCGGTGAAATGCGCAGAGATCAGGAGGAATACCAGAGGCGAAGGCGGCCTGCCGGACAGACACTGACGCTTAGGCGCGAAAGCGCGGGGAGCAAACAGG

>OTU561GTGAGGAATATTGCGCAATGGGGGAAACCCTGACGCAGCAACGCCGCGTGGAGGATGAAGTCCCTTGGGATGTAAACTCCTTTCGATCGGGACGATAATGACGGTACCGGAAGAAGAAGCACCGGCTAACTCTGTGCCAGCAGCCGCGGTAATACAGAGGGTGCAAGCGTTGTTCGGAATTATTGGGCGTAAAGGGCGCGTAGGCGGTGCGGTAAGTCTGCGGTGAAATCTCCGGGCTCAACTCGGAAATTGCCGTGGAAACTGCCGTGCTGGAGTGTGGGAGAGGTGAGTGGAATTCCCGGTGTAGCGGTGAAATGCGTAGATATCGGGAGGAACACCTGCGGCGAAAGCGGCTCACTGGACCACAACTGACGCTGAGGCGCGAAAGCTAGGGGAGCAAACAGG

>OTU562GTGGGGAATATTGCGCAATGGGCGAAAGCCTGACGCAGCGACGCCGCGTGAGGGATGAAGGCCTTCGGGTTGTAAACCTCTTTCAGCAGGGACGAAGCGAGAGTGACGGTACCTGCAGAAGAAGCACCGGCCAACTACGTGCCAGCAGCCGCGGTAATACGTAGGGTGCAAGCGTTGTCCGGAATTATTGGGCGTAAAGAGCTCGTAGGCGGTCTGTCGCGTCGGCTGTGAAAACTCGGGGCTCAACCCCGAGCCTGCAGTCGATACGGGCAGACTAGAGTGCTGTAGGGGAGACTGGAATTCCTGGTGTAGCGGTGAAATGCGCAGATATCAGGAGGAACACCGGTGGCGAAGGCGGGTCTCTGGGCAGTAACTGACGCTGAGGAGCGAAAGCGTGGGGAGCAAACAGG

>OTU563GTGGGGAATATTGCGCAATGGAGAAGAACTCTGACGCAGCAATACCGCGTGAGCGAAGAAGGTCTTCGGATCGTAAAGCTCTGTTATCAGGGAAGAAGAAAATGACGGTACCTGATGAGCAAGTCCCGGCTAACTACGTGCCAGCAGCCGCGGTAATACGTAGGGGACAAGCGTTGTCCGGAATCACTGGGCGTAAAGGGCGCGCAGGCGGTGATTTAAGCAGGATGTGAAAGGCTACGGCTCAACCGTAGACATGCACCCTGAACTGGGTCACTTGAGTATTGGAGAGGCAGGCAGAATTCCTGGTGTAGCGGTGAAATGCGTAGATATCAGGAGGAATACCGGAGGCGAAGGCGGCCTGCTGGACAAATACTGACGCTGAGGCGCGAAAGCGTGGGGAGCAAACAGG

>OTU564GTGGGGAATTTTGGACAATGGGGGAAACCCTGATCCAGCCATTCCGCGTGAGTGAAGAAGGCCTTCGGGTTGTAAAGCTCTTTTGTCCGGAGCGAAACGGTTACGGTGAATATCCGTGACTACTGACGGTACCGGAAGAATAAGCACCGGCTAACTACGTGCCAGCAGCCGCGGTAATACGTAGGGTGCGAGCGTTAATCGGAATTACTGGGCGTAAAGCGTGCGCAGGCGGGCTTTTAAGCCAGACGTGAAATCCCCGGGCTTAACCTGGGAATGGCGTTTGGGACTGGGAGTCTGGAGTACGGCAGAGGAGACTGGAATTCCTGGTGTAGCAGTGAAATGCGTAGATATCAGGAGGAACACCGATGGCGAAGGCAGGTCTCTGGGCTGATACTGACGCTCATGCACGAAAGCGTGGGGAGCAAACAGG

>OTU565GTGGGGAATATTGGACAATGGGCGCAAGCCTGATCCAGCCATACCGCGTGGGTGAAGAAGGCCTTCGGGTTGTAAAGCCCTTTTGTTGGGAAAGAAAAGCAGCCGGTTAATACCCGGTTGTTCTGACGGTACCCAAAGAATAAGCACCGGCTAACTTCGTGCCAGCAGCCGCGGTAATACGAAGGGTGCAAGCGTTACTCGGAATTACTGGGCGTAAAGCGTGCGTAGGTGGTTGTTTAAGTCTGTCGTGAAAGCCCTGGGCTCAACCTGGGAATGGCGATGGAAACTGGGCGACTAGAGTGTGGCAGAGGGTAGTGGAATTCCTGGTGTAGCAGTGAAATGCGTAGAGATCAGGAGGAACATCCGTGGCGAAGGCGACTGCCTGGGCCAACACTGACACTGAGGCACGAAAGCGTGGGGAGCAAACAGG

>OTU566GTGAGGGATATTCGGCAATGGGGGAAACCCTGACCGAGCAACGCCGCGTGAGGGAAGACGGTTTTCGGATTGTAAACCTCTGTCCTCTGTGAAGATAATGACGGTAACAGAGGAGGAAGCTCCGGCTAACTACGTGCCAGCAGCCGCGGTAATACGTAGGGAGCAAGCGTTGTCCGGATTTACTGGGTGTAAAGGGTGCGTAGGCGGATTGGCAAGTCAGAAGTGAAATCCATGGGCTTAACCCATGAACTGCTTTTGAAACTGTTAGTCTTGAGTGAAGTAGAGGTAGGCGGAATTCCCGGTGTAGCGGTGAAATGCGTAGAGATCGGGAGGAACACCAGTGGCGAAGGCGGCTTTCTGGACTGAAACTGACGCTGAGGCTCGAAAGCGTGGGGAGCAAACAGG

>OTU567GTCGGGAATTTTGCGCAATGGACGAAAGTCTGACGCAGCAACGCCGCGTGAGGGATGACGGCCTTCGGGTTGTAAACCTCTTTTGTCAGGGACGATGATGACGGTACCTGACGAATAAGTCACGGCTAACTACGTGCCAGCAGCCGCGGTAATACGTAGGTGACAAGCGTTGTCCGGATTTACTGGGCGTAAAGAGCGCGCAGGCGGTCGTTCAAGTCGAGTGTGAAAGCCCCCGGCTCAACTGGGGAGGGTCACTCGATACTGATCGACTCGAAGGCAGGAGAGGGAAGTGGAATTCCCGGTGTAGTGGTGAAATGCGTAGATATCGGGAGGAACACCAGTGGCGAAGGCGACTTCCTGGCCTGTTCTTGACGCTGAGGCGCGAAAGCTAGGGGAGCAAACGGG

>OTU568GTAGGGAATCTTCCGCAATGGACGCAAGTCTGACGGAGCAACGCCGCGTGAGTGAAGAAGGTTTTCGGATCGTAAAACTCTGTTGTTAGAGAAGAACAAGTGCTAGAGTAACTGTTAGCGCCTTGACGGTATCTAACCAGAAAGCCACGGCTAACTACGTGCCAGCAGCCGCGGTAATACGTAGGTGGCAAGCGTTGTCCGGATTTATTGGGCGTAAAGCGAGCGCAGGCGGTTCCTTAAGTCTGATGTGAAAGCCCCCGGCTCAACCGGGGAGGGTCATTGGAAACTGGGGAACTTGAGTGCAGAAGAGGAGAGTGGAATTCCATGTGTAGCGGTGAAATGCGTAGATATATGGAGGAACACCAGTGGCGAAGGCGACTCTCTGGTCTGTAACTGACGCTGAGGCTCGAAAGCGTGGGTAGCAAACAGG

>OTU569GTGAGGAATATTGGTCAATGGACGGAAGTCTGAACCAGCCATCCCGCGTGCAGGAAGACGGCGCTATGCGTTGTAAACTGCTTTTCCAGGGGAAGAAAATTTCGCACGAGTGCGGAACTGCCGGTACCTTGGGAATAAGCATCGGCTAACTCCGTGCCAGCAGCCGCGGTAATACGGAGGATGCTAGCGTTATCCGGATTTATTGGGTTTAAAGGGTGCGTAGGCGGGCTGATAAGTCAGTGGTGAAAACCTGCAGCTTAACTGTAGACGTGCCATTGATACTGTTAGTCTTGAGTGTGGTCAAGGTAGGCGGAATGTGTAATGTAGCGGTGAAATGCTTAGATATTACACAGAACACCGATTGCGAAGGCAGCTTGCTGGGCCATTACTGACGCTGATGCACGAAAGCGTGGGGAGCGAACAGG

>OTU570GTGGGGAATCTTGGACAATGGGGGCAACCCTGATCCAGCGACGCCGCGTGGGTGATGAAGGCCTTCGGGTTGTAAAGCCCTTTCACCGGGGACGATGATGACGGTACCCGGAGAAGAAGCACCGGCTAACTCCGTGCCAGCAGCCGCGGTAAGACGGAGGGTGCTAGCGTTGTTCGGAATCACTGGGCGTAAAGGGCGCGTAGGCGGCCAAGGCAGTCGGCTTTGTGAAAGCCCGGGGCTCAACCCCGGAGGTGCGGCCGATACTCCTTGGCTCGAGGCCGGAAGAGGAGGGTGGAATACCCAGTGTAGAGGTGAAATTCGTAGATATTGGGTGGAACACCGGTGGCGAAAGCGGCTCTCTGGTCCGGACCTGACGCTGAGGCGCGAAAGCGTGGGGAGCAAACAGG

>OTU571GTGGGGAATATTGGACAATGGGCGAAAGCCTGATCCAGCGACGCCGCGTGTGTGAAGAAGGCCTGCGGGTTGTAAAGCACTTTCGATGGGAACGAAATGTGCCGGGCTAATATCCCGGCAAGCTGACGTTACTCAATGAAGAAGCACCGGCTAACTCTGTGCCAGCAGCCGCGGTAATACAGAGGGTGCGAGCGTTAATCGGAATTACTGGGCGTAAAGCGCACGTAGGTGGCGACGTAAGTCGGGTGTGAAAGCCCCGGGCTCAACCTGGGAATTGCATTCGAGACTGCATTGCTGGAGTACGGAAGAGGAGGGCGGAATTCCCGGTGTAGCGGTGAAATGCGTAGATATCGGGAGGAACATCAGTGGCGAAGGCGGCCCTCTGGTCCAGTACTGACACTCAAGTGCGAAAGCGTGGGGAGCAAACAGG

>OTU573GTAACGAATCTTCCGCAATGCGCGCAAGCGTGACGGAGCAATGCCGCGTGCAGGATGAAGCCCTTCGGGGTGTAAACTGCTGTCAGGGATTAGGAACACAATGACCAATCCCAAAGGAAGAGACGACTAACTCTGTGCCAGCAGTCGCGGTAATACAGAGGTCTCGAGCGTTAGTCGGAATCACTGGGCTTAAAGCGTACGTAGGCGGATGTGCAGGCGTCGTGTGAAAGCCCTCGGCTCAACCGAGGAATTGCACGGCGAACCGCACGTCTTGAGACGAGTAGGGGCTGTCGGAACGATAGGTGGAGTGGTGAAATGCGTTGATATCTATCGGAACGCCGAAGGTGAAGACAGGCAGCTGGGCTCGTTCTGACGCTGAGGTACGAAAGCGTGGGTAGCGAACGGG

>OTU574GTCGAGAATCTTTCACAATGGGGGCAACCCTGATGGAGCGACGCCGCGTGGAGGATAAGGTCTTCGGATTGTAAACTCCTGTCATGCGCGAACAAGGTTGCCGGACTAATACCCCGGCAAATTGATGGTACCGCAAGAGGAAGAGACGGCTAACTCTGTGCCAGCAGCCGCGGTAATACAGAGGTCTCAAGCGTTGTTCGGAATCACTGGGCGTAAAGGGTGCGTAGGTGGCGTGGTAAGTCAGATGTGAAAGCCCAGGGCTCAACCCTGGAATTGCATCCGATACTACCATGCTAGAGTACTGAAGAGGTGACTAGAATTCTCGGTGTAGCAGTGAAATGCGTAGATATCGAGAGGAATACCAAAGGCGAAGGCAGGTCACTGGGCAGTTACTGACACTGAGGCACGAAGGCTAGGGGAGCAAACGGG

>OTU575GTGGGGAATTTTGGACAATGGGGGCAACCCTGATCCAGCCATGCCGCGTGCGGGAAGAAGGCCTTCGGGTTGTAAACCGCTTTTAGTCGGGAAGAAAAGGCACGTCCTAATACGGCGTGTTCTTGACGGTACCGGCGGAATAAGCACCGGCTAACTACGTGCCAGCAGCCGCGGTAATACGTAGGGTGCGAGCGTTAATCGGAATTACTGGGCGTAAAGCGTGCGCAGGCGGTTCGTCAAGTCTGATGTGAAAGCCCCGGGCTCAACCTGGGAATGGCATTGGAAACTGGCGAGCTGGAGTGCGGCAGAGGGGGGTGGAATTCCGCGTGTAGCAGTGAAATGCGTAGAGATGCGGAGGAACACCGATGGCGAAGGCAGCCCCCTGGGCCGACACTGACGCTCAGGCACGAAAGCGTGGGGAGCAAACAGG

>OTU576GTGGGGAATCTTGCGCAATGCGCGAAAGCGTGACGCAGCAACGCCGCGTGGGGGAAGAAGGCCTTCGGGTTGTAAACCCCTTTCAGGAGGGACGAAGCGTGGGCGGTTAATAGCCGATCCACGTGACGGTACCTCCAGAAGAAGCCCCGGCTAACTACGTGCCAGCAGCCGCGGTAATACGTAGGGGGCAAGCGTTGTCCGGAATCATTGGGCGTAAAGCGCGTGTAGGCGGTCCGGTAAGTCGGCTGTGAAAGTCCAGGGCTCAACCCTGGGATGCCGGTCGATACTGCCGGACTAGAGTTCGGAAGAGGCGAGTGGAATTCCCGGTGTAGCGGTGAAATGCGCAGATATCGGGAGGAACACCAATGGCGAAGGCAGCTCGCTGGGACGTTACTGACGCTGAGACGCGAAAGCGTGGGGAGCAAACAGG

>OTU577GTAGGGAATTTTCCACAATGGACGAAAGTCTGATGGAGCAATGCCGCGTGAGGGATGACGGTCTTCGGATTGTAAACCTCTGTTATAAGGGAAGAACACCCTGGATAGGAAATGATCCAGGATTGACGGTACCTTATCAGAAAGTCACGGCTAACTATGTGCCAGCAGCCGCGGTAATACATAGGTGGCGAGCGTTATCCGGATTTATTGGGCGTAAAGAGTGCACAGGTGGTTTATCAAGTCAGGCGTTAAAGCCCGGAGCTCAACTCCGGTGTGCGTCTGAAACTGATAGACTAGAGTATCGGTGGGGTTAGTAGAATTCCATGTGTAGCGGTGGAATGCATAAATATATGGAGGAATACCAGTGGCGAAGGCGGCTAACTGAACGATAACTGACACTTAAGCACGAAAGCGTGGGGAGCAAATAGG

>OTU578GTGGGGAATATTGGACAATGGGGGAAACCCTGATCCAGCAATGCCGCGTGAGTGATGAAGGCCTTAGGGTTGTAAAGCTCTTTCGGCGGGGACGATGATGACGGTACCCGCAGAAGAAGCCCCGGCTAACTTCGTGCCAGCAGCCGCGGTAAGACGAAGGGGGCTAGCGTTGTTCGGAATTACTGGGCGTAAAGCGCGCGTAGGCGGTCTTTCAAGTCAGGCGTGAAAGCCCCGGGCTCAACCCGGGAATAGCGCTTGATACTGTGAGACTCGAGACCGGGAGAGGATAGTGGAATTCCCAGTGTAGAGGTGAAATTCGTAGATATTGGGAAGAACACCAGTGGCGAAGGCGGCTATCTGGACCGGTACTGACGCTGAGGCGCGAAAGCGTGGGGAGCAAACAGG

>OTU579GTAGGGAATCTTCGTCAATGGGCGCAAGCCTGAACGAGCGATGCCGCGTGGGTGAAGGAGCTTTTCGGAGTGTAAAGCCCTGTTGTGGATGAAAAAGGGCGAGAGCAGGAAATGGCTTTTGTTTGATGGAAGTCCACGAGGAAGTCACGGCTAACTACGTGCCAGCAGCCGCGGTAATACGTAGGTGGCGAGCGTTATCCGGAATGATTGGGCGTAAAGGGTGCGCAGGCGGCCTGTTAAGTCTGCAGTAAAAGGGCGAAGCTCAACTTTGCCAGGCTGTGGAAACTGGCAGGCTGGAGGCCAGCAGAGGACGGCGGAACTCCATGTGTAGCGGTAAAATGCATAGAGATATGGAAGAACACCAGCGGCGAAGGCGGCCGTCTGGGCTGGAGCTGACGCTGAGGCACGAAAGCGTGGGGAGCAAACAGG

>OTU580GTGGGGAATCTTCCGCAATGGGCGAAAGCCTGACGGAGCAACGCCGCGTGAACGAAGAAGGTCTTCGGATCGTAAAGTTCTGTTGTTAAGGGCGAAGGATGCTTATTATAATACAGTAAGTATTTGACGGTACTTAACGAGGAAGCCACGGCTAACTACGTGCCAGCAGCCGCGGTAATACGTAGGTGGCAAGCGTTGTCCGGAATTATTGGGCGTAAAGGGAGCGCAGGCGGGAAATTAAGCGGATCTTAAAAGTGCGGGGCTCAACCTCGTGATGGGGTCCGAACTGATTTTCTTGAGTGCAGGAGAGGAAAGCGGAATTCCCAGTGTAGCGGTGAAATGCGTAGATATTGGGAAGAACACCAGTGGCGAAGGCGGCTTTCTGGACTGTAACTGACGCTGAGGCTCGAAAGCTAGGGTAGCGAACGGG

>OTU581GTGGGGAATATTGCACAATGGGGGAAACCCTGATGCAGCGACGCCGCGTGAGTGAAGAAGTTATTCGTAATGTAAAGCTCTATCAGCAGGGAAGAAAGTGACGGTACCTGAGTAAGAAGCACCGGCTAAATACGTGCCAGCAGCCGCGGTAATACGTATGGTGCAAGCGTTATCCGGATTTACTGGGTGTAAAGGGTGCGTAGGTGGCATGGCAAGTCAGAAGTGAAAGGCCGGGGCTCAACCCCGGGACTGCTTTTGAAACTGTCAGGCTGGAGTGCAGGAGAGGAAAGCGGAATTCCTAGTGTAGCGGTGAAATGCGTAGATATTAGGAGGAACACCAGTGGCGAAGGCGGCTTTCTGGACTGAAACTGACACTGAGGCACGAAAGCGTGGGGAGCAAACAGG

>OTU582GTGGGGAATATTGCACAATGGGCGCAAGCCTGATGCAGCGACGCCGCGTGAGGGATGACGGCCTTCGGGTTGTAAACCTCTGTTAGCATCGAAGAAGCGAAAGTGACGGTAGGTGCAGAGAAAGCGCCGGCTAACTACGTGCCAGCAGCCGCGGTAATACGTAGGGCGCGAGCGTTGTCCGGAATTATTGGGCGTAAAGAGCTTGTAGGCGGTTGGTCGCGTCTGCTGTGAAAGGCTGGGGCTTAACCCTGGTTTTGCAGTGGGTACGGGCTAACTAGAGTGCAGTAGGGGAGACTGGAATTCCTGGTGTAGCGGTGGAATGCGCAGATATCAGGAGGAACACCGATGGCGAAGGCAGGTCTCTGGGCTGTAACTGACGCTGAGAAGCGAAAGCATGGGGAGCGAACAGG

>OTU583GTGGGGAATATTGGACAATGGGGGCAACCCTGATCCAGCCATGCCGCGTGAGTGATGAAGGCCTTAGGGTTGTAAAGCTCTTTCGCCGGTGACGATAATGACGGTAACCGGAGAAGAAGCCCCGGCTAACTTCGTGCCAGCAGCCGCGGTAATACGAAGGGGGCTAGCGTTGTTCGGAATTACTGGGCGTAAAGCGCACGTAGGCGGATGTTTAAGTCGGGGGTGAAATCCCAGGGCTCAACCCTGGAATGGCCTTCGATACTGGGCATCTCGAGTCCGAGAGAGGTGAGTGGAATTCCGAGTGTAGAGGTGAAATTCGTAGATATTCGGAAGAACACCAGTGGCGAAGGCGGCTCACTGGCTCGGAACTGACGCTGAGGTGCGAAAGCGTGGGGAGCAAACAGG

>OTU584GTAGGGAATTTTCCACAATGGACGAAAGTCTGATGGAGCAACGCCGCGTGTGCGAAGAAGGCCTTCGGGTTGTAAAGCACTGTTGTAAGGGAAGAACAGATGTGAGAGGAAATGCTCATATTGTGACGGTACCTTACCAGAAAGCCACGGCTAACTACGTGCCAGCAGCCGCGGTAATACGTAGGTGGCGAGCGTTATCCGGAATTATTGGGCGTAAAGGGCGTGTAGGCGGTGTTTTAAGTCTGATGTGAAAGCCAACGGCTCAACCGTTGAAATGCATTGGAAACTGGAACACTTGAGTGTCGGAGAGGAAAGCGGAATTCCTAGTGTAGCGGTGAAATGCGTAGATATTAGGAGGAACACCAGTGGCGAAGGCGGCTTTCTGGACGACAACTGACGCTGAGGCGCGAAAGCGTGGGGAGCAAACAGG

>OTU585GTGGGGAATATTGCACAATGGGGGGAACCCTGATGCAGCGATGCCGCGTGGAGGAAGAAGGTTTTCGGATTGTAAACTCCTGTCGTAAGGGACGATAATGACGGTACCTTACAAGAAAGCTCCAGCTAACTACGTGCCAGCAGCCGCGGTAATACGTAGGGAGCGAGCGTTGTCCGGAATTACTGGGTGTAAAGGGCGTGTAGGCGGAGGAGCAAGTCAGAAGTGAAATCTCCGGGCTTAACCCGGAAACTGCTTTTGAAACTGTTCCCCTTGAGTACTGGAGAGGCAGGCGGAATTCCTAGTGTAGCGGTGAAATGCGTAGATATTAGGAGGAACACCAGTGGCGAAGGCGGCCTGCTGGACAGCAACTGACGCTGAGGCGCGAAAGCGTGGGGAGCAAACAGG

>OTU586GTGGGGAATCTTCCGCAATGGGCGAAAGCCTGACGGAGCGACGTCGCGTGAGTGAAGAAGGTCTTCGGACCGTAAAGCTCTTTTGTTGCAGGCGAAAGGACTTAAGAGGAAATGCTTAAGTTAAGACGGTATGGAACGAATAAGCCACGGCTAACTACGTGCCAGCAGCCGCGGTAATACGTAGGTGGCAAGCGTTGTCCGGAATTATTGGGCGTAAAGGGAGCGCAGGCGGAAGGACAAGCCGGATTTGAAAGTTCGGGGCTCAGCCCCGTGAGGGATCCGGAACTGTTCTCCTTGAGAGCGGGAGAAGGAAGCGGAATTCCTGGTGTAGCGGTGAAATGCGTAGATATTAGGAGGAACACCAGTGGCGAAGGCGGCTTGCTGGACGACAACTGACGCTGAGGCGCGAAAGCGTGGGGAGCAAACAGG

>OTU587TCCAGGCAGACGTGGCAGAGTTGAGAGTTCCATATCTTGATCCAAAGGCAGCCAGGAAGAGACTGTCTAATGTGGGCAGCCAAGGGGGAGCTTGAGGATAGGAACTCAAAGCCCGACCCCACAGTGATGAACTTCTGCCAACAAGGCCACACCTATTCTGACAAGGCCACACAACCTAATGTCACTTCCCAGGAGCCGGGGATATTTAAGCCAATACAATCCACTGTAACTTTAATTTTTTTCAAATCCTATTTTTCAATGCTTTAACCTTCCTTGTAACCCACCACCCACCAGAGGCAGTAAAAAGGAAAGGATATAAGGGAAGTAGACCTGTTTAGAAAGTTCTTTGGAGCAACTCCAATCTGTGTTGCCAGAAATTGGCAGTTTAGTACATGTGTCAGCAGCAGCAGCTCAATCCACGGGCAAACACTT

>OTU588GCTAGGAATTTTCCGCAATGGGCGAAAGCCTGACGGAGCGACGCTGCGTTGAGGATTGAAGGCCCTACGGGGTTGTAAACTCATTTTGTGCAGGACGAGAGAGGACTGTACTGCAAGAATAAGCCACGGCAAACTACGTGCCAGCAGCCGCGGTAATACGTAGGTGGCGAGTGTTGTTCGGAATCACTGGGCATAAAGGGTGCGTAGGCGGGAAATCAAGCAGGATGTGAAATCCTATGGCCCAACCATAGAATTGCATTCTGAACTGGTTTTCTTGAGTGCAGCAGGGGAGTGCGGAATTCCCGGTGTAGGGGTGAAATCTATAGATATCGGGAAGAACACCGGTCGCGAAGGCGGCACTCTGGGCTGTAACTGACGCTGAGGCACGAAAGCGTGGGTATCGAACCGG

>OTU589GTGGGGAATATTGCACAATGGGCGGAAGCCTGATGCAGCAACGCCGCGTGCGGGATGACGGCCTTCGGGTTGTAAACCGCTTTCAGCAGGGACGAAGCGTGAGTGACGGTACCTGCAGAAGAAGCACCGGCTAACTACGTGCCAGCAGCCGCGGTGATACGTAGGGTGCGAGCGTTGTCCGGATTTATTGGGCGTAAAGAGCTTGTAGGCGGTTGGTTGCGTCGAAAGTGCAAACTCAGGGCTTAACTCTGAGCCTGCTTTCGATACGGGCTGACTGGAGGAAGGTAGGGGAGAATGGAATTCCCGGTGGAGCGGTGGAATGCGCAGATATCGGGAGGAACACCAGTGGCGAAGGCGGTTCTCTGGACCTTTCCTGACGCTGAGAAGCGAAAGCGTGGGGAGCAAACAGG

>OTU590GTGAGGAATATTGCACAATGGGCGGAAGCCTGATGCAGCAACGCCGCGTGCGGGACGAAGGCCTTCGGGTTGTAAACCGCTTTTCTGAGGGACGAGGCAGGACGGTACCTCAGGAATAAGTCTCGGCTAACTACGTGCCAGCAGCCGCGGTAAAACGTAGGAGGCGAGCGTTATCCGGATTTACTGGGCGTAAAGCGCACGTAGGCGGCTTTGCAAGTTAGAGGTGAAAGCCCGGGGCTCAACTCCGGAATTGCCTTTAAAACTGCATCGCTAGAGTTGTGGAGAGGTAAGTGGAATTCCGAGTGTAGAGGTGAAATTCGTAGATATTCGGAAGAACACCAGTGGCGAAGGCGACTTACTGGACACATACTGACGCTGAGGTGCGAAAGCGTGGGGAGCAAACAGG

>OTU591GTGGGGAATATTGCACAATGGGGGGAACCCTGATGCAGCGATGCCGCGTGGAGGAAGAAGGTTTTCGGATTGTAAACTCCTGTCGTAAGGGACGATAATGACGGTACCTTACAAGAAAGCTCCGGCTAACTACGTGCCAGCAGCCGCGGTAATACGTAGGGAGCAAGCGTTATCCGGATTTATTGGGTGTAAAGGGTGCGTAGACGGGAAAACAAGTTAGTTGTGAAATCCCTCGGCTTAACTGAGGAACTGCAACTAAAACTATTTTTCTTGAGTGTTGGAGAGGAAAGTGGAATTCCTAGTGTAGCGGTGAAATGCGTAGATATTAGGAGGAACACCAGTGGCGAAGGCGGCTTGCTGGACGACAACTGACGCTGAGGCGCGAAAGCGTGGGGAGCAAACAGG

>OTU592GTGGGGAATATTGGACAATGGGCGAAAGCCTGATCCAGCAATGCCGCGTGAGTGATGAAGGCCTTAGGGTTGTAAAGCTCTTTTACCCGGGATGATAATGACAGTACCGGGAGAATAAGCTCCGGCTAACTCCGTGCCAGCAGCCGCGGTAATACGGAGGGAGCTAGCGTTGTTCGGAATTACTGGGCGTAAAGCGCGCGTAGGCGGTTTTTTAAGTCAGAGGTGAAAGCCCGGGGCTCAACCCCGGAATTGCCTTTGAAACTGGAAAACTTGAATCTTGGAGAGGTCAGTGGAATTCCGAGTGTAGAGGTGAAATTCGTAGATATTCGGAAGAACACCAGTGGCGAAGGCGACTGACTGGACAAGTATTGACGCTGAGGTGCGAAAGCGTGGGGAGCAAACAGG

>OTU593GTGGGGAATATTGGACAATGGGCGAAAGCCTGATCCAGCAATGCCGCGTGAGTGATGAAGGCCTTAGGGTTGTAAAGCTCTTTTACCCGGGATGATAATGACAGTACCGGGAGAATAAGCCCCGGCTAACTCCGTGCCAGCAGCCGCGGTAATACGGAGGGAGCTAGCGTTGTTCGGAAATACTGGGCGTAAAGCGCACGTAGGCGGCGTCGTAAGTCAGGGGTGAAATCCCGGGGCTCAACCCCGGAACTGCCCTTGAAACTGCAGCGCTAGAATCTTGGAGAGGCGAGTGGAATTCCGAGTGTAGAGGTGAAATTCGTAGATATTCGGAAGAACACCAGTGGCGAAGGCGACTCGCTGGACAAGTATTGACGCTGAGGTGCGAAAGCGTGGGGAGCAAACAGG

>OTU594GTCGGGAATATTGCCCAATGCGCGAAAGCGTGAGGCAGCAACGCCGCGTGCGCGATGAAGGCCTTCGGGTCGTAAAGCGCTTTTCAGTGGGATGAGGAAGGACAGTACCACTGGAATAAGGTACGGCTAACTACGTGCCAGCAGCCGCGGTAAAACGTAGGTACCGAGCGTTATCCGGATTTACTGGGCGTAAAGCGTGTTCAGGCGGTCTGGCAAGTCGGGCATGAAATCTCTGGGCTCAACCCGGAGGGGTTGTCCGATACTGCTGGGCTGGAGGGCAGCAGAGGGTGGTGGAATTCCGGGTGTAGTGGTGAAATGCGTAGATATCGGGAGGAACACCAGCGGCGAAGGCGGCCGCCTGGACGGTTACTGACGCTGAGACGCGAAAGCGTGGGGAGCAAACAGG

>OTU595GTGAGGAATATTGGTCAATGGGCGAGAGCCTGAACCAGCCATCCCGCGTGCAGGATGACGGCCCTATGGGTTGTAAACTGCTTTTCTACGCCAAGAACAGCGCCGACGTGTCGGTGAATGACGGTAGCGTAGGAATAAGCATCGGCTAACTCCGTGCCAGCAGCCGCGGTAATACGGAGGATGCAAGCGTTATCCGGATTCATTGGGTTTAAAGGGTGCGCAGGCGGGTTTGTAAGTCAGTGGTGAAATACTGTCGCTTAACGATAGGGTTGCCATTGATACTGCAGATCTTGAGTTTGGTTGAGGTAGGCGGAATGTGCAGTGTAGCGGTGAAATGCATAGAGATTGCACAGAACACCGATTGCGAAGGCAGCTTACTAAGCCAAAACTGACGCTGAGGCACGAAAGCGTGGGGAGCGAACAGG

>OTU596GTGGGTAATCTTGCGCAATGGGCGAAAGCCTGACGCAGCGACGCCGCGTGGGGGAAGAAGGCCTTCGGGTTGTAAACCTCTTTCAGGGGGGACGAAGCCACTCGGGTTAATAGCCCAGAGGGTGACGGTACCTCCAGAAGAAGCCCCGGCTAACTACGTGCCAGCAGCCGCGGTAATACGTAGGGGGCAAGCGTTGTCCGGATTTATTGGGCGTAAAGAGCGTGTAGGCGGCCAGGTAGGTCGGTTGTGAAAACTAGAGGCTTAACCTCTAGACGTCGACCGAAACCATCTGGCTAGAGTCCGGAAGAGGAGAGTGGAATTCCCGGTGTAGCGGTGAAATGCGCAGATATCGGGAAGAACACCCGTGGCGAAGGCGGCTCTCTGGGACGGTACTGACGCTGAGACGCGAAAGCGTGGGGAGCAAACAGG

>OTU597GTGAGGAATATTGGTCAATGGGCGGGAGCCTGAACCAGCCAAGTCGCGTGAGGGATGACGGCCCTATGGGTTGTAAACCTCTTTTGCCGGGGAGCAACGGGGTCCTTGCGAGGGCCCTATGAGAGTACCCGGAGAAAAAGCATCGGCTAACTCCGTGCCAGCAGCCGCGGTAATACGGAGGATGCGAGCGTTATCCGGATTTATTGGGTTTAAAGGGAGCGTAGGCGGGTTGTTAAGTCAGTTGTGAAAGTTTGCGGCTCAACCGTAAAATTGCAGTTGATACTGGCATCCTTGAGTACAGTAGAGGTAGGCGGAATTCGTGGTGTAGCGGTGAAATGCTTAGATATCACGAAGAACTCCGATTGCGAAGGCAGCCTGCTGGACTGTAACTGACGCTGATGCTCGAAAGTGTGGGTATCAAACAGG

>OTU598GCAGGGAATCTTGCGCAATGGGCGAAAGCCTGACGCAGCAACGCCGCGTGGGGGATGACGGCCTTCGGGTTGTAAACCCCTTTCAGGAGGGAAGAAATTGACGGTACCTCCAGAAGAAGCCCCGGCCAACTACGTGCCAGCAGCCGCGGTAATACGTAGGGGGCAAGCGTTGTCCGGAATTATTGGGCGTAAAGAGCTCGTAGGCGGCTCAGCAAGTCGGCTGTGAAATCCCGAGGCTCAACCTCGGAACTGCAGTCGATACTGCTGTGGCTAGAGTCTGGTAGAGGAGAATGGAATTCCCGGTGTAGCGGTGAAATGCGCAGATATCGGGAGGAACACCAGTAGCGAAGGCGGTTCTCTGGGCCAGTACTGACGCTGAGGAGCGAAAGCGTGGGTAGCAAACAGG

>OTU599GTGAGGAATATTGCGCAATGGGCGAAAGCCTGACGCAGCCACGCCGCGTGTCGGAAGAAGGTCCTTTGGATCGTAAACGACTTTTATCGGGGAAGAAGTCCGGGGTTTCGACCCCGGGATGACGGTACCCGAAGAATAAGCACCGGCTAACTCCGTGCCAGCAGCCGCGGTAATACGGAGGGTGCAAGCGTTGTCCGGAATCATTGGGTGTAAAGGGTGCGTAGGCGGGTTTTTAAGTCTGGGGTGAAAGGCTGCGGCTCAACCGTAGGATGGCCCTGGATACTGGGAACCTTGAGTGTGACAGAGGCAGGTAGAATTCGTGGTGTAGCGGTGAAATGCATAGATATCACGAAGAATACCAGTGGCGAAGGCGGCCTGCTGGGTCACAACTGACGCTGAGGCACGAGAGCGTGGGGAGCAAACAGG

>OTU600GTGGGGAATCTTGCGCAATGGGCGAAAGCCTGACGCAGCGACGCCGCGTGCGGGAGGACGGCCTTCGGGTCGTAAACCGCTGTTGCCCGGGACGAAAAGCCCTTTCGAGGGCGTTGACGGTACCGGGTGAGGAAGCACCGGCTAACTCCGTGCCAGCAGCCGCGGTAATACGGAGGGTGCGAGCGTTGTCCGGAATCACTGGGCGTAAAGGGCGCGTAGGCGGCGCGACAAGGCGGCGGTGAAAGTCCGGGGCTCAACCCCGGATCGGCCGTGGCGACTGTCGTGCTTGAGCACAGTAGAGGCGGGTGGAATTCCGGGTGTAGCGGTGGAATGCGTAGAGATCCGGAAGAACACCGGTGGCGAAGGCGGCCCGCTGGGCTGTGGCTGACGCTGAGGCGCGACAGCGTGGGGAGCAAACAGG

>OTU601GTGGGGAATATTGGGCAATGGGCGCAAGCCTGACCCAGCAACGCCGCGTGAAGGAAGAAGGCTTTCGGGTTGTAAACTTCTTTTAAGAGGGAAGAGAAGAAGACGGTACCTCTTGAATAAGCCACGGCTAACTACGTGCCAGCAGCCGCGGTAATACGTAGGTGGCAAGCGTTATCCGGATTTACTGGGTGTAAAGGGCGTGCAGCCGGAGAGACAAGTCAGATGTGAAATCCACGGGCTCAACCCGTGAACTGCATTTGAAACTGTTTCCCTTGAGTGTCGGAGAGGTAATCGGAATTCCTTGTGTAGCGGTGAAATGCGTAGATATAAGGAAGAACACCAGTGGCGAAGGCGGCTTACTGGACAGTAACTGACGTTGAGGCTCGAAAGCGTGGGGAGCAAACAGG

>OTU602GTGGGGAATATTGGACAATGGGCGCAAGCCTGATCCAGCCATGCCGCGTGAGTGATGAAGGCCTTAGGGTTGTAAAGCTCTTTCGTCCGGGACGATAATGACGGTACCGGAAGAAGAAGCCCCGGCTAACTTCGTGCCAGCAGCCGCGGTAATACGAAGGGGGCTAGCGTTGCTCGGAATCACTGGGCGTAAAGGGCGCGTAGGCGGCCATTCAAGTCAGAGGTGAAAGCCCAAGGCTCAACCTTGGAATTGCCTTTGATACTGTTTGGCTTGAGACCGGAAGAGGTAAGTGGAACTGCGAGTGTAGAGGTGAAATTCGTAGATATTCGCAAGAACACCAGTGGCGAAGGCGGCTTACTGGTCCGGTTCTGACGCTGAGGCGCGAAAGCGTGGGGAGCAAACAGG

>OTU603GTGAGGAATATTGGTCAATGGGCGGGAGCCTGAACCAGCCAAGTCGCGTGAGGGATGACGGCCCTATGGGTTGTAAACCTCTTTTGCCGGGGAGCAAAGTGCCGCACGTGTGCGGTTTGGAGAGTACCCGGAGAAAAAGCATCGGCTAACTCCGTGCCAGCAGCCGCGGTAATACGGAGGATGCGAGCGTTATCCGGATTTATTGGGTTTAAAGGGTGCGTAGGCGGACGCTTAAGTCAGCGGTAAAATTGCGGGGCTCAACCTCGTCGAGCCGTTGAAACTGGGTGCCTTGAGTGGGCGAGAAGTACGCGGAATGCGTGGTGTAGCGGTGAAATGCATAGATATCACGCAGAACTCCGATTGCGAAGGCAGCGTACCGGCGCCCAACTGACGCTGAAGCACGAAGGCGTGGGTATCGAACAGG

>OTU604GTGGGGAATTTTGGACAATGGGCGCAAGCCTGATCCAGCCATGCCGCGTGAGTGAAGAAGGCCTTCGGGTTGTAAAGCTCTTTCGGTGGGGACGAAACGGTTCGTGCTAATACCACGGACTAATGACGGTACCCGAAGAAGAAGCACCGGCTAACTACGTGCCAGCAGCCGCGGTAATACGTAGGGTGCAGGCGTTAATCGGAATTACTGGGCGTAAAGCGTGCGCAGGCGGTTTGTTAAGACAGATGTGAAATCCCCGGGCTTAACCTGGGAATGGCAATGGATACTGGCGAGCTAGAGTGTGTCAGAGGATGGTGGAATTCCCGGTGTAGCAGTGAAATGCGTAGATATCGGGAGGAACATCTGTGGCGAAGGCGACTACCTGGACCAGCACTGACACTGAGGCACGAAAGCGTGGGGAGCAAACAGG

>OTU605GTGAGGAATCTTCCGCAATGGGCGAAAGCCTGACGGAGCGACGCCGCGTGTGGGATGAAGTCTTTCGGGATGTAAACCACTGTCGGAGGGGAAGACATCGACGGTACCCTCCAAGAAAGCCCCGGCTAACTACGTGCCAGCAGCCGCGGTAATACGTAGGGGGCAAGCGTTATCCAGATTTACTGGGCGTAAAGCGCGTGTAGGCGGCTGGTTAGGCGTGATGTGAAATCTTCCGGCTCAACCGGAAAACTGCATTGCGAACCGGCCTGGCTAGAGTGCAGGAGAGGGAAGCGGAATTCCAGGTGTAGCGGTGAAATGCGTAGATATCTGGAGGAACACCAGTGGCGAAGGCGGCTTCCTGGCCTGCAACTGACGCTGAGACGCGAAAGCGTGGGGAGCGAACCGG

>OTU606GTGGGGAATATTGGACAATGGGCGAAAGCCTGATCCAGCAACGCCGCGTGAGGGATGACGGCCTTCGGGTTGTAAACCTCTTTCAGCGGGGACGAAGCGAAAGTGACGGTACCCGCAGAAGAAGCACCGGCCAACTACGTGCCAGCAGCCGCGGTAATACGTAGGGTGCGAGCGTTGTCCGGAATTATTGGGCGTAAAGGGCTCGTAGGCGGTTTGTCACGTCGGGAGTGAAAACTCAGGGCTTAACCCTGAGCCTGCTTCCGATACGGGCAGACTAGAGGTATGCAGGGGAGAACGGAATTCCTGGTGTAGCGGTGAAATGCGCAGATATCAGGAGGAACACCGGTGGCGAAGGCGGTTCTCTGGGCATTACCTGACGCTGAGGAGCGAAAGTGTGGGGAGCGAACAGG

>OTU607GTGGGGAATTTTCCGCAATGGGCGAAAGCCTGACGGAGTGACGCCGCGTGGGGGACGAAGGCCTTCGGGTTGTAAACCCCTGTCAGAGGGAAAGAAGGGTGTCGGCGCGATAACCGCCGGCATTTGACGGTACCTTCAGAGGAAGCCCCGGCCAACTCTGTGCCAGCAGCCGCGGTAAGACAGAGGGGGCAAGCGTTGCTCGGAATCACTGGGCGTAAAGGGCGCGTAGGCGGGATGGCAAGTCAGTCGTGAAATCTCTCGGCTCAACCGAGAAACGTCGGCTGATACTGCCGTTCTTGAGGGATGCAGAGGAGACTGGAATTCCCGGTGTAGCGGTGAAATGTGTAGAGATCGGGAGGAACACCGGTGGCGAAGGCGGGTCTCTGGGCATCTCCTGACGCTGAGGCGCGAAAGCGTGGGGAGCAAACGGG

>OTU608GTGAGGAATATTGGTCAATGGCCGAAGGGCTGAACCAGCCAAGCCGCGTGAAGGATGAAGGCCCTACGGGTCGTAAACTTCTTTTGTCAGGGAACAAAAGCGGGGACGTGTCCCCGTCTGCGTGTACCTGAAGAAAAAGCATCGGCTAACTCCGTGCCAGCAGCCGCGGTAATACGGAGGATGCGAGCGTTATCCGGATTTATTGGGTTTAAAGGGTGCGCAGGCGGAAATGCAAGTCAGCGGTAAAATCGTGGAGCTCAACTCCATCCTGCCGTTGAAACTGTTTTTCTAGAGTGGGCGAGAAGTATGCGGAATGCGTGGTGTAGCGGTGAAATGCATAGATATCACGCAGAACGCCGATTGCGAAGGCAGCTTACCGGCGCTCAACTGACGCTGAGGCACGAAAGTGCGGGTATCGAACAGG

>OTU609GTGGGGAATTGTTCGCAATGGGCGCAAGCCTGACGACGCAACGCCGCGTGGAGGATGAAGATCTTCGGGTCGTAAACTCCTGTCGAGCGGGAAGAATGCCTCGTGGGTGAATAACCCGCGGGAATGACGGTACCGCTAAAGGAAGCCCCGGCTAACTCCGTGTCAGCAGCCGCGGTAATACGGGGGGGGCAAGCGTTGTTCGGAATTACTGGGCGTAAAGGGCTCGTAGGTGGCCAACTAAGTCGGACGTGAAATCCCCAGGCTCAACTTGGGAACTGCGTCCGATACTGGATGGCTTGAATTCGGGAGAGGGATGCAGAATTCCAGGTGTAGCGGTGAAATGCGTAGATATCTGGAGGAATACCGGTGGCGAAGGCGGCATCCTGGACCGACATTGACGCTGAACGGCGAAAGCTAGGGGAGCAAACGGG

>OTU610GTGGGGAATTTTTCGCAATGGGCGCAAGCCTGACGAAGCAACGCCGCGTGGAGGATGAAGGCCTTCGGGTTGTAAACTCCTGTCGACCGGGACGAAAGTAGTCCGACCCAATACGTCGGGCTATCGACTGTACCGGTGGAGGAAGCCACGGCTAACTCTGTGCCAGCAGCCGCGGTAATACAGAGGTGGCAAGCGTTGTTCGGAATTACTGGGCGTAAAGGGCGCGTAGGCGGCCTTCTAAGTCTTGTGTGAAATCCCTCGGCTCAACTGAGGAACTGCACGGGATACTGGATGGCTTGAGTTCGGGAGAGGGAAGCGGAATTCCGGGTGTAGCGGTGAAATGCGTAGATATCCGGAGGAACACCGGTGGCGAAGGCGGCTTCCTGGACCGATTACTGACGCTGAGGCGCGAAAGCTAGGGGAGCAAACGGG

>OTU611GTGGGGAATATTGGACAATGGGCGAAAGCCTGATCCAGCCATGCCGCGTGGGTGAAGAAGGCCTTAGGGTTGTAAACCCCTTTCAGCGGGGACGATAATGACGGTACCCGCAGAAGAAGTCCCGGCTAACTCCGTGCCAGCAGCCGCGGTAATACGGAGGGGACTAGCGTTGTTCGGAATTACTGGGCGTAAAGCGCACGTAGGCGGATTTGTAAGTTAGGGGTGAAATCCCGGGGCTCAACCCCGGAACTGCCCTCGAAACTAGGTAGCTGGAATCTTGGAGAGGCGAGTGGAATTCCGAGTGTAGAGGTGAAATTCGTAGATATTCGGAAGAACACCAGTGGCGAAGGCGACTCGCTGGACAAGTATTGACGCTGAGGTGCGAAAGCGTGGGGAGCAAACAGG

>OTU612GTGGGGAATATTGCACAATGGGGGGAACCCTGATGCAGCGACGCCGCGTGAGTGAAGAAGTACCTCGGTATGTAAAGCTCTGTCAGCAGGGAAGAAAGAAATGACGGTACCTGACCAAGAAGCACCGGCTAAATACGTGCCAGCAGCCGCGGTAATACGTATGGTGCAAGCGTTATCCGGATTTACTGGGTGTAAAGGGAGCGCAGGCGGCGCGGCAAGTCCGGTGTGAAAGCCCGTGGCCCAACCACGGTACTGCACTGGAAACTGTCGTGCTTGAGTGTCGGAGGGGCAGGCGGAATTCCTGGTGTAGCGGTGAAATGCGTAGATATCAGGAGGAACACCGGTGGCGAAGGCGGCCTGCTGGACGATAACTGACGCTGAGGCTCGAAAGCGTGGGGAGCAAACAGG

>OTU613GTGGGGAATATTGGGCAATGGGCGCAAGCCTGACCCAGCAACGCCGCGTGAAGGAAGAAGGCTTTCGGGTTGTAAACTTCTTTTGGCAGGGACGAAACAAATGACGGTACCTGCAGAATAAGCTCCGGCTAAATACGTGCCAGCAGCCGCGGTAATACGTATGGAGCAAGCGTTATCCGGATTTACTGGGTGTAAAGGGCGCGTAGGCGGGACTACAAGTCAGATGTGTAATCTGGAGGCTTAACCTCCAAACTGCATTTGAAACTGTAGTTCTTGAGTGATGGAGAGGCAAGTGGAATTCCTAGTGTAGCGGTGAAATGCGTAGATATTAGGAGGAACACCAGTGGCGAAGGCGACTTGCTGGACATTAACTGACGCTGAGGCGCGAAAGCGTGGGGAGCAAACAGG

>OTU615GTGGGGAATATTGGACAATGGGCGCAAGCCTGATCCAGCCATGCCGCGTGAGTGATGAAGGCCCTAGGGTTGTAAAGCTCTTTCACCGGAGAAGATAATGACGGTATCCGGAGAAGAAGCCCCGGCTAACTTCGTGCCAGCAGCCGCGGTAATACGAAGGGGGCTAGCGTTGTTCGGAATTACTGGGCGTAAAGCGCACGTAGGCGGATCGATCAGTCAGGGGTGAAATCCCAGGGCTCAACCCTGGAACTGCCTTTGATACTGTCGATCTGGAGTATGGAAGAGGTGAGTGGAATTCCGAGTGTAGAGGTGAAATTCGTAGATATTCGGAGGAACACCAGTGGCGAAGGCGGCTCACTGGTCCATTACTGACGCTGAGGTGCGAAAGCGTGGGGAGCAAACAGG

>OTU616GTGGGGAATATTGCACAATGGGCGAAAGCCTGATGCAGCGACGCCGCGTGCGGGATGACGGCCTTCGGGTTGTAAACCGCTTTCAGCTCTGACGAAGCTTTTGTGACGGTAGGAGCAGAAGAAGCACCGGCTAACTACGTGCCAGCAGCCGCGGTAATACGTAGGGTGCGAGCGTTGTCCGGAATTATTGGGCGTAAAGAGCTTGTAGGCGGCTTGTCGCGTCTGCTGTGAAAACCCGGGGCTTAACCCCGGGCTTGCAGTGGGTACGGGCAGGCTAGAGTGTGGTAGGGGAGACTGGAATTCCTGGTGTAGCGGTGGAATGCGCAGATATCAGGAGGAACACCGATGGCGAAGGCAGGTCTCTGGGCCATTACTGACGCTGAGAAGCGAAAGCGTGGGGAGCGAACAGG

>OTU617GTAGGGAATTTTCGTCAATGGGCGCAAGCCTGAACGAGCAATGCCGCGTGAGTGAGGAAGGTCTTCGGACTGTAAAGCTCTGTTGCCGGGGAAAAAGGGGAAATGCAGGAAATGGCATTTCATGGATGGTACCCGGCCAGAAAGCCACGGCTAACTACGTGCCAGCAGCCGCGGTAATACGTAGGTGGCGAGCGTTATCCGGAATGATTGGGCGTAAAGGGTGCGCAGGCGGTCTGTTAAGTCTGAAGGCAAAGGGATGAGCTCAACTCATTTATGATTCAGAAACTGGCAGACTTGAGGACAGGAGAGGGCGGTGGAACTCCATGTGTAGCGGTAAAATGCGCAGAGATATGGAAGAACACCAGTGGCGAAGGCGGCCGCCTGGCCTGTGACTGACGCTGAGGCACGAAAGCGTGGGGAGCAAATAGG

>OTU618GTGGGGAATATTGGGCAATGGGCGCAAGCCTGACCCAGCAACGCCGCGTGAAGGAAGAAGGCTTTCGGGTTGTAAACTTCTTTGACAGGGGAAGATAATGACGGTACCCTGAAAACAAGCTCCGGCTAACTACGTGCCAGCAGCCGCGGTGATACGTAGGGAGCAAGCGTTATCCGGAATTACTGGGTGTAAAGGGCGCGTAGGCGGGCAAGCAAGTCAGATGTGAAATGCCGGGGCTTAACTCCGGAGCTGCATTTGAAACTGTTAGTCTTGAGTGATGGAGAGGCAGGCGGAATTCCCGGTGTAGCGGTGAAATGCGTAGAGATCAGGAGGAACATCGGTGGCGAAGGCGGCTTACTGGGCCTTTACTGACGCTGAGGCTCGAAAGCGTGGGGAGCAAACAGG

>OTU619GTCGAGGATCATTCGCAATGGGCGCAAGCCTGACGGTGCGACGCCGCGTGTAGGATGAAGGCCTTCGGGTCGTAAACTACTGTCATGAGGGAGCAACGGTTCTGGTGTTAACACCACCGGGAAGTGATAGTACCTCAGGAGGAAGCCACGGCTAACTCTGTGCCAGCAGCCGCGGTAATACAGAGGCGGCAAGCGTTAATCGGAATTACTGGGCGTAAAGCGCGCGTAGGTGGTTTTGTAAGTTGGATGTGAAAGCCCCGGGCTCAACCTGGGAACTGCATCCAAAACTGCATGACTAGAGTACGGTAGAGGGTGGTGGAATTTCCTGTGTAGCGGTGAAATGCGTAGATATAGGAAGGAACACCAGTGGCGAAGGCGACCACCTGGACTGATACTGACACTGAGGTGCGAAAGCGTGGGGAGCAAACAGG

>OTU620GTGGGGAATATTGGACAATGGGCGAAAGCCTGATCCAGCCATGCCGCGTGTGTGAAGAAGGTCTTCGGATTGTAAAGCACTTTAAGTTGGGAGGAAGGGCTGCTGGTTAATACCCTGCAGTTTTGACGTTACCAACAGAATAAGCACCGGCTAACTTCGTGCCAGCAGCCGCGGTAATACGAAGGGTGCAAGCGTTACTCGGAATTACTGGGCGTAAAGCGTGCGTAGGTGGTGGTTTAAGTCCGTTGTGAAAGCCCCGGGCTCAACCTGGGAACTGCATCCAAAACTGCCCGACTAGAGTACGGTAGAGGGTGGTGGAATTTCCTGTGTAGCGGTGAAATGCGTAGATATAGGAAGGAACACCAGTGGCGAAGGCGACCACCTGGACTGATACTGACACTGAGGTGCGAAAGCGTGGGGAGCAAACAGG

>OTU621GTGGGGAATATTGCGCAATGGCCTAACGGCTGACGCAGCGACGCCGCGTGTGGGATGACGCCCTTCGGGGTGTAAACCACTGTTGCCCGGGACGAACCTCTGGCTTCAGCCAGACTGACGGTACCGGGTGAGGAAGCACCGGCTAACTCCGTGCCAGCAGCCGCGGTAATACGGAGGGTGCGAGCGTTGTCCGGAATCACTGGGCGTAAAGGGCGCGTAGGTGGTTCGATAAGTGTGTGGTGAAAGCCCGGGGCTCAACCCCGGGTCTGCCGTGCAAACTGTCGGACTTGAGGACTGCAGAGGCAGGTGGAATTCCGGGTGTAGCGGTGGAATGCGTAGATATCCGGAAGAAGACCGGTGGCGAAGGCGACCTGCTGGGCAGTAACTGACACTGAGGCGCGACAGCGTGGGGAGCAAACAGG

>OTU622GTGGGGAATATTGCACAATGGGGGAAACCCTGATGCAGCGACGCCGCGTGAGTGAAGAAGTAATTCGTTATGTAAAGCTCTATCAGCAGGGAAGAAAATGACGGTACCTGATTAAGAAGCCCCGGCTAACTACGTGCCAGCAGCCGCGGTAATACGTAGGGGGCAAGCGTTATCCGGATTTACTGGGTGTAAAGGGAGCGTAGACGGTGATGCAAGTCAGAAGTGAAAGCCCGGGGCTCAACTCCGGGACTGCTTTTGAAACTGTGTGACTGGAGTGCAGGAGAGGTAAGCGGAATTCCTGGTGTAGCGGTGAAATGCGTAGATATCAGGAGGAACACCAGTGGCGAAGGCGGCTTACTGGACTGTAACTGACGTTGAGGCTCGAAAGCGTGGGGAGCAAACAGG

>OTU623GTGAGGAATATTGGTCAATGGGCGCAAGCCTGAACCAGCCATCCCGCGTGAAGGAAGAAGGCGCTATGCGTTGTAAACTTCTTTTCCAGGGGGAGAATTTTTCCGTCGTGACGGGAATTGACGGTACTCTGGGAATAAGCATCGGCTAACTCCGTGCCAGCAGCCGCGGTAATACGGAGGATGCGAGCGTTATCCGGATTTATTGGGTTTAAAGGGTGCGTAGGCGGATTGATAAGTCAGTGGTGAAAGGCTGCAGCTTAACTGTAAAAATGCCGTTGAAACTGTCGGACTTGAGTGTAAATGAGGTAGGCGGAATGCGTGGTGTAGCGGTGAAATGCATAGATATCACGCAGAACTCCGATTGCGAAGGCAGCTTACTAAGCTACAACTGACACTGAAGCACGAAAGCGTGGGGATCAAACAGG

>OTU624GTGGGGAATATTGCACAATGGGCGCAAGCCTGATGCAGCGATGCCGCGTGAGGGAAGAAGGTTTTCGGATTGTAAACCTCTGTTGACAGGGAAGATAATGACTGTACCTGTTCAGAAAGCTCCGGCTAACTACGTGCCAGCAGCCGCGGTAATACGTAGGGAGCGAGCGTTGTCCGGAATTACTGGGTGTAAAGGGAGTGTAGGCGGGCTGTTAAGTCAGCGGTCAAATCGTGCGGCTCAACCGTACCTTGCCGTTGAAACTGGTGGTCTTGAGTTCGGACAGGGCAGATGGAATTCGTGGTGTAGCGGTGAAATGCATAGATATCACGCAGAACTCCGATTGCGAAGGCAGCATACCGGCGCTCAACTGACGCTGAGGCACGAAAGCGTGGGGATCGAACAGG

>OTU625GTGAGGAATATTCCACAATGGGCGAAAGCCTGATGGAGCAATGCCGCGTGCAGGATGAAGGCCCTCGGGTTGTAAACTGCTTTTATATGAGAAGACTATGACGGTAACATATGAATAAGGACCGGCTAACTACGTGCCAGCAGCCGCGGTCATACGTAGGGTCCAAGCGTTATCCGGAGTGACTGGGCGTAAAGAGTTGCGTAGGCGGTTATGTAAGCGAGTGATGAAAACTATCGGCTCAACCGATAGCCTATTATTCGAACTGCATAACTCGAGAGTATCAGAGGTCGCTGGAATTCCTAGTGTAGGAGTGAAATCCGTAGATATTAGGAGGAACACCAATGGCGTAGGCAGGCGACTGGGATATTTCTGACGCTAAGGCACGAAAGCGTGGGGAGCGAACCGG

>OTU626GTCGGGAATATTGGGCAATGGGCGAAAGCCTGACCCAGCAATGCCGCGTGAGTGATGAAGGTCTTCGGATCGTAAAGCTCTGTCTTCAGGGACGAACAAGGTGACGGTACCTGAGGAGGAAGCCCCGGCTAACTACGTGCCAGCAGCCGCGGTAATACGTAGGGGGCGAGCGTTGTCCGGAATTACTGGGCGTAAAGGGCGAGTAGGCGGTTTTACAAGTCAGATGTGAAATACCAGGGCTCAACCCTGGGGCTGCATTTGAAACTGTAAGACTTGAGTGCAGGAGAGGAAAGTGGAATCCCCAGTGTAGCGGTGAAATGCGTAGATATTGGGGGGAACACCAGTGGCGAAGGCGACTTTCTGGACTGTAACTGACGCTCATGCACGAAAGCGTGGGGAGCAAACAGG

>OTU627GTGAGGAATATTGGTCAATGGGCGGAAGCCTGAACCAGCCAAGTCGCGTGAGGGAATAAGGCCCTACGGGTCGTAAACCTCTTTTGCCGGGGAGCAAGGGCCGGGACGTGTCCCGGCCCGGAGAGTACCCGGAGAAAAAGCATCGGCTAACTCCGTGCCAGCAGCCGCGGTAATACGGAGGATGCGAGCGTTATCCGGATTTATTGGGTTTAAAGGGTGCGTAGGCGGGCTTTTAAGTCAGCGGTAAAAATTCGGGGCTCAACCCCGTCCGGCCGTTGAAACTGGGGGCCTTGAGTGGGCGAGAAGAAGGCGGAATGCGTGGTGTAGCGGTGAAATGCATAGATATCACGCAGAACCCCGATTGCGAAGGCAGCCTTCCGGCGCCCTACTGACGCTGAGGCACGAAAGTGCGGGGATCGAACAGG

>OTU628GTGGGGAATATTGGACAATGGACCAAAAGTCTGATCCAGCAATTCTGTGTGCACGATGAAGGTCTTCGGATTGTAAAGTGCTTTCAGGTGGGAAGAAGAAAGTGACGGTACCACCAGAAGAAGCGACGGCTAAATACGTGCCAGCAGCCGCGGTAATACGTATGTCGCAAGCGTTATCCGGAATTATTGGGCGTAAAGCGAGTCTAGGCGGCTTGTTAAGTCAGATGTGAAAATGCGGGGCTCAACTCCGTATTGCGTTTGAAACTGGCAGGCTAGAGTACTGGAGAGGTGGGCGGAACTACAAGTGTAGAGGTGAAATTCGTAGATATTTGTAGGAATGCCGATAGTGAAGACAGCTCACTGGACAGATACTGACGCTAAAGCTCGAAAGCGTGGGGAGCAAACAGG

>OTU629GTAGGGAATATTGGTCAATGGATGCAAGTCTGAACCAGCCATGCCGCGTGCAGGAAGAAGGCCTTCTGGGTTGTAAACTGCTTTTGCCGGGGGATAAAAAGTCCATGCGTGGACAATTGAAGGTACCCGGTGAATAAGCCACGGCTAACTACGTGCCAGCAGCCGCGGTAATACGTAGGTGGCGAGCGTTGTCCGGATTTATTGGGTTTAAAGGGTGCGTAGGCGGCCTATTAAGTCAGTTCTGAAATATCCCGGCTTAACCGGGAGGGTGGGATTGATACTGATGGGCTTGAGTGGAATCGAGGTAGGCGGAATTGACGGTGTAGCGGTGAAATGCTTAGATATCGTCAAGAACACCGATAGTGTAGACAGCTTACTAGGCTTCAACTGACGCTGAGGCACGAAAGTGTGGGGATCAAACAGG

>OTU630GTGGGGAATATTGCACAATGGGGGAAACCCTGATGCAGCGACGCCGCGTGAGTGAAGAAGTATTTCGGTATGTAAAGCTCTATCAGCAGGGACGATAATGACGGTACCTGACTAAGAAGCCCCGGCTAACTACGTGCCAGCAGCCGCGGTAATACGTAGGGGGCAAGCGTTATCCGGATTTACTGGGTGTAAAGGGAGCGTAGACGGTGTGGTAAGTCAGGTGTGAAAGCCCGGGGCTCAACCCCGGGATTGCACTTGAAACTATCATGCTAGAGTGCAGGAGAGGTAAGTGGAATTCCTAGTGTAGCGGTGAAATGCGTAGATATTAGGAGGAACACCAGTGGCGAAGGCGGCTTACTGGACTGTAACTGACGTTGAGGCTCGAAAGCGTGGGGAGCAAACAGG

>OTU631GTAGGGAATCTTCCACAATGGGCGCAAGCCTGATGGAGCAACGCCGCGTGAGTGATGAAGGCCTTCGGGTTGTAAAACTCTGTCTTCTGTGACGAACAAGCCTGTGAGTGGAAAACTCAGGCCCTGACGGTAACAGAGGAGGAAGCCACGGCTAACTACGTGCCAGCAGCCGCGGTAATACGTAGGTGGCGAGCGTTGTCCGGATTTACTGGGCGTAAAGGGAGCGTAGGCGGATTCTTAAGTGGGATGTGAAATACCCGGGCTTAACCTGGGTGCTGCATTCCAAACTGGGAATCTAGAGTGCAGGAGGGGAGAGTGGAATTCCTAGTGTAGCGGTGAAATGCGTAGAGATTAGGAAGAACACCAGTGGCGAAGGCGACTCTCTGGACTGTAACTGACGCTGAGGCTCGAAAGCGTGGGGAGCAAACAGG

>OTU632GTGGGGAATTTTGGACAATGGGCGCAAGCCTGATCCAGCCATGCCGCGTGCGGGAAGAAGGCCTTCGGGTTGTAAACCGCTTTTGTCAGGGAAGAAACGGTCTGATCCAATACATCGGACTAATGACGGTACCTGAAGAATAAGCACCGGCTAACTACGTGCCAGCAGCCGCGGTAATACGTAGGGTGCAAGCGTTAATCGGAATTACTGGGCGTAAAGCGTGCGCAGGCGGCTATGCAAGACAGATGTGAAATCCCCGGGCTCAACCTGGGAACTGCATTTGTGACTGCATGGCTAGAGTGCGGCAGAGGGGGATGGAATTCCGCGTGTAGCAGTGAAATGCGTAGATATGCGGAGGAACACCGATGGCGAAGGCAATCCCCTGGGCCTGCACTGACGCTCATGCACGAAAGCGTGGGGAGCAAACAGG

>OTU633GTGGGGAATATTGGGCAATGGGCGAAAGCCTGACCCAGCCACGCCGCGTGAGTGATGAAGGCCTTCGGGTCGTAAAGCTCTGTGGGGAGGGAAGAAACCTTCCAGGGAACAATACCCCTGGGACTTGACGGTACCTCCTTAGCAAGCACCGGCTAACTCCGTGCCAGCAGCCGCGGTAATACGGAGGGTGCAAACGTTGCTCGGAATCATTGGGCGTAAAGCGTGTGTAGGCGGCCTGTTAAGTCGGGTGTGAAAGCCCCGGGCTCAACCCGGGAAGTGCATTCGATACTGGCAGGCTTGAGTACGGGAGAGGGTCGCGGAATTCCCGGTGTAGAGGTGAAATTCGTAGATATCGGGAGGAACACCAGTGGCGAAGGCGGCGACCTGGACCGATACTGACGCTGAGACACGAAAGCGTGGGGAGCAAACAGG

>OTU634GTCGGGAATTTTGCTCAATGGGCGAAAGCCTGAAGCAGCAACGCCGCGTGAGGGATGAAGGCCTTCGGGTTGTAAACCTCTTTTCTCAGGGAAGATTATGACGGTACCTGAGGAATAAGCCACGGCTAACTACGTGCCAGCAGCCGCGGTAATACGTAGGTGGCAAGCGTTGTCCGGATTTACTGGGCGTAAAGAGCGCGCAGGCGGTCGAGTAAGTCGAATGTGAAAGCCCCCGGCTCAACTGGGGAGGGTCATTCGATACTGTTCGACTCGAAGGCAGGAGAGGGAAGTGGAATTCCCGGTGTAGTGGTGAAATGCGTAGATATCGGGAGGAACACCAGTGGCGAAGGCGACTTCCTGGCCTGTTCTTGACGCTGAGGCGCGAAAGCTGGGGGAGCAAACGGG

>OTU635GTAAGGAATATTGGACAATGCCCGCAAGGGTGATCCAGCCATGCCGCGTGCAGGAAGACGGCCCTATGGGTTGTAAACTGCTTTTACACCGGAGAAAACCCCAGTACGTGTACTGGGCTGATAGTATGGTGAGAATAAGCATCGGCTAACTTCGTGCCAGCAGCCGCGGTAAGACGAAGGATGCAAGCGTTATCCGGATTCATTGGGTTTAAAGGGAGCGTAGGTGGCCTTATAAGTCAGTGGTGAAATCTTCGAGCTTAACTCGGAAATTGCCATTGATACTGTAGGGCTTGAGTATAGTTGCCGTTGGCGGAATATGACATGTAGTGGTGAAATACATAGAGATGTCATAGAACACCGATTGCGAAGGCAGCTAACGAAACTATAACTGACACTGAGGCTCGAAAGTGCGGGGATCAAACAGG

>OTU636GTGGGGAATATTGGACAATGGGCGCAAGCCTGATCCAGCCATGCCGCGTGTGTGAAGAAGGCCTTCGGGTTGTAAAGCACTTTTGTCCGGAAAGAAAAGCGCTCGATTAATACTCGGGTGTTATGACGGTACCGGAAGAATAAGCACCGGCTAACTTCGTGCCAGCAGCCGCGGTAATACGAAGGGTGCAAGCGTTACTCGGAATTACTGGGCGTAAAGCGTGCGTAGGTGGTTTGTTAAGTCTGATGTGAAAGCCCTGGGCTCAACCTGGGAACTGCATTGGAAACTGGCTTACTAGAGTGCGGTAGAGGGGTGTGGAATTCCCGGTGTAGCAGTGAAATGCGTAGATATCGGGAGGAACATCTGTGGCGAAGGCGACACCCTGGACCAGCACTGACACTGAGGCACGAAAGCGTGGGGAGCAAACAGG

>OTU637GTAGGGAATATTGGTCAATGGAGGCAACTCTGAACCAGCCATGCCGCGTGGAGGATGAATGCCCTCTGGGTTGTAAACTCCTTTTATCTGGGAAGAAACTCCTTGACTTTGTCAAGGCCTGACGGTACCAGAGGAATAAGCACCGGCTAACTCCGTGCCAGCAGCCGCGGTAATACGGAGGGTGCAAGCGTTATCCGGATTCACTGGGTTTAAAGGGTGCGTAGGCGGGTTGATAAGTCAGTGGTGAAAGCCCCAAGCTTAACTTGGGAATTGCCATTGATACTGTCAGTCTTGAATACGGTTGAGGTAGGCGGAATATAGCATGTAGCGGTGAAATGCTTAGATATGCTATAGAACACCGATTGCGAAGGCAGCTTACTAAGCCGTTATTGACGCTGAGGCACGAAAGCGTGGGGAGCAAACAGG

>OTU638GTGGGGAATCTTGGACAATGGGCGAAAGCCTGATCCAGCCATGCCGCGTGAGTGAAGAAGGCCTTAGGGTTGTAAAGCTCTTTTGCCCGGGAAGATAATGACGGTACCGGGAGAATAAGCCCCGGCAAACTTCGTGCCAGCAGCCGCGGTAATACGAAGGGGGCTAGCGTTGTTCGGATTTACTGGGCGTAAAGCGCACGTAGGCGGATTGTTAAGTCGGGGGTGAAATCCTGGAGCTCAACTCCAGAATTGCCTTCGATACTGGCTTTCTAGAGGCTGGAAGAGGTTAGTGGAATTCCCAGTGTAGAGGTGAAATTCGTAGATATTGGGAAGAACACCAGTGGCGAAGGCGGCTAACTGGTCCAGATCTGACGCTGAGGTGCGAAAGCGTGGGGAGCAAACAGG

>OTU639GTGGGGAATTGTTCGCAATGGGCGCAAGCCTGACGACGCAACGCCGCGTGGGGGATGAAGGTCTTCGGATTGTAAACCCCTGTCGAGCGGGACGAATGGCCAGTGACCTAACACGTCGCGGGAGTGACGGTACCGCTAAAGGAAGCCACGGCTAACTCTGTGCCAGCAGCCGCGGTAATACAGAGGTGGCAAGCGTTGTTCGGAATTACTGGGCGTAAAGGGCGCGTAGGCGGCCTGCTAAGTCAGACGTGAAATCCCCCGGCTCAACCTGGGAACTGCGTCTGATACTGGAAGGCTTGAATGCGGGAGAGGGATGCAGAATTCCAGGTGTAGCGGTGAAATGCGTAGATATCTGGAGGAATACCGGTGGCGAAGGCGGCATCCTGGACCGACATTGACGCTGAGGCGCGAAAGCTAGGGTAGCAAACGGG

>OTU640GTAGGGAATATTGGACAATGGGCGAAAGCCTGATCCAGCCATGCCGCGTGGATGACGAAGGCCTTCGGGTCGTAAAGTCCTTTTCTCAGGGAAGAACAAGTGAGAGAGTGGAAAGCTCTCGCCCTGACGGTACCTGAAGAAAAAGCATCGGCTAACTCCGTGCCAGCAGCCGCGGTAATACGGAGGATGCGAGCGTTATCCGGATTTATTGGGTTTAAAGGGTGCGTAGGCGGCGTGTTAAGTCAGCGGTAAAAGCCCGGGGCTCAACCCCGGCGAGCCGTTGAAACTGGCATGCTGGAGACGGCGAGAGGTACGCGGAATGCGCGGTGTAGCGGTGAAATGCATAGATATCGCGCAGAACCCCGATTGCGAAGGCAGCGTACCGGCGCCGGACTGACGCTGAGGCACGAAAGCGTGGGTATCGAACAGG

>OTU641GTAGGGAATATTGGGCAATGGGCGAGAGCCTGACCCAGCCATGCCGCGTGCAGGAAGACGGCGTTCTGCGTTGTAAACTGCTTTTATACGGGAAGAAAAAGCCACTGCGGTGGAAATTGCCGGTACCGTATGAATAAGCACCGGCTAACTCCGTGCCAGCAGCCGCGGTAATACGGAGGGTGCAAGCGTTGTCCGGATTTATTGGGTTTAAAGGGTGCGTAGGCGGCCTTTTAAGTCAGCGGTGAAAGTTTAGGGCTCAACCCTGAAATTGCCGTTGATACTGGAAGGCTTGAGTGTCGATGGGGTACCTGGAATTGATGGTGTAGCGGTGAAATGCATAGATACCATCAGGAACACCGATAGCGAAGGCATGGTACTTATCGACAACTGACGCTGAGGCACGAAAGCGTGGGTAGCGAACAGG

>OTU642GTGGGGAATCTTGCGCAATGGCCTAACGGCTGACGCAGCGACGCCGCGTGTGGGATGAAGCTCTTCGGAGTGTAAACCACTGTTGCCCGGGACGAATAGCTCGATTAATCGAGCCTGACGGTACCGGGTGAGGAAGCACCGGCTAACTCCGTGCCAGCAGCCGCGGTAATACGGGGGGTGCGAGCGTTGTCCGGAATCACTGGGCGTAAAGGGCGCGTAGGTGGTTTGATAAGGGTGTGGTGAAAGCCCGGGGCTCAACCCCGGGTCTGCCGTGCCGACTGTCAAACTCGAGGGCTGTAGAGGCAGACGGAATTCCGGGTGTAGCGGTGGAATGCGTAGAGATCCGGAGGAAGACCGGTGGCGAAGGCGGTCTGCTGGGCAGTTTCTGACACTGATGCGCGACAGCGTGGGGAGCAAACAGG

>OTU643ATTAGGAATCTTGCGCAATGGGGGCAACCCTGACGCAGCGACACAGCGTGGAGGAAGAAGGTCTTCGGATTGTAAACTCCTTTTATATGGGACGAATAGATGACGGTACCATATGAATAAGCACCTGCTAACTACGTGCCAGAAGCATCGGTAATGCGTAGGGTGCAAACGTTATCCGGATTTATTGGGCGTATAGAGATGCGTAGGCGTTTTTGTAAGTCCTTAGTTAAAGACCACGGCTCAATCGTGGGAAAGCTTCGGATACTGCAAAAATTGAGACAGGAAGGGGCGAACAGAATTCCCAGTGGAGCGGTGAAATGCGTTGATATTGGGAGGAATATCAGTGGCGAAGGCGGTTCGCTGGTCCTTGTCTGACGCTGAGGCTCGAAAGCGTGGGTAGCGAAACGG

>OTU644GTGGGGAATATTGGACAATGGGGGCAACCCTGATCCAGCGACGCCGCGTGTGTGAAGAAGGCCTGCGGGTTGTAAAGCACTTTTAGTGGGGATGAAAAGCCTTGTTCTAACAAAGCAGGGTCTTGACCTAACCCAAAGAAAAAGCACCGGCTAACTCTGTGCCAGCAGCCGCGGTAATACAGAGGGGGCAAGCGTTATCCGGAATTATTGGGTGTAAAGGGTGCGTAGACGGGTGTTTAAGTTGGTTGTGAAATCCCTCGGCTCAACTGAGGAACTGCAACCAAAACTGGATATCTTGAGTGTCGGAGAGGAAAGTGGAATTCCTAGTGTAGCGGTGAAATGCGTAGATATCGAGAGGAACACCTGCGGCGAAGGCGGGTTGCTGGGCTGACACTGACGCTGAGGCGCGAAAGCTAGGGGAGCGAACGGG

>OTU645GTGGGGAATATTGCACAATGGGGGAAACCCTGATGCAGCGACGCCGCGTGAGTGAAGAAGTATCTCGGTATGTAAACTTCTATCAGCAGGGAAGACAATGACGGTACCTGACTAAGAAGCCCCGGCTAACTACGTGCCAGCAGCCGCGGTAATACGTAGGGGGCAAGCGTTATCCGGATTTACTGGGTGTAAAGGGAGCGTAGACGGTAATGCAAGTCTGGAGTGAAAGGCAGGGGCCCAACCCCTGGACTGCTTTGGAAACTGTGTAACTGGAGTGCAGGAGAGGTAAGTGGAATTCCTAGTGTAGCGGTGAAATGCGTAGATATTAGGAGGAACACCAGTGGCGAAGGCGGCTTACTGGACTGTAACTGACGTTGAGGCTCGAAAGCGTGGGGAGCAAACAGG

>OTU646GTAGGGAATCTTCCGCAATGGACGAAAGTCTGACGGAGCAACGCCGCGTGAGTGATGAAGGTTTTCGGATCGTAAAGCTCTGTTGTTAGGGAAGAACAAGTACCTGTTGAATAAGCAGGTACCTTGACGGTACCTAACCAGAAAGCCACGGCTAACTACGTGCCAGCAGCCGCGGTAATACGTAGGTGGCAAGCGTTGTCCGGAATTATTGGGCGTAAAGCGCGCGCAGGCGGTTCCTTAAGTCTGATGTGAAAGCCCACGGCTCAACCGTGGAGGGTCATTGGAAACTGGGGAACTTGAGTGCAGAAGAGGAGAGCGGAATTCCACGTGTAGCGGTGAAATGCGTAGAGATGTGGAGGAACACCAGTGGCGAAGGCGGCTCTCTGGTCTGTAACTGACGCTGAGGCGCGAAAGCGTGGGGAGCGAACAGG

>OTU647GTCGAGAATTTTTCACAATGGGCGCAAGCCTGATGGAGCGACGCCGCGTGGGGGATGAAGGGTTTCGATCCGTAAACCCCTGTCATTTGCGAACAATGGTTATTCAATAACACTGGATAGCTTGATAGTAGCGGAAGAGGAAGGGACGGCTAACTCTGTGCCAGCAGCCGCGGTAATACAGAGGTCCCAAGCGTTGTTCGGATTCACTGGGCGTAAAGGGTGCGTAGGTGGCCGGGAAAGTTTGATGTGAAAGCTCGGAGCTTAACTCCGAAGGGTCATTGAATACTATTCGGCTTGAGGGTCGGAGGGGAGACTGGAATTCTCGGTGTAGCAGTGAAATGCGTAGAGATCGAGAGGAACACCAGTGGCGAAGGCGAGTCTCTGGACGACACCTGACACTGAGGCACGAAAGCTAGGGGAGCAAACAGG

>OTU648GTGGGGAATTTTGGACAATGGGGGCAACCCTGATCCAGCCATGCCGCGTGTGTGAAGAAGGTCTTCGGATTGTAAAGCACTTTAAGTTGGGAGGAAGGGCAGTAAGTTAATACCTTGCTGTTTTGACGTTACCAACAGAATAAGCACCGGCTAACTTCGTGCCAGCAGCCGCGGTAATACGAAGGGTGCAAGCGTTAATCGGAATTACTGGGCGTAAAGCGCGCGTAGGTGGTTCAGCAAGTTGGATGTGAAAGCCCCGGGCTCAACCTGGGAACTGCATCCAAAACTACTGGGCTAGAGTATGGTAGAGGGTGGTGGAATTTCCTGTGTAGCGGTGAAATGCGTAGATATAGGAAGGAACACCAGTGGCGAAGGCGACCACCTGGACTAATACTGACACTGAGGTGCGAAAGCGTGGGGAGCAAACAGG

>OTU649GCGAGGAATCTTGGGCAATGGGCGAAAGCCTGACCCAGCGACGCCGCGTGGAGGATGAAGGCTTTAGGGTTGTAAACTCCTTTTGCCGGGGAAGAAGATCTGACGGTACCCGGCGAATAAGCCACGGCTAACTCTGTGCCAGCAGCCGCGGTAATACAGAGGGTGCAAGCGTTAATCGGAATTACTGGGCGTAAAGCGTGCGTAGACGGTTACGTAAGTCGGGTGTGAAAGCCCCGGGCTCAACCCGGGAATTGCATTCGAGACTGCGTAGCTAGGGTGCGGAAGAGGGAAGCGGAATTTCCGGTGTAGCGGTGAAATGCGTAGATATCGGAAGGAACATCAGTGGCGAAAGCGGCTTCCTGGTCCAGCACCGACGTTCAGGCACGAAAGCGTGGGGAGCAAACAGG

>OTU650GTGGGGAATATTGCGCAATGGGCGAAAGCCTGACGCAGCAACGCCGCGTGCGGGATGACGGCCTTCGGGTTGTAAACCGCTTTCAGCAGGGACGAAGTGAAAATGACGGTACCTGCAGAAGAAGCACCGGCTAACTACGTGCCAGCAGCCGCGGTGATACGTAGGGTGCGAGCGTTGTCCGGAATTATTGGGCGTAAAGAGCTTGTAGGCGGTCTGTTGCGTCGGAAGTGAAAACTCAGGGCTTAACCCTGAGCCTGCTTTCGATACGGGCAGACTTGAGGGATGTAGGGGAGAACGGAATTCCTGGTGGAGCGGTGGAATGCGCAGATATCAGGAGGAACACCAGTGGCGAAGGCGGTTCTCTGGACATTTCCTGACGCTGAGAAGCGAAAGCGTGGGGAGCAAACAGG

>OTU651GTGGGGAATTTTGGACAATGGGCGCAAGCCTGATCCAGCCATGCCGCGTGAGTGAAGAAGGCCTTCGGGTTGTAAAGCTCTTTCGGCCGGAACGAAATCGTCCGGGCTAATACCCCGGATGGATGACGGTACCGGAAGAAGAAGCACCGGCTAACTACGTGCCAGCAGCCGCGGTAATACGTAGGGTGCAGGCGTTAATCGGAATTACTGGGCGTAAAGCGTGCGCAGGCGGCTTCTCAAGTCAGATGTGAAATCCCCGGGCTTAACCTGGGAACTGCGTTTGAAACTGGGAGGCTAGAGTGCGGCAGAGGGGGGTGGAATTCCACGTGTAGCAGTGAAATGCGTAGATATGTGGAGGAACACCGATGGCGAAGGCAGCCCCCTGGGCCTGCACTGACGCTCATGCACGAAAGCGTGGGGAGCAAACAGG

>OTU652GTTAGGAATATTCGTCAATGGGGGAAACCCTGAACGAGCAATGCCGCGTGAACGATGACGGCCCTATGGGTTGTAAAGTTCTGTTGCGTGGGACGAACGATTAGGATAGGAAATGATCTTAGTGTGACGGTACCACGCCAGAAAGCTCCGGCTAACTACGTGCCAGCAGCCGCGGTAATACGTAGGGAGCGAGCGTTATCCGGATTTATTGGGCGTAAAGGGTGCGTAGGCGGTTTGTTAAGTATGAAATTAAAGCCTGGAGCTTAACTCCAGTTCGTTTCATAAACTGGCAGACTAGAGTATGGTAGAGGTAAACGGAATTTCTAGTGTAGCGGTAAAATGCGTAGATATTAGAAAGAACACCAGTGGCGTAGGCGGTTTACTGGGCCAAAACTGACGCTGAGGCACGAAAGCGTGGGGAGCAAATAGG

>OTU654GTGAGGAATATTGGTCAATGGACGAGAGTCTGAACCAGCCAAGTAGCGTGAAGGATGACTGCCCTATGGGTTGTAAACTTCTTTTATACGGGAATAAAGTGAGGCACGTGTGCCTTTTTGTATGTACCGTATGAATAAGGATCGGCTAACTCCGTGCCAGCAGCCGCGGTAATACGGAGGATCCGAGCGTTATCCGGATTTATTGGGTTTAAAGGGAGCGTAGGCGGACGCTTAAGTCAGTTGTGAAAGTTTGCGGCTCAACCGTAAAATTGCAGTTGATACTGGGTGTCTTGAGTACAGTAGAGGCAGGCGGAATTCGTGGTGTAGCGGTGAAATGCTTAGATATCACGAAGAACTCCGATTGCGAAGGCAGCTTGCTGGACTGTAACTGACGCTGATGCACGAAAGCGTGGGGATCAAACAGG

>OTU656GTGGGGAATTTTCCGCAATGGGCGAAAGCCTGACGGAGCAATGCCGCGTGGAGGTAAAAGGCCTACGGGTCATGAACTTCTTTTCCTGGAGAAGAAACAATGACGGTATCCGGGGAATAAGCATCGGCTAACTCTGTGCCAGCAGCCGCGGTAAGACAGAGGATGCAAGCGTTATCCGGAATGATTGGGCGTAAAGCGTCTGTAGGTGGCTTTTTAAGTTCGCCGTCAAATCCCAGGGCTCAACCCTGGACAGGTGGTGAAAACTACTAAGCTAGAGTACGGTAGGAGCAGAGGGAATTTCCGGAGGAGCGATGAAATGCGTAGAGATCGGAAGGAACACCAACGGCGAAAGCACTCTGCTGGGCCGACACTGACACTGAGAGACGAAAGCTAGGGGAGCGAATGGG

>OTU657GTAGGGAATCTTCGGCAATGGGCGAAAGCCTGACCGAGCAACGCCGCGTGAATGATGAAGGCCTTCGGGTTGTAAAATTCTGTTATAAGGGAAGAACGACTTTAGTAGGAAATGGCTAGAGTGTGACGGTACCTTATGAGAAAGCCACGGCTAACTACGTGCCAGCAGCCGCGGTAATACGTAGGTGGCGAGCGTTATCCGGAATTATTGGGCGTAAAGAGCGCGCAGGTGGTTGATTAAGTCTGATGTGAAAGCCCACGGCTTAACCGTGGAGGGTCATTGGAAACTGGTCGACTTGAGTGCAGAAGAGGGAAGTGGAATTCCATGTGTAGCGGTGAAATGCGTAGAGATATGGAGGAACACCAGTGGCGAAGGCGGCTTCCTGGTCTGTAACTGACACTGAGGCGCGAAAGCGTGGGGAGCAAACAGG

>OTU658GTAGGGAATATTGGTTAATGTGCGAAAGCGCGAACCAGCAACGCCGCGTGTGCGATGAAGGCCTTCGGGTCGTAAAGCACTTTTTGCAGGGAAGAGGAAGGACGGTACCTGCAGAATAAGTCTCGGCTAACTACGTGCCAGCAGCCGCGGTAAAACGTAGGAGACGAGCGTTATCCGGATTTACTGGGCGTAAAGCGCGTGCAGGCGGCATGGCAAGTTGGATGTGAAAGCTCCCGGCTTAACTGGGAGAGGTCGTTCAATACTGCCAGGCTTGAGAGTGGTAGAGGGAAGCGGAATTCCGGGTGTAGTGGTGAAATGCGTAGATATCCGGAGGAACACCAGTGGCGAAAGCGGCTTCCTGGCCCATTTCTGACGCTCAGACGCGAAAGCTAGGGTAGCAAACGGG

>OTU659GTGGGGAATTTTGCGCAATGGGGGAAACCCTGACGCAGCAACGCCGCGTGAGTGATGAAGGCCTTCGGGTTGTAAAGCTCTTTTAGTGGGGAAGAAGCCCTCCGGGGTTAATACCTCCGGGGTCTGACGGTACCCACAGAAAAAGTTCCGGCTAACTACGTGCCAGCAGCCGCGGTAATACGTAGGGAGCAAGCGTTGTTCGGAATTATTGGGCGTAAAGAGCGCGTAGGCGGTTCGGTAAGTCTGACGTGAAATCCCGGAGCTTAACTCCGGAACTGCGTTGGATACTGCCGGGCTTGAGTGTGGGAGAGGAGAGTGGAATTCCCAGTGTAGAGGTGAAATTCGTAGATATTGGGAGGAACACCCGTGGCGAAGGCGGCTCTCTGGACCACAACTGACGCTGAGGCGCGAAAGCTAGGGGAGCAAACGGG

>OTU661GTAGGGAATATTGGGCAATGGCCGAGAGGCTGACCCAGCCATGCCGCGTGCAGGAAGAAGGCCTTCTGGGTTGTAAACTGCTTTTATCTGGGAAGAAAACGCCCCTGCGGGGGTAACTGACGGTACCAGATGAATAAGCACCGGCTAACTCCGTGCCAGCAGCCGCGGTAATACGGAGGGTGCAAGCGTTGTCCGGATTTATTGGGTTTAAAGGGTGCGTAGGCGGCCCTGTAAGTCAGTGGTGAAATCCCAGGGCTCAACCCTGGAACTGCCATTGATACTGCAGGGCTTGAGTTCGGTTAAGGCGGGCGGAACTGGTGGTGTAGCGGTGAAATGCATAGATACCACCAAGAACCCCGATTGCGTAGGCAGCTCGCTGAGCCGAAACTGACGCTGAGGCACGAAAGCGTGGGGAGCGAACAGG

>OTU662GTGGGGAATATTGGACAATGGGCGCAAGCCTGATCCAGCCACGCCGCGTGAGTGATGAAGGCCTTCGGGTCGTAAAGCTCTGTGGGGAGGGACGAACCGCTGCAGGTTCATAGCCTGCAGCATGACGGTACCTCCTTAGCAAGCACCGGCTAACTTCGTGCCAGCAGCCGCGGTAATACGAAGGGTGCAAACGTTGCTCGGAATTATTGGGCGTAAAGCGCACGTAGGCGGCATTGCAAGTCGGATGTGAAAGCCCTCGGCTTAACCAAGGAAGTGCATCCGAAACTGCAGTGCTTGAGTACTTAAGAGGATCGCGGAATTCCCGGTGTAGAGGTGAAATTCGTAGATATCGGGAGGAACACCAGTGGCGAAGGCGGCGATCTGGGAAGATACTGACGCTGAGGTGCGAAAGCGTGGGGAGCAAACAGG

>OTU663GTGGGGAATATTGCGCAATGGCCGAAAGGCTGACGCAGCGACGCCGCGTGTGGGAGGAAGCCCTTCGGGGTGTAAACCACTGTCAGGGGGGAAGAAACCAGGTCGGGCGAATAGGCCGATCTGTTGACGGTACCTCCAAAGGAAGCGCCGGCTAACTCCGTGCCAGCAGCCGCGGTAAGACGGAGGGCGCAAGCGTTGTTCGGAATTACTGGGCGTAAAGCGCGGGCAGGCGGCCCGTTAAGTCTGCGGTGAAATCCCGGGGCTCAACCCCGGATATGCCGCGGATACTGGCGGGCTAGAGACCGGTAGAGGCGAGTGGAATTCCTGGTGTAGCGGTGAAATGCGTAGAGATGTGGAGGAATACCAGTGGCGAAGGCGGCTCTCTGGTCTGTTACTGACGCTGAGGTGCGAAAGCGTGGGGAGCGAACAGG

>OTU664GTGAGGAATATTGCACAATGGGCGCAAGCCTGATGCAGCGACGCCGCGTGGGTGAAGAAGGATTTCGGTCTGTAAAGCCCTTTTCTGTGTGACGAGAGTGGACGGTAGCACAGGAATAAGTCTCGGCTAACTACGTGCCAGCAGCCGCGGTAAAACGTAGGAGGCAAGCGTTATCCGGAGTTACTGGGCGTAAAGGGCGTGCAGGTGGTTCAATAAGTTGGTTATGAAAGCTCCTGGCTAAACTGGGAGAGGTTGACCAAGACTGTTGAACTTGAGTGGATGAGAGGTAAACGGAATTCCGCGTGTAGTGGTGAAATGCGTAGAGATGCGGAGGAACACCAGTGGCGAAGGCGACACGCTGGGCCTGGCCTGACGCTGAGAGGCGAAAGCATGGGGAGCGAACGGG

>OTU665GTCGGGAATATTGCTCAATGGGCGAAAGCCTGAAGCAGCAACGCCGCGTGCATGATGAAGGTCTTCGGATCGTAAAGTGCTTTTCTGGGAGATGAGAAAGGACAGTATCCCAGGAATAAGTCTCGGCTAACTACGTGCCAGCAGCCGCGGTAAAACGTAGGAGGCAAGCGTTATCCGGATTCACTGGGCGTAAAGCGCGTGCAGGTGGCGCGGTAAGTTGGATGTGAAATCTCCCGGCTCAACTGGGAGAGGTCATTCAATACTACCGTGCTTGAGAGCAGAAGAGGAAGATGGAATTCCCGGTGTAGTGGTGAAATGCGTAGATATCGGGAGGAACACCAGTGGCGAAGGCGGGTTGCTGGGCTGACACTGACGCTGAGGCGCGAAAGCTAGGGGAGCGAACGGG

>OTU666GTGGGGAATATTGCACAATGGGGGAAACCCTGATGCAGCAACGCCGCGTGAGCGAAGAAGGCCTTTGGGTCGTAAAGCTCTGTCCTTAGGGAAGAAGGAAGTGACGGTACCTAAGGAGGAAGCCCCGGCTAACTACGTGCCAGCAGCCGCGGTAATACATAGGGGGCAAGCGTTATCCGGAATTATTGGGTGTAAAGGGTGCGTAGACGGGTGTTTAAGTTGGTTGTGAAATCCCTCGGCTCAACTGAGGAACTGCAACCAAAACTGGATATCTTGAGTGTCGGAGAGGAAAGTGGAATTCCTAGTGTAGCGGTGAAATGCGTAGATATTAGGAGGAACACCAGTGGCGAAGGCGGCTTACTGGACGATTACTGACGCTGAGGCTCGAAAGCGTGGGGAGCAAACAGG

>OTU668GTGGGGAATATTGCGCAATGGCCGAAAGGCTGACGCAGCGACGCCGCGTGCGGGATGACGGCCTTCGGGTTGTAAACCGCTGTCGGGAGGGACGAAGATCTGACGGTACCTCCAAAGGAAGCACCGGCTAACTCCGTGCCAGCAGCCGCGGTAATACGGAGGGTGCGAGCGTTGTCCGGAATCACTGGGCGTAAAGGGCGCGTAGGCGGCCCGGTAAGTAGGGGGTGAAATCCTGCGGCTCAACCGCAGAACCGCCTTCTAGACTGCCAGGCTCGAGCACAGTAGAGGCAGACGGAATTCCCGGTGTAGCGGTGGAATGCGTAGAGATCGGGAAGAACACCGGTGGCGAAGGCGGTCTGCTGGGCTGTTGCTGACGCTGAGGCGCGACAGCGTGGGGAGCAAACAGG

>OTU669GTGAGGAATATTGGTCAATGGCCGGGAGGCTGAACCAGCCAAGTCGCGTGAGGGAAGACGGCCCTACGGGTTGTAAACCTCTTTTGTCAGGGAGCAAGGTGCAGGTCGGGACCTGCTGTGAGAGTACCTGAAGAAAAAGCATCGGCTAACTCCGTGCCAGCAGCCGCGGTAATACGGAGGATGCGAGCGTTATCCGGATTTATTGGGTTTAAAGGGTGCGTAGGCGGGTTATCAAGTCAGCGGTAAAATCGTGGAGCTCAACTCCATCCAGCCGTTGAAACTGATGATCTTGAGTGGGCGAGAAGTATGCGGAATGCGTGGTGTAGCGGTGAAATGCATAGATATCACGCAGAACTCCGATTGCGAAGGCAGCATACCGGCGCCCGACTGACGCTGAAGCACGAAAGCGTGGGTATCGAACAGG

>OTU671GTGGGGAATTTTGCACAATGGGCGAAAGCCTGATGCAGCAACGCCGCGTGCGGGATGAAGGCCCTCGGGTTGTAAACCGCTTTCAGCAGGGACGAAACTGACGGTACCTGCAGAAGAAGCCCCGGCTAACTACGTGCCAGCAGCCGCGGTAATACGTAGGGGGCAAGCGTTATCCGGATTTATTGGGCGTAAAGCGCGCGTAGGCGGCTTGTTAAGTCAGATGTGAAAACCGGGGGCTCAACCCGCGGCCTGCATCTGAAACTGGCAGGCTTGAGTCTGGTAGAGGAAAGTGGAATTCCTGGTGTAGCGGTGAAATGCGCAGATATCAGGAGGAACACCAGTGGCGAAGGCGGCTTTCTGGGCCACGACTGACGCTGAGGCGCGAAAGCTAGGGGAGCGAACAGG

>OTU672GTGGGGAATATTGCGCAATGGGGGAAACCCTGACGCAGCAACGCCGCGTGAGTGATGAAGGCCTTAGGGTTGTAAAGCTCTGTTATCTGGGACGATAATGACGGTACCAGAGGAGGAAGCCACGGCTAACTACGTGCCAGCAGCCGCGGTAATACGTAGGTGGCGAGCGTTGTCCGGATTTACTGGGCGTAAAGAGTGCGTAGGCGGATGTTTAAGTGAGATGTGAAATACCCGAGCTCAACTTGGGTGCTGCATTTCAAACTGGGCATCTAGAGTGCAGGAGAGGAAAGCGGAATTCCTAGTGTAGCGGTGAAATGCGTAGAGATTAGGAAGAACACCAGTGGCGAAGGCGGCTTTCTGGACTGTAACTGACGCTGAGGCACGAAAGCGTGGGGAGCAAACAGG

>OTU673GTGGGGAATTTTTCGCAATGGGGGAAACCCTGACGAAGCAACGCCGCGTGGAGGATGAAGGCCTTCGGGTCGTAAACTCCTGTCGACCGGGACGAACATGGTCCGACCCAACACGTCGGGCTATCGACTGTACCGGTGGAGGAAGCCACGGCTAACTCTGTGCCAGCAGCCGCGGTAATACAGAGGTGGCAAGCGTTGTTCGGAATTACTGGGCGTAAAGGGCGCGTAGGCGGTGCTCTAAGTCCCGTGTGAAAGCCCCCGGCTCAACTGGGGAACTGCACGGGAAACTGGAGTGCTTGAGTTCGGGAGAGGGAAGCGGAATTCCGGGTGTAGCGGTGAAATGCGTAGATATCCGGAGGAACACCGGTGGCGAAGGCGGCTTCCTGGACCGACACTGACGCTGAGGCGCGAAAGCTAGGGGAGCAAACGGG

>OTU674GTGGGGAATATTGCACAATGGGCGAAAGCCTGATGCAGCCATGCCGCGTGTGTGAAGAAGGCCTTAGGGTTGTAAAGCACTTTCAGTGGGGAGGAAAGGTTAGTAGTTAATACCTGCTAGCTGTGACGTTACCCACAGAAGAAGGACCGGCTAATTCCGTGCCAGCAGCCGCGGTAATACGGAAGGTCCGAGCGTTAATCGGAATTACTGGGCGTAAAGCGCACGTAGGCGGCTAGGTCAGTTGGGTGTGAAAGCCCCGGGCTCAACCTGGGAACTGCATCCAATACTGCCTGGCTAGAGTACGAGAGAGGGGGGTAGAATTTCTAGTGTAGCGGTGAAATGCGTAGATATTAGAAGGAATACCAGTGGCGAAGGCGGCCCCCTGGCTCGATACTGACGCTGAGGTGCGAAAGCGTGGGGAGCAAACAGG

>OTU675GTGGGGAATCTTCCGCAATGGGCGAAAGCCTGACGGAGCAATGCCGCGTGAGTGAAGAAGGCCTTCGGGTTGTAAAGCTCTATCGTAATCGAAGAGAGCTTGTGATAGTAACTGATCACGAGAGGACGGTAGATTACAAGGAAGCCCCGGCTAACTATGTGCCAGCAGCCGCGGTAAAACATAGGGGGCAAGCGTTGTCCGGAATCACTGGGCGTAAAGGGCGCGTAGGTGGTTTGTTAAGTCAGATGTGAAATGTAGGGGCTCAACCCCTAACGTGCATCTGATACTGGCAGACTTGAGTGCGGAAGAGGCAAGTGGAATTCCTAGTGTAGCGGTGAAATGCGTAGATATTAGGAGGAACACCAGTGGCGAAGGCGACTTGCTGGGCCGTAACTGACGCTGAGGCGCGAAAGCGTGGGGAGCAAACAGG

>OTU676GTGGGGAATATTGCACAATGGGGGAAACCCTGATGCAGCGACGCCGCGTGAGCGAAGAAGTATTTCGGTATGTAAAGCTCTATCAGCAGGGAAGATAATGACGGTACCTGACTAAGAAGCCCCGGCTAACTACGTGCCAGCAGCCGCGGTAATACGTAGGGGGCAAGCGTTATCCGGATTTACTGGGTGTAAAGGGAGCGTAGACGGCGAAGCAAGTCTGATGTGAAATACCGGGGCTCAACCCCGGGACTGCATTGGAAACTGTTTTGCTTGAGTGCCGGAGAGGTAAGCGGAATTCCTAGTGTAGCGGTGAAATGCGTAGATATTAGGAGGAACACCGATGGCGAAGGCAGCCCCCTGGGACATGACTGACGCTCATGCACGAAAGCGTGGGGAGCAAACAGG

>OTU677GTGGGGAATATTGCGCAATGGGCGAAAGCCTGACGCAGCCACGCCGCGTGAGCGATGAAGGTCTTCGGATCGTAAAGCTCTGTGGGGGGAGAAGAAGAAAGCCGATGAAGAGTCGGCCCTGACGGTATCCCCTTAGCAAGCACCGGCTAACTCTGTGCCAGCAGCCGCGGTAAGACAGAGGGTGCGAACGTTGCTCGGAATTACTGGGCGTAAAGCGCGTGTAGGCGGGTGATCAAGTCAGATGTGAAATCCCTGGGCTCAACCCAGGAACTGCATTTGAAACTGGTCGCCTGGAGTATTGGAGAGGGAAGCGGAATTCCTGGTGTAGAGGTGAAATTCGTAGATATCAGGAGGAACACCGGTGGCGAAGGCGGCTTCCTGGACAATTACTGACGCTGAGACGCGAAAGCGTGGGGAGCAAACAGG

>OTU678GTTAGGAATTTTGGACAATGGGCGCAAGCCTGATCCAGCCACGCCGCGTGAGTGATGAAGGCCTTCGGGTCGTAAAGCTCTGTGGGGAGGGACGAACCGCCGGCGGTTAATACCCGTCGGCATGACGGTACCTCCTTAGCAAGCACCGGCTAACTCTGTGCCAGCAGCCGCGGTAATACAGAGGGTGCTAGCGTTGTTCGGAATTATTGGGCGTAAAGCGCGTGTAGGCGGCTGGCCAAGTCGGATGTGAAAGCCCTCGGCTTAACCGAGGAAGTGCGTTCGAAACTAGCTAGCTTGAGTACCGGAGAGGGTGGTGGAATTCCCGGTGTAGAGGTGAAATTCGTAGATATCGAGAGGAACACCGGTGGCGAAGGCGGCCAACTGGACGAGTACTGACGCTGAGACACGAAAGCGTGGGGAGCAAACAGG

>OTU679GTGGGGAATATTCCGCAATGGACGAAAGTCTGACGGAGCAACGCCGCGTGAGTGATGAAGGTTTTCGGACTGTAAAGCTCTGTTGTTCAGGACGAAAGTGCCTGTTGCGAACAGTTACAGGTATTGACGGTACTGAACGAGGAAGCCACGGCTAACTACGTGCCAGCAGCCGCGGTAATACGTAGGTGGCAAGCGTTGTCCGGAATTATTGGGCGTAAAGAGCATGTAGGCGGGAATCTAAGTCTGTTGTGAAAATGCGGGGCTCAACCCCGTATGGCGATGGAAACTGGATTTCTTGAGTGCAGGAGAGGAAAGGGGAATTCCCAGTGTAGCGGTGAAATGCGTAGATATTGGGAGGAACACCAGTGGCGAAGGCGCCTTTCTGGACTGTGTCTGACGCTGAGATGCGAAAGCCAGGGTAGCGAACGGG

>OTU680GTAGGGAATATTGCTCAATGGGGGAAACCCTGAAGCAGCAACGCCGCGTGGAGGATGACACTTTTCGGAGCGTAAACTCCTTTTGTTAGGGAAGAACAATGACGGTACCTAACGAATAAGCACCGGCTAACTCCGTGCCAGCAGCCGCGGTAATACGGAGGGTGCAAGCGTTACTCGGAATCACTGGGCGTAAAGGACGCGTAGGCGGATTATCAAGTCTCTTGTGAAATCCTATGGCTTAACCATAGAACTGCTTGGGAAACTGATAATCTAGAGTGAGGGAGAGGCAGATGGAATTGGTGGTGTAGGGGTAAAATCCGTAGAGATCACCAGGAATACCCATTGCGAAGGCGATCTGCTGGAACTCAACTGACGCTAATGCGTGAAAGCGTGGGGAGCAAACAGG

>OTU682GTGGGGAATTGTTCGCAATGGGCGCAAGCCTGACGACGCAACGCCGCGTGGAGGATGAAGGTCTTCGGATTGTAAACTCCTGTTGATCGGGAAGAATGGACTCCGGGTTAATACTCCGGGGTAATGACGGTACCGATTGAGGAAGCCACGGCTAACTCTGTGCCAGCAGCCGCGGTAATACAGAGGTGGCAAGCGTTGTTCGGAATTACTGGGCGTAAAGGGCGCGTAGGCGGCCTTCTAAGTCAGACGTGAAATCCCCAGGCTTAACTTGGGAACTGCAAGGGAAACAGCAGGGCTTGAGTTCGGGAGAGGGAAGTGGAATTCCCGGTGTAGTGGTGAAATGCGTAGATATCGGGAGGAACACCAGTGGCGAAGGCGACTTCCTGGCCTGTTCTTGACGCTGAGGCGCGAAAGCTAGGGTAGCAAACGGG

>OTU683GTGGGGAATATTGCACAATGGGGGAAACCCTGATGCAGCAACGCCGCGTGAGTGAAGAAGTCATTCGTGATGTAAAGCTCTATCAGCAGGGAAGAAAATGACGGTACCTGACTAAGAAGCCCCGGCTAACTACGTGCCAGCAGCCGCGGTAATACGTAGGGGGCAAGCGTTATCCGGATTTACTGGGTGTAAAGGGAGCGTAGACGGCGACGCAAGTCTGAAGTGAAATGCCAGTGCTCAACACTGGAACTGCTTTGGAAACTGTGTGGCTAGAGTGCAGGAGAGGTAAGTGGAATTCCTAGTGTAGCGGTGAAATGCGTAGATATTAGGAGGAACACCAGTGGCGAAGGCGGCTTACTGGACTGTAACTGACGTTGAGGCTCGAAGGCGTGGGGAGCAAACAGG

>OTU684GTGGGGAATTTTGGACAATGGGGGCAACCCTGATCCAGCCATGCCGCGTGCAGGATGAAGGCCTTCGGGTTGTAAACTGCTTTTGTCAGGGACGAAAAGGACCGTGTTAATACCATGGTCTGCTGACGGTACCTGAAGAATAAGCACCGGCTAACTACGTGCCAGCAGCCGCGGTAATACGTAGGGTGCAAGCGTTAATCGGAATTACTGGGCGTAAAGCGTGCGCAGGCGGTTCTGTAAGACAGATGTGAAATCCCCGGGCTCAACCTGGGAATTGCATTTGTGACTGCAGGACTAGAGTTCATCAGAGGGGGGTGGAATTCCAAGTGTAGCAGTGAAATGCGTAGATATTTGGAAGAACACCAATGGCGAAGGCAGCCCCCTGGGATGCGACTGACGCTCATGCACGAAAGCGTGGGGAGCAAACAGG

>OTU685GTCGAGAATTTTTCTCAATGGGGGAAACCCTGAAGGAGCGACGCCGCGTGAAGGATGAAGGTCTTCGGATCGTAAACTTCTGTCATTGGTGAACAAGGCCCACGCAGTAACTGGTGTTGGGATTGATAGTAGCCGAAGAGGAAGAGACGGCTAACTCTGTGCCAGCAGCCGCGGTAATACAGAGGTCTCAAGCGTTGTTCGGATTCATTGGGCGTAAAGGGTGCGTAGGTGGCGAGGCAAGTCTGGTGTGAAATCCCGGGGCTCAACCCCGGAACTGCACTGGATACTGCCCTGCTTGAGTACTGGAGAGGAGATTGGAATTTACGGTGTAGCAGTGAAATGCGTAGATATCGTAAGGAAGACCAGTGGCGAAGGCGAATCTCTGGACAGTTACTGACACTGAGGCACGAAGGCCAGGGGAGCAAACGGG

>OTU686GTGGGGAATCTTGCGCAATGGGGGAAACCCTGACGCAGCAACGCCGCGTGGGTGATGAAGGCCTTCGGGTCGTAAAGCCCTGTCAGGTGGGAAGAAACTTGTGGATGCTAATATCATCCACACTTGACGGTACCACCGGAGGAAGCACCGGCTAACTACGTGCCAGCAGCCGCGGTAATACGTAGGGTGCGAGCGTTAATCGGAATTACTGGGCGTAAAGCGTGCGCAGGCGGTTTTGTAAGACAGCTGTGAAATCCCCGGGCTCAACCTGGGAACTGCGGTTGTGACTGCAAGACTTGAGTACGGCAGAGGGGGGTGGAATTCCTGGTGTAGCAGTGAAATGCGTAGATATCAGGAGGAACACCGATGGCGAAGGCAGCCCCCTGGGCCTGTACTGACGCTCATGCACGAAAGCGTGGGGAGCAAACAGG

>OTU687GTGGGGAATATTGCACAATGGGCGCAAGCCTGATGCAGCCATGCCGCGTGTATGAAGAAGGCCCTAGGGTTGTAAAGTACTTTCAGTCGGGAGGAAGGCGTTGATGCTAATATCATCAACGATTGACGTTACCGACAGAAGAAGCACCGGCTAACTCCGTGCCAGCAGCCGCGGTAATACGGAGGGTGCAAGCGTTAATCGGAATTACTGGGCGTAAAGCGCACGCAGGCGGTTGATTAAGTTAGATGTGAAATCCCCGGGCTTAACCTGGGAATGGCATCTAAGACTGGTCAGCTAGAGTCTTGTAGAGGGGGGTAGAATTCCATGTGTAGCGGTGAAATGCGTAGAGATGTGGAGGAATACCGGTGGCGAAGGCGGCCCCCTGGACAAAGACTGACGCTCAGGTGCGAAAGCGTGGGGAGCAAACAGG

>OTU688GTGGGGAATCTTCCACAATGGGCGAAAGCCTGATGGAGCAACGCCGCGTGAGTGATGAAGGCCTTCGGGTTGTAAAACTCTGTCTTGCGAGAATAAAGCTGCAAGGTGAACAATCTTGCAGTTTGACTGTATCGCAGGAGGAAGACACGGCTAACTCTGTGCCAGCAGCCGCGGTAATACAGAGGTGTCAAGCGTTGTCCGGAATTACTGGGCGTAAAGGGCGTGTAGGTGGGCGTGTAAGTCGGATGTGAAATCGCTGGGCTTAACCCAGCAACCGCATCCGAAACTGCTCGTCTTGAGTGCAGAAGAGGAGAGTGGAATTCCCAGTGTAGCGGTGAAATGCGTAGATATTGGGAGGAACACCAGTGGCGAAGGCGGCTCTCTGGTCTGCAACTGACACTGAAACGCGAAAGCTAGGGGAGCAAACGGG

>OTU690GTGGGGAATATTGGACAATGGGGGCAACCCTGATCCAGCGATGCCGCGTGTGCGAAGAAGGCCTGCGGGTTGTAAAGCACTTTAGGTGGGGAGGAAAGGCGGCGAGTGAATAACTTGCTGAGTTGACGTTACCCACAGAATAAGCACCGGCTAACTCTGTGCCAGCAGCCGCGGTAATACAGAGGGTGCAAGCGTTAATCGGAATGACTGGGCGTAAAGGGTGCGTAGGCGGTTGATGAAGTCAGATGTGAAATACCTGGGCTTAACCTGGGAAGGCCATTTGAGACGCATCGGCTAGAGTAGAGTAGAGGGAAGTGGAATTTCCGGTGTAGCGGTGAAATGCGTAGATATCGGAAAGAACACCAGTGGCGAAGGCGGCTTCCTGGACTCATACTGACGCTGAGGCACGAAAGCGTGGGGAGCAAACAGG

>OTU691GTGGGGAATATTGGACAATGGGGGCAACCCTGATCCAGCCATGCCGCGTGAGTGATGAAGGCCTTAGGGTTGTAAAGCTCTTTCACCGGTGAAGATAATGACGGTAACCGGAGAAGAAGCCCCGGCTAACTTCGTGCCAGCAGCCGCGGTAATACGAAGGGGGCTAGCGTTGTTCGGAATTACTGGGCGTAAAGCGCACGTAGGCGGATTGGTCAGTTAGAGGTGAAATCCCGGAGCTCAACTCCGGAACTGCCTTTAATACTGCCAATCTAGAGACCGAGAGAGGTAAGTGGAACTCCTAGTGTAGAGGTGGAATTCGTAGATATTAGGAAGAACACCAGTGGCGAAGGCGGCTTACTGGCTCGGAACTGACGCTGAGGTGCGAAAGCGTGGGGAGCAAACAGG

>OTU692GTGAGGAATATTGGTCAATGGAGGCAACTCTGAACCAGCCAAGTAGCGTGCAGGATGACGGCCCTATGGGTTGTAAACTGCTTTTATGCGGGGATAAACTGAGCCACGTGTGGCTTATTGCAGGTACCGCATGAATAAGGACCGGCTAATTCCGTGCCAGCAGCCGCGGTAATACGGAAGGTCCGGGCGTTATCCGGATTTATTGGGTTTAAAGGGAGCGCAGGCCGCCCCTTAAGCGTGTTGTGAAATGCCGCGGCTCAACCGTGGCACTGCAGCGCGAACTGGGGGGCTTGAGTGCGCCGGAGGTAGGCGGAATTCGTGGTGTAGCGGTGAAATGCTTAGATATCACGAAGAACTCCGATTGCGAAGGCAGCCTACCGTAGCGTTACTGACGCTGAGGCTCGAAGGTGCGGGTATCGAACAGG

>OTU693GTGAGGAATATTGGTCAATGGTCGGGAGACTGAACCAGCCAAGCCGCGTGAGGGAAGAAGGTACAGCGTATCGTAAACCTCTTTTGTCAGGGAACAAAGGCGGGGACGTGTCCCCGGATGAGTGTACCTGAAGAAAAAGCATCGGCTAACTCCGTGCCAGCAGCCGCGGTAATACGGAGGATGCGAGCGTTATCCGGATTTATTGGGTTTAAAGGGTGCGTAGGCGGACGCTTAAGTCAGCGGTAAAATTGCGGGGCTCAACCCCGACGAGCCGTTGAAACTGGCTTGCTAGAGTGGGCGAGAAGTATGCGGAATGCGTGGTGTAGCGGTGAAATGCATAGATATCACGCAGAACTCCGATTGCGAAGGCAGCATACCGGCGCTCAACTGACGCTCATGCACGAAAGCGTGGGTATCGAACAGG

>OTU694GTGAGGAATATTGGACAATGGATGGAAATCTGATCCAGCCATGCCGCGTGCAGGAAGACGGCCCTATGGGTTGTAAACTGCTTTTATATGGGAGCAATAAGGTCTACGCGTAGACCGATGAGAGTACCATATGAATAAGCATCGGCTAACTCCGTGCCAGCAGCCGCGGTAATACGGAGGATGCGAGCGTTATCCGGATTTATTGGGTTTAAAGGGTGCGTAGGCGGCCTTATAAGTCAGCGGTGAAATTTTGCAGCTTAACTGTAACAGTGCCGTTGATACTGTTTGGCTAGGATTTGGTTGTAGTGTGCGGAATGTGTGGTGTAGCGGTGAAATGCTTAGATATCACACAGAACACCGATTGCGAAGGCAGCACACTAAGTCATTATTGACGCTGAGGCACGAAAGTGTGGGGATCAAACAGG

>OTU695GTGAGGAATCTTCCGCAATGGGCGAAAGCCTGACGGAGCGACGCCGCGTGTGGGATGAAGACCTTCGGGTTGTAAACCACTGTCGGAGGGGAAGACTCGGACGGTACCCTCCAAGAAAGCCCCGGCTAACTACGTGCCAGCAGCCGCGGTAATACGTAGGGGGCGAGCGTTACCCGGATTTACTGGGCTTAAAGCGCGTGTAGGCGGCTGGCCAAGTGTGCTGTGAAATCCCTCGGCTCAACCGAGGAACTGCAGTGCAAACTGGTCTGGCTAGAGTGCAGGAGAGGGTAGTGGAATTCCCGGTGTAGCGGTGAAATGCGTAGATATCGGGAGGAACACCAGTGGCGAAGGCGGCTACCTGGCCTGTAACTGACGCTGAGACGCGAAAGTGTGGGGAGCGAACCGG

>OTU696GTGGGGAATCTTGCGCAATGGGCGAAAGCCTGACGCAGCGACGCCGCGTGAGCGATGAAGGCCTTCGGGTTGTAAAGCTCTGTGGGGAGGGAAGAATAAGGGTTGGCTAATATCCAACTCGATGACGGTACCTCCTTAGCAAGCACCGGCTAACTCTGTGCCAGCAGCCGCGGTAATACAGAGGGTGCAAACGTTGTTCGGAATTACTGGGCGTAAAGCGCGTGCAGGCGGCCTTGCAAGTCGGACGTGAAATCCCACGGCTCAACCGTGGAAGTGCATTCGAAACTGCGAGGCTGGAGTCCTGGAGAGGAAAGTGGAATACTCGGTGTAGAGGTGAAATTCGTAGATATCGAGTGGAACACCGGTGGCGAAGGCGGCTTTCTGGACAGGTACTGACGCTGAGACGCGAAAGCGTGGGGAGCAAACAGG

>OTU697GTGGGGAATATTGTGCAATGGGGGAAACCCTGACACAGCGATGCCGCACGGGTGAAGAAGGGATTCGTTTTGTAAAGCCCTGTCCCCGGGGAAGAGAGTGACGGTACCCGGGTAAGAGCCCCAGCTAACTACGTGCCAGCAGCTGCGGTAACACGTGGGGGGCGAGCGTTGTCCGGAATTATTGGGCGTAAAGGGCACGCAGGCGGCCCCTTAGGCTGTTGATGAAAGTGCTGGGCCCAACCCAGTGATGTTCAACAGGACCGAGGGGCTCGGGTGCCAGAGAGGCAGGCAGAATTCCTGGTGTAGCGGTGAAATGCGCAGAGATCAGGAGGAATACCAGAGGCGAAGGCGGCCTGCTGGCTGGCAACCGACGCTCAGGTGCGAAAGCGTGGGGAGCGAACAGG

>OTU699GTGGGGAATATTGGACAATGGGCGCAAGCCTGATCCAGCAATGCCGCGTGGGTGAAGAAGGTCTTCGGATTGTAAAGCCCTTTCGGCGGGGACGATGATGACGGTACCCGCAGAAGAAGCCCCGGCTAACTTCGTGCCAGCAGCCGCGGTAATACGAAGGGGGCTAGCGTTGCTCGGAATGACTGGGCGTAAAGGGCGCGTAGGCGGATCGGATAGTCAGGCGTGAAATTCCTGGGCTCAACCTGGGGGCTGCGCTTGATACGTCTGGTCTGGAGTGGGGAAGAGGGTCGTGGAATTCCCAGTGTAGAGGTGAAATTCGTAGATATTGGGAAGAACACCGGTGGCGAAGGCGGCGACCTGGTCCTTGACTGACGCTGAGGCGCGAAAGCGTGGGGAGCAAACAGG

>OTU701GTGGGGAATATTGCACAATGGGCGCAAGCCTGATGCAGCAACGCCGCGTGAGGGATGACGGCCTTCGGGTTGTAAACCTCTTTTAGTTGGGAAGAAGCCTTCGGGTGACGGTACCTTCAGAAAAAGCGCCGGCTAACTACGTGCCAGCAGCCGCGGTAATACGTAGGGCGCAAGCGTTATCCGGAATTATTGGGCGTAAAGAGCTCGTAGGCGGTTTGTCGCGTCTGCTGTGAAAACTGGAGGCTCAACCTCCAGCCTGCAGTGGGTACGGGCAGACTAGAGTGCGGTAGGGGAGATTGGAATTCCTGGTGTAGCGGTGGAATGCGCAGATATCAGGAGGAACACCGATGGCGAAGGCAGATCTCTGGGCCGTAACTGACGCTGAGGAGCGAAAGAGTGGGGAGCAAACAGG

>OTU704GTGGGGAATCTTGCACAATGGGCGCAAGCCTGATGCAGCAACGCCGCGTGAGTGAAGAAGGTCTTCGGATTGTAAAGCTCTTTCGGCCGGGAAGAAGGGTCATATGGTTAATACCCATGTGATTTGACGGTACTGGAAGAAGAAGCACTGGCTAATTTCGTGCCAGCAGCTGCGGTAATACGAGAGGTGCAAGCGTTGTTCGGAGTGACTGGGCGTAAAGCGCACGTAGGCGGTCTTGTAAGTCGATTGTGAAATCCCCGGGCTTAACCTGGGAACAGCAGTCGAAACTGCAAGTCTTGAATGTCCGAGAGGTTAGTGGAATTCCTGGTGTAGGAGTGAAATCCGTAGATATCAGGAGGAACACCGGAGGCGAAGGCGGCTAACTGGTGGAACATTGACGCTGAGGTGCGAAAGCGTGGGTAGCAAACAGG

>OTU705GTGGGGGATATTGCACAATGGGGGGAACCCTGATGCAGCGACGCCGCGTGGGTGAAGAAGCGCCTCGGCGCGTAAAGCCCTGTCAGCAGGGAAGAAAATGACGGTACCTGACCAAGAAGCCCCGGCTAACTACGTGCCAGCAGCCGCGGTAATACGTAGGGGGCAAGCGTTATCCGGATTTACTGGGTGTAAAGGGGGCGCAGACGGCGAAGCAAGCCAGGAGTGAAAGCCCGGGGCCCAACCCCGGGACTGCTCTTGGAACTGCTTGGCTGGAGTGCAGGAGGGGCAGGCGGAATTCCTGGTGTAGCGGTGAAATGCGTAGATATCAGGAGGAACACCGGTGGCGAAGGCGGCCTGCTGGACTGCAACTGACGTTGAGGCCCGAAAGCGTGGGGAGCAAACAGG

>OTU706GTTAGGAATATTCGTCAATGGGGGAAACCCTGAACGAGCAATGCCGCGTGAGTGAGGAAGGTCTTCGGATTGTAAAGCTCTGTTGCCGGGGAAAAGAGGGGAAGAGAGGAAATGCTCTTTCCAGGATGGTACCCGGCCAGAAAGTCACGGCTAACTACGTGCCAGCAGCCGCGGTAATACGTAGGTGGCAAGCGTTATCCGGAATGATTGGGCGTAAAGGGTGAGCAGGCGGCCTGGACAAGTCATGAGTGAAAGGTGGAAGCTTAACTTTCACAGGCTGATGATACTGACAGGCTTGAGTACAGGAGAGGGCGGCGGAACTCCATGTGTAGCGGTAAAATGCGTAGATATATGGAAGAACACCAGTGGCGAAGGCGGCCGCCTGGACTGTAACTGACGCTGAGGCACGAAAGCGTGGGGAGCAAATAGG

>OTU707GTGAGGAATATTGGTCAATGGGCGGAAGCCTGAACCAGCCAAGTCGCGTGAGGGAATAAGGCCCTACGGGTCGTAAACCTCTTTTGCCGGGGAGCAAAGTGCCGCACGTGTGCGGTTTGGAGAGTACCCGGAGAAAAAGCATCGGCTAACTCCGTGCCAGCAGCCGCGGTAATACGGAGGGTGCAAGCGTTAATCGGAATTACTGGGCGTAAAGCGCACGCAGGCGGTCTGTTAAGTCAGATGTGAAATCCCCGGGCTCAACCTGGGAACTGCATCTGATACTGGCAGGCTTGAGTCTCGTAGAGGGGGGTAGAATTCCAGGTGTAGCGGTGAAATGCGTAGAGATCTGGAGGAATACCGGTGGCGAAGGCGGCCCCCTGGACGAAGACTGACGCTCAGGTGCGAAAGCGTGGGGAGCAAACAGG

>OTU708GTGGGGAATATTGCGCAATGGAGGAAACTCTGACGCAGCAATGCCGCGTGAGTGAAGAAGGGATTCGTTCCGTAAAGCTCTGTTGTCAGGGAAGAAAAAGACGGTACCTGACGAGCAAGCCCCAGCCAACCACGTGCCAGCAGCTGCGGTAATACGTGGGGGGCGAGCGTTATCCGGATTTATTGGGCGTAAAGGGCACGCAGGCGGACTTTCAGGCGCCGTGTTAAAGACCGGGGCTCAACCCCGGGAGGCCACGGCGAACCGGAGGTCTCGAGTGCTGGAGAGGCAGACAGAATTCCTGGTGTAGCGGTGAAATGCGCAGAGATCAGGAGGAATACCGGAGGCGAAGGCGGTCTGCCGGCCAGTGACTGACGCTCAGGTGCGAAAGCGTGGGGAGCGAACAGG

>OTU709GTGGGGAATTTTGGACAATGGGCGCAAGCCTGATCCAGCCATTCCGCGTGCAGGATGAAGGCCCTCGGGTTGTAAACTGCTTTTGTACGGAACGAAACGGCCTGCTCTAATACAGCGGGCTAATGACGGTACCGTAAGAATAAGCACCGGCTAACTACGTGCCAGCAGCCGCGGTAATACGTAGGGTGCAAGCGTTAATCGGAATTACTGGGCGTAAAGCGTGCGCAGGCGGTTATGTAAGACAGATGTGAAATCCCCGGGCTTAACCTGGGAACTGCATTTGTGACTGCATAGCTAGAGTACGGTAGAGGGGGATGGAATTCCGCGTGTAGCAGTGAAATGCGTAGATATGCGGAGGAACACCGATGGCGAAGGCAATCCCCTGGACCTGTACTGACGCTCATGCACGAAAGCGTGGGGAGCAAACAGG

>OTU710GTGGGGAATATTGCACAATGGGGGAAACCCTGATGCAGCAATGCCGCGTGAAGGATGAAGGTTTTCGGATTGTAAACTTCTTTTGTACGGGACGAAGAAAGTGACGGTACCGTAAGAATAAGCCACGGCTAACTACGTGCCAGCAGCCGCGGTAATACGTAGGTGGCAAGCGTTATCCGGATTTACTGGGTGTAAAGGGTGCGTAGGCGGCAAGGCAAGTCAGATGTGAAATGTAAGGGCTCAACCCTTAAGCTGCATTTGAAACTGTTTTGCTTGAGTGAAGTAGAGGCAGGCGGAATTCCCGGTGTAGCGGTGAAATGCGTAGAGATCGGGAGGAACACCAGTGGCGAAGGCGGCCTGCTGGGCTTTAACTGACGCTGAGGCACGAAAGCGTGGGTAGCAAACAGG

>OTU711GTGGGGAATATTGCGCAATGGAGGCAACTCTGACGCAGCAATGCCGCGTGAGTGAAGAAGGGATTCGTTTTGTAAAGCTCTGTTGCCGGGAAAGAGAGAGACGGTACCCGGCGAGCAAGCCCCAGCCAACTACGTGCCAGCAGCTGCGGTAATACGTAGGGGGCGAGCGTTATCCGGATTTATTGGGCGTAAAGGGCGCGCAGGCGGCCGGCCAGGCGCGGTGTGAAAGCCCGGGGCTCAACCCCGGAGGGTCACCGCGAACCGGTCGGCTCGAGTGCCGGAGAGGCAGACAGAATTCCTGGTGTAGCGGTGAAATGCGCAGAGATCAGGAGGAATACCGGAGGCGAAGGCGGTCTGCCGGCCGGAGACTGACGCTGAGGCGCGAAAGCGTGGGGAGCGAACAGG

>OTU712GTGAGGAATATTGGTCAATGGGCGGGAGCCTGAACCAGCCAAGCCGCGTGAGGGAAGAAGGCGCTCAGCGTCGTAAACCTCTTTAGCCGGGGAACAAAGAGCTGCTCGGGAAGCAGCGTTGAGCGTACCCGGAGAATAAGCATCGGCTAACTCCGTGCCAGCAGCCGCGGTAATACGGAGGATGCGAGCGTTATCCGGATTTATTGGGTTTAAAGGGTGCGCAGGCGGACGCGCAAGTCTGCGGTCAAATTCCGGGGCTCAACCCCGGACCGCCGTGGAAACTGTGCGACTAGAGTGGGCGAGAGGCATGCGGAATGCGCGGTGTAGCGGTGAAATGCTTAGAGATCGCGCAGAACTCCGATTGCGAAGGCAGCATGCCGGCGCCTTACTGACGCTCATGCACGAAAGCGTGGGGATCGAACAGG

>OTU713GTAGGGAATTTTGGACAATGGGGGAAACCCTGATCCAGCAACACTGCGTGGAGGATGAAGGCCTTCGGGTCGTAAACTCCTTTTGTCCCTGACGAAAAAGCCCGCAAGGGTCCTGACGGTAGGGGATGAATAAGCCACGGCTAACTTCGTGCCAGCAGCCGCGGTAAGACGAAGGTGGCAAGCGTTATTCGGAATCACTAGGCGTAAAGCGCGTGTAGGTGGGTGCTTAAGTCCGCTGTGAAATTTCCCGGCTTAACCGGGAAGGGTCAGCGGATACTGGGCATCTTGAGTTAGGCAGGGGGTACTGGAATTCCCGGTGTAGCGGTGAAATGCGTAGATATCGGGAGGAACACCAGTGGCGAAGGCGGGTACCTGGGCCTATACTGACACTAAGACGCGAAAGTTAGGGGAGCAAACAGG

>OTU714GTGAGGAATATTGGTCAATGGGCGAGAGCCTGAACCAGCCAAGCCGCGTGAAGGATGAAGGCGCTAAGCGTCGTAAACTTCTTTTGTCGGGGAACAACGGCATCCACGTGTGGGTGTATGAGTGTACCCGAAGAAAAAGCATCGGCTAACTCCGTGCCAGCAGCCGCGGTAATACGGAGGATGCGAGCGTTATCCGGATTTATTGGGTTTAAAGGGTGCGTAGGCGGTCCGGAAAGTCAGCGGTAAAAGCCCGGGGCTCAACCCCGGCGAGCCGTTGAAACTGTCGGACTAGAGACGGCGAGAAGTACGCGGAATGCGCGGTGTAGCGGTGAAATGCATAGATATCGCGCAGAACTCCGATTGCGAAGGCAGCGTACCGGCGCCGGACTGACGCTGAGGCACGAAAGCGTGGGTATCGAACAGG

>OTU715GTGAGGAATATTGGTCAATGGGCGCTAGCCTGAACCAGCCAAGTAGCGTGAGGGAAGAATGTCCTATGGATTGTAAACCTCTTTTGTCGGGGAGCAAGGCAGGAACGTGTTCCTGTTTGAGAGTACCCGAAGAAAAAGCATCGGCTAACTCCGTGCCAGCAGCCGCGGTAATACGGAGGATGCGAGCGTTATCCGGATTTATTGGGTTTAAAGGGTGCGTAGGCGGACTTTTAAGTCAGTGGTCAAATCGAGAGGCTCAACCTCTTTCCGCCATTGAAACTGGGAGTCTTGAGTGAGCGAGAAGTATGCGGAATGCGTAGTGTAGCGGTGAAATGCATAGATATTACGCAGAACTCCGATTGCGAAGGCAGCATACCGGCGCTCAACTGACGCTGAAGCACGAAAGCGTGGGTATCGAACAGG

>OTU716GTGGGGGATATTGCACAATGGGCGCAAGCCTGATGCAGCAACGCCGCGTGAGGGAAGACGGTTTTCGGATTGTAAACCTTTGTCTTTGGTGAAGAAGAAAGTGACGGTAACCAAGGAGGAAGCCACGGCTAACTACGTGCCAGCAGCCGCGGTAATACGTAGGTGGCAAGCGTTGTCCGGAATTACTGGGTGTAAAGGGAGCGCAGGCGGGACTGCAAGTTGGATGTGAAATACCGAGGCTCAACCTCGGGGCTGCATCCAAAACTGTAGTTCTTGAGTGAAGTAGAGGTAGGCGGAATTCCGAGTGTAGCGGTGAAATGCGTAGATATTCGGAGGAACACCAGTGGCGAAGGCGGCCTACTGGGCTTTAACTGACGCTGAGGCTCGAAAGTGTGGGGAGCAAACAGG

>OTU718GTGGGGAATCTTGCGCAATGGGCGAAAGCCTGACGCAGCGACGCCGCGTGAGTGATGAAGGCCTTCGGGTTGTAAAGCTCTGTGGGGAGGGACGAATAAGTGTTGGCTAATATCCAGCGCGATGACGGTACCTCTTTAGCAAGCACCGGCTAACTCTGTGCCAGCAGCCGCGGTAAGACAGAGGGTGCAAACGTTGTTCGGAATTACTGGGCGTAAAGCGTGTGTAGGCGGCTTGGCAAGTCGGATGTGAAAGCCCCGGGCTCAACCCGGGAAGTGCACTCGATACTGCCGAGCTTGAGTATCGGAGAGGTGGGTGGAATTCTCGGTGTAGAGGTGAAATTCGTAGATATCGAGAGGAACACCGGCGGCGAAGGCGGCCCACTGGACGAATACTGACGCTGAGACACGAAAGCGTGGGGAGCAAACAGG

>OTU720GTGAGGAATATTGGTCAATGGGCGGAAGCCTGAACCAGCCAAGTAGCGTGCAGGAAGACGGCCCTATGGGTTGTAAACTGCTTTTATACGAGAATAATTTGATGCACGTGTGCGTTATTGCATGTATCGTATGAATAAGGACCGGCTAATTCCGTGCCAGCAGCCGCGGTAATACGGAAGGTCCAGGCGTTATCCGGATTTATTGGGTTTAAAGGGAGTGTAGGCGGTTTGTTAAGCGTGTTGTGAAATTTAGGTGCTCAACATTTAACTTGCAGCGCGAACTGTCAGACTTGAGTACACGCAACGTATGCGGAATTCATGGTGTAGCGGTGAAATGCTTAGATATCATGAAGAACTCCGATTGCGAAGGCAGCATACGGGAGTGTAACTGACGCTTAAGCTCGAAGGTGCGGGTATCGAACAGG

>OTU721GTGAGGAATATTGCGCAATGGAGGCAACTCTGACGCAGCCATGCCGCGTGCAGGAAGAAGGCGCTACGCGTTGTAAACTGCTTTTATCCGGGAAGAAACCCTCCGACGTGTCGGAGGTTGACGGTACCGGAGGAATAAGCACCGGCTAACTTCGTGCCAGCAGCCGCGGTAATACGAAGGGTGCAAGCGTTATCCGGATTCATTGGGTTTAAAGGGTGCGTAGGTGGGGCGGTAAGTCAGTGGTGAAATCCTGCAGCTTAACTGTAGAATCGCCATTGATACTGTCGCTCTTGAGTACGCTTGACGTGGGCGGAATGTGCCGTGTAGCGGTGAAATGCTTAGATATGGCACAGAACACCGATAGTGAAGACAGCTCACGAAGGCGAAACTGACACTGAGGCACGAAAGCGTGGGGATCGAACAGG

>OTU722GTGGGGAATATTGCACAATGGGCGCAAGCCTGATGCAGCGACGCCGCGTGAGTGAAGAAGTATCTCGGTATGTAAAGCTCTATCAGCAGGGAAGAAAATGACGGTACCTGACTAAGAAGCCCCGGCTAACTACGTGCCAGCAGCCGCGGTAATACGTAGGGGGCAAGCGTTATCCGGATTTACTGGGTGTAAAGGGAGCGTAGACGGCGACGCAAGTCTGGAGTGAAAGCCCGGGGCCCAACCCCGGGACTGCTTTGGAAACTGTGTTGCTGGAGTGCAGGAGAGGTAAGTGGAATTCCTAGTGTAGCGGTGAAATGCGTAGATATTAGGAGGAACACCAGTGGCGAAGGCGGCTTACTGGACTGTAACTGACGTTGAGGCTCGAAAGCGTGGGGAGCAAACAGG

>OTU723GTGGGGAATTGTTCACAATGGGCGCAAGCCTGATGACGCAACGCCGCGTGGGGGATGAAGGTCTTCGGATCGTAAACCCCTGTCGAATGGGACGAATAGCCACCGGATCAATACCCCGGTGGAATGACGGTACCGTTAAAGGAAGCCACGGCTAACTCTGTGCCAGCAGCCGCGGTAATACAGAGGTGGCAAGCGTTGTTCGGAATTACTGGGCGTAAAGGGCGCGTAGGCGGCCATCTAAGTCAGACGTGAAATCCCCCGGCTCAACCTGGGAACTGCGTCTGATACTGGGAGGCTAGAGTTTGGGAGAGGGATGTAGAATTCCAGGTGTAGCGGTGAAATGCGTAGATATCTGGAGGAATACCGGTGGCGAAGGCGGCATCCTGGACCAATACTGACGCTGAGGCGCGAAAGCTAGGGGAGCAAACGGG

>OTU724GTGGGGAATATTGCGCAATGGGGGAAACCCTGACGCAGCAACGCCGCGTGCGGGATGAAGGCCTTCGGGTTGTAAACCGCTTTCAGCAGGGAAGACTTACGACGGTACCTGCAGAAGAAGCCCCGGCTAACTACGTGCCAGCAGCCGCGGTAATACGTAGGGGGCGAGCGTTATCCGGATTCATTGGGCGTAAAGCGCGCGCAGGCGGGCTGCTAAGCGGGACCTCTAATCTCGGGGCTCAACCTCGAGCCGGGTCCCGAACTGGCAGCCTCGAGTGCGGTAGGGGAGGTCGGAATTCCCGGTGTAGCGGTGGAATGCGCAGATATCGGGAAGAACACCGATGGCGAAGGCAGACCTCTGGGCCGACACTGACGCTGAGGCGCGAAAGCTAGGGGAGCGAACAGG

>OTU725GTGGGGGATATTGCGCAATGGGGGAAACCCTGACGCAGCAACGCCGCGTGAAGGAAGACGGTTTTCGGATTGTAAACTTCTTTTGTTAAGGACGAAAAAGATGACGGTACTTAACGAATAAGCTCCGGCTAACTATGTGCCAGCAGCCGCGGTAATACGGAGGGTGCAAGCGTTATCCGGATTTATTGGGTTTAAAGGGTCCGCAGGCGGATCGTTAAGTCAGTGGTGAAAGCCCACAGCTCAACTGTGGAATTGCCATTGATACTGGCGGTCTTGAGTATGTATGAAGTTGGCGGAATGTGTGGTGTAGCGGTGAAATGCTTAGATATCACGAAGAACTCCGATTGCGAAGGCAGCTTACTGGACTGTAACTGACGCTGATGCTCGAAAGTGTGGGTATCAAACAGG

>OTU726GTGAGGAATATTGGTCAATGGGCGGAAGCCTGAACCAGCCAAGTCGCGTGAGGGAAGACGGTCCTATGGATTGTAAACCTCTTTTGTCGGGGAGCAAAGCCGCTCACGTGTGAGCGGAAGGAGAGTACCCGAAGAAAAAGCATCGGCTAACTCCGTGCCAGCAGCCGCGGTAATACGGAGGATGCGAGCGTTATCCGGATTTATTGGGTTTAAAGGGTGCGCAGGCGGCGCGTTAAGTCAGCGGTAAAATTTCGGGGCTCAACCCCGACGAGCCGTTGAAACTGGCGTGCTAGAGTGGGCGAGAAGTATGCGGAATGCGTGGTGTAGCGGTGAAATGCATAGATATCACGCAGAACTCCGATTGCGAAGGCAGCATACCGGCGCCCAACTGACGCTCATGCACGAAAGCGTGGGTATCGAACAGG

>OTU727GTGGGGAATTGTTCGCAATGGGCGCAAGCCTGACGACGCAACGCCGCGTGGAGGATGAAGATCTTCGGGTCGTAAACTCCTTTCGATCGAGACGAATGGCCCCTGGGCTAATCACCAGGGGAATGACGGTACCGAGAGAAGAAGCCCCGGCTAACTCCGTGCCAGCAGCCGCGGTAATACGGGGGGGGCAAGCGTTGTTCGGAATTACTGGGCGTAAAGGGCTCGTAGGCGGCCAACTAAGTCATACGTGAAATCCCTCGGCTTAACCGGGGAACTGCGTCTGATACTGGATGGCTTGAGTTCGGGAGAGGGATGCGGAATTCCAGGTGTAGCGGTGAAATGCGTAGATATCTGGAGGAACACCGGTGGCGAAGGCGGCATCCTGGACCGATACTGACGCTGAGGAGCGAAAGCCAGGGGAGCAAACGGG

>OTU730GTGGGGAATATTGCACAATGGGGGAAACCCTGATGCAGCAACGCCGCGTGGAGGAAGAAGGTTTTCGGATTGTAAACTCCTGTCTTTTGGGACGATAATGACGGTACCAAAAGAGGAAGCCACGGCTAACTACGTGCCAGCAGCCGCGGTAATACGTAGGTGGCGAGCGTTGTCCGGAATTACTGGGCGTAAAGGGCGCGTAGGCGGCCTGATAAGTCAGATGTGAAACCCCCAGGCTCAACTTGGGGCATGCATTTGAAACTGTCGGGCTCGAGTGTCGGAGAGGAAAGCGGAATTCCTAGTGTAGCGGTGAAATGCGTAGATATTGGGGGGAACACCAGTGGCGAAGGCGACTTTCTGGACTGTAACTGACGCTGAGGCGCGAAAGCGTGGGGAGCAAACAGG

>OTU731GTGAGGAATATTGCGCAATGGACGAAAGTCAGACGCAGCAACGCCGCGTGAACGATGAAGGCCTTCGGGTTGTAAAGTTCTTTTCTGCGTGAAGAAACGGACGGTAGCGCAGGAATAAGTGTCGGCTAACTACGTGCCAGCAGCCGCGGTAAAACGTAGGACGCAAGCGTTATCCGGATTTACTGGGCGTAAAGCGCGTGCAGGCGGCCTGTTAAGTCGGACGTGAAAGCTCCCTGGCTCAACCGGGAGAGGTCGTTCGAAACTGGGAGGCTGGAGGGCAGCAGAGGAGTGTGGAATTCCGGGTGTAGTGGTGAAATGCGTAGATATCCGGAGGAACACCAGTGGCGAAGGCGGCACTCTGGGCTGTACCTGACGCTAAGACGCGAAAGCGTGGGTAGCGAACGGG

>OTU732GTAGGGAATATTGGTCAATGGTCGAGAGACTGAACCAGCCATGCCGCGTGCAGGAAGAAGGCCTTCTGGGTTGTAAACTGCTTTTATTTGGGGCGAAAAAGGCGATGCGTCGCTAGATGACGGTACCAGATGAATAAGCACCGGCTAACTCCGTGCCAGCAGCCGCGGTAATACGGAGGGTGCAAGCGTTGTCCGGATTTATTGGGTTTAAAGGGTGCGTAGGTGGCTAATTAAGTCAGTGGTGAAAGACGGCAGCTCAACTGTCGCAGTGCCATTGATACTGGTTAGCTTGAGTACAGACGAGGTAAGCGGAATTTATGGTGTAGCGGTGAAATGCATAGATACCATAAAGAACACCGATAGCGAAGGCAGCTTACCAGGGTGTAACTGACACTGAGGCACGAAAGCATGGGGATCAAACAGG

>OTU733GTGAGGAATATTGCACAATGGGCGCAAGCCTGATGCAGCAACGCCGCGTGAGGGACGAAGGCCTTCGGGTCGTAAACCTCTTTTCTGGGGGAAGAGACAGGACGGTACCTCAGGAATAAGTCTCGGCTAACTACGTGCCAGCAGCCGCGGTAAAACGTAGGAGGCGAGCGTTATCCGGATTCACTGGGTGTAAAGCGCATGCAGGCGGCCGGATAAGTTGGCTGTGAAATCTCCTGGCTCAACTGGGAGAGGCCGGTCAAGACTATCGGGCTCGAGGACGGTAGAGGAAGGTAGAATTCCCGGTGTAGTGGTGAAATGCGTAGATATCGGGAGGAATACCCGTGGCGAAAGCGGCCTTCTGGGCCGTTCCTGACGCTCAGATGCGAAAGCCAGGGGAGCAAACAGG

>OTU734GTGGGGAATATTGCACAATGGGGGAAACCCTGATGCAGCGACGCCGCGTGAGTGAGGAAGTATTTCGGTATGTAAAGCTCTATCAGCAGGGAAGAAAATGACGGTACCTGACTAAGAAGCCCCGGCTAACTACGTGCCAGCAGCCGCGGTAATACGTAGGGGGCAAGCGTTATCCGGATTTACTGGGTGTAAAGGGAGCGTAGACGGAATTGCAAGTCTGATGTGAAAATCCGGGGCCCAACCCCGGAACTGCATTGGAAACTGTATTTCTAGAGTGTCGGAGAGGCAAGTGGAATTCCTGGTGTAGCGGTGAAATGCGTAGATATCAGGAGGAACACCAGTGGCGAAGGCGGCTTGCTGGACGATGACTGACGTTGAGGCTCGAAAGCGTGGGGAGCAAACAGG

>OTU736GTGGGGAATATTGGACAATGGGCGCAAGCCTGATCCAGCCATACCGCGTGGGTGAAGAAGGCCTTCGGGTTGTAAAGCCCTTTTGTTGGGAAAGAAATCCTTCAGGCTAATACCCTGAGGGGATGACGGTACCCAAAGAATAAGCACCGGCTAACTTCGTGCCAGCAGCCGCGGTAATACGAAGGGTGCAAGCGTTACTCGGAATTACTGGGCGTAAAGCGTGCGTAGGTGGTTGCTTAAGTCTGCTGTGAAAGCCCTGGGCTCAACCTGGGAATTGCAGTGGATACTGGGCGACTAGAGTAAGGTAGAGGATAGTGGAATTTCCGGTGTAGCAGTGAAATGCGTAGAGATCGGAAGGAACATCTGTGGCGAAGGCGACTATCTGGGCCATTACTGACACTGAGGCACGAAAGCGTGGGGAGCAAACAGG

>OTU737GTGAGGAATATTGGTCAATGGGCGCGAGCCTGAACCAGCCAAGTCGCGTGAGGGATGGCGGTCCTACGGATTGTAAACCTCTTTTGCCGGGGAGCAAGCGTGCGTTCGTGAACGCGCGTCGAGAGTACCCGGAGAAAAAGCATCGGCTAACTCCGTGCCAGCAGCCGCGGTAATACGGAGGATGCGAGCGTTATCCGGATTTATTGGGTTTAAAGGGTGCGTAGGCGGAATGCCAAGTCAGCGGTAAAATCGCGGGGCTCAACCTCGCTCCGCCGTTGAAACTGGCGTTCTTGAGTGGGCGAGAAGTATGCGGAATGCGTGGTGTAGCGGTGAAATGCATAGATATCACGCAGAACTCCGATTGCGAAGGCAGCATACCGGCGCCCAACTGACGCTGAAGCACGAAAGCGTGGGTATCGAACAGG

>OTU738GTGGGGAATATTGGACAATGGGCGCAAGCCTGATCCAGCAATGCCGCGTGGGTGAAGAAGGCCTTCGGGTTGTAAAGCCCTTTTGTACGGGAAGAAATCCTGTCGGTTAATACCCGGCGGGGATGACGGTACCGGAAGAAGAAGCACCGGCTAACTACGTGCCAGCAGCCGCGGTAATACGTAGGGTGCGAGCGTTAATCGGAATTACTGGGCGTAAAGTGTGCGCAGGCGGCCGAGCAAGTCGAGTGTGAAAGCCCCGAGCTTAACTTGGGAACTGCGCTCGAAACTACTTGGCTAGAGTGTGGCAGAGGGAGGTGGAATTCCACGTGTAGCGGTGAAATGCGTAGAGATGTGGAGGAACACCGATGGCGAAGGCAGCCTCCTGGGCCAACACTGACGCTCATGCACGAAAGCGTGGGGAGCAAACAGG

>OTU739GTGGGGAATATTGCACAATGGAGGAAACTCTGATGCAGCGATGCCGCGTGAGGGAAGAAGGTTTTCGGATTGTAAACCTCTGTCTTAAGGGACGATAATGACGGTACCTTAGGAGGAAGCTCCGGCTAACTACGTGCCAGCAGCCGCGGTAATACGTAGGGAGCGAGCGTTGTCCGGAATTACTGGGTGTAAAGGGAGTGTAGGCGGGATGGTAAGTTAGGTGTGAAATGCGGAGGCTCAACCTCCGAGCTGCACTTAAAACTGCTGTTCTTGAGTGAAGTAGAGGTTGGCGGAATTCCCAGTGTAGCGGTGAAATGCGTAGATATTGGGAGGAACATCAGTGGCGAAGGCGGCCAACTGGGCTTTTACTGACGCTGAGGCTCGAAAGCGTGGGGAGCAAACAGG

>OTU740GTGGGGAATCATGGTCAATGGGCGAAAGCCTGAACCAGCAACGCCGCGTGTGCGAAGAAGGCCTTCGGGTCGTAAAGCACTTTTTGCCGGGAAGAGGAAGGACGGTACCGGCGGAATAAGCCTCGGCTAACTACGTGCCAGCAGCCGCGGTAAAACGTAGGAGGCGAGCGTTATCCGGATTTACTGGGTGTAAAGCGCGTGCAGGCGGACGGGTAAGTTGGGTGTGAAAGCTCCTGGCTAAACTGGGAGAGGTCGCTCAAGACTGCCGGTCTGGAGCATGGTAGGGGAAGGCGGAATTCCGGGTGTAGTGGTGAAATGCGTAGATATCCGGAGGAACACCAGTGGCGAAAGCGGCCTTCTAGACCATGACTGACGCTCAGACGCGAAAGCTAGGGTAGCAAACGGG

>OTU742GTGAGGAATATTGGTCAATGGGCGGAAGCCTGAACCAGCCAAGTCGCGTGAGGGAAGACGGTCCTATGGATTGTAAACCTCTTTTGCAGGGGAGCAAGGCGCCTTACGAGTAAGGCGTTGGAGAGTACCCTGAGAAAAAGCATCGGCTAACTCCGTGCCAGCAGCCGCGGTAATACGGAGGATGCGAGCGTTATCCGGATTTATTGGGTTTAAAGGGTGCGTAGGCGGGCTGGTAAGTCAGCGGTAAAAATGCGGTGCTCAACGCCGTAGTGCCGTTGAAACTGCCGGTCTTGAGTGAGCGAGAAGTATGCGGAATGCGTGGTGTAGCGGTGAAATGCATAGATATCACGCAGAACTCCGATTGCGAAGGCAGCATACCGGCGCTCAACTGACGCTGAGGCACGAAAGCGTGGGGATCGAACAGG

>OTU744GTGGGGAATTTTGCGCAATGGGCGAAAGCCTGACGCAGCGACGCCGCGTGGGTGAGGAAGTCCTTCGGGATGTAAAGCCCTTTTCTCGGGGACGAAACGGACGGTACCCGAGGAAGAAGCCCCGGCTAACTACGTGCCAGCAGCCGCGGTAATACGTAGGGGGCAAGCGTTGTCCGGATTTACTGGGCGTAAAGCGCGCGTAGGTGGAGCGATCAGTCCGGTCTGAAAGCCCCCGGCTCAACCGGGGAGGGTGGCTGGAAACTGTCGTTCTTGAGGGCGAGAGAGGAGGGTGGAATTCCTGGTGTAGCGGTGAAATGCGTAGAGATCAGGAGGAACACCCGTGGCGAAGGCGGCCCTCTGGCTCGACCCTGACGCTGAGGCGCGAAAGCGTGGGGAGCGAACGGG

>OTU745GTGAGGAATATTGGACAATGGGTGAGAGCCTGATCCAGCCATCCCGCGTGAAGGAATAAGGACCTATGGTTTGTAAACTTCTTTTGTACAGGGATAAACCTACTCACGTGTGAGTAGCTGAAGGTACTGTACGAATAAGCACCGGCTAACTCCGTGCCAGCAGCCGCGGTAATACGGAGGGTGCAAGCGTTATCCGGATTTATTGGGTTTAAAGGGTCCGTAGGCGGGCCCGTAAGTCAGTGGTGAAATCTCACAGCTTAACTGTGAAACTGCCGTTGATACTGCGGGTCTTGAGTGTAGTTGAGGTAGCTGGAATGAGTAGTGTAGCGGTGAAATGCATAGATATTACTCAGAACACCAATTGCGAAGGCAGGTTACCAAGTTACAACTGACGCTGAGGGACGAAAGCGTGGGGAGCGAACAGG

>OTU746GTGGGGAATTTTGGACAATGGGGGAAACCCTGATCCAGCCATGCCGCGTGCGGGAAGAAGGCCTTCGGGTTGTAAACCGCTTTTGTCGGGGAAGAAATCCCCCGGGTTAATACCTCGGGCTGATGACGGTACCGTAAGAATAAGCACCGGCTAACTACGTGCCAGCAGCCGCGGTAATACGTAGGGTGCGAGCGTTAATCGGAATTACTGGGCGTAAAGCGTGCGCAGGCGGTGATGTAAGACAGGTGTGAAATCCCCGGGCTTAACCTGGGAACTGCATTTGTGACTGCATTGCTGGAGTGCGGCAGAGGGGGATGGAATTCCGCGTGTAGCAGTGAAATGCGTAGATATGCGGAGGAACACCGATGGCGAAGGCAATCCCCTGGGCCTGCACTGACGCTCATGCACGAAAGCGTGGGGAGCAAACAGG

>OTU747GTCGAGAATCTTTTGCAATGCCCGAAAGGGTGACAATGCGACGCCGCGTGTGCGATGAAGGTCTTCGGATCGTAAAGCACTGTCAGCTGGGAGGAATCTCTGGAGTCTTAACAAGATTGCAGGGTGACAGTACCAGCAGAGGAAGCCACGGCTAACTCCGTGCCAGCAGCCGCGGTAATACGGAGGTGGCAGGCGTTACTCGGATTGATTGGGTGTAAAGGGCGCGTAGGCGGCCCTTCAAGTGGTAGGTGAAATCCCTCGGCTCAACCGAGGAGCTGCCCGCCAAACTGAAGAGCTTGAGTCCGGGAGAGGTGAGTGGAATTCCCAGTGTAGCGGTGAAATGCGTAGATATTGGGAGGAACACCAGTGGCGAAGGCGGCTCACTGGACCGGAACTGACGCTGAGGCGCGAAAGCGTGGGGAGCAAACAGG

>OTU748GTGGGGAATTTTGGACAATGGGGGCAACCCTGATCCAGCCATGCCGCGTGAGTGAAGAAGGCCTTCGGGTTGTAAAGCTCTTTCAGACGGAAAGAAACGGTCACGGCCAATACCCGTGACTAATGACGGTACCGTAAGAAGAAGCACCGGCTAACTACGTGCCAGCAGCCGCGGTAATACGTAGGGTGCGAGCGTTAATCGGAATTACTGGGCGTAAAGCGTGCGCAGGCGGTTTTGTAAGACAGACGTGAAATCCCCGGGCTTAACCTGGGAACTGCGTTTGTGACTGCAAGGCTAGAGTGTAGCAGAGGGGGGTAGAATTCCACGTGTAGCAGTGAAATGCGTAGATATGTGGAGGAATACCGATGGCGAAGGCAGCCCCCTGGGCTAACACTGACGCTCATGCACGAAAGCGTGGGGAGCAAACAGG

>OTU749GTTAGGAATATTCGTCAATGGGGGAAACCCTGAACGAGCAATGCCGCGTGAGTGATGACGGTCCTATGGATTGTAAAACTCTGTTGTTTGGAAAGAAATGTATAAGTAGGAAATGACTTATACTTGACGGTACCATTCAAGAAAGCCACGGCTAACTACGTGCCAGCAGCCGCGGTAATACGTAGGTGGCGAGCGTTATCCGGATTTATTGGGCGTAAAGCGTCCGCAGCCGGTTTTATAAGTCTAGAATTAAAGCCTGGGGCTCAACCCCAGTTCGTTCTAGAAACTGTAAGACTCGAGTGTGGTAGAGGCAAATGGAATTTCTAGTGTAGCGGTAAAATGCGTAGATATTAGAAGGAACACCAGTGGCGAAGGCGATTTGCTGGGCCATCACTGACGGTCAGGGACGAAAGCGTGGGGAGCAAATAGG

>OTU750GTGGGGAATCTTGCGCAATGGGCGGAAGCCTGACGCAGCGACGCCGCGTGCGGGACGAAGGCCTTCGGGTCGTAAACCGCTTTCAGCAGGGACGAGGCCCTCACGGTGACGGTACCTGCAGAAGAAGCCCCGGCTAACTACGTGCCAGCAGCCGCGGTAATACGTAGGGGGCGAGCGTTATCCGGATTCATTGGGCGTAAAGCGCGCGTAGGCGGCCTGTTAGGTCGGGGGTCAAATACCGGGGCTCAACCCCGGTCCGCCCCCGATACCGGCAGGCTTGAGTCTGGTAGGGGAAGGCGGAATTCCCAGTGTAGCGGTGGAATGCGCAGATATTGGGAAGAACACCGGCGGCGAAGGCGGCCTTCTGGGCCACGACTGACGCTGAGGCGCGAAAGCTAGGGGAGCGAACAGG

>OTU751GTGGGGAATATTGCACAATGGGGGAAACCCTGATGCAGCGACGCCGCGTGAGCGAAGAAGTATTTCGGTATGTAAAGCTCTATCAGCAGGGAAGAAAAATGACGGTACCTGACCAAGAAGCCCCGGCTAACTACGTGCCAGCAGCCGCGGTAATACGTAGGGGGCAAGCGTTATCCGGATTTACTGGGTGTAAAGGGAGCGTAGGCGGCGGCGCAAGTCAGAAGTGAAAGCCCGGGGCTCAACTCCGGGACTGCTTTTGAAACTGCGTTGCTAGATTGCGGGAGAGGCAAGTGGAATTCCTAGTGTAGCGGTGAAATGCGTAGATATTAGGAGGAACACCAGTGGCGAAGGCGGCTTGCTGGACCGTGAATGACGCTGAGGCTCGAAAGCGTGGGGAGCAAACAGG

>OTU752GTGGGGAATCTTGGACAATGGGGGCAACCCTGATCCAGCCATGCCGCGTGAGCGATGAAGGCCCTAGGGTTGTAAAGCTCTTTCACCCGTGAAGATAATGACGGTAGCGGGAGAAGAAGCCCCGGCTAACTCCGTGCCAGCAGCCGCGGTAATACGGGGGGGGCAAGCGTTGTTCGGAATTACTGGGCGTAAAGGGCCAGTAGGCGGCCAACTAAGTCGGATGTGAAATCCCCAGGCTTAACCTGGGAACTGCATCCGATACTGGATGGCTTGAATTCGGGAGAGGGATGCAGAATTCCAGGTGTAGCGGTGAAATGCGTAGATATCTGGAGGAATACCGGTGGCGAAGGCGGCATCCTGGACCGACATTGACGCTGAACGGCGAAAGCCAGGGGAGCAAACGGG

>OTU754GTGAGGAATATTGGGCAATGGGCGCAAGCCTGACCCAGCCATGCCGCGTGAAGGATGAATGCCCTATGGGTTGTAAACTTCTTTTGTACGGGACGAAACTTCCGGTCGTGACCGGGATTGACTGTACCGTGCGAATAAGCATCGGCTAACTCCGTGCCAGCAGCCGCGGTAATACGGAGGATGCAAGCGTTATCCGGATTCATTGGGTTTAAAGGGTGCGTAGGCGGAATAATAAGTCAGTGGTGAAAGCCTGTGGCTTAACCATAGAATTGCCATTGATACTGTTATTCTTGAGTATAGTTGAAGTGGGCGGAATGTGTAATGTAGCGGTGAAATGCTTAGATATTACACAGAACACCAATTGCGAAGGCAGCTCACTAAGCTGTTACTGACGCTGAGGCACGAAAGCGTGGGGAGCAAACAGG

>OTU756GTCGAGAATAATTCACAATGGGGGAAACCCTGATGGTGCAACGCCGCGTGGAGGATGAAGGCCCTAGGGTCGTAAACTCCTGTCATTCGAGAGCAAGACCTGGGTGTTAACAGCACACAGGGTTGATAGTATCGAAAGAGGAAGGGACGGCTAACTTCGTGCCAGCAGCCGCGGTAATACGAAGGTCCCGAGCGTTGTTCGGAATCACTGGGCGTAAAGGGAGCGTAGGCGGTTCGGTAAGTCAGGTGTGAAATCCCGGGGCTCAACCCCGGAACTGCACCCGATACTGCCGGACTTGAGTGATGGAGGGGTAACTGGAATTCTCGGTGTAGCAGTGAAATGCGTGGATATCGAGAGGAACACTCGTGGCGAAGGCGAGTTACTGGACATCTACTGACGCTGAGGCTCGAAGGCCAGGGGAGCGAAAGGG

>OTU757GTGAGGAATATTGGTCAATGGCCGGGAGGCTGAACCAGCCAAGTCGCGTGAGGGAAGACGGCCCTACGGGTTGTAAACCTCTTTTGTCAGGGAGCAAGAGGCGGGTCGAGACCTGCTGTGAGAGTACCTGAAGAAAAAGCATCGGCTAACTCCGTGCCAGCAGCCGCGGTAATACGGAGGATGCGAGCGTTATCCGGATTTATTGGGTTTAAAGGGTGCGCAGGCGGAGGCACAAGTCAGCGGTAAAATCGCGGGGCTCAACCCCGCTCCGCCGTTGAAACTGTGTCCCTTGAGTGCGCGAAGGGTAGGCGGAATGCGTGGTGTAGCGGTGAAATGCATAGATATCACGCAGAACTCCGATCGCGAAGGCAGCCTACCGGCGCGCAACTGACGCTCATGCACGAAAGCGTGGGTATCGAACAGG

>OTU758GTCGAGAAGCTTCGGCAATGGGCGAAAGCCTGACCGAGCGACGCCGCGTGAGCGATGAAGGCCCTAGTGGTTGTAAAGCTCTGTCAGATTGGGAATAAACGCCGGGGGTGAATACCCCCCGGAACTGAAGGTACCGTCAGAGGAAGCACCGGCTAACTCCGTGCCAGCAGCCGCGGTAATACGGAGGGTGCAAGCGTTGTTCGGATTCACTGGGTATAAAGGGTTCGTAGGCGGCCCCGTAAGTCAGAGGTGAAAGCTGCCGGCTCAACTGGCAAATTGCCTTTGAAACTGCAGGGCTTGAGTCCGGAAGAGGAAGGTGGAATTCCTGGTGTAGCGGTGAAATGCGTAAATATCAGGAGGAACACCGGTGGCGAAGGCGGCCTTCTGGTCCGGTACTGACGCTGAGGAACGAAAGCCAGGGGAGCAAACGGG

>OTU759GTAACGAATCTTCCGCAATGCGCGAAAGCGTGACGGAGCAATGCCGCGTGCAGGATGAAGCCCCTCGGGGTGTAAACTGCTGTCAGGGATTAGGAACACAATGACCAGTCCCAAAGGAAGAGACGACTAACTCTGTGCCAGCAGTCGCGGTAATACAGAGGTCTCGAGCGTTAGTCGGAATCACTGGGCTTAAAGCGTGCGTAGGCGGATGTGCAGGCGTCGTGTGAAAGCCCTCGGCTCAACCGGGGAATTGCACGGCGAACCGCACGTCTCGAGACAAGTAGGGGCGGCCGGAACGATAGGTGGAGTGGTGAAATGCGTTGATATCTATCTGAACGCCAAAGGTGAAGACAGGCCGCTGGGCTTGTTCTGACGCTGAGGCACGAAAGCGTGGGGAGCAAACGGG

>OTU760GTGAGGAATATTGGTCAATGGAGGCAACTCTGAACCAGCCATGCCGCGTGCAGGATGACGGTCCTATGGATTGTAAACTGCTTTTGCACAGGAAGAAACAACTCTACGTGTAGAGTCTTGACGGTACTGTGAGAATAAGGATCGGCTAACTCCGTGCCAGCAGCCGCGGTAATACGGAGGATCCAAGCGTTATCCGGAATCATTGGGTTTAAAGGGTCCGTAGGCGGTCAGGTAAGTCAGTGGTGAAATCCCATCGCTCAACGGTGGAACGGCCATTGATACTGTCTGACTTGAATTATTAGGAAGTAACTAGAATATGTAGTGTAGCGGTGAAATGCTTAGAGATTACATGGAATACCAATTGCGAAGGCAGGTTACTACTAATATATTGACGCTGATGGACGAAAGCGTGGGTAGCGAACAGG

>OTU761GTGAGGAATATTGCGCAATGGGGGAAACCCTGACGCAGCGACGCCGCGTGGGCGACGAAGGCCTTCGGGTCGTAAAGCCCTTTTGGCAGGGACGAGTGAGGACGGTACCTGCTGAATAAGTCTCGGCTAACTACGTGCCAGCAGCCGCGGTAAAACGTAGGAGGCGAGCGTTATCCGGATTCACTGGGCGTAAAGCGCGCGTAGGCGGTTGGGTAAGTCGGACGTGAAAGCCCCTGGCTCAACTGGGGGAGGCCGTTCGATACTGCCCGGCTTGAGGGTGAGAGAGGGAAGTGGAATTCCCGGTGTAGCGGTGGAATGCGTAGAGATCGGGAGGAACACCAGTGGCGAAAGCGGCTTCCTGGCTCACTCCTGACGCTGAGAGCGCGAAAGCGTGGGGAGCAAACGGG

>OTU762GTAGGGAATCTTCCACAATGGGCGAAAGCCTGATGGAGCAACGCCGCGTGGGTGAAGAAGGTCTTCGGATCGTAAAACCCTGTTGTTAGAGAAGAAAGTGCGTGAGAGTAACTGTTCACGCAGTGACGGTACCCAACCAGAAAGTCACGGCTAACTACGTGCCAGCAGCCGCGGTAATACGTAGGGGGCAAGCGTTATCCGGAATTACTGGGTGTAAAGGGAGCGTAGACGGTTAGGTAAGTCTGGAGTGAAAGGCGGGGGCCCAACCCCCGGACTGCTCTGGAAACTATCGGACTAGAGTGCAGGAGAGGTAAGCGGAATTCCTAGTGTAGCGGTTAAATGCGTAGATATTAGGAGGAACACCAGTGGCGAAGGCGGCTTACTGGACTGTAACTGACGTTGAGGCTCGAAAGCGTGGGGAGCAAACAGG

>OTU763GTGAGGGATATTGCACAATGGGGGAAACCCTGATGCAGCGACGCCGCGTGGGCGATGAAGGTCTTCGGATCGTAAAGCCCTTTTCTGTGTGACGAGCAAGGACGGTAGCACAGGAATAAGTCTCGGCTAACTACGTGCCAGCAGCCGCGGTAAAACGTAGGAGGCAAGCGTTATCCGGAGTTACTGGGCGTAAAGCGCAAGTAGGCGGTTGTGCAAGTTGGTTGTGAAAGCGCCCGGCTAAACTGGGCGAGGCCGATCAAGACTGCACGGCTAGAGAGTGCTAGAGGGGCGCAGAATTCGGGGTGTAGCGGTGAAATGTGTAGAGATCCCGAGGAATACCAGTGGGGAAGCCGGCGCCCTGGGGCATATCTGACGCTGAGATGCGAAAGCGTGGGGAGCGAACGGG

>OTU764GTGGGGAATTTTGGACAATGGGCGAAAGCCTGATCCAGCAATGCCGCGTGAGTGAAGAAGGCCTTCGGGTTGTAAAGCTCTTTTGTCAGGGAAGAAAAGGCTATGGCTAATATCCATGGCTGATGACGGTACCTGAAGAATAAGCACCGGCTAACTACGTGCCAGCAGCCGCGGTAATACGTAGGGTGCGAGCGTTAATCGGAATTACTGGGCGTAAAGGGTGCGCAGGCGGTTGTACAAGACGGATGTGAAATCCCCGGGCTTAACCTGGGAATGGCATTCGTGACTGTACGGCTAGAGTGTGTCAGAGGGGGGTAGAATTCCACGTGTAGCAGTGAAATGCGTAGATATGTGGAGGAATACCGATGGCGAAGGCAGCCCCCTGGGATAACACTGACGCTCATGCACGAAAGCGTGGGGAGCAAACAGG

>OTU765GCTAAGAATCTTGCGCAATGGGCGAAAGCCTGACGCAGCAATTCCGCGTGGGGGACGAAGGCCCTCGGGTCGTAAACCCCTTTTACGAGGGAAGAAGTTCTGACGGTACCTCGTGAATAAGACACGGCTAACTACGTGCCAGCAGCCGCGGTAATACGTAGGTGTCGAGCGTTGTCCGGATTTATTGGGCGTAAAGCGCCCGCAGGCGGCTTGCCAAGTTCGAAGTGAAATCTCCCGGCTTAACTGGGAGGGTGCTCCGAAAACTGGCAGGCTTGAGTCATGGAGAGGGATGTGGAATTCCCGGTGTAGTGGTGAAATGCGTAGATATCGGGAGGAACACCAGTGGCGAAGGCGGCATCCTGGCCATGTACTGACGCTCAGGGGCGAAAGCGTGGGGAGCAAACCGG

>OTU767GTGGGGAATCTTGCGCAATGGCCGCAAGGCTGACGCAGCGACGCCGCGTGTGGGAGGACGCCCTTCGGGGTGTAAACCACTGTTGCCCGGGACGAACCTTCCCTTTCGAGGGGACTGACGGTACCGGAAGAATAAGCACCGGCTAACTACGTGCCAGCAGCCGCGGTAATACGTAGGGTGCGAGCGTTAATCGGAATTACTGGGCGTAAAGCGTGCGTAGGCGGCGATGCAAGACAGATGTGAAATCCCCGGGCTTAACCTGGGAACTGCATTTGTGACTGCATGGCTAGAGTGTGTCAGAGGGAGGTGGAATTCCGCGTGTAGCAGTGAAATGCGTAGAGATGCGGAGGAACACCGATGGCGAAGGCAGCCTCCTGCGATAACACTGACGCTCAGGCACGAAAGCGTGGGGAGCAAACAGG

>OTU768GTGGGGAATATTGCACAATGGGGGGAACCCTGATGCAGCAACGCCGCGTGAGTGAAGAAGTATTTCGGTATGTAAAGCTCTATCAGCAGGGAAGAAAATGACGGTACCTGACTAAGAAGCTCCGGCTAAATACGTGCCAGCAGCCGCGGTAATACGTATGGAGCAAGCGTTATCCGGATTTACTGGGTGTAAAGGGAGCGCAGGCGGTGCGGCAAGTCTGATGTGAAAGCCCGGGGCTCAACCCCGGGACTGCATTGGAAACTGCCGCACTAGAGTGCCGGAGAGGTAAGTGGAATTCCTAGTGTAGCGGTGAAATGCGTAGATATTAGGAGGAACACCAGTGGCGAAGGCGGCTTACTGGACGGTAACTGACGCTGAGGCTCGAAAGCGTGGGGAGCAAACAGG

>OTU769GTGGGGAATATTGGACAATGGGGGCAACCCTGATCCAGCAACGCCGCGTGAATGATGAAGGCCTTCGGGTCGTAAAGTTCTTTTAGCAGGGAAGAAAATGACGGTACCTGCAGAATAAGCACCGGCTAACTTCGTGCCAGCAGCCGCGGTAATACGAAGGGTGCTAGCGTTGTTCGGAATTACTGGGCGTAAAGCGTGCGTAGGTTGTTTGGTTAGTTAGTGGTGAAAGCCCGGGGCTTAACTCCGGAATTGCCATTAAAACTGCCAGGCTAGAGAGTGTTAGAGGTGAGTGGAATTTCTAGTGTAGGAGTGAAATCCGTAGATATTAGAAGGAACATCAATGGCGAAGGCAGCTCACTGGGTCACATCTGACACTGAGGCACGAAAGCGTGGGTAGCAAACAGG

>OTU771GTCGAGAATTTTTCACAATGGGGGCAACCCTGATGGAGCGACGCCGCGTGGAGGATGAAGGCCTTCGGGTTGTAAACTCCTGTCATGCGGGAACAAGAAAGTGATAGTACCGCAAGAGGAAGAGACGGCTAACTCTGTGCCAGCAGCCGCGGTAATACAGAGGTCTCAAGCGTTGTTCGGATTCATTGGGCGTAAAGGGTGCGTAGGTGGTGATGCAAGTCTGGTGTGAAATCTCGGAGCTTAACTCCGAAATTGCACCGGATACTGCGTTGCTCGAGGACTGTAGAGGAGATCGGAATTCACGGTGTAGCAGTGAAATGCGTAGATATCGTGAGGAAGACCAGTTGCGAAGGCGGATCTCTGGGCAGTTCCTGACACTGAGGCACGAAGGCCAGGGGAGCAAACGGG

>OTU772GTAGGGAATATTGGTCAATGGGCGAGAGCCTGAACCAGCCATGCCGCGTGCAGGATGACAGCCTTCTGGGTTGTAAACTGCTTTTATACAGGAAGAAAAAGTCCTTGCGAGGAACATTGCCGGTACTGTATGAATAAGCACCGGCTAACTCCGTGCCAGCAGCCGCGGTAATACGGAGGGTGCAAGCGTTGTCCGGATTTATTGGGTTTAAAGGGTGCGTAGGCGGGTCTTTAAGTCAGTGGTGAAATCCTACAGCTTAACTGTAGAACTGCCATTGATACTGGAGACCTTGAATTCAGTTGAGGTAGGCGGAATTAATGATGTAGCGGTGAAATGCATAGATATCATTAAGAACACCGATTGCGAAGGCAGCTTACTGGGCTTGAATTGACGCTGAGGCACGAAAGCGTGGGGAGCGAACAGG

>OTU773GTAGGGAATCTTTCACAATGGGCGAAAGCCTGATGAAGCAACACCGCGTGAATGAAGAAGGGTTTCGGCTCGTAAAATTCTGTTGCTAGAGAAGAACGTACCAAGTAGGAAATGGCTTGGTAGTGACGGTATCTGGTTAGAAAGTCACGGCTAACTACGTGCCAGCAGCCGCGGTAATACGTAGGTGGCAAGCGTTATCCGGATTTATTGGGCGTAAAGCGAGCGCAGGCGGTTGTTTAAGTCCGATGTGAAAGCCTTCGGCTTAACCGAAGAAGTGCATTGGAAACTGAGCGACTTGAGTACAGAAGAGGACAGTAGAACACCATGTGTAGCGGTGAAATGCGTAGATATATGGTAGAATACCAGTGGCGAAAGCGGCTGTCTGGTCTGTCACTGACGCTGAGGCTCGAAAGCATGGGTAGCAAACAGG

>OTU774GTGAGGAATATTGGTCAATGGGCGCAAGCCTGAACCAGCCATCCCGCGTGCAGGAAGAAGGCGCTATGCGTTGTAAACTGCTTTTGCAGGGGAAGAATGTTCTGTATGCATACGGAATTGACGGTACCTTGTGAATAAGCATCGGCTAACTCCGTGCCAGCAGCCGCGGTAATACGGAGGATGCGAGCGTTATCCGGATTTATTGGGTTTAAAGGGTGCGTAGGCGGATTGATAAGTCAGTGGTGAAATACTGCAGCTTAACTGTAGAATTGCCGTTGATACTGTTAGTCTTGAGTACGGTCAAGGTAGGCGGAATGTGTAATGTAGCGGTGAAATGCTTAGATATTACACAGAACACCGATTGCGAAGGCAGCTTACTGGGCCGCAACTGACGCTGATGCACGAAAGCGTGGGGAGCGAACAGG

>OTU775GTAGGGAATCTTGCACAATGGGCGCAAGCCTGATGCAGCGATGCCGCGTGAGGGATGAAGGCCTTCGGGTTGTAAACCTCTTTCGACAGGGAAGAAGCCACCCTTCAAGGTGGTGACGGTACCTGTATAAGAAGCCCCGGCTAACTACGTGCCAGCAGCCGCGGTAAAACGTAGGGGGCAAGCGTTGCCCGGATTCACTGGGCGTAAAGCGGGTGTAGGCGGTCCTCTAAGTCAGATGTGAAATCCTACGGCTCAACCGTGGAACTGCATCTGATACTGGAGGACTTGAGGACGGTAGAGGTCGGTGGAATTCCCGGTGTACCGGTGAAATGGGCAGATATCGGGAGGAACACCAGTGGCGAAGGCGGCCGACTGGGCCGATCCTGACGCTGAGACCCGAAAGCCAGGGTAGCGAACCGG

>OTU776GTGGGGAATCTTGCGCAATGGTCGAAAGACTGACGCAGCGACGCCGCGTGCGGGAGGAAGCTCTTCGGGGTGTAAACCGCTGTTGCCCGGAACGAAACGCCTTTTTCGAGAGGCTTGACGGTACCGGGTGAGGAAGCGCCGGCTAACTCTGTGCCAGCAGCCGCGGTAATACAGAGGGCGCGAGCGTTGTCCGGAATCACTGGGCGTAAAGGGCGCGTAGGTGGCGGCATAAGGCCGTGGTGAAAGTCTGGGGCTCAACTCCAGATCGGCCATGGCGACTGTGTCGCTAGGGCACCGTAGAGGCAGATGGAATTCCGGGTGTAGCGGTGGAATGCGTAGAGATCCGGAAGAACACCAGTGGCGAAGGCGGTCTGCTGGGCGGTGAGCTCGCAAGGGCTCAATAGCCGACACTGAGGCGCGACAGCGTGGGGAGCAAACAGG

>OTU777GTGGGGAATATTGCACAATGGGGGAAACCCTGATGCAGCGACGCCGCGTGAGGGAAGAAGGTATTCGTATTGTAAACCTCTGTCTTCGTTGACGATAATGACGGTAAACGAGGAGGAAGCACCGGCTAACTACGTGCCAGCAGCCGCGGTAAAACGTAGGGTGCAAGCGTTGTCCGGAATTACTGGGTGTAAAGGGAGCGCAGGCGGTTCTTTAAGTTGGATGTTTAATCTACGGGCTCAACCCGTATTCGCATTCAAAACTGAAGAACTTGAGTTAAGTAGAGGTAGGCGGAATTTCCGGTGTAGCGGTGGAATGCGTAGATATCGGAAGGAACACCAGTGGCGAAGGCGGCCTGCTGGACATTAACTGACGCTGAGGAGCGAAAGCGTGGGGAGCAAACAGG

>OTU778GTGGGGAATATTGGACAATGGGGGCAACCCTGATCCAGCGACGCCGCGTGTGTGAAGAAGGCCTGCGGGTTGTAAAGCACTTTTAGTGGGGATGAAAAGCACAGGGCTAATACTTCTGTGTCTTGACCTAACCCAAAGAAAAAGCACCGGCTAACTCTGTGCCAGCAGCCGCGGTAATACAGAGGGTGCAAGCGTTAATCGGAATTACTGGGCGTAAAGCGTGCGTAGTCGGTTATTTAAGTCGGATGTGAAATCCCCGGGCTCAACCTGGGAATTGCATTCGATACTGGATGGCTAGAGTTCGGCAGAGGGAAGTGGAATTTCTGGTGTAGCGGTGAAATGCGTAGATATCAGAAGGAACATCAGTGGCGAAAGCGACTTCCTGGACCAGAACTGACGATCAGGCACGAAAGCGTGGGGAGCAAACAGG

>OTU781GTGGGGAATATTGGACAATGGGGGCAACCCTGATCCAGCAATGCCGCGTGTGTGAAGAAGGCCTTCGGGTTGTAAAGCACTTTTATCCGGAACGAAACGCGCTTGGTGAATAGCCAGGCGAACTGACGGTACCGGAGGAATAAGCACCGGCTAACTTCGTGCCAGCAGCCGCGGTAATACGAAGGGTGCAAGCGTTACTCGGAATTACTGGGCGTAAAGCGTGCGTAGGTGGTTTGTTAAGTCTGTTGTGAAAGCCCCGGGCTCAACCTGGGAATGGCAATGGATACTGGCAGGCTAGAGTGCGGTAGAGGGTAGTGGAATTCCCGGTGTAGCGGTGAAATGCGTAGAGATCGGGAGGAACACCAGTGGCGAAGGCGGCTACCTGGACCAGCACTGACACTGAGGCACGAAAGCGTGGGGAGCAAACAGG

>OTU783GTGGGGAATATTGCGCAATGGGCGAAAGCCTGACGCAGCCAATCCGCGTGGGTGATGAAGGCCTTCGGGTTGTAAAGCCCTGTCGTGAGGGACGAAGTTCTGACGGTACCTCACAAGAAAGCACCGGCTAACCCCGTGCCAGCAGCCGCGGTAATACGGGGGGTGCAAGCGTTGCTCGGAATCACTGGGCGTAAAGCGTCCGCAGGTGGAAAGGTCAGTCGGTCGTGAAAGCCCGGGGCTCAACCCCGGAAATGCGATCGAAACTTCCTTTCTTGAGTTCGGGAGAGGGGAGTGGAATTCCTGGTGTAGAGGTGAAATTCGTAGATATCAGGAGGAACACCGGTGGCGAAAGCGGCTCCCTGGCCCGAAACTGACACTCATGGACGAAAGCGTGGGTAGCAAACAGG

>OTU784GTGGGGAATATTGCGCAATGGAGGCAACTCTGACGCAGCGATGCCGCGTGGGCGATGAAGGGCCCAGGCCCGTAAAGCCCCGCCGCCGGGGAGGAAGGAATGACTGAACCCGGCACGAAAGCCCCGGCCAACCACGTGCCAGCAGCCGCGGCAATACGTGGGGGGCGAGCGTTGTCCGGAATCACTGGGCGTAAAGCGCGCGCAGGCGGCCGCTTAGGCGGGGTGTGAAAAGCCGGGGCCCAACCCCGGGCAAGCGCCCCGGACCGGGCGGCTGGAGTGCCGGAGGGGCGGGCGGAACTCCTGGCGTAGCGGTGAAATGCGCAGAGGCCAGGAGGAACACCGGAGGCGAAGGCGGCCCGCCGGACGGACACTGACGCTGAGGCGCGAGAGCGCGGGGAGCGAACAGG

>OTU785GTGGGGAATCTTGCGCAATGGGCGAAAGCCTGACGCAGCAACGCCGCGTGCGGGATGAAGGCCTTCGGGTTGTAAACCGCTTTCAGTGGGGACGAACCAAGACGGTACCCACAGAAGAAGCCCCGGCCAACTACGTGCCAGCAGCCGCGGTAACACGTAGGGGGCAAGCGTTGTCCGGATTTATTGGGCGTAAAGAGCTCGTAGGTGGCTCAGTAAGTCGGGTGTGAAACCTCCAGGCTCAACCTGGAGACGCCACCCGATACTGCTGTGGCTAGAGTCCAGTAGGGGAGCGTGGAATTCCTGGTGTAGCGGTGAAATGCGCAGATATCAGGAGGAACACCAGTGGCGAAGGCGGCGCTCTGGGCTGGAACTGACACTGAGGAGCGAAAGCGTGGGGAGCGAACAGG

>OTU786GTGGGGAATATTGCACAATGGGGGAAACCCTGATGCAGCGACGCCGCGTGAGTGAAGAAGTATTTCGGTATGTAAAGCTCTATCAGCAGGGAAGAAGATGACGGTACCTGCAGAAGCAGCCCCGGCTAACTACGTGCCAGCAGCCGCGGTAATACGTAGGGGGCAAGCGTTATCCGGATTTACTGGGTGTAAAGGGAGCGTAGACGGATTGGCAAGTCAGATGTGAAAGGCGGGGGCTTAACCCCTGGATTGCATTTGAAACTGCGGATCTTGAGTACCGGAAGGGCAGATGGAATTCCTGGTGTAGCGGTGAAATGCGTAGAGATCAGGAGGAACACCGGTGGCGAAAGCGGTCTGCTGGACGGTAACTGACGTTGAGGCTCGAAGGCGTGGGGAGCAAACAGG

>OTU787GTGGGGAATATTGGGCAATGGGCGAAAGCCTGACCCAGCAACGCCGCGTGAAGGAAGAAGGTTTTCGGATCGTAAACTTCTATCCTTGGTGAAGATAATGACGGTAGCCAAGAAGGAAGCCCCGGCTAACTACGTGCCAGCAGCCGCGGTAATACGTAGGGGGCAAGCGTTGTCCGGAATGATTGGGCGTAAAGGGCGCGTAGGCGGCCCGGTAAGTCTGAAGTGAAAGTCCTGCTTTTAAGGTGGGAATTGCTTTGGATACTGTCGGGCTTGAGTGCAGGAGAGGTAAGCGGAATTCCCGGTGTAGCGGTGAAATGCGTAGAGATCGGGAGGAACACCAGTGGCGAAGGCGGCTTACTGGACTGTAACTGACGCTGAGGCGCGAAAGTGTGGGGAGCAAACAGG

>OTU788GTGGGGAATCTTGGACAATGGGGGAAACCCTGATCCAGCGATGCCGCGTGAGTGATGAAGGCCTTCGGGTTGTAAAACTCTTTCGACGGGGACGATAATGACGGTACCCGTAGAAGAAGCTCCGGCTAACTTCGTGCCAGCAGCCGCGGTAATACGAAGGGGGCTAGCGTTGTTCGGAATTACTGGGCGTAAAGCGTGCGCAGGCGGCCATTCAAGTCAGGGGTGAAAGCCCAGAGCTCAACTCTGGAACTGCCTTTGAAACTGTATGGCTTGAGTATGAGAGAGGTGAGTGGAATTCCCAGTGTAGAGGTGAAATTCGTAGATATTGGGAAGAACACCGGTGGCGAAGGCGGCTCACTGGCTCATAACTGACGCTCATGCACGACAGCGTGGGGATCAAACAGG

>OTU790GTGGGGAATTTTCCGCAATGGGCGAAAGCCTGACGGAGTGACGCCGCGTGGGGGACGAAGGCCTTCGGGTCGTAAACCCCTGTCAGAGGGAAAGAAGGGTCGCCGACCTAATACGTCGGTGATCTGACGGTACCTTCAGAGGAAGCCCCGGCCAACTCTGTGCCAGCAGCCGCGGTAAGACAGAGGGGGCAAGCGTTGCTCGGAATCACTGGGCGTAAAGGGCGCGTAGGCGGGATGGCAAGTCAGTCGTGAAATCTCTCGGCTCAACCGAGAAACGTCGGCTGATACTGCCGTTCTTGAGGGATGCAGAGGAGACTGGAATTCCCGGTGTAGCGGTGAAATGTGTAGAGATCGGGAGGAACACCGGTGGCGAAGGCGGGTCTCTGGGCATTTCCTGACGCTGAGGCGCGAAAGCGTGGGGAGCAAACGGG

>OTU791GTGGGGAATATTGCGCAATGGGGGGAACCCTGACGCAGCAATGCCGCGTGAAGGATGAAGGTTTTCGGATTGTAAACTTCTTTTACTGAGGACGAAGAATGACGGTACTCAGAGAATAAGCCACGGCTAACTACGTGCCAGCAGCCGCGGTAATACGTAGGGGGCAAGCGTTATCCGGATTTACTGGGTGTAAAGGGAGCGTAGGTGGCATGGCAAGCCAGAAGTGAAAACCCGGGGCTTAACTCCGCGGATTGCTTTTGGAACTGTCAGGCTGGAGTGCAGGAGGGGCAGGCGGAATTCCTGGTGTAGCGGTGAAATGCGTAGATATCAGGAAGAACACCAGTGGCGAAGGCGAGTTGCTGGACTGTAACTGACACTGAGGCACGAAAGCGTGGGGAGCAAACAGG

>OTU792GTGAGGAATATTGGTCAATGGGCGCAAGCCTGAACCAGCCAAGTCGCGTGAGGGAAGACGGTCCTAAGGATTGTAAACCTCTTTTGTCAGGGAGCAAAACGTCCACGAGTGGATAATCGAGAGTACCTGAAGAAAAAGCATCGGCTAACTCCGTGCCAGCAGCCGCGGTAATACGGAGGATGCGAGCGTTATCCGGATTTATTGGGTTTAAAGGGTGCGTAGGCGGCGACTTAAGTCAGCGGTAAAATTGCGGGGCTCAACCCCGTCGAGCCGTTGAAACTGGGGCGCTTGAGTTGGCGAGAAGTACGCGGAATGCGCGGTGTAGCGGTGAAATGCATAGATATCGCGCAGAACTCCGATTGCGAAGGCAGCGTACCGGCGCTCAACTGACGCTGAAGCACGAAAGCGTGGGGATCGAACAGG

>OTU793GTGGGGAATCTTGCGCAATGGGCGAAAGCCTGACGCAGCAACGCCGCGTGGGGGATGAAGGCCCTCGGGTTGTAAACCCCTTTCAGCAGGAACGAATCTGACGGTACCTGCAGAAGAAGGCCCGGCCAACTACGTGCCAGCAGCCGCGGTAATACGTAGGGGCCAAGCGTTGTCCGGATTTATTGGGCGTAAAGAGCTCGTAGGCGGTTGGGTAAGTCGGGTGTGAAAACTCCAGGCTCAACTTGGAGACGCCACCCGATACTGCTCTGACTTGAGTTCGGTAGGGGAGTGGGGAATTCCTGGTGTAGCGGTGAAATGCGCAGATATCAGGAGGAACACCGGTGGCGAAGGCGCCACTCTGGGCCGAAACTGACGCTGAGGAGCGAAAGCGTGGGTAGCAAACAGG

>OTU795GTAAGGGATATTGCACAATGGGCGCAAGCCTGATGCAGCAACGCCGCGTGCAGGAAGAAGGGTTTCGGCTCGTAAACTGCTTTTCTGCGGGACGAGGATGAAGGTACCGCAGGAAGAAGTCTCGGCTAACTACGTGCCAGCAGCCGCGGTAAAACGTAGGAGGCAAGCGTTATCCGGATTTACTGGGCGTAAAGGGCGTGCAGGTGGCTGTGTAAGTGGTGCGTGAAAGCGCCCGGCTCAACCGGGCGAGGCCGTGCCAAACTGCACAACTGGAAACAGGCAGAGGAAAGTGGAATTCGGGGTGTAGTGGTGAAATGCGTAGAGATCCCGAGGAACTCCTGTGGCGAAGGCGACTTTCTGGGCCTGCTTTGACACTCAGACGCGAAAGCATGGGGAGCGAACGGG

>OTU797GTGGGGAATATTGCGCAATGGGCGGAAGCCTGACGCAGCGACGCCGCGTGGGGGATGACGGCCTTCGGGTTGTAAACCTCTTTCGGCCCCGACGAAGCGTGAGTGACGGTAGGGGTGGAAGAAGCACCGGCCAACTACGTGCCAGCAGCCGCGGTAACACGTAGGGTGCGAGCGTTGTCCGGAATTATTGGGCGTAAAGAGCTCGTAGGCGGTGTGTCGCGTCGGCCGTGAAAACCTGCAGCTTAACTGTGGGCTTGCGGTCGATACGGGCATCACTGGAGTTCGGCAGGGGAGACTGGAATTCCTGGTGTAGCGGTGAAATGCGCAGATATCAGGAGGAACACCGGTGGCGAAGGCGGGTCTCTGGGCCGATACTGACGCTGAGGAGCGAAAGCGTGGGGAGCGAACAGG

>OTU798GTGAGGAATATTGGTCAATGGGCGGGAGCCTGAACCAGCCAAGCCGCGTGGGCGAAGAAGGCCCTATGGGTCGTAAAGTCCTTTTGTCAGGGAACAAAGGGCGGTACGTGTACCGCTGTGAGTGTACCTGAAGAAAAAGCATCGGCTAACTCCGTGCCAGCAGCCGCGGTAATACGGAGGATGCGAGCGTTATCCGGATTTATTGGGTTTAAAGGGTGCGCAGGCGGCGCGGTAAGTCAGCGGTAAAAGCCCGGGGCTCAACCCCGGCGAGCCGTTGAAACTGCCGTGCTAGAGTAAGGTCGAGGTATGCGGAATGCGCGGTGTAGCGGTGAAATGCATAGATATCGCGCAGAACTCCGATTGCGAAGGCAGCATACCGGTCCTTTACTGACGCTCAGGCACGAAAGCGTGGGGATCAAACAGG

>OTU800GTGGGGAATCTTGCGCAATGCGCGAAAGCGTGACGCAGCAACGCCGCGTGGAGGAAGACGGTCTTCGGATTGTAAACTCCTTTCAGTTGGGACGAAGCTCAACGGGTTAATAGCTCGGTGGAGTGACGGTACCTTCAGAAGAAGCCCCGGCTAACTACGTGCCAGCAGCCGCGGTAATACGTAGGGGGCTAGCGTTGTCCGGAATCATTGGGCGTAAAGAGCGTGTAGGCGGTCCGGTAAGTCCGCTGTGAAAGTCGGGGGCTCAACCCTCGAATGCCGGTGGATACTGTCGGGCTAGAGTGCGGAAGAGGCGAGTGGAATTCCTGGTGTAGCGGTGAAATGCGCAGATATCAGGAGGAACACCTATTGCGAAGGCAGCTCGCTGGGACGTAACTGACGCTGAGACGCGAAAGCGTGGGGAGCAAACAGG

>OTU802GTCGAGAGGCTTTGGCAATGGGGGAAACCCTGACCAAGCGACGCCGCGTGGAGGATGAAGGCCCTTGGGTTGTAAACTCCTTTTGTCGGGGAAGAAACGCGATCAGGTGAATAATCTGGTCGCTTGACGGTACCTGGCGAATAAGCTCCGGCTAACTCCGTGCCAGCAGCCGCGGTAACACGGGGGGAGCAAGCGTTGTTCGGAATCACTGGGCGTAAAGGGCGTGTAGGCGGTCAGGTAAGTGGAATGTGAAATCCCTCGGCTTACCCGAGGATCTGCATCCCAAACTGCTTGGCTTGAGTATGGGAGAGGATGAGGGAATTCCTGGTGTAGCGGTGAAATGCGTAGATATCAGGAGGAACACCGGTGGCGAAGGCGCTCATCTGGCCCAATACTGACGCTGAGGCGCGAAAGCTAGGGGAGCAAACGGG

>OTU803GTGAGGAATATTGGTCAATGGGCGCGAGCCTGAACCAGCCAAGTCGCGTGAGGGAGGACGGCCCTACGGGTTGTAAACCTCTTTTGCCGGGGAGCAACGGGCGTCACGTGTGGCGCCACTGAGAGTACCCGGAGAAAAAGCATCGGCTAACTCCGTGCCAGCAGCCGCGGTAATACGGAGGATGCGAGCGTTATCCGGATTTATTGGGTTTAAAGGGTGCGTAGGCGGATCGTTAAGTCAGTGGTCAAATTGAGGGGCTCAACCCCTTCCCGCCATTGAAACTGGCGTTCTTGAGTGGAAGAGAAGTATGCGGAATGCGTGGTGTAGCGGTGAAATGCATAGATATCACGCAGAACCCCGATTGCGAAGGCAGCATGCCGGCTTCCGACTGACGCTGAAGCACGAAAGCGTGGGGATCGAACAGG

>OTU804GTGAGGAATATTGGTCAATGGGCGCAGGCCTGAACCAGCCAAGTAGCGTGAAGGATGACTGCCCTATGGGTTGTAAACTTCTTTTATATGGGAATAAAGTTTTCCACGTGTGGAATTTTGTATGTACCATATGAATAAGGATCGGCTAACTCCGTGCCAGCAGCCGCGGTAATACGGAGGATCCGAGCGTTATCCGGATTTATTGGGTTTAAAGGGAGCGTAGGTGGACAGTTAAGTCAGTTGTGAAAGTTTGCGGCTCAACCGTAAAATTGCAGTTGATACTGGCTGTCTTGAGTACAGTAGAGGTGGGCGGAATTCGTGGTGTAGCGGTGAAATGCTTAGATATCACGAAGAACTCCGATTGCGAAGGCAGCTCACTGGACTGCAACTGACACTGATGCTCGAAAGTGTGGGTATCAAACAGG

>OTU805GTCGAGGATCTTCGGCAATGGGCGCAAGCCTGACCGAGCGACGCCGCGTGCGCGATGAAGGCCTTCGGGTTGTAAAGCGCTGTCGAGGGGGAGAAAAGTCCGCAAGGATCTGATCGACCCCTGGAGGAAGCACGGGCTAAGTTCGTGCCAGCAGCCGCGGTAAGACGAACCGTGCGAACGTTGTTCGGAATCACTGGGCTTAAAGGGCGCGTAGGCGGGCCATCAAGTCAGGGGTGAAATCCTCCGGCTCAACCGGAGAACGGCTTTTGATACTGGTGGTCTCGAGGGGGGTAGGGGCATGCGGAACTTCCGGTGGAGCGGTGAAATGCGTAGATATCGGAAGGAACGCCGGTGGCGAAAGCGGCGTGCTGGACCCTTTCTGACGCTGAGGCGCGAAAGCCAGGGGAGCAAACGGG

>OTU806GTAGGGAATATTGCACAATGGGCGAAAGCCTGATGCAGCAACGCCGCGTGAGGGATGAAGGTCTTCGGATTGTAAACCTCTTTTCTAAGGGACGAGAAGGGACGGTACCTTAGGAATAAGCAACGGCTAACTACGTGCCAGCAGCCGCGGTAATACGTAGGTTGCAAGCGTTATTCGGATTTACTGGGCGTAAAGCGCACCGAGGTGGCTTCTTAAGTTGGATGTAAAATCTCCCGGCTTAACTGGGAGGCGCCATTCAATACTGAGGAGCTTGAGGTCAGCAGGGGAAGGTGGAATTCCCGGTGTAGTGGTGGAATGCGTAGAGATCGGGAGGAACACCAGTGGCGAAAGCGACCTTCTGGGCTGCACCTGACACTCTAGTGCGAAAGCGTGGGGAGCAAACGGG

>OTU807GTAGGGAATATTGGTAATGGGCGAAAGCCTGAACCAGCAACGCCGCGTGTGCGAAGAAGGCCTTCGGGTTGTAAAGCACTTTTTAAGGGGATGAGGAAGGACAGTACCCTTAGAATAAGCCTCGGCTAACTACGTGCCAGCAGCCGCGGTAACACGTAGGAGGCAAGCGTTATTCGGATTTACTGGGCGTAAAGCGCGTGCAGGCGGTTTGGTAAGTTGGATGTGAAAGCTCCCGGCCTAACTGGGAGAGGTCGTTCAATACTACCAGACTAGAGGATGGGAGAGGGAGGTGGAATTCCGGGTGTAGTGGTGAAATGCGTAGATATCCGGAGGAACACCAGTGGCGAAAGCGGCCTCCTGGCCCATTTCTGACGCTCAGACGCGAAAGCTAGGGTAGCAAACGGG

>OTU808GTGGGGAATTGTTCACAATGGGCGCAAGCCTGATGACGCAACGCCGCGTGGAGGATGAAGGTCTTCGGATTGTAAACTCCTGTTGCTCGGGAAGAATAGCTCCGACCTAATACGTCGGAGTGTGACGGTACCGGGTGAGGAAGCCCCGGCTAACTCTGTGCCAGCAGCCGCGGTAATACAGAGGGGGCAAGCGTTGTTCGGAATTACTGGGCGTAAAGGGCGCGTAGGCGGCCTGTTAAGTCGAACGTGAAATCCCCGGGCTCAACCCGGGAACTGCGTCCGATACTGGCAGGCTTGAATCCGGGAGAGGGATGTGGAATTCCAGGTGTAGCGGTGAAATGCGTAGATATCTGGAGGAACACCGGTGGCGAAGGCGGCATCCTGGACCGGTATTGACGCTGAGGCGCGAAAGCCAGGGGAGCAAACGGG

>OTU809GTGGGGAATCATGGTCAATGGGCGAAAGCCTGAACCTGCGACGCCGCGTGAGTGATGAAGGTCGTAAGATCGTAAAACTCTTTTCAGGGATCTTAAAGCCGGTCCGTCAACAACGGTCCGGTGTGACTCTCCCTGGAATAAGCCCCAGCCAACTACGTGCCAGCAGCTGCGGTAATACGTAGGGGGCGAGCGTTATTCGGAATTACTGGGTGTAAAGGGTATGTAGGCGGTTTCTCAAGTCGCGTGTAAAAGTCCAGCGCTCAACGCTGGGTTCGCACGCGATACTGAGAAACTTGAGGGCTGGAGAGGTGAGCGGAATTCCCGGTGTAGTGGTAAAATACGTAGATATCGGGAGGAACACCAGCGGCGTAGGCGGCTCACTAGACAGTTTCTGACGCTAAGATACGAAAGCGTGGGGAGCAAACAGG

>OTU810GTGGGGAATCTTGCGCAATGGACGAAAGTCTGACGCAGCGACGCCGCGTGGGTGATGAAGGCCTTCGGGTTGTAAAGCCCTGTGGGGAGGGACGAATAAGTTCCGGTTAATACCCGGAGCGATGACGGTACCTCCTTAGCAAGCACCGGCTAACTCTGTGCCAGCAGCCGCGGTAAGACAGAGGGTGCAAACGTTGTTCGGAATTACTGGGCGTAAAGCGCGTGTAGGCGGCTATGCAAGTCGGATGTGAAAGCCCTCGGCTCAACCGGGGAAGTGCACCCGAAACTGCAAAGCTAGAGTCTCGGAGAGGATCGTGGAATTCTCGGTGTAGAGGTGAAATTCGTAGATATCGAGAGGAACACCGGTGGCGAAGGCGGCGATCTGGACGATGACTGACGCTGAGACGCGAAAGCGTGGGGAGCAAACAGG

>OTU811GTGGGGAATATTGGACAATGGGGGCAACCCTGATCCAGCCATGCCGCGTGAGTGAAGAAGGCCTTAGGGTTGTAAAGCTCTTTCGGCGGGGAAGATAATGACGGTACCCGCAGAAGAAGCCCCGGCTAACTCCGTGCCAGCAGCCGCGGTAATACGGAGGGGGCTAGCGTTGTTCGGAATTACTGGGCGTAAAGGGCACGTAGGCTGGCTGTTAAGTCGGGGGTGAAATCCTGAGGCTCAACCTCAGAATTGCCTCCGATACTGGTAGTCTTGAGTGCGGGAGAGGTGAGTGGAATTCCCAGTGTAGAGGTGAAATTCGTAGATATTGGGAAGAACACCAGTGGCGAAGGCGGCTCACTGGCCCGCAACTGACGCTGAGGTGCGAAAGCGTGGGGAGCAAACAGG

>OTU813GTGAGGAATTTTGCGCAATGGCCGCAAGGCTGACGCAGCAACGCCGCGTGGGTGATGAAGGCCTTCGGGTCGTAAAGCCCTGTCAGGTGGGAAGAAAGGCCTGGAGAGGAAAGGTTTCAGGTTTGACGGTACCACCAGAGGAAGCACCGGCTAACTCCGTGCCAGCAGCCGCGGTAATACGGAGGGTGCGAGCGTTATTCGGAATTACTGGGCGTAAAGCGCGTGTAGGCGGGGGGACAAGTCTGATGTGAAAGCCCTGGGCTTAACCTGGGAAGTGCATTGGAAACTGTTTTTCTTGAGTACTGGAGAGGAAGGGGGAATTCCCGGTGTAGAGGTGAAATTCGTAGAGATCGGGAGGAATACCAGTGGCGTAGGCGCCCTTCTGGACGGTAACTGACGCTGAGACGCGAAAGCGTGGGGAGCAAACAGG

>OTU817GTGAGGAATATTGGTCAATGGGCGGAAGCCTGAACCAGCCAAGTAGCGTGCAGGATGACGGCCCTACGGGTTGTAAACTGCTTTTTTGCGGGAATAAAGCGGCTCACGCGTGAGCCTTTGCATGTACCGCACGAATAAGGACCGGCTAATTCCGTGCCAGCAGCCGCGGTAATACGGAAGGTCCGGGCGTTATCCGGATTTATTGGGTTTAAAGGGAGCGTAGGCCGCCTTTTAAGCGTGCTGTGAAATACCGTTGCCCAACAACGGGGCTGCAGCGCGAACTGGAGGGCTTGAGTTCACGGGAAGCCGGCGGAATTCGTCGTGTAGCGGTGAAATGCTTAGATATGACGAAGAACTCCGATTGCGAAGGCAGCCGGCTGTAGTGTTACTGACGCTGAAGCTCGAAAGTGCGGGTATCGAACAGG

>OTU818GTGGGGAATTTTGCGCAATGGGCGAAAGCCTGATGACGCAACGCCGCGTGGGGGATGAAGGTCTTCGGATTGTAAACCCCTGTCGAATGGGACGAAAGACCCGGGAGCTAATCATCCCGGGAGTGACGGTACCGTTAAAGGAAGCCACGGCTAACTCTGTGCCAGCAGCCGCGGTAATACAGAGGTGGCAAGCGTTGTTCGGAATTACTGGGCGTAAAGGGCGCGTAGGCGGCCATCTAAGTCAGACGTGAAATCCCCCGGCTTAACCTGGGAACTGCGTCTGATACTGGACGGCTCGAGTTTGGGAGAGGGATGCAGAATTCCAGGTGTAGCGGTGAAATGCGTAGATATCTGGAGGAATACCGGTGGCGAAGGCGGCATCCTGGACTAATACTGACGCTGAGGCGCGAAAGCTAGGGGAGCAAACAGG

>OTU820GTGGGGAATATTGCGCAATGGAGGAAACTCTGACGCAGCAATGCCGCGTGAGTGAAGAAGGGATTCGTTCCGTAAAGCTCTGCCGTTGGGGAAGAAAGAAGACGGTACCCAACAAGCAAGCCCCGGCTAACTACGTGCCAGCAGCCGCGGTAACACGTAGGGGGCGAGCGTTGTCCGGAATTACTGGGCGTAAAGGGCACGCAGGCGGCTGTGCAGGCAGGATGTGAAAAGTCAGGGCCCAACCCTGAAGGTGCATCCTGAACCACGCAGCTAGAGTGCTGGAGAGGCAGGCAGAATTCCTGGTGTAGCGGTGAAATGCGCAGATATCAGGAGGAATACCAGAGGCGAAGGCGGCCTGCCGGACAGACACTGACGCTCAGGTGCGAAAGCGTGGGGAGCAAACAGG

>OTU823GTGGGGAATTTTGGACAATGGGCGCAAGCCTGATCCAGCCATGCCGCGTGCGGGAAGAAGGCCTTCGGGTTGTAAACCGCTTTTGTCAGGGAAGAAACGCGCTGGGCTAATACCTTGGCGTAATGACGGTACCTGAAGAATAAGCACCGGCTAACTACGTGCCAGCAGCCGCGGTAATACGTAGGGTGCGAGCGTTAATCGGAATTACTGGGCGTAAAGCGTGCGCAGGCGGCTTTGCAAGACAGATGTGAAATCCCCGGGCTTAACCTGGGAACTGCATTTGTGACTGCATGGCTAGAGTGCGGCAGAGGGGGATGGAATTCCGCGTGTAGCAGTGAAATGCGTAGATATGCGGAGGAACACCGATGGCGAAGGCAATCCCCTGGGCCTACACTGACGCTCATGCACGAAAGCGTGGGGAGCAAACAGG

>OTU825GTGGGGAATATTGCACAATGGGCGCAAGCCTGATGCAGCCATGCCGCGTGTGTGAAGAAGGCCTTCGGGTTGTAAAGCACTTTCAGTAAGGAGGAAAGGTTAGTAGTTAATACCTGCTAGCTGTGACGTTACTTACAGAAGAAGCACCGGCTAACTCCGTGCCAGCAGCCGCGGTAATACGGAGGGTGCGAGCGTTAATCGGAATTACTGGGCGTAAAGCGTACGCAGGCGGTTTGTTAAGCGAGATGTGAAAGCCCCGGGCTCAACCTGGGAACTGCATTTCGAACTGGCAAACTAGAGTGTGATAGAGGGTGGTAGAATTTCAGGTGTAGCGGTGAAATGCGTAGAGATCTGAAGGAATACCGATGGCGAAGGCAGCCACCTGGGTCAACACTGACGCTCATGTACGAAAGCGTGGGGAGCAAACGGG

>OTU827GTGGGGAATCTTGCGCAATGCGCGAAAGCGTGACGCAGCAACGCCGCGTGGGGGAAGAAGGCCTTCGGGTTGTAAACCCCTTTCAGGAGGGACGAAGGTTCGGCGGTGAATAGCCGACCGGACTGACGGTACCTCCACAAGAAGCCCCGGCTAACTACGTGCCAGCAGCCGCGGTAATACGTAGGGGGCTAGCGTTGTCCGGAATCATTGGGCGTAAAGCGCGCGTAGGCGGCCCGGTAAGTCCGCTCTGAAAGTCCAGGGCTCAACCCTGGAAAGCGGGTGGATACTGCCGGGCTAGAATCCGGAAGAGGCGAGTGGAATTCCTGGTGTAGCGGTGGAATGCGCAGATATCAGGAGGAACACCAATAGCGAAGGCAGCTCGCTGGGACGGTATTGACGCTGAGGCGCGAAAGCGTGGGGAGCAAACAGG

>OTU832GTCGGGAATCTTGCGCAATGGGCGAAAGCCTGACGCAGCAACACCGTGTGAGCGACGAAGGCCTTCGGGTCGTAAAGCTCTGTTGTTGGGGACGAAGGGTTAGGGGTTAATAGCCCCGAGCCTGACGGTACCCTTCGAGGAAGCCCCGGCTAACTACGTGCCAGCAGCCGCGGTAATACGTAGGGGGCGAGCGTTGTCCGGAATTATTGGGCGTAAAGAGCGTGTAGGCGGTTCGGTAAGTCTGCCGTGAAAACCCAGGGCTCAACCCTGGGCGTGCGGTGGATACTGCCGGGCTAGAGGGTGGTAGAGGCGAGTGGAATTCCCGGTGTAGCGGTGAAATGCGCAGATATCGGGAGGAACACCAGTAGCGAAGGCGGCTCGCTGGGCCACACCTGACGCTGAGACGCGAAAGCGTGGGGAGCAAACAGG

>OTU835ATGAGGAATATTGCGCAATGGGGGAAACCCTGACGCAGCGACGCCGCGTGGGTGAGGAAGTCCTTCGGGATGTAAAGCCCTTTTCTGGGGGAAGACAACCGACGGTACCCCAGGAAGAAGCCCCGGCTAACTACGTGCCAGCAGCCGCGGTAATACGTAGGGGGCAAGCGTTGTCCGGATTTACTGGGCGTAAAGCGCGTGTAGGCGGGAGAGGCAGTCCGGTGTGAAAGCCCTCGGCTCAACCGAGGAAGGTCGCTGGAAACTCCTCTTCTTGAGGGTGGCAGAGGGAAGTGGAATTCCCGGTGTAGCGGTGAAATGCGTAGATATCGGGAGGAACACCCGTGGCGAAGGCGGCTTCCTGGGCCACCACTGACGCTGAGACGCGAAAGCGTGGGGAGCGAACGGG

>OTU836GTGGGGAATATTGGACAATGGGGGGAACCCTGATCCAGCCATGCCGCGTGTGTGAAGAAGGCCTTTTGGTTGTAAAGCACTTTAAGTGGGGAGGAGGCTTACCTAGTTAATACCTGGGCTAAGTGGACGTTACCCACAGAATAAGCACCGGCTAACTCTGTGCCAGCAGCCGCGGTAATACAGAGGGTGCGAGCGTTAATCGGATTTACTGGGCGTAAAGCGCGCGTAGGTGGTTAATTAAGTCAAATGTGAAATCCCCGAGCTTAACTTGGGAATTGCATTCGATACTGGTTAGCTAGAGTATGGGAGAGGATGGTAGAATTCCAGGTGTAGCGGTGAAATGCGTAGAGATCTGGAGGAATACCGATGGCGAAGGCAGCCATCTGGCCTAATACTGACACTGAGGTGCGAAAGCATGGGGAGCAAACAGG

>OTU838GTGGGGAATCTTCCGCAATGGGCGAAAGCCTGACGGAGCAACGCCGCGTGAGTGAAGAAGGTCTTCGGATTGTAAAGCTCTGTTGTACATGACGAATGTGCCGGTTGTGAATAATGGCTGGCAATGACGGTAGTGTACGAGGAAGCCACGGCTAACTACGTGCCAGCAGCCGCGGTAATACGTAGGTGGCAAGCGTTGTCCGGAATTATTGGGCGTAAAGAGCATGTAGGCGGCCTATTAAGTCGGGCGTGAAAATGCGGGGCTCAACCCCGTATGGCGCCCGATACTGGTGGGCTTGAGTGCAGGAGAGGAAAGGGGAATTCCCAGTGTAGCGGTGAAATGCGTAGATATTGGGAGGAACACCAGTGGCGAAGGCGCCTTTCTGGACTGTGTCTGACGCTGAGATGCGAAAGCCAGGGGAGCGAACGGG

>OTU839GTAGGGAATCTTCCACAATGGACGAAAGTCTGATGGAGCAACGCCGCGTGAGTGATGAAGGTTTTCGGATCGTAAAACTCTGTTGTAAGGGAAGAACACGTACGAGAGGGAATGCTCGTACCTTGACGGTACCTTACGAGAAAGCCACGGCTAACTACGTGCCAGCAGCCGCGGTAATACGTAGGTGGCAAGCGTTGTCCGGAATTATTGGGCGTAAAGCGCGCGCAGGCGGCCTTTTAAGTCTGATGTGAAAGCCCCCGGCTCAACCGGGGAGGGCCATTGGAAACTGGAAGGCTTGAGTACAGAAGAGAAGAGTGGAATTCCACGTGTAGCGGTGAAATGCGTAGAGATGTGGAGGAACACCAGTGGCGAAGGCGACTCTTTGGTCTGTAACTGACGCTGAGGCGCGAAAGCGTGGGGAGCAAACAGG

>OTU841GTGGGGAATTTTGCGCAATGGGCGAAAGCCTGACGCAGCAATGCCGCGTGAGTGATGAAGGCCTTCGGGTCGTAAAGCTCTGTCAGAGGGGAAGAACCTCCTGTCGGTTAATACCCGGCAGGCCTGACGGTACCCTCAAAGGAAGCACCGGCTAACTCCGTGCCAGCAGCCGCGGTAATACGGAGGGTGCGAGCGTTGTTCGGAATTATTGGGCGTAAAGCGCGTGCAGGCGGCTCGCTAAGTCTGATGTGAAAGCCCCGGGCTCAACCCGGGAAGTGCATTGGAAACTGGCGAACTTGAGTACGGGAGAGGGAAGTGGAATTCCGAGTGTAGGGGTGAAATCCGTAGATATTCGGAGGAACACCAGTGGCGAAGGCGGCTTCCTGGACCGATACTGACGCTGAGACGCGAAAGCGTGGGGAGCAAACAGG

>OTU842GTGGGGAATATTGGACAATGGGGGCAACCCTGATCCAGCAATGCCGCGTGAGTGATGAAGGCCTTCGGGTTGTAAAGCTCTTTTGCCAGGGACGATGATGACGGTACCTGGAGAATAAGCCCCGGCTAACTTCGTGCCAGCAGCCGCGGTAATACGAAGGGGGCAAGCGTTGTTCGGAATTACTGGGCGTAAAGGGCGCGTAGGCGGCTTATCAAGTCAGGCGTGAAATTCCCGGGCTCAACCTGGGGGCTGCGCTTGATACTGATGAGCTTGAATGCGGGAGAGGATAGTGGAATTCCCAGTGTAGAGGTGAAATTCGTAGATATTGGGAAGAACACCGGTGGCGAAGGCGGCTATCTGGCCCGTAATTGACGCTGAGGCGCGAAAGCGTGGGGAGCAAACAGG

>OTU844GTGGGGGATATTGCACAATGGGGGGAACCCTGATGCAGCGACGCCGCGTGAGCGAAGAAGTATTTCGGTATGTAAAGCTCTATCAGCAGGGAAGAAGACGACGGTACCTGACTAAGAAGCTCCGGCTAAATACGTGCCAGCAGCCGCGGTAATACGTATGGAGCAAGCGTTATCCGGATTTACTGGGTGTAAAGGGAGCGTAGGCGGCCTGGCAAGTCTGGTGTGAAAGGCCGGGGCCCAACCCCGGGACTGCATTGGAAACTGCCAGGCTGGAGTGCCGGAGGGGCAGGCGGAATTCCTAGTGTAGCGGTGAAATGCGTAGATATTAGGAGGAACACCGGTGGCGAAGGCGGCCTGCTGGACGGCAACTGACGCTGAGGCTCGAAAGCGTGGGGAGCAAACAGG

>OTU845GTGGGGAATTTTGCGCAATGGGCTAACGCCTGACGCAGCAACGCCGCGTGGAGGATGAAGGTCTTTGGATTGTAAACTCCTGTCAGCAGGGAAAAAGGGACTGCGGTTAATACCCGTGGAACTTGATTGTACCTGCAGAGGAAGCCCCGGCTAACTCTGTGCCAGCAGCCGCGGTAATACAGAGGGGGCAAGCGTTATTCGGAATTACTGGGCGTAAAGCGCGCGTAGGCGGCTTTGCAAGTCGGGTGTGAAATCCCCAGGCTTAACCTGGGAACTGCATTCGAGACTGCATTGCTAGAGTATGGGAGAGGGAAGTGGAATTTCCGGTGTAGCGGTGAAATGCGTAGATATCGGAAGGAACATCAGTGGCGAAAGCGACTTCCTGGACCAATACTGACGCTCATGTGCGAAAGCGTGGGGAGCAAACAGG

>OTU846GTGGGGGATATTGCACAATGGGGGGAACCCTGATGCAGCGACGCCGCGTGGGTGAAGGAGCGTTTCGGCGCGTAAAGCCCTATCGGCAGGGAGGAAGATGACGGTACCTGGCTAAGAAGCCCCGGCTAACTACGTGCCAGCAGCCGCGGTAATACGTAGGGGGCGAGCGTTATCCGGATTTATTGGGTTTAAAGGGTGCGCAGGTGGCAGGGCAAGTCAGATGTGAAAGCCCGGGGCTCAACCCCGGTATTGCATTTGAAACTGTCCGGCTAGAGTGCAGGAGAGGTAAGCGGAATTCCTAGTGTAGCGGTGAAATGCGTAGATATTAGGAGGAACACCAGTGGCGAAGGCGGCTTGCTGGACGATGACTGACGTTGAGGCTCGAAAGCGTGGGGAGCAAACAGG

>OTU847GTAGGGAATTTTCGTCAATGGGGGAAACCCTGAACGAGCAATGCCGCGTGAGTGAAGAAGGTCTTCGGATCGTAAAGCTCTGTTGTAAGTGAAGAACGGCTCATAGAGGAAATGCTATGGGAGTGACGGTAGCTTACCAGAAAGCCACGGCTAACTACGTGCCAGCAGCCGCGGTAATACGTAGGTGGCAAGCGTTATCCGGAATCATTGGGCGTAAAGGGTGCGTAGGTGGCGTACTAAGTCTGTAGTAAAAGGCAATGGCTCAACCATTGTAAGCTATGGAAACTGGTATGCTGGAGTGCAGAAGAGGGCGATGGAATTCCATGTGTAGCGGTAAAATGCGTAGATATATGGAGGAACACCAGTGGCGAAGGCGGTCGCCTGGTCTGTAACTGACACTGAGGCACGAAAGCGTGGGGAGCAAATAGG

>OTU851GTGGGGAATTGTTCGCAATGGGCGCAAGCCTGACGACGCAACGCCGCGTGGAGGATGAAGGTCTTCGGATTGTAAACTCCTGTTGCTCGGGACGAAAAGCTTCGACCTAACACGTCGAGGTCTGACGGTACCGAGTGAGGAAGCCCCGGCTAACTCTGTGCCAGCAGCCGCGGTAATACAGAGGGGGCTAGCGTTGTTCGGAATTACTGGGCGTAAAGGGCGCGTAGGTGGCCTGTTAAGTCGAACGTGAAATCCCCGGGCTCAACCCGGGAACTGCGTCCGAGACTGGCAGGCTCGAATCCGGGAGAGGGATGTGGAATTCCAGGTGTAGCGGTGAAATGCGTAGATATCTGGAGGAACACCGGTGGCGAAGGCGGCATCCTGGACCGGTATTGACGCTGAGGCGCGAAAGCCAGGGGAGCAAACGGG

>OTU853GTGGGGAATATTGCACAATGGGGGAAACCCTGATGCAGCGACGCCGCGTGAGTGAAGAAGTATTTCGGTATGTAAAGCTCTATCAGCAGGGAAGAAAAAGAAATGACGGTACCTGATTAAGAAGCCCCGGCTAACTACGTGCCAGCAGCCGCGGTAATACGTAGGGGGCAAGCGTTATCCGGATTTACTGGGTGTAAAGGGAGCGTAGACGGCAGCGCAAGTCTGGAGTGAAATCCCGGGGCTCAACCCCGGAACTGCTTTGGAAACTGTGCAGCTGGAGTGCAGGAGAGGTAAGCGGAATTCCTAGTGTAGCGGTGAAATGCGTAGATATTAGGAGGAACACCGGTGGCGAAGGCGGCTTACTGGACTGTAACTGACGTTGAGGCTCGAAAGCGTGGGGAGCAAACAGG

>OTU856GTGGGGAATCTTGCGCAATGGGCGAAAGCCTGACGCAGCGACGCCGCGTGGGGGATGAAGCTTCTCGGAGTGTAAACCCCTTTCGACCGGGACGAATGCCCGCAAGGGAGTGACGGTACCGGTAGAAGAAGCCCCGGCTAACTACGTGCCAGCAGCCGCGGTAAGACGTAGGGGGCCAGCGTTGTTCGGAATTACTGGGTGTAAAGGGTTCGTAGGCGGTGTGGCAAGTCGGGAGTGAAATCTCTGGGCTTAACTCAGAGGCTGCTTCCGAAACTGCTGTGCTAGAGTGCGGGAGAGGCGCGTGGAATTGCAGGTGTAGCGGTGAAATGCGTAGATATCTGCAGGAACACCCGTGGCGAAAGCGGCGCGCTGGACCGTTACTGACGCTGAGGAACGAAAGCTAGGGGAGCAAACAGG

>OTU857GTGGAGAATCTTGCACAATGGGCGCAAGCCTGATGCAGCCACGCCGCGTGAGGGAAGAAGGCTTTCGAGTTGTAAACCTCTTTCAGCCGTGAAGAAGCGAGAGTGACGGTAACGGCAGAAGAAGCCCCGGCCAACTACGTGCCAGCAGCCGCGGTAATACGTAGGGGGCGAGCGTTATCCGGAATTATTGGGCGTAAAGCGCGTGTAGGCGGCCCTGTAAGTCGGATGTGAAATCTCTGGGCTCAACCCAGAAACTGCAGCCGATACTGGTGGCCTTGAGGTAGCTAGAGGAGAGTGGAATTCCCGGTGTAGCGGTGGAATGCGCAGATATCGGGAGGAACACCAATGGCGAAGGCAGCTCTCTGGAGCTCACCTGACGCTGAGACGCGAAAGCATGGGTAGCAAACAGG

>OTU858GTTGGGAATCTTGGACAATGGGGGAAACCCTGATCCAGCCATGCCGCGTGAGTGATGAAGGCCTTCGGGTTGTAAAGCTCTTTTACCCGGGAAGATAATGACTGTACCGGGAGAATAAGCTCCGGCTAACTTCGTGCCAGCAGCCGCGGTAATACGAAGGGGGCTAGCGTTGTTCGGAATTACTGGGCGTAAAGCGCGCGCAGGCGGTTTCTCAAGTCAGTGGTGAAAGCCCGGAGCTCAACTCCGGAACTGCCATTGAAACTGTGAAACTTGAGGACGAGAGAGGTGAGTGGAATTCCCAGTGTAGAGGTGAAATTCGTAGATATTGGGAAGAACACCGGTGGCGAAGGCGGCTCACTGGCTCGTTTCTGACGCTCAGGCGCGACAGCGTGGGGATCAAACAGG

>OTU859GTGGGGAATATTGCACAATGGGCGCAAGCCTGATGCAGCAACGCCGCGTGCGGGATGACGGCCTTCGGGTTGTAAACCGCTTTCAGCAGGGACGAAGCTAACGTGACGGTACCTGCAGAAGAAGCACCGGCCAACTACGTGCCAGCAGCCGCGGTGATACGTAGGGTGCGAGCGTTGTCCGGAATTATTGGGCGTAAAGAGCTTGTAGGCGGTTTGTCGCGTCGGAAGTGAAAACTCAGGGCTTAACTCTGAGCCTGCTTCCGATACGGGCAGACTGGAGGAAGGTAGGGGAGAACGGAATTCCTGGTGGAGCGGTGGAATGCGCAGATATCAGGAGGAACACCGGTGGCGAAGGCGGTTCTCTGGACCTTTCCTGACGCTGAGAAGCGAAAGCGTGGGGAGCAAACAGG

>OTU863GTGGGGAATATTGCACAATGGGGGAAACCCTGATGCAGCGACGCCGCGTGAGTGAAGAAGTATTTCGGTATGTAAAGCTCTATCAGCAGGGAAGATGATGACGGTACCTGAGTAAGAAGCCCCGGCTAACTACGTGCCAGCAGCCGCGGTAATACGTAGGGGGCAAGCGTTATCCGGATTTACTGGGTGTAAAGGGAGCGTAGACGGCATGGCAAGTCTGAAGTGAAAGCCCCTGGCTAAACTGGGGGAGGCGCGGTGAGACTGCTGGACTAGAGTACGGAAGAGGGATGCGGAATTCCGGGTGTAGTGGTGAAATGCGTAGAGATCCGGAGGAACACCAGAGGCGAAGGCGGCATCCTGGTTCGTGACTGACTCTGAGGCGCGAAAGCATGGGGAGCGAACGGG

>OTU865GTGGGGAATATTGCACAATGGGGGAAACCCTGATGCAGCGACGCCGCGTGAGTGAAGAAGTATTTCGGTACGTAAAGCTCTATCAGCAGGGAAGAAGAAGGACGGTACCTGAGTAAGAAGCCCCGGCTAACTACGTGCCAGCAGCCGCGGTAATACGTAGGGGGCAAGCGTTATCCGGATTTACTGGGTGTAAAGGGAGCGTAGACGGTGCGGCAAGTCTGGAGTGAAAGCCCGGGGCCCAACCCCGGGACTGCTTTGGAAACTGCCGAGCTAGAGTGCTGGAGAGGTAAGTGGAATTCCTAGTGTAGCGGTGAAATGCGTAGATATTAGGAGGAACACCAGTGGCGAAGGCGGCTTACTGGACAGTTACTGACGTTGAGGCTCGAAAGCATGGGTAGCGAACAGG

>OTU866GCTAAGGATATTCCGCAACGGGCGGAAGCCCGGCGGAGCGACGCCGCGTGGACGAGGAAGGCCGGAAGGTTGCAGAGTCCTTTTGCGGGGGAAGAAGGAGCCGCGGAGGGAATGCCGCGGCGGCGACCGAACCCCGCGAATAAGGGGCGGCTAATTACGTGCCAGCAGCCGCGGTAACACGTAAGCCCCGAGCGTTGTTCGGAATCATTGGGCGTAAAGGGCGCGTAGGCGGCCGCTCAAGCCCGGAGTGAAAGGCGGGGGCCCAACCCCCGGACTGCTCCGGGAACTGGGCGGCTGGAGTCGCCGAGGGGGAGCCGGAATTCCTGGTGTAGCGGTGAAATGCGTAGATATCAGGAGGAACACCGGCGGCGAAGGCGGCCTGCTGGACTGAGACTGACGTTGAGGCTCGAAAGCGTGGGGAGCAAACAGG

>OTU867GTGGGGAATATTGGACAATGGGCGCAAGCCTGATCCAGCAATGCCGCGTGGGTGAAGACGGCCTGCGGGTTGTAAAGCCCTTTAGGCGGGGAGGAACGACGGGACGCTAATAGCGTTTCGAAGTGACGTTACCCGCGGAATAAGCACCGGCTAACTCCGTGCCAGCAGCCGCGGTAATACGGAGGGTGCGAGCGTTAATCGGAATTACTGGGCGTAAAGCGTGCGTAGGCGGATGGGTCAGTCAGCCGTGAAAGCCCCGGGCTTAACCTGGGAACGGCGGTTGAGACGGCTCGTCTGGAGTGGGCTAGAGGATCGTGGAATTCCGGGTGTAGCGGTGAAATGCGTAGAGATCCGGAGGAACACCGATGGCGAAGGCAGCGGTCTGGGGCCACACTGACGCTGAGGCACGAAAGCGTGGGGAGCAAACAGG

>OTU868GTGAGGAATATTGGTCAATGGGCGCGAGCCTGAACCAGCCAAGTCGCGTGAGGGATGACGGTCCTATGGATTGTAAACCTCTTTTGTCAGGGAGCAAAGGACGTTACGTGTAGCGTTTCGAGAGTACCTGAAGAAAAAGCATCGGCTAACTCCGTGCCAGCAGCCGCGGTAATACGGAGGATGCGAGCGTTATCCGGATTTATTGGGTTTAAAGGGTGCGCAGGCGGAAAGTCAAGTCAGCGGTAAAATTGAGAGGCTCAACCTCTTCGAGCCGTTGAAACTGGTTTTCTTGAGTGAGCGAGAAGTATGCGGAATGCGTGGTGTAGCGGTGAAATGCATAGATATCACGCAGAACTCCGATTGCGAAGGCAGCATACCGGCGCTCAACTGACGCTCATGCACGAAAGCGTGGGTATCGAACAGG

>OTU874GTCGAGAATCTTGGGCAATGCGCGAAAGCGTGACCCAGCAATGCCGCGTGCGGGATGAAGGTCTTCGGATTGTAAACCGCTGTCACTCGGGACGAAAAAAATGACGGTACCGAGGGAGGAAGCCACGGCTAACTACGTGCCAGCAGCCGCGGTAATACGTAGGTGGCGAGCGTTGTTCGGATTTATTGGGCGTAAAGGGTCCGCAGGAGGTTTGTTAAAATTGGGGTGAAATCCCGGAGCTCAACTCCGGAACTGCCTTGATGACTGACGAACTAGAGTGCTGGAGAGGTAAGCGGAATACCAGGTGTAGCGGTGGAATGCGTAGATATCTGGTAGAACACCGAAAGCGAAGGCAGCTTACTGGACAGCAACTGACTCTCAGGGACGAAAGCGTGGGGAGCAAACAGG

>OTU878GTAGGGAATATTGGACAATGGGCGAGAGCCTGATCCAGCCATGCCGCGTGCAGGAAGACGGCCCTCTGGGTTGTAAACTGCTTTTATATGGGAAGAAAAGGGCTTTGCGAAGCACATTGACGGTACCATAGGAATAAGCCACGGCTAACTACGTGCCAGCAGCCGCGGTAATACGTAGGTGGCGAGCGTTATCCGGATTTATTGGGTTTAAAGGGTGCGTAGGCGGCTTTTTAAGTCGGTGGTTAAAGGTAGCAGCTCAACTGTTTTACATGCCATCGATACTGAAGAGCTTGAGTTATCAGAAGGCAGGCGGAATTTCTGGTGTAGCGGTGAAATGCATAGATACCAGAAGGAACACCTATTGCGAAGGCAGCTTGCTGCAGATAAACTGACGCTGATGCACGAAAGCGTGGGGAGCGAACAGG

>OTU880GTAGGGAATTTTCCGCAATGGGCGAAAGCCTGACGGAGCAACGCCGCGTGAGTGAGGACGGCCTTCGGGTTGTAAAACTCTGTTCTTGAGGAAGAATTCCTTCCAGGCGAACAGCCTGGAAGGTTGACGGTACTCAAGGAGAAAGCCCCGGCTAACTACGTGCCAGCAGCCGCGGTAATACGTAGGGGGCGAGCGTTATCCGGAATTATTGGGCGTAAAGCGCGCGCAGGCGGCCGATTAAGTCAGGTGTGAAAGGCTGCGGCTCAACCGCAGAGCGGCACCTGAAACTGGTCAGCTTGAGTGCAGGAGAGGGGAGCGGAATTCCCGGTGTAGCGGTGGAATGCGTAGAGATCGGGAGGAACACCAGTGGCGAAGGCGGCTCTCTGGCCTGTAACTGACGCTGAGGCGCGAAAGCGTGGGGAGCAAACAGG

>OTU881GTAAGGAATCTTCCACAATGGGGGCAACCCTGATGGAGCAACGCCGCGTGCAGGATGAAGGCCTTAGGGTCGTAAACTGCTTTTATGAGTGAAGAATATGACGGTAACTCATGAATAAGGGTCGGCTAACTACGTGCCAGCAGCCGCGGTCATACGTAGGACCCAAGCGTTATCCGGAGTGACTGGGCGTAAAGAGTTGCGTAGGTGGCTTGTAAAGTGAATAGTGAAATCCGGGGGCTCAACCTCACGGGCTATTATTCAAACTCACAAGCTCGAGAACAGCAGAGGTAACTGGAATTTCTGAAGTAGGGGTAATATCCGTAGATATCAGAAGGAACACCAATGGCGTAGGCAGGTTACTGGGCTGTTTCTGACACTGAGGCACGAAAGCGTGGGGAGCGAACCGG

>OTU885GTGGGGAATTTTGGGCAATGGCCGAAAGGCTGACCCAGCGACGCCGCGTGCGGGAGGACGCCCTTCGGGGTGTAAACCGCTGTCAGAGGGGACGAATGGCCCAGGCGGGAATACTGTCTGGGTGTGACGGTACCCTCAGAGGAAGCACCGGCTAACTCTGTGCCAGCAGCCGCGGTAATACGGAGGGTGCGAGCGTTGTCCGGAATCACTGGGCGTAAAGGGCGCGTAGGTGGTTCGATAAGTGTGTGGTGAAAGCCCGGGGCTCAACCCCGGGTCTGCCGTGCAAACTGTCGGACTGGAGGACTGCAGAGGCAGGTGGAATTCCGGGTGTAGCGGTGGAATGCGTAGATATCCGGAAGAAGACCGGTGGCGAAGGCGACCTGCTGGGCAGTTACTGACACTGAGGCGCGACAGCGTGGGGAGCAAACAGG

>OTU888GTGGGGAATCTTGCGCAATGGGCGAAAGCCTGACGCAGCAACGCCGCGTGGGGGATGAAGGCCTTCGGGTTGTAAACCCCTTTCAGCAGGGACGAAACTGACGGTACCTGCAGAAGAAGCCCCGGCCAACTACGTGCCAGCAGCCGCGGTAACACGTAGGGGGCAAGCGTTGTCCGGATTTATTGGGCGTAAAGAGCTCGTAGGCGGTTCGGTAAGTCGGGTGTGAAACCTCCAGGCTCAACCTGGAGACGCCACCCGATACTGCTGTGACTAGAGTCCGGTAGGGGAGCGTGGAATTCCTGGTGTAGCGGTGAAATGCGCAGATATCAGGAGGAACACCAGCGGCGAAGGCGGCGCTCTGGGCCGGAACTGACGCTGAGGAGCGAAAGCGTGGGTAGCAAACAGG

>OTU889GTAAGGAATATTGGTCAATGGACGCAAGTCTGAACCAGCCATGCCGCGTGGAGGATGAAGGCCCTCTGGGTTGTAAACTTCTTTTATGGGGGACGAAATCACTTATTCTTAAGTGTCTGACGGTACCCCAGGAATAAGCACCGGCTAACTCCGTGCCAGCAGCCGCGGTAATACGGAGGGTGCAAGCGTTATCCGGATTCACTGGGTTTAAAGGGTGCGTAGGAGGGCAGGAAAGTCAGTGGTGAAATCTCCGGGCTTAACTCGGAAACTGCCGTTGATACTACTTGTCTTGAATATCGTGGAGGTGAGCGGAATATGTCATGTAGCGGTGAAATGCTTAGATATGACATAGAACACCAATTGCGAAGGCAGCTCGCTACACGAATATTGACTCTGAGGCACGAAAGCGTGGGGATCAAACAGG

>OTU891GTAGGGAATATTGCATAATGGGCGAAAGCCTGATGCAGCAACGCCGCGTGTGCGATGAAGGCCTTCGGGTCGTAAAGCACTTTTGGAGGGGATGAGGAAGGACAGTACCCTCTGAATAAGCCTCGGCTAACTACGTGCCAGCAGCCGCGGTAAAACGTAGGAGGCGAGCGTTATCCGGATTTACTGGGCGTAAAGCGCGTGCAGGTGGTTCGGTAAGTTGGATGTGAAAGCTCCTGGCTTAACTGGGAGAGGTCGTTCAATACTACCGGACTTGAGAGTGGGAGAGGAAGGTGGAATTCCGGGTGTAGTGGTGAAATGCGTAGATATCCGGAGGAACACCAGTGGCGAAAGCGGCCTTCTGGCCCATTACTGACACTCATACGCGAAAGCTAGGGGAGCGAACGGG

>OTU893GTGAGGAATATTGGTCAATGGGCGAGAGCCTGAACCAGCCAAGTCGCGTGAGGGAAGACGGCCCTACGGGTTGTAAACCTCTTTTGTCAGGGAGCAAGAGCAGATACGTGTATCTGAGCGAGAGTACCTGAAGAAAAAGCATCGGCTAACTCCGTGCCAGCAGCCGCGGTAATACGGAGGATGCGAGCGTTATCCGGATTTATTGGGTTTAAAGGGTGCGTAGGCGGGCTGCCAAGTCAGCGGTAAAATTGCGGGGCTCAACCCCGTCGAGCCGTTGAAACTGGCGGTCTTGAGTGGGCGAGAAGTATGCGGAATGCGTGGTGTAGCGGTGAAATGCATAGATATCACGCAGAACTCCGATTGCGAAGGCAGCATACCGGCGCTCAACTGACGCTCATGCACGAAAGTGTGGGTATCGAACAGG

>OTU895GTCGGGAATTTTGGGCAATGGGCGAAAGCCTGACCCAGCAACGCCGCGTGAGGGATGAAGGCCTTCGGGTCGTAAACCTCTGTCGGGAGGGACGAAAGGTCGCCGAGCGAACAGGTCGGCGGCTTGACGGTACCTCTAAAGGAAGCGCCGGCTAACTCCGTGCCAGCAGCCGCGGTAATACGTAGGGGGCAAGCGTTGTCCGGATTTATTGGGCGTAAAGAGCGTGTAGGCGGCCAGACAGGTCCGTTGTGAAAACTGGATGCTTAACCTTCAGACGTCGATGGAAACCGTCTGGCTAGAGTCCGGAAGAGGAGAATGGAATTCCTGGTGTAGCGGTGAAATGCGCAGATATCAGGAAGAACACCCGTGGCTAAGGCGGTTCTCTAGTACGGTACTGACGCTGAGACGCGAAAGCGTGGGGAGCGAACAGG

>OTU896GTAAGGAATATTGGTCAATGGACGAAAGTCTGAACCAGCCATGCCGCGTGGAGGATGAAGGTCCTCTGGATTGTAAACTTCTTTTATCTGGGACGAAAAAAGACTTTTCTAAGTCGTCTGACGGTACCAGATGAATAAGCACCGGCTAACTCCGTGCCAGCAGCCGCGGTAATACGGAGGGTGCAAGCGTTATCCGGATTCACTGGGTTTAAAGGGTGCGCAGGCGGGCAATTAAGTCAGTGGTGAAATCCTGGAGCTTAACTCCAGAACTGCCATTGATACTATTTGTCTTGAATGTCGTGGAGGTCAGCGGAATATGTCATGTAGCGGTGAAATGCTTAGATATGACATAGAACACCGATTGCGAAGGCAGCTGACTACGCGAATATTGACGCTCATGCACGAAAGCGTGGGGATCAAACAGG

>OTU899GTGGGGGATATTGCACAATGGGGGGAACCCTGATGCAGCGATGCCGCGTGGAGGAAGAAGGTTTTCGGATTGTAAACTCCTGTCCTGAAGGACGATAATGACGGTACTTCAGGAGGAAGCTCCGGCTAACTATGTGCCAGCAGCCGCGGTAATACATAGGGAGCGAGCGTTATCCGGAATTACTGGGTGTAAAGGGAGCGTAGGCGGGACTGCAAGTCAGATGTGAAAACCACGGGCTCAACCTGTGGACTGCATTTGAAACTGTGGTTCTTGAGTGAAGTAGAGGCAGGCGGAATTCCTAGTGTAGCGGTGAAATGCGTAGATATTAGGAGGAACATCGGTGGCGAAGGCGGCTTGCTGGGCTTTTACTGACGCTGAGGCTCGAAAGCGTGGGGAGCAAACAGG

>OTU901GTGGGGAATCTTGCGCAATGGCCGAAAGGCTGACGCAGCGACGCCGCGTGTGGGAGGAAGCTCTTCGGGGTGTAAACCACTGTTGCCCGGGACGAACGAGCAGCTTTTGCTGCTGTGACGGTACCGGGTGAGGAAGCACCGGCTAACTCTGTGCCAGCAGCCGCGGTAATACAGAGGGTGCGAGCGTTGTCCGGAATCACTGGGCGTAAAGGGCGCGTAGGCGGCTTGGTAAGGTTGCGGTGAAAGCCCGGGGCTCAACCCCGGGTCGGCCGTGGCAACTGCTGAGCTGGAGCACTGTAGAGGCAGGTGGAATTCCGGGTGTAGCGGTGGAATGCGTAGAGATCCGGAAGAACACCGGTGGCGAAGGCGGCCTGCTGGGCAGTAGCTGACGCTGAGGCGCGACAGCGTGGGGAGCAAACAGG

>OTU910GTGGGGAATTTTGGACAATGGGGGCAACCCTGATCCAGCCATTCCGCGTGAGTGAAGAAGGCCTTCGGGTTGTAAAGCTCTTTCGGCAGGAACGAAACGGTGTGGGTTAATACCCTGCATCAATGACGGTACCTGAAGAAGAAGCACCGGCTAACTACGTGCCAGCAGCCGCGGTAATACGTAGGGTGCGAGCGTTAATCGGAATTACTGGGCGTAAAGCGTGCGCAGGCGGTTTTGTAAGACAGATGTGAAATCCCCGGGCTTAACCTGGGAACTGCACTTGTGACTGCACAGCTGGAGTACGGCAGAGGGGGATGGAATTCCGCGTGTAGCAGTGAAATGCGTAGATATGCGGAGGAACACCGATGGCGAAGGCAGTCCCCTGGGCCTGTACTGACGCTCATGCACGAAAGCGTGGGGAGCAAACAGG

>OTU911GTGGGGAATATTGGACAATGGGCGAAAGCCTGATCCAGCGACGCCGCGTGGGTGAAGAAGGCCTGCGGGTTGTAAAGCCCTTTCGGTGGGGAAGAAAAGCCTCGTTTTAATACAGCGGGGTCTTGACGTAACCCAAGAAAGAAGCACCGGCTAACTCTGTGCCAGCAGCCGCGGTAATACGTAGGGTGCGAGCGTTAATCGGAATTACTGGGCGTAAAGTGTGCGCAGGTGGCCTCGCAAGTCGAGTGTGAAATCCCCGGGCTTAACTTGGGAATTGCGCTCGAAACTACGGGGCTCGAGTGTGGCAGAGGGAGGTGGAATTCCACGTGTAGCGGTGAAATGCGTAGAGATGTGGAGGAACACCAATGGCGAAGGCAGCCTCCTGGGCCAACACTGACGCTCATGCACGAAAGCGTGGGGAGCAAACAGG

>OTU912GTAGGGAATATTGCGCAATGGACGAAAGTCTGACGCAGCCACGCCGCGTGAGTGAGGAAGGCCTTCGGGTTGTAAAGCTCTGTCATCCGGGAAAAACGGCTACTGGGGGAAAGTTCCAGTAGCGTGATGGTACCGGAGGAGGAAGCACCGGCAAACTCTGTGCCAGCAGCCGCGGTAATACAGAGGGTGCAAGCGTTAATCGGATTTACTGGGCGTAAAGCGTGCGTAGGCGGTTTGTTAAGTCGGATGTGAAATCCCTGGGCTCAACCTGGGAACTGCATTCGATACTGGCAGGCTAGAAAACGGTAGAGGGAGGCGGAACTCCAGGTGTAGCAGTGAAATGCGTAGATATCTGGAAGAACACCGATGGCGAAGGCAACCTCCTGGGCCTGTTTTGACGCTGAGGCACGAAAGCGTGGGGAGCAAACAGG

>OTU916GTGAGGAATATTGGTCAATGGGCGGGAGCCTGAACCAGCCAAGTCGCGTGAGGGAAGACGGTCCTATGGATTGTAAACCTCTTTTGCCGGGGAGCAACGGGGTCCTTGCGAGGGCCTAATGAGAGTACCCGGAGAAAAAGCATCGGCTAACTCCGTGCCAGCAGCCGCGGTAATACGGAGGATGCGAGCGTTATCCGGATTTATTGGGTTTAAAGGGTGCGTAGGCGGGCTGTTAAGTCAGCGGTCAAATGTCGGGGCTCAACCCCGGCCTGCCGTTGAAACTGGCGGCCTCGAGTGGGCGAGAAGTATGCGGAATGCGTGGTGTAGCGGTGAAATGCATAGATATCACGCAGAACTCCGATTGCGAAGGCAGTATACCGGCGCCCAACTGACGCTGAAGCACGAAAGCGTGGGTATCGAACAGG

>OTU919GTCGGGAATTTTGGGCAATGGGCGAAAGCCTGACCCAGCAACGCCGCGTGAAGGATGAAATCCCTCGGGATGTAAACTTCGCAAGAACGGGAAGAATAAGCCGGTGTTAACATCACCGGCGATGACGGTACCGTTTGTAAGCTCCGGCTAACTCCGTGCCAGCAGCCGCGGTAATACGGGGGGAGCAAGCGTTGTTCGGATTTACTGGGCGTAAAGGGCGCGTAGGCGGCCACCGCAAGTCGACTGTGAAATCTCCGGGCTTAACTCGGAAAGGTCAGCCGATACTGCGGGGCTAGAGTGCAGAAGGGGCAACTGGAATTCTCGGTGTAGCGGTGAAATGCGTAGATATCGAGAGGAACACCTGCGGCGAAGGCGGGTTGCTGGGCTGACACTGACGCTGAGGCGCGAAAGCTAGGGGAGCGAACGGG

>OTU924GTGGGGGATCTTGCGCAATGGGCGAAAGCCTGACGCAGCGACGCCGCGTGGGGGAAGAAGGCCTTCGGGTTGTAAACCTCTTTCAGCAGGGACGAAGCCACTCGGGTTAATAGCCCAGAGGGTGACGGTACCTGCAGAAGAAGCCCCGGCTAACTACGTGCCAGCAGCCGCGGTAATACGTAGGGACCAAGCGTTGTTCGGATTTACTGGGCGTAAAGGGCGCGTAGGCGGCATTGTTAGTCACTTGTGAAATCTCCGGGCTTAACTCGGAACGGCCAAGTGATACTGCAGTGCTAGAGTGCGGAAGGGGCAATCGGAATTCTTGGTGTAGCGGTGAAATGCGTAGATATCAAGAGGAACACCAGAGGTGAAGACGGGTTGCTGGGCCGACACTGACGCTGAGGCGCGAAAGCCAGGGGAGCAAACGGG

>OTU929GTCGAGAATCTTTCGCAATGGGCGAAAGCCTGACGAAGCGACGCCGTGTGAATGAAGAAGGCCCTTGGGTCGTAAAGTTCTTTCGCAAGGGAACAAGAGAAGGTAGCTAATATCAACCTGATTTGAGCGTACCTTGTAAAGAAGCACCGGCTAACTCCGTGCCAGCAGCTGCGGTAATACGGAGGGTGCGAGCATTAATCGGATTTATTGGGCGTAAAGGGGGCTAAGGCGGGAATATAAGTCAGTCGTGAAATCCCGGGGCTCAACCCCGGAACTGCGCTTGAAACTATATTTCTAGAGGCAAGACGGAGAAAACGGAATTCCACATGTAGCGGTGAAATGCGTAGATATGTGGAAGAACACCAGTGGCGAAGGCGGTTTTCTAGTTTTGTCCTGACGCTGATGCCCGAAAGCTAGGGGAGCGAACAGG

>OTU930GTGGGGAATATTGCACAATGGGGGAAACCCTGATGCAGCGACGCCGCGTGAGTGAAGAAGTATTTCGGTACGTAAAGCTCTATCAGCAGGGAAGAAAAAAAGTCCTTTGGACTTTGGACGGTACCTGACCAAGAAGCCCCGGCTAACTACGTGCCAGCAGCCGCGGTAATACGTAGGGGGCAAGCGTTATCCGGATTTACTGGGTGTAAAGGGAGCGTAGACGGCAGCGCAAGTCTGGAGTGAAATGCCGGGGCCCAACCCCGGAACTGCTTTGGAAACTGTGCAGCTCGAGTGCAGGAGAGGTAAGCGGAATTCCTAGTGTAGCGGTGAAATGCGTAGATATTAGGAGGAACACCAGTGGCGAAGGCGGCTTACTGGACTGTAACTGACGTTGAGGCTCGAAAGCGTGGGGAGCAAACAGG

>OTU931GTGGGGAATTTTGGACAATGGGCGCAAGCCTGATCCAGCAATGCCGCGTGAGTGAAGAAGGCCTTCGGGTTGTAAAGCTCTTTTGTCAGGGAAGAAACGGATTGGGTGAATACCCTGGTCTAATGACGGTACCTGAAGAATAAGCACCGGCTAACTACGTGCCAGCAGCCGCGGTAATACGTAGGGTGCAAGCGTTAATCGGAATTACTGGGCGTAAAGCGTGCGCAGGCGGTTATGTAAGACAGATGTGAAATGCCCGGGCTTAACCTGGGAACTGCATTTGTGACTGCATGGCTAGAGTTTGGCAGAGGGGGGTAGAATTCCACGTGTAGCAGTGAAATGCGTAGATATGTGGAGGAACACCGATGGCGAAGGCAGCCCCCTGGGTCAAGACTGACGCTCATGCACGAAAGCGTGGGGAGCAAACAGG

>OTU935GCTAAGAATATTCCGCAATGGGGGGAACCCTGACGGAGCGACGCCGCGTGGGCGAGGAAGGCCGGAAGGTTGTAAAGCCCTTTTATGCGCGGGGAATAAGCCGGGGAGGGAATGCCCCGGCGGTGACTGCGGCGCATGAATAAGCGCCGGCTAACTACGTGCCAGCAGCCGCGGTAACACGTAGGGCGCGAGCGTTGTTCGGAATCATTGGGCGTAAAGGGCGTGTAGGCGGCAGCGCAAGCGCGGCGTGAAAGGCCGGGGCCCAACCCCGGGAGTGCGCCGCGGACTGCGCAGCTGGAGCGGCCGGGGGGCAGCCGGAATTCCTGGTGTAGGGGTGAAATCTGTAGATATCAGGAGGAACACCGATGGCGAAGGCAGGCTGCCGGCGGACCGCTGACGCTGAGGCGCGAAGGCGCGGGGAGCGAACAGG

>OTU937GTAGGGAATATTGCACAATGGGCGCAAGCCTGATGCAGCAACGCCGCGTGCCCGATGAAGGCCTTCGGGTTGTAAAGGGCTTTTCTGGAGGAAGAGCAAGGACGGTACTCCAGGAAGAAGGATCGGCTAACTACGTGCCAGCAGCCGCGGTAAAACGTAGGATCCGAGCGTTATCCGAATTCACTGGGCGTAAAGCGCGTGCAGGCGGCGAGGTAAGTTGGGTGTGAAATCTCCCGGCTCAACTGGGAGAGGCCGCTCAAAACTGCCTGGCTCGAGGACGGTAGAGGAAGGTGGAATTCCCGGTGTAGTGGTGAAATGCGTAGATATCGGGAGGAACACCCGTGGCGAAAGCGGCCTTCTGGACCGTACCTGACGCTCAGACGCGAAAGCTAGGGGAGCAAACGGG

>OTU941GTGGGGAATTTTGGACAATGGGCGCAAGCCTGATCCAGCAATGCCGCGTGCGGGAAGAAGGCCTTCGGGTTGTAAACCGCTTTTGTACGGAACGAAAAGGCTCTGGCTAATACCTGGGGCTGATGACGGTACCGTAAGAATAAGCACCGGCTAACTACGTGCCAGCAGCCGCGGTAATACGTAGGGTGCGAGCGTTAATCGGAATTACTGGGCGTAAAGCGTGCGCAGGCGGTCTTGTAAGACAGAGGTGAAATCCCTGGGCTCAACCTAGGAATGGCCTTTGTGACTGCAAGGCTGGAGTGCGGCAGAGGGGGATGGAATTCCGCGTGTAGCAGTGAAATGCGTAGATATGCGGAGGAACACCGATGGCGAAGGCAGTCCCCTGGGCCTGCACTGACGCTCATGCACGAAAGCGTGGGGAGCAAACAGG

>OTU942GTAGGGAATCTTGGACAATGGGCGAAAGCCTGATCCAGCCATGCCGCGTGAGTGAAGAAGGCCTTAGGGTTGTAAAGCTCTTTTGGCGGGGACGATAATGACGGTACCCGCAGAATAAGCCCCGGCTAACTTCGTGCCAGCAGCCGCGGTAATACGAAGGGGGCTAGCGTTGTTCGGAATTACTGGGCGTAAAGCGCACGCAGGCGGATTGATAAGTCGGGGGTGAAATCCCGGGGCTCAACCTCGGAATTGCCTTCGATACTGTTAGTCTTGAGTCCGGGAGAGGTGAGTGGAATTCCTAGTGTAGAGGTGAAATTCGTAGATATTAGGAAGAAAACCAGTGGCGAAGGCGGCTCACTGGCCCGGTACTGACGCTCATGTGCGAAAGCGTGGGGAGCAAACAGG

>OTU946GTGGGGAATATTGCGCAATGGAGGCAACTCTGACGCAGCAATGCCGCGTGAGTGAAGAAGGGATTCGTTCTGTAAAGCTCTGTTATCGGGGAAGAGGTGACGGTACCCGATGAGCAAGCCCCAGCCAACTACGTGCCAGCAGCTGCGGTAATACGTAGGGGGCGAGCGTTATCCGGATTTATTGGGCGTAAAGGGCACGCAGGCGGCTGTTTAGGTGTGATGTGAAAGCCCGGGGCTTAACCCTGAGGTAGCATTGCAAACCGGGCAGCTAGAGTGAGGTAGAGGCAGACAGAATTTCTGGTGTAGCGGTGAAATGCGCAGAGATCAGGAGGAATACCGGAGGCGAAGGCGGTCTGCTGAGCCTTTGACTGACGCTCAGGTGCGAAAGCGTGGGGAGCGAACAGG

>OTU947GTGGGGGATATTGCACAATGGGGGGAACCCTGATGCAGCGACGCCGCGTGAGTGAAGGAGTACTTCGGTACGTAAAGCTCTATCAGCAGGGAAGAAAAGGGCGGGCTTGCCCGCCCGGACGGTACCTGACCAAGAAGCCCCGGCTAACTACGTGCCAGCAGCCGCGGTAATACGTAGGGGGCAAGCGTTATCCGGATTTACTGGGTGTAAAGGGAGCGTAGACGGCAAGGCAAGTCTGGAGTGAAAGCCCGGGGCCCAACCCCGGGACTGCTCTGGAAACTGTGGTGCTAGAGTGCAGGAGGGGTGGGCGGAATTCCTAGTGTAGCGGTGAAATGCGTAGATATTAGGAGGAACACCAGCGGCGAAGGCGGCCCACTGGACTGCGACTGACGTTGAGGCTCGAAGGCGTGGGGAGCAAACAGG

>OTU948GTGAGGAATATTGGTCAATGGGCGAGAGCCTGAACCAGCCAAGTAGCGTGCAGGACGACGGCCCTATGGGTTGTAAACTGCTTTTATAAGGGAATAAAGTGAGTCTCGTGAGACTTTTTGCATGTACCTTATGAATAAGGACCGGCTAATTCCGTGCCAGCAGCCGCGGTAATACGGAAGGTCCGGGCGTTATCCGGATTTATTGGGTTTAAAGGGAGCGTAGGCCGGAGATTAAGCGTGTTGTGAAATGTAGACGCTCAACGTCTGCACTGCAGCGCGAACTGGTTTCCTTGAGTACGCACAAAGTGGGCGGAATTCGTGGTGTAGCGGTGAAATGCTTAGATATCACGAAGAACTCCGATTGCGAAGGCAGCTCACTGGAGCGCAACTGACGCTGAAGCTCGAAAGTGCGGGTATCGAACAGG

>OTU956GTAAGGGATATTGCGACAATGGGCGAAAGCCTGACGCAGCAACGCCGCGTGCGGGATGACGGCCTTCGGGTTGTAAACCGCTTTTCAGGGGGAAGAGGAAGGACGGTACCCCTGGAATAAGTCTCGGCTAACTACGTGCCAGCAGCCGCGGTAAAACGTAGGAGGCGAGCGTTATCCGGATTTACTGGGTGTAAAGCGCGTGCAGGCGGTCTGGGAAGTGGTGCGTGAAAGCGCCCGGCTCAACCGGGCGAGGCCGTGCCAAACTGCCAGGCTTGAGGTAGGTAGAGGCGTGTGGAATTCCGGGTGTAGTGGTGAAATGCGTAGAGATCCGGAGGAACACCAGTGGCGAAGGCGACACGCTGGGCCTGACCTGACGCTCAGACGCGAAAGCATGGGGAGCGAACGGG

>OTU960GTGGGGAATATTGGACAATGGGCGCAAGCCTGATCCAGCCATGCCGCGTGAGTGACGAAGGCCTTAGGGTTGTAAAGCTCTTTTGGCGGGGAAGATAATGACGGTACCCGCAGAATAAGCTCCGGCTAACTTCGTGCCAGCAGCCGCGGTAATACGAAGGGAGCTAGCGTTGTTCGGAATCACTGGGCGTAAAGCGCACGTAGGCGGATGTGTCAGTCAGGGGTGAAATCCCGGAGCTCAACTTCGGAACTGCCCTTGATACAGCACGTCTCGAGTCCGAGAGAGGTGAGTGGAATTCCTAGTGTAGAGGTGAAATTCGTAGATATTAGGAAGAACACCGGTGGCGAAGGCGGCTCACTGGCTCGGTACTGACGCTGAGGTGCGAAAGCGTGGGGAGCAAACAGG

>OTU965GTGGGGGATATTGCACAATGGGGGAAACCCTGATGCAGCGACGCCGCGTGAGTGAAGAAGTATTTCGGTATGTAAAGCTCTATCAGCAGGGAAGAAAATGACAGTACCTGAGTAAGAAGCCCCGGCTAACTACGTGCCAGCAGCCGCGGTAATACGTAGGGGGCAAGCGTTATCCGGATTTACTGGGTGTAAAGGGAGCGTAGGTGGCATGACAAGTCAGAAGTGAAACCCTTGGGCTCAACCTGAGGCATGCTTTTGAAACTGTCAGGCTGGAGTGCTGGAGAGGTAAGCGGAATTCCTAGTGTAGCGGTGAAATGCGTAGATATTAGGAGGAACACCGGTGGCGAAGGCGGCTTACTGGACAGTCACTGACACTGAGGCTCGAAAGCGTGGGGAGCAAACAGG

>OTU967GTGGGGAATATTGCACAATGGGGGAAACCCTGATGCAGCCATGCCGCGTGTGTGAAGAAGGCCTTCGGGTTGTAAAGCACTTTCAGCGAGGAGGAAAGGTTGATGCCTAATACGTATCAACTGTGACGTTACTCGCAGAAGAAGCACCGGCTAACTCCGTGCCAGCAGCCGCGGTAATACGGAGGGTGCAAGCGTTAATCGGAATTACTGGGCGTAAAGCGCACGCAGGCGGTTGGATAAGTTAGATGTGAAAGCCCCGGGCTCAACCTGGGAATTGCATTTAAAACTGTCCAGCTAGAGTCTTGTAGAGGGGGGTAGAATTCCAGGTGTAGCGGTGAAATGCGTAGAGATCTGGAGGAATACCGGTGGCGAAGGCGGCCCCCTGGACAAAGACTGACGCTCAGGTGCGAAAGCGTGGGGAGCAAACAGG

>OTU968GTGGGGAATCTTGCGCAATGGGCGAAAGCCTGACGCAGCAACGCCGCGTGTGTGATGAAGGTCTTCGGATCGTAAAGCACTGTCGGGAGGGACGAATAAGGTGGCGGCTAACATCCGTCGCTGATGACGGTACCTCCAAAGGAAGCACCGGCTAACTCTGTGCCAGCAGCCGCGGTAATACAGAGGGTGCAAGCGTTGTTCGGAATTATTGGGCGTAAAGCGCGTGTAGGCGGCCAGGCAAGTTGGGTGTGAAAGCCCTCGGCTTAACCGAGGAAGTGCGCCCAAAACTACTTGGCTTGAGTACCGGAGAGGGTGGCGGAATTCCCGGTGTAGAGGTGAAATTCGTAGATATCGGGAGGAACACCAGTGGCGAAGGCGGCCACCTGGACGGATACTGACGCTGAGACGCGAAAGCGTGGGGAGCAAACAGG

>OTU970GTGGGGAATCTTCCGCAATGGGCGAAAGCCTGACGGAGCAACGCCGCGTGAACGATGAAGGTCTTAGGATCGTAAAGTTCTGTTGTTAGGGACGAAGGGTAAGAATCATAATACGGTTTTTATTTGACGGTACCTAACGAGGAAGCCACGGCTAACTACGTGCCAGCAGCCGCGGTAATACGTAGGCGGCAAGCGTTGTCCGGAATTATTGGGCGTAAAGGGAGCGCAGGCGGGAAACTAAGCGGATCTTAAAAGTGCGGGGCTCAACCCCGTGATGGGGTCCGAACTGGTTTTCTTGAGTGCAGGAGAGGAAAGCGGAATTCCCAGTGTAGCGGTGAAATGCGTAGATATTGGGAAGAACACCAGTGGCGAAGGCGGCTTTCTGGACTGTAACTGACGCTGAGGCTCGAAAGCTAGGGTAGCGAACGGG

>OTU971GTGGGGGATATTGGACAATGGGGGGAACCCTGATCCAGCGACGCCGCGTGAGTGAAGAAGTATCTCGGTATGTAAAGCTCTATCAGCAGGGAAGAAGGAAGTGACGGTACCTGACTAAGAAGCACCGGCTAAATACGTGCCAGCAGCCGCGGTAATACGTATGGTGCAAGCGTTATCCGGATTTACTGGGTGTAAAGGGAGCGCAGGCGGCGCGCTAAGTCCGATGTGAAAGCCCGGGGCTCAACCGCGGGACTGCATTGGAAACTGGCGGGCTGGAGTTCCGGAGAGGCAGGCGGAATTCCTAGTGTAGCGGTGAAATGCGTAGATATTAGGAGGAACACCAGTGGCGAAGGCGGTCTGCTGGACAGCAACTGACGCTGAGGCGCGAAAGCGTGGGGAGCAAACAGG

>OTU975GCTAGGAATCTTGGGAATGGGCGAAAGCCTGACCCAGCAACGCCGCGTGGGCGATGAAGGCCTTCGGGTCGTAAAGCCCTTTTGCAGGGGACGATGATGACGGTACCCTGCGAATAAGCCACGGCTAACTACGTGCCAGCAGCCGCGGTAATACGTAGGTGGCAAGCGTTGTCCGGATTTACTGGGCGTAAAGGGCGTGCAGGCGGTTTGTTAAGTTCAGGGTGAAAGCTCCCGGCTCAACTGGGAGAGGTCCTTGGATACTGGCAGACTTGAGGAGGGTAGAGGAGAGTGGAATTCCCGGTGTAGTGGTGATATGCGTAGATATCGGGAGGAACACCAGTGGCGAAGGCGGCTCTCTGGGCCCTACCTGACGCTGAGACGCGAAAGCGTGGGGAGCGAACCGG

>OTU976GTGGGGAATTTTGGACAATGGGCGCAAGCCTGATCCAGCCATGCCGCGTGTGTGAAGAAGGCCTTCGGGTTGTAAAGCACTTTCGGACGGAACGAAATCGCGCGGTCGAACATTCCGCGTGGATGACGGTACCGTAAGAAGAAGCACCGGCTAACTACGTGCCAGCAGCCGCGGTAATACGTAGGGTGCGAGCGTTAATCGGAATTACTGGGCGTAAAGGGTGCGCAGGCGGCCCCGCATGTCAGGCGTGAAATCCCCGGGCTCAACCTGGGAATGGCGCTTGAAACTACGGGGCTGGAGTGTGGCAGAGGGAGGTGGAATTCCACGTGTAGCGGTGAAATGCGTAGAGATGTGGAGGAACACCGATGGCGAAGGCAGCCTCCTGGGCCAACACTGACGCTCATGCACGAAAGCGTGGGGAGCAAACAGG

>OTU980GTGGGGAATATTGCACAATGGGGGAAACCCTGATGCAGCGACGCCGCGTGAGTGAAGAAGTATTTCGGTATGTAAAGCTCTATCAGCAGGAAAGAAATACTGACCTTACGGTCAGCAGACGGTACCTGACTAAGAAGCCCCGGCTAACTACGTGCCAGCAGCCGCGGTAATACGTAGGGGGCAAGCGTTATCCGGATTTACTGGGTGTAAAGGGAGCGTAGACGGCAGCGCAAGTCTGAAGTGAAATGCCGGGGCTTAACCCCGGAACTGCTTTGGAAACTGTGCAGCTAGAGTGCAGGAGAGGTAAGTGGAATTCCTAGTGTAGCGGTGAAATGCGTAGATATTAGGAGGAACACCAGTGGCGAAGGCGGCTTACTGGACTGTAACTGACGTTGAGGCTCGAAAGCGTGGGGAGCAAACAGG

>OTU981GTAGGGAATATTGCACAATGGGGGAAACCCTGATGCAGCGACGCCGCGTGAAGGAAGAAGTATCTCGGTATGTAAACTTCTATCAGCAGGGAAGACAATGACGGTACCTGACTAAGAAGCCCCGGCTAACTACGTGCCAGCAGCCGCGGTAATACGTAGGGGGCAAGCGTTATCCGGATTTACTGGGTGTAAAGGGAGCGTAGACGGATCTGCAAGTCTGGAGTGAAAGCCCGGGGCTCAACCCCGGGACTGCTTTGGAAACTGTGGATCTGGAGTGCCGGAGAGGTAAGCGGAATTCCTAGTGTAGCGGTGAAATGCGTAGATATTAGGAGGAACACCAGTGGCGAAGGCGGCTTACTGGACGGTAACTGACGTTGAGGCTCGAAAGCGTGGGGAGCAAACAGG

>OTU983GCGAGGAATATTGCACAATGGGCGCAAGCCTGATGCAGTAACGCCGCGTGCGGGATGAAGGCCTTCGGGTCGTAAACCGCTTTTCGCAGGGACGAGCAAGGACGGTACCTGTGGAATCAGTCTCGGCTAACTACGTGCCAGCAGCCGCGGTAAAACGTAGGAGGCGAGCGTTATCCGGATTCACTGGGCGTAAAGCGCATGTAGGTGGTTCGTTAAGTAGGTGGTGAAAGCCCCTGGCTCAACTGGGGGAGGTCCACCTAAACTGGCGGACTTGAGGTTCAGAGAGGAGAGTGGAATTCCCGGTGTAGCGGTGGAATGCGTAGAGATCGGGAGGAACACCAGTGGCGAAAGCGGCTCTCTGGCTGAATCCTGACACTGAGATGCGAAAGCGTGGGGAGCAAACGGG

>OTU985GTGAGGAATATTGGTCAATGGGCGAGAGCCTGAACCAGCCAAGTCGCGTGAGGGATGACGGTCTTACGGATTGTAAACCTCTTTTGTCAGGGAGCACAAGCGTCACGTGTGACGCGACGAGAGTACCTGAAGAAAAAGCATCGGCTAACTCCGTGCCAGCAGCCGCGGTAATACGGAGGATGCGAGCGTTATCCGGATTTATTGGGTTTAAAGGGTGCGTAGGCGGGCCCTTAAGTCAGCGGTAAAATCGCGTGGCTCAACCACGTCAAGCCGTTGAAACTGGGGGTCTTGAGTGAGCGAGAAGTATGCGGAATGCGTGGTGTAGCGGTGAAATGCATAGATATCACGCAGAACTCCGATTGCGAAGGCAGCATACCGGCGCTCAACTGACGCTGAAGCACGAAAGCGTGGGTATCGAACAGG

>OTU989GTGGGGAATATTGGGCAATGGGCGAAAGCCTGACCCAGCGACGCCGCGTGGGTGATGAAGGCCTTCGGGTTGTAAAGCCCTTTCGTGCGGAAAGAACAGCCCTGTAGTTAATACCTGCAGGGTTTGACGGTACCGCAGGAAGAAGCACCGGCTAACTCCGTGCCAGCAGCCGCGGTAATACGGAGGGTGCAAGCGTTGTTCGGAATTATTGGGCGTAAAGGGCGCGTAGGTGGTTGTGATAGTCAGATGTGAAATCCTTTGGCTTAACTGAAGAACTGCATCTGAAACTTCACAACTCGAGTACAGGAGAGGGAAACGGAATTCCCGGTGTAGAGGTGAAATTCGTAGATATCGGGAGGAACACCAGTGGCGAAGGCGGTTTCCTGGCCTGATACTGACACTGAGGCGCGAAAGCGTGGGGAGCAAACAGG

>OTU991GTAGGGAATCTTCCGCAATGGGCGAAAGCCTGACGGAGCAACGCCGCGTGAGTGAAGAAGGATTTCGGTTCGTAAAGCTCTGTTGTTAGGGAAGAATGATTGTGTAGTAACTATACACAGTAGAGACGGTACCTAACCAGAAAGCCACGGCTAACTACGTGCCAGCAGCCGCGGTAATACGTAGGTGGCAAGCGTTGTCCGGAATTATTGGGCGTAAAGCGCGCGCAGGTGGTTTAATAAGTCTGATGTGAAAGCCCACGGCTCAACCGTGGAGGGTCATTGGAAACTGTTAAACTTGAGTGCAGGAGAGAAAAGTGGAATTCCTAGTGTAGCGGTGAAATGCGTAGAGATTAGGAGGAACACCAGTGGCGAAGGCGGCTTTTTGGCCTGTAACTGACACTGAGGCGCGAAAGCGTGGGGAGCAAACAGG

>OTU992GTGGGGAATATTGCGCAATGGGCGAAAGCCTGACGCAGCAACGCCGCGTGGGGGATGAAGGTTCTCGGATTGTAAACCCCTTTCGATCGGAACGAATCTCCTCCTGGTAAATAATCAGGAGGATCGACGGTACCGAGAGAAGAAGCCACGGCTAACTCTGTGCCAGCAGCCGCGGTAAGACAGAGGTGGCGAGCGTTGTTCGGATTTATTGGGCGTAAAGCGAACGCAGGTGGTTTGGTAAGTCAGGGGTGAAAGTCCACAGCTTAACTGTGGCACTGCCTTTGATACTACCTTTCTTGAGTGCATGAGGGGGAAGCGGAATTCCGAGTGTAGAGGTGAAATTCGTAGATATTCGGAGGAACACCGGTGGCGAAGGCGGCTTCCTGGCATGTAACTGACACTCATGTTCGAAAGCGTGGGGAGCAAACAGG

>OTU994GTGGGGAATATTGGACAATGGGCGAAAGCCTGATCCAGCCATGCCGCGTGAGTGATGAAGGCCCTAGGGTTGTAAAGCTCTTTCACCGGTGAAGATAATGACGGTAACCGGAGAAGAAGCCCCGGCTAACTTCGTGCCAGCAGCCGCGGTAATACGAAGGGGGCTAGCGTTGTTCGGAATTACTGGGCGTAAAGCGCACGTAGGCGGATACTTAAGTTAGGGGTGAAATCCCAGGGCTCAACCCTGGAACTGCCTTTAATACTGGGTATCTCGAGTCCGGAAGAGGTGAGTGGAATTCCGAGTGTAGAGGTGAAATTCGTAGATATTCGGAGGAACACCAGTGGCGAAGGCGGCTCACTGGTCCGGTACTGACGCTGAGGTGCGAAAGCGTGGGGAGCAAACAGG

>OTU998GTGGGGAATATTGCGCAATGGGCGAAAGCCTGACGCAGCGACGCCGCGTGGGTGATGAAGGCCTTCGGGTCGTAAAGCCCTGTCGCAGGGGAAGAACAAGCAGCGGGATAATACCCCGCTGCCCTGACGGTACCCTGCAAGAAAGCACCGGCTAACTCCGTGCCAGCAGCCGCGGTAATACGGAGGGTGCGAGCGTTGTTCGGAATTACTGGGCGTAAAGCGCGTGTAGGCGGCCGATTAAGTCTGGTGTGAAAGCCCGGGGCTCACCCCCGGAAGTGCGCTGGAAACTGGTCGGCTAGAGTATGGGAGAGGAAAGTGGAATTCCCGGTGTAGCGGTGAAATGCGTAGATATCGGGAGGAACACCAGCGGCGAAGGCGGCTTTCTGGACCAATACTGACGCTGAGACGCGAAAGCGTGGGGAGCAAACAGG

>OTU1002GTGGGGAATATTGGACAATGGGGGGAACCCTGATCCAGCCATGCCGCGTGTGTGAAGAAGGCCTTATGGTTGTAAAGCACTTTAAGCGAGGAGGAGGCTACTAGTATTAATACTACTGGATAGTGGACGTTACTCGCAGAATAAGCACCGGCTAACTCTGTGACAGCAGCCGCGGTAATACAGAGGGTGCGAGCGTTAATCGGATTTACTGGGCGTAAAGCGTGCGTAGGCGGCCATTTAAGTCAAATGTGAAATCCCCGAGCTTAACTTGGGAATTGCATTCGATACTGGATGGCTAGAGTATGGGAGAGGATGGTAGAATTCCAGGTGTAGCGGTGAAATGCGTAGAGATCTGGAGGAATACCGATGGCGAAGGCAGCCATCTGGCCTAATACTGACGCTGAGGTACGAAAGCATGGGGAGCAAACAGG

>OTU1006GTGAGGAATATTGGTCAATGGTCGCGAGACTGAACCAGCCAAGTAGCGTGCAGGATGACGGCCCTCTGGGTTGTAAACTGCTTTTAGTTGGGAATAAAAGGCGGTACGTGTACCGCGTATTGTATGTACCATCAGAAAAAGGGCCGGCTAATTCCGTGCCAGCAGCCGCGGTAATACGGAAGGTCCAGGCGTTATCCGGATTTATTGGGTTTAAAGGGAGCGTAGGCGGATTTTTAAGTCAGTTGTGAAAGTTTGAGGCTCAACCTTAAAATTGCAGTTGAAACTGGAAGTCTTGAGTGCACGCAGAGGTGCCGGAATTCATGGTGTAGCGGTGAAATGCTTAGATATCATGAAGAACTCCGATCGCGAAGGCAAGTGCCTGGAGTGCTACTGACGCTGAGGCTCGAAAGTGCGGGTATCAAACAGG

>OTU1010GTGGGGAATATTGCACAATGGGGGAAACCCTGATGCAGCGACGCCGCGTGAGTGAAGAAGTATTTCGGTATGTAAAGCTCTATCAGCAGGGAAGATAATGACGGTACCTGACTAAGAAGCCCCGGCTAACTACGTGCCAGCAGCCGCGGTAATACGTAGGGGGCAAGCGTTATCCGGATTTACTGGGTGTAAAGGGAGCGTAGACGGCAGCGCAAGTCTGGAGTGAAATGCCGGGGCCCAACCCCGGAACTGCTCTGGAAACTGTGAAGCTGGAGTGCAGGAGAGGTAAGCGGAATTCCTGGTGTAGCGGTGAAATGCGTAGATATCAGGAGGAACACCAGTGGCGAAGGCGGCTTACTGGACTGTAACTGACGTTGAGGCTCGAAAGCGTGGGGAGCAAACAGG

>OTU1013GTAGGGAATCTTCCACAATGGACGCAAGTCTGATGGAGCAACGCCGCGTGAGTGAAGAAGGTTTTCGGATCGTAAAGCTCTGTTGTTGGTGAAGAAGGACATGGGTAGTAACTGATCTATGTTTGACGGTAATCAACCAGAAAGTCACGGCTAACTACGTGCCAGCAGCCGCGGTAATACGTAGGTGGCAAGCGTTGTCCGGATTTATTGGGCGTAAAGCGAACGCAGGCGGGAGAACAAGTCAGCTGTGAAAGCCCTCGGCTTAACCGAGGAACGGCAACTGAAACTGTTTTTCTTGAGTGCAGAAGAGGAGAGTGGAACTCCATGTGTAGCGGTGAAATGCGTAGATATATGGAAGAACACCGGTGGCGAAGGCGGCGACCTGGTTCACAACTGACGCTGAGGCGCGAAAGCGTGGGGAGCAAACAGG

>OTU1016GTGGGGAATATTGGACAATGGGCGAAAGCCTGATCCAGCCATGCCGCGTGAGTGATGAAGGCCTTAGGGTTGTAAAGCTCTTTCAGTGGGGAAGATAATGACGGTACCCACAGAAGAAGCCCCGGCTAACTTCGTGCCAGCAGCCGCGGTAATACGAAGGGGGCTAGCGTTGTTCGGATTTACTGGGCGTAAAGCGCACGTAGGCGGATCGTTAAGTCGGGGGTGAAATCCTGGAGCTCAACTCCAGAACTGCCTTCGATACTGGCGATCTTGAGTCCGGAAGAGGTGAGTGGAACTCCTAGTGTAGAGGTGGAATTCGTAGATATTAGGAAGAACACCAGTGGCGAAGGCGGCTCACTGGTCCGGTACTGACGCTGAGGTGCGAAAGCGTGGGGAGCAAACAGG

>OTU1020GTGGGGAATATTGGACAATGGGGGCAACCCTGATCCAGCAATGCCGCGTGTGTGAAGAAGGCCTGCGGGTTGTAAAGCACTTTCAGAGGGGAAGAAAAAATCGGAGCTAACACCTCCGAGCTTGACGTTACCCTTAGAAGAAGCACCGGCTAACTCCGTGCCAGCAGCCGCGGTAATACGGAGGGTGCAAGCGTTAATCGGAATTACTGGGCGTAAAGCGCACGCAGGTGGCTGAGTCAGTCGATTGTGAAAGCCCTGGGCTTAACCTGGGAATTGCAGTCGATACTACTCAGCTGGAGTATGGGAGAGGGTAGTGGAATTCCCGGTGTAGCGGTGAAATGCGTAGATATCGGGAGGAACATCAGTGGCGAAGGCGGCTACCTGGCCCAATACTGACACTCAGGTGCGACAGCGTGGGGAGCAAACAGG

>OTU1022GTGGGGAATATTGGACAATGGGCGCAAGCCTGATCCAGCCATGCCGCGTGTGTGAAGAAGGCCTTCGGGTTGTAAAGCACTTTCAGTGGGGAGGAAGGGGTTGTAGTTAATAGCTGCAATTTTTGACGTTACCCACAGAAGAAGCACCGGCTAACTCCGTGCCAGCAGCCGCGGTAATACGGAGGGTGCAAGCGTTAATCGGAATTACTGGGCGTAAAGCGCACGCAGGCGGCTTTTTAAGTCGGATGTGAAAGCCCCGGGCTCAACCTGGGAATTGCATCTGATACTGGGAAGCTAGAGTATGTGAGAGGGGGGTAGAATTCCAAGTGTAGCGGTGAAATGCGTAGAGATTTGGAGGAATACCAGTGGCGAAGGCGGCCCCCTGGCACAATACTGACGCTCAGGTGCGAAAGCGTGGGGAGCAAACAGG

>OTU1023GTGAGGAATATTGGTCAATGGGCGGGAGCCTGAACCAGCCAAGTCGCGTGAGGGATGACGGTCCTACGGATTGTAAACCTCTTTTGCCGGGGAGCAAGCGTGCGTTCGTGAACGCGCGTCGAGAGTACCCGGAGAAAAAGCATCGGCTAACTCCGTGCCAGCAGCCGCGGTAATACGGAGGATGCGAGCGTTATCCGGATTTATTGGGTTTAAAGGGTGCGTAGGCGGGATGCCAAGTCAGCGGTAAAAATGCGGTGCTCAACGCCGTCGAGCCGTTGAAACTGGCGTTCTTGAGTGGGCGAGAAGTATGCGGAATGCGTGGTGTAGCGGTGAAATGCATAGATATCACGCAGAACTCCGATTGCGAAGGCAGCATACCGGCGCCCTACTGACGCTGAGGCACGAAAGCGTGGGTATCGAACAGG

>OTU1026GTGAGGAATATTGGTCAATGGGCGGAAGCCTGAACCAGCCAAGTAGCGTGAGGGAAGACTGCCCTATGGGTTGTAAACCTCTTTTATGCGGGGATAAAGGTGTCCACGTGTGGATGTTTGCAGGTACCGCATGAATAAGGACCGGCTAATTCCGTGCCAGCAGCCGCGGTAATACGGAAGGTCCGGGCGTTATCCGGATTTATTGGGTTTAAAGGGAGCGTAGGCCGCATGTCAAGCGTGCTGTGAAATGTCCGGGCTCAACCCGGGCCTTGCAGCGCGAACTGGCGTGCTTGAGTCCGCGGAAAGCAGGCGGAATTCGTCGTGTAGCGGTGAAATGCTTAGATATGACGAAGAACCCCGATTGCGAAGGCAGCTTGCTGCAGCGGAACTGACGCTGATGCTCGAAAGCGCGGGTATCGAACAGG

>OTU1028GTGGGGAATATTGGGCAATGGGGGGAACCCTGACCCAGCAACGCCGCGTGAAGGAAGAAGGTTTTCGGATTGTAAACTTCTGTCCTTGGTGAAGAGGAAGAGACGGTAGCCAAGGAGGAAGCCCCGGCTAACTACGTGCCAGCAGCCGCGGTAATACGTAGGGGGCGAGCGTTGTCCGGAATGATTGGGCGTAAAGGGCGCGTAGGCGGTCATTTAAGTCTGGAGTGGAAGTCCACTTTTCAAGGGTGGAATTGCTTTGGATACTGGGTGGCTTGAGTGCAGGAGAGGTTATCGGAATTCCCGGTGTAGCGGTGAAATGCGTAGATATCGGGAGGAACACCAGTGGCGAAGGCGGGTAACTGGACTGCAACTGACGCTGAGGCGCGAAAGTGTGGGGAGCAAACAGG

>OTU1029GTAGGGAATTTTGCGCAATGGGCGAAAGCCTGACGCAGCAACGCCGCGTGATTGATTAAGCCCTTCGGGGTGTAAAGATCTGTCAGTGGGGACGAAACTTGACGGTACCCACAGAGGAAGCACCGGCTAACTCCGTGCCAGCAGCCGCGGTAATACGGAAGGTCCGGGCGTTATCCGGATTTATTGGGTTTAAAGGGAGCGTAGGCGGGCCGTTAAGTCAGTTGTGAAAGTTCGTGGCTCAACCCCGGAATTGCAGTTGAAACTGCCGGTCTTGAGTACACGCAGGGAAGCCTGAATTCATGGTGTAGCGGTGAAATGCTTAGATATCATGAAGACGACCGATCGCGAAGGCAGGTTTCCGGAGTGTAACTGACGCTGAGGCTCGAAAGTGCGGGTATCAAACAGG

>OTU1030GTGGGGAATATTGCACAATGGGCGAAAGCCTGATGCAGCAACGCCGCGTGAGCGATGAAGGCCTTCGGGTCGTAAAGCTCTGTCCTCAAGGAAGATAATGACGGTACTTGAGGAGGAAGCCCCGGCTAACTACGTGCCAGCAGCCGCGGTAATACGTAGGGGGCTAGCGTTATCCGGATTTACTGGGCGTAAAGGGTGCGTAGGCGGTCTTTCAAGTCAGGAGTGAAAGGCTACGGCTCAACCGTAGTAAGCTCTTGAAACTGGGAGACTTGAGTGCAGGAGAGGAGAGTGGAATTCCTAGTGTAGCGGTGAAATGCGTAGATATTAGGAGGAACACCAGTAGCGAAGGCGGCTCTCTGGACTGTAACTGACGCTGAGGCACGAAAGCGTGGGGAGCGAACAGG

>OTU1033GTGAGGAATATTGGTCAATGGTCGGGAGACTGAACCAGCCAAGCCGCGTGAGGGATGAAGGTGCAGAGCATCGTAAACCTCTTTTGTCAGGGAACAAAAAGGGCCACGTGTGGCCCGCTGAGTGTACCTGAAGAAAAAGCATCGGCTAACTCCGTGCCAGCAGCCGCGGTAATACGGAGGATGCGAGCGTTATCCGGATTTATTGGGTTTAAAGGGTGCGTAGGCGGCCTGTTAAGTCAGCGGTAAAAGCCCGGGGCTCAACCCCGGCACGCCGTTGAAACTGGCGGGCTCGAGTTGGAGAGAAGTATGCGGAATGCGCGGTGTAGCGGTGAAATGCATAGATATCACGCAGAACTCCGATTGCGAAGGCAGCATACCGGCGCTCAACTGACGCTCATGCACGAAAGCGTGGGTATCGAACAGG

>OTU1052GTCGGGAATTTTGGGCAATGGGCGAAAGCCTGACCCAGCAACGCCGCGTGAAGGATGAAATCCCTCGGGATGTAAACTTCGCAAGAACAGGAAGAATAAGTAGGGGACAATACCCCCTATGATGACGGTACTGTTTGTAAGCTCCGGCTAACTCCGTGCCAGCAGCCGCGGTAATACGGGGGGAGCAAGCGTTGTTCGGATTTACTGGGCGTAAAGGGCGCGTAGGCGGCGCCGCAAGTCGGTTGTGAAATCTCTGGGCTTAACCCAGAAAGGTCAACCGATACTGCGGGGCTAGAGTGCAGAAGGGGCAACTGGAATTCTCGGTGTAGCGGTGAAATGCGTAGATATCGAGAGGAACACCTGCGGCGAAGGCGGGTTGCTGGGCTGACACTGACGCTGATGCGCGAAAGCTAGGGGAGCGAACGGG

>OTU1059GTGGGGAATATTGCACAATGGGGGAAACCCTGATGCAGCGACGCCGCGTGAGTGAAGAAGTAATTCGTTATGTAAAGCTCTATCAGCAGGGAAGAAACAGACGGTACCTGAGTAAGAAGCCCCGGCTAACTACGTGCCAGCAGCCGCGGTAATACGTAGGGGGCAAGCGTTATCCGGATTTACTGGGTGTAAAGGGAGCGTAGACGGCGAAGAAAGTCTGAAGTGAAAGCCCGCGGCTTAACCGCGGAACTGCTTTGGAAACTTTTTTGCTGGAGTACCGGAGAGGTAAGCGGAATTCCTAGTGTAGCGGTGAAATGCGTAGATATTAGGAGGAACACCAGTGGCGAAGGCGGCTTACTGGACGGTAACTGACGTTGAGGCTCGAAAGCGTGGGGAGCAAACAGG

>OTU1065GTGGGGAATCTTCCGCAATGGACGAAAGTCTGACGGAGCAACGCCGCGTGAACGATGACGGCCTTCGGGTTGTAAAGTTCTGTTATACGGGACGAATGGTACGACGGTCAATACCCGTCGTAAGTGACGGTACCGTAAGAGAAAGCCACGGCTAACTACGTGCCAGCAGCCGCGGTAATACGTAGGTGGCAAGCGTTGTCCGGAATTATTGGGCGTAAAGGGCGCGCAGGCGGCGTCGTAAGTCGGTCTTAAAAGTGCGGGGCTTAACCCCGTGAGGGGACCGAAACTGCGATGCTAGAGTATCGGAGAGGAAAGCGGAATTCCTAGTGTAGCGGTGAAATGCGTAGATATTAGGAGGAACACCAGTGGCGAAAGCGGCTTTCTGGACGACAACTGACGCTGAGGCGCGAAAGCCAGGGGAGCAAACGGG

>OTU1066GTGAGGAATATTGGTCAATGGACGCAAGTCTGAACCAGCCACGTCGCGTGAAGGAAGACGGCCCTACGGGTTGTAAACTTCTTTTGTAAGGGAATAAAGTGTAGTACGTGTACTATTTTGCATGTACCTTACGAATAAGGATCGGCTAACTCCGTGCCAGCAGCCGCGGTAATACGGAGGATCCGAGCGTTATCCGGATTTATTGGGTTTAAAGGGTGCGCAGGCGGGAGATTAAGTCGGCGGTGAAATTTTGCAGCTTAACTGTAAAAGTGCCTTCGAAACTGGTTTCCTTGAGTGTAGATGAAGTAGGCGGAATTTGTGGTGTAGCGGTGAAATGCATAGATATCACGAGGAACTCCGATTGCGCAGGCAGCTTACTAAACTACTACTGACGCTCAGGCACGAAGGCGTGGGGATCAAACAGG

>OTU1067GTGGGGAATATTGGGCAATGGGGGAAACCCTGACCCAGCGACGCCGCGTGAGGGAAGACGGCCTTCGGGTTGTAAACCTCTTTTGTACGGGAAGAAGGACGTGACGGTACCGTGCGAATAAGTCCCGGCTAACTACGTGCCAGCAGCCGCGGTAATACGTAGGGGACGAGCGTTGTCCGGAATTACTGGGCGTAAAGGGCGCGCAGGCTGTAGATCAAGTCAGCTGTGAAATGTCTGGGCTTAACCCGGGCATGCGGTTGAAACTGATGTACTGGAGTGCTGTAGAGGCAAGTGGAATTCCCAGTGTAGCGGTGAAATGCGTAGATATTGGGAAGAACACCGGTGGCGAAGGCGACTTGCTGGGCAGCAACTGACGCTGAGGCGCGAAAGCCAGGGGAGCAAACGGG

>OTU1068GTGGGGAATTGTTCGCAATGGGCGCAAGCCTGACGACGCAACGCCGCGTGGAGGATGAAGATCTTCGGGTCGTAAACTCCTGTCGAGTGGGAAGAAACGCTTCGGGGCTAACAACCCCGGAGTTTGACGGTACCACTAAAGGAAGCCCCGGCTAACTCCGTGCCAGCAGCCGCGGTAATACGGGGGGGGCAAGCGTTGTTCGGAATTACTGGGCGTAAAGGGCTCGTAGGCGGCCAACTAAGTCGGATGTGAAATCCCCAGGCTCAACCTGGGAACTGCATCCGATACTGGATGGCTTGAATCCGGGAGAGGGATGCAGAATTCCAGGTGTAGCGGTGAAATGCGTAGATATCTGGAGGAATACCGGTGGCGAAGGCGGCATCCTGGACCGGCATTGACGCTGAGGAGCGAAAGCCAGGGGAGCAAACGGG

>OTU1074GTGGGGAATATTGGGCAATGGACGCAAGTCTGACCCAGCAACGCCGCGTGAAGGAAGAAGGCTTTCGGGTTGTAAACTTCTTTTAAGGGGGAAGAGCAGAAGACGGTACCCCTTGAATAAGCCACGGCTAACTACGTGCCAGCAGCCGCGGTAATACGTAGGTGGCAAGCGTTGTCCGGATTTACTGGGTGTAAAGGGCGTGCAGCCGGAGAGGCAAGTCAGATGTGAAATCCGCGGGCTCAACCCGCGAACTGCATTTGAAACTGCTTCCCTTGAGTATCGGAGAGGTAACCGGAATTCCTAGTGTAGCGGTGAAATGCGTAGATATTAGGAAGAACACCAGTGGCGAAGGCGGGTTACTGGACGACAACTGACGGTGAGGCGCGAAAGCGTGGGGAGCAAACAGG

>OTU1079GTGGGGAATATTGGACAATGGGGGAAACCCTGATCCAGCGACGCCGCGTGTGTGAAGAAGGCCTGCGGGTTGTAAAGCACTTTTAGTGGGGATGAAATGTGCAGGGCTAACACCTCTGCATTTTGACCTAACCCACAGAAAAAGCACCGGCTAACTCTGTGCCAGCAGCCGCGGTAATACAGAGGGTGCAAGCGTTAATCGGAATTACTGGGCGTAAAGCGTGCGTAGACGGTTACATAAGTCGGGTGTGAAAGCCCCGGGCTCAACCTGGGAATTGCATTCGAGACTGCGTAGCTAGGGTGCGGAAGAGGGAAGCGGAATTTCCGGTGTAGCGGTGAAATGCGTAGATATCGGAAGGAACACCAGTGGCGAAAGCGGCTTCCTGGTCCAGCACCGACGTTCAGGCACGAAAGCGTGGGGAGCAAACAGG

>OTU1082GTGGGGAATATTGGGCAATGGGCGCAAGCCTGACCCAGCAATGCCGCGTGAAGGAAGAAGGCCCTCGGGTTGTAAACTTCTTTTATCAGGGACGAAGGATGTGACGGTACCTGATGAATAAGCTCCGGCTAACTACGTGCCAGCAGCCGCGGTAATACGTAGGTGGCAAGCGTTATCCGGATTTACTGGGTGTAAAGGGCGTGTAGGCGGGAAAGCAAGTCAGATGTGAAAACCATGGGCTCAACCTGTGGCCTGCATTTGAAACTGTTTTTCTTGAGTACTGGAGAGGCAGACGGAATTCCTAGTGTAGCGGTGAAATGCGTAGATATTAGGAGGAACACCAGTGGCGAAGGCGGTCTGCTGGACAGCAACTGACGCTGAGGCGCGAAAGCGTGGGGAGCAAACAGG

>OTU1083GTAGGGAATATTGCGCAATGGGGGAAACCCTGACGCAGCGACGCCGCGTGAGTGATGAAGGCCTTCGGGTCGTAAAGCTCTGTTGTACGGGAAGAAGTTTGCAATGCTAATACCATTGCAAGTTGACGGTACCGTATAAGAAAGGATCGGCTAACTTCGTGCCAGCAGCCGCGGTAAGACGAGGGATCCTAGCGTTGTTCGGAATCACTGGGCGTAAAGCGTTTGTAGGCGGCTCTATAAGTCGGGTGTGAAATCCCATGGCTCAACCATGGAAGTGCATTCGAAACTGCAGAGCTTGAATATGGTAGAGGAGAGTAGAATTCCTGGTGTAGTGGTGAAATACGTAGATATCAGGAGGAATACCGGTGGCGAAGGCGGCTCTCTGGACCAATATTGACGCTGAGAAACGAAAGTGCGGGGATCAAACAGG

>OTU1089GTGGGGAATATTGGGCAATGGGGGAAACCCTGACCCAGCAACGCCGCGTGAAGGATGAAGGCTTTCGGGTTGTAAACTTCTTTGATCAGGGACGAAACAAATGACGGTACCTGAAGAACAAGCCACGGCTAACTACGTGCCAGCAGCCGCGGTAATACGTAGGTGGCAAGCGTTATCCGGATTTACTGGGTGTAAAGGGCGTGTAGGCGGTTCTGCAAGTCGGGTGTGAAATTTCCGGGCTTAACCCGGACGGGTCACCCGAAACTGCGGAACTTGAGTACTGGAGAGGATAGTGGAATTCCTAGTGTAGCGGTAAAATGCGTAGATATTAGGAGGAACACCAGTGGCGAAGGCGGCTATCTGGACAGTAACTGACGCTGAGGCGCGAAAGCGTGGGGAGCAAACAGG

>OTU1092GTGGGGAATTTTGGACAATGGGGGCAACCCTGATCCAGCCATTCCGCGTGAGTGAAGAAGGCCTTCGGGTTGTAAAGCTCTTTCAGCGGGAACGAAACGGTAGTCTCTAACATAGGCTGCTAATGACGGTACCCGCAGAAGAAGCACCGGCTAACTACGTGCCAGCAGCCGCGGTAATACGTAGGGTGCGAGCGTTAATCGGAATTACTGGGCGTAAAGCGTGCGCAGGCGGATTGTTAAGCAAGATGTGAAATCCCCGGGCTTAACCTGGGAATGGCATTTTGAACTGGCAATCTAGAGTGTGTCAGAGGGGGGTAGAATTCCACGTGTAGCAGTGAAATGCGTAGAGATGTGGAGGAATACCGATGGCGAAGGCAGCCCCCTGGGATAACACTGACGCTCATGTACGAAAGCGTGGGTAGCAAACAGG

>OTU1095GTGGGGAATATTGGGCAATGGGCGAAAGCCTGACCCAGCAACGCCGCGTGAGGGAAGAAGGTTTTCGGATTGTAAACCTCTGTCGCAGATGACGAAGAAAGTGACGGTAATCTGTGAGGAAGCCCCGGCTAACTACGTGCCAGCAGCCGCGGTAATACGTAGGGGGCGAGCGTTGTCCGGAATTACTGGGCGTAAAGGGTGCGCAGGCGGTTCTTTAAGTTGGATGTGAAATACCCGGGCTTAACTCGGGGGGTGCATTCAAAACTGGGGAACTAGAGTTCAGGAGAGGGAAGCGGAATTCCTAGTGTAGCGGTGAAATGCGTAGATATTAGGAGGAACATCAGTGGCGAAGGCGGCTTCCTGGACTGACACTGACGCTGAGGCACGAAAGCGTGGGGAGCAAACAGG

>OTU1109GTGGGGAATTGTTCGCAATGGGCGCAAGCCTGACGACGCAACGCCGCGTGGAGGACGAAGATCTTCGGGTCGTAAACTCCTGTCGAGCGGGAAGAACGCTTCCCGGGTGAATAATCCGGGGAATTGACGGTACCGCTAAAGGAAGCCCCGGCTAACTCCGTGCCAGCAGCCGCGGTAATACGGGGGGGGCAAGCGTTGTTCGGAATTACTGGGCGTAAAGGGCTCGTAGGCGGCCAACTAAGTCGGATGTGAAATCCCCAGGCTTAACCTGGGAACTGCATCCGATACTGGATGGCTTGAATCCGGGAGAGGGATGCAGAATTCCAGGTGTAGCGGTGAAATGCGTAGATATCTGGAGGAATACCGGTGGCGAAGGCGGCATCCTGGACCGGCATTGACGCTGAGTGAGCGAAAGCTAGGGGAGCAAACGGG

>OTU1110GTGGGGAATATTGCGCAATGGAGGCAACTCTGACGCAGCAATGCCGCGTGAGTGAGGAAGGGATTCGTCCCGTAAAGCTCTGTTGCCGGGGAAGAGGCAGACGGTACCCGGCGAGCAAGCCCCAGCCAACTACGTGCCAGCAGCTGCGGTAATACGTAGGGGGCGAGCGTTATCCGGATTTATTGGGCGTAAAGGGCACGCAGGCGGCTGTTTAGGCGCAGTGTAAAAGACCGGGGCCCAACCCCGGGAGGTCACGGCGAACCGGGCAGCTCGAGTACTGGAGAGGCAGGCAGAATTCCTGGTGTAGCGGTGAAATGCGCAGAGATCAGGAGGAATACCGGAGGCGAAGGCGGCCTGCCGGCCAGCTACTGACGCTCAGGTGCGAAAGCGTGGGGAGCGAACAGG

>OTU1111GTGGGGAATTGTTCGCAATGGGCGCAAGCCTGACGACGCAACGCCGCGTGGAGGATGAAGATTTTCGGATCGTAAACTCCTGTCGAACGGGACGAACAGATTGCGGGTTAACAGCCCGTAGTCCTGACGGTACCGTTAAAGGAAACCCCGGCTAACTCCGTGCCAGCAGCCGCGGTAATACGGGGGGGGTAAGCGTTGTTCGGAATTACTGGGCGTAAAGGGCTCGTAGGCGGCCAACTAAGTCGGACGTGAAATCCCTCAGCTTAACTGGGGAACTGCGTCCGATACTGGATGGCTTGGATTCGGGAGAGGGATGCGGAATTCCAGGTGTAGCGGTGAAATGCGTAGATATCTGGAGGAACACCGGTGGCGAAGGCGGCATCCTGGACCGATATTGACGCTGAGGAGCGAAAGCCAGGGGAGCAAACGGG

>OTU1123GTCGAGAATTTTTCTCAATGGGGGAAACCCTGAAGGAGCGACGCCGCGTGGGGGATGAAGGGCTTCGGCTCGTAAACCCCTGTCATTTGTGAGCAAGTGTTATTGTTTAAGAGATGATGACATTGATAGTAGCAAAAGAGGAAGGGACGGCTAACTCTGTGCCAGCAGCCGCGGTAATACAGAGGTCCCAAGCGTTGTTCGGAATCACTGGGCGTAAAGGGTGCGTAGGCGGTATGGTAAGACTTGGGTGAAATCTCCATGCTTAACGTGGAGGGAGCCTGGGAGACTGCCGTGCTAGAGGATTGGAAGGGAGACTGGAATTCTTGGAGTAGCAGTGAAATGCGTAGATATCAAGAGGAACACCAGTGGCGAAGGCGAGTCTCTGGACAAGACCTGACGCTGAGGCACGAAGGCCAGGGGATCAAACGGG

>OTU1124GTGGGGAATTTTGCGCAATGGGGGAAACCCTGACGCAGCAACGCCGCGTGGAGGATGAAGTCCCTTGGGACGTAAACTCCTTTCGACCGGGACGATTATGACGGTACCGGTGGAAGAAGCCCCGGCTAACTTCGTGCCAGCAGCCGCGGTAATACGAGGGGGGCAAGCGTTGTTCGGAATTATTGGGCGTAAAGGGCGCGTAGGCGGTGCGGTAAGTCACCTGTGAAAACTCTGGGCTCAACTCAGAGCCTGCAGGCGAAACTACCGTGCTTGAGTGCGGAAGAGGTGAGTGGAATTCCCGGTGTAGCGGTGAAATGCGTAGATATCGGGAGGAACACCTGTGGCGAAAGCGGCTCACTGGACCGCAACTGACGCTGAGGCGCGAAAGCTAGGGGAGCAAACAGG

>OTU1133GTAGGGAATCTTCCGCAATGGGCGAAAGCCTGACGGAGCAACGCCGCGTGAGTGATGAAGGTTTTCGGATCGTAAAGCTCTGTTGCCAAGGAAGAACGTCTCATAGAGTAACTGCTATGAGAGTGACGGTACTTGAGAAGAAAGCCCCGGCTAACTACGTGCCAGCAGCCGCGGTAATACGTAGGGGGCAAGCGTTGTCCGGAATTATTGGGCGTAAAGCGCGCGCAGGCGGTTCTTTAAGTCTGGTGTTTAAACCTGGGGCTCAACTTCAGGTCGCACTGGAAACTGGGGAACTTGAGTGCAGAAGAGGAGAGTGGAATTCCACGTGTAGCGGTGAAATGCGTAGATATGTGGAGGAACACCAGTGGCGAAGGCGACTCTCTGGGCTGTAACTGACGCTGAGGCGCGAAAGCGTGGGGAGCAAACAGG

>OTU1139GTGAGGAATATTGGGCAATGGACGGAAGTCTGACCCAGCCATGCCGCGTGCAGGAAGACGGCCTTATGGGTTGTAAACTGCTTTTATGTAGGGAAAAAGTTCATATTGCGATATGATTTGATGGTACTACAGGAATAAGAATCGGCTAACTCCGTGCCAGCAGCCGCGGTAATACGGAGGATTCGAGCGTTATCCGGATTTATTGGGTTTAAAGGGTGCGCAGGCGGACTATTAAGTCAGTGGTGAAATTTTCCGGCTCAACCGGGACAGTGCCATTGATACTGATAGACTAGAGTATAATTGACGTATGTGGAATGTAGCATGTAGCGGTGAAATGCATAGAGATGCTACAGAACACCGATAGCGAAGGCAGCATACGAAGCTATAACTGACGCTCATGCACGAAAGCGTGGGGATCAAACAGG

>OTU1146GTGGGGAATATTGGACAATGGGCGAAAGCCTGATCCAGCAATGCCGCGTGAGTGATGAAGGCCTTAGGGTTGTAAAGCTCTTTTACCCGGGATGATAATGACAGTACCGGGAGAATAAGCCCCGGCTAACTCCGTGCCAGCAGCCGCGGTAATACGGAGGGGGCTAGCGTTGTTCGGAATTACTGGGCGTAAAGCGCACGTAGGCGGCTTTGCAAGTCAGAGGTGAAAGCCCGGGGCTCAACCCCGGAATTGCCTTTGAGACTGCATCGCTAGAACGTCGGAGAGGTGAGTGGAATTCCGAGTGTAGAGGTGAAATTCGTAGATATTCGGAAGAACACCAGTGGCGAAGGCGACTCACTGGACGACCGTTGACGCTGAGGTGCGAAAGCGTGGGGAGCAAACAGG

>OTU1147GTGGGGAATTTTGGACAATGGACGCAAGTCTGATCCAGCCATGCCGCGTGCGGGAAGAAGGCCTTCGGGTTGTAAACCGCTTTTGTCAGGGAAGAAACGCTCTGGGTTAATACCTCGGGGTAATGACGGTACCTCGAGAGGAAGCACCGGCTAACTCTGTGCCAGCAGCCGCGGTAATACAGAGGGTGCAAGCGTTGCTCGGAATCATTGGGCGTAAAGGGCAAGTAGGTGGTCTCATTTGTCTCGTGTGAAATCCTTGGGCTTAACTCAAGAAGTGCGCGAGAAACGGTGAGACTCGAGTTCTGGAGAGGGTCGTGGAATTCCCGGTGTAGCGGTGAAATGCGTAGAGATCGGGAGGAACACCAGAGGCGAAGGCGGCGACCTGGACAGACACTGACACTCAACTGCGAAAGCGTGGGGAGCAAACAGG

>OTU1157GTGGGGAATCTTGCGCAATGGGCGAAAGCCTGACGCAGCGACGCCGCGTGGGGGAAGAAGGCTTTCGGGTTGTAAACCCCTTTCAGGAAGGACGAAGCCACTCGGGTTAATAGCCCAGAGGGTGACGGTACTTCCAGAAGAAGCCCCGGCTAACTACGTGCCAGCAGCCGCGGTAATACGTAGGGGGCAAGCGTTGTCCGGATTTATTGGGCGTAAAGAGCGTGTAGGCGGTCTGGTAGGTCCGTTGTGAAAACTCGAGGCTCAACCTCGAGACGCCGATGGAAACCCTCAGACTAGAGTCCGGAAGAGGAGAGTGGAATTCCTGGTGTAGCGGTGAAATGCGCAGATATCAGGAAGAACACCCGTGGCTAAGGCGGCTCTCTAGTACGGTACTGACGCTGAGACGCGAAAGCGTGGGGAGCGAACAGG

>OTU1165GTAAGGAATATTGGTCAATGGACGAAAGTCTGAACCAGCCATGCCGCGTGAAGGATGACTGCCCTCTGGGTTGTAAACTTCTTTTATAGGGGGCGAAAAAAGGGAATTCTTTCCCGTCTGACGGTACCCTATGAATAAGCACCGGCTAACTCCGTGCCAGCAGCCGCGGTAATACGGAGGGTGCAAGCGTTATCCGGATTCACTGGGTTTAAAGGGAGCGCAGGCGGACTTGTAAGTCAGTGGTGAAATACCTGAGCTTAACTCGGGAACTGCCATTGATACTATAGGTCTTGAATGTTGTTGAGGTTTGCGGAATGGGTCATGTAGCGGTGAAATGCATAGATATGACCCGGAACACCTATTGCGAAGGCAGCAGGCTAAACAAATATTGACGCTGAGGCTCGAAAGCGTGGGGATCAAACAGG

>OTU1172GTGGGGAATATTGGACAATGGGCGAAAGCCTGATCCAGCCATGCCGCGTGTGTGAAGAAGGTCTTCGGATTGTAAAGCACTTTAAGTTGGGAGGAAGGGCAGTAAATTAATACTTTGCTGTTTTGACGTTACCGACAGAATAAGCACCGGCTAACTCTGTGCCAGCAGCCGCGGTAATACAGAGGGTGCAAGCGTTAATCGGAATTACTGGGCGTAAAGCGCGCGTAGGTGGTTTGTTAAGTTGGATGTGAAAGCCCCGGGCTCAACCTGGGAACTGCGCTTGTAACTGGCAAGCTAGAGTGTGGCAGAGGGGGGTGGAATTCCACGTGTAGCAGTGAAATGCGTAGAGATGTGGAGGAACACCGATGGCGAAGGCAGCCCCCTGGGCTAACACTGACGCTCATGCACGAAAGCGTGGGGAGCAAACAGG

>OTU1188GTAGGGAATCTTCGGCAATGGGGGCAACCCTGACCGAGCAACGCCGCGTGAGTGAAGAAGGTTTTCGGATCGTAAAGCTCTGTTGTAAGTCAAGAACGAGTGTGAGAGTGGAAAGTTCACACTGTGACGGTAGCTTACCAGAAAGGGACGGCTAACTACGTGCCAGCAGCCGCGGTAATACGTAGGTCCCGAGCGTTGTCCGGATTTATTGGGCGTAAAGCGAGCGCAGGCGGTTTGATAAGTCTGAAGTTAAAGGCTGTGGCTCAACCATAGTTCGCTTTGGAAACTGTCAAACTTGAGTGCAGAAGGGGAGAGTGGAATTCCATGTGTAGCGGTGAAATGCGTAGATATATGGAGGAACACCGGTGGCGAAAGCGGCTCTCTGGTCTGTAACTGACGCTGAGGCTCGAAAGCGTGGGTAGCGAACAGG

>OTU1194GTGGGGAATTTTGGACAATGGGGGCAACCCTGATCCAGCCATCCCGCGTGTGCGATGAAGGCCTTCGGGTTGTAAAGCACTTTTGGCAGGAAAGAAACGGCACGGGCTAATATCCTGTGCAACTGACGGTACCTGCAGAATAAGCACCGGCTAACTACGTGCCAGCAGCCGCGGTAATACGTAGGGTGCAAGCGTTAATCGGAATTACTGGGCGTAAAGCGTGCGCAGGCGGTTCGGAAAGAAAGGTGTGAAATCCCAGGGCTTAACCTTGGAACTGCATTTTTAACTACCGGGCTAGAGTGTGTCAGAGGGAGGTGGAATTCCGCGTGTAGCAGTGAAATGCGTAGATATGCGGAGGAACACCGATGGCGAAGGCAGCCTCCTGGGATAACACTGACGCTCATGTGCGAAAGCGTGGGGAGCAAACAGG

>OTU1195GTGAGGAATATTGCGCAATGGGGGCAACCCTGACGCAGCGACGCCGCGTGAGTGAGGAAGGCCTTCGGGTCGTAAAGCTCTGTCAAGAGGGAAGAAATGCATGGTGGTTAATATCTGTCATGTTTGACGGTACCTCTAAAGGAAGCACCGGCTAACTCCGTGCCAGCAGCCGCGGTAATACGGAGGGTGCAAGCGTTGTTCGGAATCACTGGGCGTAAAGGGCGCGCAGGCGGTCTGATCAGTCAGATGTGAAAGCCCACGGCTTAACCGTGGAAGTGCATTTGAAACTGTCAGGCTTGAGTACCAGAGGGGAAAGTGGAATTCCCGGTGTAGAGGTGAAATTCGTAGATATCGGGAGGAATACCGGTGGCGAAGGCGACTTTCTGGCTGGATACTGACGCTGAGGCGCGAAAGCGTGGGGAGCAAACAGG

>OTU1203GTGGGGAATTTTCCGCAATGGGCGAAAGCCTGACGGAGTGACGCCGCGTGTGGGACGAAGGCCTTCGGGTCGTAAACCACTGTCAGTGGGAAAGATGGGTGTCGGCGCGATAACCGCCGGCATTTGACGGTACCTGCAGAGGAAGCCCCGGCTAACTCTGTGCCAGCAGCCGCGGTAAGACAGAGGGGGCAAGCGTTGCTCGGAATTACTGGGCGTAAAGGGCGCGTAGGCGGGATGGCAAGTCGGTCGTGAAATCCCTCGGCTCAACTGAGGATCGTCGATCGATACTGCCGTTCTTGAGGATTGCAGAGGAGACTGGAATTCCCGGTGTAGCGGTGAAATGTGTAGAGATCGGGAGGAACACCGGTGGCGAAGGCGGGTCTCTGGGCAATTCCTGACGCTGAGGCGCGAAAGCGTGGGGAGCAAACGGG

>OTU1206GTGGGGAATATTGCACAATGGGGGAAACCCTGATGCAGCAACGCCGCGTGAAGGATGAAGGTTTTCGGATTGTAAACTTCTTTAGTCGGGGACGAAAAAAATGACTGTACCCGAAGAATAAGCCACGGCTAACTACGTGCCAGCAGCCGCGGTAATACGTAGGTGGCAAGCGTTGTCCGGATTTACTGGGTGTAAAGGGCGTGTAGGCGGGTCTGCAAGTCAGATGTGAAATTTAGGGGCTCAACCCCTAAGCTGCAACTGAAACTGTGGATCTTGAGTACTGGAGAGGGAAGCGGAATTCCTAGTGTAGCGGTGAAATGCGTAGATATTAGGAGGAACACCAGTGGCGAAGGCGGCTTTCTGGACAGTAACTGACGCTGAGGCGCGAAAGCGTGGGGAGCAAACAGG

>OTU1208GTGGGGAATATTGGACAATGGGCGAAAGCCTGATCCAGCGACGCCGCGTGAGTGAAGAAGTATTTCGGTATGTAAAGCTCTATCAGCAGGGAAGAAAATGACGGTACCTGACTAAGAAGCCCCGGCTAACTACGTGCCAGCAGCCGCGGTAATACGTAGGGGGCAAGCGTTATCCGGATTTACTGGGTGTAAAGGGAGCGTAGACGGTTAAGCAAGTCTGAAGTGAAAGCCCGGGGCTCAACCCCGGTACTGCTTTGGAAACTGTTTGACTTGAGTGCAGGAGAGGTAAGTGGAATTCCTAGTGTAGCGGTGAAATGCGTAGATATTAGGAGGAACACCAGTGGCGAAGGCGGCTTACTGGACTGTAACTGACGTTGAGGCTCGAAAGCGTGGGGAGCAAACAGG

>OTU1210GTGGGGAATTTTGGACAATGGGGGCAACCCTGATCCAGCAATGCCGCGTGAGTGAAGAAGGCCTTCGGGTTGTAAAGCCCTTTCGGCGGGGACGATGATGACGGTACCCGCAGAAGAAGCCCCGGCTAACTTCGTGCCAGCAGCCGCGGTAATACGAAGGGGGCTAGCGTTGTTCGGAATGACTGGGCGTAAAGGGCGCGTAGGCGGCCGAGTAAGTCAGGCGTGAAAGCCCCGGGCTCAACCTGGGAACGGCGCTTGATACTGTTTGGCTTGAGGACGGGAGAGGATGGTGGAATTCCCAGTGTAGAGGTGAAATTCGTAGATATTGGGAAGAACACCGGTGGCGAAGGCGGCCATCTGGACCGTTTCTGACGCTGAGGCGCGAAAGCGTGGGGAGCAAACAGG

>OTU1211GTGGGGAATATTGCACAATGGGGGAAACCCTGATGCAGCAACGCCGCGTGAAGGATGAAGGCCCTTGGGTCGTAAACTTCTGTTCTAAGGGAAGATAATGACGGTACCTTAGGAGCAAGTCCCGGCTAACTACGTGCCAGCAGCCGCGGTAATACGTAGGGGGCAAGCGTTATCCGGAATTATTGGGCGTAAAGCGTACGTAGGTGGTTACCTAAGCACAGGGTTTAAGGCTATGGCTTAACTATAGTTCGCCTTGTGAACTGGGCTACTTGAGTGCAGGAGAGGAAAGCGGAATTCCTAGTGTAGCGGTGAAATGCGTAGATATTAGGAGGAACACCAGTGGCGAAGGCGGCTTTCTGGACTGTAACTGACACTGAGGTACGAAAGCGTGGGGAGCAAACAGG

>OTU1224GTGGGGAATATTGGACAATGGGGGCAACCCTGATCCAGCAATGCCGCGTGTATGAAGAAGGCCTTCGGGTTGTAAAGTACTTTCAGCGAGGAGGAAGGCGTCGTACTTAATACGTGCGACGATTGACGTTACTCGCAGAAGAAGCACCGGCTAACTCCGTGCCAGCAGCCGCGGTAATACGGAGGGTGCAAGCGTTAATCGGAATTACTGGGCGTAAAGCGCACGCAGGCGGTCTGTCATGTCGGATGTGAAATCCCCGGGCTCAACCTGGGAACTGCATTCGAAACGGGCAGGCTAGAGTCTTGTAGAGGGGGGTAGAATTCCAGGTGTAGCGGTGAAATGCGTAGAGATCTGGAGGAATACCGGTGGCGAAGGCGGCCCCCTGGACAGAGACTGACGCTCAGGTGCGAAAGCGTGGGGAGCAAACAGG

>OTU1225GTGGGGAATATTGCGCAATGGGGGGAACCCTGACGCAGCAACGCCGCGTGATTGAAGAAGGCCTTCGGGTTGTAAAGATCTTTAATCGGGGACGAAACATGACGGTACCCGAAGAATAAGCTCCGGCTAACTACGTGCCAGCAGCCGCGGTAATACGTAGGGAGCAAGCGTTATCCGGATTTACTGGGTGTAAAGGGCGTGTAGGCGGGCCTGCAAGTCAGAAGTGAAATCTGGGGGCTTAACCCCCAAACTGCTTTTGAAACTGCGGGTCTTGAGTGATGGAGAGGCAGGCGGAATTCCCAGTGTAGCGGTGAAATGCGTAGATATTGGGAGGAACACCAGTGGCGAAGGCGGCCTGCTGGACATTAACTGACGCTGAGGCGCGAAAGCGTGGGGAGCAAACAGG

>OTU1227GTAGGGAATATTGGACAATGGGCGGAAGCCTGATCCAGCCATGCCGCGTGCAGGAAGACTGCCCTATGGGTTGTAAACTGCTTTTATACGGGAAGAAACCGCCCTACGTGTAGGGCTCTGACGGTACCGTAAGAATAAGGATCGGCTAACTCCGTGCCAGCAGCCGCGGTAATACGGAGGATCCAAGCGTTATCCGGAATCATTGGGTTTAAAGGGTCCGTAGGTGGACGATTAAGTCAGAGGTGAAAGTCTGCGGCTCAACCGTAGAATTGCCTTTGATACTGGTTGTCTTGAATCATTGTGAAGTGGTTAGAATATGTAGTGTAGCGGTGAAATGCATAGATATTACATAGAATACCGATTGCGAAGGCAGATCACTAACAATGTATTGACACTGATGGACGAAAGCGTGGGTAGCGAACGGG

>OTU1228GTGGGGAATTGTTCGCAATGGGCGCAAGCCTGACGACGCAACGCCGCGTGGAGGATGAAGATCTTCGGGTCGTAAACTCCTGTCGAGCGGGAAGAACACCTTCCGGGTGAATATCCAGGAGGGCTGACGGTACCGCTAAAGGAAGCCCCGGCTAACTCCGTGCCAGCAGCCGCGGTAATACGGGGGGGGCAAGCGTTGTTCGGAATTACTGGGCGTAAAGGGCTCGTAGGCGGCCAACTAAGTCGGACGTGAAATCCCCAGGCTCAACCTGGGAACTGCGTCCGATACTGGATGGCTTGAATTCGGGAGAGGGATGCAGAATTCCAGGTGTAGCGGTGAAATGCGTAGATATCTGGAGGAATACCGGTGGCGAAGGCGGCATCCTGGACCGACATTGACGCTGATGAGCGAAAGCTAGGGGAGCAAACGGG

>OTU1240GTGGGGGATATTGGACAATGGGGGGAACCCTGATCCAGCGACGCCGCGTGAGTGAAGAAGTATCTCGGTATGTAAAGCTCTGTCAGCAGGGAAGAAGGAAGTGACGGTACCTGACCAAGAAGCCCCGGCTAACTACGTGCCAGCAGCCGCGGTAATACGTAGGGGGCAAGCGTTATCCGGAATTACTGGGTGTAAAGGGAGCGTAGACGGTTAAGTAAGTCTGGAGTGAAAGGCGGGGGCCCAACCCCCGGACTGCTCTGGAAACTATTTGACTGGAGTGCAGGAGAGGTAAGCGGAATTCCTGGTGTAGCGGTGAAATGCGTAGATATCAGGAGGAACATCGGTGGCGAAGGCGGCTTACTGGACTGTAACTGACGTTGAGGCTCGAAAGCGTGGGGAGCAAACAGG

>OTU1241GTGGGGAATATTGCACAATGGGGGAAACCCTGATGCAGCAACGCCGCGTGAGTGAAGAAGTATTTCGGTATGTAAAGCTCTATCAGCAGGGAAGAAGATGACGGTACCTGAGTAAGAAGCCCCGGCTAACTACGTGCCAGCAGCCGCGGTAATACGTAGGGGGCAAGCGTTATCCGGATTTACTGGGTGTAAAGGGAGCGTAGACGGCCAGACAAGTCTGAAGTGAAAGCCCGGGGCTCAACCCCGGGACTGCTTTGGAAACTGCCAGGCTAGAGTGCTGGAGAGGCAAGCGGAATTCCTAGTGTAGCGGTGAAATGCGTAGATATTAGGAAGAACACCAGTGGCGAAGGCGGCTTGCTGGACAGTAACTGACGTTGAGGCTCGAAAGCGTGGGGAGCAAACAGG

>OTU1245GTGGGGAATCTTAGACAATGGGGGAAACCCTGATCTAGCCATGCCGCGTGATCGATGAAGGCCTTAGGGTTGTAAAGATCTTTCAGTGGGGAAGATAATGACGGTACCCACAGAAGAAGCCCCGGCTAACTCCGTGCCAGCAGCCGCGGTAATACGGAGGGGGCTAGCGTTGTTCGGAATTACTGGGCGTAAAGCGCACGTAGGCGGACCGGAAAGTCAGAGGTGAAATCCCAGGGCTCAACCTTGGAACTGCCTTTGAAACTCCTGGTCTTGAGGTCGAGAGAGGTGAGTGGAATTCCGAGTGTAGAGGTGAAATTCGTAGATATTCGGAGGAACACCAGTGGCGAAGGCGGCTCACTGGCTCGATACTGACGCTGAGGTGCGAAAGCGTGGGGAGCAAACAGG

>OTU1246GTGGGGAATATTGCACAATGGGGGGAACCCTGATGCAGCGACGCCGCGTGAAGGAAGAAGTATCTCGGTATGTAAACTTCTATCGGCAGGGAAGAAAAAGGACGGTACCTGACTAAGAAGCCCCGGCTAACTACGTGCCAGCAGCCGCGGTAATACGTAGGGGGCGAGCGTTATCCGGATTCACTGGGCGTAAAGGGAGCGTAGACGGCTGGGCAAGCCTGATGTGAAAGGCGGGGGCGCAACCCTCGGACTGCATTGGGAACTGCCCGGCTGGAGCGCCGGAGGGGTAAGCGGAATTCCTGGTGTAGCGGTGAAATGCGTAGATATCAGGAGGAACACCGGTGGCGAAGGCGGCTTACTGGACGGCGACTGACGTTGAGGCTCGAAAGCGTGGGGAGCGAACAGG

>OTU1253GTGGGGAATCTTGCGCAATGGTCGAAAGACTGACGCAGCGACGCCGCGTGTGGGAGGACGCCTTTCGGGGTGTAAACCACTGTTGCCCGGGACGAATTGCGCTATTCGTAGCGCGTGACGGTACCGGGTGAGGAAGCACCGGCTAACTCCGTGCCAGCAGCCGCGGTAATACGGAGGGTGCGAGCGTTGTCCGGAATCATTGGGCGTAAAGGGCGCGTAGGTGGCCCGGTCAGTCCGTGGTGAAAGCGCGGGGCTCAACCCTGCGTCGGCCATGGATACTGCCGCGGCTCGAGCACTGTAGAGGCAGGCGGAATTCCGGGTGTAGCGGTGGAATGCGTAGAGATCCGGAAGAACACCGGTGGCGAAGGCGGCCTGCTGGGCAGTTTGGCTGACACTGAGGCGCGACAGCGTGGGGAGCAAACAGG

>OTU1256GTGAGGAATTTTGGGCAATGGGCGAAAGCCTGACCCAGCGACGCCGCGTGGAGGATGAAGGCCTTCGGGTCGTAAACTCCTGTTCTGGGGGAAGAAAACGGGATGCGTGAAGAATTCATCCCGCTGACGGTACCCCAGGAGAAAGCTCCGGCTAATTACGTGCCAGCAGCCGCGGTAATACGTAAGGAGCAAGCGTTGTCCGGATTTACTGGGCGTAAAGAGCTCGTAGGCGGCCGATCAGGTCTCGCGTAAAATCCCCCGGCTCAACCGGGGAGGGTCGCGGGAAACCGACCGGCTTGAGGACAGAAAAGGAAAGTGGAATTCCCGGTGTAGCGGTGAAATGCGTAGATATCGGGAGGAACACCAGTGGTGAAGACGGCTTTCTGGTCTGTTACTGACGCTGAGGAGCGAAAGCCAGGGGAGCGAACCGG

>OTU1263GTGAGGAATTTTGGGCAATGGGCGAAAGCCTGACCCAGCAACACCGCGTGGGGGATGACGGCCCTCGGGTTGTAAACCCCTTTTCTTGGGGAAGAATAATGACGGTACCTAAGGAATAAGCTTCGGCGGACTACGTGCCAGCAGCCGCGGTAATACGTAGGAAGCGAGCGTTATCCGGAATTACTGGGCGTAAAGGGCGCGTAGGTGGTCTTTCAAGTCGAATGTGAAATTTCACGGCTTAATCGGGAAGGGTCATTCGATACTGTTGGACTAGAGAGCAGCAGGGGGAAGCGGAATTCCCGGTGTAGTAGTGAAATGCGTAGATATCGGGAGGAACACCAGTGGCGAAGGCGGCTTCCTGGGCTGTTTCTGACACTGAGGCGCGAAAGTGTGGGTAGCGAACAGA

>OTU1266GTAGGGAATCTTCCACAATGGACGCAAGTCTGATGGAGCAACGCCGCGTGAGTGAAGAAGGTTTTCGGATCGTAAAGCTCTGTTGTTGGTGAAGAAGGACAAGGAAAGTAACTGATCTTTGTTTGACGGTAATCAACCAGAAAGTCACGGCTAACTACGTGCCAGCAGCCGCGGTAATACGTAGGTGGCAAGCGTTGTCCGGATTTATTGGGCGTAAAGCGAACGCAGGCGGGAAGATAAGTCAGCTGTGAAAGCCCCCAGCTTAACTGGGGAACTGCAGCTGAAACTATGTTTCTTGAGTGCAGAAGAGGAGAGTGGAACTCCATGTGTAGCGGTGGAATGCGTAGATATATGGAAGAACACCAGTGGCGAAGGCGGCTCTCTGGTCTGTAACTGACGCTGAGGTTCGAAAGCATGGGTAGCGAACAGG

>OTU1271GTGGGGAATCTTGCGCAATGGTCGAAAGACTGACGCAGCGACGCCGCGTGGAGGATGACGCCCTTCGGGGTGTAAACTCCTGTTGCCCGGGACGAACCTCCCCTTTCGAGGGGACTGACGGTACCGGGTGAGGAAGCACCGGCTAACTCTGTGCCAGCAGCCGCGGTAATACAGAGGGTGCGAGCGTTGTCCGGAATCACTGGGCGTAAAGGGCGCGTAGGCGGTTTCTCAAGCGTGCGGTGAAAGCTCAGGGCTCAACCCTGAGTCGGCCGTGCGAACTGGGAGACTGGAGCACGGTAGAGGCACATGGAATTCCGGGTGTAGCGGTGGAATGCGTAGAGATCCGGAAGAACACCGGTGGCGAAGGCGGTGTGCTGGGCCGTTGCTGACGCTGAGGCGCGACAGCGTGGGGAGCAAACAGG

>OTU1278GTTAGGAATTTTGCGCAATGGAGGAAACTCTGACGCAGCCACGCCGCGTGAGGGATGAAGGCCTTCGGGTCGTAAACCTCTTTTCTCAGGGAAGACACCCGACGGTACCTGAGGAATAAGCAACGGCTAACTACGTGCCAGCAGCCGCGGTAATACGTAGGTTGCGAGCGTTGTCCGGATTTACTGGGCGTAAAGCGCGCGCAGGTGGTCCTGAAAGTCGAGCGTGAAAGCTCCCGGCCCAACTGGGAGAGGCCGTTCGAAACTTCAGGACTTGAAGGCGGGAGAGGCAAATGGAATTTCCGGTGTAGCGGTAAAATGCGTAGATACCGGAAGGAACACCAGTGGCGAAAGCGATTTGCTGGCCCGCCCTTGACACTCAGGCGCGAAAGCTAGGGGAGCGACCGGG

>OTU1282GTGAGGAATATTGGTCAATGGGCGCATGCCTGAACCAGCCAAGTCGCGTGAGGGACGACGGCCCTACGGGTTGTAAACCTCTTTTGTCGGGGAGCAAGACCCGCCTCGTGCAGGCGGAGCGAGAGTACCCGAAGAAAAAGCATCGGCTAACTCCGTGCCAGCAGCCGCGGTAATACGGAGGATGCGAGCGTTATCCGGATTTATTGGGTTTAAAGGGTGCGCAGGCGGACCATCAAGTCAGCGGTAAAATCGTGGGGCCCAACCCCATCCCGCCGTTGAAACTGGCGGTCTTGAGTGAGCGAGAAGTATGCGGAATGCGTGGTGTAGCGGTGAAATGCATAGATATCACGCAGAACCCCGATTGCGAAGGCAGCATACCGGTGCTCAACTGACGCTGAGGCACGAAAGCGTGGGGATCAAACAGG

>OTU1300GTGGGGAATATTGGACAATGGGCGAAAGCCTGATCCAGCAATGCCGCGTGAGTGATGAAGGCCTTAGGGTTGTAAAGCTCTTTTACCCGGGAAGATAATGACTGTACCGGGAGAATAAGCCCCGGCTAACTCCGTGCCAGCAGCCGCGGTAATACGGAGGGGGCTAGCGTTGTTCGGAATTACTGGGCGTAAAGCGCACGTAGGCGGCTTTACAAGTCAGGGGTGAAAGCCCGCGGCTCAACCGCGGAATTGCCCTTGAGACTGTATCGCTTGAACACGGGAGAGGTGAGTGGAATTCCGAGTGTAGAGGTGAAATTCGTAGATATTCGGAAGAACACCAGTGGCGAAGGCGGCTCACTGGACCGTTGTTGACGCTGAGGTGCGAAAGCGTGGGGAGCAAACAGG

>OTU1305GTGGGGAATTTTGGACAATGGGGGAAACCCTGATCCAGCCATTCCGCGTGAGTGAAGAAGGCCTTCGGGTTGTAAAGCTCTTTTGTCCGGAACGAAACGGTGGACGTGAATATCGTCCGCTACTGACGGTACCGGAAGAATAAGCACCGGCTAACTACGTGCCAGCAGCCGCGGTAATACGTAGGGTGCGAGCGTTAATCGGAATTACTGGGCGTAAAGCGTGCGCAGGCGGTTTTGCAAGCCGGATGTGAAATCCCCGGGCTTAACCTGGGAATGGCATTTGGGACTGCAAGGCTGGAGTGCGGCAGAGGAGACTGGAATTCCTGGTGTAGCAGTGAAATGCGTAGAGATGCGGAGGAACACCGATGGCGAAGGCAGCCCCCTGGGCTGACACTGACGCTCAGGCACGAAAGCGTGGGGAGCAAACAGG

>OTU1312GCAGGGAATCTTGCGCAATGGGCGAAAGCCTGACGCAGCAACGCCGCGTGGGGGATGACGGCCTTCGGGTTGTAAACCCCTTTCAGGAGGGAAGAAATTGACGGTACCTCCAGAAGAAGCCCCGGCCAACTACGTGCCAGCAGCCGCGGTAATACGTAGGGGGCGAGCGTTGTCCGGAATCATTGGGCGTAAAGAGCTCGTAGGCGGTTCAGAGAGTCGGCTGTGAAAGACCAAGGCTCAACCTTGGAGCGGCAGTCGATACTTCTGTGACTCGAGTCCGGTAGAGGAGAATGGAATTCCCGGTGTAGCGGTGAAATGCGCAGATATCGGGAGGAACACCAGTAGCGAAGGCGGTTCTCTGGGCCGGTACTGACGCTGAGGAGCGAAAGCGTGGGGAGCAAACAGG

>OTU1334GTGGGGAATATTGCACAATGGGCGAAAGCCTGATGCAGCGACGACGCGTGAGGGATGACGGCCTTCGGGTTGTAAACCTCTTTCAGCACGGAAGAAGCGAAAGTGACGGTACGTGCAGAAGAAGCGCCGGCTAACTACGTGCCAGCAGCCGCGGTAATACGTAGGGCGCAAGCGTTGTCCGGAATTATTGGGCGTAAAGAGCTCGTAGGCGGTTTGTCGCGTCTGCTGTGAAAGCCCGGGGCTTAACCCCGTGGTGTGCAGTGGGTACGGGCAGACTAGAGTGCAGTAGGGGAGACTGGAATTCCTGGTGTAGCGGTGAAATGCGCAGATATCAGGAGGAACACCGATGGCGAAGGCAGGTCTCTGGGCTGTTACTGACGCTGAGGAGCGAAAGCATGGGGAGCGAACAGG

>OTU1340GTCGAGAATAGTCTACAATGGACGAAAGTCTGATAGTGCGACGCCGCGTGAACGAAGAATCCCTTCGGGGTGTAAAGTTCTTTTATATGTGAGCAATGCTTTTTATGTGAATAGCATGAGAAGAGAGATATTAGCATATGAATAAGCAACGGCTAACTCCGTGCCAGCAGCCGCGGTAACACGGGGGTTGCAAGCGTTGTCCGGAATTACTAGGCGTAAAGGGCAGGTAGGCGGATCTATAAGTTTGATGTTAAAGGCAGCGGCTCACCCGCTGTAGGGCATTAGATACTGTAGATCTGGAATACGGTTGGGGAAGACAGAATTCCTGGTGTAGCGGTGGAATGCGCAGAGATCAGGAGGAATACCGAAGGCGAAGGCAGTCTTCTAAGCCGTTATTGACGCTAAACTGCGAAGGTGTGGGTATCAAACAGG

>OTU1346GTAGGGAATCTTCCGCAATGGACGAAAGTCTGACGGAGCGACGCCGCGTGAGCGAAGAAGGTCTTCGGATCGTAAAGCTCTGTTGTTAGGGAAGAAGAAGCGCCGTTCGAAGAGGGCGGCGCGGTGACGGTACCTAACGAGAAAGCCCCGGCTAACTACGTGCCAGCAGCCGCGGTAATACGTAGGGGGCGAGCGTTGTCCGGAATTATTGGGCGTAAAGCGCGCGCAGGCGGTCCCTTAAGTCTGATGTGAAAGCCCACGGCTTAACCGTGGAGGGTCATTGGAAACTGGGGGACTTGAGTGCAGAAGAGGAGAGCGGAATTCCACGTGTAGCGGTGAAATGCGTAGAGATGTGGAGGAACACCAGTGGCGAAGGCGGCTCTCTGGTCTGTAACTGACGCTGAGGCGCGAAAGCGTGGGGAGCAAACAGG

>OTU1349GTGGGGAATTTTGGACAATGGGCGAAAGCCTGATCCAGCAATGCCGCGTGCAGGATGAAGGCCTTCGGGTTGTAAACTGCTTTTGTACGGAACGAAAAGCTTCTGGTTAATACCTGGGAGTCATGACGGTACCGTAAGAATAAGCACCGGCTAACTACGTGCCAGCAGCCGCGGTAATACGTAGGGTGCAAGCGTTAATCGGAATTACTGGGCGTAAAGCGTGCGCAGGCGGTTTTGTAAGACAGAGGTGAAATCCCCGGGCTCAACCTGGGAACTGCCTTTGTGACTGCAAGGCTAGAGTACGGCAGAGGGGGGTGGAATTCCGCGTGTAGCAGTGAAATGCGTAGATATGCGGAGGAACACCGATGGCGAAGGCAACCCCCTGGGCCTGTACTGACGCTCATGCACGAAAGCGTGGGGAGCAAACAGG

>OTU1351GTCGGGAATATTGCGCAATGGAGGAAACTCTGACGCAGTGACGCCGCGTGCAGGAAGAAGGTTTTCGGATTGTAAACTGCTTTAGACAGGGAAGAAACAAGACAGTACCTGTAGAATAAGCTCCGGCTAACTACGTGCCAGCAGCCGCGGTAATACGTAGGGAGCAAGCGTTATCCGGATTTATTGGGTGTAAAGGGTGCGTAGACGGGAATGCAAGTTGGTTGTGAAATCCCTCGGCTCAACCGAGGAATTGCAACCAAAACTATATTTCTTGAGTGCTGGAGAGGAAAGTGGAATTCCTAGTGTAGCGGTGAAATGCGTAGATATTAGGAGGAACACCAGTGGCGAAGGCGACTTTCTGGACAGTAACTGACGTTGAGGCACGAAAGTGTGGGGAGCAAACAGG

>OTU1352GTGGGGAATATTGGACAATGGGCGCAAGCCTGATCCAGCCATGCCGCGTGAGTGATGAAGGCCTTAGGGTTGTAAAGCTCTTTCACCGGAGAAGATAATGACGGTATCCGGAGAAGAAGCCCCGGCTAACTTCGTGCCAGCAGCCGCGGTAATACGAAGGGGGCTAGCGTTGTTCGGAATTACTGGGCGTAAAGCGCACGTAGGCGGATATTTAAGTCAGGGGTGAAATCCCAGAGCTCAACTCTGGAACTGCCTTTGATACTGGGTATCTTGAGTATGGAAGAGGTAAGTGGAATTCCGAGTGTAGAGGTGAAATTCGTAGATATTCGGAGGAACACCAGTGGCGAAGGCGGCTTACTGGTCCATTACTGACGCTGAGGTGCGAAAGCGTGGGGAGCAAACAGG

>OTU1356GTGAGGAATATTGGTCAATGGGCGGAAGCCTGAACCAGCCAAGTCGCGTGAGGGAATAAGGCCCTACGGGTCGTAAACCTCTTTTGTCAGGGAGCAAAGCTTGGTACGTGTACCGAGAAGGAGAGTACCTGAAGAAAAAGCATCGGCTAACTCCGTGCCAGCAGCCGCGGTAATACGGAGGATGCGAGCGTTATCCGGATTTATTGGGTTTAAAGGGTGCGTAGGCGGGCTTTTAAGTCAGCGGTCAAATTGAGGGGCTCAACCCCTTCCCGCCGTTGAAACTGGGAGTCTTGAGTGAGCGAGAAGTAAGCGGAATGCGTGGTGTAGCGGTGAAATGCATAGATATCACGCAGAACCCCGATTGCGAAGGCAGCTTACCGGCGCTCAACTGACGCTGAGGCACGAAAGTGTGGGTATCGAACAGG

>OTU1383GTGGGGAATTTTGGACAATGGGGGCAACCCTGATCCAGCCATTCCGCGTGAGTGAAGAAGGCCTTCGGGTTGTAAAGCTCTTTTGTCCGGAACGAAACGGGTGGTGTTAACAGCGCCATCTACTGACGGTACCGGAAGAATAAGCACCGGCTAACTACGTGCCAGCAGCCGCGGTAATACGTAGGGTGCGAGCGTTAATCGGAATTACTGGGCGTAAAGCGTGCGCAGGCGGTTTTGCAAGCCGGATGTGAAATCCCCGGGCTTAACCTGGGAATGGCATTTGGGACTGCAAGGCTGGAGTGCGGCAGAGGAGACTGGAATTCCTGGTGTAGCAGTGAAATGCGTAGATATCAGGAGGAACACCGATGGCGAAGGCAGGTCTCTGGGCTGACACTGACGCTCATGCACGAAAGCGTGGGGAGCAAACAGG

>OTU1417GTAGGGAATCTTCCGCAATGGGCGAAAGCCTGACGGAGCAATGCCGCGTGAGTGATGAAGGTTTTCGGATCGTAAAGCTCTGTTGCCAGGGAAGAACGCTTGGGAGAGTAACTGCTCTCAAGGTGACGGTACCTGAGAAGAAAGCCCCGGCTAACTACGTGCCAGCAGCCGCGGTAATACGTAGGGGGCAAGCGTTGTCCGGAATTATTGGGCGTAAAGCGCGCGCAGGCGGTCCTTTAAGTCTGATGTGAAAGCCCTCGGCTCAACCGAGGAGGGTCATTGGAAACTGGAGGACTTGAGTGCAGAAGAGGAAAGTGGAATTCCAAGTGTAGCGGTGAAATGCGTAGATATGTGGAGGAACACCAGTGGCGAAGGCGACTCTCTGGGCTGTAACTGACGCTGAGGCGCGAAAGCGTGGGGAGCAAACAGG

>OTU1418GTAGGGAATCTTCCACAATGGGCGAAAGCCTGATGGAGCAACGCCGCGTGAGTGAAGAAGGTTTTCGGATCGTAAAACTCTGTTGTAAGGGAAGAACAAGTACAGTAGTAACTGGCTGTACCTTGACGGTACCTTATTAGAAAGCCACGGCTAACTACGTGCCAGCAGCCGCGGTAATACGTAGGTGGCAAGCGTTGTCCGGAATTATTGGGCGTAAAGCGCGCGCAGGCGGTCATTTAAGTCTGGTGTTTAATCCCGGGGCTCAACCCCGGATCGCACTGGAAACTGGATGACTTGAGTGCAGAAGAGGAAAGTGGAATTCCAAGTGTAGCGGTGAAATGCGTAGAGATTTGGAGGAACACCAGTGGCGAAGGCGACTTTCTGGTCTGTAACTGACGCTGAGGCGCGAAAGCGTGGGGAGCAAACAGG

>OTU1424GTCGAGAATTTTTCACAATGGGCGCAAGCCTGATGGAGCGACGCCGCGTGGGGGATGAATGGCTTCGGCCCGTAAACCCCTGTCATTCGCGATCAAACCTCGTTATTTAAAAGATGACGAGCTGATAGTAGCGGAAGAGGAAGGGACGGCTAACTCTGTGCCAGCAGCCGCGGTAATACAGAGGTCCCAAGCGTTGTTCGGATTCACTGGGCGTAAAGGGTGCGTAGGTGGTGAGGTAAGTCGGATGTGAAATCTCGGAGCTCAACTCCGAAACGGCATTGGAAACTACCTTGCTCGAGGATTGGAGGGGGGACTGGAATACTTGGTGTAGCAGTGAAATGCGTAGATATCAAGTGGAACACCAGTGGCGAAGGCGAGTCCCTGGACAATTCCTGACACTGAGGCACGAAAGCTAGGGGAGCAAACAGG

>OTU1438GTGGGGAATATTGCGCAATGGAGGCAACTCTGACGCAGCAATGCCGCGTGAGTGAAGAAGGGATTCGTTCCGTAAAGCTCTGTTACCGGGGAAGAGAGAGGACGGTACCCGGTGAGCAAGCCCCAGCCAACTACGTGCCAGCAGCTGCGGTAATACGTAGGGGGCGAGCGTTATCCGGATTTATTGGGCGTAAAGGGCACGCAGGCGGTTATTCAGGTGTGATGTTCAAGGGTGGGGCTCAACCCCGCAGGTCATGGCAAACCGGATAACTCGAGTAATGTCGGGGCAGACAGAATTCTTGGTGTAGCGGTGAAATGCGCAGAGATCAGGAGGAATACCAGAGGCGAAGGCGGTCTGCCAGGCACTTACTGACGCTGAGGTGCGAAAGCGTGGGGAGCAAACAGG

>OTU1446GTGGGGAATATTGCACAATGGGGGAAACCCTGATGCAGCGACGCCGCGTGAGCGAAGAAGTATTTCGGTATGTAAAGCTCTATCAGCAGGGAAGAAACTGACGGTACCTGACTAAGAAGCACCGGCTAAATACGTGCCAGCAGCCGCGGTAATACGTATGGTGCAAGCGTTATCCGGATTTACTGGGTGTAAAGGGAGCGTAGACGGATAGGCAAGTCTGGAGTGAAAGCCCGGGGCTCAACCCCGGGACTGCTTTGGAAACTGTTTATCTAGAGTGCTGGAGAGGCAAGTGGAATTCCTAGTGTAGCGGTGAAATGCGTAGATATTAGGAGGAACACCAGTGGCGAAGGCGGCTTGCTGGACAGTAACTGACGTTGAGGCTCGAAAGCGTGGGGAGCAAACAGG

>OTU1451GTGGGGAATTTTGGACAATGGGGGAAACCCTGATCCAGCCATGCCGCGTGCGGGAAGAAGGCCTTCGGGTTGTAAACCGCTTTTGGCAGGAACGAAACGGCGCGCGCCAATACCGCGCGTTAATGACGGTACCTGCAGAATAAGCACCGGCTAACTACGTGCCAGCAGCCGCGGTAATACGTAGGGTGCAAGCGTTAATCGGAATTACTGGGCGTAAAGCGTACGCAGGCGGCTATGCAAGACAGGTGTGAAATCCCCGGGCTCAACCTGGGAACTGCACTTGTGACTGCATGGCTAGAGTCCGTAAGAGGGGGGTGGAATTCCACGTGTAGCAGTGAAATGCGTAGAGATGTGGAGGAACACCGATGGCGAAGGCAGCCCCCTGGGATGAGACTGACGCTCATGTACGAAAGCGTGGGGAGCAAACGGG

>OTU1463GTGAGGAATATTGGTCAATGGCCGAGAGGCTGAACCAGCCAAGTCGCGTGAGGGATGACGGCCCTACGGGTTGTAAACCTCTTTTGTCAGGGAGCAAAGAACGCCACGTGTGGCGTCTCGAGAGTACCTGAAGAAAAAGCATCGGCTAACTCCGTGCCAGCAGCCGCGGTAATACGGAGGATGCGAGCGTTATCCGGATTTATTGGGTTTAAAGGGTGCGCAGGCGGACTGTCAAGTCAGCGGTAAAATTGCGGGGCTCAACCCCGTCGAGCCGTTGAAACTGGCGGTCTTGAGTGAGCGAGAAGTATGCGGAATGCGTGGTGTAGCGGTGAAATGCATAGATATCACGCAGAACTCCGATTGCGAAGGCAGCATACCGGCTCCACACTGACGCTGAGGCACGAAAGCGTGGGTATCGAACAGG
[truncated: 15,471 more chars]
